# Construction of 1,2,3-Triazole-Embedded Polyheterocyclic Compounds via CuAAC and C–H Activation Strategies

**DOI:** 10.3390/molecules30122588

**Published:** 2025-06-13

**Authors:** Antonia Iazzetti, Dario Allevi, Giancarlo Fabrizi, Yuri Gazzilli, Antonella Goggiamani, Federico Marrone, Francesco Stipa, Karim Ullah, Roberta Zoppoli

**Affiliations:** 1 Dipartimento di Scienze Biotecnologiche di Base, Cliniche Intensivologiche e Perioperatorie, Università Cattolica del Sacro Cuore, L.go Francesco Vito 1, 00168 Rome, Italy; antonia.iazzetti@unicatt.it (A.I.); dario.allevi@unicatt.it (D.A.); 2 Policlinico Universitario ‘A. Gemelli’ Foundation-IRCCS, 00168 Rome, Italy; 3 Dipartimento di Chimica e Tecnologie del Farmaco, Sapienza, Università di Roma, P. le A. Moro 5, 00185 Rome, Italy; yuri.gazzilli@uniroma1.it (Y.G.); antonella.goggiamani@uniroma1.it (A.G.); federico.marrone@uniroma1.it (F.M.); f.stipa@outlook.it (F.S.); roberta.zoppoli@uniroma1.it (R.Z.)

**Keywords:** palladium catalysis, 1,2,3-triazoles, triazoloquinolines, triazoloazepinoindoles, CuAAC, C–H bond activation

## Abstract

Over the past two decades, the copper(I)-catalyzed azide–alkyne 1,3-dipolar cycloaddition (CuAAC), commonly known as click chemistry, and C–H bond activation have gained significant attention and have emerged as key synthetic methodologies. In our efforts to synthesize fused nitrogen-containing heterocycles, we developed a palladium-catalyzed protocol for the synthesis of functionalized 7,10-dihydropyrrolo[3,2,1-ij][1,2,3]triazolo[4,5-c]quinolines and 5,8-dihydrobenzo[3,4][1,2,3]triazolo[4′,5′:5,6]azepino[1,2-a]indoles from suitable bromo-substituted *N*-propargyl-indoles. The reaction conditions demonstrate broad functional group compatibility including halogen, alkoxyl, cyano, ketone, and ester, affording the target compounds in good to high yields.

## 1. Introduction

The strategic incorporation of multiple pharmacophores, particularly heterocycles, into single molecular frameworks is a powerful approach in designing novel therapeutic agents. This strategy often yields hybrid molecules with enhanced biological properties. Among these, triazole-fused polycyclic heterocycles have attracted significant interest due to their broad spectrum of pharmacological activities, including anticancer, antibacterial, anti-HIV, antifungal, and anti-inflammatory effects [1,2,3,4,5,6,7]. The triazole unit is particularly valuable for its metabolic stability, hydrogen-bonding capabilities, and function as a non-classical amide isostere [8]. The widespread relevance of triazole-fused polycyclic heterocycles in medicinal chemistry and interdisciplinary research has spurred efforts to develop efficient and sustainable synthetic methodologies [9,10,11].

Transition metal-catalyzed strategies have emerged as indispensable tools for constructing complex architectures, offering advantages such as broad functional group tolerance, operational simplicity, and high efficiency. In particular, the copper(I)-catalyzed azide–alkyne cycloaddition (CuAAC), a hallmark of click chemistry, has become the method of choice for triazole formation due to its regioselectivity, modularity, high efficiency, and facile nature [12,13,14,15]. Triazole derivatives have found diverse applications in organic chemistry, medicinal chemistry, and functional materials, with numerous reports highlighting their versatility [16,17,18,19,20,21]. The C–H bond activation is an attractive alternative to “classical” cross-coupling reactions because it is a process capable of forming carbon–carbon, carbon–nitrogen, or carbon–oxygen bonds via the direct functionalization of a carbon–hydrogen bond with transition metal catalysis, avoiding the preparation and use of organometallic reagents [22,23].

**Figure 1:**
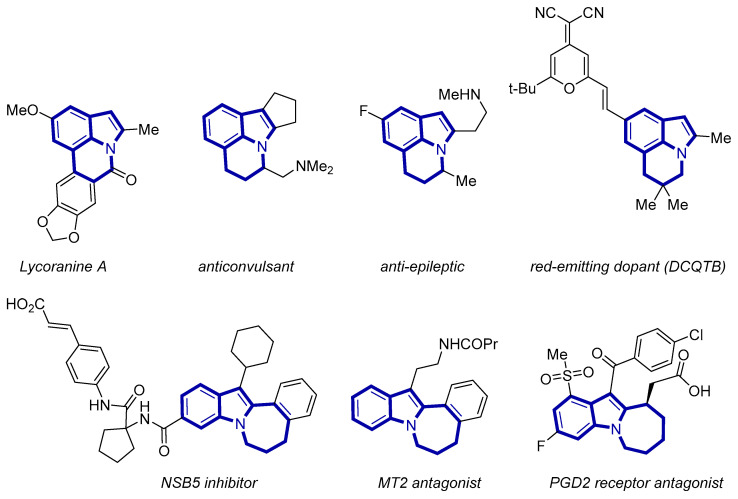
Natural products, biologically active compounds, and red-emitting dopants containing 5,6-dihydro-4*H*-pyrrolo [3,2,1-*ij*]quinoline and azepino[1,2-*a*]indole scaffold.

According to our background in the construction of heterocycles [24,25,26,27], here we report an efficient process providing ready access to hybrid molecules incorporating key pharmacophores (Figure 1), including pyrroloquinoline [28,29,30] and azepino[1,2-*a*]indole [31,32,33] cores linked to triazole moieties by combining CuAAC and palladium-catalyzed C–H bond activation. Furthermore, the pyrroloquinoline moiety has also demonstrated potential in material chemistry as red-emitting dopants (DCQTB) for organic light-emitting diodes [34].

**Scheme 1:**
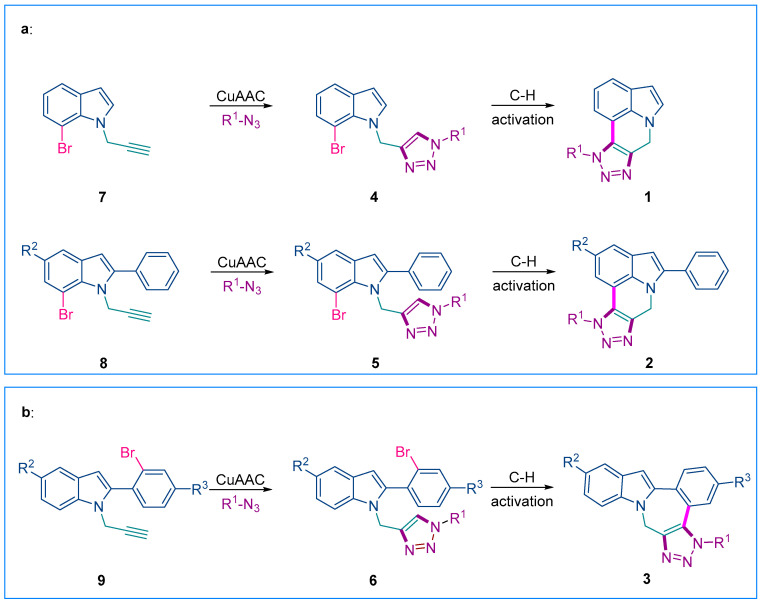
This work. (**a**): construction of 7,10-dihydropyrrolo[3,2,1-*ij*][1,2,3]triazolo[4,5-c]quinoline nucleus; (**b**): construction of 5,8-dihydrobenzo[3,4][1,2,3]triazolo[4′,5′:5,6]azepino[1,2-a]indole nucleus.

Notably, by designing opportunely the starting materials, we successfully synthesized 10-substituted-**1**, 5,10-disubstituted-7,10-dihydropyrrolo[3,2,1-*ij*][1,2,3]triazolo[4,5-*c*]quinolines **2** (Scheme 1a) and 5-substituted-5,8-dihydrobenzo[3,4][1,2,3]triazolo[4′,5′:5,6]azepino[1,2-*a*]indoles **3** (Scheme 1b).

## 2. Results and Discussion

**Scheme 2:**
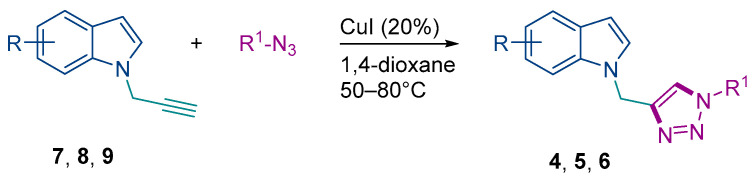
CuAAC with functionalized halo *N*-propargyl indoles **7**, **8**, and **9**.

We selected 1-((1-substituted-1*H*-1,2,3-triazol-4-yl)methyl)-1*H*-indoles **4**, **5**, and **6** as the starting materials for the palladium-catalyzed annulation. The synthesis of the triazole core in precursors of types **4**, **5**, and **6** was carried out via a copper-catalyzed azide–alkyne cycloaddition (CuAAC) on functionalized halo *N*-propargyl indoles, specifically designed for this purpose, under reaction conditions previously optimized by our group for structurally related substrates (Scheme 2) [26]. The desired products were obtained in good yields (see Supplementary Materials).

**Scheme 3:**
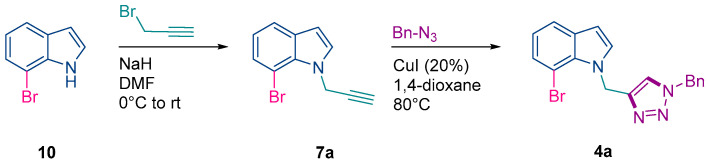
Preparation of 1-((1-benzyl-1H-1,2,3-triazol-4-yl)methyl)-7-bromo-1H-indole **4a**.

To evaluate our working hypothesis, we initiated our investigation with the cyclization of 1-((1-benzyl-1*H*-1,2,3-triazol-4-yl)methyl)-7-bromo-1*H*-indole **4a** into 10-benzyl-7,10-dihydropyrrolo[3,2,1-*ij*][1,2,3]triazolo[4,5-*c*]quinoline **1a** as a model system to optimize reaction conditions. It is worth noting that **4a** was readily synthesized in two steps from commercially available 7-bromo-1*H*-indole **10** via *N*-propargylation, followed by a CuAAC reaction with benzyl azide (Scheme 3).

Different reaction parameters were screened, including transition metal catalysts, ligands, solvents, and temperatures (Table 1). For our initial attempt, we employed conditions previously developed in our copper-catalyzed synthesis of heterocycles [35], expecting that copper catalysis could enable a domino or one-pot transformation of **7a** to **1a** [CuAAC/C–H activation]. However, despite testing various Cu catalysts, solvents, and bases (Table 1, entries 1–5), no reaction occurred (Table 1, entries 1–5). Consequently, we switched to palladium catalysis for the intramolecular C–H activation. Using Pd(OAc)_2_ as the palladium source, DavePhos as the ligand, and CsOAc as the base in dry DMF at 120 °C, the desired annulation product **1a** was obtained in 14% yield, while the major product was the hydrodebromination derivative 1-((1-benzyl-1*H*-1,2,3-triazol-4-yl)methyl)-1*H*-indole, isolated in 42% yield (Table 1, entry 6) [36,37,38]. Interestingly, replacing the ligand with PPh_3_ significantly improved the reaction outcome, affording 10-benzyl-7,10-dihydropyrrolo[3,2,1-*ij*][1,2,3]triazolo[4,5-*c*]quinoline **1a** in 68% yield. Encouraged by this result, we systematically optimized the reaction parameters (Table 1, entries 8–13). Notably, the combination of Pd(OAc)_2_, PPh_3_, and CsOAc in dry DMSO provided the best outcome, affording **1a** in excellent 80% yield (Table 1, entry 8). Lowering the temperature (Table 1, entries 9–10) or replacing DMSO with MeCN or 1,4-dioxane led to decreased yields. Furthermore, reducing the palladium catalyst load resulted in less efficient cyclization (Table 1, entry 13).

Once the optimal reaction conditions were established, we investigated the scope and generality of this method. Initially, we modified only the *N*-substitution on the triazole ring in **4**, introducing aryl groups with electron-donating and electron-withdrawing substituents (**4b**–**g**). The required starting materials were prepared according to Scheme 3, employing various aryl azides instead of benzyl azide. The resulting 10-aryl-7,10-dihydropyrrolo[3,2,1-*ij*][1,2,3]triazolo[4,5-*c*]quinoline derivatives **1b**–**g** were obtained in good to excellent yields (Table 2, 70–91% yields). Subsequently, the newly designed reaction procedure was tested on the gram scale to determine its synthetic scalability, producing **1a** in 69% yield.

We further investigated a domino strategy involving the simultaneous addition of all reagents and catalysts at the beginning of the reaction. However, this approach proved inefficient, resulting in a complex reaction mixture. Consequently, a sequential protocol was adopted: the CuAAC reaction was first performed in DMSO, and, without intermediate workup, the catalyst required for C–H activation was subsequently introduced. This protocol afforded the final compound **1a** in 45% yield, along with the recovery of the starting triazole derivative **4a** in 25% yield after 24 h. Finally, a one-pot strategy incorporating an intermediate workup following the CuAAC reaction was implemented, which enabled the isolation of the desired product **1a** in a good yield of 65%.

**Scheme 4:**
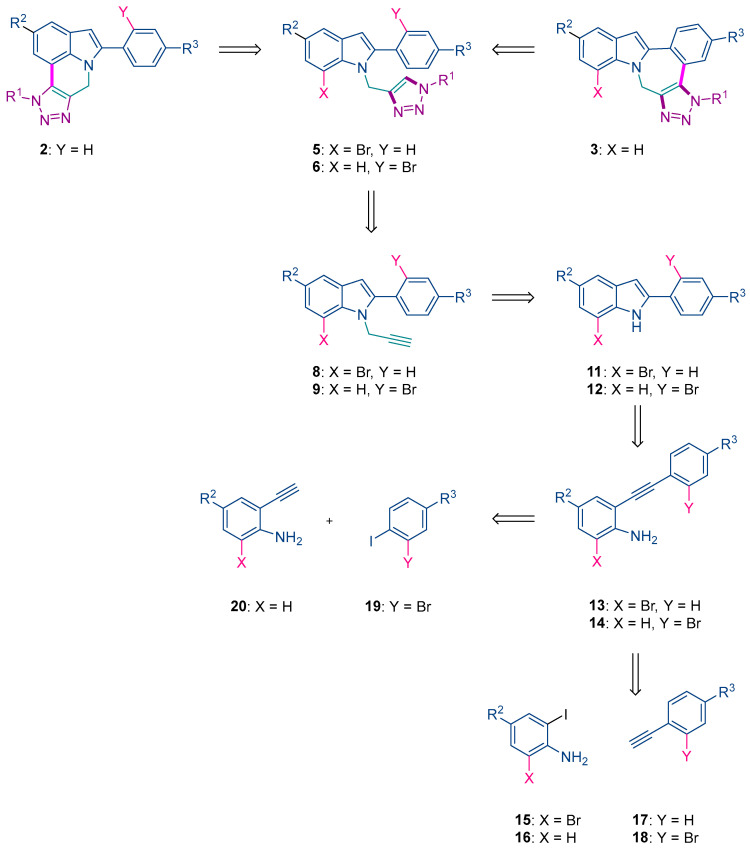
Retrosynthetic approach for assembly of 7,10-dihydropyrrolo[3,2,1-*ij*][1,2,3]triazolo[4,5-*c*]quinolines **2** and 5,8-dihydrobenzo[3,4][1,2,3]triazolo[4′,5′:5,6]azepino[1,2-*a*]indoles **3** derivatives.

With these encouraging results in hand, we envisioned increasing molecular complexity by introducing different substituents on 7,10-dihydropyrrolo[3,2,1-*ij*][1,2,3]triazolo[4,5-*c*]quinoline nucleus. Our approach to assembling the required substrates **5** involves a one-pot, chemoselective Sonogashira cross-coupling of substituted 2-bromo-6-iodoanilines **15** with aryl acetylenes **17**, followed by an endo-dig cyclization of the resulting 2-alkynylanilines **13**. The ensuing indole derivatives **11** are then subjected to *N*-propargylation to afford **8**, which subsequently undergo a Cu-catalyzed azide–alkyne cycloaddition (CuAAC) to yield the desired starting materials (**5a**–**k**) (Scheme 4).

Further details on the preparation of the starting materials, based on slightly modified known procedures, are provided in Supplementary Materials [39].

As shown in Table 3, good to excellent results were usually obtained with a variety of 7-bromo-1-((1-substituted-1*H*-1,2,3-triazol-4-yl)methyl)-2-phenyl-1*H*-indoles **5** containing different functionalities such as ether, ester, benzyl, and halogens both in indole and triazole cores (Table 3, **2a**–**k**, 52–99%).

The potential of the reported strategy for the synthesis of polyfused *N*-heterocycles containing triazole was further demonstrated by the ready construction of the 5,8-dihydrobenzo[3,4][1,2,3]triazolo[4′,5′:5,6]azepino[1,2-*a*]indole skeleton, albeit through a slight modification in the starting 1-((1*H*-1,2,3-triazol-4-yl)methyl)-1*H*-indole. In particular, we envisaged that 2-((2-bromoaryl)ethynyl)-4-substituted aniline **14** might represent suitable building blocks for the synthesis of this core through a process that involves the assembly of the indole ring followed by *N*-propargylation/CuAAC/palladium-catalyzed intramolecular C–H bond activation. (Scheme 3)

**Scheme 5:**
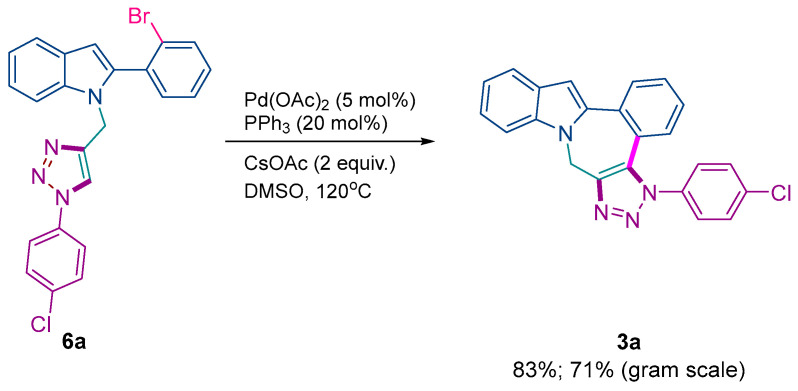
Synthesis of 5-(4-chlorophenyl)-5,8-dihydrobenzo[3,4][1,2,3]triazolo[4′,5′:5,6]azepino[1,2-*a*]indole **3a** from 2-(2-bromophenyl)-1-((1-(4-chlorophenyl)-1*H*-1,2,3-triazol-4-yl)methyl)-1*H*-indole **6a** on a 0.30 mmol scale and a gram scale.

We selected 2-(2-bromophenyl)-1-((1-(4-chlorophenyl)-1*H*-1,2,3-triazol-4-yl)methyl)-1*H*-indole **6a** as the model substrate for obtaining the corresponding 5-(4-chlorophenyl)-5,8-dihydrobenzo[3,4][1,2,3]triazolo[4′,5′:5,6]azepino[1,2-*a*]indole **3a**. Fortunately, under the optimized reaction condition [Pd(OAc)_2_ (2 mol %), PPh_3_ (4 mol %), and CsOAc (2.0 equiv.) in DMSO at 120 °C], we successfully isolated **3a** in 83% yield after one hour (Scheme 5) and on the gram scale in 71% yield.

To demonstrate the versatility of intramolecular C–H annulation, we prepared several 2-(2-bromoaryl)-1-((1-substituted-1*H*-1,2,3-triazol-4-yl)methyl)-1*H*-indole **6**. All the tested starting materials (**6b**–**n**) were converted into the corresponding functionalized 5,8-dihydrobenzo[3,4][1,2,3]triazolo[4′,5′:5,6]azepino[1,2-*a*]indole derivative **3** in good to excellent yield (Table 4, **3b**–**q**, 55–96%), regardless of the electronic effect of the substituents.

**Scheme 6:**
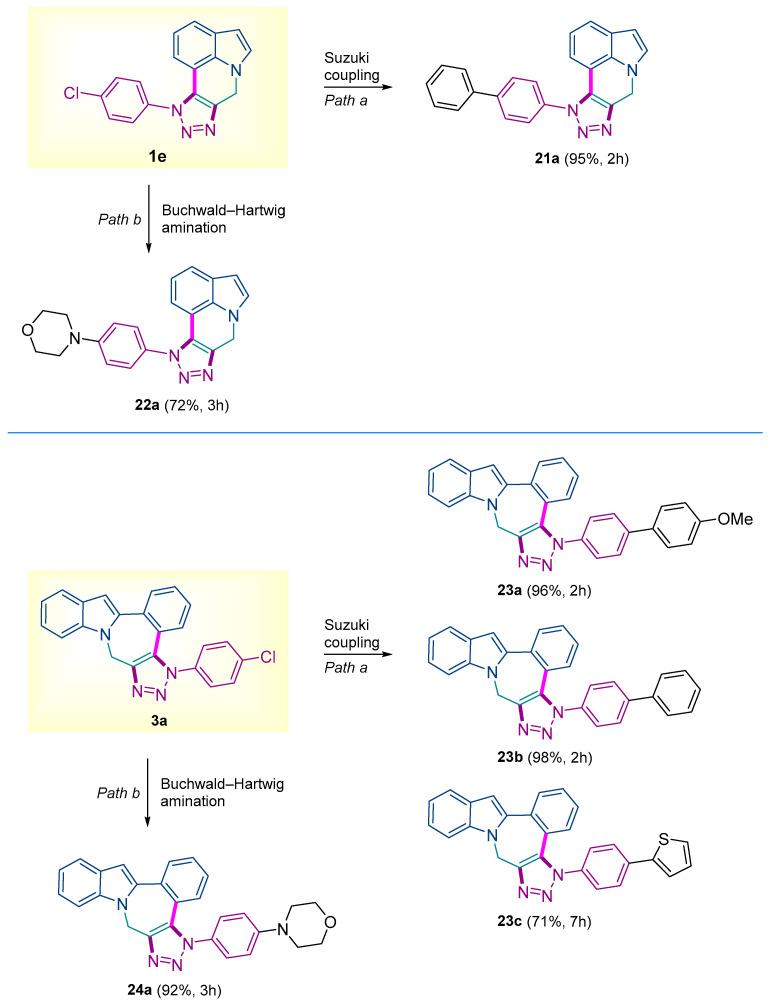
Post-synthetic modification of **1e** and **3a**. Path (a): reactions were carried out on a 0.25 mmol scale using 1.0 equiv. of **1e** or **3a**, 1.5 equiv. of boronic acid derivatives, 0.02 equiv. of Pd_2_(dba)_3_, 0.04 equiv. of Sphos, and 3.0 equiv. of K_3_PO_4_ in 2.0 mL of dry 1,4-dioxane under argon at 100 °C. Path (b): reaction was carried out on a 0.25 mmol scale using 1.0 equiv. of **1e** or **3a**, 1.5 equiv. of morpholine, 0.02 equiv. of Pd_2_(dba)_3_, 0.04 equiv of Xphos, and 2.0 equiv. of ^t^BuOK in 2.0 mL of dry toluene under argon at 100 °C.

It is worth noting that the developed protocol tolerates a–Cl substituent in the indole (Table 3, **2a**–**d**; Table 4, **3o**, **3p**) and/or triazole framework (Scheme 5, **3a**; Table 2, **1e**; Table 4, **3f**, **3g**, **3k**, **3o**) of hybrid heterocycles **1**, **2**, and **3**, which may serve as a useful handle for introducing other functional groups through cross-coupling reactions. For example, we performed post-synthetic modification of **1e** and **3a** via palladium-catalyzed Suzuki–Miyaura coupling and Buchwald–Hartwig C-N bond-forming reactions. As shown in Scheme 6, compounds **1e** and **3a** were successfully treated with different aryl and heteroaryl boronic acids under reaction conditions previously reported by us [Pd_2_dba_3_, Sphos, K_3_PO_4_, 1,4-dioxane, 100 °C] (Scheme 6, *path a*; **21a**, **23a**–**c**, 71–98%) [40].

Furthermore, the Buchwald–Hartwig amination reactions were carried out in the presence of Pd_2_(dba)_3_, Xphos, and ^t^BuOK in toluene, leading to the desired *N*-derivatives **22a** and **24a** in a yield of 72% and 92%, respectively (Scheme 6, *path b*). 

**Scheme 7:**
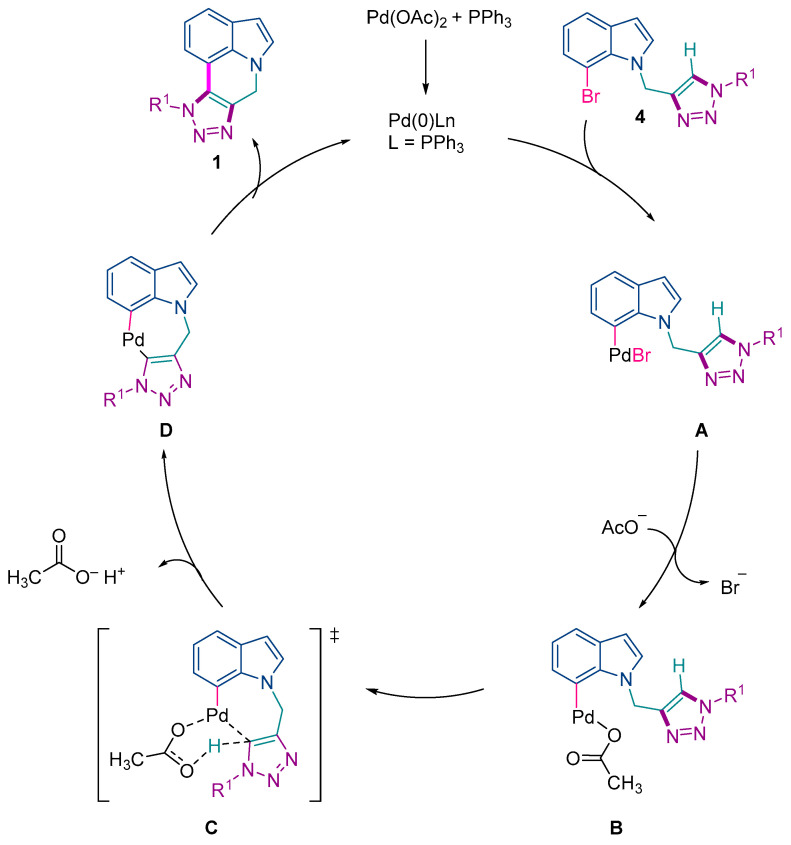
Proposed reaction mechanism.

Based on the literature reports highlighting the role of the acetate ion in promoting the cyclization process [41], the mechanism of palladium-catalyzed C–H functionalization/C-C bond formation can be rationalized, as depicted in Scheme 7, for the formation of the final product **1** (ligands omitted for clarity). Following the in situ reduction of Pd(II) to Pd(0), oxidative addition of substrate **4** to Pd(0) generates the aryl palladium complex **A**. In the presence of the acetate ion, a rapid ligand exchange occurs, affording the palladium(II)-acetate complex **B**. This intermediate undergoes cyclopalladation via transition state **C**, forming the seven-member palladacycle **D**. The transition state likely involves a concerted metalation–deprotonation (CMD) process, where the Pd–C bond formation and C–H bond cleavage occur simultaneously, facilitated by the acetate ligand acting as base. Finally, reductive elimination regenerates the active Pd(0) species and delivers the triazole-fused heterocycle **1**.

However, the involvement of an electrophilic mechanism reported for direct Pd-catalyzed arylation of 1,2,3-triazoles cannot be definitively ruled out [42]. 

## 3. Materials and Methods

### 3.1. General Information

All of the commercially available reagents, catalysts, bases, and solvents were used as purchased, without further purification. Starting materials and reaction products were purified by flash chromatography using SiO_2_ as the stationary phase, eluting with *n*-hexane/ethyl acetate (EtOAc) mixtures. ^1^H NMR (400.13 MHz), ^13^C NMR (100.6 MHz), and ^19^F spectra (376.5 MHz) were recorded with a Bruker Avance 400 spectrometer equipped with a Nanobay console and Cryoprobe Prodigy probe (Bruker, Billerica, MA, USA). Splitting patterns are designed as s (singlet), d (doublet), t (triplet), q (quartet), m (multiplet), or bs (broad singlet). IR spectra were recorded with a Jasco FT/IR-430 spectrometer (JASCO Corporation, Tokyo, Japan). HRMS were recorded with an Orbitrap Exactive Mass spectrometer with ESI source (Thermo Fisher, Waltham, MA, USA). Melting points were determined with a Büchi B-545 apparatus and are uncorrected.

### 3.2. General Experimental Procedures

#### 3.2.1. Synthetic Procedures for Starting Materials

##### General Procedure for the Preparation of 1-((1-Substituted)-1H-1,2,3-triazol-4-yl)methyl)-7-bromo-1H-indole **4**

Typical procedure for the preparation of 1-((1-benzyl)-1H-1,2,3-triazol-4-yl)methyl)-7-bromo-1*H*-indole **4a**

STEP 1: Synthesis of 7-bromo-1-(prop-2-yn-1-yl)-1*H*-indole **7a**

A flame-dried 100 mL three-necked round-bottom flask, equipped with a magnetic stirring bar, was charged under argon with NaH (60% dispersion in mineral oil, 240.0 mg, 6.00 mmol, 1.2 equiv.). The solid was washed three times with *n*-hexane and then suspended in anhydrous DMF (15.0 mL). The suspension was cooled to 0 °C, and a solution of 7-bromo-1*H*-indole **10** (980.0 mg, 5.00 mmol, 1.0 equiv.) in DMF (15.0 mL) was added dropwise. The reaction mixture was allowed to warm to room temperature and stirred for 5 min. After cooling again to 0 °C, propargyl bromide (solution 80 wt % in toluene) (808 μL, 7.50 mmol, 1.5 equiv.) was added dropwise. The mixture was subsequently warmed to room temperature and stirred for 30 min. Upon completion, the reaction was quenched with water, diluted with diethyl ether, and washed with brine. The organic layer was dried over Na_2_SO_4_, filtered, and concentrated under reduced pressure. The residue was purified by flash chromatography on SiO_2_ (25–40 μm), followed by eluting with a 90/10 (*v*/*v*) *n*-hexane-AcOEt mixture (R*_f_* = 0.25) to obtain 7-bromo-1-(prop-2-yn-1-yl)-1*H*-indole **7a** (1.111 g, 95% yield).

7-bromo-1-(prop-2-yn-1-yl)-1*H*-indole **7a**: 95% yield; white solid; mp: 65–67 °C; IR (neat): 3267, 1303, 917, 779, 710 cm^−1^; ^1^H NMR (400.13 MHz) (CDCl_3_): δ 7.46 (dd, *J* = 7.8, 1.0 Hz, 1H), 7.28 (dd, *J* = 7.6, 1.0 Hz, 1H), 7.15 (s, 1H), 6.86 (t, *J* = 7.7 Hz, 1H), 6.45 (d, *J* = 3.3 Hz, 1H), 5.29 (d, *J* = 2.5 Hz, 2H), 2.35 (d, *J* = 2.5 Hz, 1H); ^13^C NMR (100.6 MHz) (CDCl_3_): δ 132.4 (C), 132.2 (C), 130.1 (CH), 127.2 (CH), 121.2 (CH), 120.6 (CH), 103.6 (C), 102.8 (CH), 78.8 (C), 74.2 (CH), 38.1 (CH_2_). HRMS: *m*/*z* [M + H]^+^ calcd for C_11_H_9_BrN: 233.9913; found: 233.9926.

STEP 2: synthesis of 1-((1-benzyl)-1H-1,2,3-triazol-4-yl)methyl)-7-bromo-1H-indole **4a**

In a 50 mL Radley’s Discovery Technology Carousel reactor equipped with a magnetic stirrer (Radley, London, UK), 7-bromo-1-(prop-2-yn-1-yl)-1*H*-indole **7a** (1.0 g, 4.27 mmol, 1.0 equiv.) was dissolved in 4 mL of 1,4-dioxane. Then, CuI (162.3 mg, 0.85 mmol, 0.20 equiv.) and benzyl azide (624.7 mg, 4.70 mmol, 1.1 equiv.) were sequentially added, the vial was sealed, and the reaction mixture was stirred at 80 °C overnight. Upon completion, the mixture was concentrated under reduced pressure, and the crude product was purified by flash chromatography on SiO_2_ (25–40 μm), eluting with an 80/20 (*v*/*v*) *n*-hexane-AcOEt mixture (R*_f_* = 0.25) to afford 1-((1-benzyl)-1*H*-1,2,3-triazol-4-yl)methyl)-7-bromo-1*H*-indole **4a** (1.489 g, 95% yield).

1-((1-benzyl-1*H*-1,2,3-triazol-4-yl)methyl)-7-bromo-1*H*-indole **4a**: 95% yield; off-white solid; mp: 85–87 °C; IR (neat): 3067, 1554, 1313, 912, 709 cm^−1^; ^1^H NMR (400.13 MHz) (CDCl_3_): δ 7.46–7.36 (m, 1H), 7.25–7.15 (m, 4H), 7.15–7.09 (m, 2H), 7.04 (dd, *J*_1_ = 6.7, *J*_2_ = 2.9 Hz, 2H), 6.80 (t, *J* = 7.7 Hz, 1H), 6.39 (d, *J* = 3.3 Hz, 1H), 5.75 (s, 2H), 5.26 (s, 2H); ^13^C NMR (100.6 MHz) (CDCl_3_): δ 146.1 (C), 134.6 (C), 132.1 (C), 132.1 (C), 131.1 (CH), 129.1 (CH), 128.7 (CH), 127.8 (CH), 127.2 (CH), 122.1 (CH), 120.9 (CH), 120.7 (CH), 103.5 (C), 102.9 (CH), 54.1 (CH_2_), 43.6 (CH_2_); HRMS: *m*/*z* [M + H]^+^ calcd for C_18_H_16_BrN_4_: 367.0553; found: 367.0561.

##### One-Pot Protocol for the Preparation of 1-((1-Substituted)-1H-1,2,3-triazol-4-yl)methyl)-7-bromo-1*H*-indole **4** Starting from **10**

Starting materials **4a**–**e** were prepared through a one-pot, two-steps protocol omitting the isolation of **7a**–**e.**

Starting materials **5a**–**5k** and **6a**–**6q** were prepared according to slightly modified literature procedures described in the Supplementary Materials [43] through the retrosynthesis depicted in Scheme 3.

#### 3.2.2. Synthetic Procedures for Final Products

##### Typical Procedure for the Preparation of Functionalized 10-Substituted 7,10-dihydropyrrolo[3,2,1-ij][1,2,3]triazolo[4,5-c]quinoline **1** and **2**: Synthesis of 10-benzyl-7,10-dihydropyrrolo[3,2,1-ij][1,2,3]triazolo[4,5-c]quinoline **1a**

In a 50 mL Carousel Tube Reactor (Radley, London, UK), equipped with a magnetic stirrer, Pd(OAc)_2_ (3.4 mg, 0.015 mmol, 0.05 equiv.) and PPh_3_ (7.9 mg, 0.03 mmol, 0.10 equiv.) were dissolved in anhydrous DMSO (2 mL) under an argon atmosphere at room temperature. To this solution, 1-((1-benzyl-1*H*-1,2,3-triazol-4-yl)methyl)-7-bromo-1*H*-indole **4a** (110.2 mg, 0.30 mmol, 1.0 equiv.) and Cs(OAc) (114.6 mg, 0.60 mmol, 2.0 equiv.) were added. The reaction mixture was stirred at 120 °C and monitored by TLC (*n*-hexane/EtOAc, 80/20) for the conversion of **4a** into 10-benzyl-7,10-dihydropyrrolo[3,2,1-*ij*][1,2,3]triazolo[4,5-*c*]quinoline **1a**. Upon completion, the mixture was diluted with diethyl ether, washed with saturated NaCl solution, dried over Na_2_SO_4_, filtered, and concentrated under reduced pressure. The crude product was purified by flash chromatography on SiO_2_ (25–40 μm), eluting with an 80/20 (*v*/*v*) *n*-hexane–AcOEt mixture (R*_f_* = 0.20) to afford **1a** (68.6 mg, 80% yield).

##### Gram-Scale Synthesis of 10-Benzyl-7,10-dihydropyrrolo[3,2,1-ij][1,2,3]triazolo[4,5-c]quinoline **1a**

In a 50 mL Carousel Tube Reactor (Radley, London, UK), equipped with a magnetic stirrer, Pd(OAc)_2_ (10.2 mg, 0.045 mmol, 0.05 equiv.) and PPh_3_ (23.7 mg, 0.09 mmol, 0.10 equiv.) were dissolved in anhydrous DMSO (6.0 mL) under an argon atmosphere at room temperature. To this solution, 1-((1-benzyl-1*H*-1,2,3-triazol-4-yl)methyl)-7-bromo-1*H*-indole **4a** (1.10 g, 3.00 mmol, 1.0 equiv.) and CsOAc (343.8 mg, 1.8 mmol, 2.0 equiv.) were added. The reaction mixture was stirred at 120 °C and monitored by TLC (*n*-hexane/EtOAc, 80/20) for the conversion of **4a** into 10-benzyl-7,10-dihydropyrrolo[3,2,1-*ij*][1,2,3]triazolo[4,5-*c*]quinoline **1a**. Upon completion, the mixture was diluted with diethyl ether, washed with saturated NaCl solution, dried over Na_2_SO_4_, filtered, and concentrated under reduced pressure. The crude product was purified by flash chromatography on SiO_2_ (25–40 μm), eluting with an 80/20 (*v*/*v*) *n*-hexane–AcOEt mixture (R*_f_* = 0.20) to afford **1a** (592.7 mg, 69% yield).

10-benzyl-7,10-dihydropyrrolo[3,2,1*-ij*][1,2,3]triazolo[4,5-*c*]quinoline **1a**: white solid; mp: 246–248 °C; IR (neat): 3156, 1309, 1230, 684, 599 cm^−1^; ^1^H NMR (400.13 MHz) (CDCl_3_): δ 7.37 (dd, *J*_1_ = 8.0 Hz, *J*_2_ = 1.0 Hz, 1H), 7.29–7.09 (m, 5H), 7.03 (d, *J* = 3.1 Hz, 1H), 6.91 (d, *J* = 7.5 Hz, 1H), 6.83 (t, *J* = 7.5 Hz, 1H), 6.43 (d, *J* = 3.2 Hz, 1H), 5.73 (s, 2H), 5.57 (s, 2H); ^13^C NMR (100.6 MHz) (CDCl_3_): δ 139.9 (C), 134.6 (C), 133.4 (C), 129.1 (CH), 128.3 (CH), 126.84 (CH), 126.82 (C), 126.6 (CH), 125.9 (C), 122.4 (CH), 120.1 (CH), 114.5 (CH), 109.1 (C), 103.6 (CH), 53.3 (CH_2_), 44.6 (CH_2_); HRMS: *m*/*z* [M + H]^+^ calcd for C_18_H_15_N_4_: 287.1291; found: 287.1287.

##### Typical Procedure for the Preparation of Functionalized 5-Substituted-5,8-dihydrobenzo[3,4][1,2,3]triazolo[4′,5′:5,6]azepino[1,2-a]indole **3**: 5-(4-chlorophenyl)-5,8-dihydrobenzo[3,4][1,2,3]triazolo[4′,5′:5,6]azepino[1,2-a]indole **3a**

In a 50 mL Carousel Tube Reactor (Radley, London, UK), equipped with a magnetic stirrer, Pd(OAc)_2_ (3.4 mg, 0.015 mmol, 0.05 equiv.) and PPh_3_ (7.9 mg, 0.03 mmol, 0.10 equiv.) were dissolved in anhydrous DMSO (2 mL) under argon. Then, 2-(2-bromophenyl)-1-((1-(4-chlorophenyl)-1*H*-1,2,3-triazol-4-yl)methyl)-1*H*-indole **6a** (139.1 mg, 0.3 mmol, 1 equiv.) and CsOAc (114.6 mg, 0.6 mmol, 2.0 equiv.) were added, and the reaction mixture was stirred at 120 °C and monitored by TLC. Upon completion, the mixture was diluted with diethyl ether, washed with saturated NaCl solution, dried over Na_2_SO_4_, filtered, and concentrated under reduced pressure. The residue was purified by flash chromatography on SiO_2_ (25–40 μm), eluting with an 70/30 (*v*/*v*) *n*-hexane-AcOEt mixture (R*_f_* = 0.19) to afford **3a** (95.3 mg, 83% yield).

5-(4-chlorophenyl)-5,8-dihydrobenzo[3,4][1,2,3]triazolo[4′,5′:5,6]azepino[1,2-*a*]indole **3a**: 83% yield; yellow solid; IR (neat): 1600, 1459, 1053, 786, 765 cm^−1^; mp: 232–234 °C; ^1^H NMR (400.13 MHz) (CDCl_3_): δ 7.90 (d, *J* = 8.1 Hz, 1H), 7.66 (d, *J* = 7.8 Hz, 1H), 7.58 (d, *J* = 8.3 Hz, 1H), 7.50–7.44 (m, 3H), 7.39–7.37 (m, 2H), 7.31 (td, *J*_1_ = 8.3 Hz, *J*_2_ = 1.2 Hz, 1H), 7.25 (td, *J*_1_ = 8.6 Hz, *J*_2_= 1.2 Hz, 1H), 7.15 (t, *J* = 7.5 Hz, 1H), 6.97 (d, *J* = 7.9 Hz, 1H), 6.86 (s, 1H), 5.44 (s, 2H); ^13^C NMR (100.6 MHz) (CDCl_3_): δ 145.0 (C),138.4 (C), 137.0 (C), 135.7 (C), 135.2 (C), 133.8 (C), 132.5 (C), 131.9 (CH), 129.9 (CH), 129.8 (CH), 128.6 (CH), 127.9 (C), 127.8 (CH), 126.3 (CH), 122.7 (CH), 122.6 (C), 121.0 (CH), 120.2 (CH), 109.3 (CH), 104.0 (CH), 39.4 (CH_2_). HRMS: *m*/*z* [M + H]^+^ calcd for C_23_H_16_ClN_4_: 383.1058; found: 383.1066.

#### 3.2.3. Synthetic Procedures for Post-Synthetic Derivatives Compounds **21a**, **23a**–**c**, **22a**, and **24a**

##### Typical Procedure for the Suzuki Cross-Coupling: Synthesis of 10-([1,1′-Biphenyl]-4-yl)-7,10-dihydropyrrolo[3,2,1-ij][1,2,3]triazolo[4,5-c]quinolone **21a**

A Carousel Tube Reactor (Radley, London, UK), equipped with a magnetic stirrer, was charged with Pd_2_(dba)_3_ (4.6 mg, 0.005 mmol, 0.02 equiv.), Sphos (4.1 mg, 0.010 mmol, 0.04 equiv.), and dry dioxane (2.0 mL). After solubilization of this precatalyst system at room temperature under an argon atmosphere, compound **1e** (76.7 mg, 0.25 mmol, 1.0 equiv.), phenyl boronic acid (45.7 mg, 0.375 mmol, 1.5 equiv.), and K_3_PO_4_ (0.159 g, 0.750 mmol, 3.0 equiv.) were added. The resulting mixture was heated at 100 °C and stirred for 2 h. Afterwards, the mixture was cooled, added with ethyl acetate, and washed with brine. The organic phase was separated, dried over Na_2_SO_4_, filtered, and concentrated under reduced pressure. The residue was purified by flash chromatography on SiO_2_ (25–40 μm), (90:10 *n*-hexane/EtOAc) to obtain **21a** (82.6 mg, 95% yield).

10-([1,1′-biphenyl]-4-yl)-7,10-dihydropyrrolo[3,2,1-*ij*][1,2,3]triazolo[4,5-*c*]quinoline **21a**: 95% yield; red solid; mp: 220–222 °C; IR (neat): 3152, 1333, 1248, 1068, 708 cm^−1^; ^1^H NMR (400.13 MHz) (CDCl_3_): δ 7.77 (d, *J* = 8.30 Hz, 2H), 7.65–7.60 (m, 5H), 7.46–7.41 (m, 3H), 7.14 (d, *J* = 3.00 Hz, 1H), 6.80–6.70 (m, 2H), 6.5 (d, *J* = 3.00 Hz, 1H), 5.73 (s, 2H); ^13^C NMR (100.6 MHz) (CDCl_3_): δ 143.4 (C), 139.6 (C), 136.0 (C), 133.7 (C), 129.9 (C), 129.1 (CH), 128.4 (C), 128.3 (CH), 128.2 (CH), 127.3 (C), 126.9 (CH), 126.3 (CH), 126.0 (C), 122.7 (CH), 120.0 (CH), 114.4 (CH), 109.2 (C), 103.8 (CH), 44.6 (CH_2_). HRMS: *m*/*z* [M + H]^+^ calcd for C_23_H_17_N_4_: 349.1448; found: 349.1432. 

##### Typical Procedure for the Buchwald–Hartwig N-Arylation: Synthesis of 4-(4-(Benzo[3,4][1,2,3]triazolo[4′,5′:5,6]azepino[1,2-a]indol-5(8H)-yl)phenyl)morpholine **24a**

In a 50 mL Carousel Tube Reactor (Radley, London, UK) containing a magnetic stirring bar, Pd_2_(dba)_3_ (4.6 mg, 0.0053 mmol, 0.02 equiv.) and Xphos ligand (4.8 mg, 0.01 mmol, 0.04 equiv.) were dissolved at room temperature with 2.0 mL of toluene under argon. Then, 5-(4-chlorophenyl)-5,8-dihydrobenzo[3,4][1,2,3]triazolo[4′,5′:5,6]azepino[1,2-*a*]indole **3a** (95.7 mg, 0.25 mmol, 1.0 equiv.), morpholine (33 μL, 0.375 mmol, 1.5 equiv.), and *^t^*BuOK (56.1 mg, 0.50 mmol, 2.0 equiv.) were added. The reaction mixture was stirred for 3 h at 100 °C. Afterwards, the reaction mixture was cooled to room temperature, diluted with EtOAc, and washed with a saturated NaCl solution. The organic layer was separated, dried over Na_2_SO_4_, and concentrated under reduced pressure. The residue was purified by flash chromatography (SiO_2_ 25–40 μm, *n*-hexane/EtOAc 70/30) to afford 4-(4-(benzo[3,4][1,2,3]triazolo[4′,5′:5,6]azepino[1,2-*a*]indol-5(8*H*)-yl)phenyl)morpholine **24a** (92% yield, 43.2 mg).

4-(4-(benzo[3,4][1,2,3]triazolo[4′,5′:5,6]azepino[1,2-*a*]indol-5(8*H*)-yl)phenyl)morpholine **24a**: 92% yield; brown solid; mp: 238–240 °C; IR (neat): 3115, 2980, 1365, 911, 709 cm^−1^; ^1^H NMR (400.13 MHz) (CDCl_3_): δ 7.80 (d, *J* = 7.9 Hz, 1H), 7.58 (d, *J* = 7.9 Hz, 1H), 7.51 (d, *J* = 8.4 Hz, 1H), 7.35 (d, *J* = 7.6 Hz, 1H), 7.23–7.09 (m, 4H), 7.05 (t, *J* = 7.5 Hz, 1H), 6.96 (d, *J* = 7.9 Hz, 1H), 6.85 (d, *J* = 8.6 Hz, 2H), 6.75 (s, 1H), 5.35 (s, 2H), 3.79 (t, *J* = 4.8 Hz, 4H), 3.14 (t, *J* = 4.8 Hz, 4H).; ^13^C NMR (100.6 MHz) (CDCl_3_): δ 151.7 (C), 144.5 (C), 138.7 (C), 136.9 (C), 133.6 (C), 132.3 (C), 131.6 (CH), 129.3 (CH), 128.5 (CH), 127.8 (C), 127.6 (CH), 126.0 (CH), 123.19 (C), 123.18 (C), 122.5 (CH), 120.9 (CH), 120.1 (CH), 115.5 (CH), 109.3 (CH), 103.8 (CH), 66.7 (CH_2_), 48.6 (CH_2_), 39.5 (CH_2_). HRMS: *m*/*z* [M + H]^+^ calcd for C_27_H_24_N_5_O: 434.1974; found: 434.1988.

### 3.3. Characterization Data of Synthesized Compounds

Characterization data for the starting materials **4b**–**e**, **5a**–**k**, and **6a**–**q**, with their precursors reported in the Supplementary Materials.

#### 3.3.1. Characterization Data of Final Compounds **1b**–**g** and **2a**–**k**

10-(4-methoxyphenyl)-7,10-dihydropyrrolo[3,2,1-*ij*][1,2,3]triazolo[4,5-*c*]quinoline **1b**: 81% yield; brown solid; mp: 238–240 °C; IR (neat): 3115, 2980, 1365, 911, 709 cm^−1^; ^1^H NMR (400.13 MHz) (CDCl_3_): δ 7.45 (d, *J* = 8.8 Hz, 2H), 7.40 (d, *J* = 8.3 Hz, 1H), 7.11 (d, *J* = 3.1 Hz, 1H), 7.03 (d, *J* = 8.8 Hz, 2H), 6.76 (t, *J* = 7.7 Hz, 1H), 6.59 (d, *J* = 7.3 Hz, 1H), 6.48 (d, *J* = 3.1 Hz, 1H), 5.69 (s, 2H), 3.85 (s, 3H); ^13^C NMR (100.6 MHz) (CDCl_3_): δ 161.0 (C), 139.1 (C), 133.6 (C), 129.9 (C), 129.8 (C), 127.4 (CH), 126.8 (CH), 125.9 (C), 122.5 (CH), 119.9 (CH), 114.8 (CH), 114.2 (CH), 109.3 (C), 103.7 (CH), 55.7 (CH_3_), 44.6 (CH_2_). HRMS: *m*/*z* [M + H]^+^ calcd for C_18_H_15_N_4_O: 303.1240; found: 303.1233.

10-(3,4,5-trimethoxyphenyl)-7,10-dihydropyrrolo[3,2,1-*ij*][1,2,3]triazolo[4,5-*c*]quinoline **1c**: 91% yield; yellow solid; mp: 240–242 °C; IR (neat): 3109, 2991, 1344, 924, 715 cm^−1^; ^1^H NMR (400.13 MHz) (CDCl_3_): δ 7.40 (d, *J* = 7.9 Hz, 1H), 7.08 (d, *J* = 2.9 Hz, 1H), 6.84–6.72 (m, 4H), 6.46 (d, *J* = 2.9 Hz, 1H), 5.63 (s, 2H), 3.88 (s, 3H), 3.80 (s, 6H); ^13^C NMR (100.6 MHz) (CDCl_3_): δ 153.8 (C), 139.4 (C), 139.2 (C), 133.6 (C), 132.3 (C), 129.8 (C), 126.9 (CH), 126.0 (C), 122.7 (CH), 120.0 (CH), 114.4 (CH), 109.1 (C), 103.7 (CH), 103.6 (CH), 61.2 (CH_3_), 56.5 (CH_3_), 44.5 (CH_2_). HRMS: *m*/*z* [M + H]^+^ calcd for C_20_H_19_N_4_O_3_: 363.1452; found: 363.1466.

10-(2-fluoro-4-methylphenyl)-7,10-dihydropyrrolo[3,2,1-*ij*][1,2,3]triazolo[4,5-c]quinoline **1d**: 70% yield; brown solid; mp: 270–272 °C; IR (neat): 2877, 1537, 1194, 820, 722 cm^−1^; ^1^H NMR (400.13 MHz) (CDCl_3_): δ 7.46–7.36 (m, 2H), 7.21–7.10 (m, 3H), 6.77 (t, *J* = 7.7 Hz, 1H), 6.52–6.45 (m, 2H), 5.73 (s, 2H), 2.46 (s, 3H); ^13^C NMR (100.6 MHz) (CDCl_3_): δ 156.6 (d, *J_C-F_* = 253.0 Hz, C), 143.8 (d, *J_C-F_* = 7.7 Hz, C), 138.9 (C), 133.6 (C), 131.0 (C), 128.3 (CH), 126.8 (CH), 125.9 (C), 125.79 (d, *J_C-F_* = 3.8 Hz, CH), 122.7 (CH), 122.4 (d, *J_C-F_* = 12.4 Hz, C), 120.1 (CH), 117.6 (d, *J_C-F_* = 18.6 Hz, CH), 113.7 (CH), 109.0 (C), 103.7 (CH), 44.6 (CH_2_), 21.6 (CH_3_); ^19^F NMR (376.5 MHz) (CDCl_3_): δ −121.29 (m). HRMS: *m*/*z* [M + H]^+^ calcd for C_18_H_14_FN_4_: 305.1197; found: 305.1181.

10-(4-chlorophenyl)-7,10-dihydropyrrolo[3,2,1-*ij*][1,2,3]triazolo[4,5-*c*]quinoline **1e**: 85% yield; yellow solid; mp: 230–232 °C; IR (neat): 3059, 1508, 1198, 1087, 717 cm^−1^; ^1^H NMR (400.13 MHz) (CDCl_3_): δ 7.59–7.47 (m, 4H), 7.41 (d, *J* = 8.1 Hz, 1H), 7.11 (d, *J* = 3.2 Hz, 1H), 6.77 (t, *J* = 7.7 Hz, 1H), 6.61 (d, *J* = 7.3 Hz, 1H), 6.49 (d, *J* = 3.2 Hz, 1H), 5.68 (s, 2H); ^13^C NMR (100.6 MHz) (CDCl_3_): δ 139.4 (C), 136.5 (C), 135.5 (C), 133.6 (C), 130.0 (C), 129.8 (CH), 127.3 (CH), 126.9 (CH), 126.1 (C), 122.9 (CH), 120.0 (CH), 114.2 (CH), 108.9 (C), 103.8 (CH), 44.5 (CH_2_). HRMS: *m*/*z* [M + H]^+^ calcd for C_17_H_12_ClN_4_: 307.0745; found: 307.0730.

10-(4-nitrophenyl)-7,10-dihydropyrrolo[3,2,1-*ij*][1,2,3]triazolo[4,5-*c*]quinoline **1f**: 89% yield; yellow solid; mp: 246–248 °C; IR (neat): 3102, 1486, 1092, 815 cm^−1^; ^1^H NMR (400.13 MHz) (CDCl_3_): δ 8.51 (d, *J* = 8.8 Hz, 2H), 7.90 (d, *J* = 8.8 Hz, 2H), 7.53 (d, *J* = 8.0 Hz, 1H), 7.22 (d, *J* = 3.0 Hz, 1H), 6.87 (t, *J* = 7.7 Hz, 1H), 6.75 (d, *J* = 7.1 Hz, 1H), 6.59 (d, *J* = 2.6 Hz, 1H), 5.80 (s, 2H); ^13^C NMR (100.6 MHz) (CDCl_3_): δ 148.8 (C), 142.1 (C), 140.3 (C), 133.9 (C), 130.2 (C), 127.3 (CH), 127.0 (CH), 126.6 (C), 125.4 (CH), 123.6 (CH), 120.3 (CH), 114.6 (CH), 108.7 (C), 104.3 (CH), 44.8 (CH_2_). HRMS: *m*/*z* [M + H]^+^ calcd for C_17_H_12_N_5_O_2_: 318.0985; found: 318.0973.

4-(pyrrolo[3,2,1-*ij*][1,2,3]triazolo[4,5-*c*]quinolin-10(7*H*)-yl)benzonitrile **1g**: 83% yield; yellow solid; mp: 232–234 °C; IR (neat): 3048, 1517, 1185, 1076, 678 cm^−1^; ^1^H NMR (400.13 MHz) (CDCl_3_): δ 7.96 (d, *J* = 8.2 Hz, 2H), 7.84 (d, *J* = 8.2 Hz, 2H), 7.54 (d, *J* = 7.8 Hz, 1H), 7.22 (d, *J* = 3.2 Hz, 1H), 6.90 (t, *J* = 7.7 Hz, 1H), 6.75 (d, *J* = 7.3 Hz, 1H), 6.61 (d, *J* = 3.2 Hz, 1H), 5.79 (s, 2H); ^13^C NMR (100.6 MHz) (CDCl_3_): δ 140.5 (C), 140.0 (C), 133.8 (CH), 133.7 (C), 129.9 (C), 127.1 (CH), 126.7 (CH), 126.3 (C), 123.4 (CH), 120.0 (CH), 117.6 (C), 114.4 (C), 114.3 (CH) 108.6 (C), 104.0 (CH), 44.6 (CH_2_). HRMS: *m*/*z* [M + H]^+^ calcd for C_18_H_12_N_5_: 298.1087; found: 298.1099.

10-benzyl-2-chloro-5-phenyl-7,10-dihydropyrrolo[3,2,1-*ij*][1,2,3]triazolo[4,5-*c*]quinoline **2a**: 80% yield; white solid; mp: 240–242 °C; IR (neat): 3014, 1489, 1347, 1015, 985 cm^−1^; ^1^H NMR (400.13 MHz) (CDCl_3_): δ 7.63–7.21 (m, 10H), 7.16 (s, 1H), 6.91 (s, 1H), 6.43 (s, 1H), 5.77 (s, 2H), 5.54 (s, 2H); ^13^C NMR (100.6 MHz) (CDCl_3_): δ 142.0 (C), 140.8 (C), 134.2 (C), 132.9 (C), 131.4 (C), 129.2 (CH), 129.0 (CH), 128.8 (CH), 128.6 (CH), 128.5 (CH), 128.0 (C), 127.1 (C), 126.8 (CH), 126.0 (C), 121.0 (CH), 114.7 (CH), 110.3 (C), 102.9 (CH), 53.5 (CH_2_), 44.2 (CH_2_). HRMS: *m*/*z* [M + H]^+^ calcd for C_24_H_18_ClN_4_: 397.1214; found: 397.1225.

2-chloro-5-phenyl-10-(3,4,5-trimethoxyphenyl)-7,10-dihydropyrrolo[3,2,1-*ij*][1,2,3]triazolo[4,5-*c*]quinoline **2b**: 74% yield; brown solid; mp: 250–252 °C; IR (neat): 2924, 1596, 1414, 1228, 1127 cm^−1^; ^1^H NMR (400.13 MHz) (CDCl_3_): δ 7.53–7.48 (m, 2H), 7.45 (t, *J* = 7.5 Hz, 2H), 7.42–7.39 (m, 2H), 6.83 (s, 1H), 6.80 (s, 2H), 6.49 (s, 1H), 5.62 (s, 2H), 3.91 (s, 3H), 3.85 (s, 6H); ^13^C NMR (100.6 MHz) (CDCl_3_): δ 153.9 (C), 142.1 (C), 139.6 (C), 133.3 (C), 131.9 (C), 131.4 (C), 129.4 (C), 129.0 (CH), 128.9 (CH), 128.6 (CH), 127.9 (C), 127.3 (C), 125.9 (C), 121.4 (CH), 114.7 (CH), 110.4 (C), 103.3 (CH), 103.1 (CH), 61.2 (CH_3_), 56.5 (2CH_3_), 44.1(CH_2_); HRMS: *m*/*z* [M + H]^+^ calcd for C_26_H_22_ClN_4_O_3_: 473.1375; found: 473.1383. 

2-chloro-10-(3,5-dimethylphenyl)-5-phenyl-7,10-dihydropyrrolo[3,2,1-*ij*][1,2,3]triazolo[4,5-*c*]quinoline **2c**: 87% yield; white solid; mp: 258–260 °C; IR (neat): 3179, 1380, 1259, 1170, 999 cm^−1^; ^1^H NMR (400.13 MHz) (CDCl_3_): δ 7.53–7.34 (m, 6H), 7.21–7.13 (m, 3H), 6.65 (d, *J* = 1.8 Hz, 1H), 6.46 (s, 1H), 5.60 (s, 2H), 2.38 (s, 6H); ^13^C NMR (100.6 MHz) (CDCl_3_): δ 142.0 (C), 140.0 (C), 139.8 (C), 136.4 (C), 133.2 (C), 132.2 (CH), 131.4 (C), 129.0 (CH), 128.8 (CH), 128.6 (CH), 128.4 (C), 127.2 (C), 125.8 (C), 123.4 (CH), 121.1 (CH), 114.6 (CH), 110.5 (C), 103.0 (CH), 44.1 (CH_2_), 21.3 (CH_3_); HRMS: *m*/*z* [M + H]^+^ calcd for C_25_H_20_ClN_4_: 411.1371; found: 411.1389.

1-(3-(2-chloro-5-phenylpyrrolo[3,2,1-*ij*][1,2,3]triazolo[4,5-*c*]quinolin-10(7*H*)-yl)phenyl)ethan-1-one **2d**: 52% yield; yellow solid; mp: 250–252 °C; IR (neat): 3185, 1734, 1495, 1107, 662 cm^−1^; ^1^H NMR (400.13 MHz) (CDCl_3_): δ 8.24–8.14 (m, 2H), 7.81–7.70 (m, 2H), 7.52–7.38 (m, 6H), 6.56 (d, *J* = 1.8 Hz, 1H), 6.48 (s, 1H), 5.62 (s, 2H), 2.61 (s, 3H); ^13^C NMR (100.6 MHz) (CDCl_3_): δ 196.2 (C), 142.2 (C), 140.4 (C), 138.6 (C), 137.0 (C), 133.2 (C), 131.3 (C), 130.3 (CH), 130.2 (CH), 129.9 (CH), 129.0 (CH), 128.9 (CH), 128.7 (C), 128.59 (C), 128.56 (CH), 127.4 (C), 125.8 (C), 125.7 (CH), 121.6 (CH), 114.3 (CH), 110.1 (C), 103.1 (CH), 44.1 (CH_2_), 26.8 (CH_3_); HRMS: *m*/*z* [M + H]^+^ calcd for C_25_H_18_ClN_4_O: 425.1164; found: 425.1152. 

10-(9*H*-fluoren-2-yl)-5-phenyl-2-(trifluoromethyl)-7,10-dihydropyrrolo[3,2,1-*ij*][1,2,3]triazolo[4,5-*c*]quinoline **2e**: 76% yield; brown solid; mp: 280–282 °C; IR (neat): 2921, 1487, 1294, 1109, 735 cm^−1^; ^1^H NMR (400.13 MHz) (CDCl_3_): δ 7.94 (d, *J* = 8.1 Hz, 1H), 7.84 (d, *J* = 7.5 Hz, 1H), 7.77–7.70 (m, 2H), 7.61–7.50 (m, 4H), 7.48–7.30 (m, 5H), 7.00 (s, 1H), 6.62 (s, 1H), 5.67 (s, 2H), 3.97 (s, 2H); ^13^C NMR (100.6 MHz) (CDCl_3_): δ 144.8 (C), 144.1 (C), 143.8 (C), 142.6 (C), 140.2 (C), 140.1 (C), 136.0 (C), 134.7 (C), 131.2 (C), 129.1 (CH), 128.64 (CH), 128.55 (C), 127.9 (CH), 127.2 (CH), 125.7 (C), 125.35 (CH), 125.34 (CH), 124.4 (CH), 124.7 (q, *J_C-F_* = 273.0 Hz, C), 122.9 (q, *J_C-F_* = 31.3 Hz, C), 122.5 (CH), 120.72 (CH), 120.68 (CH), 119.7 (q, *J_C-F_* = 4.4 Hz, CH), 110.8 (q, *J_C-F_* = 4.4 Hz, CH), 110.0 (C), 104.2 (CH), 44.2 (CH_2_), 37.0 (CH_2_); ^19^F NMR (376.5 MHz) (CDCl_3_): δ −60.92 (s); HRMS: *m*/*z* [M + H]^+^ calcd for C_31_H_20_F_3_N_4_: 505.1635; found: 505.1647.

4-(5-phenyl-2-(trifluoromethyl)pyrrolo[3,2,1-*ij*][1,2,3]triazolo[4,5-*c*]quinolin-10(7*H*)-yl)benzonitrile **2f**: 80% yield; white solid; mp: 224–226 °C; IR (neat): 3176, 2284, 1240, 789, 655 cm^−1^; ^1^H NMR (400.13 MHz) (CDCl_3_): δ 7.84 (s, 1H), 7.67–7.52 (m, 9H), 7.00 (s, 1H), 6.73 (s, 1H), 5.75 (s, 2H); ^13^C NMR (100.6 MHz) (CDCl_3_): 142.7 (C), 140.3 (C), 136.9 (C), 135.9 (C), 135.0 (C), 131.1 (C), 130.1 (CH), 129.1 (CH), 129.1 (CH), 128.6 (CH), 127.0 (CH), 125.8 (C), 124.5 (q, *J_C-F_* = 273.0 Hz, C), 122.9 (q, *J_C-F_* = 31.8 Hz, C), 120.0 (q, *J_C-F_* = 4.3 Hz, CH), 110.6 (q, *J_C-F_* = 4.3 Hz, CH), 109.6 (C), 104.3 (CH), 44.2 (CH_2_); ^19^F NMR (376.5 MHz) (CDCl_3_): δ −60.93 (s); HRMS: *m*/*z* [M + H]^+^ calcd for C_25_H_15_F_3_N_5_: 442.1274; found: 442.1283.

5-phenyl-2-(trifluoromethyl)-10-(3,4,5-trimethoxyphenyl)-7,10-dihydropyrrolo[3,2,1*ij*][1,2,3]triazolo[4,5-*c*]quinoline **2g**: 99% yield; white solid; mp: 212–214 °C; IR (neat): 3125, 1602, 1336, 1126, 816 cm^−1^; ^1^H NMR (400.13 MHz) (CDCl_3_): δ 7.91 (s, 1H), 7.65 (s, 1H), 7.49 (s, 4H), 7.40 (s, 1H), 6.83 (s, 2H), 6.74 (s, 1H), 5.99 (s, 2H), 3.91 (s, 6H), 3.87 (s, 3H); ^13^C NMR (100.6 MHz) (CDCl_3_): δ 153.9 (C), 146.7 (C), 145.8 (C), 138.4 (C), 135.2 (C), 132.7 (C), 131.3 (C), 131.1 (C), 129.8 (CH), 129.2 (CH), 128.8 (CH), 124.0 (CH), 124.0 (q, *J_C-F_* = 273.0 Hz, C), 124.1 (q, *J_C-F_* = 31.8 Hz, C), 124.0 (q, *J_C-F_* = 3.6 Hz, CH), 119.9 (CH), 117.7 (q, *J_C-F_* = 3.6 Hz, CH), 105.0 (CH), 104.2 (C), 98.5 (CH), 61.0 (CH_3_), 56.4 (CH_3_), 41.5 (CH_2_); ^19^F NMR (376.5 MHz) (CDCl_3_): δ −60.78 (s); HRMS: *m*/*z* [M + H]^+^ calcd for C_27_H_22_F_3_N_4_O_3_: 507.1638; found: 507.1645.

10-(4-(*tert*-butyl)phenyl)-5-phenyl-2-(trifluoromethyl)-7,10-dihydropyrrolo[3,2,1-*ij*][1,2,3]triazolo[4,5-*c*]quinoline **2h**: 84% yield; orange solid; mp: 230–232 °C; IR (neat): 3152, 2978, 1325, 1254, 595 cm^−1^; ^1^H NMR (400.13 MHz) (CDCl_3_): δ 7.91 (s, 1H), 7.65 (s, 1H), 7.57–7.52 (m, 2H), 7.51–7.46 (m, 6H), 7.37 (s, 1H), 6.73 (s, 1H), 6.00 (s, 2H), 1.35 (s, 9H); ^13^C NMR (100.6 MHz) (CDCl_3_): δ 154.4 (C), 142.5 (C), 139.9 (C), 136.0 (C), 133.8 (C), 131.2 (C), 129.1 (CH), 128.71 (C), 128.62 (CH), 126.8 (CH), 125.67 (C), 125.45 (CH), 124.7 (q, *J_C-F_* = 273.2 Hz, C) 122.8 (q, *J_C-F_* = 31.5 Hz, C), 120.5 (d, *J_C-F_* = 4.0 Hz, C), 119.6 (q, *J_C-F_* = 3.7 Hz, CH), 110.6 (q, *J_C-F_* = 3.7 Hz, CH), 109.8 (C), 104.1 (CH), 44.2 (CH_2_), 35.1 (C), 31.3 (CH_3_); ^19^F NMR (376.5 MHz) (CDCl_3_): δ −60.93 (s); HRMS: *m*/*z* [M + H]^+^ calcd for C_28_H_24_F_3_N_4_: 473.1948; found: 473.1961.

methyl 10-benzyl-5-phenyl-7,10-dihydropyrrolo[3,2,1-*ij*][1,2,3]triazolo[4,5-*c*]quinoline-2-carboxylate **2i**: 78% yield; white solid; mp: 242–244 °C; IR (neat): 3042, 1768, 1225, 1015, 966 cm^−1^; ^1^H NMR (400.13 MHz) (CDCl_3_): δ 8.15 (s, 1H), 7.74 (s, 1H), 7.50–7.34 (m, 5H), 7.33–7.16 (m, 5H), 6.56 (s, 1H), 5.83 (s, 2H), 5.56 (s, 2H), 3.85 (s, 3H); ^13^C NMR (100.6 MHz) (CDCl_3_): δ 167.4 (C), 161.4 (C), 142.1 (C), 140.4 (C), 136.8 (C), 134.3 (C), 131.3 (C), 129.1 (CH), 129.0 (CH), 128.9 (CH), 128.6 (CH), 128.5 (CH), 127.1 (CH), 125.7 (C), 124.8 (CH), 122.6 (C), 115.5 (CH), 109.1 (C), 104.6 (CH), 53.6 (CH_2_), 52.1 (CH_3_), 44.3 (CH_2_); HRMS: *m*/*z* [M + H]^+^ calcd for C_26_H_21_N_4_O_2_: 421.1659; found: 421.1677.

methyl 5-phenyl-10-(3,4,5-trimethoxyphenyl)-7,10-dihydropyrrolo[3,2,1-*ij*][1,2,3]triazolo [4,5-*c*]quinoline-2-carboxylate **2j**: 68% yield; white solid; mp: 242–244 °C; IR (neat): 3108, 2897, 1759, 1047, 987 cm^−1^; ^1^H NMR (400.13 MHz) (CDCl_3_): δ 8.34 (s, 1H), 8.12 (s, 1H), 7.48 (s, 4H), 7.42 (s, 1H), 6.83 (s, 2H), 6.74 (s, 1H), 5.99 (s, 2H), 3.96 (s, 3H), 3.90 (s, 6H), 3.87 (s, 3H); ^13^C NMR (100.6 MHz) (CDCl_3_): δ 166.7 (C), 153.9 (C), 147.0 (C), 145.2 (C), 138.4 (C), 136.2 (C), 132.7 (C), 131.4 (C), 131.2 (C), 129.8 (CH), 129.1 (CH), 128.8 (CH), 128.6 (CH), 123.8 (C), 122.7 (CH), 119.9 (CH), 105.5 (CH), 103.7 (C), 98.5 (CH), 61.1 (CH_3_), 56.5 (CH_3_), 52.2 (CH_3_), 41.6 (CH_2_); HRMS: *m*/*z* [M + H]^+^ calcd for C_28_H_25_N_4_O_5_: 497.1819; found: 497.1826.

10-benzyl-2-methyl-5-phenyl-7,10-dihydropyrrolo[3,2,1-*ij*][1,2,3]triazolo[4,5-*c*]quinoline **2k**: 67% yield; white solid; mp: 250–252 °C; IR (neat): 3314, 3111, 1487, 1019, 971 cm^−1^; ^1^H NMR (400.13 MHz) (CDCl_3_): δ 7.65–7.42 (m, 5H), 7.42–7.22 (m, 6H), 6.91 (s, 1H), 6.53 (s, 1H), 5.91 (s, 2H), 5.67 (s, 2H), 2.37 (s, 3H); ^13^C NMR (100.6 MHz) (CDCl_3_): δ 140.9 (C), 140.5 (C), 134.7 (C), 133.3 (C), 132.0 (C), 129.7 (C), 129.1 (CH), 129.0 (CH), 128.9 (CH), 128.5 (CH), 128.41 (CH), 128.36 (CH), 126.8 (CH), 126.6 (C), 121.5 (CH), 116.0 (CH), 109.1 (C), 102.8 (CH), 53.4 (CH_2_), 44.1 (CH_2_), 21.7 (CH_3_); HRMS: *m*/*z* [M + H]^+^ calcd for C_25_H_21_N_4_: 377.1761; found: 377.1749. 

#### 3.3.2. Characterization Data of Final Compounds **3b**–**k**

5-(3,4,5-trimethoxyphenyl)-5,8-dihydrobenzo[3,4][1,2,3]triazolo[4′,5′:5,6]azepino [1,2-*a*]indole **3b**: 92% yield; yellow solid; mp: 192–194 °C; IR (neat): 1506, 1460, 1230, 1032, 748 cm^−1^; ^1^H NMR (400.13 MHz) (CDCl_3_): δ 7.81 (d, *J* = 7.8 Hz, 1H), 7.56 (d, *J* = 7.8 Hz, 1H), 7.49 (d, *J* = 8.4 Hz, 1H), 7.36 (t, *J* = 7.6 Hz, 1H), 7.22–7.15 (m, 2H), 7.05–6.99 (m, 2H), 6.77 (s, 1H), 6.53 (s, 2H), 5.33 (s, 2H), 3.80 (s, 3H), 3.65 (s, 6H); ^13^C NMR (100.6 MHz) (CDCl_3_): δ 153.8 (C), 144.6 (C), 138.9 (C), 138.5 (C), 136.9 (C), 133.7 (C), 132.2 (C), 132.1 (C), 131.6 (CH), 129.62 (CH), 128.6 (CH), 127.7 (CH), 127.6 (C), 122.7 (C), 122.6 (CH), 120.8 (CH), 120.2 (CH), 109.3 (CH), 103.9 (CH), 102.82 (CH), 61.10 (CH_3_), 56.40 (CH_3_), 39.4 (CH_2_). HRMS: *m*/*z* [M + H]^+^ calcd for C_26_H_23_N_4_O_3_: 439.1765; found: 439.1774.

5-(4-fluorophenyl)-5,8-dihydrobenzo[3,4][1,2,3]triazolo[4′,5′:5,6]azepino [1,2-*a*]indole **3c**: 87% yield; white solid; mp: 243–245 °C; IR (neat): 1596, 1457, 1237, 1032, 762, 669 cm^−1^; ^1^H NMR (400.13 MHz) (CDCl_3_): δ 7.80 (d, *J* = 7.9 Hz, 1H), 7.57 (d, *J* = 7.9 Hz, 1H), 7.49 (d, *J* = 7.34 Hz, 1H), 7.38–7.30 (m, 3H), 7.21 (t, *J* = 7.5 Hz, 1H), 7.14 (t, *J* = 7.1 Hz, 1H), 7.10–7.03 (m, 3H), 6.87 (d, *J* = 7.8 Hz, 1H), 6.76 (s, 1H), 5.34 (s, 2H); ^13^C NMR (100.6 MHz) (CDCl_3_): δ 162.9 (d, *J_C-F_* = 254 Hz, C), 144.8 (C), 138.5 (C), 137.0 (C), 133.9 (C), 132.8 (d, *J_C-F_* = 3.1 Hz, C), 132.5 (C), 131.8 (CH), 129.7 (CH), 128.5 (CH), 127.8 (C), 127.7 (CH), 127.1 (d, *J_C-F_* = 8.7 Hz, CH), 122.7 (C), 122.7 (CH), 121.0 (CH), 120.2 (CH), 116.8 (d, *J_C-F_* = 22.6 Hz, CH), 109.3 (CH), 104.0 (CH), 39.5 (CH_2_); ^19^F NMR (376.5 MHz) (CDCl_3_): δ −110.36 (m). HRMS: *m*/*z* [M + H]^+^ calcd for C_28_H_25_N_4_O_5_: 497.1819; found: 497.1827.

5-(2-fluoro-4-methylphenyl)-5,8-dihydrobenzo[3,4][1,2,3]triazolo[4′,5′:5,6]azepino [1,2-*a*]indole **3d**: 83% yield; orange solid; mp: 255–257 °C; IR (neat): 1506, 1456, 1230, 1019, 989 cm^−1^; ^1^H NMR (400.13 MHz) (CDCl_3_): δ 7.78 (d, *J* = 7.8 Hz, 1H), 7.56 (d, *J* = 7.8 Hz, 1H), 7.49 (d, *J* = 8.3 Hz, 1H), 7.34–7.26 (m, 2H), 7.20 (t, *J* = 7.4 Hz, 1H), 7.10 (td, *J*_1_ = 7.9 Hz, *J*_2_ = 0.8 Hz, 1H), 7.03 (t, *J* = 7.5 Hz, 1H), 6.99 (d, *J* = 8.0 Hz, 1H), 6.92–6.89 (m, 2H), 6.74 (s, 1H), 5.34 (s, 2H), 2.32 (s, 3H); ^13^C NMR (100.6 MHz) (CDCl_3_): δ 155.6 (d, *J_C-F_* = 254 Hz, C), 143.8 (C), 143.1 (d, *J_C-F_* = 7.41 Hz, C), 138.6 (C), 137.0 (C), 135.3 (C), 132.2 (C), 131.7 (CH), 129.7 (CH), 127.9 (C), 127.8 (d, *J_C-F_* = 8.1 Hz, CH), 126.9 (CH), 125.9 (d, *J_C-F_* = 3.54 Hz, CH), 122.9 (C), 122.5 (CH), 122.2 (d, *J_C-F_* = 12.56 Hz, C), 120.9 (CH), 120.1 (CH), 117.6 (d, *J_C-F_* = 18.30Hz, CH), 109.3 (CH), 104.0 (CH), 39.4 (CH_2_), 21.5 (CH_3_); ^19^F NMR (376.5 MHz) (CDCl_3_): δ −121.03 (bs). HRMS: *m*/*z* [M + H]^+^ calcd for C_24_H_18_FN_4_: 381.1510; found: 381.1531.

5-(4-methoxyphenyl)-5,8-dihydrobenzo[3,4][1,2,3]triazolo[4′,5′:5,6]azepino[1,2-*a*]indole **3e**: 96% yield; white solid; mp: 211–213 °C; IR (neat): 1509, 1458, 1250, 1032, 832 cm^−1^; ^1^H NMR (400.13 MHz) (CDCl_3_): δ 7.81 (d, *J*_1_ = 8.2 Hz, *J*_2_ = 0.6 Hz 1H), 7.59 (d, *J* = 7.8 Hz, 1H), 7.51 (d, *J* = 8.3 Hz, 1H), 7.36 (td, *J*_1_ = 7.7 Hz, *J*_2_ = 0.7 Hz, 1H), 7.27–7.20 (m, 3H), 7.13 (td, *J*_1_ = 7.5 Hz, *J*_2_ = 1.1 Hz, 1H), 7.06 (t, *J* = 7.5 Hz, 1H), 6.93–6.87 (m, 3H), 6.76 (s, 1H), 5.36 (s, 2H), 3.78 (s, 3H); ^13^C NMR (100.6 MHz) (CDCl_3_): 160.4 (C), 144.6 (C), 138.7 (C), 137.0 (C), 133.8 (C), 132.4 (C), 131.7 (CH), 129.7 (C), 129. (CH), 128.5 (CH), 127.9 (C), 127.7 (CH), 126.6 (CH), 123.1 (C), 122.6 (CH), 120.9 (CH), 120.2 (CH), 114.8 (CH), 109.4 (CH), 103.9 (CH), 55.7 (CH_3_), 39.5 (CH_2_). HRMS: *m*/*z* [M + H]^+^ calcd for C_24_H_19_N_4_O: 379.1553; found: 379.1565.

5-(2-chlorophenyl)-5,8-dihydrobenzo[3,4][1,2,3]triazolo[4′,5′:5,6]azepino[1,2-*a*]indole **3f**: 55% yield; white solid; mp: 210–212 °C; IR (neat): 1508, 1457, 1125, 1037, 764 cm^−1^; ^1^H NMR (400.13 MHz) (CDCl_3_): δ 7.83 (dd*, J*_1_= 7.85 Hz, *J*_2_= 0.54 Hz, 1H), 7.60 (d, *J =* 7.86 Hz, 1H), 7.54 (d, *J =* 8.40 Hz, 1H), 7.50–7.35 (m, 5H), 7.25 (td, *J*_1_= 15.39 Hz, *J*_2_= 7.67 Hz, *J*_3_= 0.92 Hz, 1H), 7.13–7.06 (m, 2H), 6.82 (dd, *J*_1_= 8.05 Hz, *J*_2_= 0.53 Hz, 1H), 6.77 (s, 1H), 5.49–5.34 (m, 2H); ^13^C NMR (100.6 MHz) (CDCl_3_): δ 14.72 (C), 138.64 (C), 137.13 (C), 135.56 (C), 134.78 (C), 132.36 (C), 131.81 (CH), 131.71 (CH), 131.61 (C), 131.05 (CH), 129.78 (CH), 129.21 (CH), 128.22 (CH), 127.97 (C), 127.95 (CH), 127.02 (CH), 122.96 (C), 122.65 (CH), 121.04 (CH), 120.20 (CH), 109.43 (CH), 104.13 (CH), 39.54 (CH_2_). HRMS: *m*/*z* [M + H]^+^ calcd for C_23_H_16_ClN_4_: 383.1058; found: 383.1073.

5-(4-chlorophenyl)-3-methyl-5,8-dihydrobenzo[3,4][1,2,3]triazolo[4′,5′:5,6]azepino [1,2-*a*]indole **3g**: 86% yield; yellow solid; mp: 281–283 °C; IR (neat): 1501, 1460, 1228, 989, 750 cm^−1^; ^1^H NMR (400.13 MHz) (CDCl_3_): δ 7.63 (s, 1H), 7.57 (d, *J* = 7.8 Hz, 1H), 7.49 (d, *J* = 8.3 Hz, 1H), 7.37–7.35 (m, 2H), 7.29–7.27 (m, 2H), 7.23–7.19 (m, 1H), 7.05 (t, *J* = 7.4 Hz, 1H), 6.97 (dd, *J*_1_ = 7.9 Hz, *J*_2_ = 0.9 Hz, 1H), 6.76 (d, *J* = 7.8 Hz, 2H), 5.33 (s, 2H), 2.36 (s, 3H); ^13^C NMR (100.6 MHz) (CDCl_3_): δ 144.7 (C), 140.0 (C), 138.6 (C), 136.9 (C), 135.6 (C), 135.3 (C), 133.9 (C), 132.4 (C), 132.3 (CH), 129.9 (CH), 128.8 (CH), 128.5 (CH), 127.8 (C), 126.3 (CH), 122.6 (CH), 120.9 (CH), 120.2 (CH), 119.9 (C), 109.3 (CH), 103.8 (CH), 39.4 (CH_2_), 21.5 (CH_3_). HRMS: *m*/*z* [M + H]^+^ calcd for C_24_H_18_ClN_4_: 397.1214; found: 397.1203.

5-(4-methoxyphenyl)-3-methyl-5,8-dihydrobenzo[3,4][1,2,3]triazolo[4′,5′:5,6]azepino [1,2-*a*]indole **3h**: 88% yield; yellow solid; mp: 237–238 °C; IR (neat): 1513, 1555, 1034, 742, 670 cm^−1^; ^1^H NMR (400.13 MHz) (CDCl_3_): δ 7.63 (s, 1H), 7.57 (d, *J* = 7.8 Hz, 1H), 7.50 (d, *J* = 8.3 Hz, 1H), 7.26–7.20 (m, 3H), 7.05 (t, *J* = 7.4, 1H), 6.95 (dd, *J*_1_ = 7.9 Hz, *J*_2_ = 1.1 Hz, 1H), 6.91–6.87 (m, 2H), 6.80 (d, *J* = 8.05 Hz, 1H), 6.76 (s, 1H), 5.33 (s, 2H), 3.78 (s, 3H), 2.35 (s, 3H); ^13^C NMR (100.6 MHz) (CDCl_3_): δ 160.4 (C), 144.2 (C), 139.7 (C), 138.9 (C), 136.9 (C), 133.9 (C), 132.3 (C), 132.1 (CH), 129.8 (C), 128.7 (CH), 128.4 (CH), 127.9 (C), 126.5 (CH), 122.5 (CH), 120.9 (CH), 120.4 (C), 120.1 (CH), 114.8 (CH), 109.4 (CH), 103.7 (CH), 55.7 (CH_3_), 39.6 (CH_2_), 21.5 (CH_3_). HRMS: *m*/*z* [M + H]^+^ calcd for C_25_H_21_N_4_O: 393.1710; found: 393.1722.

5-benzyl-12-methyl-5,8-dihydrobenzo[3,4][1,2,3]triazolo[4′,5′:5,6]azepino[1,2-*a*] indole **3i**: 90% yield; white solid; mp: 163–165 °C; IR (neat): 1510, 1452, 1032, 762, 738 cm^−1^; ^1^H NMR (400.13 MHz) (CDCl_3_): δ 7.80 (d, *J* = 7.9 Hz, 1H), 7.43–7.37 (m, 2H), 7.33 (s, 1H), 7.3–7.24 (m, 5H), 7.14–7.12 (m, 2H), 7.03 (dd, *J*_1_ = 8.5 Hz, *J*_2_ = 1.3 Hz, 1H), 6.61 (s, 1H), 5.59 (s, 2H), 5.29 (s, 2H), 2.37 (s, 3H); ^13^C NMR (100.6 MHz) (CDCl_3_): δ 144.3 (C), 138.4 (C), 135.5 (C), 134.6 (C), 132.7 (C), 131.9 (CH), 129.8 (CH), 129.3 (C), 129.2 (CH), 128.4 (CH), 128.1 (C), 128.0 (CH), 127.7 (CH), 126.8 (CH), 124.3 (CH), 122.9 (C), 120.5 (CH), 109.1 (CH), 103.3 (CH), 52.5 (CH_2_), 39.6 (CH_2_), 21.5 (CH_3_). HRMS: *m*/*z* [M + H]^+^ calcd for C_25_H_21_N_4_: 377.1761; found: 377.1778.

12-methyl-5-(3,4,5-trimethoxyphenyl)-5,8-dihydrobenzo[3,4][1,2,3]triazolo[4′,5′:5,6] azepino[1,2-*a*]indole **3j**: 95% yield; white solid; mp: 234–236 °C; IR (neat): 1596, 1450, 1224, 1024, 1031 cm^−1^; ^1^H NMR (400.13 MHz) (CDCl_3_): δ (dd, *J*_1_ =7.9 Hz, *J*_2_ = 0.78 Hz, 1H), 7.51–7.46 (m, 3H), 7.29–7.25 (m, 1H), 7.16 (dd, *J*_1_ = 8.5 Hz, *J*_2_ = 1.2 Hz, 1H), 7.10 (dd, *J*_1_ = 7.9 Hz, *J*_2_ = 0.8 Hz, 1H), 6.81 (s, 1H), 6.65 (s, 2H), 5.44 (s, 2H), 3.93 (s, 3H), 3.78 (s, 6H), 2.49 (s, 3H); ^13^C NMR (100.6 MHz) (CDCl_3_): δ153.8 (C), 144.7 (C), 138.9 (C), 138.6 (C), 135.5 (C), 133.8 (C), 132.4 (C), 132.3 (C), 131.6 (CH), 129.6 (C), 129.5 (CH), 128.7 (CH), 128.1 (C), 127.5 (CH), 124.5 (CH), 122.7 (C), 120.4 (CH), 109.1 (CH), 103.4 (CH), 102.9 (CH), 61.2 (CH_3_), 56.5 (CH_3_), 39.6 (CH_2_), 21.5 (CH_3_). HRMS: *m*/*z* [M + H]^+^ calcd for C_27_H_25_N_4_O_3_: 453.1921; found: 453.1907.

5-(4-chlorophenyl)-12-methyl-5,8-dihydrobenzo[3,4][1,2,3]triazolo[4′,5′:5,6] azepino [1,2-*a*]indole **3k**: 95% yield; white solid; mp: 215–217 °C; IR (neat): 1499, 1456, 1120, 1041, 789 cm^−1^; ^1^H NMR (400.13 MHz) (CDCl_3_): δ (dd*, J*_1_ = 7.9 Hz, *J*_2_ = 0.7 Hz, 1H), 7.39–7.36 (m, 5H), 7.30–7.28 (m*,* 2H), 7.17–7.13 (m, 1H), 7.05 (dd*, J*_1_= 8.5 Hz, *J*_2_ = 1.1 Hz, 1H), 6.88 (dd*, J*_1_ = 7.8 Hz, *J*_2_ = 0.8 Hz, 1H), 6.68 (*s*, 1H), 5.32 (*s*, 2H), 2.37 (*s*, 3H); ^13^C NMR (100.6 MHz) (CDCl_3_): δ 145.1 (C), 138.4 (C), 135.7 (C), 135.6 (C), 135.3 (C), 133.8 (C), 132.6 (C), 131.8 (CH), 130.0 (CH), 129.8 (CH), 129.5 (C), 128.6 (CH), 128.1 (C), 127.7 (CH), 126.4 (CH), 124.5 (CH), 122.6 (C), 120.5 (CH), 109.0 (CH), 103.5 (CH), 39.6 (CH_2_), 21.5 (CH_3_). HRMS: *m*/*z* [M + H]^+^ calcd for C_24_H_18_ClN_4_: 397.1214; found: 397.1228.

5-(2-fluoro-4-methylphenyl)-12-methyl-5,8-dihydrobenzo[3,4][1,2,3]triazolo [4′,5′:5,6]azepino[1,2-*a*]indole **3l**: 90% yield; white solid; mp: 231–233 °C; IR (neat): 1499, 1448, 1338, 1053, 767 cm^−1^; ^1^H NMR (400.13 MHz) (CDCl_3_): δ 7.78 (dd, *J*_1_ = 8.0 Hz, *J*_2_ = 0.8 Hz, 1H), 7.39–7.27 (m, 4H), 7.12–7.08 (m, 1H), 7.05–6.99 (m, 2H), 6.94–6.89 (m, 2H), 6.66 (s, 1H), 5.33 (s, 2H), 2.36 (s, 3H), 2.34 (s, 3H); ^13^C NMR (100.6 MHz) (CDCl_3_): δ 155.7 (*J_C-F_* = 253.0 Hz, C), 143.9 (C), 143.14 (*J_C-F_* = 7.5 Hz, C), 138.6 (C), 135.5 (C), 135.3 (C), 132.i4 (C), 131.7 (CH), 129.6 (CH), 129.4 (C), 128.2 (C), 127.8 (*J_C-F_* = 10.2 Hz, CH), 126.9 (CH), 125.9 (*J_C-F_* = 3.16 Hz, CH), 124.3 (CH), 122.9 (C), 122.3 (*J_C-F_* = 12.5 Hz, C), 120.5 (CH), 117.6 (*J_C-F_* = 18.8 Hz, CH), 109.0 (CH), 103.5 (CH_3_), 39.5 (CH_2_), 21.5 (CH_3_); ^19^F NMR (376.5 MHz) (CDCl_3_): δ −121.01 (bs). HRMS: *m*/*z* [M + H]^+^ calcd for C_25_H_20_FN_4_: 395.1666; found: 395.1681.

5-benzyl-3-(trifluoromethyl)-5,8-dihydrobenzo[3,4][1,2,3]triazolo[4′,5′:5,6]azepino [1,2-*a*]indole **3m**: 85% yield; gray solid; mp: 227–229 °C; IR (neat): 1455, 1338, 1124, 1031, 738 cm^−1^; ^1^H NMR (400.13 MHz) (CDCl_3_): δ 7.90 (d, *J* = 8.2 Hz, 1H), 7.62 (dd, *J*_1_ = 8.3 Hz, *J*_2_ = 1.2 Hz, 1H), 7.57 (d, *J* = 8.06 Hz, 1H), 7.54–7.47 (m, 2H), 7.33–7.23 (m, 4H), 7.21 (m, 2H), 7.09–7.05 (m, 1H), 6.67 (s, 1H), 5.58 (s, 2H), 5.33 (s, 2H); ^13^C NMR (100.6 MHz) (CDCl_3_): δ 144.6 (C), 137.4 (C), 137.0 (C), 135.6 (C), 134.8 (C), 133.4 (C), 132.3 (CH), 131.2 (C), 130.0 (*J_C-F_* = 33.0 Hz) (C), 129.4 (CH), 128.8 (CH), 127.7 (C), 127.1 (CH), 126.2 (*J_C-F_* = 3.5 Hz, CH), 125.1 (*J_C-F_* = 3.6 Hz, CH), 123.8 (*J_C-F_* = 272.0 Hz, C), 123.4 (CH), 121.3 (CH), 120.5 (CH), 109.5 (CH), 105.2 (CH), 53.1 (CH_2_), 39.5 (CH_2_); ^19^F NMR (376.5 MHz) (CDCl_3_): δ −62.64 (s). HRMS: *m*/*z* [M + H]^+^ calcd for C_25_H_18_F_3_N_4_: 431.1478; found: 431.1485.

5-(4-methoxyphenyl)-3-(trifluoromethyl)-5,8-dihydrobenzo[3,4][1,2,3]triazolo [4′,5′:5,6]azepino[1,2-*a*]indole **3n**: 84% yield; yellow solid; mp: 260–262 °C; IR (neat): 1513, 1336, 1296, 1251, 1123 cm^−1^; ^1^H NMR (400.13 MHz) (CDCl_3_): δ 7.93 (d, *J* = 8.3 Hz, 1H), 7.62–7.57 (m, 2H), 7.53 (d, *J* = 8.4 Hz, 1H), 7.29–7.22 (m, 3H), 7.14–7.07 (m, 2H), 6.95–6.90 (m, 2H), 6.85 (s, 1H), 5.40 (s, 2H), 3.79 (s, 3H); ^13^C NMR (100.6 MHz) (CDCl_3_): δ 160.8 (C), 144.8 (C), 137.4 (C), 137.2 (C), 135.5 (C), 132.76 (C), 132.0 (CH), 129.6 (q, *J_C-F_* = 33.0 Hz, C), 129.1 (C), 127.8 (C), 126.6 (CH), 125.8 (*q*, *J_C-F_* = 3.5 Hz, CH), 125.5 (*q*, *J_C-F_* = 3.8 Hz, CH), 123.5 (C), 123.4 (CH), 123.2 (*q*, *J_C-F_* = 270.3 Hz), 121.3 (CH), 120.6 (CH), 115.09 (CH), 109.53 (CH), 105.41 (CH), 55.8 (CH3), 39.6 (CH_2_); ^19^F NMR (376.5 MHz) (CDCl_3_): δ −62.24 (s). HRMS: *m*/*z* [M + H]^+^ calcd for C_25_H_18_F_3_N_4_O: 447.1427; found: 447.1411.

12-chloro-5-(4-chlorophenyl)-5,8-dihydrobenzo[3,4][1,2,3]triazolo[4′,5′:5,6]azepino [1,2-*a*]indole **3o**: 93% yield; yellow solid; mp: 273–275 °C; IR (neat): 1497, 1333, 992, 760, 539 cm^−1^; ^1^H NMR (400.13 MHz) (CDCl_3_): δ 7.81 (d, *J*_1_ = 7.9 Hz, 1H), 7.55 (d, *J* = 1.8 Hz, 1H), 7.43–7.38 (m, 4H), 7.4–7.28 (m, 2H), 7.20–7.16 (m, 2H), 6.91 (dd, *J*_1_ = 7.9 Hz, *J*_2_ = 0.9 Hz, 1H), 6.71 (s, 1H), 5.33 (s, 2H); ^13^C NMR (100.6 MHz) (CDCl_3_): δ 144.8 (C), 139.7 (C), 135.8 (C), 135.4 (C), 135.1 (C), 133.8 (C), 132.0 (C), 131.9 (CH), 130.0 (CH), 129.9 (CH), 128.7 (C), 128.6 (CH), 128.2 (CH), 126.3 (CH), 125.9 (C), 123.0 (CH), 122.7 (C), 120.3 (CH), 110.4 (CH), 103.5 (CH), 39.8 (CH_3_). HRMS: *m*/*z* [M + H]^+^ calcd for C_23_H_15_Cl_2_N_4_: 417.0668; found: 417.0653.

12-chloro-5-(3,4,5-trimethoxyphenyl)-5,8-dihydrobenzo[3,4][1,2,3]triazolo[4′,5′:5,6] azepino[1,2-*a*]indole **3p**: 92% yield; yellow solid; mp: 232–234 °C; IR (neat): 1605, 1461, 1125, 826, 774 cm^−1^; ^1^H NMR (400.13 MHz) (CDCl_3_): δ 7.81 (dd, *J*_1_ = 7.9 Hz, *J*_2_ = 0.7 Hz, 1H), 7.54 (d, *J* = 1.9 Hz, 1H), 7.43–7.38 (m, 2H); 7.24–7.14 (m, 2H); 7.02 (dd, *J*_1_ = 7.9 Hz, *J*_2_ = 0.7 Hz; 1H), 6.72 (s, 1H), 6.56 (s, 2H), 5.33 (s, 2H), 3.83 (s, 3H), 3.68 (s, 6H); ^13^C NMR (100.6 MHz) (CDCl_3_): δ 153.9 (C), 144.4 (C), 139.9 (C), 139.0 (C), 135.3 (C), 133.7 (C), 132.1 (C), 131.8 (C), 131.7 (CH), 129.8 (CH), 128.7 (CH), 128.7 (C), 128.0 (CH), 125.8 (C), 122.9 (CH), 122.8 (C), 120.2 (CH), 110.4 (CH), 103.4 (CH), 102.9 (CH), 61.2 (CH_3_), 56.5 (CH_3_), 39.8 (CH_2_). HRMS: *m*/*z* [M + H]^+^ calcd for C_26_H_22_ClN_4_O_3_: 473.1375; found: 473.1389.

methyl 5-(4-methoxyphenyl)-5,8-dihydrobenzo[3,4][1,2,3]triazolo[4′,5′:5,6] azepino[1,2-*a*]indole-12-carboxylate **3q**: 94% yield; white solid; mp: 200–202 °C; IR (neat): 1703, 1508, 1243, 1053, 1032 cm^−1^; ^1^H NMR (400.13 MHz) (CDCl_3_): δ 8.34 (d, *J* = 1.1 Hz, 1H), 7.90 (dd, *J*_1_ = 8.8 Hz, *J*_2_ = 1.4 Hz, 1H), 7.79 (d, *J* = 7.9 Hz, 1H), 7.49 (d, *J* = 8.8 Hz, 1H), 7.35 (dt, *J*_1_ = 8.0 Hz, *J*_2_ = 1.1 Hz, 1H), 7.23 (d, *J* = 8.8 Hz, 2H), 7.15 (dt, *J*_1_ = 8.0 Hz, *J*_2_ = 1.1 Hz, 1H), 6.93–6.87 (m, 3H), 6.82 (s, 1H), 5.35 (s, 2H), 3.84 (s, 3H), 3.76 (s, 3H); ^13^C NMR (100.6 MHz) (CDCl_3_): δ 168.0 (C), 160.5 (C), 144.1 (C), 140.2 (C), 139.2 (C), 133.7 (C), 131.8 (CH), 131.7 (C), 129.6 (CH), 129.5 (C), 128.5 (CH), 128.1 (CH), 127.4 (C), 126.5 (CH), 124.1 (CH), 123.7 (CH), 123.2 (C), 122.1 (C), 114.8 (CH), 109.0 (CH), 105.1 (CH), 55.7 (CH_3_), 51.9 (CH_3_), 39.9 (CH_2_). HRMS: *m*/*z* [M + H]^+^ calcd for C_26_H_21_N_4_O_3_: 437.1608; found: 437.1621.

#### 3.3.3. Characterization Data of Post-Synthetic Derivatives Compounds **22a** and **23a**–**c**

4-(4-(pyrrolo[3,2,1-*ij*][1,2,3]triazolo[4,5-*c*]quinolin-10(7*H*)-yl)phenyl)morpholine **22a**: 72% yield; yellow solid; mp: 190–192 °C; IR (neat): 2876, 1582, 1236, 1132, 991 cm^−1^; ^1^H NMR (400.13 MHz) (CDCl_3_): δ 7.63–7.53 (m, 1H), 7.51–7.30 (m, 5H), 7.10 (d, *J* = 3.2 Hz, 1H), 7.02–6.94 (m, 2H), 6.75 (t, *J* = 7.6 Hz, 1H), 6.64 (d, *J* = 7.3 Hz, 1H), 6.47 (d, *J* = 3.2 Hz, 1H), 5.68 (s, 2H), 3.83 (t, *J* = 4.8 Hz, 4H), 3.22 (dd, *J* = 6.1, 3.6 Hz, 4H); ^13^C NMR (100.6 MHz) (CDCl_3_): δ 152.4 (C), 139.0 (C), 133.6 (C), 132.9 (C), 132.2 (CH), 132.1 (CH), 132.0 (CH), 131.9 (CH), 129.9 (C), 128.6 (CH), 128.5 (CH), 128.4 (C), 126.9 (CH), 126.8 (CH), 125.9 (C), 122.5 (CH), 119.9 (CH), 115.3 (CH), 114.3 (CH), 109.4 (C), 103.6 (CH), 66.7 (CH_2_), 48.5 (CH_2_), 44.6 (CH_2_). HRMS: *m*/*z* [M + H]^+^ calcd for C_21_H_20_N_5_O: 358.1662; found: 358.1654. 

5-(4′-methoxy-[1,1′-biphenyl]-4-yl)-5,8-dihydrobenzo[3,4][1,2,3]triazolo[4′,5′:5,6] azepino[1,2-*a*]indole **23a**: 96% yield; yellow solid; mp: 248–250 °C; IR (neat): 1460, 1340, 1058, 763, 740 cm^−1^; ^1^H NMR (400.13 MHz) (CDCl_3_): δ 7.84 (d, *J* = 7.7 Hz, 1H), 7.61–7.57 (m, 3H), 7.53 (d, *J* = 8.6 Hz, 1H), 7.49 (d, *J* = 8.4 Hz, 2H), 7.40–7.38 (m, 3H), 7.24 (td, *J*_1_ = 8.2 Hz, *J*_3_ = 0.8 Hz, 1H), 7.18 -7.16 (m, 1H), 7.07 (t, *J* = 7.4 Hz, 1H), 7.00 (dd, *J*_1_ = 7.9 Hz, *J*_2_ = 0.9 Hz, 1H), 6.93 (d, *J* = 7.7 Hz, 2H), 6.79 (s, 1H), 5.39 (s, 2H), 3.79 (s, 3H);^13^C NMR (100.6 MHz) (CDCl_3_): δ 159.9 (C), 144.9 (C), 142.2 (C), 138.7 (C), 137.0 (C), 135.2 (C), 133.8 (C), 131.4 (C), 132.0 (C), 131.8 (CH), 129.6 (CH), 128.7 (CH), 128.3 (CH), 127.8 (CH), 125.4 (CH), 123.1 (C), 122.7 (CH), 121.0 (CH), 120.2 (CH), 114.6 (CH), 109.4 (CH), 103.9 (CH), 55.5 (CH_3_), 39.6 (CH_2_). HRMS: *m*/*z* [M + H]^+^ calcd for C_30_H_23_N_4_O: 455.1866; found: 455.1879. 

5-([1,1′-biphenyl]-4-yl)-5,8-dihydrobenzo[3,4][1,2,3]triazolo[4′,5′:5,6]azepino[1,2-*a*]indole **23b**: 98% yield; white solid; mp: 220–222 °C; IR (neat): 1439, 1160, 1054, 995, 767 cm^−1^; ^1^H NMR (400.13 MHz) (CDCl_3_): δ 7.84 (d, *J* = 7.9 Hz, 1H), 7.63–7.59 (m, 3H), 7.55–7.51 (m, 3H), 7.43–7.37 (m, 5H), 7.33 (m, 1H), 7.23 (td, *J*_1_ = 7.7 Hz, *J*_2_ = 0.8 Hz, 1H), 7.18–7.14 (m, 1H), 7.07 (t, *J* = 7.5 Hz, 1H), 7.00 (dd, *J*_1_ = 7.9 Hz, *J*_2_ = 0.6 Hz, 1H), 6.80 (s, 1H), 5.38 (s, 2H); ^13^C NMR (100.6 MHz) (CDCl_3_): δ 144.8 (C), 142.5 (C), 139.5 (C), 138.6 (C), 136.9 (C), 135.7 (C), 133.7 (C), 132.4 (C), 131.7 (CH), 129.6 (CH), 129.0 (CH), 128.7 (CH), 128.2 (CH), 128.1 (CH), 127.8 (C), 127.7 (CH), 127.2 (CH), 125.3 (CH), 122.9 (C), 122.6 (CH), 120.9 (CH), 120.1 (CH), 109.3 (CH), 103.8 (CH), 39.5 (CH_2_). HRMS: *m*/*z* [M + H]^+^ calcd for C_29_H_21_N_4_: 425.1766; found: 425.1753.

5-(4-(thiophen-2-yl)phenyl)-5,8-dihydrobenzo[3,4][1,2,3]triazolo[4′,5′:5,6]azepino [1,2-*a*]indole **23c**: 71% yield; gray solid; mp: 218–220 °C; IR (neat): 1458, 1319, 1032, 994, 728 cm^−1^; ^1^H NMR (400.13 MHz) (CDCl_3_): δ 7.83 (d, *J* = 8.0 Hz, 1H), 7.62–7.59 (m, 3H), 7.52 (d, *J* = 8.3, 1H), 7.46–7.44 (m, 1H), 7.39–7.32 (m, 5H), 7.23 (dt, *J*_1_ = 6.9 Hz, *J*_2_ = 0.8 Hz, 1H), 7.17–7.12 (m, 1H), 7.07 (t, *J* = 7.3 Hz, 1H), 6.97 (d, *J* = 7.86 Hz, 1H), 6.79 (s, 1H), 5.33 (s, 2H); ^13^C NMR (100.6 MHz) (CDCl_3_): δ 144.9 (C), 140.8 (C), 138.6 (C), 137.2 (C), 137.0 (C), 135.4 (C), 133.8 (C), 132.4 (C), 131.8 (CH), 129.6 (CH), 128.7 (CH), 127.9 (C), 127.8 (CH), 127.5 (CH), 127.0 (CH), 126.2 (CH), 126.0 (C),125.5 (CH), 122.7 (CH), 121.6 (CH), 121.0 (CH), 120.2 (CH), 109.4 (CH), 103.9 (CH), 39.6 (CH_2_). HRMS: *m*/*z* [M + H]^+^ calcd for C_27_H_19_N_4_S: 431.1330; found: 431.1346.

## 4. Conclusions

We have developed an efficient protocol for the synthesis of polysubstituted 7,10-dihydropyrrolo[3,2,1-*ij*][1,2,3]triazolo[4,5-*c*]quinoline and 5,8-dihydrobenzo[3,4][1,2,3]triazolo[4′,5′:5,6]azepino[1,2-*a*]indole from bromo-substituted *N*-propargyl-indoles. The reaction conditions exhibit broad functional group tolerance, accommodating halogens, alkoxy, cyano, ketone, and ester functionalities, with yields from good to high. Notably, their compatibility with chloro substituent provides a valuable handle for further molecular diversification through transition metal-catalyzed transformations, expanding the synthetic utility of this methodology.

## Figures and Tables

**Table 1:** Optimization studies for the synthesis of 10-benzyl-7,10-dihydropyrrolo[3,2,1-*ij*][1,2,3]triazolo[4,5-*c*]quinoline **1a** from 1-((1-benzyl-1*H*-1,2,3-triazol-4-yl)methyl)-7-bromo-1*H*-indole **4a** ^a^.

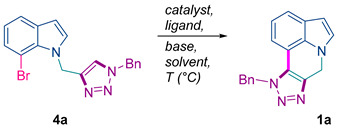							
Entry	Catalyst	Ligand	Base	Solvent	T (°C)	Time (h)	Yield (%) ^b^
1 ^c^	CuI	1,10-Phen	Li_2_CO_3_	DMF	120	48	-
2 ^c^	CuI	1,10-Phen	K_2_CO_3_	DMSO	120	48	-
3 ^c^	CuI	DMEDA	Cs_2_CO_3_	DMF	120	48	-
4 ^c^	CuOAc	1,10-Phen	K_2_CO_3_	DMF	120	48	-
5 ^c^	CuBr	1,10-Phen	K_2_CO_3_	DMF	120	48	-
6	Pd(OAc)_2_	DavePhos	Cs(OAc)	DMF	120	2	14 ^d^
7	Pd(OAc)_2_	PPh_3_	Cs(OAc)	DMF	120	1	68
8	Pd(OAc)_2_	PPh_3_	Cs(OAc)	DMSO	120	1	80
9	Pd(OAc)_2_	PPh_3_	Cs(OAc)	DMSO	100	2	69
10	Pd(OAc)_2_	PPh_3_	Cs(OAc)	DMSO	80	4	58
11	Pd(OAc)_2_	PPh_3_	Cs(OAc)	1,4-dioxane	100	24	-
12	Pd(OAc)_2_	PPh_3_	Cs(OAc)	MeCN	100	2	25
13 ^e^	Pd(OAc)_2_	PPh_3_	Cs(OAc)	DMSO	120	1	60

^a^ Unless otherwise stated, reactions were carried out on a 0.30 mmol scale under an argon atmosphere using 0.05 equiv. of catalyst, 0.20 equiv. of ligand, 2.0 equiv. of base in 2.0 mL of solvent. ^b^ Yields are given for isolated products. ^c^ 0.10 equiv. of ligand. ^d^ 1-((1-benzyl-1*H*-1,2,3-triazol-4-yl)methyl)-1*H*-indole was isolated in 42%. ^e^ The reaction was carried out using 0.02 equiv. of catalyst, 0.08 equiv. of ligand, and 2 equiv. of base.

**Table 2:** Synthesis of 10-substituted-7,10-dihydropyrrolo[3,2,1-*ij*][1,2,3]triazolo[4,5-*c*]quinoline **1** from **4** through a palladium-catalyzed C–H activation ^a,b^.

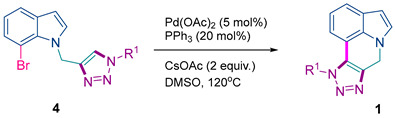
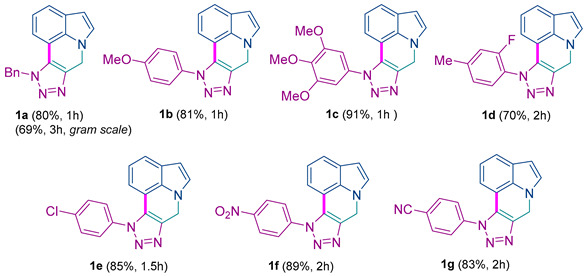

^a^ Unless otherwise stated, reactions were carried out on a 0.30 mmol scale under an argon atmosphere using 0.05 equiv. of Pd(OAc)_2_, 0.20 equiv. of PPh_3_, 2.0 equiv. of CsOAc in 2.0 mL of anhydrous DMSO at 120 °C. ^b^ Yields are given for isolated products.

**Table 3:** Synthesis of functionalized 2,5,10-trisubstituted-7,10-dihydropyrrolo[3,2,1-*ij*][1,2,3]triazolo[4,5-*c*]quinoline **2** from **5** through a palladium-catalyzed C–H activation ^a,b^.

** 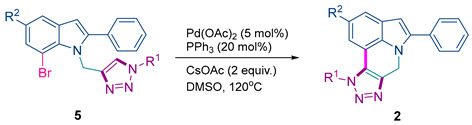 **
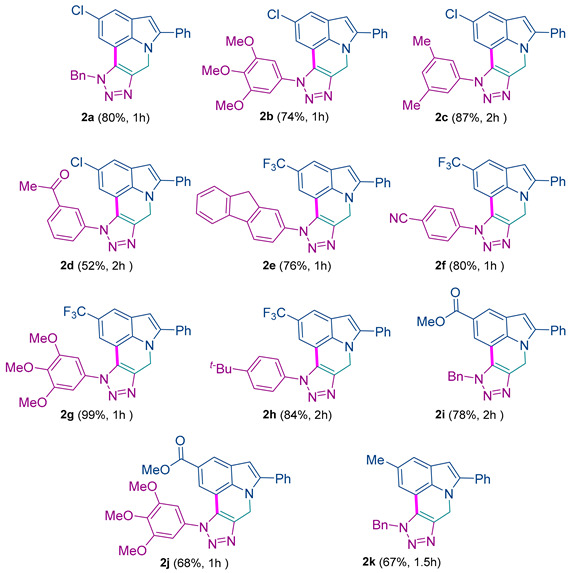

^a^ Unless otherwise stated, reactions were carried out on a 0.30 mmol scale under an argon atmosphere using 0.05 equiv. of Pd(OAc)_2_, 0.20 equiv. of PPh_3_, and 2.0 equiv. of CsOAc in 2.0 mL of anhydrous DMSO at 120 °C. ^b^ Yields are given for isolated products.

**Table 4:** Synthesis of polysubstituted 5,8-dihydrobenzo[3,4][1,2,3]triazolo[4′,5′:5,6]azepino[1,2-*a*]indole **3** from **6** through a palladium-catalyzed C–H activation ^a,b^.

** 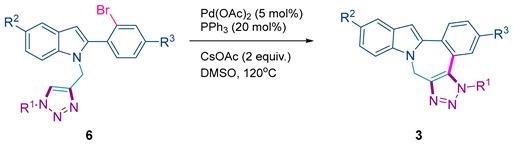 **
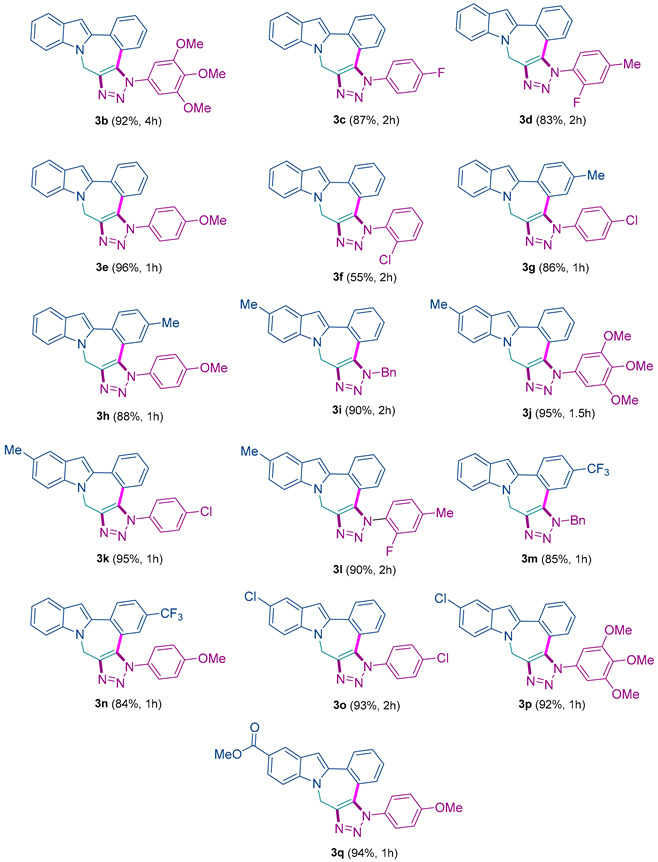

^a^ Unless otherwise stated, reactions were carried out on a 0.30 mmol scale under an argon atmosphere using 0.05 equiv. of Pd(OAc)_2_, 0.20 equiv of PPh_3_, and 2.0 equiv. of CsOAc in 2.0 mL of anhydrous DMSO at 120 °C. ^b^ Yields are given for isolated products.

## Data Availability

Data are contained within the article or Supplementary Materials.

## Supplementary Materials

The following supporting information can be downloaded at: https://www.mdpi.com/article/10.3390/molecules30122588/s1. References [39,43,44] are cited in Supplementary Materials.
