# Supplementary material for: Construction of 1,2,3-Triazole-Embedded Polyheterocyclic Compounds via CuAAC and C–H Activation Strategies
Source: Molecules. 2025 Jun 13;30(12):2588. doi: 10.3390/molecules30122588 (PMC12195963; doi:10.3390/molecules30122588)

Construction of 1,2,3-Triazole-Embedded Polyheterocyclic Compounds via CuAAC and C–H Activation Strategies

Antonia Iazzetti [1,2], Dario Allevi [1], Giancarlo Fabrizi [3,*], Yuri Gazzilli [3], Antonella Goggiamani [3], Federico Mar-rone [3], Francesco Stipa [3], Karim Ullah [3,†] and Roberta Zoppoli [3]

[1] Dipartimento di Scienze Biotecnologiche di Base, Cliniche Intensivologiche e Perioperatorie, Università Cattolica del Sacro Cuore, L.go Francesco Vito 1, 00168 Rome, Italy; antonia.iazzetti@unicatt.it (A.I.); dario.allevi@unicatt.it (D.A.)

[2] Policlinico Universitario 'A. Gemelli' Foundation-IRCCS, 00168 Rome, Italy

[3] Dipartimento di Chimica e Tecnologie del Farmaco, Sapienza, Università di Roma, P. le A. Moro 5, 00185 Rome, Italy; yuri.gazzilli@uniroma1.it (Y.G.); antonella.goggiamani@uniroma1.it (A.G.); federico.marrone@uniroma1.it (F.M.); f.stipa@outlook.it (F.S.); karim.ullah@uniroma1.it (K.U.); roberta.zoppoli@uniroma1.it (R.Z.)

* Correspondence: giancarlo.fabrizi@uniroma1.it

† Current Address: Department of Chemistry, Sustainable Chemistry for Metals and Molecules (SCM2), KU Leuven, Celestijnenlaan 200F—Box 2404, B-3001 Leuven, Belgium.

Contents

1. GENERAL INFORMATION S2

1.1. Reagents and methods S2

2. SYNTHETIC PROCEDURES FOR STARTING MATERIALS S2

2.1 General procedure for the preparation of 7-bromo-1-((1-substituted-1*H*-1,2,3-triazol-4-yl)methyl)-2-phenyl-1*H*-indole **5** S2

2.1.1 Typical one-pot four-step procedure for the preparation of 7-bromo-1-((1-substituted-1*H*-1,2,3-triazol-4-yl)methyl)-2-phenyl-1*H*-indole synthesis of 1-((1-benzyl-1*H*-1,2,3-triazol-4-yl)methyl)-7-bromo-5-chloro-2-phenyl-1*H*-indole **5a** S3

2.2 General procedure for the preparation of functionalized 2-(2-bromophenyl)-1-((1-substituted-1*H*-1,2,3-triazol-4-yl)methyl)-1*H*-indole **6** S4

2.2.a Typical one-pot four-step procedure for the preparation of methyl 2-(2-bromophenyl)-1-((1-(4-methoxyphenyl)-1*H*-1,2,3-triazol-4-yl)methyl)-1*H*-indole-5-carboxylate **6q (path a)** S5

2.2.b Typical one-pot four-step procedure for the preparation of 2-(2-bromophenyl)-1-((1-(4-chlorophenyl)-1*H*-1,2,3-triazol-4-yl)methyl)-1*H*-indole **6a (path b)** S5

2.3. General procedure for the preparation of azides S6

2.3.1 Typical procedure for the preparation of 5-azido-1,2,3-trimethoxybenzene S6

3. CHARACTERIZATION DATA OF STARTING MATERIALS... S7

3.1 Characterization data of **4b-g**.. S7

3.2 Characterization data of **5b-k**.. S9

3.3 Characterization data of **6b-p**.. S12

4. REFERENCES.. S19

5. ^{1}H,^{13}C, ^{13}C DEPT, ^{19}F NMR Spectra S20

1. GENERAL INFORMATION

1.1. Reagents and methods

All the commercially available reagents, catalysts, bases, and solvents were used as purchased, without further purification. Starting materials and reaction products were purified by flash chromatography using SiO_2 as stationary phase, eluting with *n*-hexane/ethyl acetate. ^{1}H NMR (400.13 MHz), ^{13}C NMR (100.6 MHz), and ^{19}F spectra (376.5 MHz) were recorded with a Bruker Avance 400 spectrometer equipped with a Nanobay console and Cryoprobe Prodigy probe. Splitting patterns are designed as s (singlet), d (doublet), t. (triplet), q (quartet), m (multiplet), or bs (broad singlet). IR spectra were recorded with a Jasco FT/IR-430 spectrometer. HRMS were recorded with an Orbitrap Exactive Mass spectrometer with ESI source. Melting points were determined with a Büchi B-545 apparatus and are uncorrected. All the azides are commercially available except for 5-azido-1,2,3-trimethoxybenzene, which was synthesized according to the reported procedure.

2. SYNTHETIC PROCEDURES FOR STARTING MATERIALS

2.1 General procedure for the preparation of 7-bromo-1-((1-substituted-1*H*-1,2,3-triazol-4-yl)methyl)-2-phenyl-1*H*-indole 5

Starting materials **5** were prepared following a one-pot four-step sequence based on a slightly modified literature procedure [39] (Scheme S1).

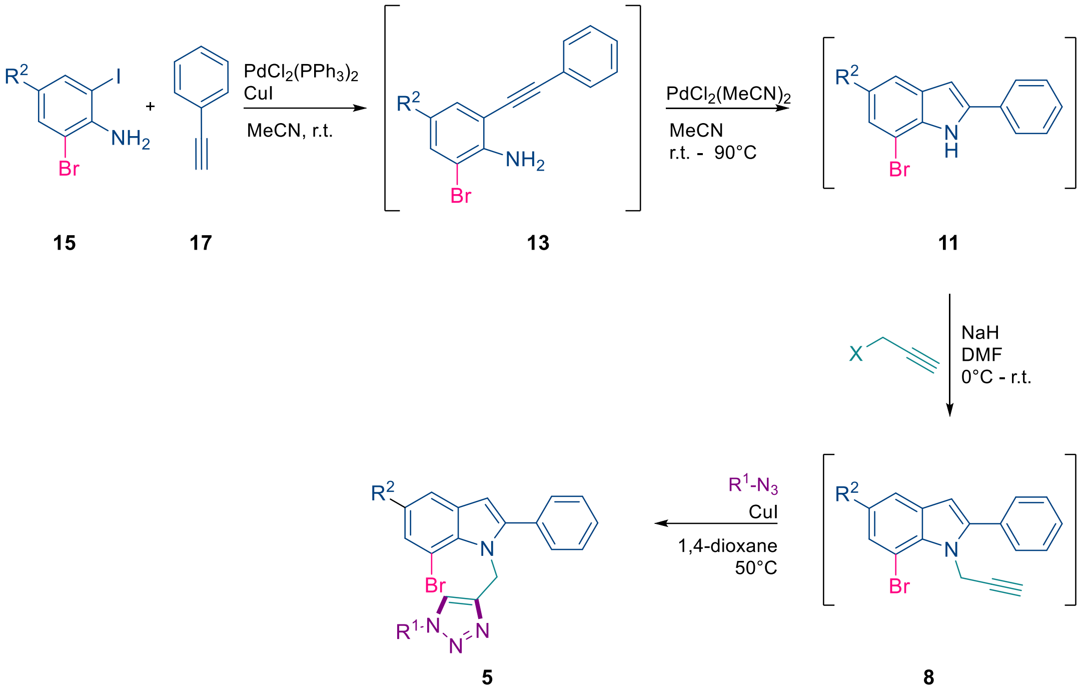

Scheme S1. Preparation of starting material **5**

<u>*2.1.1 Typical one-pot four-step procedure for the preparation of 7-bromo-1-((1-substituted-1H-1,2,3-triazol-4-yl)methyl)-2-phenyl-1H-indole: synthesis of 1-((1-benzyl-1H-1,2,3-triazol-4-yl)methyl)-7-bromo-5-chloro-2-phenyl-1H-indole* ***5a***</u>

In a 50 mL Carousel Tube Reactor (Radely Discovery Technology) containing a magnetic stirring bar, CuI (3.0 mg, 0.01 mmol) and $PdCl_2(PPh_3)_2$ (14.0 mg, 0.02 mmol) were dissolved at room temperature with 1.0 mL of anhydrous CH_3CN under argon. Then, 2-bromo-6-iodo-4-chloroaniline **15** (332.4 mg, 1.0 mmol), phenylacetylene **17** (131.6 µl, 1.2 mmol), and 1.0 mL of solvent were added. The reaction mixture was stirred for 12 h at room temperature. After this time, $PdCl_2(CH_3CN)_2$ (13.0 mg, 0.05 mmol) was added, and the reaction mixture was stirred for 12 h at 90°C. After cooling, the reaction mixture was evaporated under reduced pressure. Then, the residue containing **11a** was diluted with 2.0 mL of anhydrous DMF and added dropwise to a DMF suspension of NaH (60% dispersion in mineral oil, previously washed with *n*-hexane three times, 48.0 mg, 1.2 mmol) at 0°C. Then, propargyl bromide (solution 80 wt% in toluene) (162 µL, 1.5 mmol) was added, and the solution was warmed to room temperature and stirred for 1 h. After this, the reaction mixture was diluted with Et_2O and washed with a saturated solution of $NaHCO_3$ and brine. The organic layer was dried over Na_2SO_4, filtered, and concentrated under reduced pressure. The crude product 7-bromo-5-chloro-2-phenyl-1-(prop-2-yn-1-yl)-1*H*-indole **8a** was dissolved in 2.0 mL of 1,4-dioxane, and CuI (38.1 mg, 0.20 mmol) and benzyl azide (146.3 mg, 1.1 mmol) were added. The reaction mixture was stirred at 50°C for 12 hours. Upon completion, the mixture was concentrated under reduced pressure, and the residue was purified by flash chromatography (*n*-hexane/EtOAc, 70/30 v/v) to obtain 1-((1-benzyl-1*H*-1,2,3-triazol-4-yl)methyl)-7-bromo-5-chloro-2-phenyl-1*H*-indole **5**a (291.5 mg, 61 % yield).

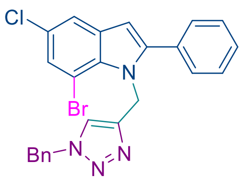

1-((1-benzyl-1*H*-1,2,3-triazol-4-yl)methyl)-7-bromo-5-chloro-2-phenyl-1*H*-indole 5a: 61% yield; orange solid; mp: 230 – 232 °C; IR (neat): 3071, 1737, 1358, 1130, 690 cm^{-1}; ^{1}H NMR (400.13 MHz) ($CDCl_3$): δ 7.54 (d, *J* = 2.0 Hz, 1H), 7.48 – 7.30 (m, 9H), 7.16 – 7.07 (m, 2H), 6.86 (s, 1H), 6.51 (s, 1H), 5.84 (s, 2H), 5.43 (s, 2H); ^{13}C NMR (100.6 MHz) ($CDCl_3$): δ 146.9 (C), 145.5 (C), 134.8 (C), 132.7 (C), 132.3 (C), 131.5 (C), 129.6 (CH), 129.0 (CH), 128.9 (CH), 128.7 (CH), 128.6 (CH), 127.5 (CH), 127.0 (CH), 126.2 (C), 121.5 (CH), 119.4 (CH), 104.3 (C), 103.8 (CH), 54.0 (CH_2), 41.7 (CH_2); HRMS: m/z $[M + H]^+$ calcd for $C_{24}H_{19}BrClN_4$: 477.0476; found: 477.0482.

2.2 General procedure for the preparation of functionalized 2-(2-bromophenyl)-1-((1-substituted-1*H*-1,2,3-triazol-4-yl)methyl)-1*H*-indole 6

Starting materials **6** were prepared following the one-pot four-step sequence depicted in Scheme S2. Depending on the commercial availability of substituted 2-ethynylanilines **20**, 1-bromo-2-ethynylbenzenes **18**, and 1-bromo-2-iodobenzenes **19**, the Sonogashira cross-coupling / Utimoto cyclization could be carried out according to ***path a*** or ***path b***.

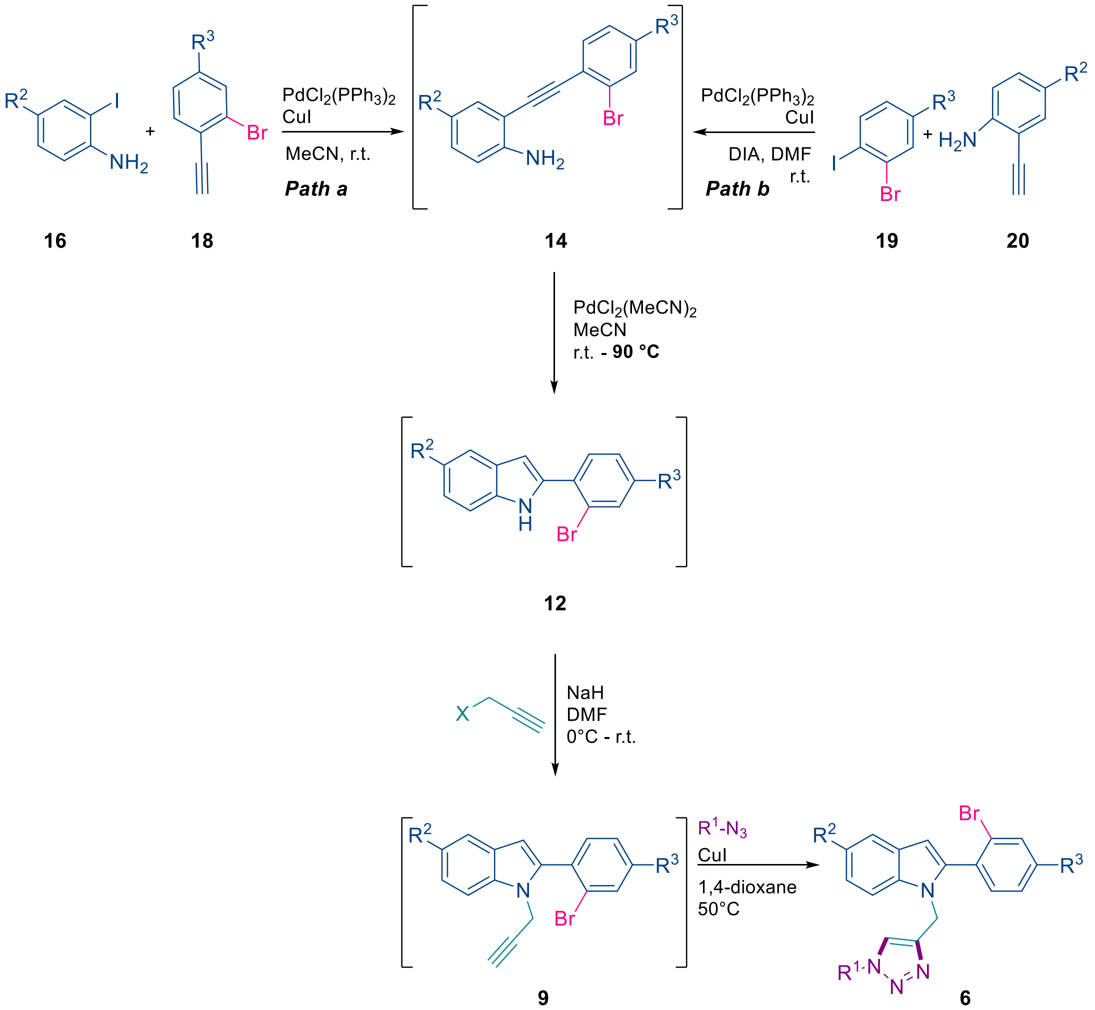

Scheme S2. Preparation of starting materials **6**

*2.2.a Typical one-pot four-step procedure for the preparation of methyl 2-(2-bromophenyl)-1-((1-(4-methoxyphenyl)-1H-1,2,3-triazol-4-yl)methyl)-1H-indole-5-carboxylate **6q** **(path a)***

In a 50 mL Carousel Tube Reactor (Radely Discovery Technology) containing a magnetic stirring bar, CuI (3.0 mg, 0.01 mmol) and $PdCl_2(PPh_3)_2$ (14.0 mg, 0.02 mmol) were dissolved at room temperature with 1.0 mL of anhydrous CH_3CN under argon. Then, methyl 4-amino-3-iodobenzoate **15** (277.1 mg, 1.0 mmol), 2-bromophenylacetylene **18** (131.6 µl, 1.2 mmol), and 1.0 mL of solvent were added. The reaction mixture was stirred for 4 h at room temperature. After this time, $PdCl_2(CH_3CN)_2$ (13.0 mg, 0.05 mmol) was added, and the reaction mixture was stirred for 12 h at 90 °C. After cooling, the reaction mixture was evaporated under reduced pressure. Then, the residue containing **12q** was diluted with 2.0 mL of anhydrous DMF and added dropwise to a DMF suspension of NaH (60% dispersion in mineral oil, previously washed with *n*-hexane three times, 48.0 mg, 1.2 mmol) at 0°C. Then, propargyl bromide (solution 80 wt% in toluene) (162 µL, 1.5 mmol) was added, and the solution was warmed to room temperature and stirred for 1 h. After this time, the reaction mixture was diluted with Et_2O and washed with a saturated solution of $NaHCO_3$ and brine. The organic layer was dried over Na_2SO_4, filtered, and concentrated under reduced pressure. The crude product methyl 2-(2-bromophenyl)-1-(prop-2-yn-1-yl)-1*H*-indole-5-carboxylate **9q** was dissolved in 2.0 mL of 1,4-dioxane, and CuI (38.1 mg, 0.200 mmol) and 1-azido-4-methoxybenzene (164.1 mg, 1.1 mmol) were added. The reaction mixture was stirred at 50°C for 12 hours. Upon completion, the mixture was concentrated under reduced pressure, and the residue was purified by flash chromatography (*n*-hexane/EtOAc, 70/30 v/v) to obtain methyl 2-(2-bromophenyl)-1-((1-(4-methoxyphenyl)-1*H*-1,2,3-triazol-4-yl)methyl)-1*H*-indole-5-carboxylate **6q** (296.8 mg, 57 % yield).

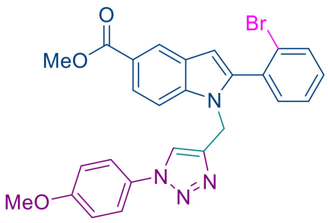

methyl 2-(2-bromophenyl)-1-((1-(4-methoxyphenyl)-1*H*-1,2,3-triazol-4-yl)methyl)-1*H*-indole-5-carboxylate 6q: 57% yield; yellow solid; mp: 200 – 202 °C; IR (neat): 1607, 1456, 1226, 1022, 793 cm^{-1}; ^1H NMR (400.13 MHz) ($CDCl_3$): δ 8.36 (d, *J* = 1.36 Hz, 1H), 7.87 (dd, J_1 = 8.7 Hz, J_2 = 1.5 Hz, 1H), 7.65 (d, *J* = 7.8 Hz, 1H), 7.42 (d, *J* = 8.9 Hz, 1H), 7.40 – 7.36 (m, 2H), 7.34 – 7.31 (m, 2H), 7.28 – 7.24 (m, 1H), 7.22 (s, 1H), 6.88 – 6.84 (m, 2H), 6.60 (s, 1H), 5.32 (s, 2H), 3.86 (s, 3H), 3.74 (s, 3H); ^{13}C NMR (100.6 MHz) ($CDCl_3$): δ 168.1 (C) ,159.9 (C), 144.8 (C), 140.3 (C), 139.1 (C), 133.3 (CH), 133.1 (C), 132.9 (CH), 130.9 (CH), 130.3 (C), 127.7 (CH), 127.6 (C), 125.1 (C), 124.0 (CH), 123.8 (CH), 122.6 (C), 122.2 (CH), 120.1 (CH), 114.8 (CH), 110.1 (CH), 105.2 (CH), 55.7 (CH_3), 52.0 (CH_3), 40.3 (CH_2). HRMS: m/z $[M + H]^+$ calcd for $C_{26}H_{22}BrN_4O_3$: 517.0870; found: 517.0879.

*2.2.b Typical one-pot four-step procedure for the preparation of 2-(2-bromophenyl)-1-((1-(4-chlorophenyl)-1H-1,2,3-triazol-4-yl)methyl)-1H-indole **6a** **(path b)***

In a 50 mL Carousel Tube Reactor (Radely Discovery Technology) containing a magnetic stirring bar, CuI (3.0 mg, 0.01 mmol) and $PdCl_2(PPh_3)_2$ (14.0 mg, 0.02 mmol) were dissolved at room temperature with 1.0 mL of anhydrous DMF under argon. Then, 1-bromo-2-iodobenzene **19** (283.0 mg, 1.0 mmol), 2-ethynylaniline **20a** (136.5 µl, 1.2 mmol), and 2.0 mL of DIA were added, and the resulting solution was stirred for 1 h at room temperature. After this, the reaction mixture was diluted with Et_2O and washed with a saturated solution of $NaHCO_3$ and brine. The organic layer was dried over Na_2SO_4, filtered, and concentrated under reduced

pressure. Then, the crude product **14a** was dissolved in 3.0 mL of MeCN, $PdCl_2(CH_3CN)_2$ (13.0 mg, 0.05 mmol) was added, and the solution was stirred overnight at 90 °C. After completion, the reaction mixture was evaporated under reduced pressure. Then, the residue containing **12a** was diluted with 2.0 mL of anhydrous DMF and added dropwise to a DMF suspension of NaH (60% dispersion in mineral oil, previously washed with *n*-hexane three times, 48.0 mg, 1.2 mmol) at 0 °C. Then, propargyl bromide (solution 80 wt% in toluene) (162 μL, 1.5 mmol) was added, and the solution was warmed to room temperature and stirred for 1 h. After this, the reaction mixture was diluted with Et_2O and washed with a saturated solution of $NaHCO_3$ and brine. The organic layer was dried over Na_2SO_4, filtered, and concentrated under reduced pressure. The crude product 2-(2-bromophenyl)-1-(prop-2-yn-1-yl)-1*H*-indole **9a** was dissolved in 2.0 mL of 1,4-dioxane, and CuI (38.1 mg, 0.200 mmol) and 1-azido-4-chlorobenzene (169.0 mg, 1.1 mmol) were added. The reaction mixture was stirred at 50 °C for 12 hours. Upon completion, the mixture was concentrated under reduced pressure, and the residue was purified by flash chromatography (*n*-hexane/EtOAc, 70/30 v/v) to obtain 2-(2-bromophenyl)-1-((1-(4-chlorophenyl)-1*H*-1,2,3-triazol-4-yl)methyl)-1*H*-indole **6a** (292.1 mg, 63 % yield).

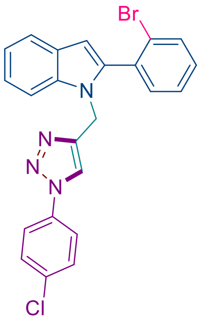

2-(2-bromophenyl)-1-((1-(4-chlorophenyl)-1*H*-1,2,3-triazol-4-yl)methyl)-1*H*-indole 6a: 63% yield; yellow solid; mp: 78 – 80 °C; IR (neat): 1499, 1455, 1041, 828, 749 cm^{-1}; ^1H NMR (400.13 MHz) ($CDCl_3$): δ 7.63 (d, *J* = 8.1 Hz, 1H), 7.59 (d, *J* = 7.8 Hz, 1H), 7.44 – 7.39 (m, 2H), 7.37 – 7.31 (m, 5H), 7.28 (s, 1H), 7.25 – 7.21 (m, 1H), 7.18 – 7.12 (m, 1H), 7.11 – 7.05 (m, 1H), 6.53 (s,1H), 5.31 (s, 2H); ^{13}C NMR (100.6 MHz) ($CDCl_3$): δ 145.9 (C), 138.8 (C), 136.6 (C), 135.4 (C), 134.6 (C), 133.60 (C), 133.3 (CH), 132.9 (CH), 130.63 (CH), 129.9 (CH), 128.2 (C), 127.5 (CH), 125.1 (C), 122.5 (CH), 121.6 (CH), 121.1 (CH), 120.5 (CH), 119.9 (CH), 110.3 (CH), 104.1 (CH), 40.06 (CH_2). HRMS: m/z $[M + H]^+$ calcd for $C_{23}H_{17}BrClN_4$: 463.0320; found: 463.0312.

2.3. General procedure for the preparation of azides

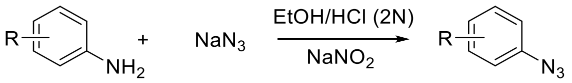

Scheme S3

<u>*2.3.1 Typical procedure for the preparation of 5-azido-1,2,3-trimethoxybenzene*</u>

Following the same procedure reported in literature [43], the 3,4,5-trimethoxyaniline (1.832 g, 10.0 mmol, 1.0 equiv.) was suspended in 17% hydrochloric acid at room temperature, and ethanol was added until a clear solution was obtained. The solution was then cooled to 0°C, and $NaNO_2$ (1.035 g, 15.0 mmol, 1.5 equiv.) was added in small portions. After stirring at 0°C for 15-30 minutes, NaN_3 (0.975 g, 15.0 mmol, 1.5 equiv.) was added slowly, and the mixture was stirred for an additional 2 hours at room temperature. The reaction mixture was extracted with diethyl ether, and the combined organic fractions were washed with saturated $NaHCO_3$

solution and brine, dried over Na_2SO_2, and concentrated under reduced pressure. The desired azide was obtained in quantitative yield without further purification.

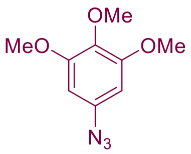

5-azido-1,2,3-trimethoxybenzene: 99% yield, 2.08 g, 9.9 mmol; brown solid; lit. mp [44]: 42 – 43 °C; mp: 45 – 47 °C; R_f = 0.20 (*n*-hexane-EtOAc, 75:25); IR (neat): 2910, 2485, 1461, 1135, 993 cm^{-1}; ^{1}H NMR (400.13 MHz, $CDCl_3$) δ 6.18 (s, 2H), 3.78 (s, 6H), 3.74 (s, 3H); ^{13}C NMR (100.6 MHz, $CDCl_3$) δ 154.1 (C), 135.7 (C), 135.4 (C), 96.4 (CH), 61.0 (CH_3), 56.2 (CH_3). HRMS: m/z [M + H]$^+$ calcd for $C_9H_{12}N_3O_3$:210.0873; found: 210.0888.

3. CHARACTERIZATION DATA OF STARTING MATERIALS

3.1 Characterization data of 4b-g

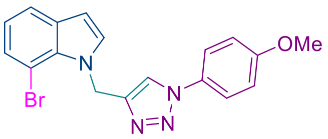

7-bromo-1-((1-(4-methoxyphenyl)-1*H*-1,2,3-triazol-4-yl)methyl)-1*H*-indole 4b: 67% yield; brown solid; mp: 214 – 216 °C; IR (neat): 3180, 2945, 1364, 912, 719 cm^{-1}; ^{1}H NMR (400.13 MHz) ($CDCl_3$): δ 7.65 (s, 1H), 7.59 (d, *J* = 7.7 Hz, 1H), 7.53 (d, *J* = 8.8 Hz, 2H), 7.39 (d, *J* = 7.7 Hz, 1H), 7.32 (d, *J* = 3.2 Hz, 1H), 7.02 – 6.91 (m, 3H), 6.57 (d, *J* = 3.2 Hz, 1H), 6.01 (s, 2H), 3.84 (s, 3H); ^{13}C NMR (100.6 MHz) ($CDCl_3$): δ 159.8 (C), 146.2 (C), 132.14 (C), 132.06 (C), 131.1 (CH), 130.3 (C), 127.2 (CH), 122.2 (CH), 121.0 (CH), 120.7 (CH), 120.4 (CH), 114.7 (CH), 103.4 (C), 103.0 (CH), 55.6 (CH_3), 43.6 (CH_2); HRMS: m/z [M + H]$^+$ calcd for $C_{18}H_{16}BrN_4O$: 383.0502; found: 383.0514.

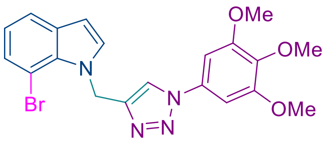

7-bromo-1-((1-(3,4,5-trimethoxyphenyl)-1H-1,2,3-triazol-4-yl)methyl)-1*H*-indole 4c: 78% yield; brown solid; mp: 222 – 224 °C; IR (neat): 3101, 3015, 1368, 1074, 750 cm^{-1}; ^{1}H NMR (400.13 MHz) ($CDCl_3$): δ 7.59 (s, 1H), 7.50 (d, *J* = 7.8 Hz, 1H), 7.30 (d, *J* = 7.8 Hz, 1H), 7.22 (d, *J* = 3.3 Hz, 1H), 6.88 (t, *J* = 7.7 Hz, 1H), 6.76 (s, 2H), 6.49 (d, *J* = 3.3 Hz, 1H), 5.93 (s, 2H), 3.80 (s, 6H), 3.78 (s, 3H); ^{13}C NMR (100.6 MHz) ($CDCl_3$): δ 153.9 (C), 146.4 (C), 138.4 (C), 132.8 (C), 132.09 (C), 132.06 (C), 131.0 (CH), 127.3 (CH), 121.0 (CH), 120.7 (CH), 120.6 (CH), 103.5 (C), 103.0 (CH), 98.7 (CH), 61.0 (CH_3), 56.5 (CH_3), 43.5 (CH_2); HRMS: m/z [M + H]+ calcd for $C_{20}H_{20}BrN_4O_3$: 443.0713; found: 443.0720.

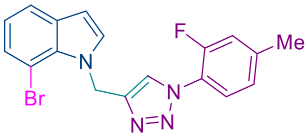

7-bromo-1-((1-(2-fluoro-4-methylphenyl)-1*H*-1,2,3-triazol-4-yl)methyl)-1*H*-indole 4d: 67% yield; yellow solid; mp: 228 – 230 °C; IR (neat): 3099, 1502, 1297, 947, 674 cm^{-1}; ^1H NMR (400.13 MHz) ($CDCl_3$): δ 7.71 (d, *J* = 2.6 Hz, 1H), 7.62 (t, *J* = 7.8 Hz, 1H), 7.47 (d, *J* = 7.8 Hz, 1H), 7.28 (d, *J* = 7.6 Hz, 1H), 7.23 (d, *J* = 3.3 Hz, 1H), 6.96 (t, *J* = 8.1 Hz, 2H), 6.86 (t, *J* = 7.7 Hz, 1H), 6.47 (d, *J* = 3.3 Hz, 1H), 5.92 (s, 2H), 2.30 (s, 3H); ^{13}C NMR (100.6 MHz) ($CDCl_3$): δ 154.4 (C), 151.9 (C), 145.8 (C), 141.3 (d, J_{C-F} = 7.3 Hz, C), 132.1 (d, J_{C-F} = 4.9 Hz, C), 131.1 (CH), 127.2 (CH), 125.8 (d, J_{C-F} = 3.3 Hz, CH), 124.6 (CH), 123.3 (d, J_{C-F} = 8.3 Hz, CH), 122.7 (d, J_{C-F} = 10.6 Hz, C), 121.0 (CH), 120.7 (CH), 117.2 (d, J_{C-F} = 20.6 Hz, CH), 103.5 (C), 103.0 (CH), 43.4 (CH_2), 21.2 (CH_3); ^{19}F NMR (376.5 MHz) ($CDCl_3$): δ -124.41 (m); HRMS: m/z $[M + H]^+$ calcd for $C_{18}H_{15}BrFN_4$: 385.0457; found: 385.0444.

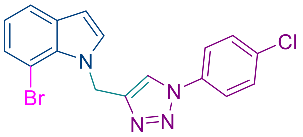

7-bromo-1-((1-(4-chlorophenyl)-1*H*-1,2,3-triazol-4-yl)methyl)-1*H*-indole 4e: 69% yield; white solid; mp: 190 – 192 °C; IR (neat): 3071, 1498, 1322, 998, 724 cm^{-1}; ^1H NMR (400.13 MHz) ($CDCl_3$): δ 7.58 (s, 1H), 7.51 – 7.47 (m, 3H), 7.35 – 7.32 (m, 2H), 7.28 (dd, J_1 = 7.6 Hz, J_2 = 1.1 Hz, 1H), 7.20 (d, *J* = 3.4 Hz, 1H), 6.87 (t, *J* = 7.6 Hz, 1H), 6.47 (d, *J* = 3.4 Hz, 1H), 5.91 (s, 2H); ^{13}C NMR (100.6 MHz) ($CDCl_3$): δ 146.8 (C), 135.4 (C), 134.6 (C), 132.1 (C), 132.0 (C), 131.0 (CH), 129.9 (CH), 127.3 (CH), 121.7 (CH), 121.1 (CH), 120.8 (CH), 120.1 (CH), 103.4 (C), 103.1 (CH), 43.5 (CH_2); HRMS: m/z $[M + H]^+$ calcd for $C_{17}H_{13}BrClN_4$: 387.0007; found: 387.0019.

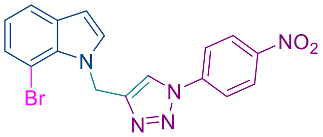

7-bromo-1-((1-(4-nitrophenyl)-1*H*-1,2,3-triazol-4-yl)methyl)-1*H*-indole 4f: 92% yield; white solid; mp: 200 – 202 °C; IR (neat): 3012, 1519, 1095, 784 cm^{-1}; ^1H NMR (400.13 MHz) ($CDCl_3$): δ 8.33 (d, *J* = 9.1 Hz, 2H), 7.86 7.77 (s, 1H), 7.57 (d, *J* = 7.6 Hz, 1H), 7.37 (d, *J* = 7.5 Hz, 1H), 7.28 (d, *J* = 3.2 Hz, 1H), 7.20 (d, *J* = 3.4 Hz, 1H), 6.96 (t, *J* = 7.6 Hz, 1H), 6.57 (d, *J* = 3.2 Hz, 1H), 6.01 (s, 2H); ^{13}C NMR (100.6 MHz) ($CDCl_3$): δ 147.8 (C), 147.5 (C), 141.2 (C), 132.4 (C), 132.2 (C), 131.3 (CH), 127.6 (CH), 125.7 (CH), 121.5 (CH), 121.1 (CH), 120.8 (CH), 120.3 (CH), 103.66 (C), 103.60 (CH), 43.7 (CH_2); HRMS: m/z $[M + H]^+$ calcd for $C_{17}H_{13}BrN_5O_2$: 410.0247; found: 410.0255.

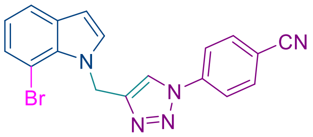

4-(4-((7-bromo-1*H*-indol-1-yl)methyl)-1*H*-1,2,3-triazol-1-yl)benzonitrile 4g: 69% yield; white solid; mp: 195 – 197 °C; IR (neat): 3015, 1465, 1335, 988, 767 cm^{-1}; ^1H NMR (400.13 MHz) ($CDCl_3$): δ 7.73 – 7.67 (m, 5H), 7.49 (d, *J* = 7.7 Hz, 1H), 7.28 (d, *J* = 7.7 Hz, 1H), 7.19 (d, *J* = 3.2 Hz, 1H), 6.88 (t, *J* = 7.7 Hz, 1H), 6.48 (d, *J* = 3.4 Hz, 1H), 5.92 (s, 2H); ^{13}C NMR (100.6 MHz) ($CDCl_3$): δ 147.6 (C), 139.7 (C), 133.9 (CH), 132.3 (C), 132.0 (C), 131.1 (CH), 127.4 (CH), 121.3 (CH), 120.9 (CH), 120.7 (CH), 120.0 (CH), 117.8 (C), 112.5 (C), 103.4 (C), 103.3 (CH), 43.5 (CH_2); HRMS: m/z $[M + H]^+$ calcd for $C_{18}H_{13}BrN_5$: 378.0349; found: 378.0336.

3.2 Characterization data of 5b-k

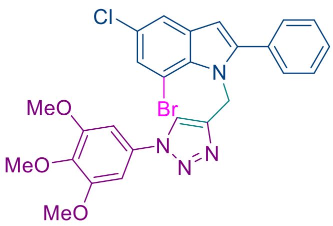

7-bromo-5-chloro-2-phenyl-1-((1-(3,4,5-trimethoxyphenyl)-1*H*-1,2,3-triazol-4-yl)methyl)-1*H*-indole 5b: 64% yield; brown solid; mp: 232 – 240 °C; IR (neat): 3078, 1505, 1313, 1110, 770 cm^{-1}; ^{1}H NMR (400.13 MHz) ($CDCl_3$): δ 7.65 (d, *J* = 8.3 Hz, 1H), 7.54 (d, *J* = 2.0 Hz, 1H), 7.38 – 7.19 (m, 5H), 7.10 (dd, J_1 = 8.7 Hz, J_2 = 2.0 Hz, 1H), 6.70 (s, 2H), 6.47 (s, 1H), 5.28 (s, 2H), 3.78 (s, 6H), 3.77 (s, 3H); ^{13}C NMR (100.6 MHz) ($CDCl_3$): δ 153.8 (C), 145.0 (C), 139.9 (C), 138.4 (C), 134.9 (C), 133.3 (CH), 133.1 (C), 132.7 (CH), 132.6 (C), 130.8 (CH), 129.0 (C), 127.5 (CH), 126.1 (C), 124.9 (C), 122.6 (CH), 120.3 (CH), 120.2 (CH), 111.4 (CH), 103.6 (CH), 98.5 (CH), 61.0 (CH_3), 56.5 (CH_3), 40.1 (CH_2); HRMS: m/z [M + H]$^+$ calcd for $C_{26}H_{23}BrClN_4O_3$: 553.0637; found: 553.0621.

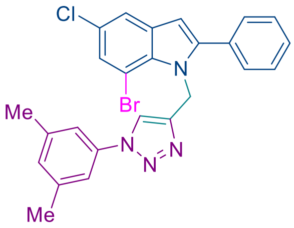

7-bromo-5-chloro-1-((1-(3,5-dimethylphenyl)-1*H*-1,2,3-triazol-4-yl)methyl)-2-phenyl-1*H*-indole 5c: 75% yield; white solid; mp: 226 – 228 °C; IR (neat): 3211, 1417, 1314, 1101, 739 cm^{-1}; ^{1}H NMR (400.13 MHz) ($CDCl_3$): δ 7.48 (d, *J* = 2.0 Hz, 1H), 7.36 (s, 5H), 7.30 (d, *J* = 2.0 Hz, 2H), 7.24 (s, 1H), 7.13 (s, 2H), 6.93 (s, 1H), 6.48 (s, 1H), 5.83 (s, 2H), 2.27 (s, 6H); ^{13}C NMR (100.6 MHz) ($CDCl_3$): δ 147.0 (C), 145.4 (C), 139.7 (C), 136.7 (C), 132.7 (C), 132.3 (C), 131.5 (C), 130.4 (CH), 129.7 (CH), 129.0 (CH), 128.8 (CH), 127.1 (CH), 126.3 (C), 119.6 (CH), 119.5 (CH), 118.2 (CH), 104.3 (C), 103.9 (CH), 41.7 (CH_2), 21.3 (CH_3); HRMS: m/z [M + H]$^+$ calcd for $C_{25}H_{21}BrClN_4$: 491.0633; found: 491.0641.

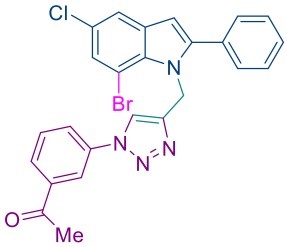

1-(3-(4-((7-bromo-5-chloro-2-phenyl-1*H*-indol-1-yl)methyl)-1*H*-1,2,3-triazol-1-yl)phenyl)ethan-1-one 5d: 68% yield; yellow solid; mp: 240 – 242 °C; IR (neat): 3176, 3044, 1740, 1022, 775 cm^{-1}; ^{1}H NMR (400.13 MHz) ($CDCl_3$): δ 8.16 (s, 1H), 7.98 (d, *J* = 7.8 Hz, 1H), 7.90 (d, *J* = 7.8 Hz, 1H), 7.64 – 7.56 (m, 2H), 7.53 – 7.46 (m, 6H), 7.39 (s, 1H), 6.60 (s, 1H), 5.96 (s, 2H), 2.66 (s, 3H); ^{13}C NMR (100.6 MHz) ($CDCl_3$): δ 196.7 (C), 147.4 (C), 145.4 (C), 138.4 (C), 137.3 (C), 132.7 (C), 132.4 (C), 131.5 (C), 130.2 (CH), 129.7 (CH), 129.1 (CH), 128.8 (CH), 128.4 (CH), 127.2 (CH), 126.4 (C), 124.7 (CH), 119.6 (CH), 119.5 (CH), 119.4 (CH), 104.3 (C), 104.1 (CH), 41.5 (CH_3), 26.8 (CH_2); HRMS: m/z [M + H]$^+$ calcd for $C_{25}H_{19}BrClN_4O$: 505.0425; found: 505.0411

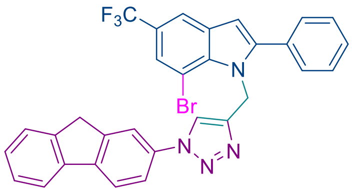

1-((1-(9*H*-fluoren-2-yl)-1*H*-1,2,3-triazol-4-yl)methyl)-7-bromo-2-phenyl-5-(trifluoromethyl)-1*H*-indole 5e: 55% yield; brown solid; mp: 258 – 260 °C; IR (neat): 3088, 1494, 1350, 1134, 799 cm^{-1}; ^1H NMR (400.13 MHz) ($CDCl_3$): δ 7.92 (s, 1H), 7.83 (t, *J* = 9.3 Hz, 3H), 7.66 (s, 1H), 7.60 (t, *J* = 10.5 Hz, 2H), 7.50 (s, 5H), 7.46 (s, 1H), 7.44 – 7.34 (m, 2H), 6.74 (s, 1H), 6.02 (s, 2H), 3.97 (s, 2H); ^{13}C NMR (100.6 MHz) ($CDCl_3$): δ 145.7 (C), 144.7 (C), 143.5 (C), 142.4 (C), 140.3 (C), 135.4 (C), 135.2 (C), 131.3 (C), 131.1 (C), 129.8 (CH), 129.2 (CH), 128.9 (CH), 127.5 (CH), 127.1 (CH), 125.2 (CH), 124.2 (q, J_{C-F} = 4.2 Hz, CH), 124.1 (q, J_{C-F} = 271.8 Hz, C), 124.0 (q, J_{C-F} = 21.4 Hz, C), 120.6 (CH), 120.3 (CH), 119.4 (CH), 117.8 (q, J_{C-F} = 4.2 Hz, CH), 117.5 (CH), 105.0 (CH), 104.2 (C), 41.7 (CH_2), 37.0 (CH_2); ^{19}F NMR (376.5 MHz) ($CDCl_3$): δ -60.77 (s); HRMS: m/z $[M + H]^+$ calcd for $C_{31}H_{21}BrF_3N_4$: 585.0896; found: 585.0883.

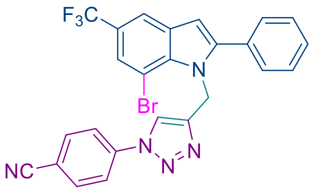

4-(4-((7-bromo-2-phenyl-5-(trifluoromethyl)-1*H*-indol-1-yl)methyl)-1*H*-1,2,3-triazol-1-yl)benzonitrile 5f: 49% yield; yellow solid; mp: 214 – 216 °C; IR (neat): 3082, 1500, 1313, 1111, 778 cm^{-1}; ^1H NMR (400.13 MHz) ($CDCl_3$): δ 7.68 (s, 1H), 7.42 (s, 1H), 7.36 (d, *J* = 8.6 Hz, 2H), 7.30 – 7.19 (m, 7H), 7.16 (s, 1H), 6.51 (s, 1H), 5.79 (s, 2H); ^{13}C NMR (100.6 MHz) ($CDCl_3$): δ 145.7 (C), 135.4 (C), 135.14 (C), 135.12 (C), 134.6 (C), 131.2 (C), 131.1 (C), 129.9 (CH), 129.8 (CH), 129.8 (CH), 129.2 (CH), 128.9 (CH), 124.3 (q, J_{C-F} = 271.8 Hz, C), 124.2 (q, J_{C-F} = 4.2 Hz, CH), 124.1 (q, J_{C-F} = 21.4 Hz, C), 121.6 (CH), 117.8 (q, J_{C-F} = 4.2 Hz, CH), 105.1 (CH), 104.2 (C), 41.5 (CH_2); ^{19}F NMR (376.5 MHz) ($CDCl_3$): δ -60.71 (s); HRMS: m/z $[M + H]^+$ calcd for $C_{25}H_{16}BrF_3N_5$: 522.0536; found: 522.0549.

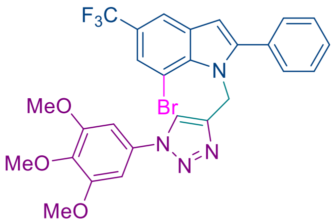

7-bromo-2-phenyl-5-(trifluoromethyl)-1-((1-(3,4,5-trimethoxyphenyl)-1*H*-1,2,3-triazol-4-yl)methyl)-1*H*-indole 5g: 78% yield; orange solid; mp: 228 – 230 °C; IR (neat): 2941, 1601, 1367, 1096, 753 cm^{-1}; ^1H NMR (400.13 MHz) ($CDCl_3$): δ 7.91 (s, 1H), 7.65 (s, 1H), 7.49 (s, 5H), 7.40 (s, 1H), 6.83 (s, 2H), 6.74 (s, 1H), 5.99 (s, 2H), 3.91 (s, 6H), 3.87 (s, 3H); ^{13}C NMR (100.6 MHz) ($CDCl_3$): δ 153.9 (C), 146.8 (C), 145.8 (C), 138.5 (C), 135.2 (C), 132.7 (C), 131.3 (C), 131.1 (C), 129.8 (CH), 129.2 (CH), 128.9 (CH), 124.2 (q, J_{C-F} = 271.8 Hz, C), 124.1 (q, J_{C-F} = 4.2 Hz, CH), 124.0 (q, J_{C-F} = 21.4 Hz, C), 119.8 (CH), 117.7 (q, J_{C-F} = 4.2 Hz, CH), 105.0 (CH), 104.2 (C), 98.5 (CH), 61.1 (CH_3), 56.5

(CH$_3$), 41.6 (CH$_2$); ^{19}F NMR (376.5 MHz) (CDCl$_3$): δ -60.79 (s); HRMS: m/z [M + H]$^+$ calcd for $C_{27}H_{23}BrF_3N_4O_3$: 587.0900; found: 587.0913.

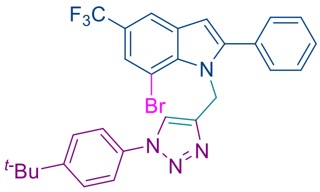

7-bromo-1-((1-(4-(tert-butyl)phenyl)-1*H*-1,2,3-triazol-4-yl)methyl)-2-phenyl-5-(trifluoromethyl)-1*H*-indole 5h: 74% yield; brown solid; mp: 210 – 212 °C; IR (neat): 3156, 2977, 1322, 1219, 667 cm^{-1}; ^{1}H NMR (400.13 MHz) (CDCl$_3$): δ 7.91 (s, 1H), 7.65 (s, 1H), 7.65 (s, 1H), 7.54 (s, 1H), 7.50 – 7.46 (m, 7H), 7.37 (s, 1H), 6.73 (s, 1H), 6.00 (s, 2H), 1.35 (s, 9H); ^{13}C NMR (100.6 MHz) (CDCl$_3$): δ 152.2 (C), 145.7 (C), 135.2 (C), 131.3 (C), 131.1 (C), 129.8 (CH), 129.2 (CH), 128.9 (CH), 126.66 (CH), 126.65 (CH), 124.2 (q, J_{C-F} = 271.8 Hz, C), 124.1 (q, J_{C-F} = 4.2 Hz, CH), 124.0 (q, J_{C-F} = 21.4 Hz, C), 120.3 (CH), 117.7 (q, J_{C-F} = 4.2 Hz, CH), 105.0 (CH), 104.2 (C), 41.4 (CH$_2$), 34.8 (C), 31.2 (CH$_3$); ^{19}F NMR (376.5 MHz) (CDCl$_3$): δ -60.81 (s); HRMS: m/z [M + H]$^+$ calcd for $C_{28}H_{25}BrF_3N_4$: 553.1209; found: 553.1225.

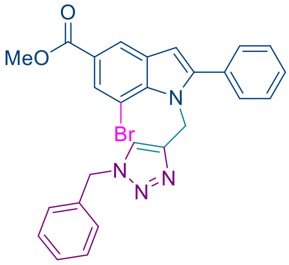

methyl1-((1-benzyl-1*H*-1,2,3-triazol-4-yl)methyl)-7-bromo-2-phenyl-1*H*-indole-5 carboxylate 5i: 75% yield; white solid; mp: 222 – 224 °C; IR (neat): 3104, 1751, 1215, 1014, 708 cm^{-1}; ^{1}H NMR (400.13 MHz) (CDCl$_3$): δ 8.20 (s, 1H), 7.98 (s, 1H), 7.47 – 7.21 (m, 8H), 7.02 – 7.01 (m, 2H), 6.80 (s, 1H), 6.57 (s, 1H), 5.80 (s, 2H), 5.33 (s, 2H), 3.86 (s, 3H); ^{13}C NMR (100.6 MHz) (CDCl$_3$): δ 166.8 (C), 146.8 (C), 145.2 (C), 136.2 (C), 134.7 (C), 131.4 (C), 131.1 (C), 129.7 (CH), 129.0 (CH), 128.67 (CH), 128.62 (CH), 128.5 (CH), 127.56 (C), 127.52 (CH), 123.7 (C), 122.6 (CH), 121.6 (CH), 105.3 (CH), 103.7 (C), 54.0 (CH$_2$), 52.1 (CH$_3$), 41.7 (CH$_2$); HRMS: m/z [M + H]$^+$ calcd for $C_{26}H_{22}BrN_4O_2$: 501.0921; found: 501.0917.

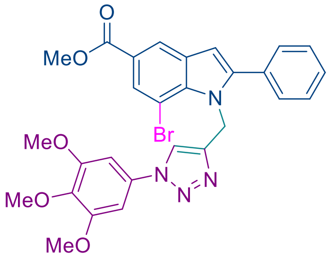

methyl 7-bromo-2-phenyl-1-((1-(3,4,5-trimethoxyphenyl)-1*H*-1,2,3-triazol-4-yl)methyl)-1*H*-indole-5-carboxylate 5j: 69% yield; white solid; mp: 250 – 252 °C; IR (neat): 3091, 2987, 1743, 1075, 965 cm^{-1}; ^{1}H NMR (400.13 MHz)) (CDCl$_3$): δ 8.35 (d, J = 1.5 Hz, 1H), 8.12 (d, J = 1.5 Hz, 1H), 7.49 (bs, 6H), 6.83 – 6.75 (m, 2H), 6.00

(s, 2H), 4.01 – 3.72 (m, 12H).; ^{13}C NMR (100.6 MHz) ($CDCl_3$): δ 166.2 (C), 154.2 (C), 145.7 (C), 137.9 (C), 136.2 (C), 132.7 (C), 131.6 (C), 131.4 (C), 130.0 (CH), 129.5 (CH), 129.2 (CH), 128.9 (C), 127.7 (CH), 123.4 (C), 122.7 (CH), 121.4 (CH), 105.7 (CH), 103.8 (C), 98.5 (CH), 61.0 (CH_3), 56.5 (CH_3), 52.2 (CH_3) 41.7 (CH_2); HRMS: m/z [M + H]$^+$ calcd for $C_{28}H_{26}BrN_4O_5$: 577.1081; found: 577.1096

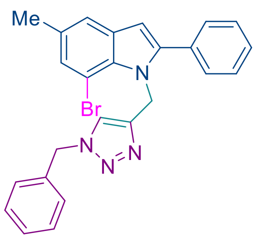

1-((1-benzyl-1*H*-1,2,3-triazol-4-yl)methyl)-7-bromo-5-methyl-2-phenyl-1*H*-indole 5k: 54% yield; white solid; mp: 234 – 236 °C; IR (neat): 3091, 2987, 1743, 1075, 965 cm^{-1}; ^{1}H NMR (400.13 MHz) ($CDCl_3$): δ 7.32 – 7.15 (m, 9H), 7.11 (s, 1H), 7.05 – 6.94 (m, 2H), 6.76 (s, 1H), 6.38 (s, 1H), 5.75 (s, 2H), 5.31 (s, 2H), 2.31 (s, 3H); ^{13}C NMR (100.6 MHz) ($CDCl_3$): δ 147.4 (C), 144.2 (C), 134.9 (C), 132.6 (C), 132.1 (C), 132.0 (C), 131.3 (C), 129.6 (CH), 129.0 (CH), 128.9 (CH), 128.62 (CH), 128.58 (CH), 128.5 (CH), 127.5 (CH), 121.8 (CH), 119.9 (CH), 103.9 (CH), 103.8 (C), 53.9 (CH_2), 41.7 (CH_2), 20.8 (CH_3); HRMS: m/z [M + H]$^+$ calcd for $C_{25}H_{22}BrN_4$: 457.1022; found: 457.1036.

3.3 Characterization data of 6b-p

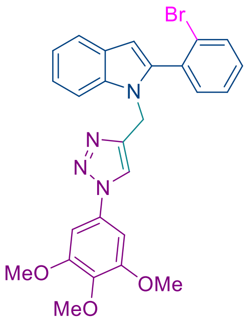

2-(2-bromophenyl)-1-((1-(3,4,5-trimethoxyphenyl)-1*H*-1,2,3-triazol-4-yl)methyl)-1*H*-indole 6b: 78% yield; yellow solid; mp: 147 – 149 °C; IR (neat): 1601, 1454, 1042, 750, 735 cm^{-1}; ^{1}H NMR (400.13 MHz) ($CDCl_3$): δ 7.74 (d, *J* = 7.9 Hz, 1H), 7.71 (d, *J* = 7.9 Hz, 1H), (d, *J* = 8.1 Hz, 1H), 7.46 – 7.31 (m, 3H), 7.37 – 7.32 (m, 1H), 7.30 – 7.24 (m, 1H), 7.20 (t, *J* = 7.4 Hz, 1H), 6.81 (s, 2H), 6.65 (s, 1H), 5.43 (s, 2H), 3.88 (s, 6H), 3.87 (s, 3H); ^{13}C NMR (100.6 MHz) ($CDCl_3$): δ 153.9 (C), 145.5 (C), 138.7 (C), 138.4 (C), 136.6 (C), 133.5 (C), 133.3 (CH), 132.9 (CH), 132.8 (C), 130.6 (CH), 128.1 (C), 127.5 (CH), 125.1 (C), 122.5 (CH), 121.0 (CH), 120.5 (CH), 120.2 (CH), 110.4 (CH), 104.1 (CH), 98.5 (CH), 61.1 (CH_3), 56.5 (overlapping, CH_3), 40.0 (CH_2). HRMS: m/z [M + H]$^+$ calcd for $C_{26}H_{24}BrN_4O_3$: 519.1026; found: 519.1011.

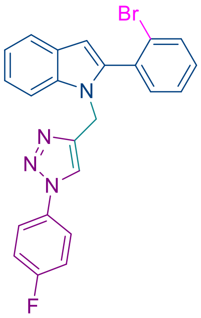

2-(2-bromophenyl)-1-((1-(4-fluorophenyl)-1*H*-1,2,3-triazol-4-yl)methyl)-1*H*-indole 6c: 79% yield; orange solid; mp: 136 – 138 °C; IR (neat): 1508, 1455, 1228, 833, 749 cm^{-1}; ^{1}H NMR (400.13 MHz) ($CDCl_3$): δ 7.74 (d, *J* = 7.9 Hz, 1H), 7.70 (d, *J* = 7.8 Hz, 1H), 7.59 – 7.54 (m, 2H), 7.47 (d, *J* = 8.1 Hz, 1H), 7.45 – 7.40 (m, 2H), 7.39 – 7. 31 (m, 2H), 7.29 – 7.24 (m, 1H), 7.22 – 7.12 (m, 3H), 6.64 (s, 1H), 5.42 (s, 2H); ^{13}C NMR (100.6 MHz) ($CDCl_3$): δ 162.5 (*d*, J_{C-F} = 253.0 Hz, C), 145.8 (C), 138.8 (C), 136.6 (C), 133.6 (C), 133.3 (CH), 133.2 (*d*, J_{C-F} = 3.2 Hz, C), 132.9 (CH), 130.6 (CH), 128.2 (C), 127.5 (CH), 125.2 (C), 122.6 (CH), 122.5 (CH), 121.1 (CH), 120.5 (CH), 120.17 (CH), 116.7 (d, J_{C-F} = 23.5 Hz, CH), 110.4 (CH), 104.1 (CH), 40.0 (CH_2); ^{19}F NMR (376.5 MHz) ($CDCl_3$): δ -112.02 (m). HRMS: m/z $[M + H]^+$ calcd for $C_{23}H_{17}BrFN_4$: 447.0615; found: 447.0628.

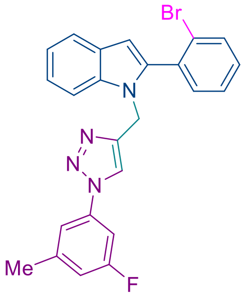

2-(2-bromophenyl)-1-((1-(2-fluoro-4-methylphenyl)-1*H*-1,2,3-triazol-4-yl)methyl)-1*H*-indole 6d: 77% yield; yellow solid; mp: 165 – 167 °C; IR (neat): 1507, 1336, 1021, 764, 747 cm^{-1}; ^{1}H NMR (400.13 MHz) ($CDCl_3$): δ 7.74 (d, *J* = 7.9 Hz, 1H), 7.72 – 7.66 (m, 2H), 7.52 (d, *J* = 8.3 Hz, 1H), 7.47 – 7.40 (m, 3H), 7.37 – 7.31 (m, 1H), 7.30 – 7.24 (m, 1H), 7.19 (t, *J* = 7.5 Hz, 1H), 7.07 – 7.00 (m, 2H), 6.62 (s, 1H), 5.42 (s, 2H), 2.40 (s, 3H); ^{13}C NMR (100.6 MHz) ($CDCl_3$): δ 153. 3 (d, J_{C-F} = 246.0 Hz, C), 145.1 (C), 141.3 (d, J_{C-F} = 7.6 Hz, C), 138.9 (C), 136.6 (C), 133.7 (C), 133.16 (CH),133.0 (CH), 130.6 (CH), 128.2 (C), 127.4 (CH), 125.8 (d, J_{C-F} = 3.2 Hz, CH), 125.2 (C), 124.5 (CH), 123.1 (*d*, J_{C-F} = 7.6 Hz, CH), 122.6 (*d*, J_{C-F} = 10.8 Hz, C), 122.3 (CH), 121.0 (CH), 120.4 (CH), 117.3 (*d*, J_{C-F} = 19.6 Hz, CH), 110.4 (CH), 103.8 (CH), 40.0 (CH_2), 21.2 (*d*, J_{C-F} = 1.4 Hz) (CH_3); ^{19}F NMR (376.5 MHz) ($CDCl_3$): δ -124.47 (m). HRMS: m/z $[M + H]^+$ calcd for $C_{24}H_{19}BrFN_4$: 461.0771; found: 461.0762.

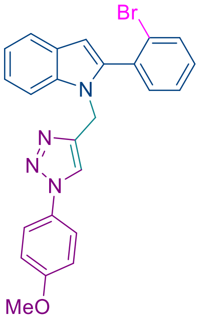

2-(2-bromophenyl)-1-((1-(4-methoxyphenyl)-1*H*-1,2,3-triazol-4-yl)methyl)-1*H*-indole 6e: 72% yield; yellow solid; mp: 76 – 78 °C; IR (neat): 1507, 1336, 1021, 834, 747 cm^{-1}; ^{1}H NMR (400.13 MHz) ($CDCl_3$): δ 7.74 (d, *J* = 7.8 Hz, 1H), 7.70 (d, *J* = 7.8 Hz, 1H), 7.50 – 7.47 (m, 3H), 7.43 – 7.41 (m, 2H), 7.36 – 7.33 (m, 2H), 7.28 – 7.25 (m, 1H), 7.20 (dt, J_1 = 7.4 Hz, J_2 = 0.80 Hz, 1H), 6.95 (d, *J* = 8.9 Hz, 2H), 6.63 (s, 1H), 5.41 (s, 2H), 3.85 (s, 3H); ^{13}C NMR (100.6 MHz) ($CDCl_3$): δ 159.9 (C), 145.5 (C), 138.8 (C), 136.6 (C), 133.6 (C), 133.2 (CH), 132.9 (CH), 130.6 (CH), 130.4 (C), 128.2 (C), 127.5 (CH), 125.2 (C), 122.4 (CH) 122.2 (CH), 121.0 (CH), 120.4 (CH) , 120.1 (CH), 114.8 (CH), 110.5 (CH), 104.0 (CH), 55.7 (CH_3), 40.2 (CH_2). HRMS: m/z [M + H]$^+$ calcd for $C_{24}H_{20}BrN_4O$: 459.0815; found: 459.0803.

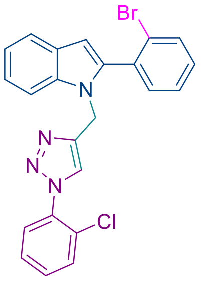

2-(2-bromophenyl)-1-((1-(2-chlorophenyl)-1*H*-1,2,3-triazol-4-yl)methyl)-1*H*-indole 6f: 72% yield; white solid; mp: 199 – 201 °C; IR (neat): 1586, 1533, 1465, 1226, 1022 cm^{-1}; ^{1}H NMR (400.13 MHz) ($CDCl_3$): δ 7.68 (d, *J* = 7.80 Hz, 1H), 7.50 – 7.46 (m, 2H), 7.44 (s, 1H), 7.40 – 7.36 (m, 4H), 7.33 (s, 1H), 7.31 (m, 2H), 7.04 (dd, J_1 = 8.5 Hz, J_2 = 1.2 Hz, 1H), 6.50 (d, *J* = 0.6 Hz, 1H), 5.34 (s, 2H), 2.43 (s, 3H); ^{13}C NMR (100.6 MHz) ($CDCl_3$): δ 146.09 (C), 138.85 (C), 135.4 (C), 135.00 (C), 134.60 (C), 133.68 (C), 133.31 (CH), 132.86 (CH), 130.54 (CH), 129.94 (CH), 129.88 (C), 128.45 (C), 127.52 (CH), 125.17 (C), 124.10 (CH), 121.68 (CH), 120.75 (CH), 119.86 (CH), 110.07 (CH), 103.72 (CH), 40.11 (CH_2), 21.53 (CH_3);). HRMS: m/z [M + H]$^+$ calcd for $C_{23}H_{17}BrClN_4$: 463.0320; found: 463.0334.

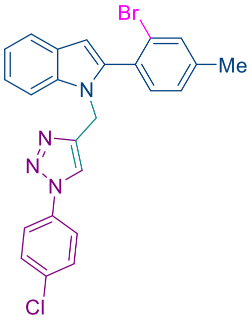

2-(2-bromo-4-methylphenyl)-1-((1-(4-chlorophenyl)-1*H*-1,2,3-triazol-4-yl)methyl)-1*H*-indole 6g: 73% yield; yellow solid; mp: 86 – 88 °C; IR (neat): 1456, 1309, 1040, 764, 747 cm^{-1}; ^{1}H NMR (400.13 MHz) ($CDCl_3$): δ 7.70 (d, *J* = 7.7 Hz, 1H), 7.60 (d, *J* = 8.2 Hz, 1H), 7.55 – 7.52 (m, 2H), 7.47 – 7.43 (m, 3H), 7.40 (s, 1H), 7.26 – 7.24 (m, 2H), 7.19 (td, J_1 = 7.5 Hz, J_2 = 0.8 Hz, 1H), 7.15 (dd, J_1 = 8.2 Hz, J_2 = 1.7 Hz, 1H), 6.62 (s, 1H), 5.43 (s, 2H), 2.36 (s, 3H); ^{13}C NMR (100.6 MHz) ($CDCl_3$): δ 146.0 (C), 138.9 (C), 137.6 (C), 136.6 (C), 135.4 (C), 134.6 (C), 133.5 (CH), 133.2 (C), 133.0 (CH), 131.5 (CH), 129.9 (CH), 128.2 (C), 122.4 (CH), 121.7 (CH), 121.6 (C), 121.1 (CH), 120.5 (CH), 119.9 (CH), 110.3 (CH), 104.0 (CH), 40.0 (CH_2), 21.0 (CH_3). HRMS: m/z [M + H]$^+$ calcd for $C_{24}H_{19}BrClN_4$: 477.0476; found: 477.0491.

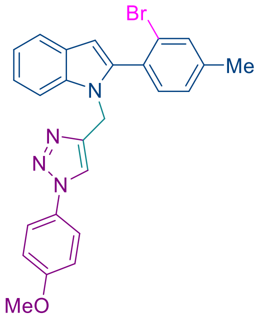

2-(2-bromo-4-methylphenyl)-1-((1-(4-methoxyphenyl)-1*H*-1,2,3-triazol-4-yl)methyl)-1*H*-indole 6h: 55% yield; pink solid; mp: 71 – 73 °C; IR (neat): 1517, 1319, 1250, 1031, 834, cm^{-1}; ^{1}H NMR (400.13 MHz) ($CDCl_3$): δ 7.59 (d, *J* = 7.7 Hz, 1H), 7.49 (d, *J* = 8.2 Hz, 1H), 7.40 – 7.36 (m, 3H), 7.23 (s, 1H), 7.17 – 7.13 (m, 2H), 7.08 (t, *J* = 7.4 Hz, 1H), 7.04 (dd, J_1 = 8.1 Hz, J_2 = 1.8 Hz, 1H), 6.85 (d, *J* = 9.0 Hz, 2H), 6.51 (s, 1H), 5.32 (s, 2H), 3.74 (s, 3H), 2.25 (s, 3H); ^{13}C NMR (100.6 MHz) ($CDCl_3$): δ 159.9 (C), 145.5 (C), 139.0 (C), 137.5 (C), 136.6 (C), 133.5 (CH), 133.3 (C), 132.9 (CH), 131.44 (C), 130.4 (CH), 128.2 (C), 122.3 (CH), 122.2 (CH), 121.7 (CH), 121.0 (CH), 120.4 (CH), 120.2 (C), 114.7 (CH), 110.3 (CH), 103.9 (CH), 55.6 (CH_3), 40.2 (CH_2), 20.9 (CH_3). HRMS: m/z [M + H]$^+$ calcd for $C_{25}H_{22}BrN_4O$: 473.0971; found: 473.0965.

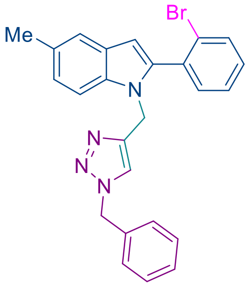

1-((1-benzyl-1*H*-1,2,3-triazol-4-yl)methyl)-2-(2-bromophenyl)-5-methyl-1*H*-indole 6i: 82% yield; orange solid; mp: 85 – 87 °C; IR (neat): 1506, 1455, 1125, 1022, 749 cm^{-1}; ^{1}H NMR (400.13 MHz) ($CDCl_3$): δ 7.53 – 7.51 (m, 1H), 7.31 (s, 1H), 7.21 – 7.10 (m, 7H), 7.00 – 6.97 (m, 2H), 6.93 (dd, J_1 = 8.4 Hz, J_2 = 1.1 Hz, 1H), 6.76 (s, 1H), 6.33 (s, 1H), 5.19 (s, 2H), 5.15 (s, 2H), 2.34 (s, 3H); ^{13}C NMR (100.6 MHz) ($CDCl_3$): δ 145.4 (C), 138.9 (C), 134.9 (C), 134.6 (C), 133.7 (C), 133.0 (CH), 132.8 (CH), 130.3 (CH), 129.5 (C), 129.0 (CH), 128.7 (CH), 128.3 (C), 127.9 (CH), 127.2 (CH), 125.0 (C), 123.8 (CH), 121.7 (CH), 120.5 (CH), 110.0 (CH), 103.2 (CH), 54.0 (CH_2), 40.1 (CH_2), 51.5 (CH_3). HRMS: m/z [M + H]$^+$ calcd for $C_{25}H_{22}BrN_4$: 457.1022; found: 457.1039.

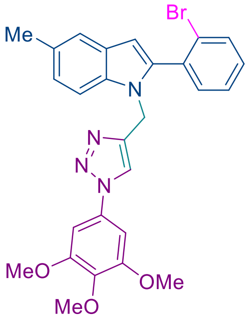

2-(2-bromophenyl)-5-methyl-1-((1-(3,4,5-trimethoxyphenyl)-1*H*-1,2,3-triazol-4-yl)methyl)-1*H*-indole 6j: 80% yield; yellow solid; mp: 74 – 76 °C; IR (neat): 1499, 1456, 1120, 1041, 789 cm^{-1}; ^{1}H NMR (400.13 MHz) ($CDCl_3$):

δ 7.64 (d, J = 8.1 Hz, 1H), 7.40 (s, 1H), 7.34 – 7.31 (m, 3H), 7.28 – 7.21 (m, 2H), 7.00 (dd, J_1 = 8.5 Hz, J_2 = 1.2 Hz, 1H), 6.71 (s, 2H), 6.47 (s, 1H), 5.30 (s, 2H), 3.79 (s, 6H), 3.77 (s, 3H), 2.39 (s, 3H); ^{13}C NMR (100.6 MHz) ($CDCl_3$): δ 153.9 (C), 145.7 (C), 138.8 (C), 138.4 (C), 135.0 (C), 133.7 (C), 133.3 (CH), 132.9 (CH), 132.8 (C), 130.5 (CH), 129.9 (C), 128.4 (C), 127.5 (CH), 125.1 (C), 124.1 (CH), 120.7 (CH), 120.2 (CH), 110.1 (CH), 103.7 (CH), 98.6 (CH), 61.1 (CH_3), 56.6 (CH_3), 40.1 (CH_2), 21.5 (CH_3). HRMS: m/z [M + H]$^+$ calcd for $C_{27}H_{26}BrN_4O_3$: 501.1284; found: : 501.1298.

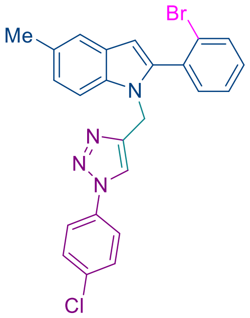

2-(2-bromophenyl)-1-((1-(4-chlorophenyl)-1*H*-1,2,3-triazol-4-yl)methyl)-5-methyl-1*H*-indole 6k: 83% yield; yellow solid; mp: 197 – 199 °C; IR (neat): 1596, 1513, 1455, 1226, 1022 cm^{-1}; ^{1}H NMR (400.13 MHz) ($CDCl_3$): δ 7.68 (d, J = 7.8 Hz, 1H), 7.50 – 7.46 (m, 2H), 7.44 (s, 1H), 7.40 – 7.36 (m, 4H), 7.33 (s, 1H), 7.31 – 7.27 (m, 2H), 7.04 (dd, J_1 = 8.4 Hz, J_2 = 1.2 Hz, 1H), 6.50 (d, J = 0.6 Hz, 1H), 5.34 (s, 2H), 2.43 (s, 3H);^{13}C NMR (100.6 MHz) ($CDCl_3$): δ 146.1 (C), 138.8 (C), 135.4 (C), 135.0 (C), 134.6 (C), 133.7 (C), 133.3 (CH), 132.8 (CH), 130.5 (CH), 129.9 (CH), 129.8 (C), 128.4 (C), 127.5 (CH), 125.2 (C), 124.1 (CH), 121.7 (CH), 120.7 (CH), 119.8 (CH), 110.1 (CH), 103.72 (CH), 40.1 (CH_2), 21.5 (CH_3). HRMS: m/z [M + H]$^+$ calcd for $C_{24}H_{19}BrClN_4$: 477.0476; found: 477.0461.

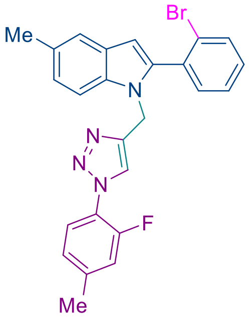

2-(2-bromophenyl)-1-((1-(2-fluoro-4-methylphenyl)-1*H*-1,2,3-triazol-4-yl)methyl)-5-methyl-1*H*-indole 6l: 76% yield; brown solid; mp: 162 – 162 °C; IR (neat): 1506, 1463, 1032, 1019, 749 cm^{-1}; ^{1}H NMR (400.13 MHz) ($CDCl_3$): δ 7.62 (dd, J_1 = 7.8 Hz, J_2 = 0.8 Hz, 1H), 7.58 (t, J = 8.0 Hz, 1H), 7.37 – 7.28 (m, 5H), 7.24 – 7.21 (m, 1H), 6.98 (td, J_1 = 8.4 Hz, J_2 = 1.2 Hz, 2H), 6.93 (d, J = 11.4 Hz, 1H), 6.42 (d, J = 0.6 Hz, 1H), 5.28 (s, 2H), 2.38 (s, 3H), 2.30 (s, 3H); ^{13}C NMR (100.6 MHz) ($CDCl_3$): δ 153.2 (d, J_{C-F} = 254.0 Hz, C), 145.3 (C), 141.3 (d, J_{C-F} =8.1 Hz (C), 139.0 (C), 135.0 (C), 133.9 (C), 133.2 (CH), 133.1 (CH), 130.5 (CH), 129.7 (C), 128.5 (C), 127.4 (CH), 125.8 (d, J_{C-F} = 2.9 Hz, CH), 125.3 (C), 124.6 (CH), 124.0 (CH), 123.1 (d, J_{C-F} = 7.35 Hz, CH), 122.8 (d, J_{C-F} = 11.12 Hz, C), 120.7 (CH), 117.3 (d, J_{C-F} =20.5 Hz, CH), 110.1 (CH), 103.4 (CH), 40.1 (CH_2), 21.5 (CH_3), 21.3 (CH_3); ^{19}F NMR (376.5 MHz) ($CDCl_3$): δ -124.44 (m). HRMS: m/z [M + H]$^+$ calcd for $C_{25}H_{21}BrFN_4$: 475.0928; found: 475.0919.

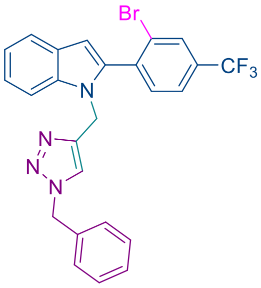

1-((1-benzyl-1*H*-1,2,3-triazol-4-yl)methyl)-2-(2-bromo-4-(trifluoromethyl)phenyl)-1*H*-indole 6m: 77% yield; orange solid; mp: 102 – 104 °C; IR (neat): 1499, 1456, 1310, 1020, 748 cm^{-1}; ^{1}H NMR (400.13 MHz) ($CDCl_3$): δ 7.83 (s, 1H), 7.58 (d, *J* = 7.7 Hz, 1H), 7.45 (d, *J* = 7.9 Hz, 1H), 7.32 (dd, J_1 = 5.6 Hz, J_2 = 1.6 Hz, 2H), 7.26 – 7.24 (m, 3H), 7.18 – 7.14 (m, 1H), 7.09 (t, *J* = 7.5 Hz, 1H), 7.05 – 7.03 (m, 2H), 6.79 (s, 1H), 6.48 (s, 1H), 5.28 (s, 2H), 5.19 (s, 2H); ^{13}C NMR (100.6 MHz) ($CDCl_3$): 145.0 (C), 137.5 (C), 137.4 (C), 136.9 (C), 134.6 (C), 133.3 (CH), 132.5 (q, $J_{C\text{-}F}$ = 33. 0 Hz), 130.14 (q, $J_{C\text{-}F}$ = 3.6 Hz, CH), 129.2 (CH), 128.9 (CH), 128.03 (CH), 128.01 (C), 125.4 (C), 124.4 (C), 124.2 (q, $J_{C\text{-}F}$ = 3.6 Hz, CH), 122.8 (CH), 122.6 (q, $J_{C\text{-}F}$ = 273.0 Hz) 121.7 (CH), 121.2 (CH), 120.6 (CH), 110.4 (CH), 104.6 (CH), 54.2 (CH_2), 40.2 (CH_2). ^{19}F NMR (376.5 MHz) ($CDCl_3$): δ -62.79 (s). HRMS: m/z $[M + H]^+$ calcd for $C_{25}H_{19}BrF_3N_4$: 511.0740; found: 511.0754.

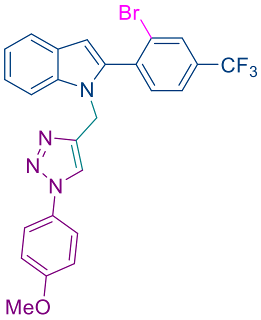

2-(2-bromo-4-(trifluoromethyl)phenyl)-1-((1-(4-methoxyphenyl)-1*H*-1,2,3-triazol-4-yl)methyl)-1*H*-indole 6n: 75% yield; orange solid; mp: 76 – 78 °C; IR (neat): 1499, 1456, 1043, 1023, 783 cm^{-1}; ^{1}H NMR (400.13 MHz) ($CDCl_3$): δ 8.01 (s, 1H), 7.72 (d, *J* = 7.9 Hz, 1H), 7.69 (dd, J_1 = 7.8 Hz, J_2 = 1.0 Hz, 1H), 7.58 (d, *J* = 7.8 Hz, 1H), 7.51 – 7.47 (m, 3H), 7.36 (s, 1H), 7.32 – 7.28 (m, 1H), 7.22 (td, J_1 = 7.8 Hz, J_2 = 0.9 Hz, 1H), 6.96 (d, *J* = 8.0 Hz, 2H), 6.67 (s, 1H), 5.41 (s, 2H), 3.85 (s, 3H); ^{13}C NMR (100.6 MHz) ($CDCl_3$): δ 159.9 (C), 145.1 (C), 137.5 (C), 137.4 (C), 136.9 (C), 133.4 (CH), 133.5 (q, $J_{C\text{-}F}$ = 33.0 Hz, C), 130.3 (q, $J_{C\text{-}F}$ = 3.8 Hz, CH), 128.1 (C), 125.5 (C), 124.4 (q, $J_{C\text{-}F}$ = 3.4 Hz, CH), 123.1 (q, $J_{C\text{-}F}$ = 275.0 Hz) 122.9 (overlapping, CH), 122.2 (CH), 121.7 (C), 121.3 (CH), 120.7 (CH), 120.0 (CH), 114.8 (CH), 110.5 (CH), 104.8 (CH), 55.7 (CH_3), 40.6 (CH_2); ^{19}F NMR (376.5 MHz) ($CDCl_3$): δ -62.77 (s). HRMS: m/z $[M + H]^+$ calcd for $C_{25}H_{19}BrF_3N_4O$: 527.0689; found: 527.0673.

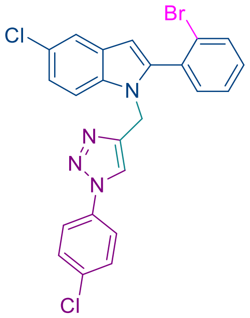

2-(2-bromophenyl)-5-chloro-1-((1-(4-chlorophenyl)-1*H*-1,2,3-triazol-4-yl)methyl)-1*H*-indole 6o: 73% yield; yellow solid; mp: 188 – 190 °C; IR (neat): 1499, 1456, 1020, 988, 750 cm^{-1}; ^1H NMR (400.13 MHz) ($CDCl_3$): δ 7.65 (d, *J* = 8.0 Hz, 1H), 7.55 (d, *J* = 1.9 Hz, 1H), 7.44 (m, 2H), 7.36 – 7.35 (m, 1H), 7.34 – 7.31 (m, 3H), 7.29 – 7.27 (m, 2H), 7.11 (dd, J_1 = 8.6 Hz, J_2 = 2.0 Hz, 1H), 6.47 (s, 1H), 5.28 (s, 2H); ^{13}C NMR (100.6 MHz) ($CDCl_3$): δ 145.5 (C), 140.1 (C), 135.3 (C), 134.9 (C), 134.8 (C), 133.4 (CH), 133.2 (C), 132.8 (CH), 130.9 (CH), 130.0 (CH), 129.2 (C), 127.6 (CH), 126.3 (C), 125.1 (C), 122.8 (CH), 121.7 (CH), 120.5 (CH), 119.9 (CH), 111.5 (CH), 103.7 (CH), 40.2 (CH_2). HRMS: m/z $[M + H]^+$ calcd for $C_{23}H_{16}BrCl_2N_4$: 496.9930; found: 496.9914.

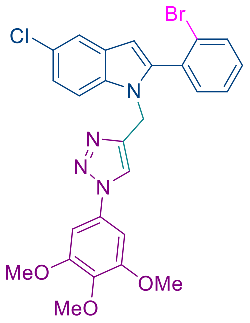

2-(2-bromophenyl)-5-chloro-1-((1-(3,4,5-trimethoxyphenyl)-1*H*-1,2,3-triazol-4-yl)methyl)-1*H*-indole 6p: 71% yield; yellow solid; mp: 152 – 154 °C; IR (neat): 1499, 1456, 1020, 988, 831 cm^{-1}; ^1H NMR (400.13 MHz) ($CDCl_3$): δ 7.65 (d, *J* = 7.9 Hz, 1H), 7.55 (d, *J* = 1.9 Hz, 1H), 7.35 – 7.30 (m, 3H), 7.29 (s, 1H), 7.28 – 7.23 (m, 1H), 7.10 (dd, J_1 = 8.8 Hz, J_2 = 2.0 Hz, 1H), 6.70 (s, 2H), 6.47 (s, 1H), 5.28 (s, 2H), 3.78 (s, 6H), 3.77 (s, 3H); ^{13}C NMR (100.6 MHz) ($CDCl_3$): δ 153.9 (C), 145.0 (C), 140.0 (C), 138.5 (C), 135.0 (C), 133.3 (CH), 133.1 (C), 132.8 (CH), 132.8 (C), 130.8 (CH), 129.0 (C), 127.63 (CH), 126.2 (C), 125.0 (C), 122.7 (CH), 120.4 (CH), 120.3 (CH), 111.5 (CH), 103.7 (CH), 98.6 (CH), 61.1 (CH_3), 56.6 (CH_3), 40.1 (CH_2). HRMS: m/z $[M + H]^+$ calcd for $C_{26}H_{23}BrClN4O_3$: 553.0637; found: 553.0645.

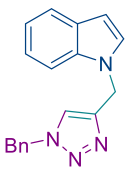

1-((1-benzyl-1*H*-1,2,3-triazol-4-yl)methyl)-1*H*-indole: 42% yield; yellow wax; IR (neat): 3060, 1340, 1130, 700 cm^{-1}; ^1H NMR (400.13 MHz) ($CDCl_3$): δ 7.53 (d, *J* = 7.8 Hz, 1H), 7.28 – 7.21 (m, 4H), 7.13 – 6.99 (m, 6H), 6.41 (s, 1H), 5.40 – 5.20 (m, 4H); ^{13}C NMR (100.6 MHz) ($CDCl_3$): δ 145.2 (C), 135.9 (C), 134.4 (C), 129.1 (CH), 128.79

(CH), 128.77 (C) 128.0 (CH), 127.8 (CH), 121.9 (CH), 121.6 (CH), 121.1 (CH), 119.7 (CH), 109.5 (CH), 102.1 (CH), 54.2 (CH_2), 42.1 (CH_2); HRMS: m/z $[M + H]^+$ calcd for $C_{18}H_{17}N_4$: 289.1448; found: 289.1436.

4. REFERENCES

References [39, 43, and 44] are cited in the manuscript.

[39] Cacchi, S.; Fabrizi, G.; Goggiamani, A.; Iazzetti, A.; Verdiglione, R. A facile palladium-catalyzed route to 2,5,7-trisubstituted indoles. *Tetrahedron* **2015,** *71* (49), 9346-9356, https://doi.org/10.1016/j.tet.2015.10.002.
[43] Wilkening, I.; del Signore, G.; Hackenberger, C. P. J. C. C., Synthesis of phosphonamidate peptides by Staudinger reactions of silylated phosphinic acids and esters. *Chem. Commun.,* **2011,** *47* (1), 349-351, DOI: 10.1039/C0CC02472D.
[44] Wang, F.-C.; Peng, B.; Ren, T.-T.; Liu, S.-P.; Du, J.-R.; Chen, Z.-H.; Zhang, T.-T.; Gu, X.; Li, M.; Cao, S.-L. J. J. o. M. C., A 1, 2, 3-Triazole derivative of quinazoline exhibits antitumor activity by tethering RNF168 to SQSTM1/P62. *J. Med. Chem.*, **2022**, *65* (22), 15028-15047, https://doi.org/10.1021/acs.jmedchem.2c00432.

^{1}H,^{13}C, DEPT 135, ^{19}F NMR Spectra

^{1}H NMR-spectrum (400 MHz, $CDCl_3$)

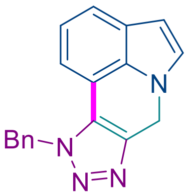

1a

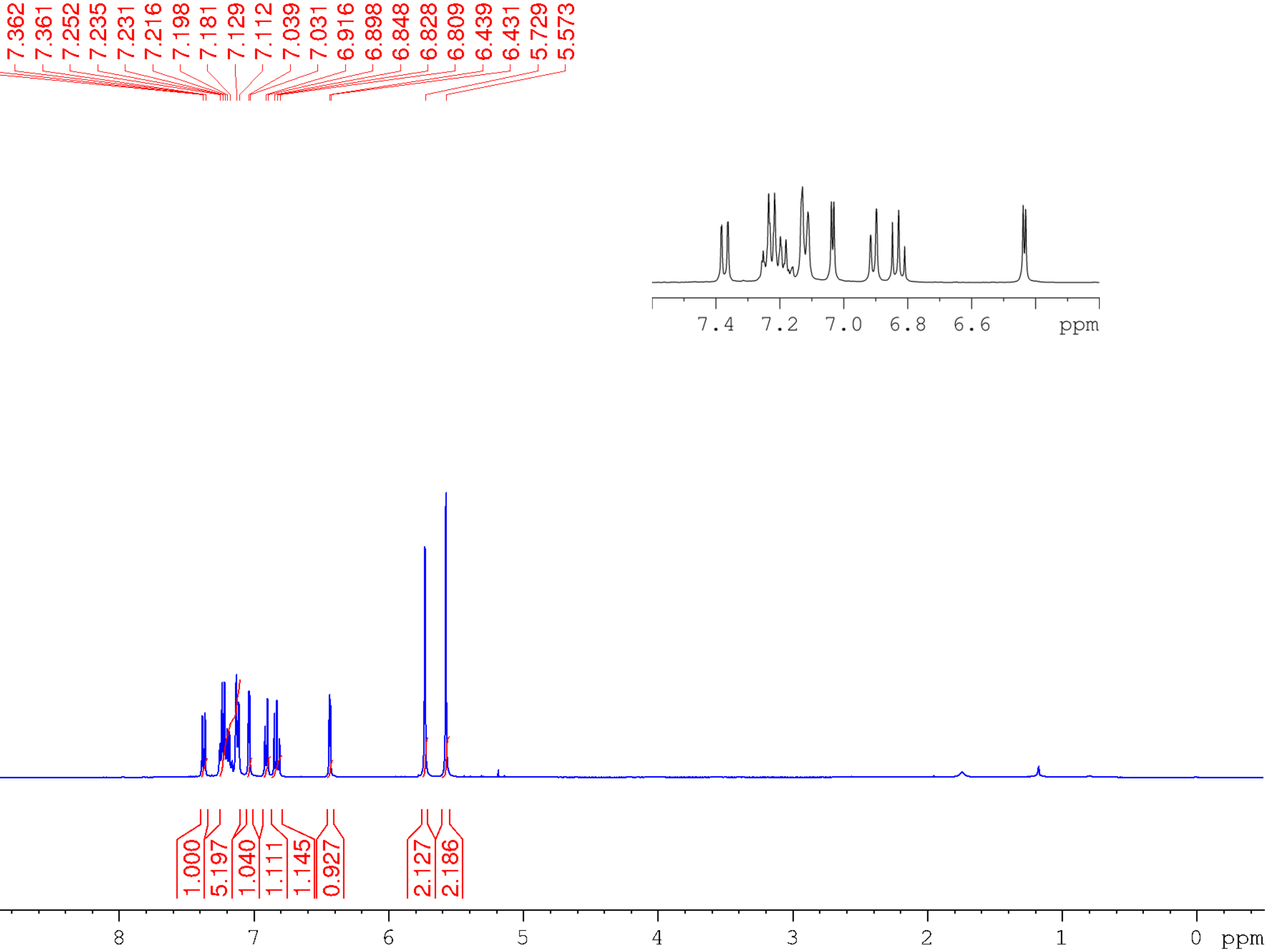

^{13}C NMR-spectrum (100 MHz, $CDCl_3$)

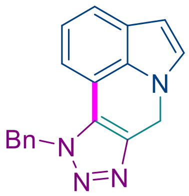

1a

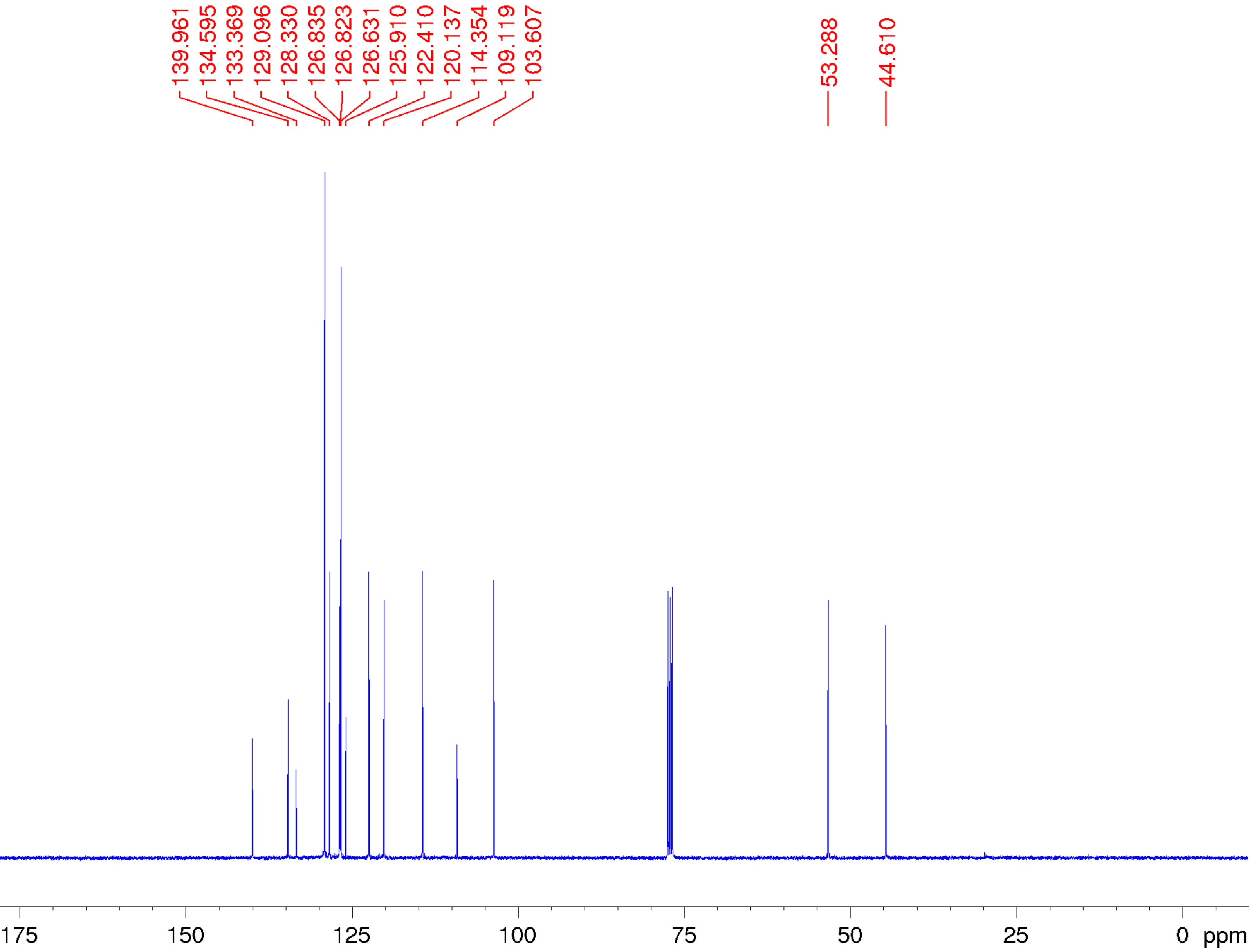

DEPT 135 NMR-spectrum ($CDCl_3$)

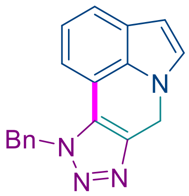

1a

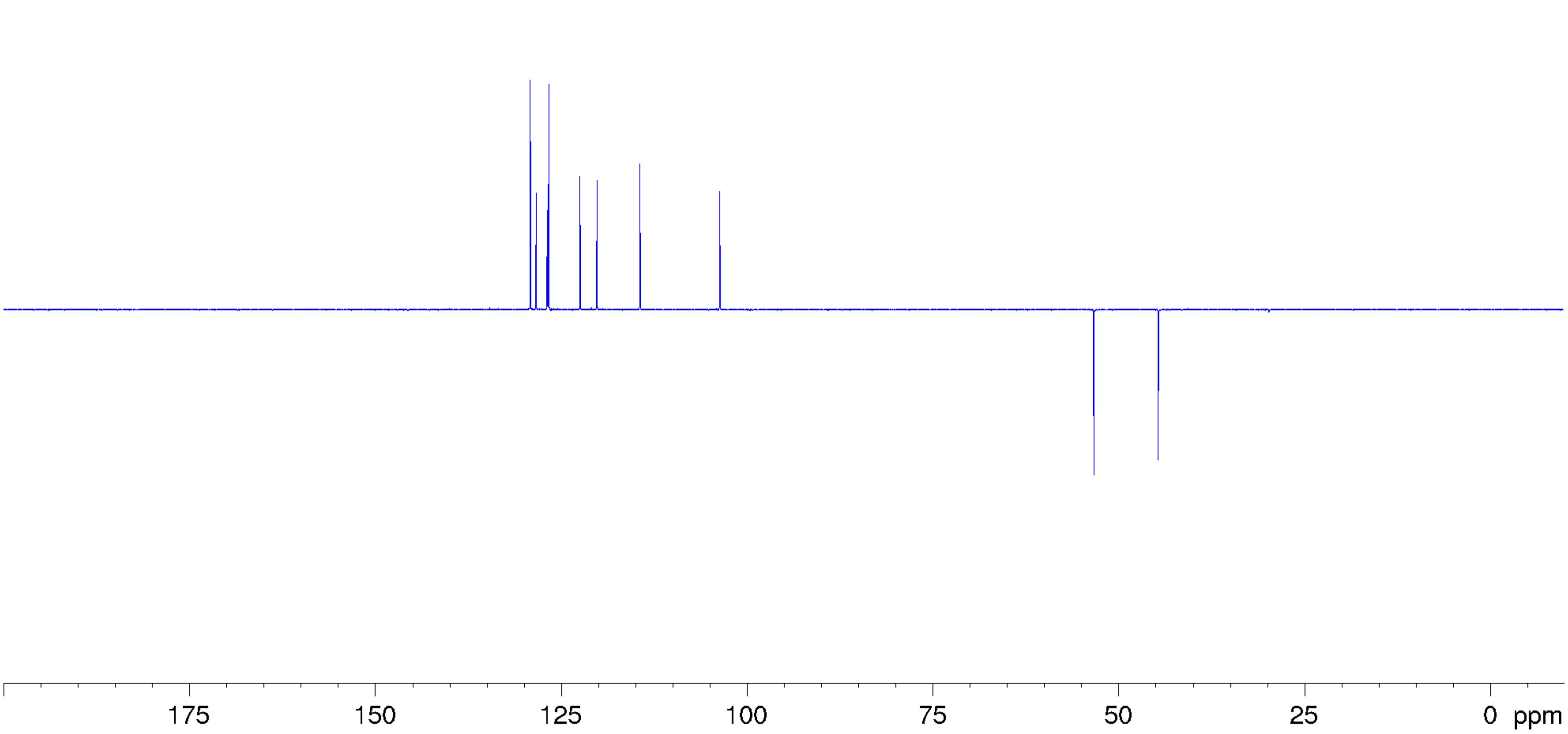

^{1}H NMR-spectrum (400 MHz, $CDCl_3$)

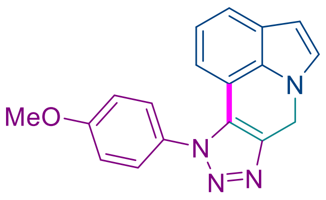

1b

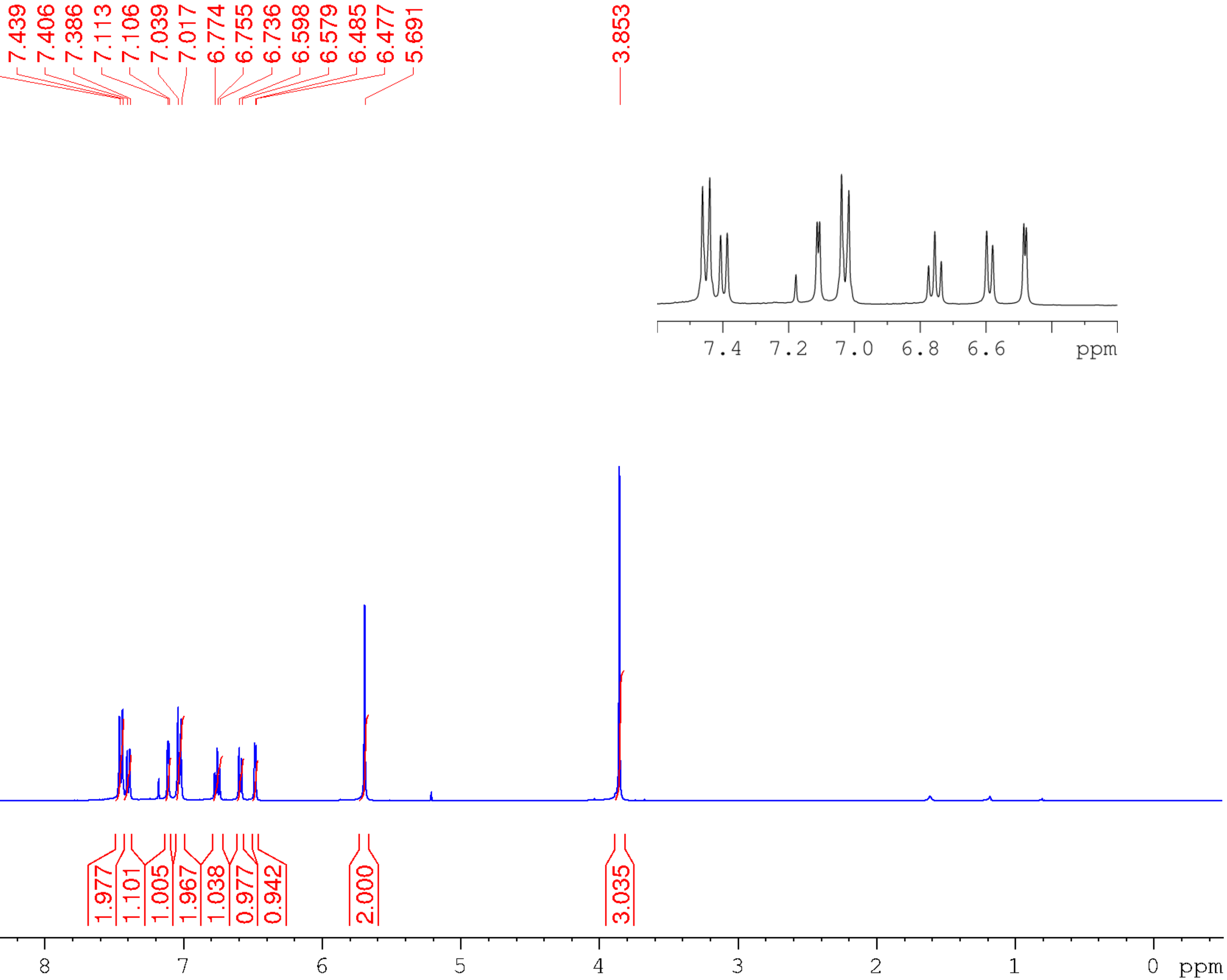

^{13}C NMR-spectrum (100 MHz, $CDCl_3$)

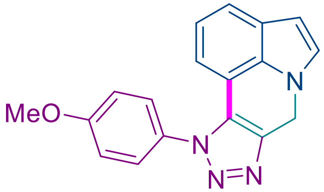

1b

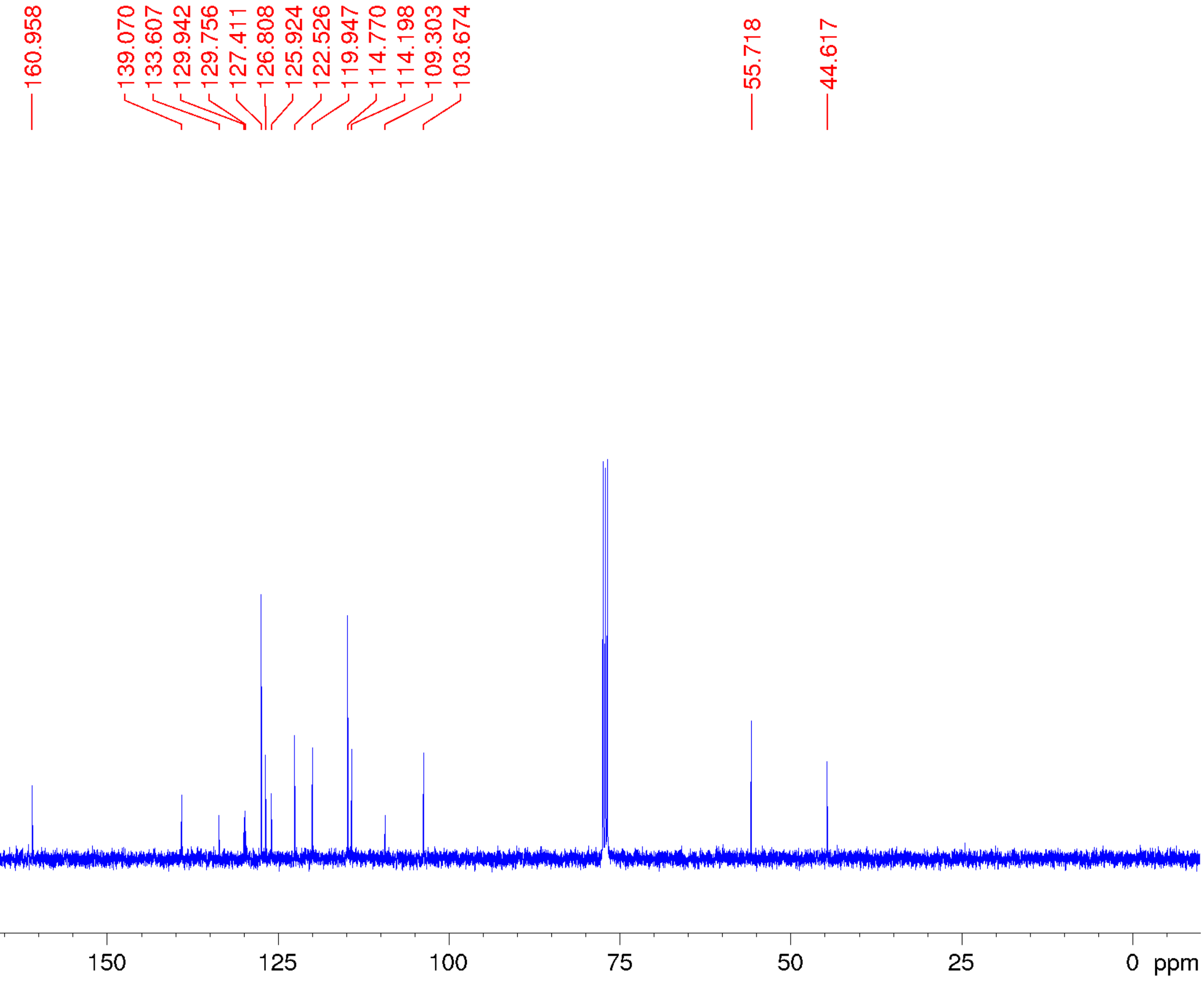

DEPT 135 NMR-spectrum ($CDCl_3$)

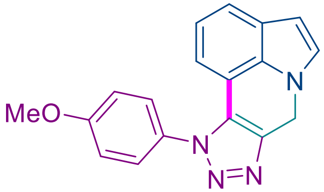

1b

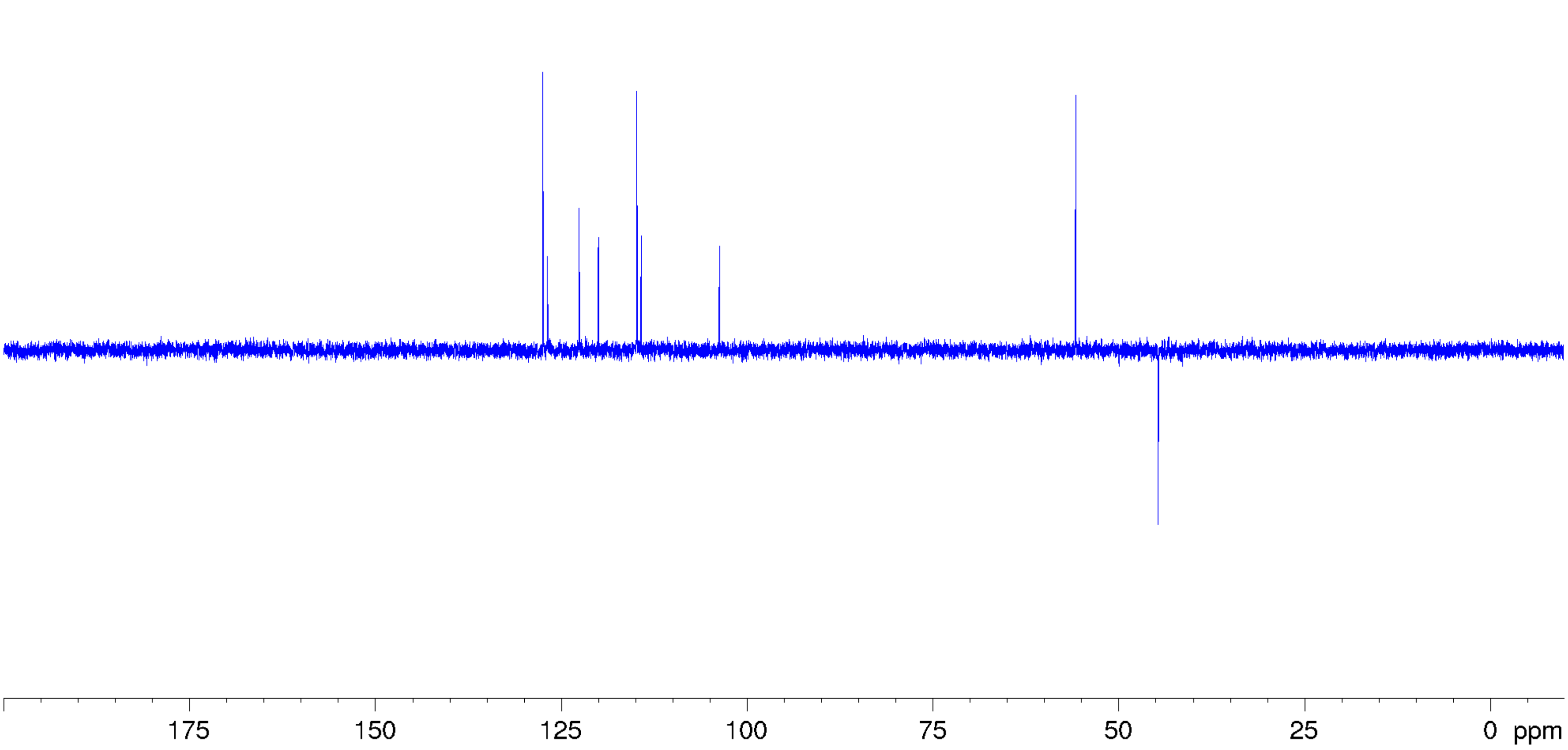

^{1}H NMR-spectrum (400 MHz, $CDCl_3$)

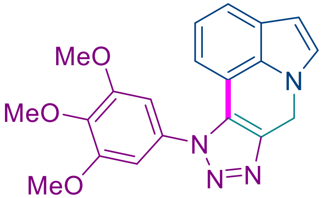

1c

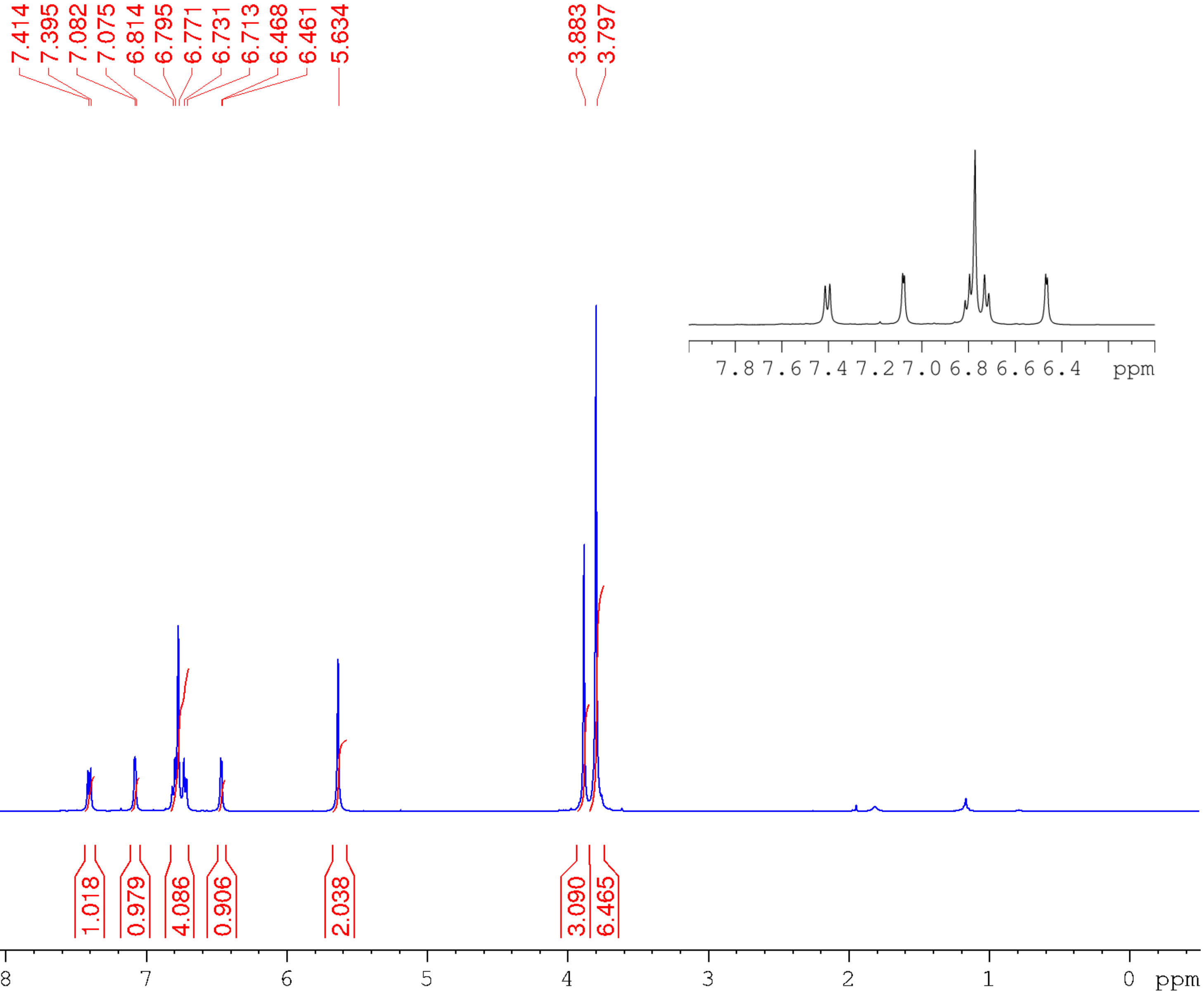

^{13}C NMR-spectrum (100 MHz, $CDCl_3$)

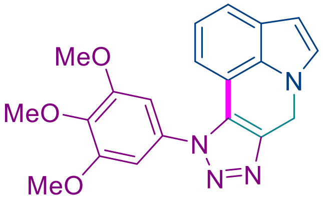

1c

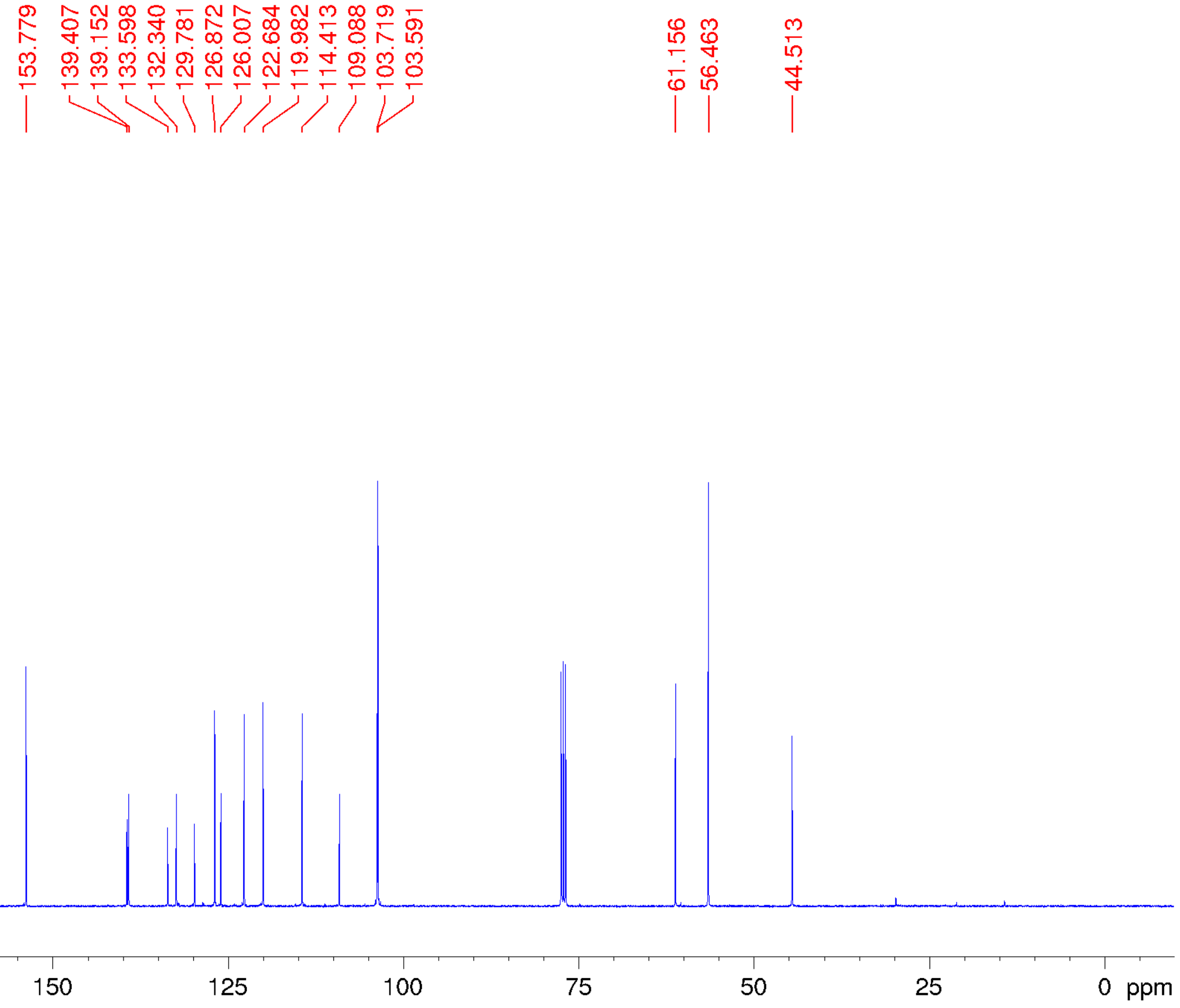

DEPT 135 NMR-spectrum ($CDCl_3$)

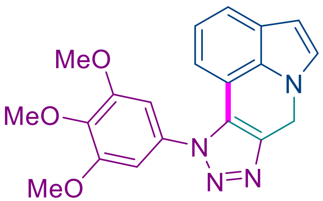

1c

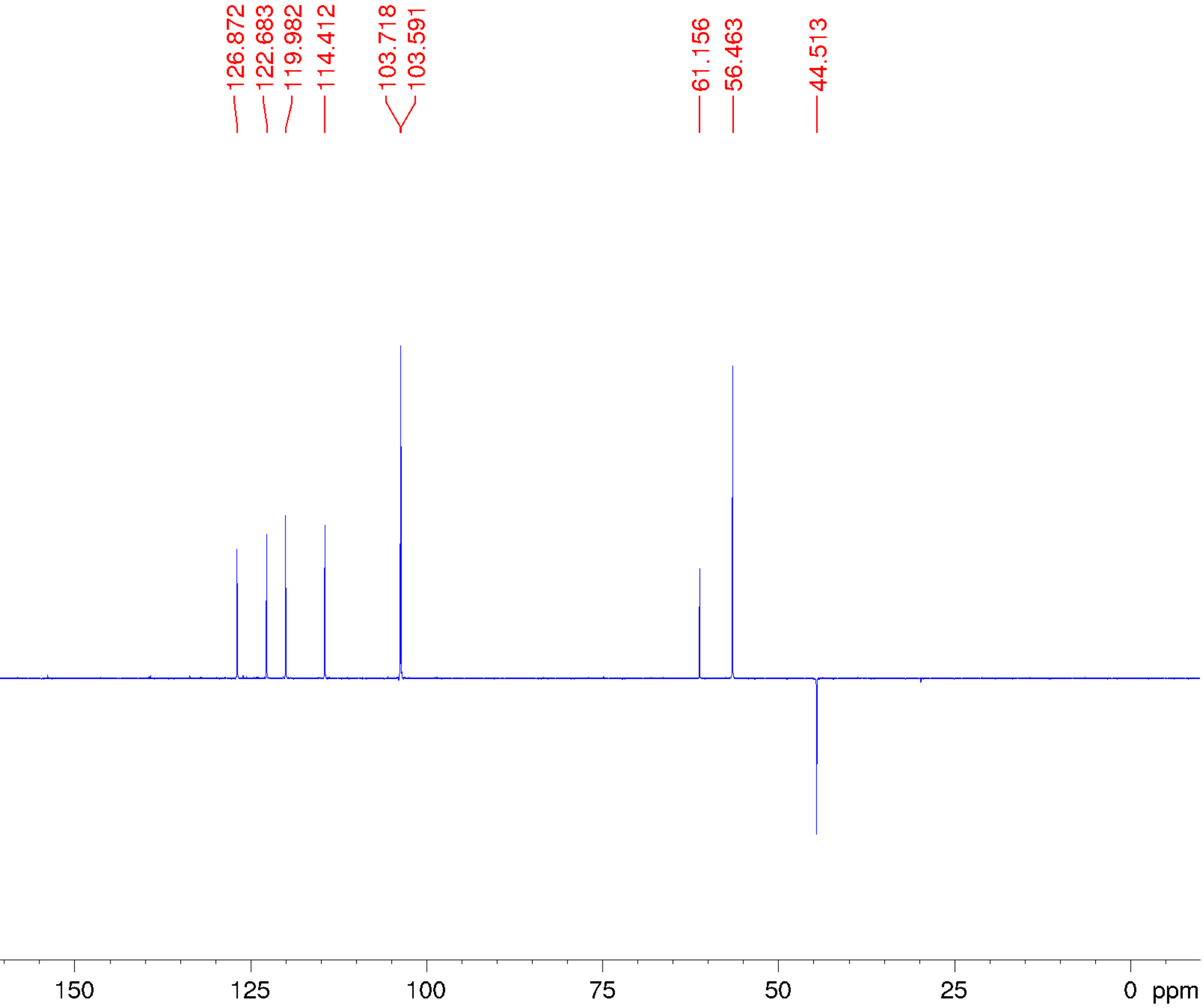

^{1}H NMR-spectrum (400 MHz, $CDCl_3$)

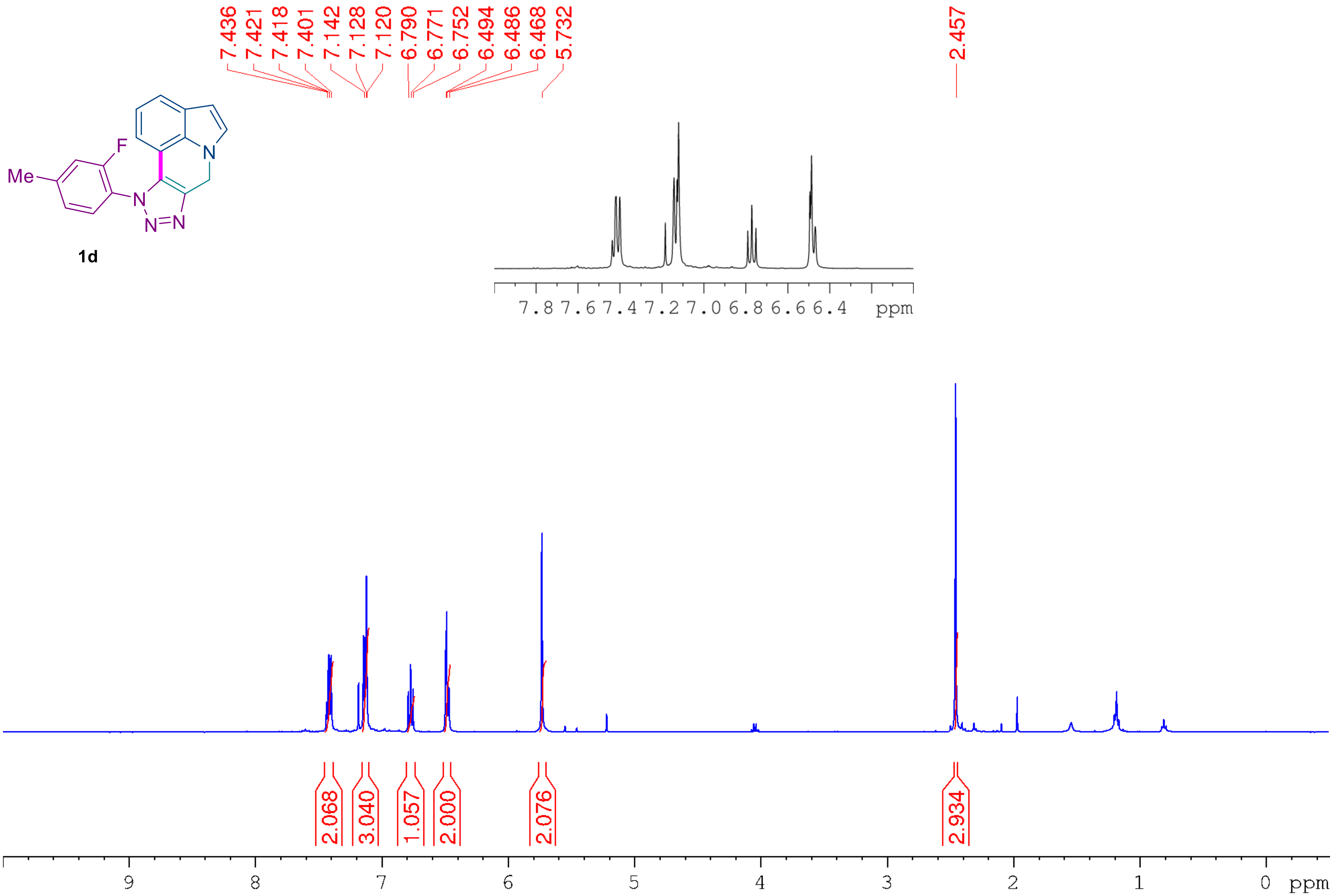

1d

^{13}C NMR-spectrum (100 MHz, $CDCl_3$)

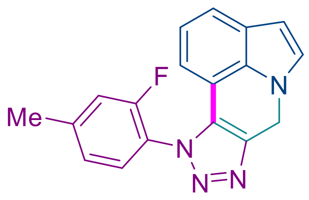

1d

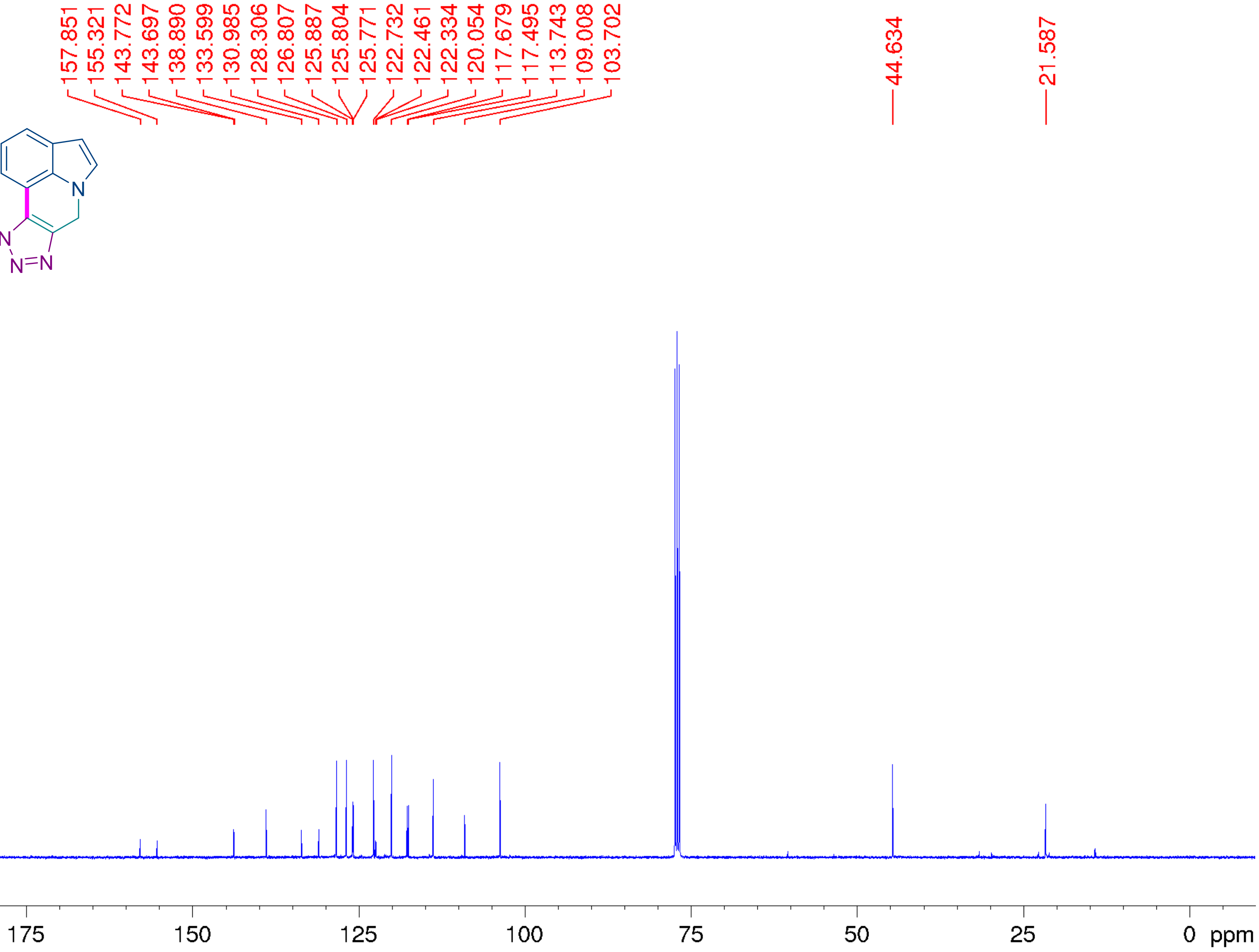

DEPT 135 NMR-spectrum ($CDCl_3$)

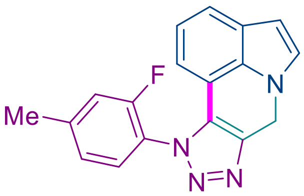

1d

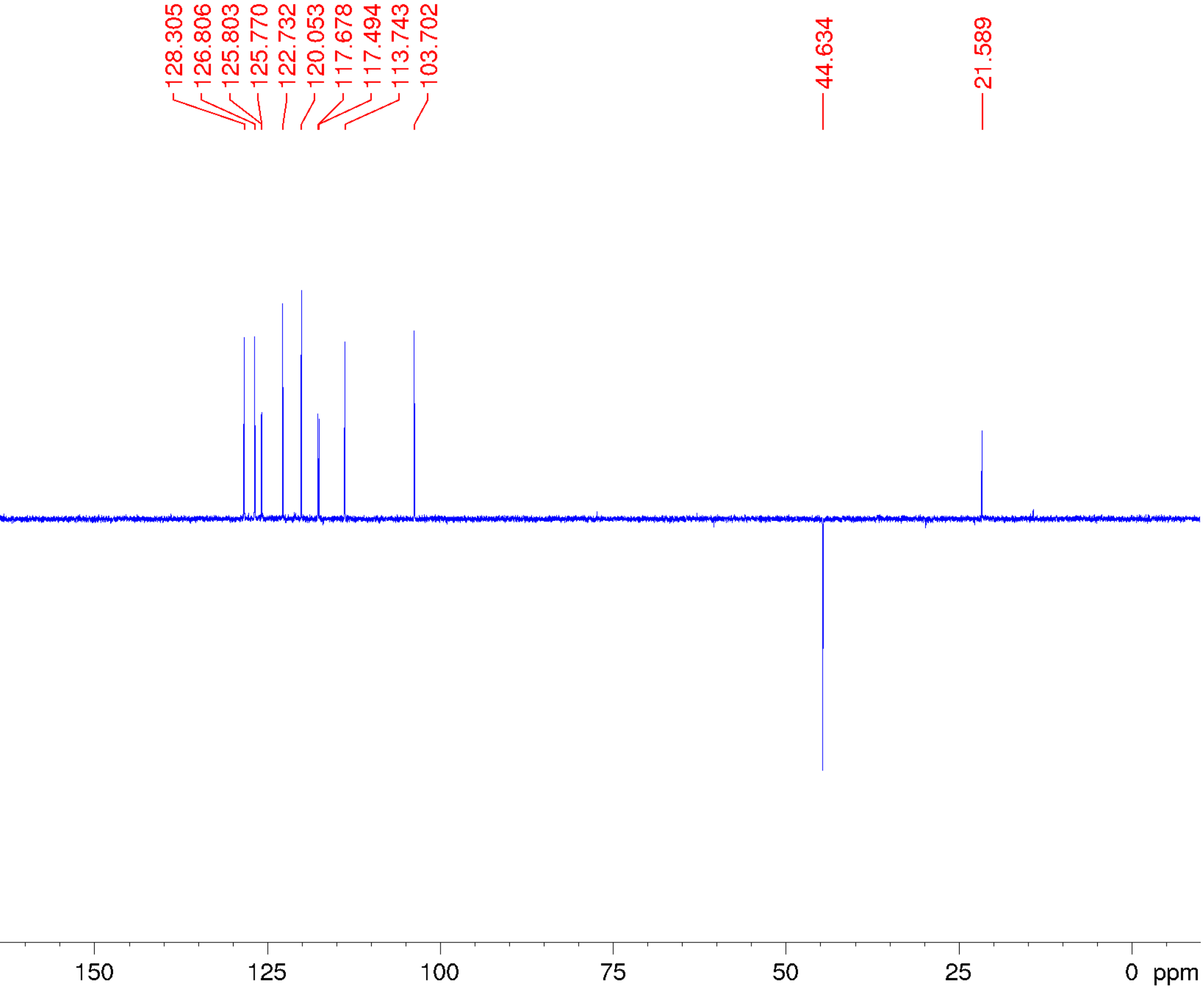

^{19}F NMR-spectrum (376.5 Hz, $CDCl_3$)

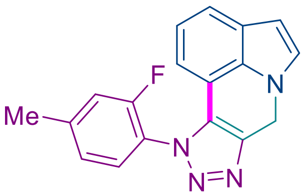

1d

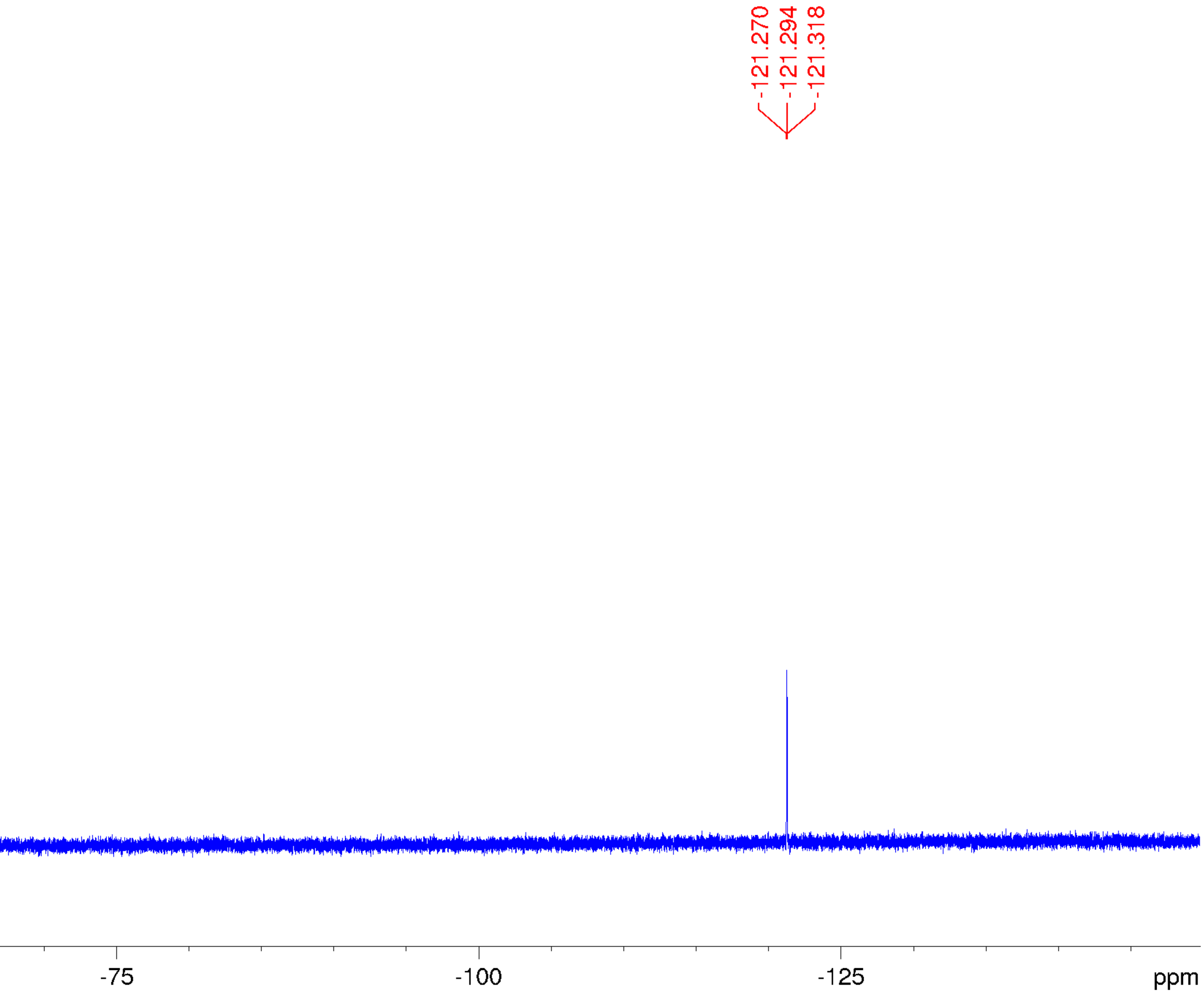

^{1}H NMR-spectrum (400 MHz, $CDCl_3$)

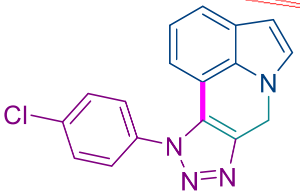

1e

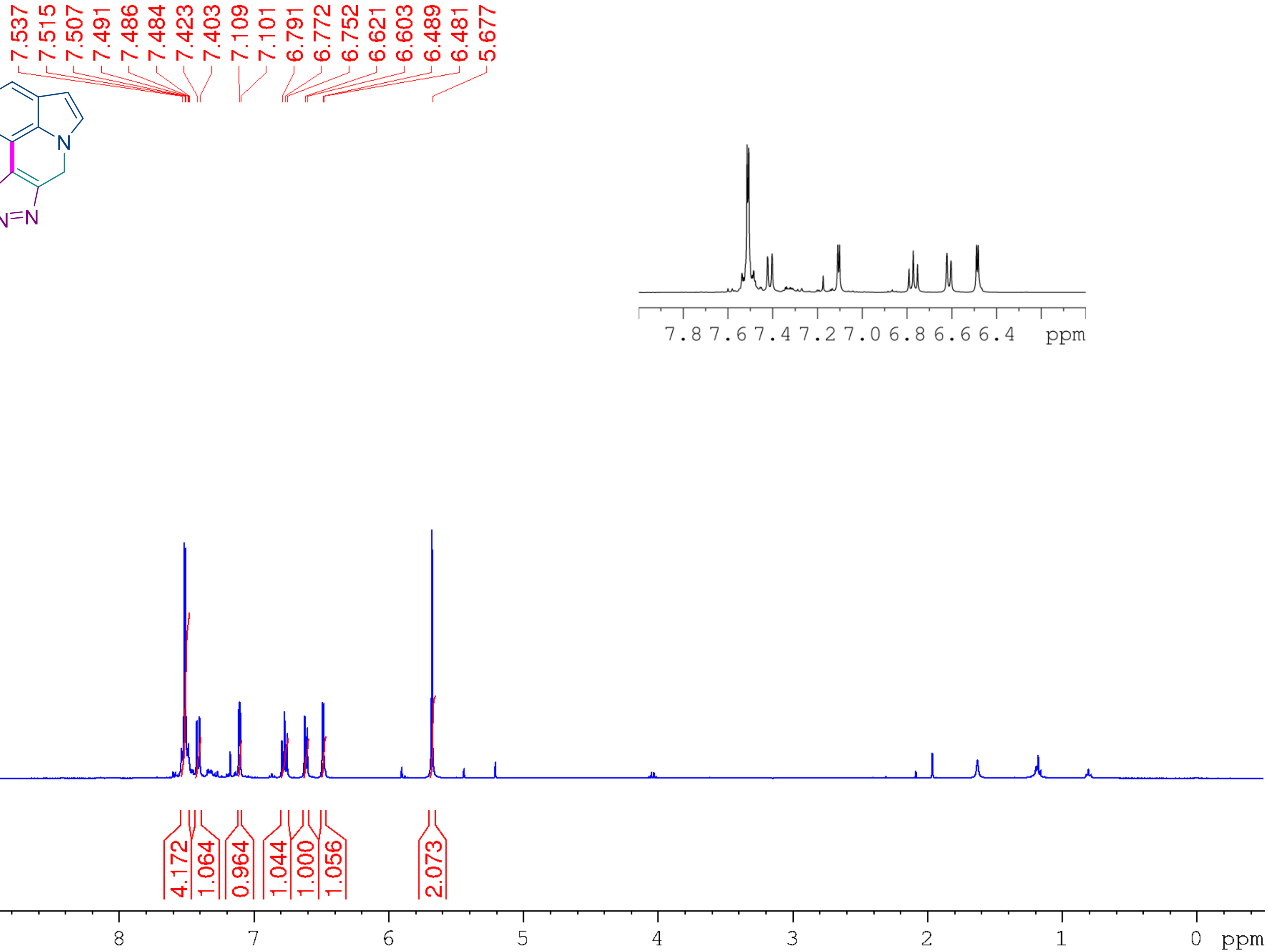

^{13}C NMR-spectrum (100 MHz, $CDCl_3$)

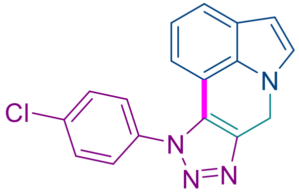

1e

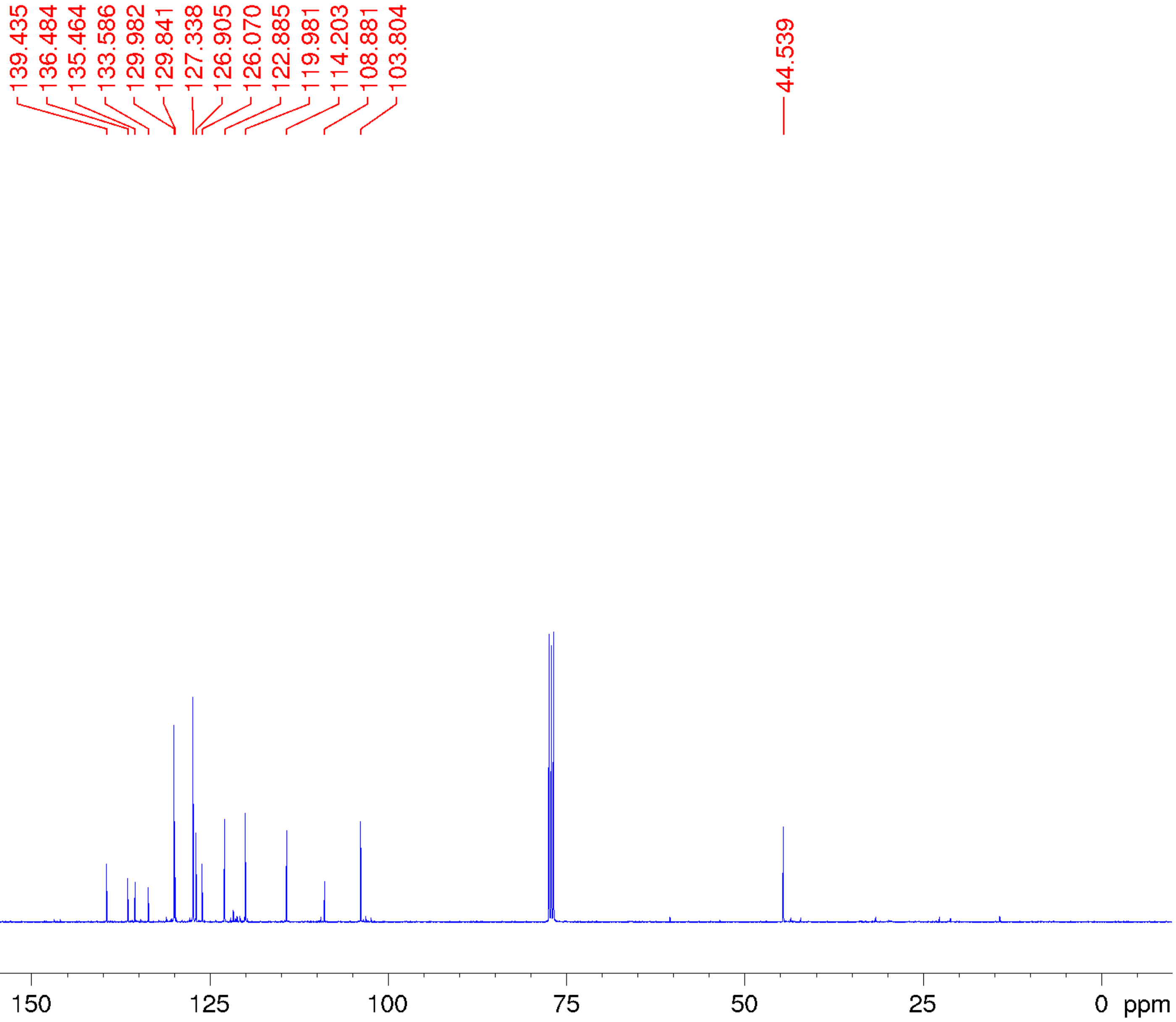

DEPT 135 NMR-spectrum ($CDCl_3$)

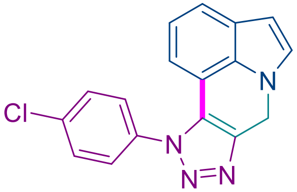

1e

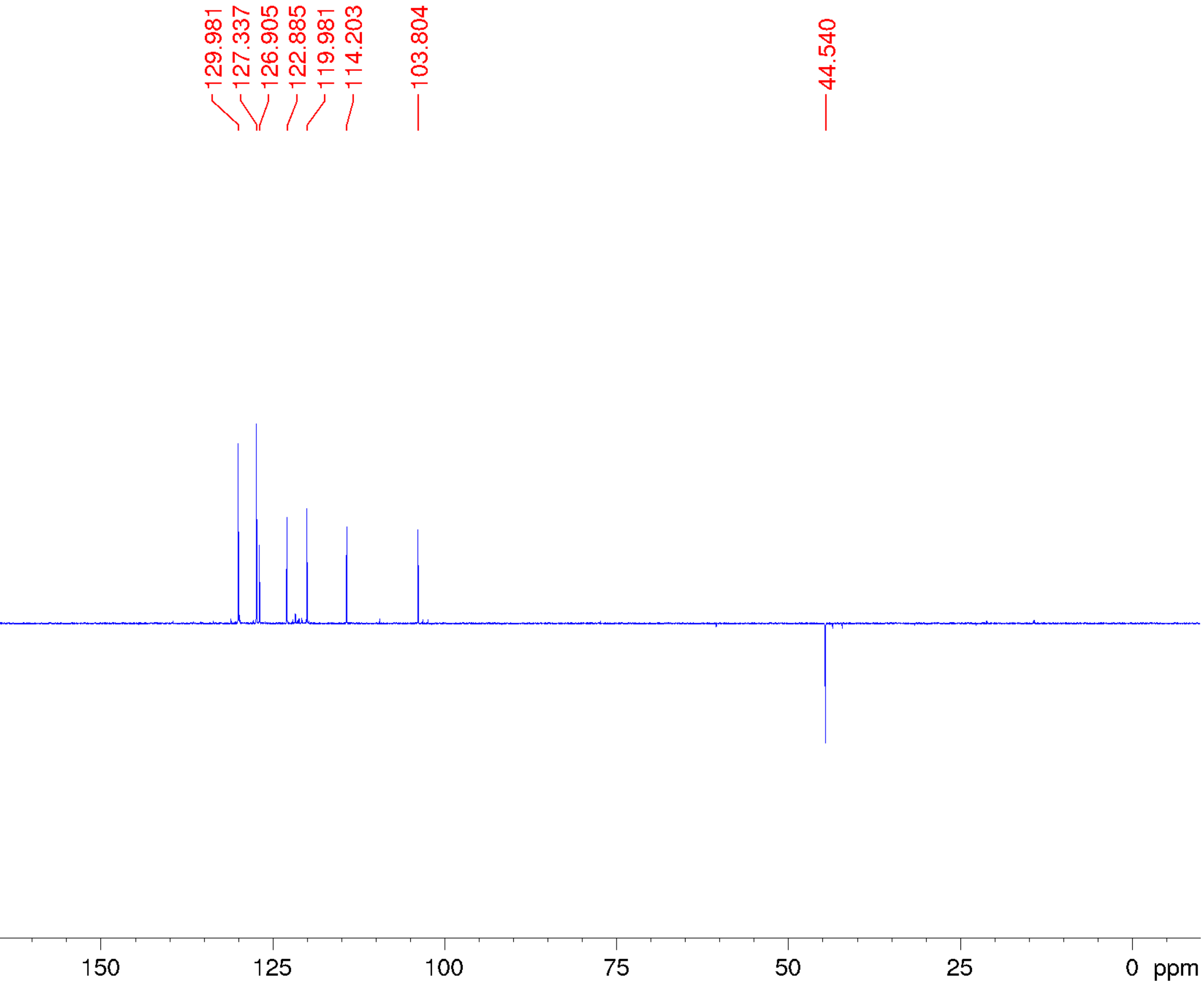

^{1}H NMR-spectrum (400 MHz, $CDCl_3$)

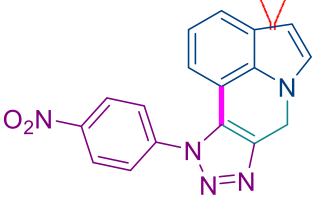

1f

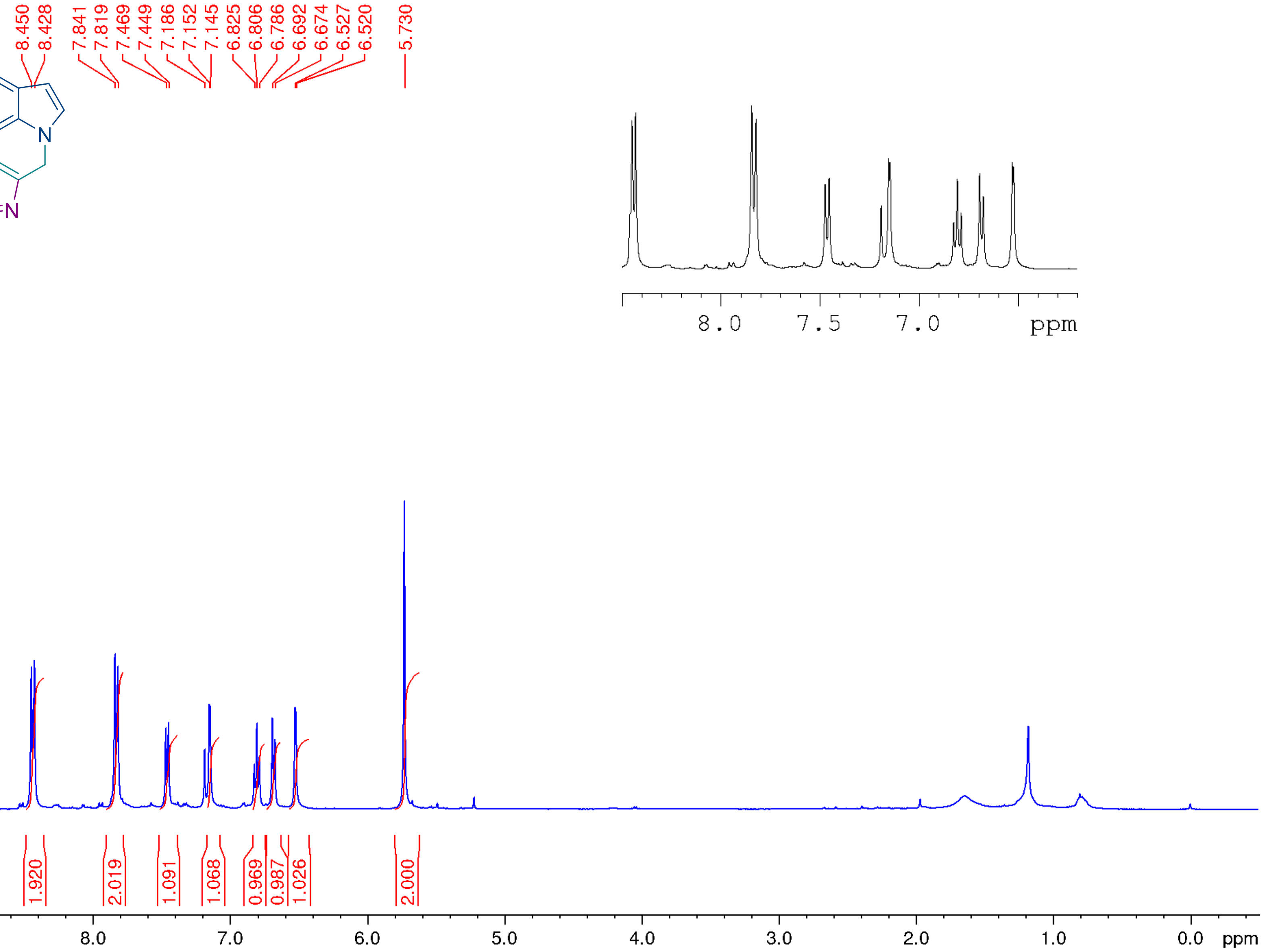

^{13}C NMR-spectrum (100 MHz, $CDCl_3$)

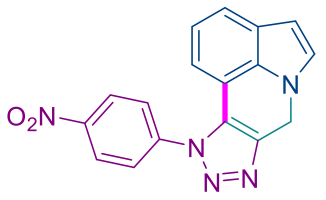

1f

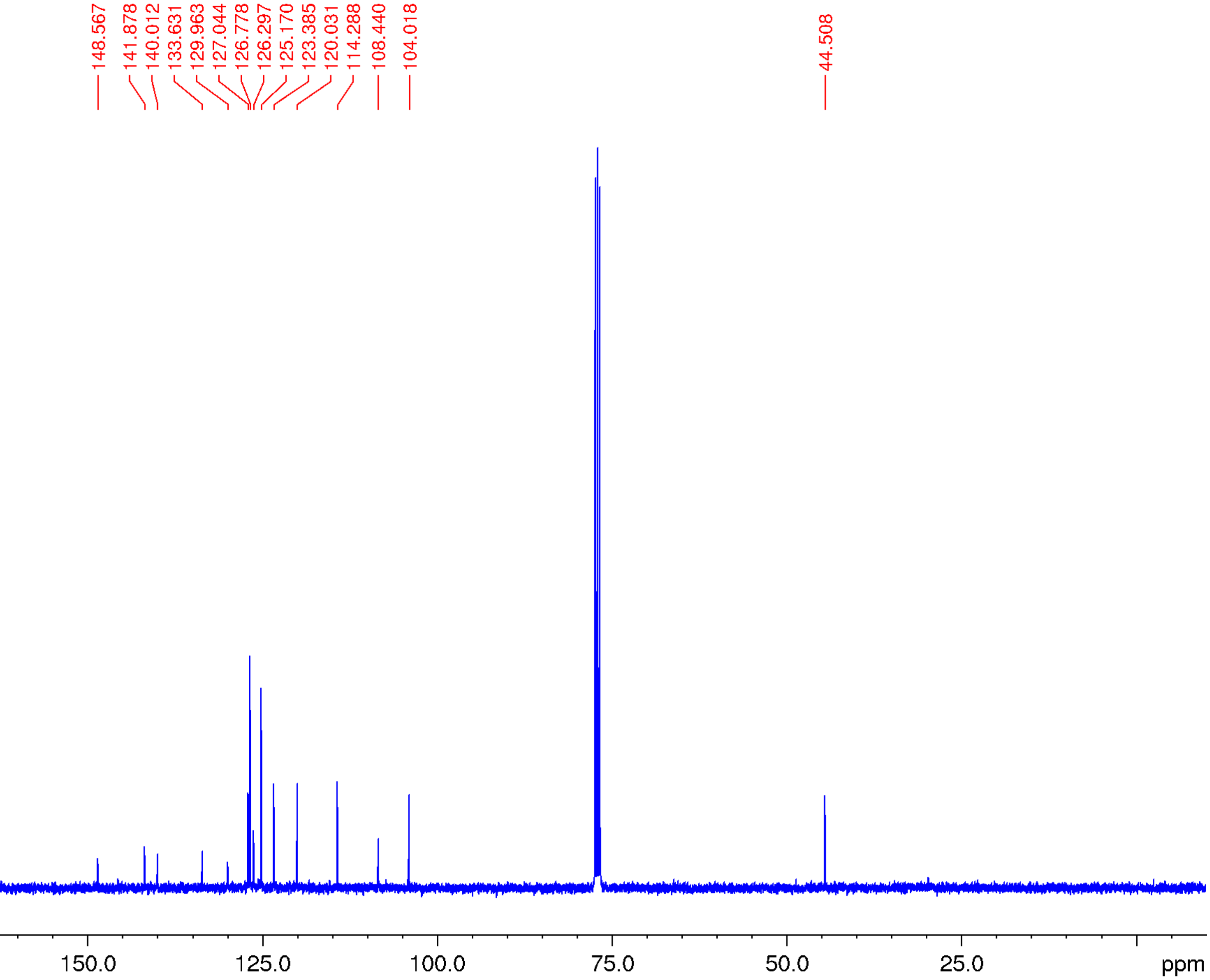

DEPT 135 NMR-spectrum ($CDCl_3$)

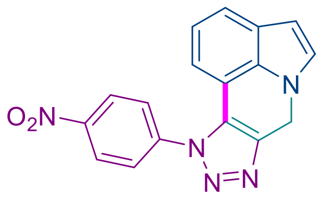

1f

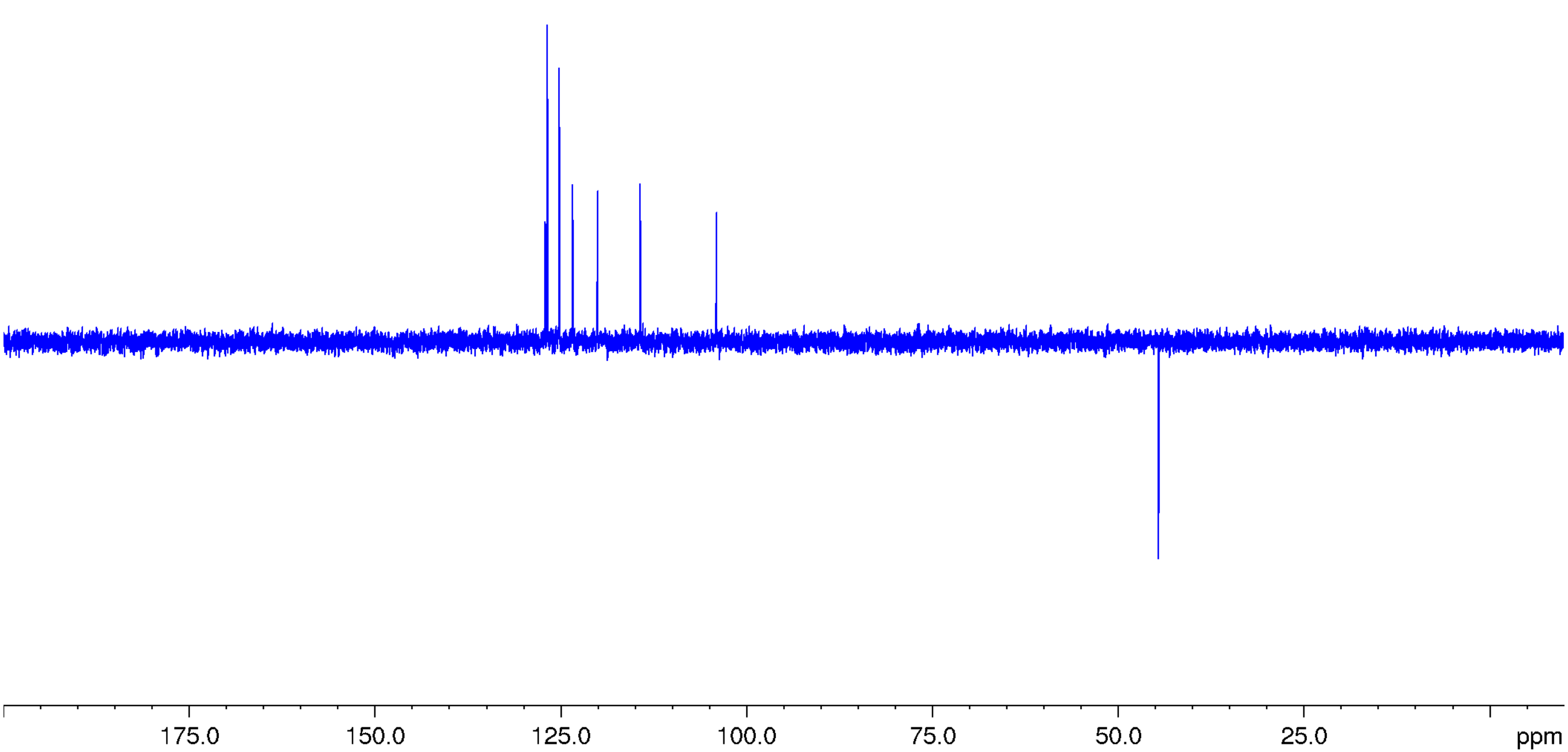

^{1}H NMR-spectrum (400 MHz, $CDCl_3$)

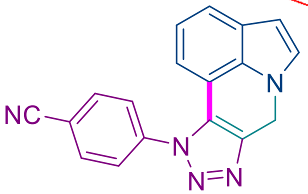

1g

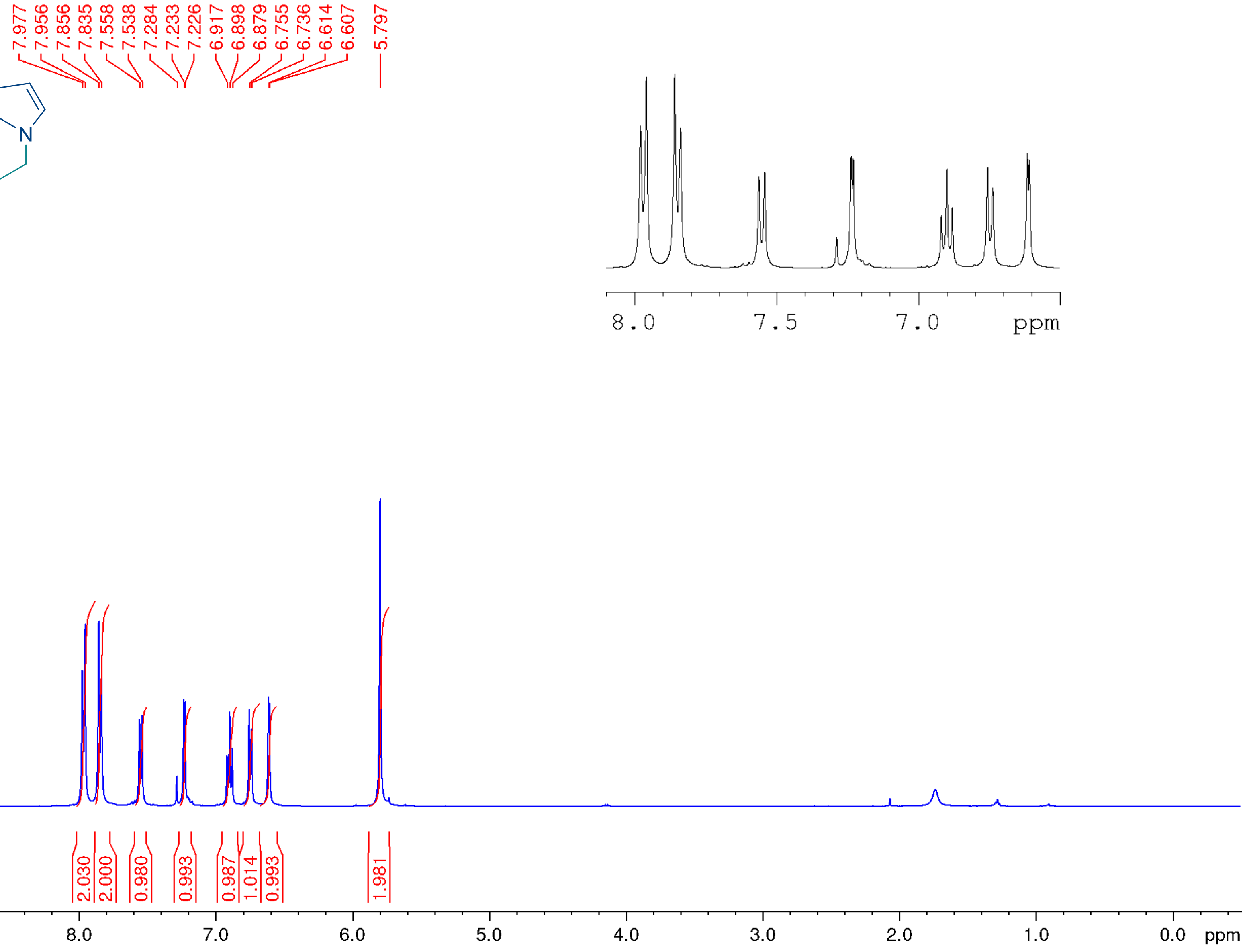

^{13}C NMR-spectrum (100 MHz, $CDCl_3$)

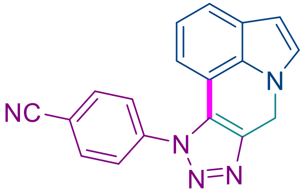

1g

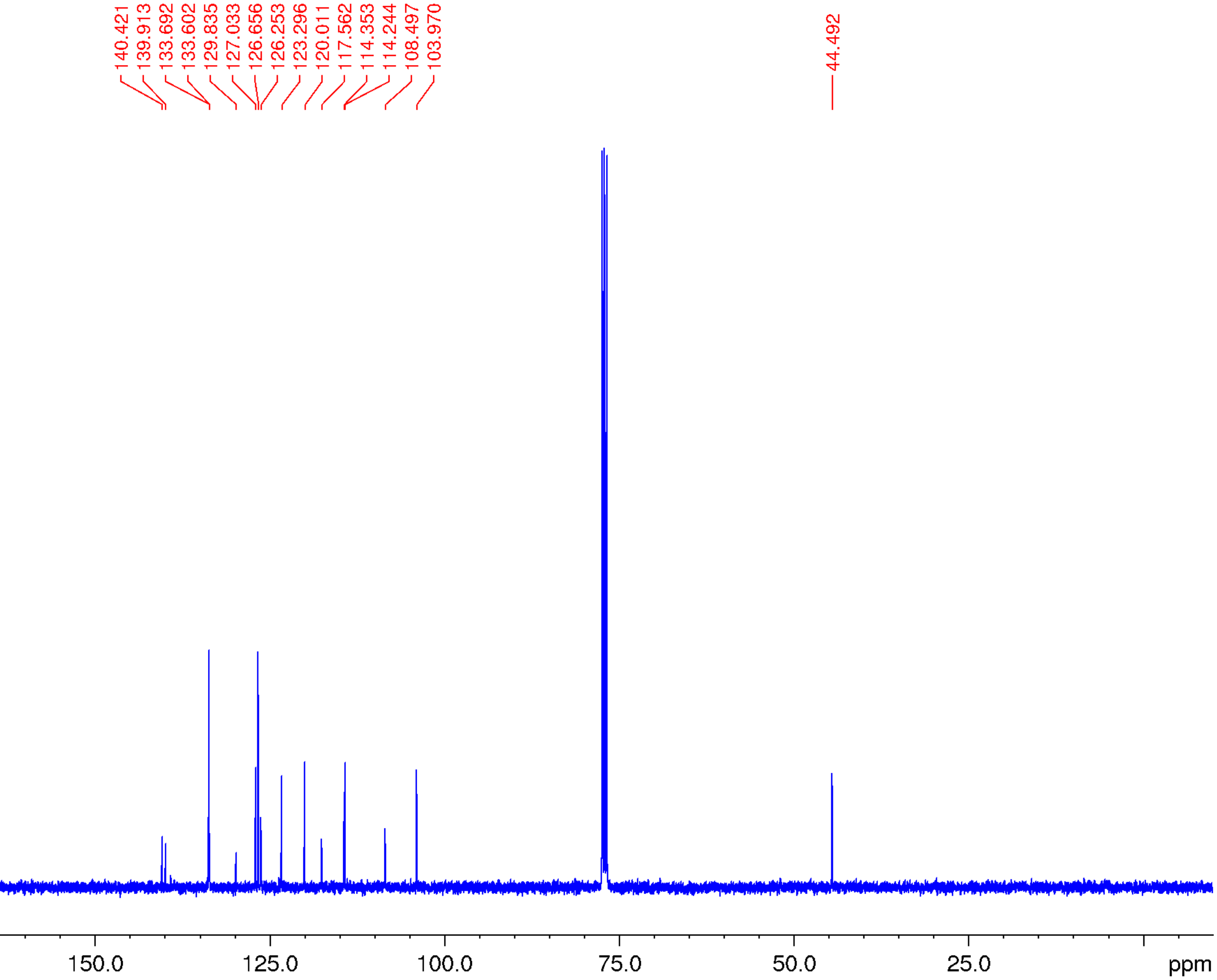

DEPT 135 NMR-spectrum ($CDCl_3$)

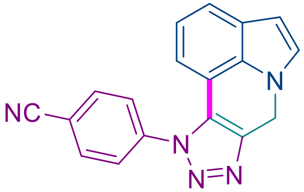

1g

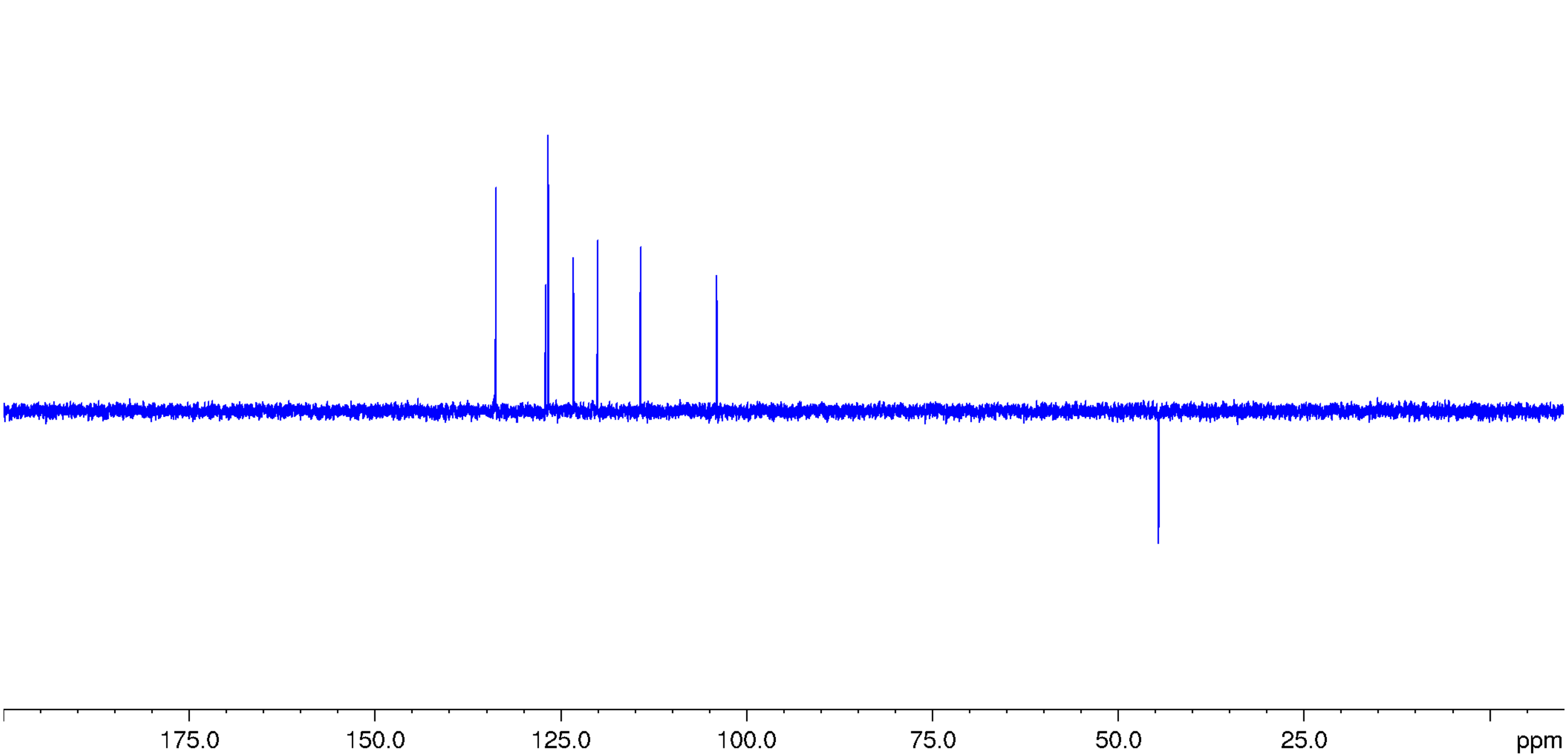

^{1}H NMR-spectrum (400 MHz, $CDCl_3$)

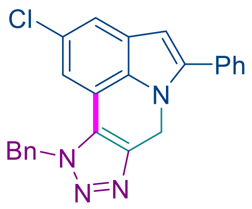

2a

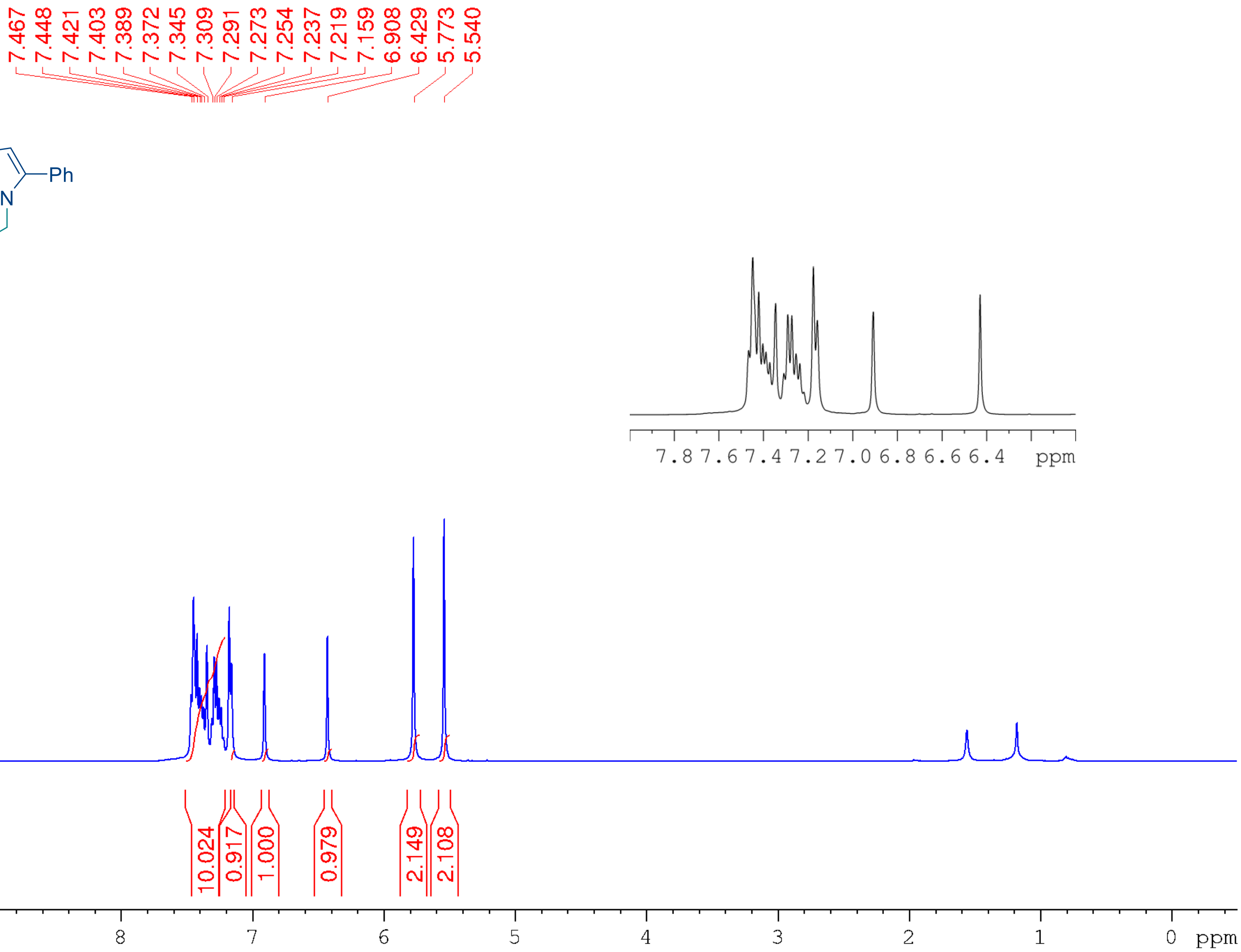

^{13}C NMR-spectrum (100 MHz, $CDCl_3$)

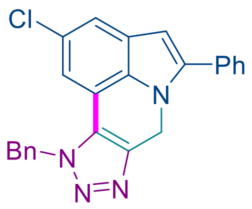

2a

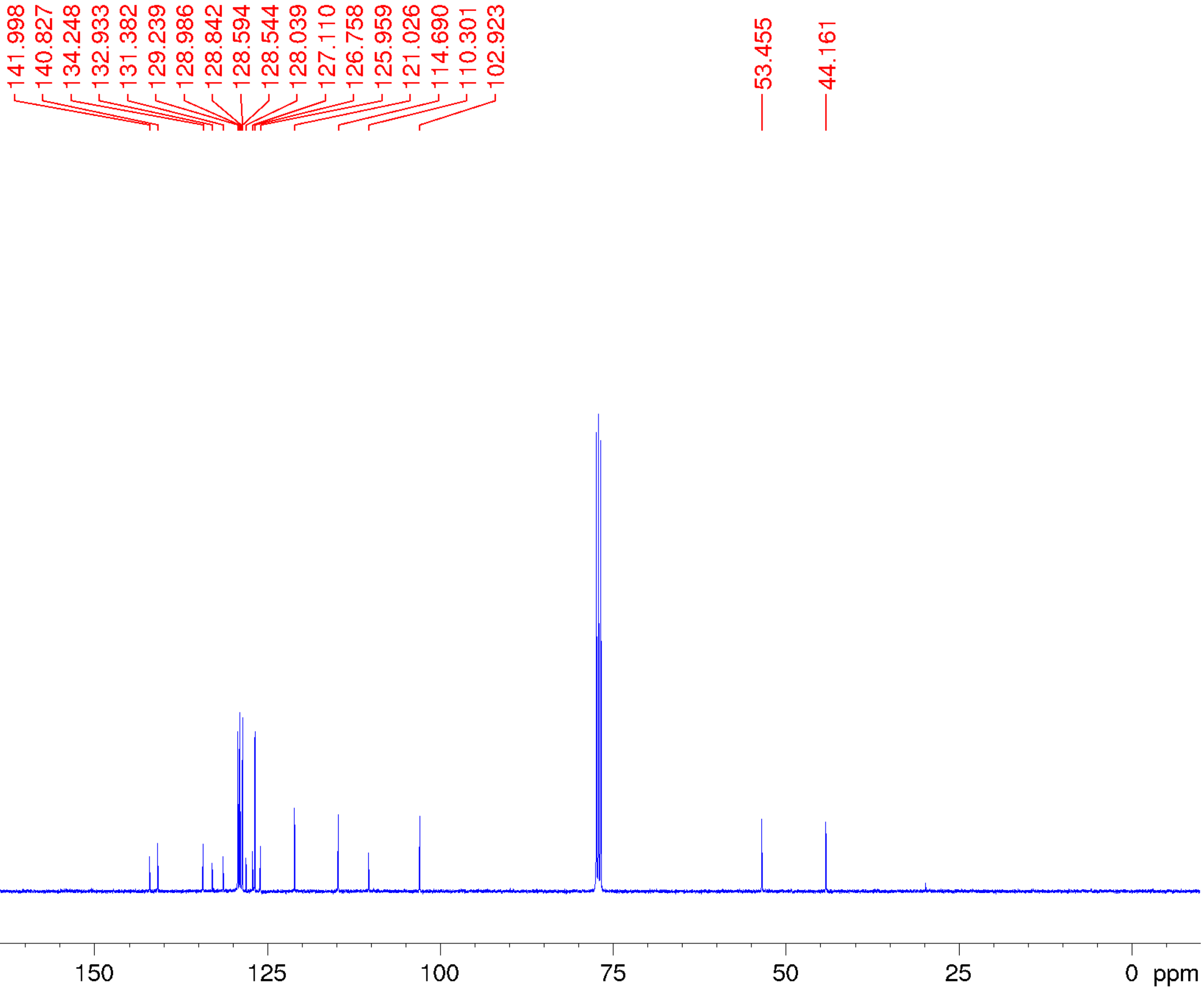

DEPT 135 NMR-spectrum ($CDCl_3$)

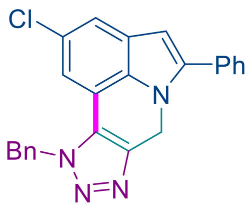

2a

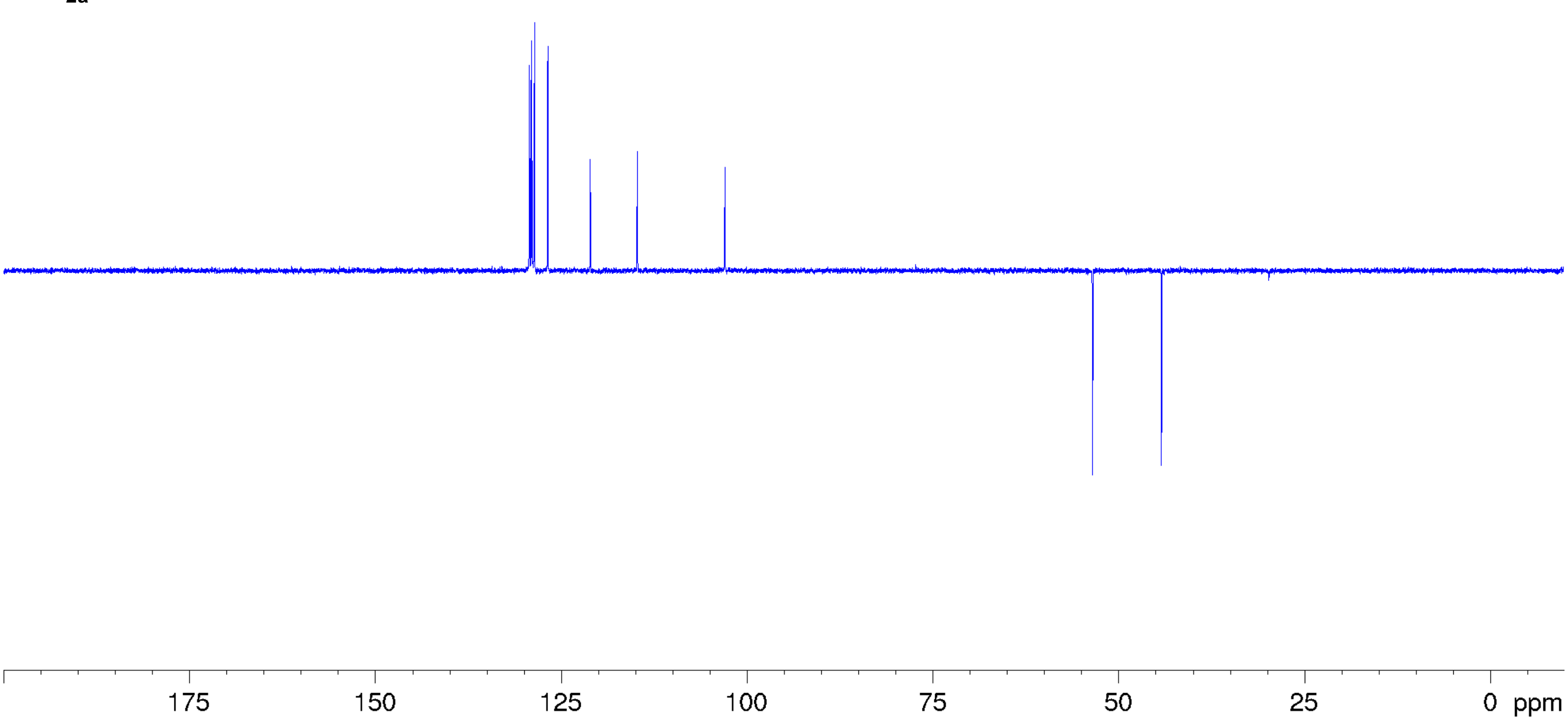

^{1}H NMR-spectrum (400 MHz, $CDCl_3$)

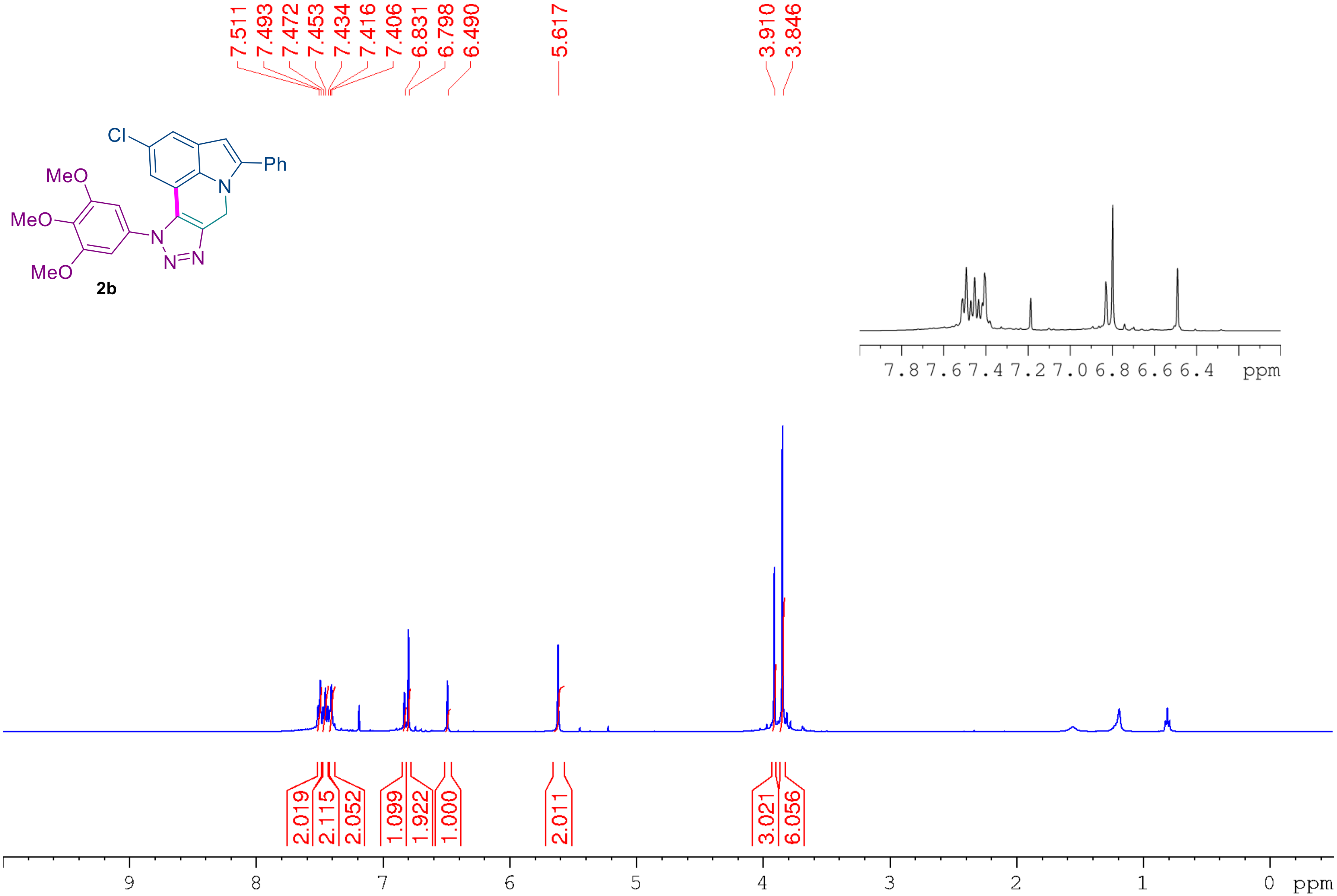

2b

^{13}C NMR-spectrum (100 MHz, $CDCl_3$)

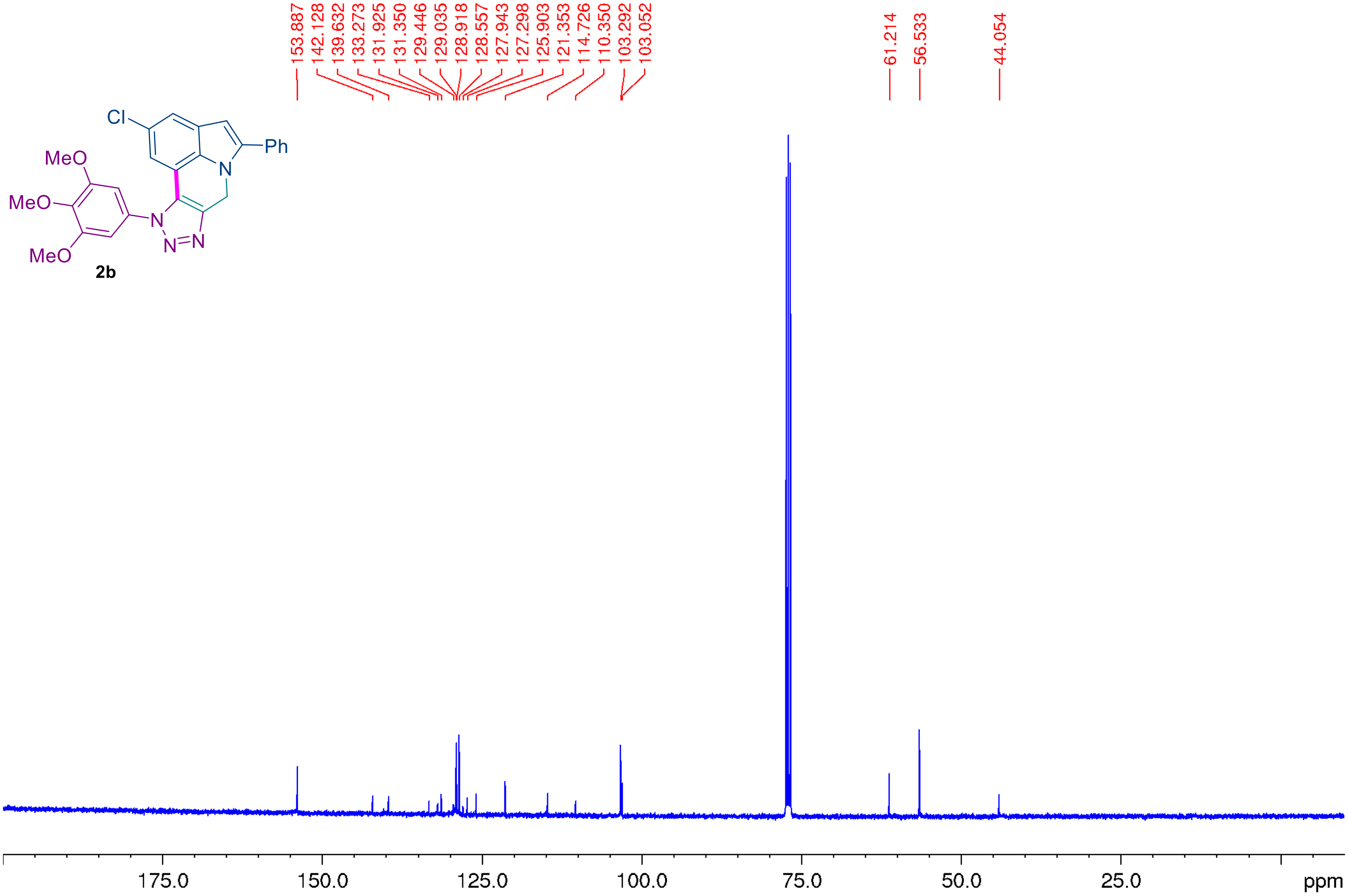

DEPT 135 NMR-spectrum ($CDCl_3$)

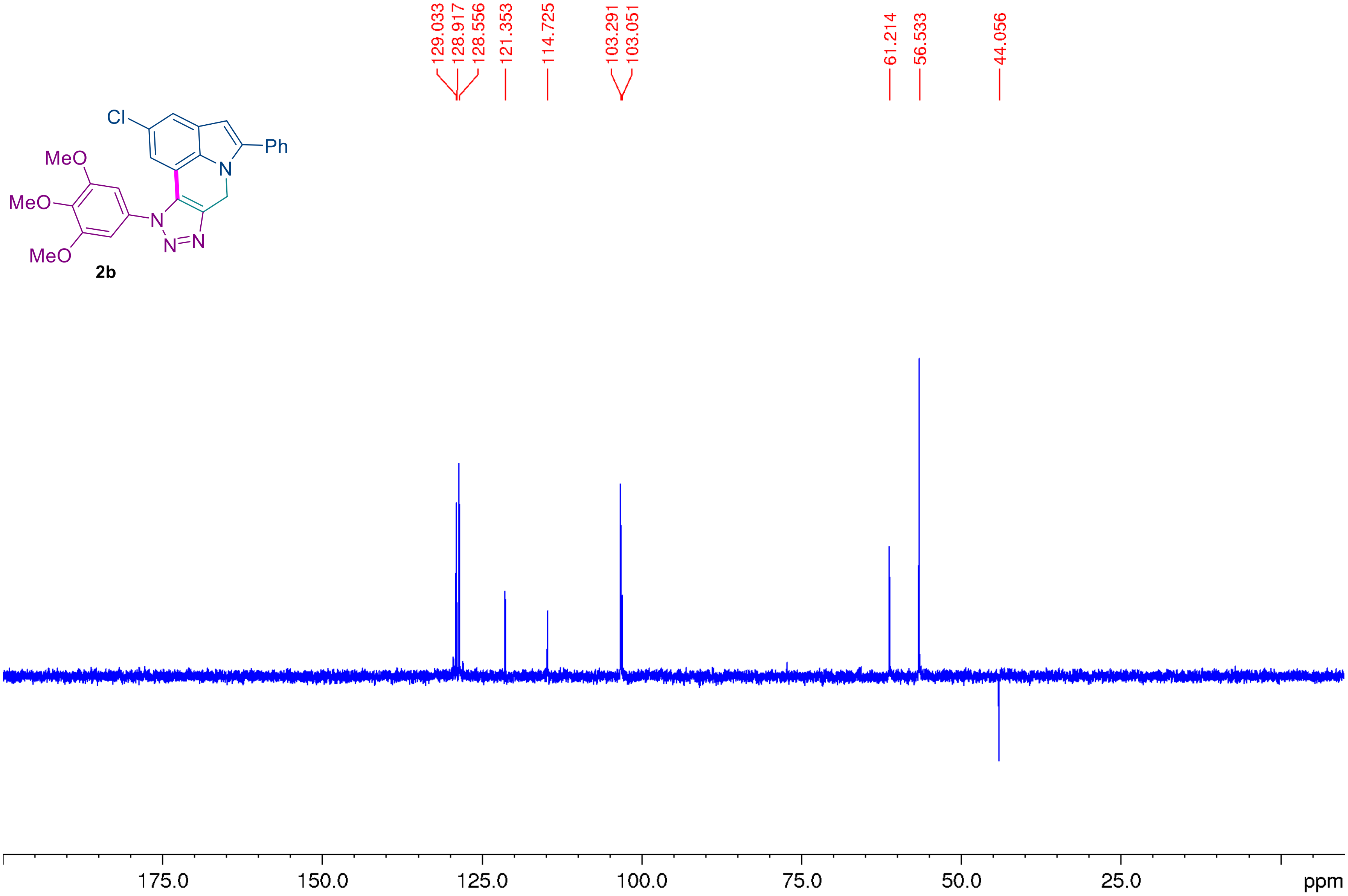

^{1}H NMR-spectrum (400 MHz, $CDCl_3$)

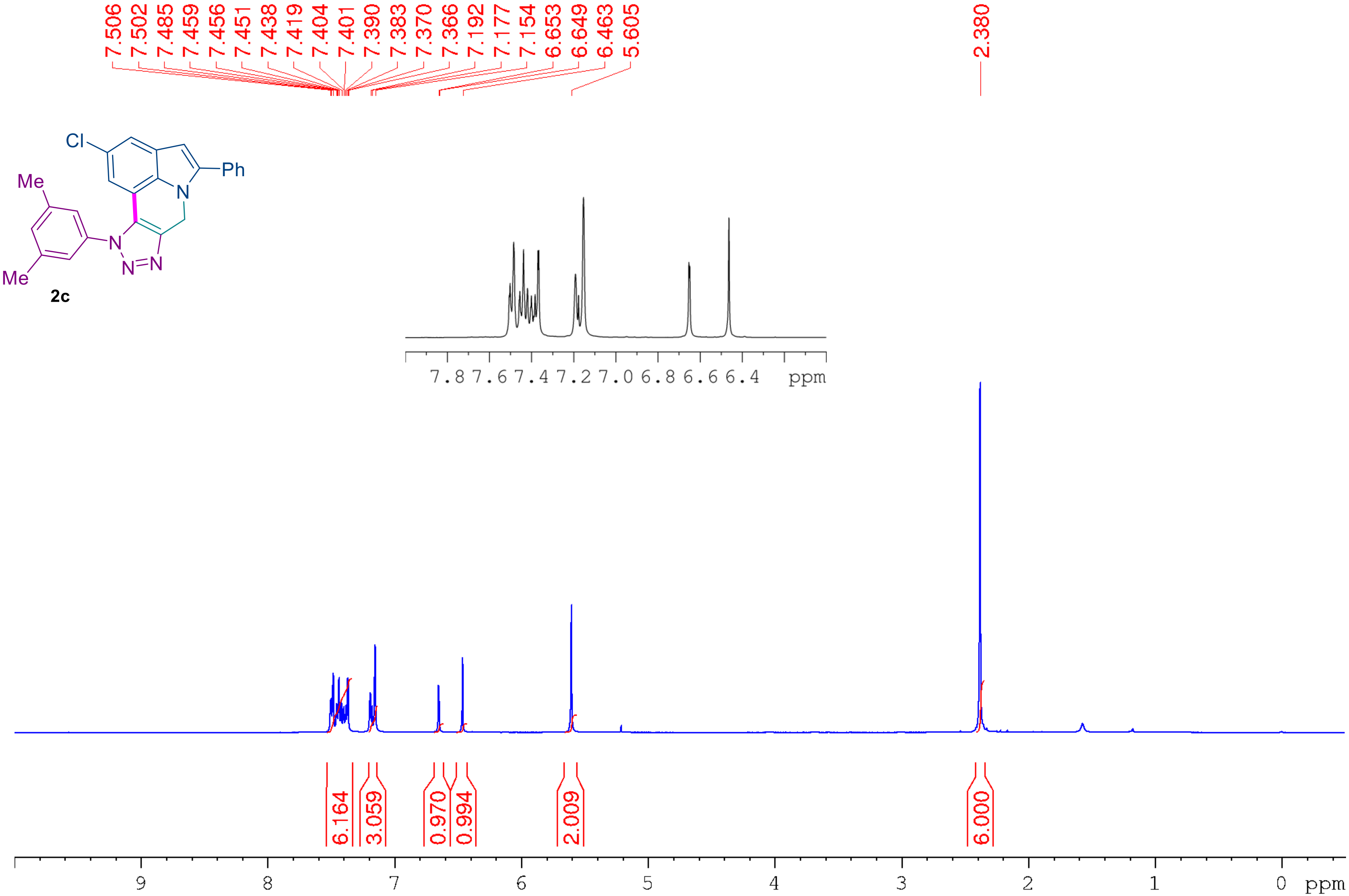

^{13}C NMR-spectrum (100 MHz, $CDCl_3$)

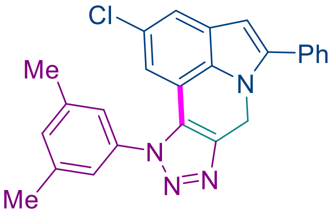

2c

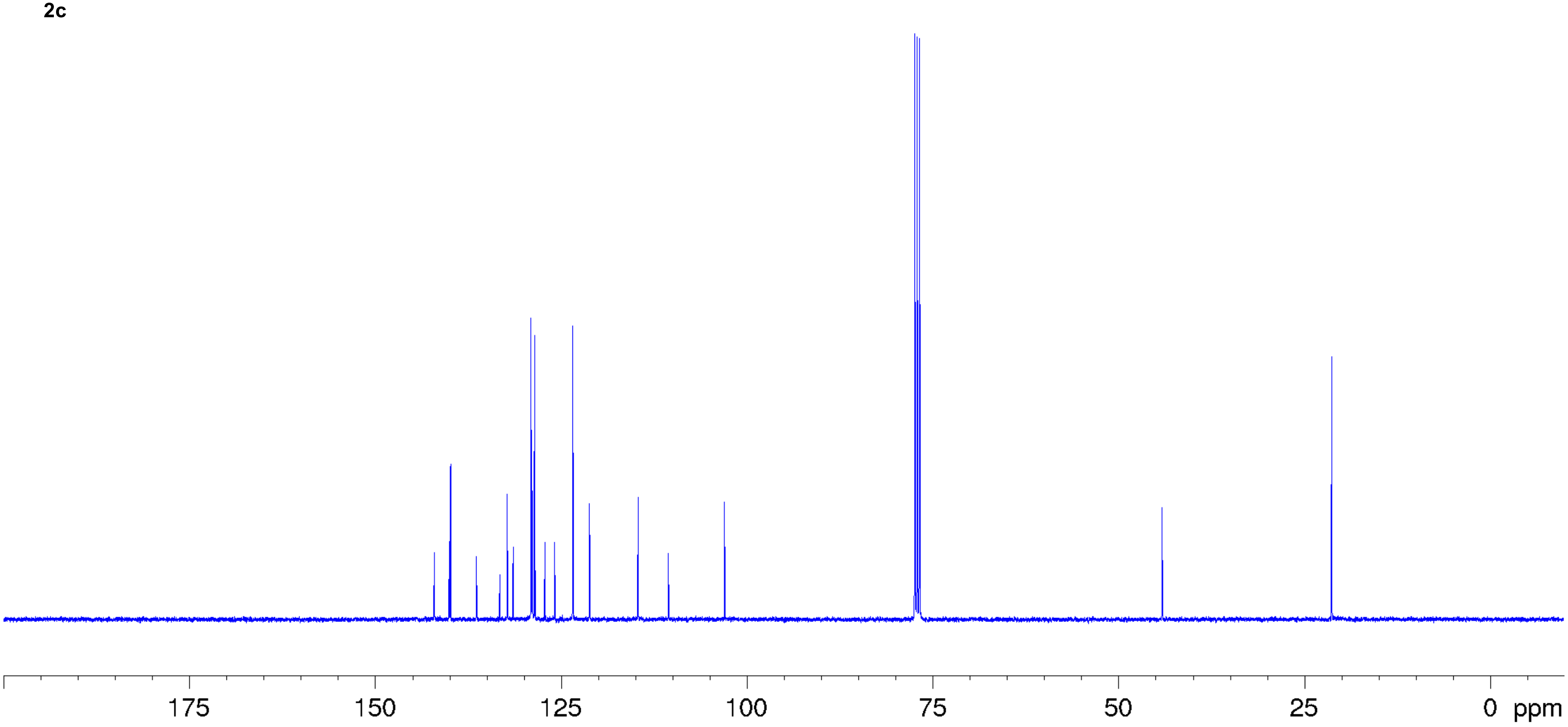

DEPT 135 NMR-spectrum ($CDCl_3$)

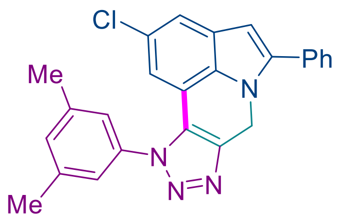

2c

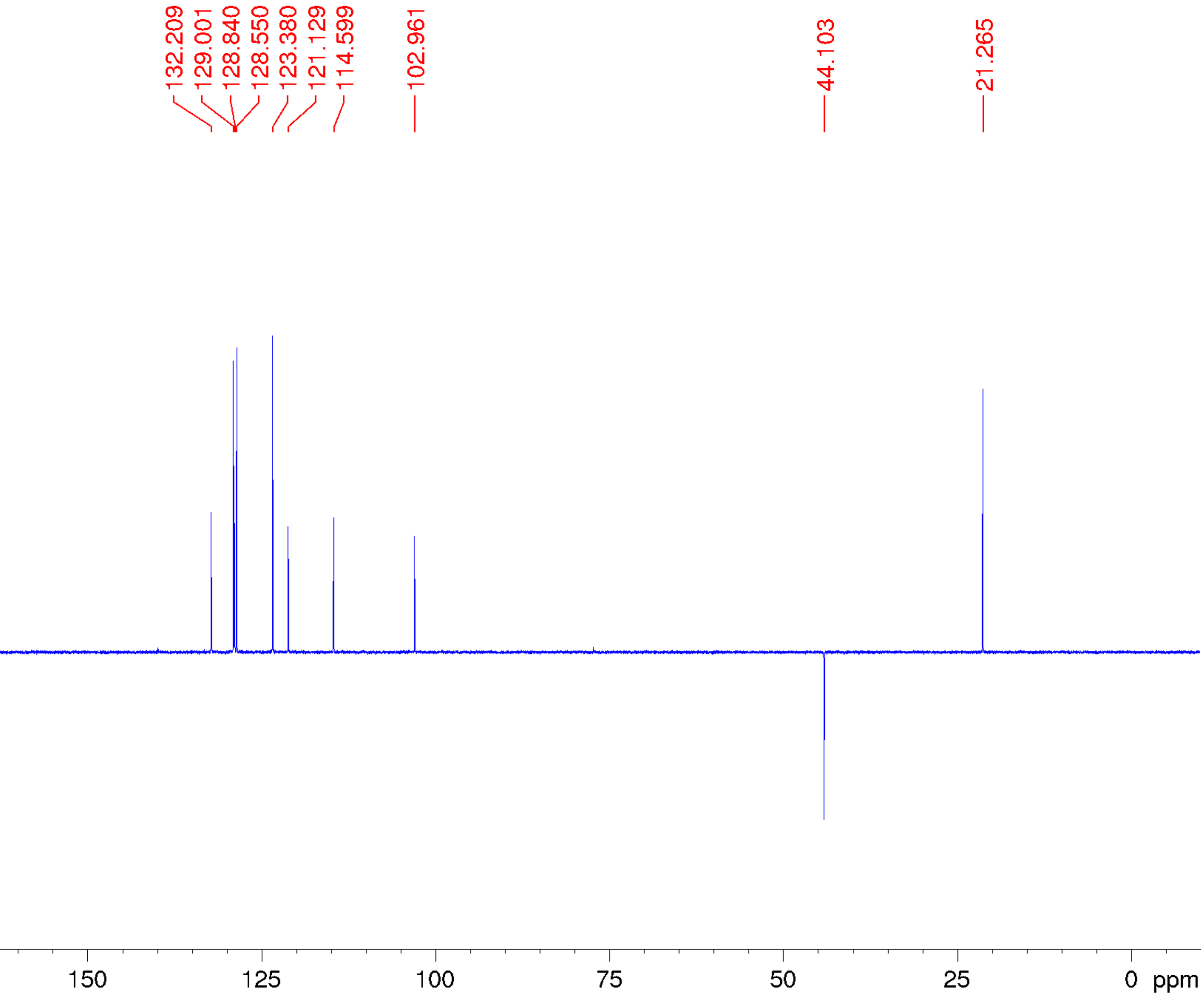

^{1}H NMR-spectrum (400 MHz, $CDCl_3$)

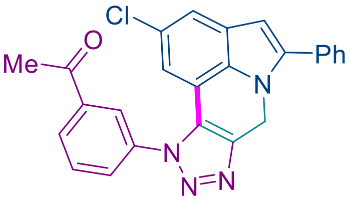

2d

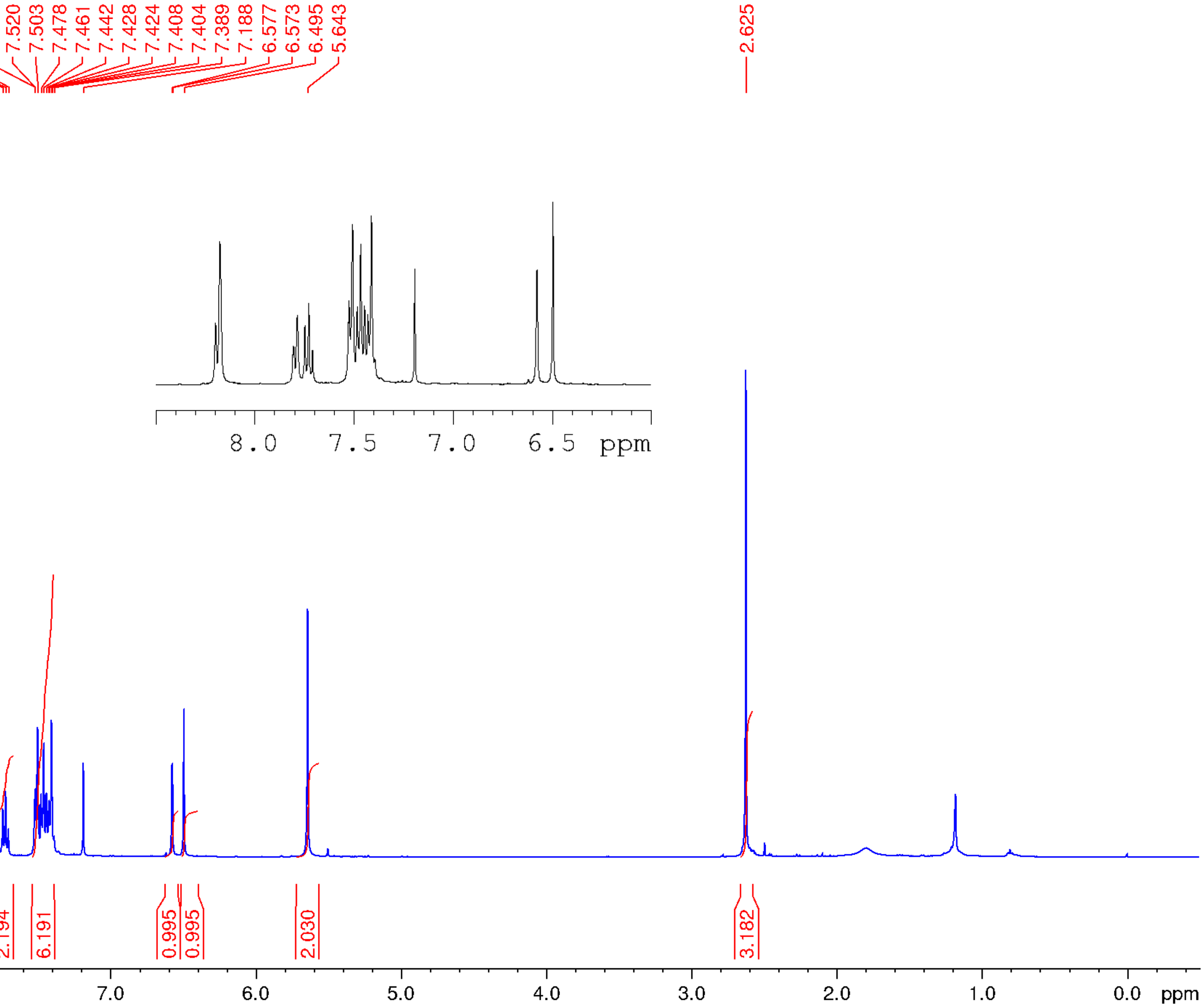

^{13}C NMR-spectrum (100 MHz, $CDCl_3$)

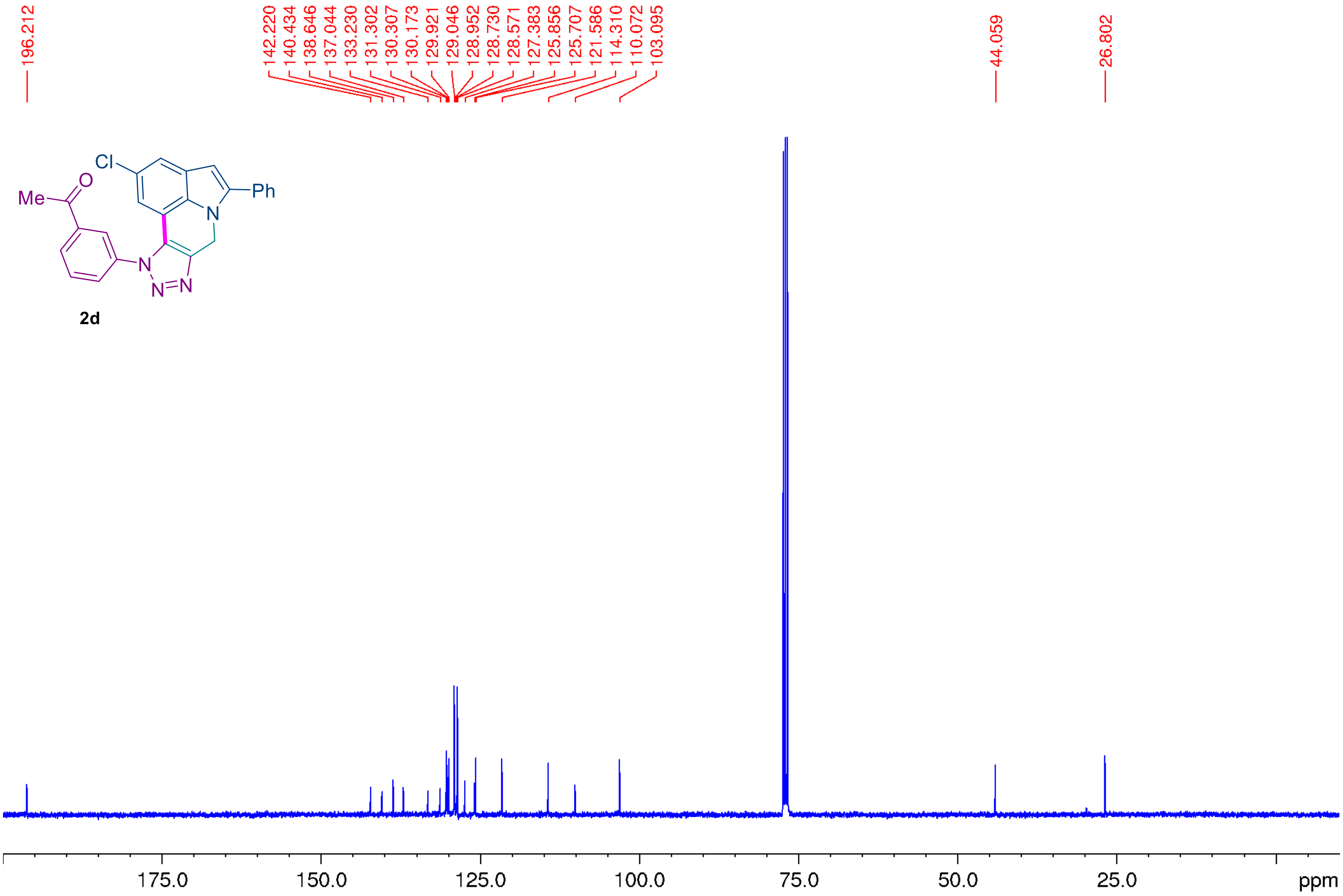

DEPT 135 NMR-spectrum ($CDCl_3$)

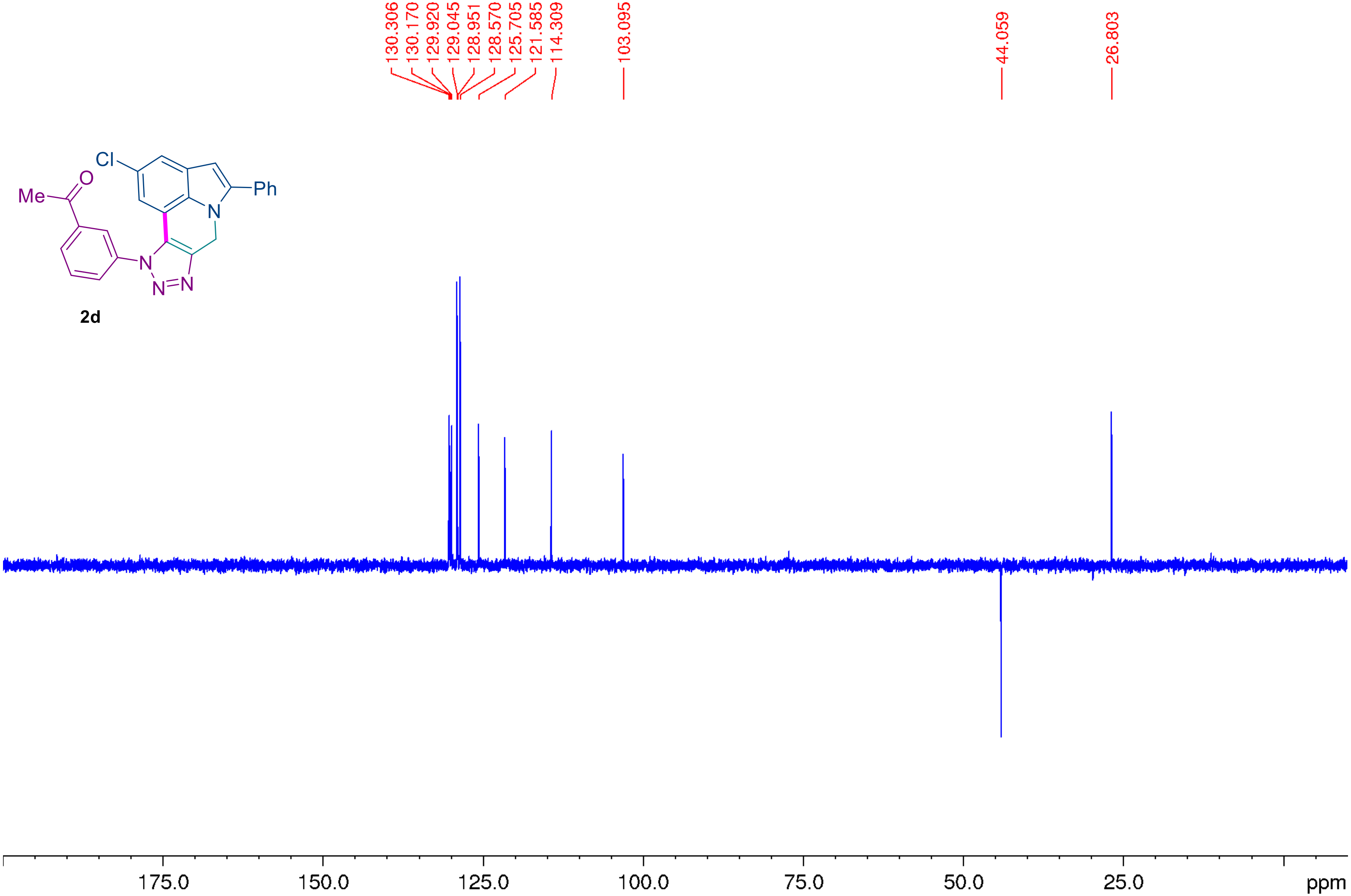

^1H NMR-spectrum (400 MHz, $CDCl_3$)

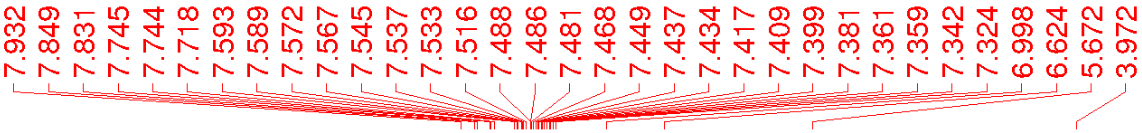

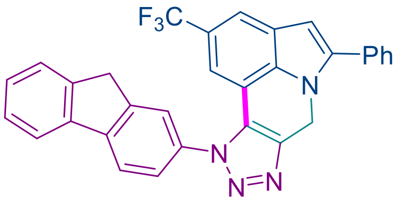

2e

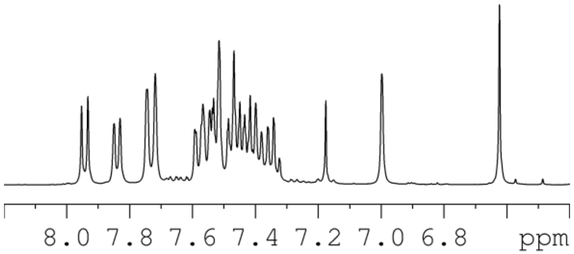

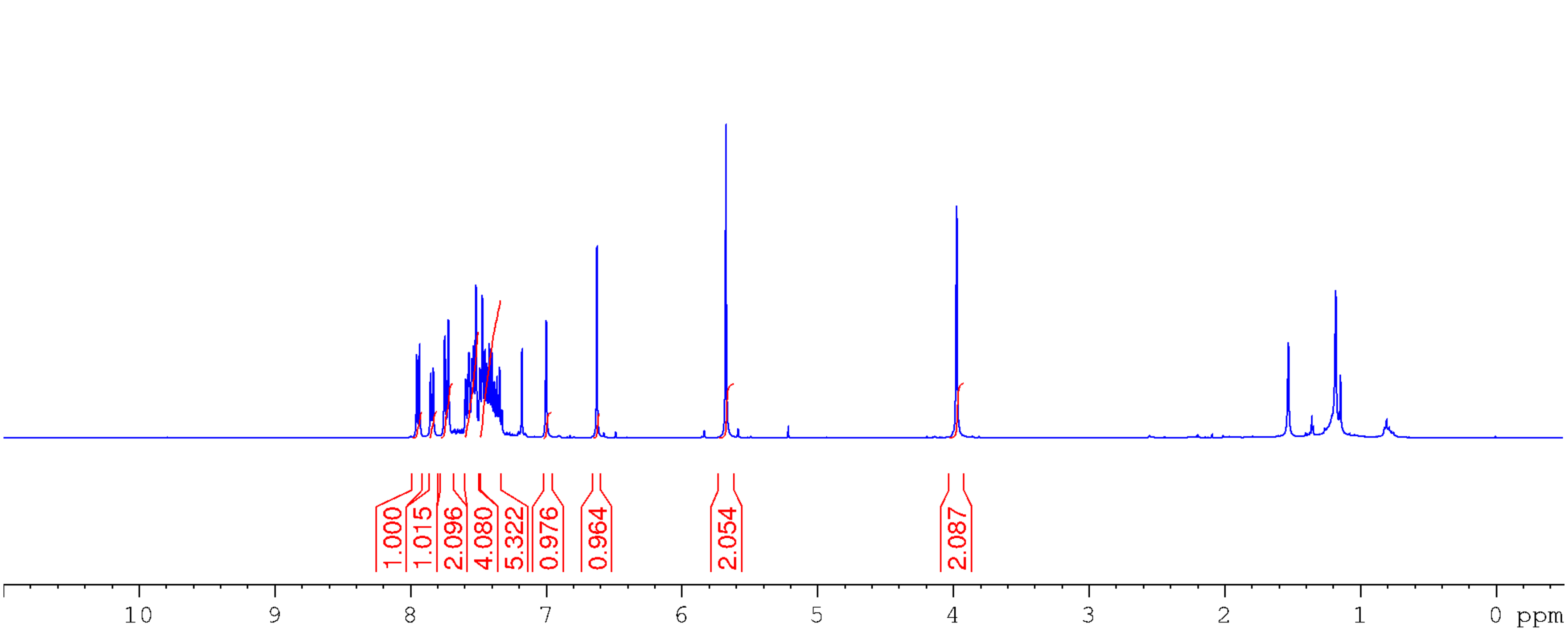

^{13}C NMR-spectrum (100 MHz, $CDCl_3$)

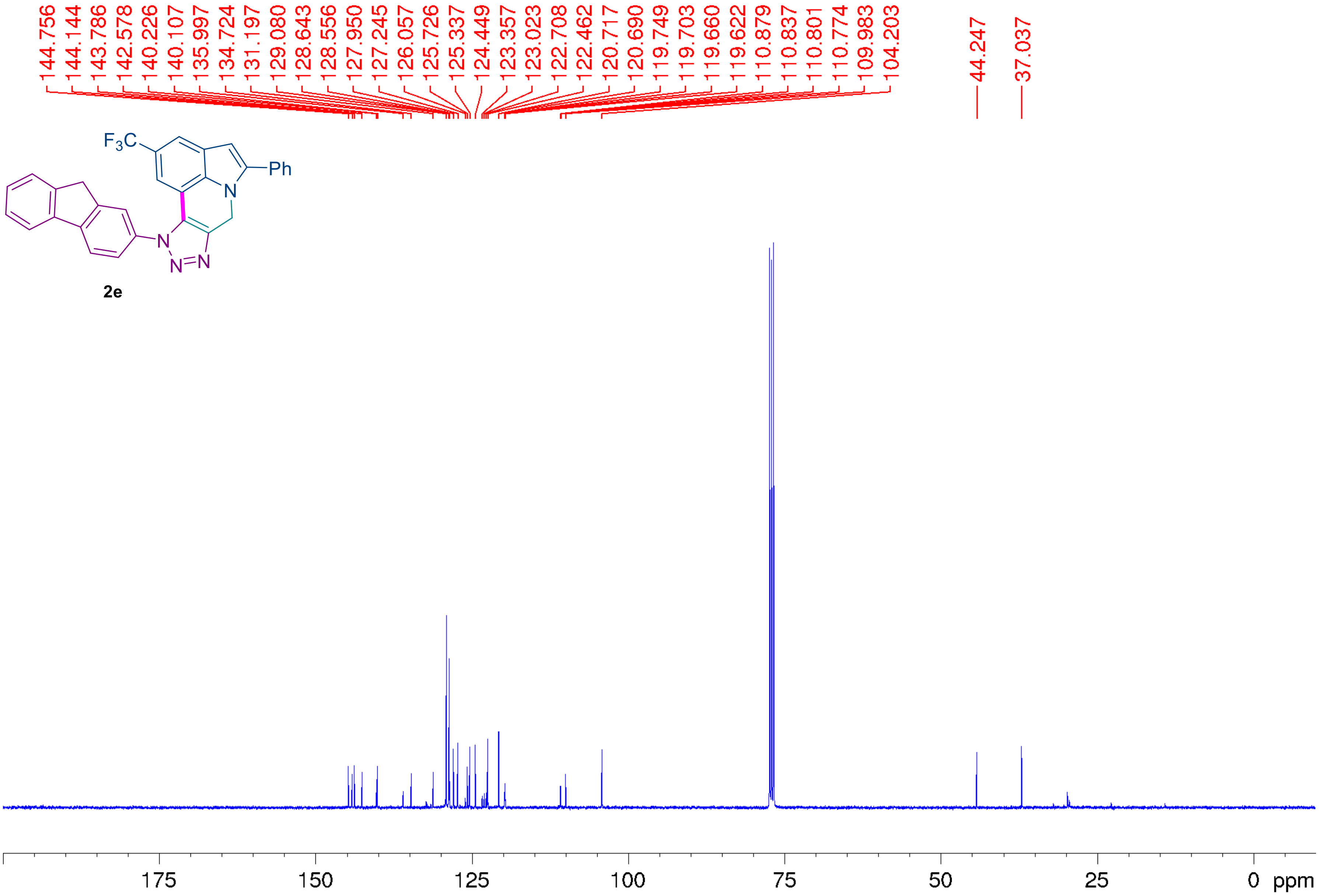

DEPT 135 NMR-spectrum ($CDCl_3$)

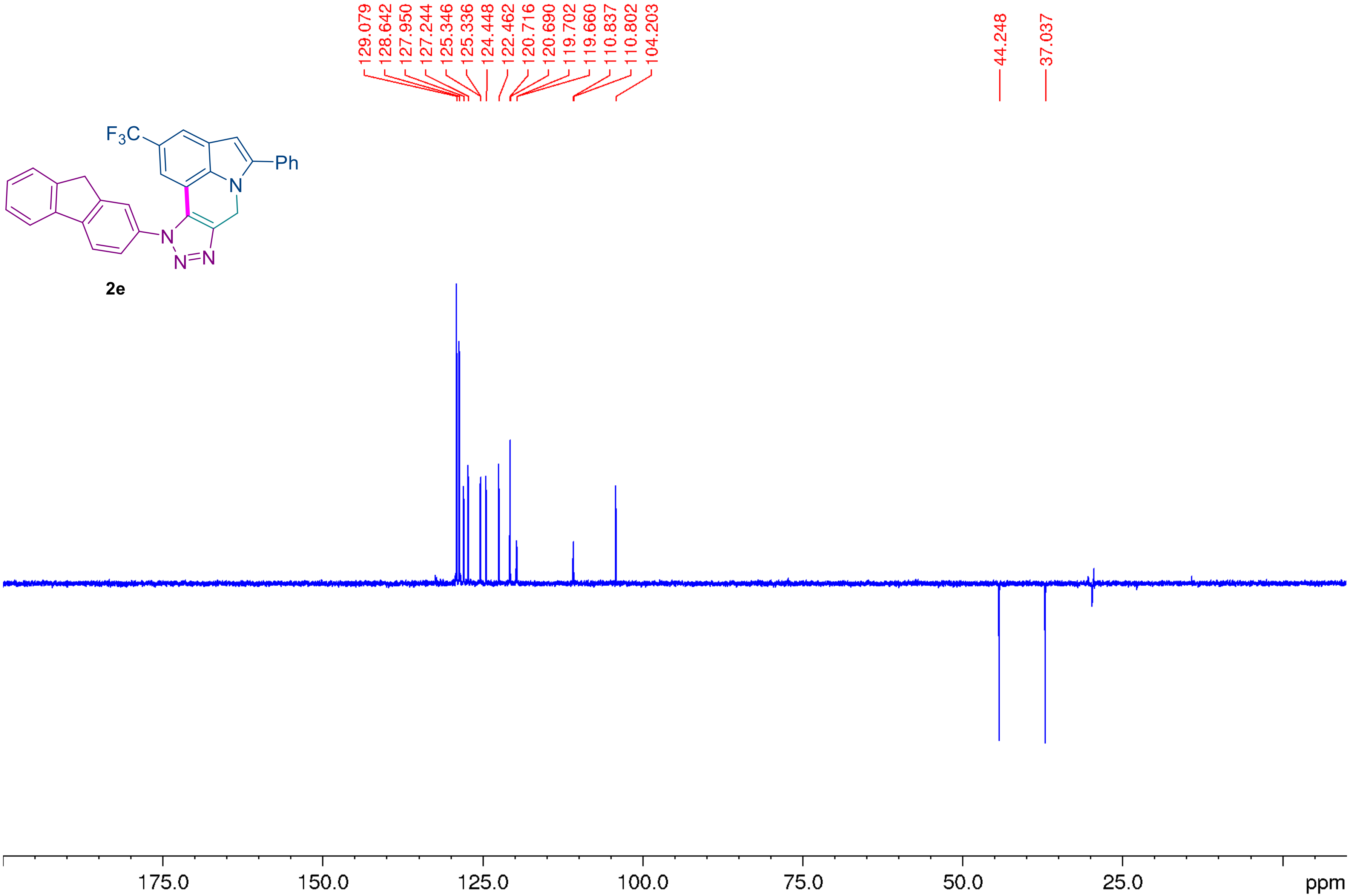

^{19}F NMR-spectrum (376.5 Hz, $CDCl_3$)

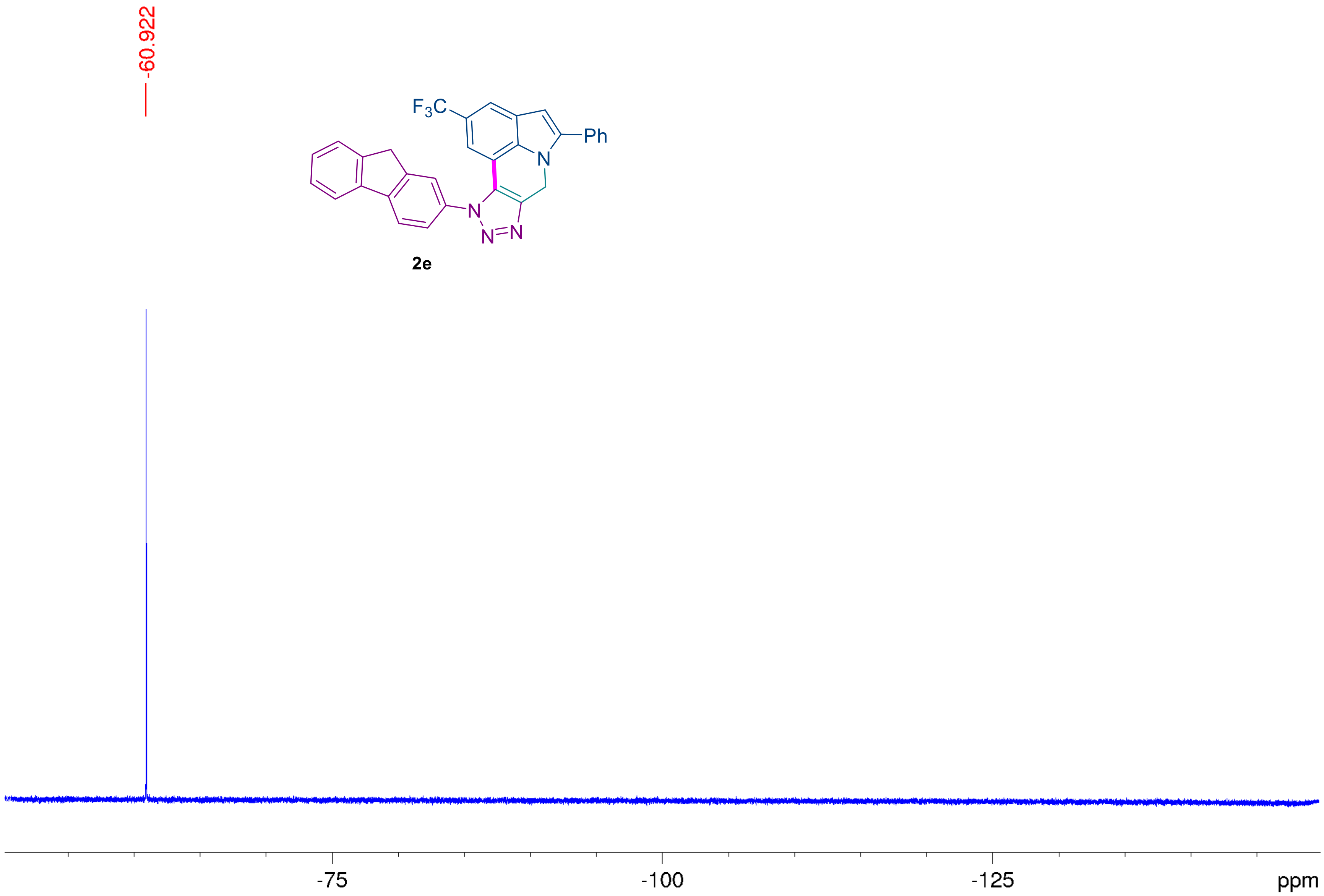

^1H NMR-spectrum (400 MHz, $CDCl_3$)

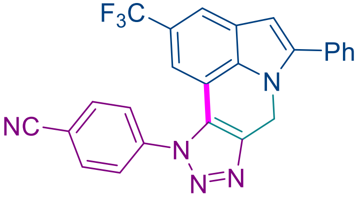

2f

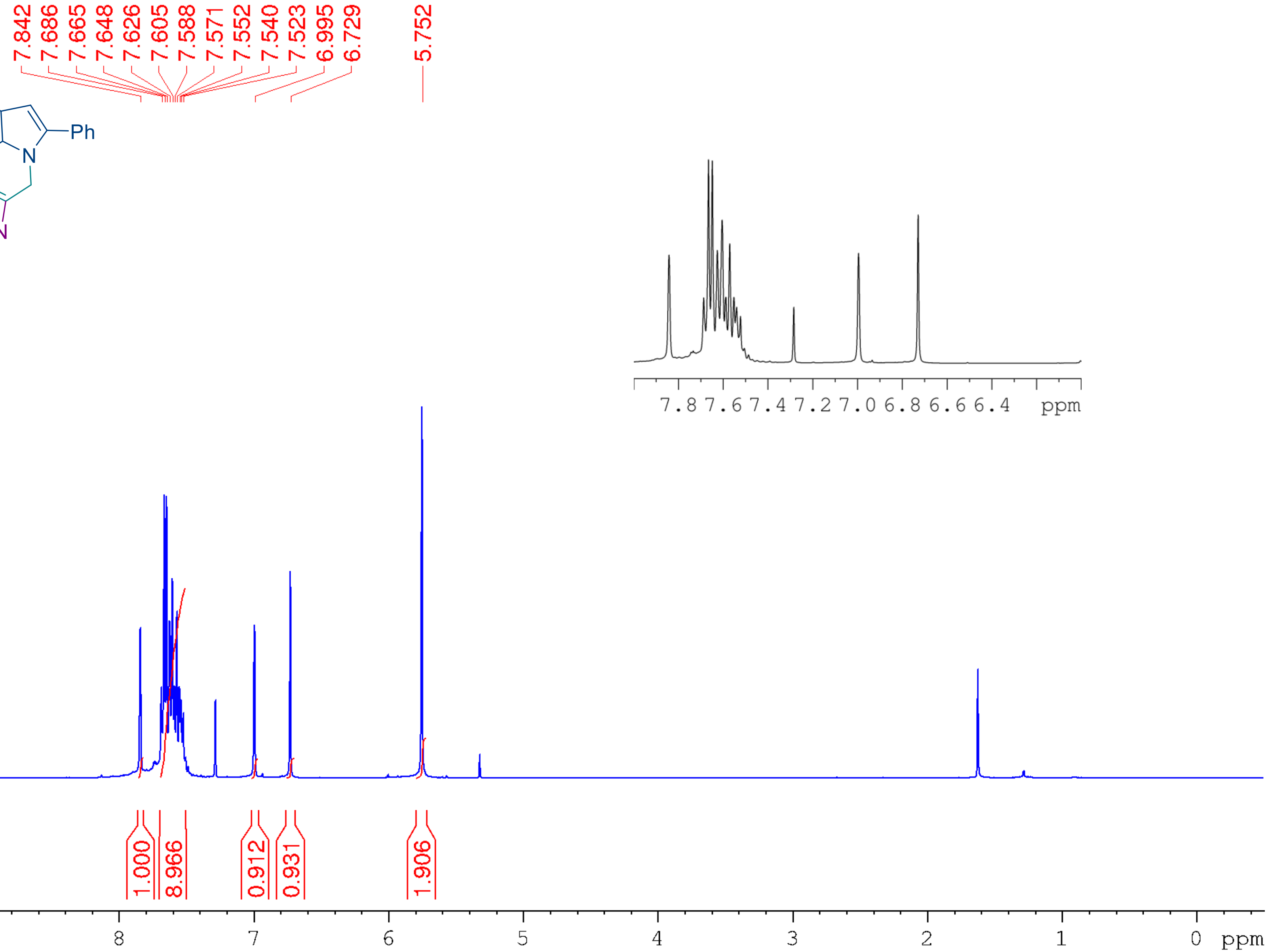

^{13}C NMR-spectrum (100 MHz, $CDCl_3$)

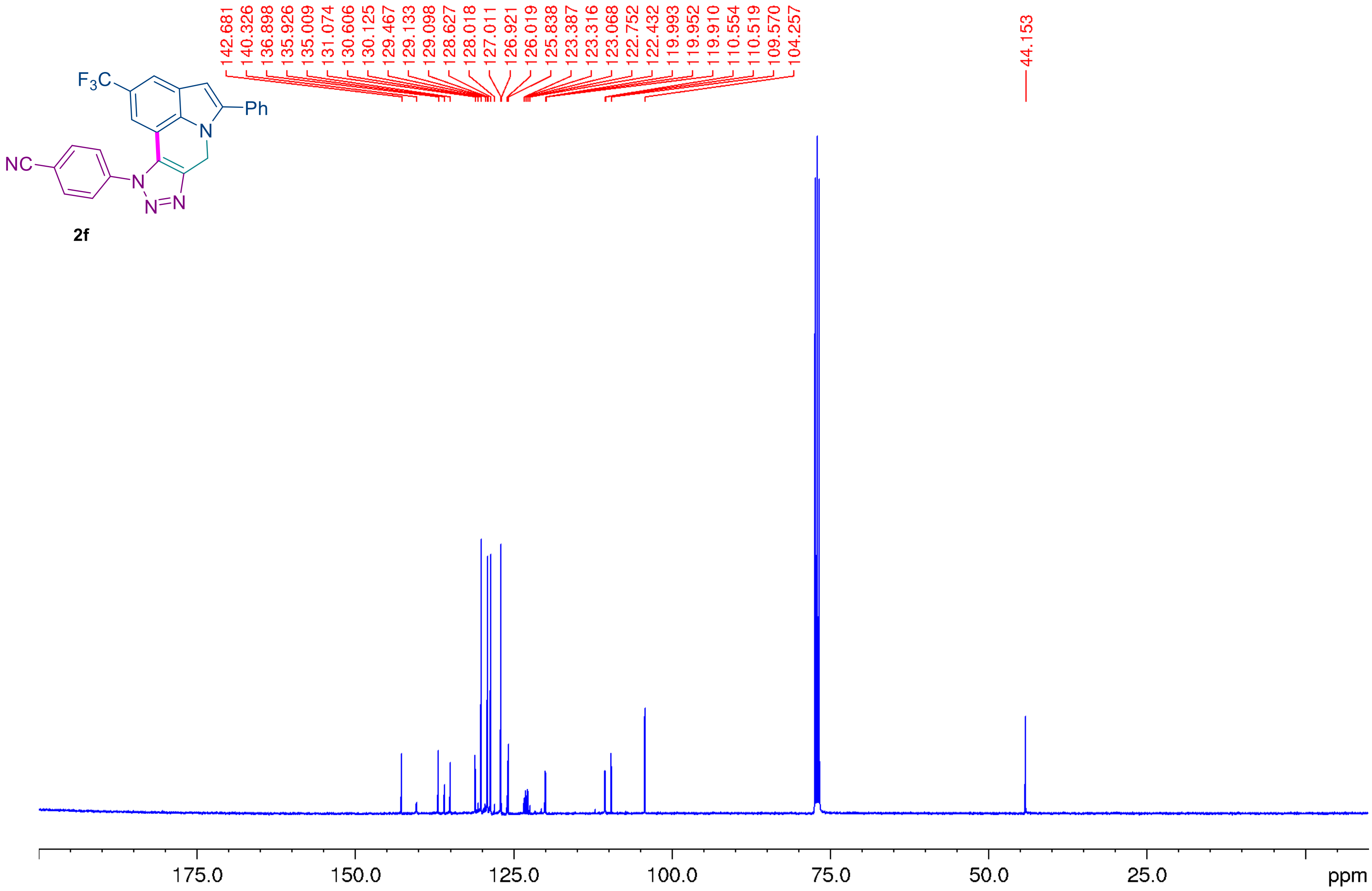

DEPT 135 NMR-spectrum ($CDCl_3$)

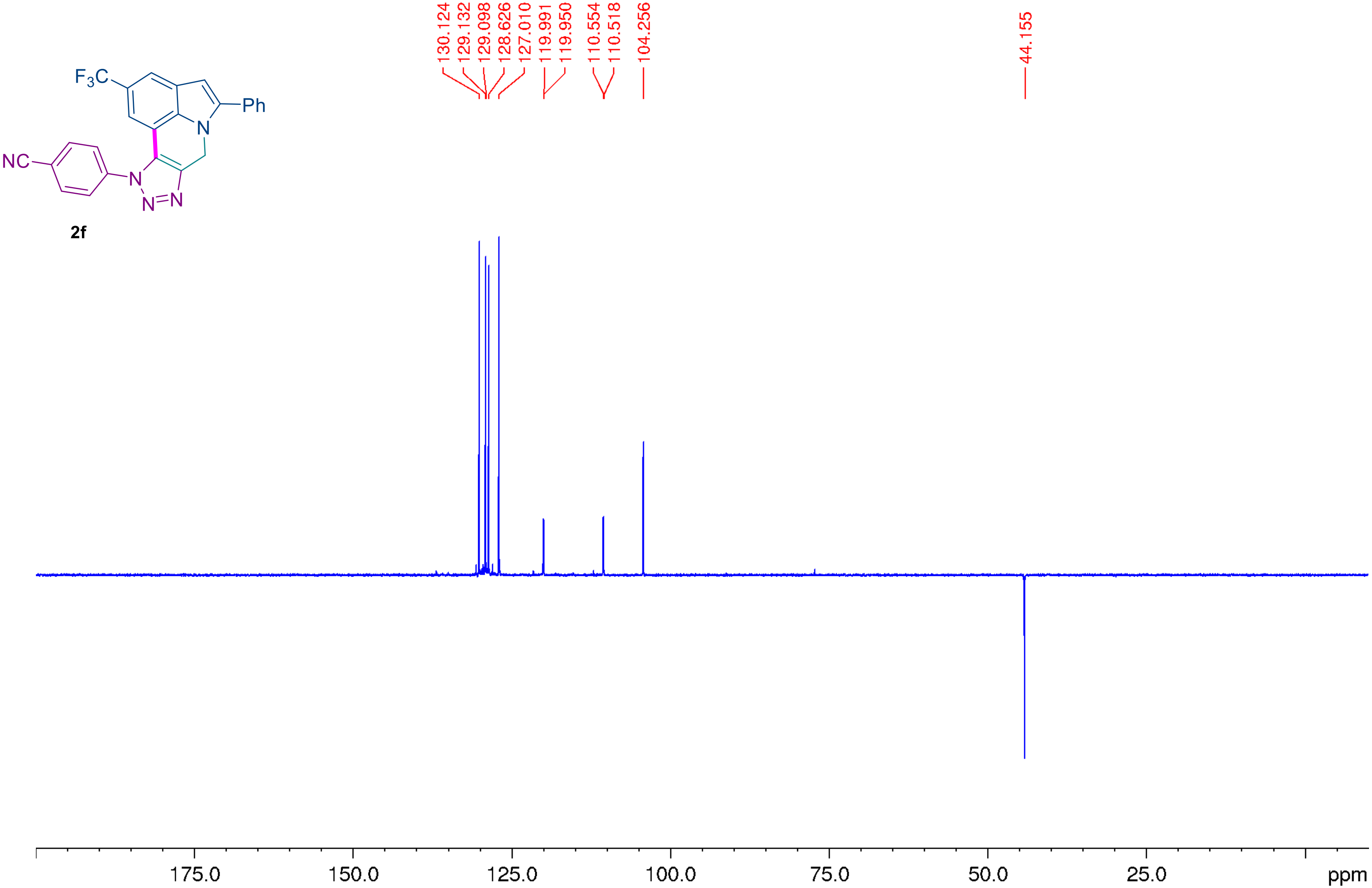

^{19}F NMR-spectrum (376.5 Hz, $CDCl_3$)

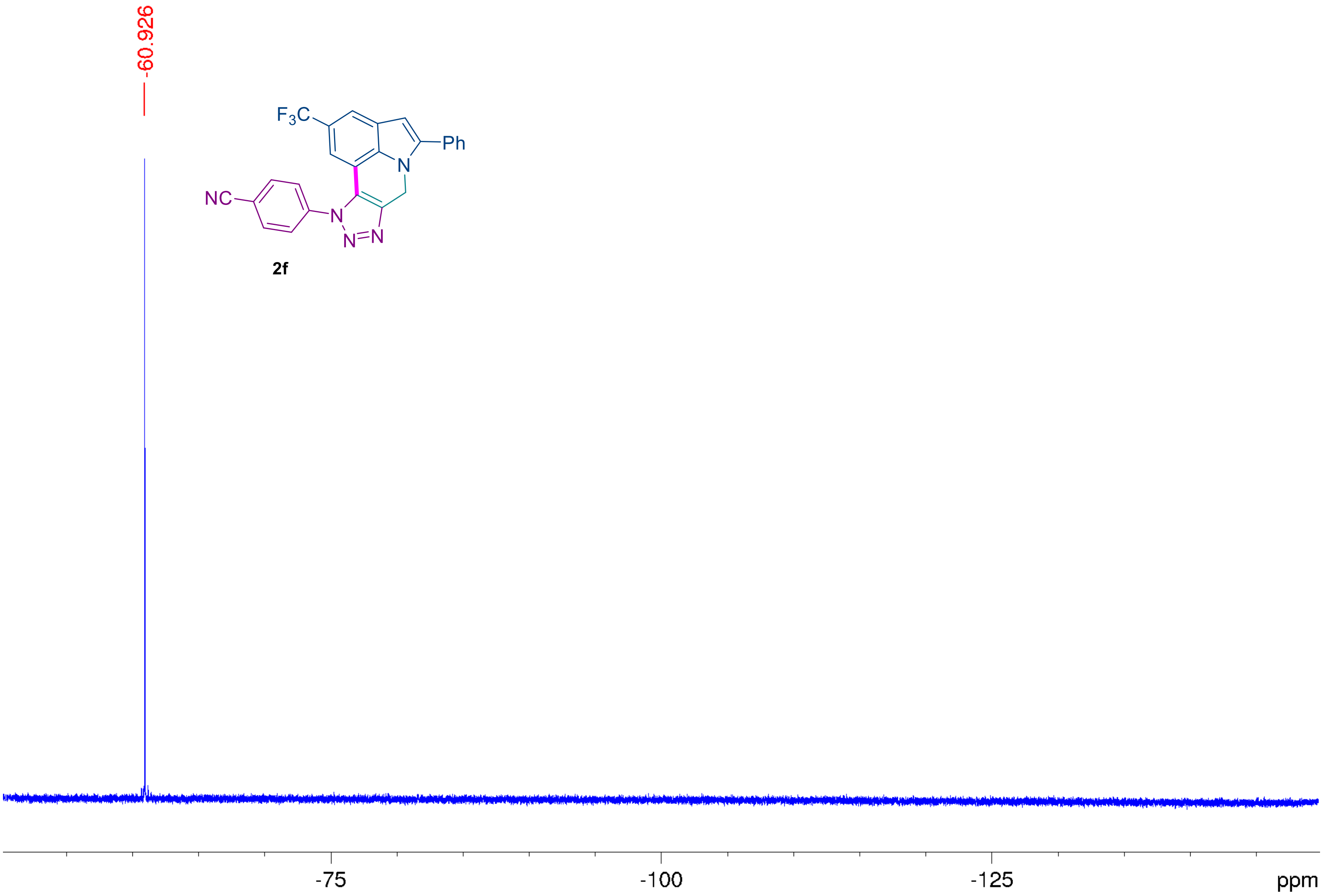

^{1}H NMR-spectrum (400 MHz, $CDCl_3$)

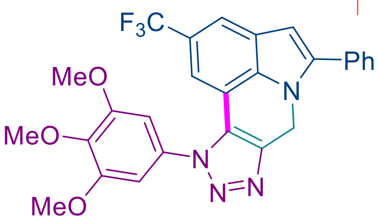

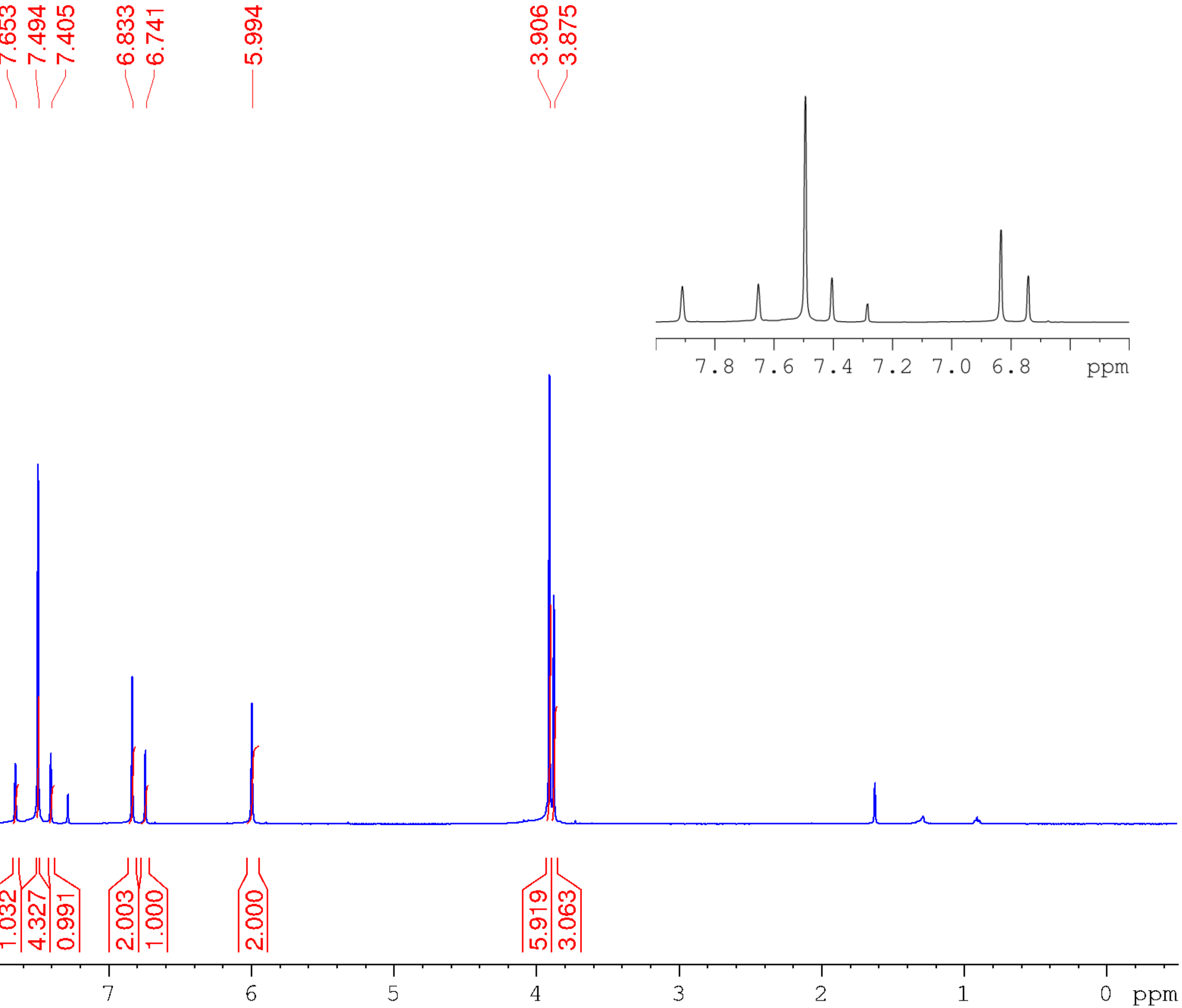

^{13}C NMR-spectrum (100 MHz, $CDCl_3$)

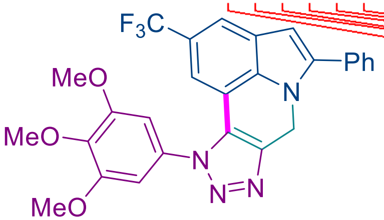

2g

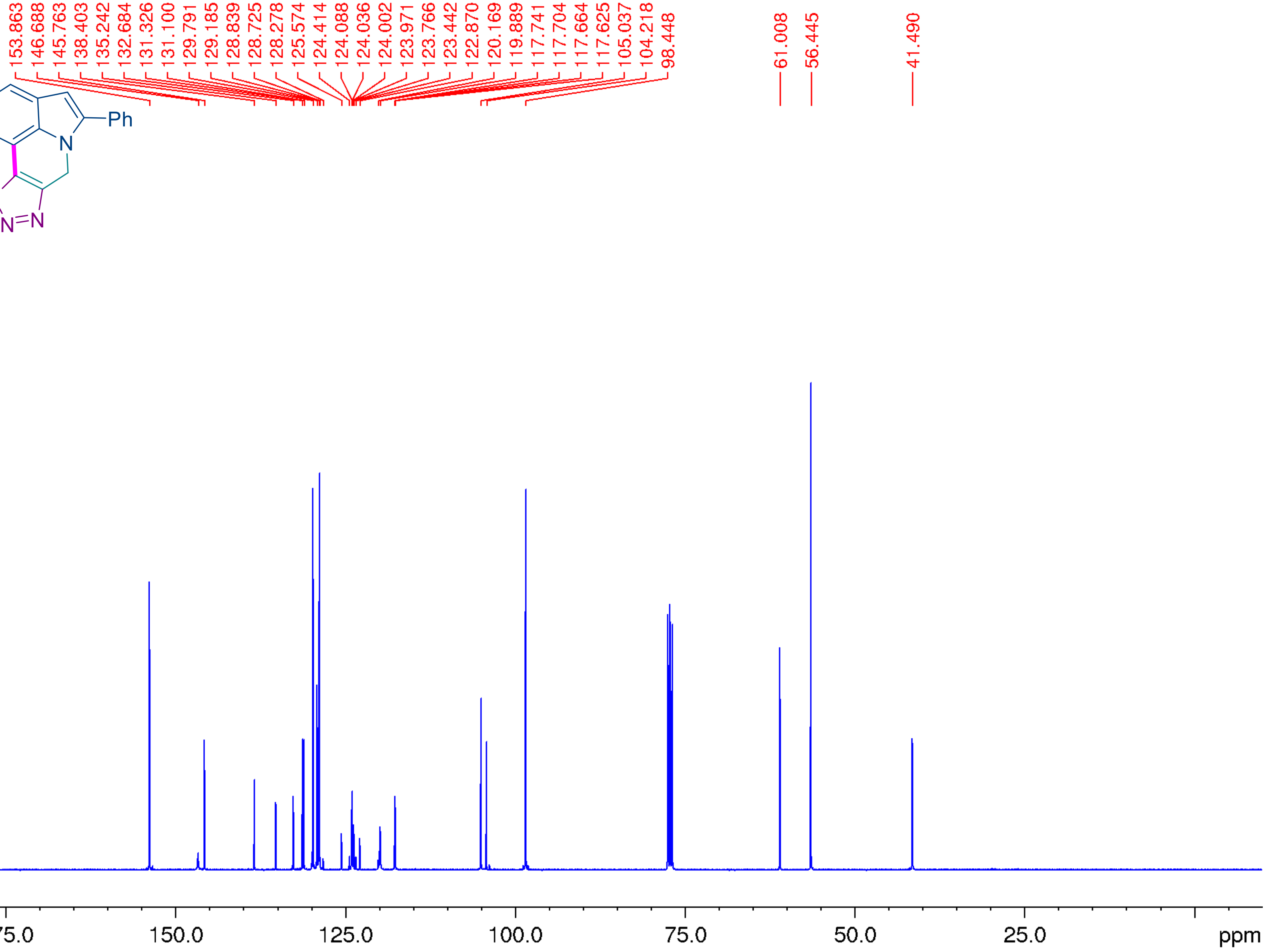

DEPT 135 NMR-spectrum ($CDCl_3$)

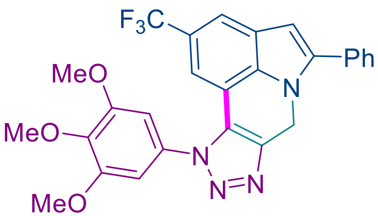

2g

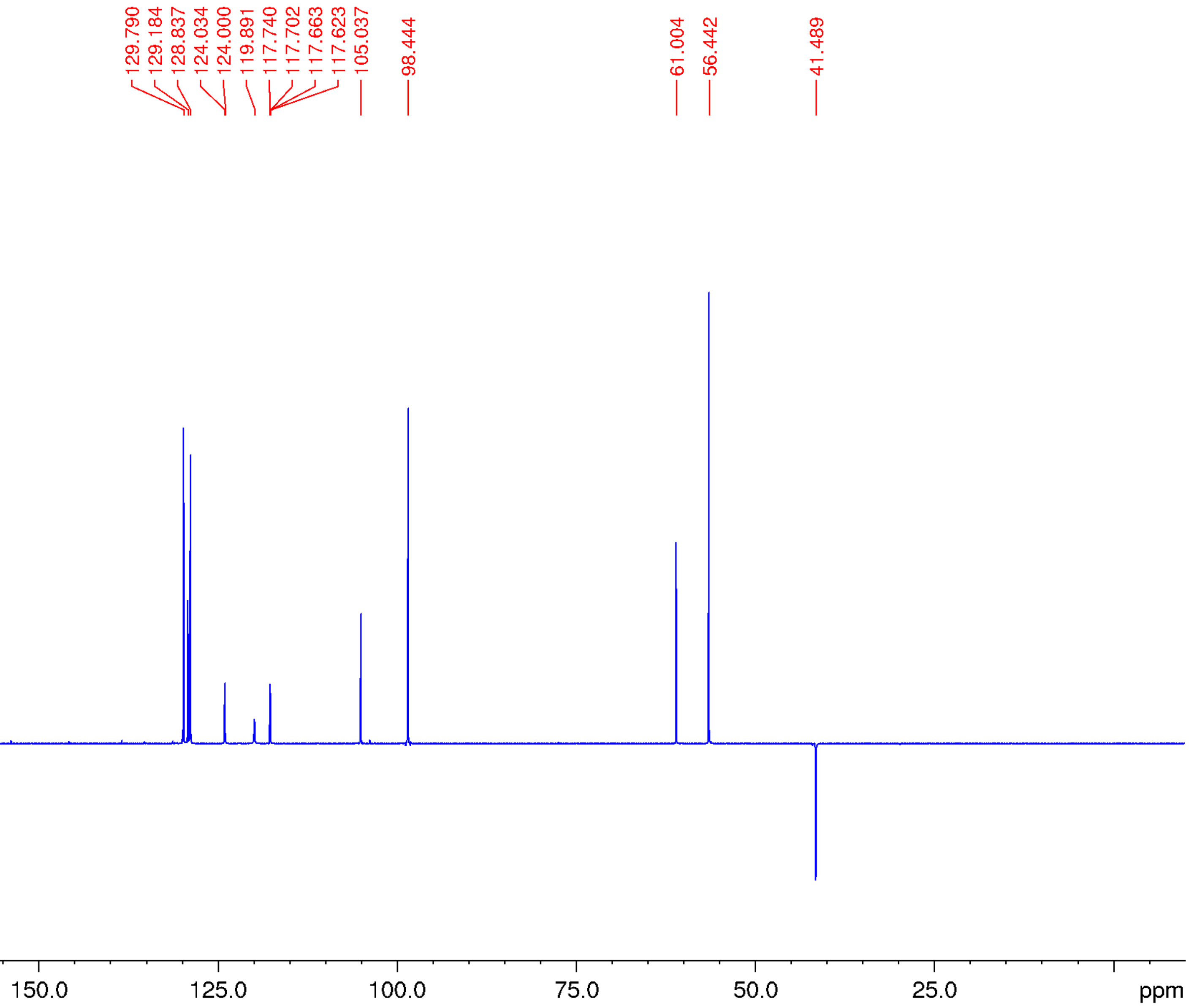

^{19}F NMR-spectrum (376.5 Hz, $CDCl_3$)

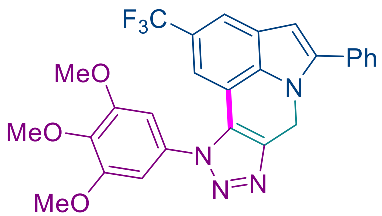

2g

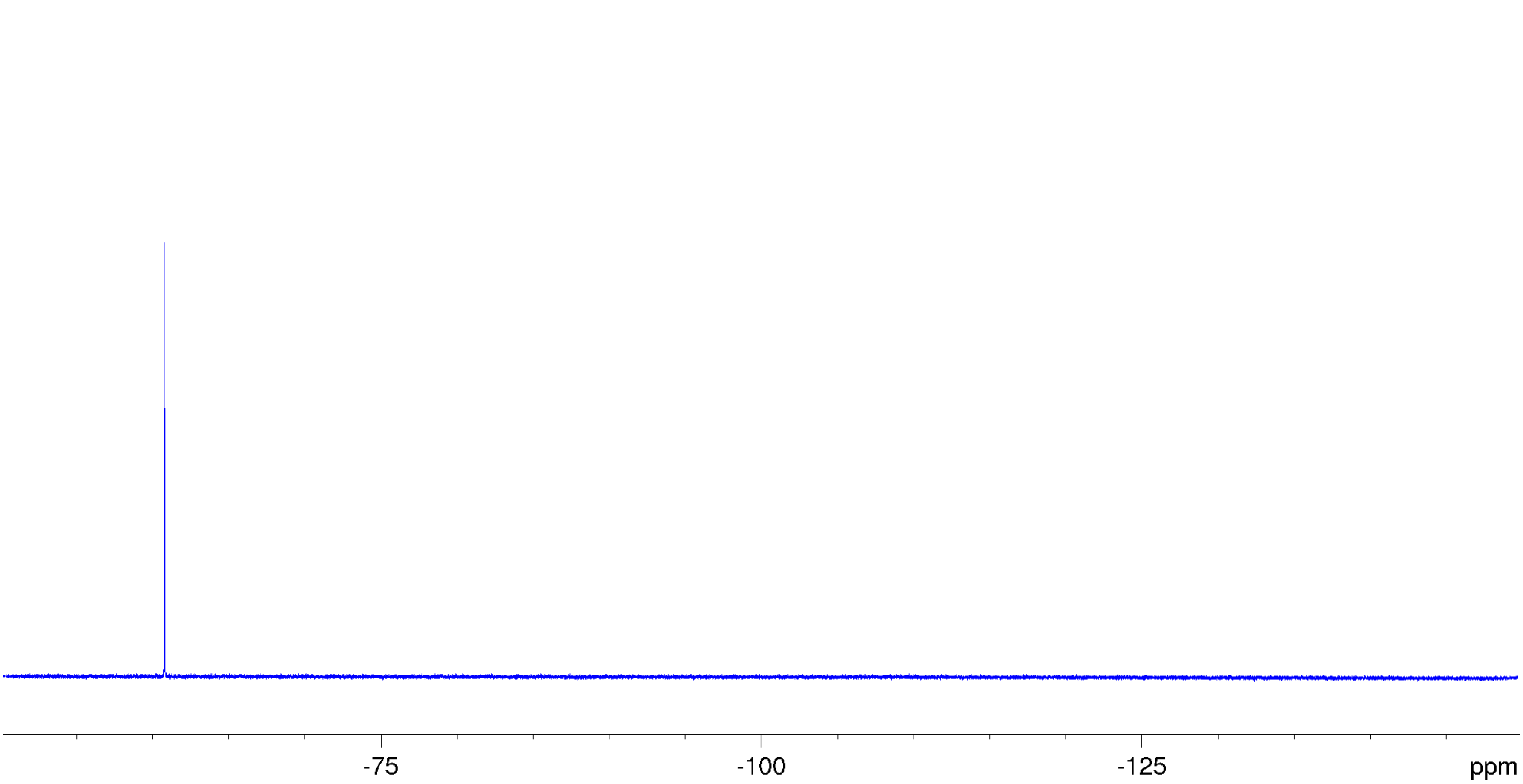

^{1}H NMR-spectrum (400 MHz, $CDCl_3$)

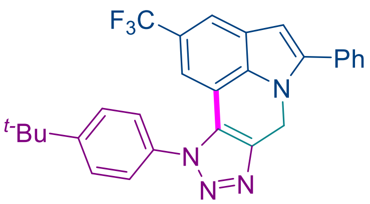

2h

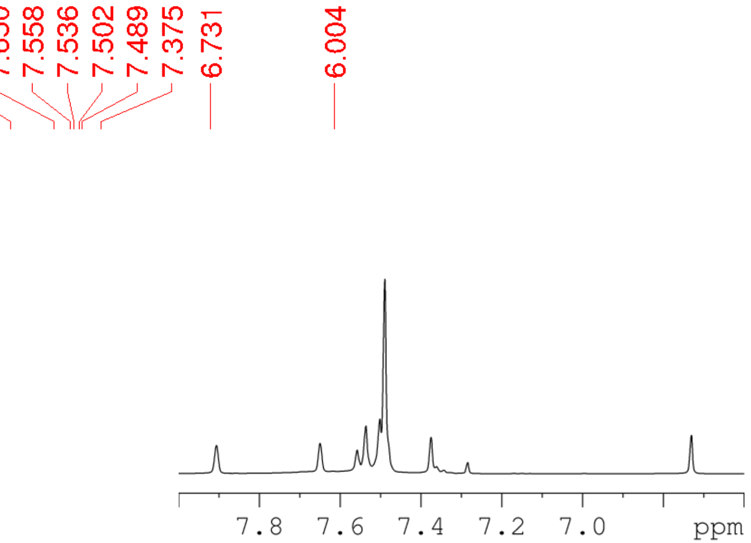

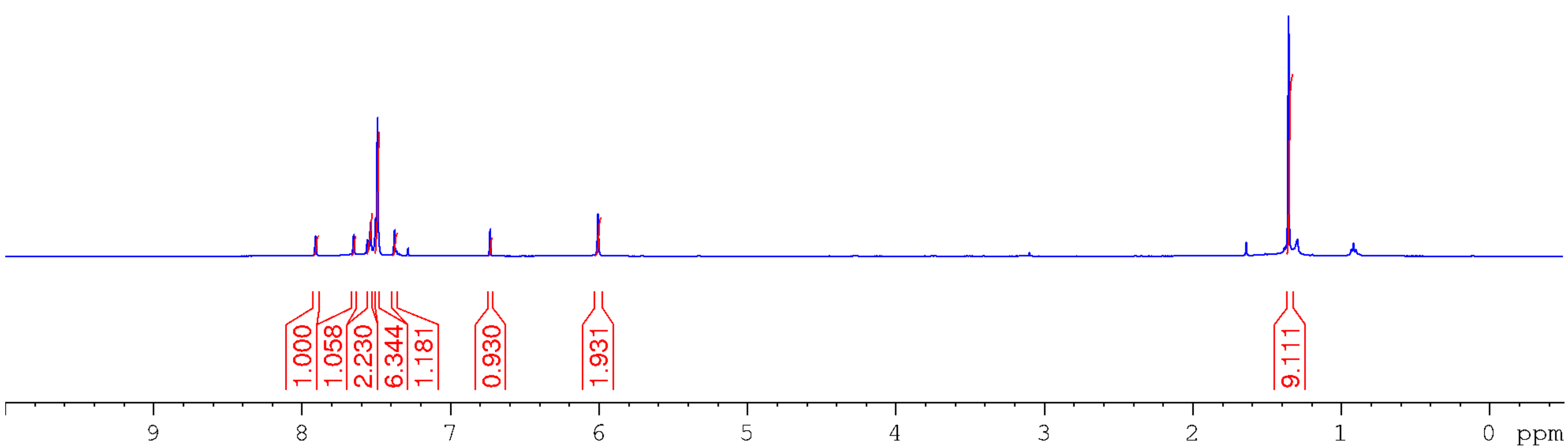

^{13}C NMR-spectrum (100 MHz, $CDCl_3$)

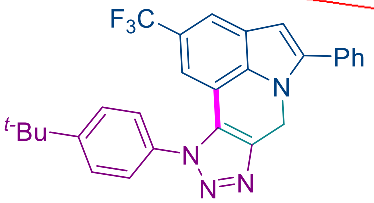

2h

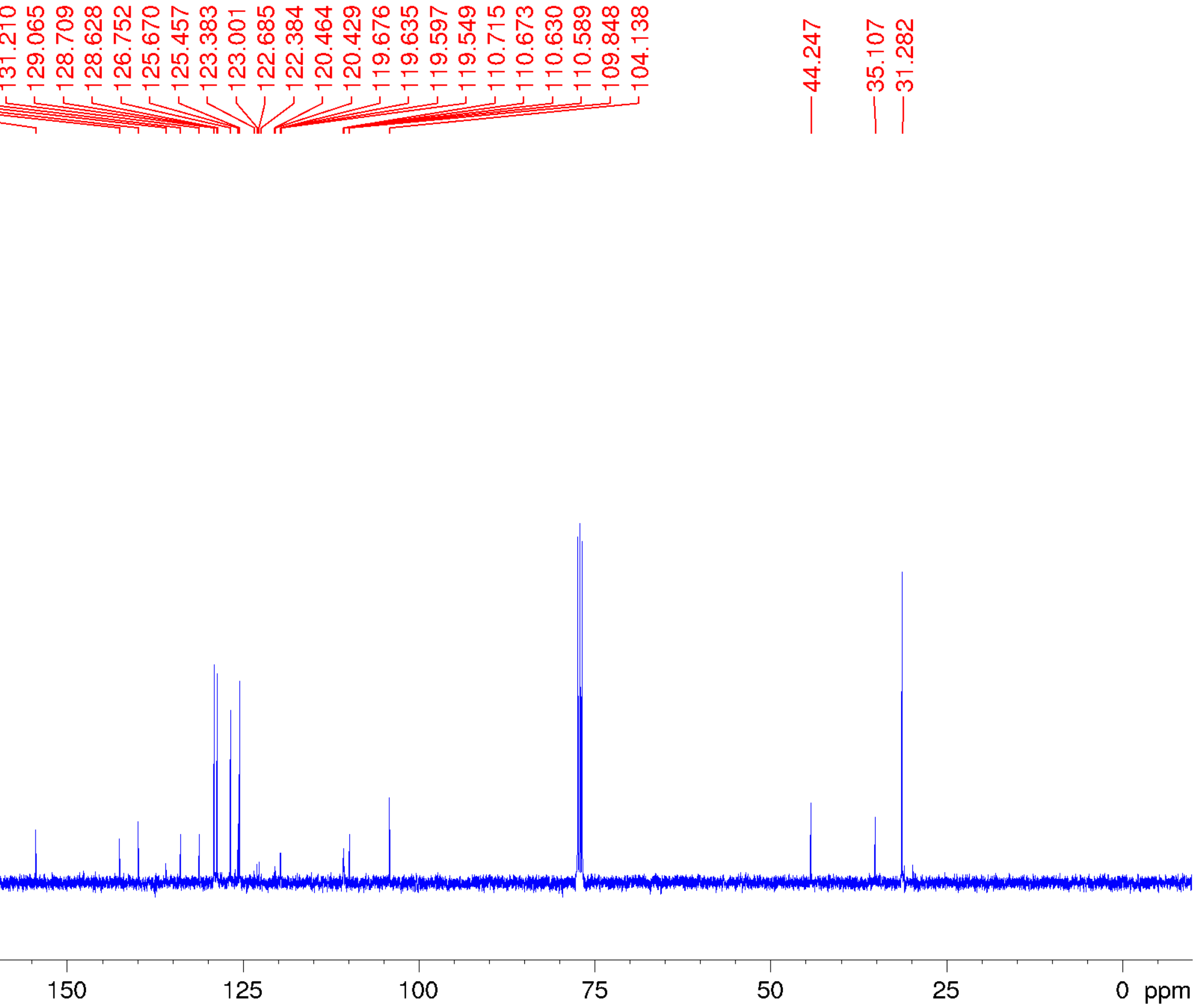

DEPT 135 NMR-spectrum ($CDCl_3$)

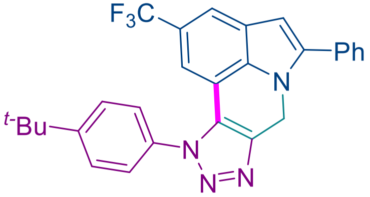

2h

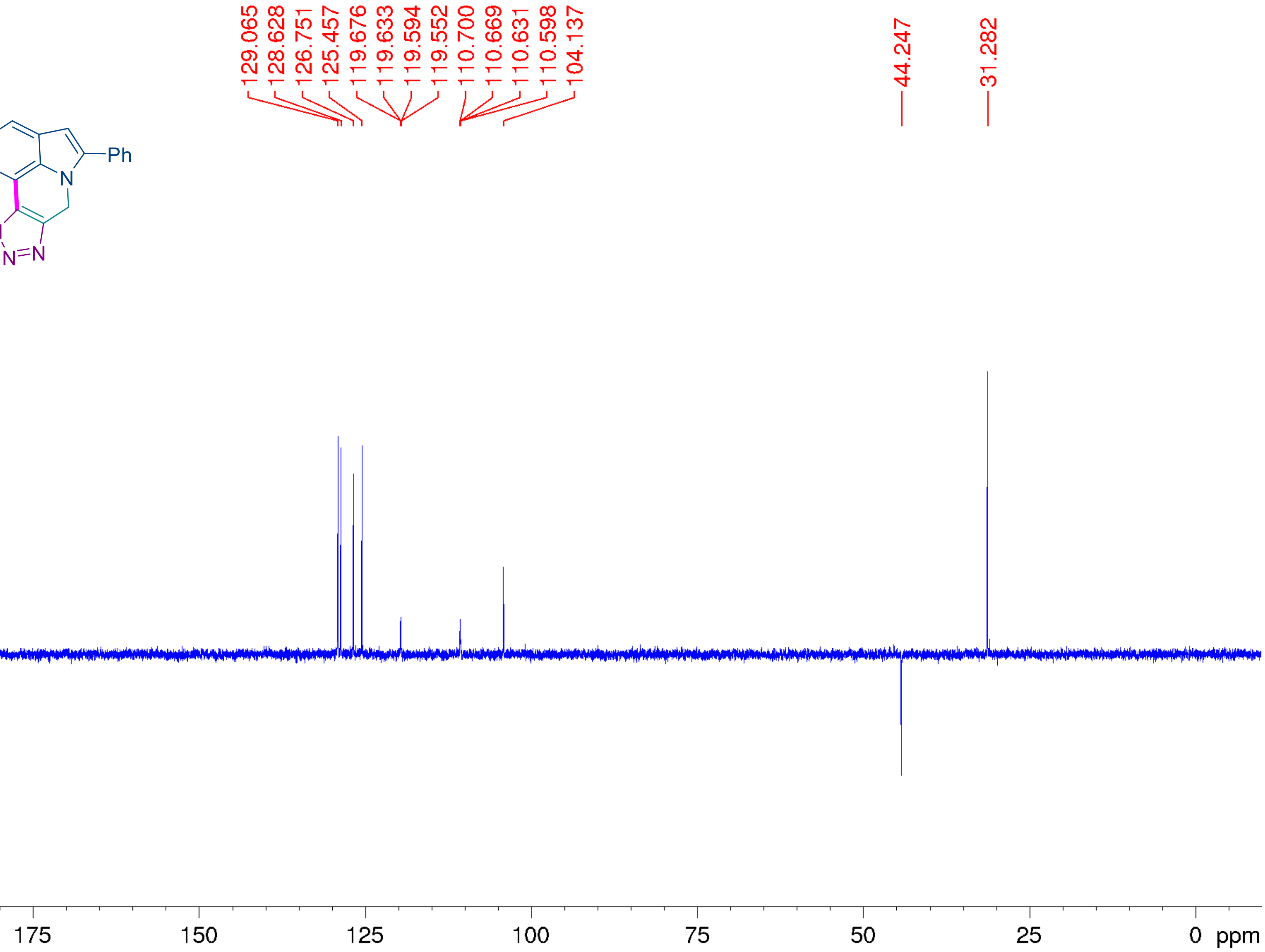

^{19}F NMR-spectrum (376.5 Hz, $CDCl_3$)

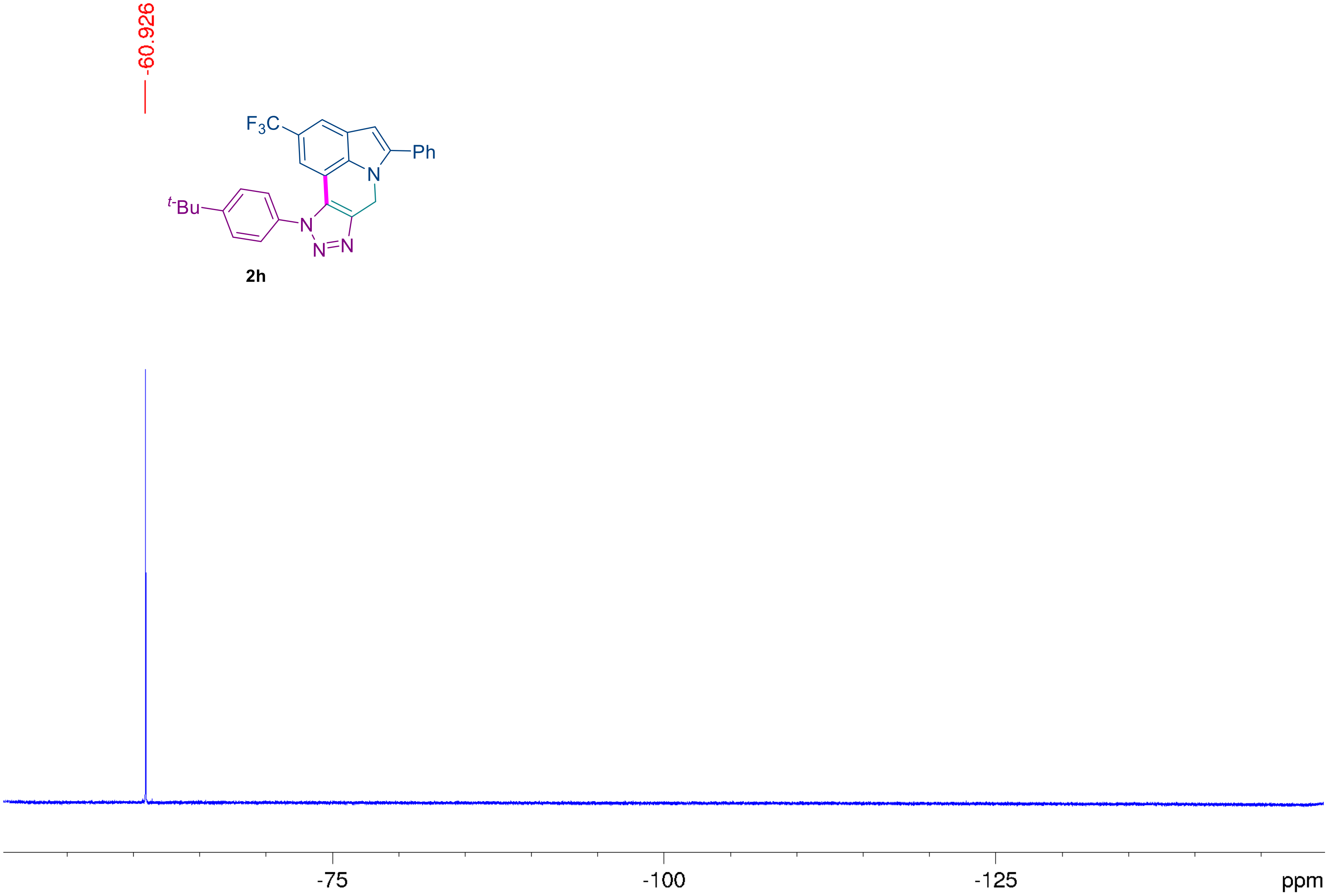

^{1}H NMR-spectrum (400 MHz, $CDCl_3$)

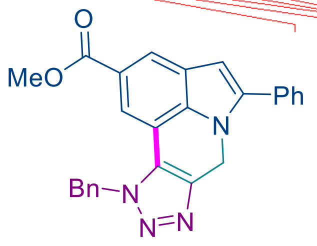

2i

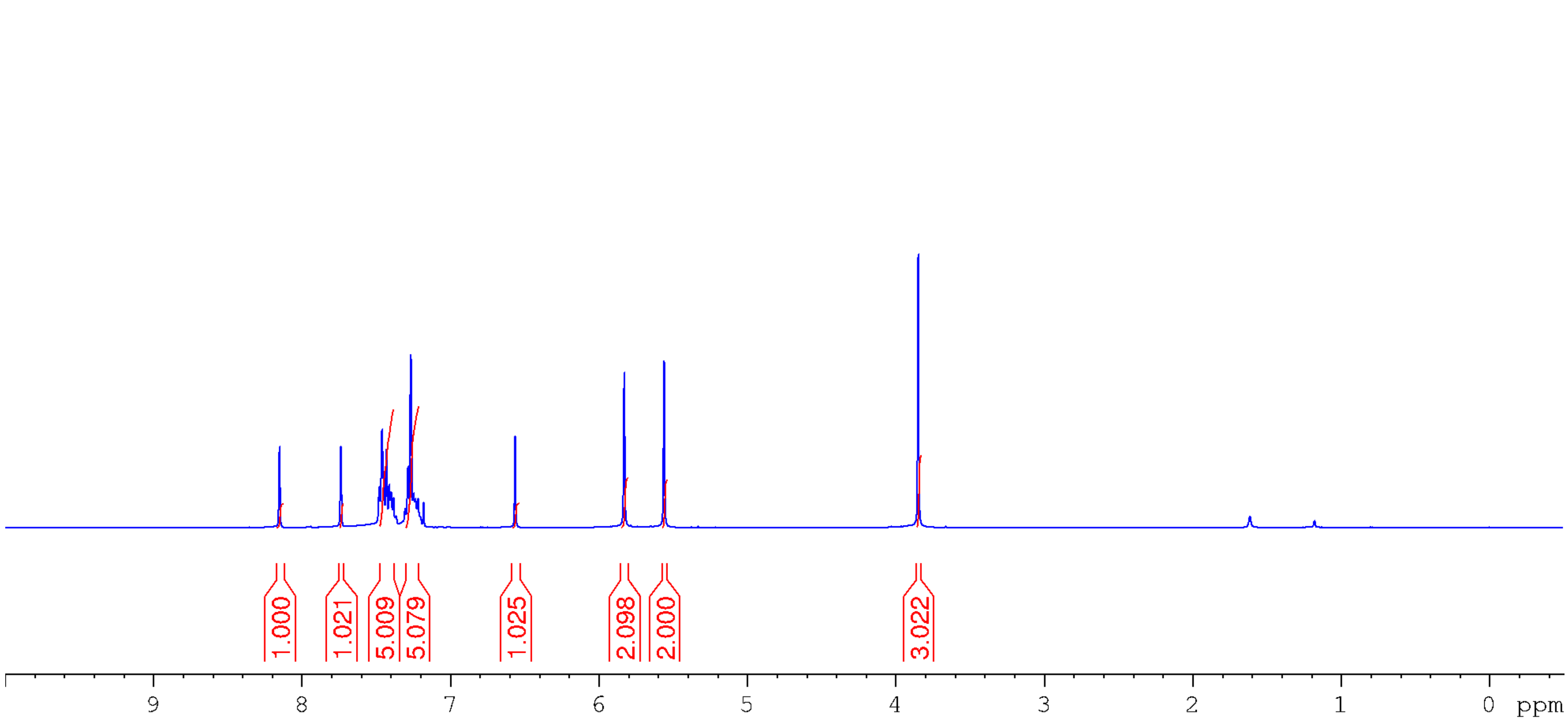

^{13}C NMR-spectrum (100 MHz, $CDCl_3$)

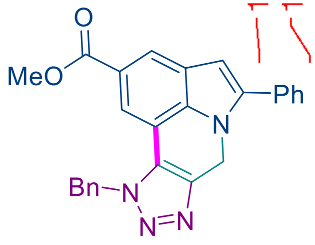

2i

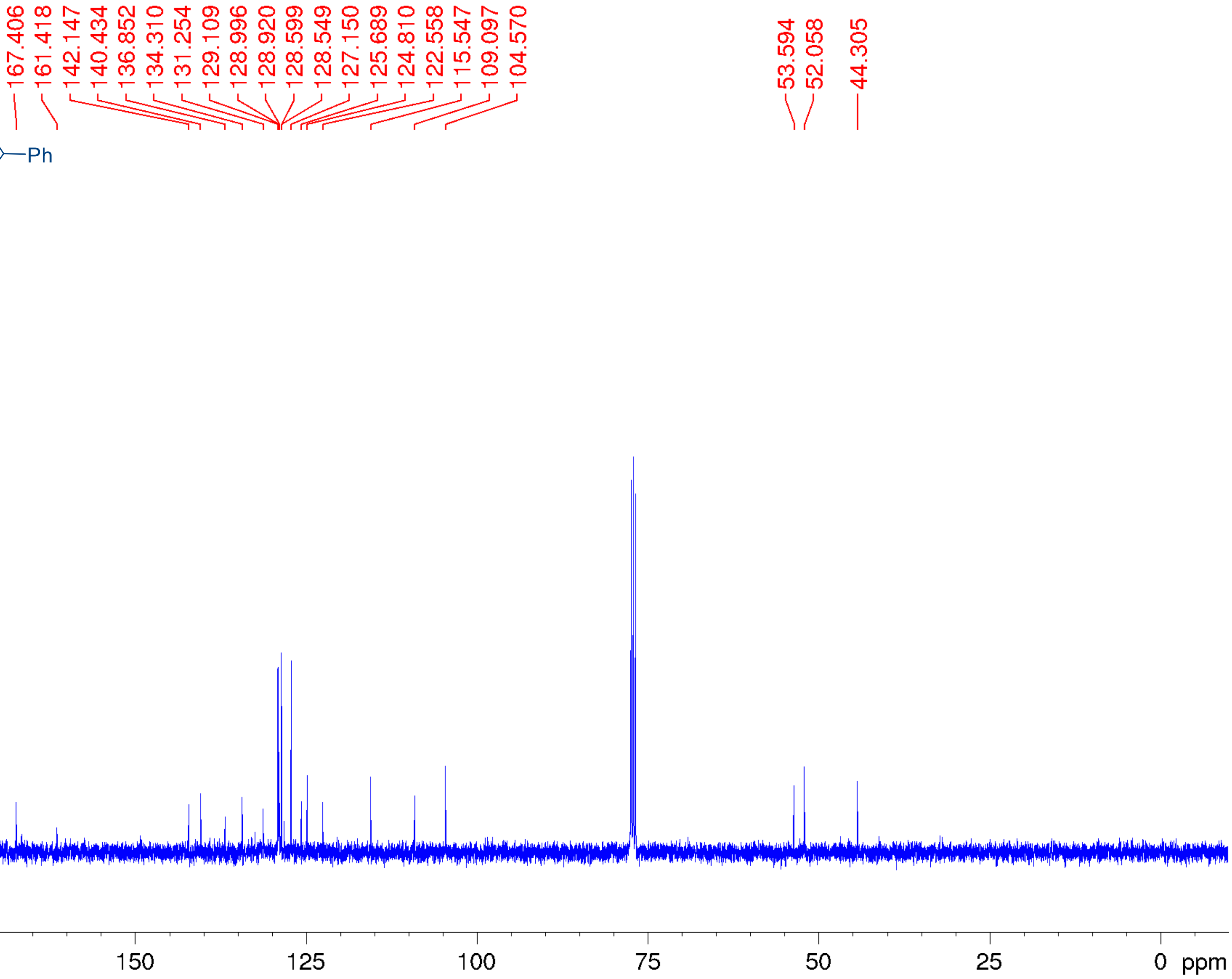

DEPT 135 NMR-spectrum ($CDCl_3$)

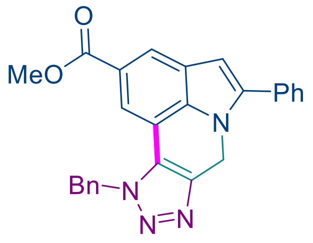

2i

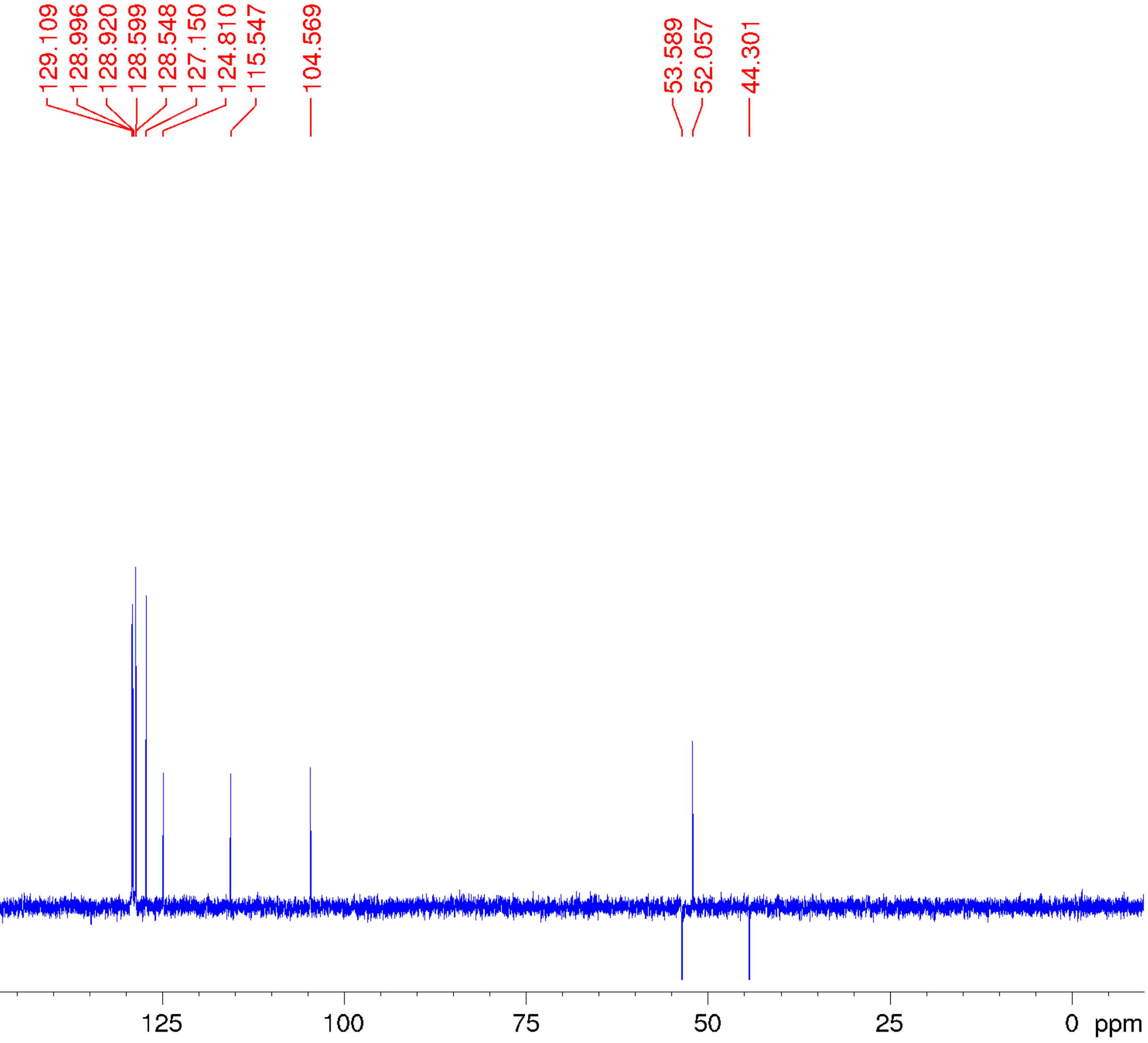

^{1}H NMR-spectrum (400 MHz, $CDCl_3$)

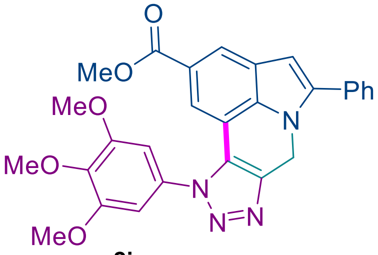

2j

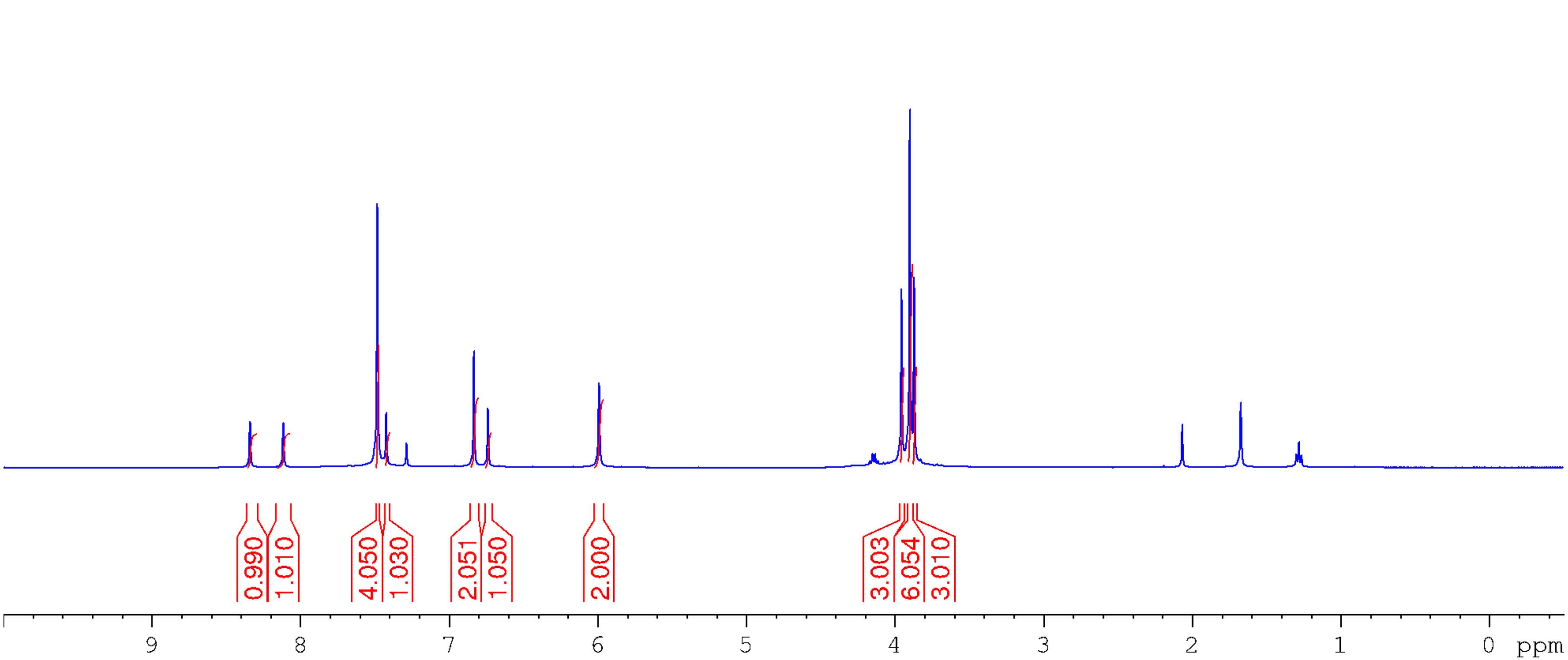

^{13}C NMR-spectrum (100 MHz, $CDCl_3$)

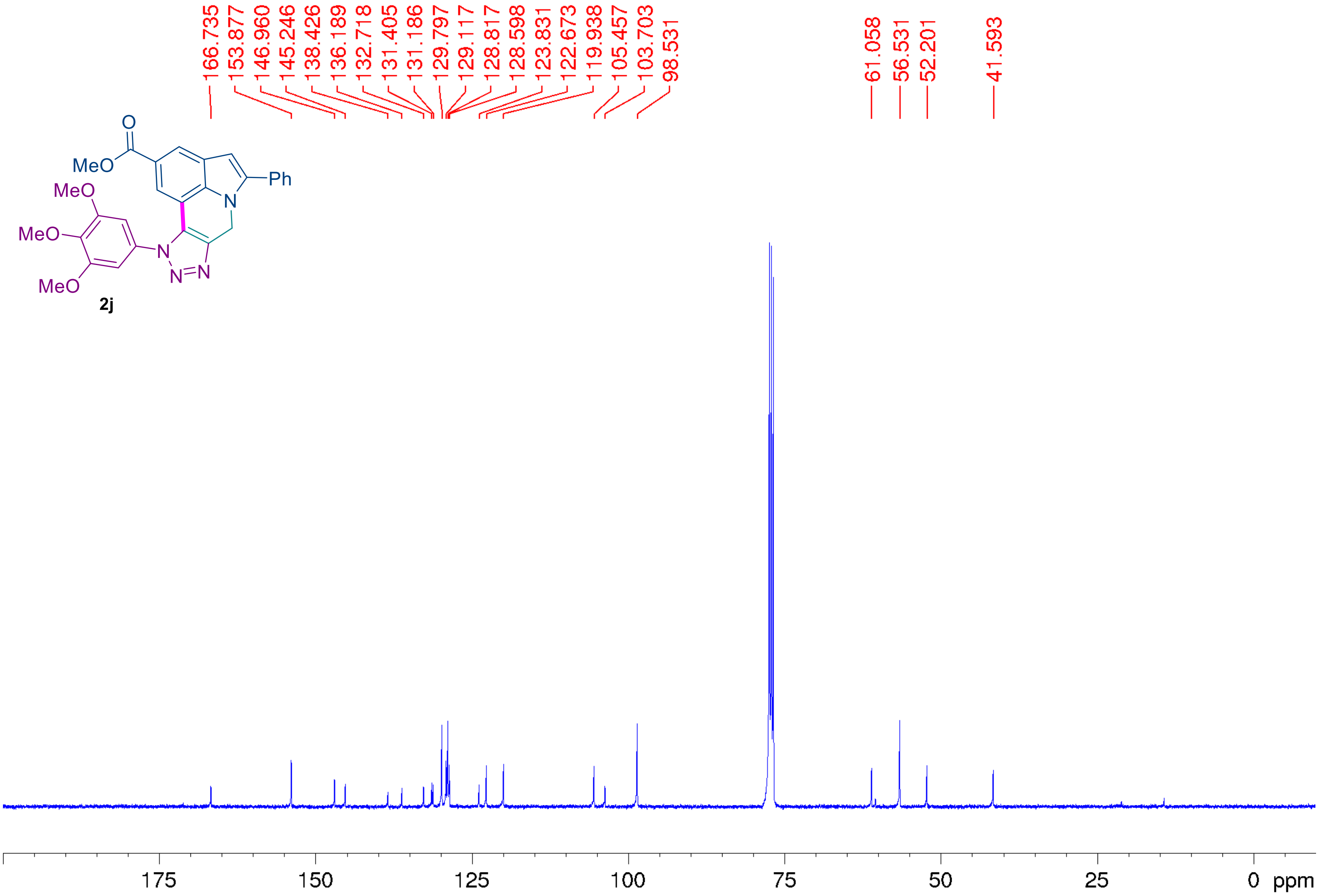

DEPT 135 NMR-spectrum ($CDCl_3$)

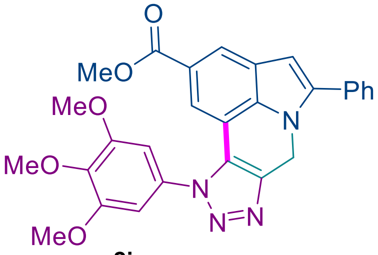

2j

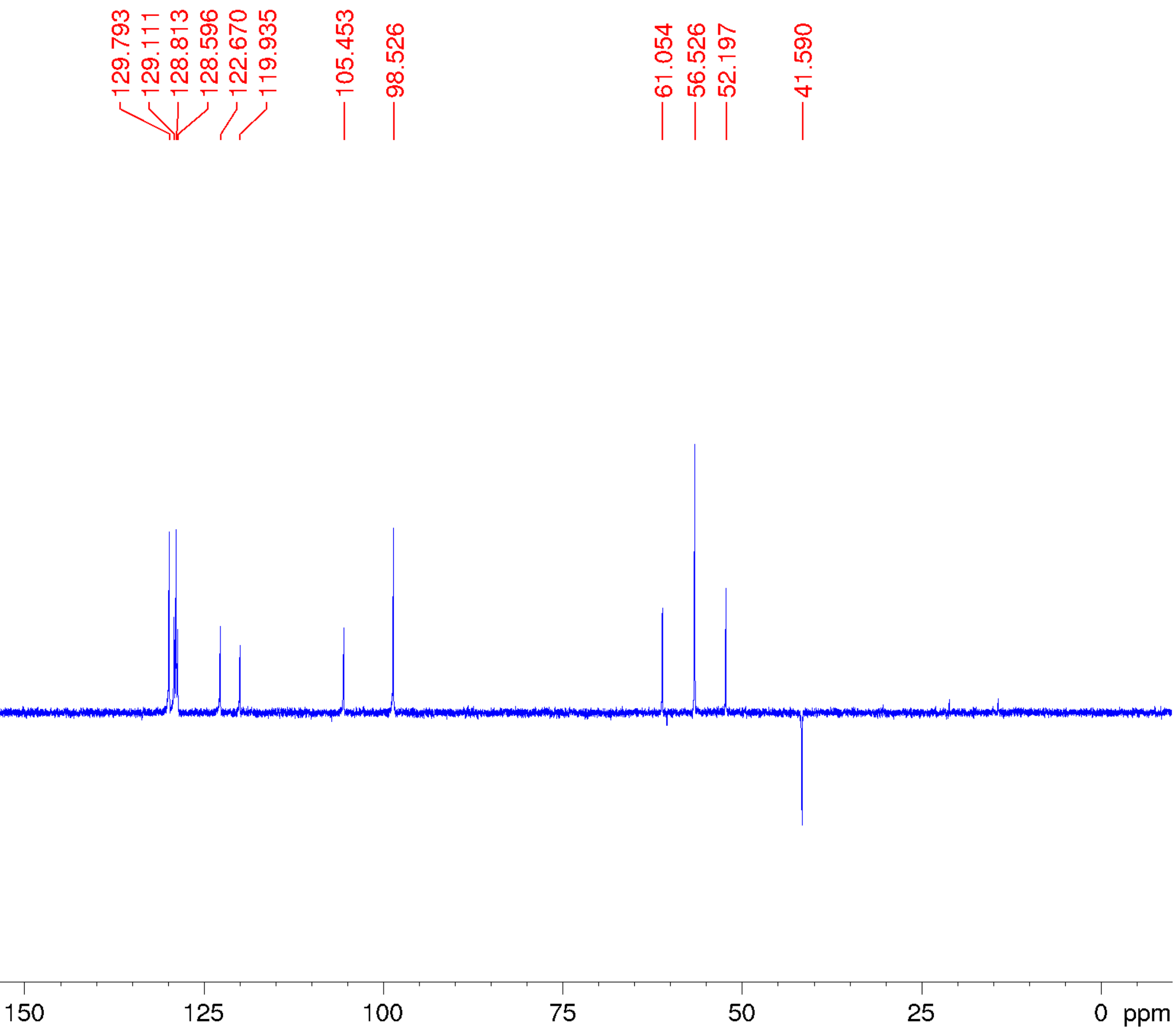

^{1}H NMR-spectrum (400 MHz, $CDCl_3$)

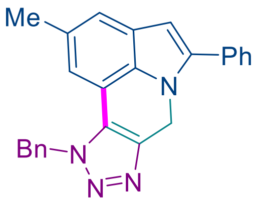

2k

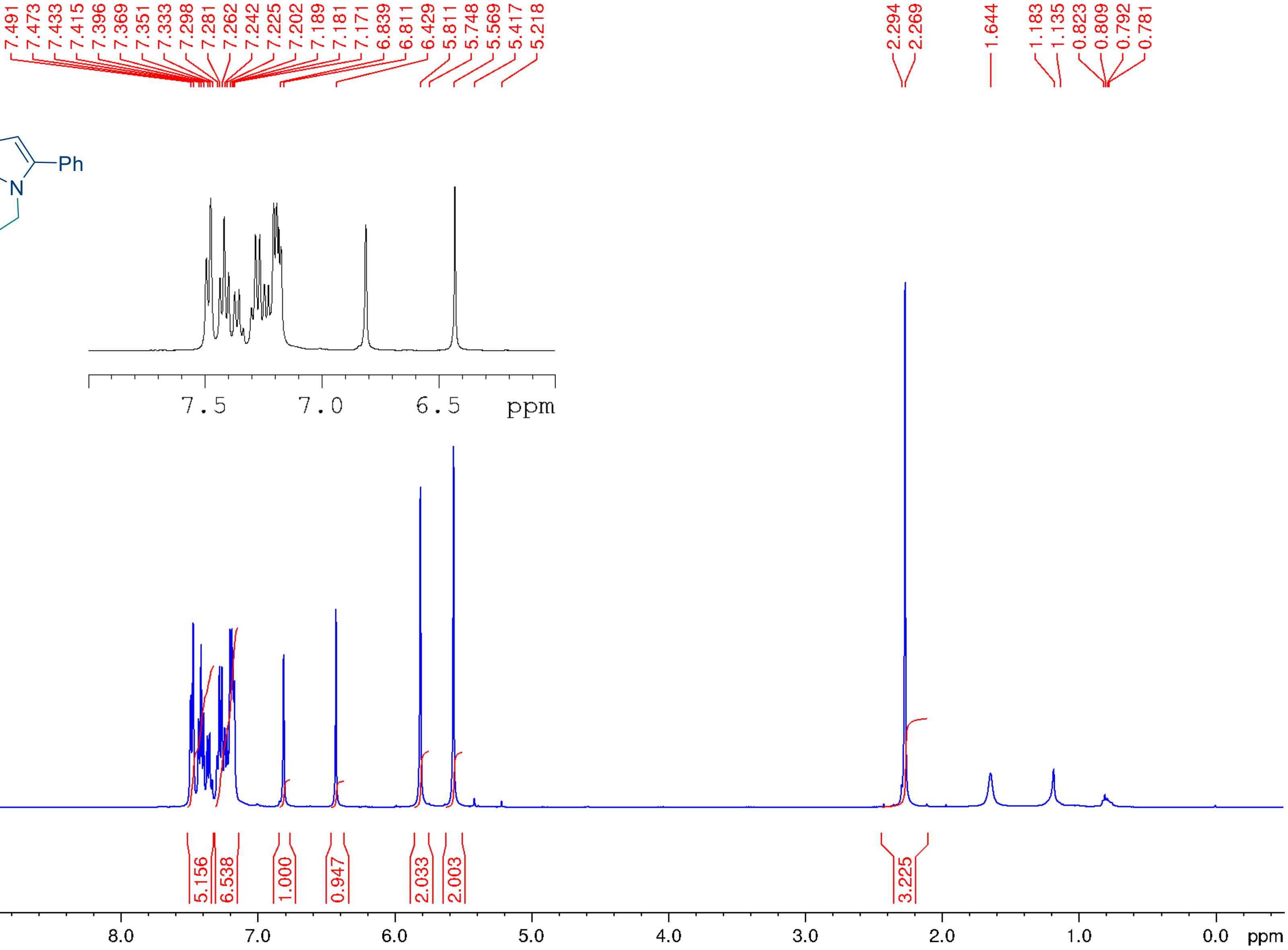

^{13}C NMR-spectrum (100 MHz, $CDCl_3$)

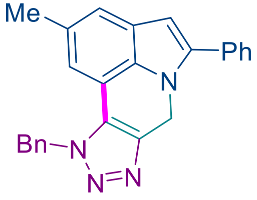

2k

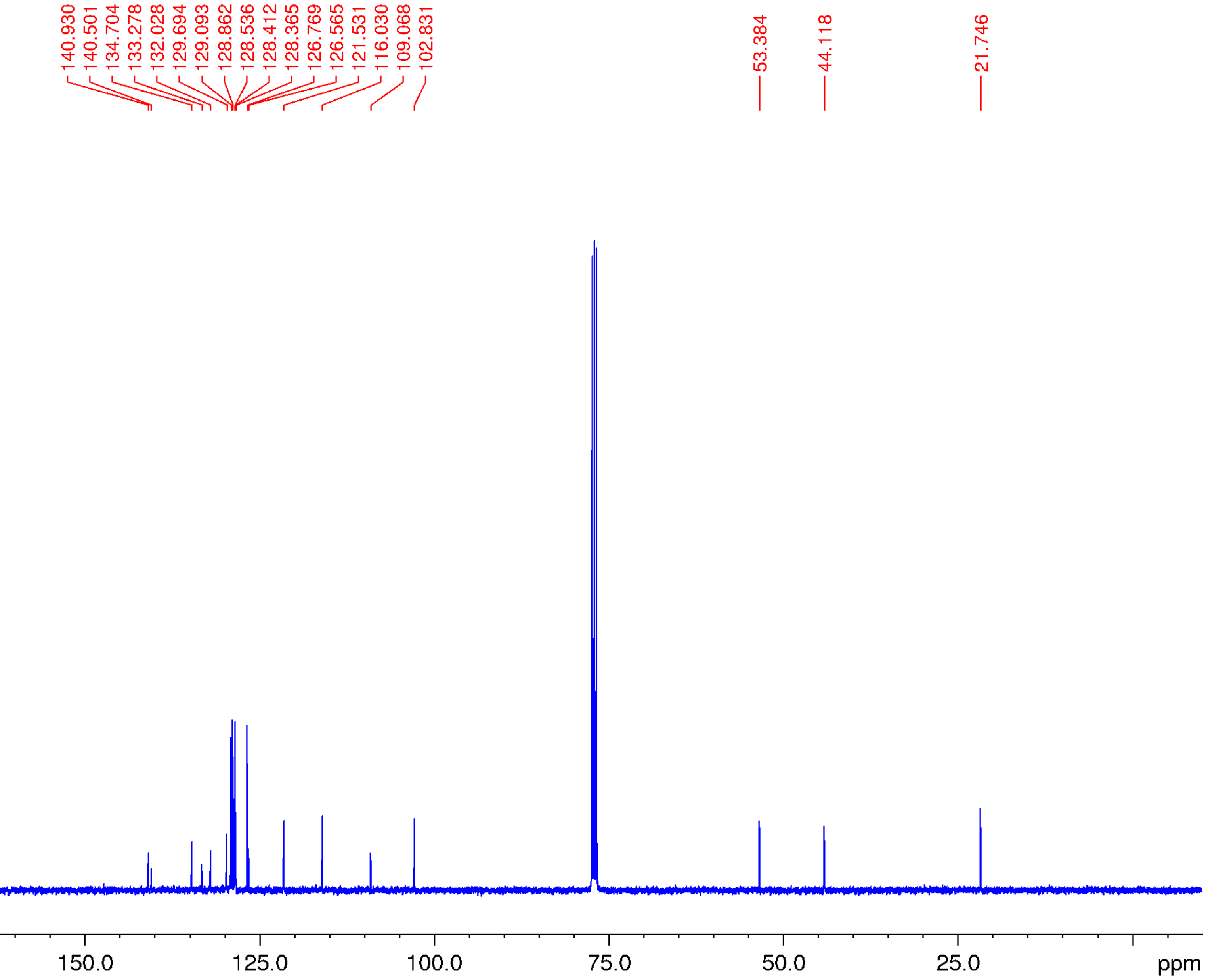

DEPT 135 NMR-spectrum ($CDCl_3$)

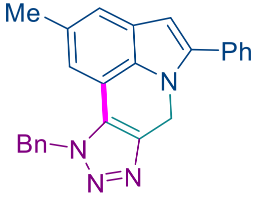

2k

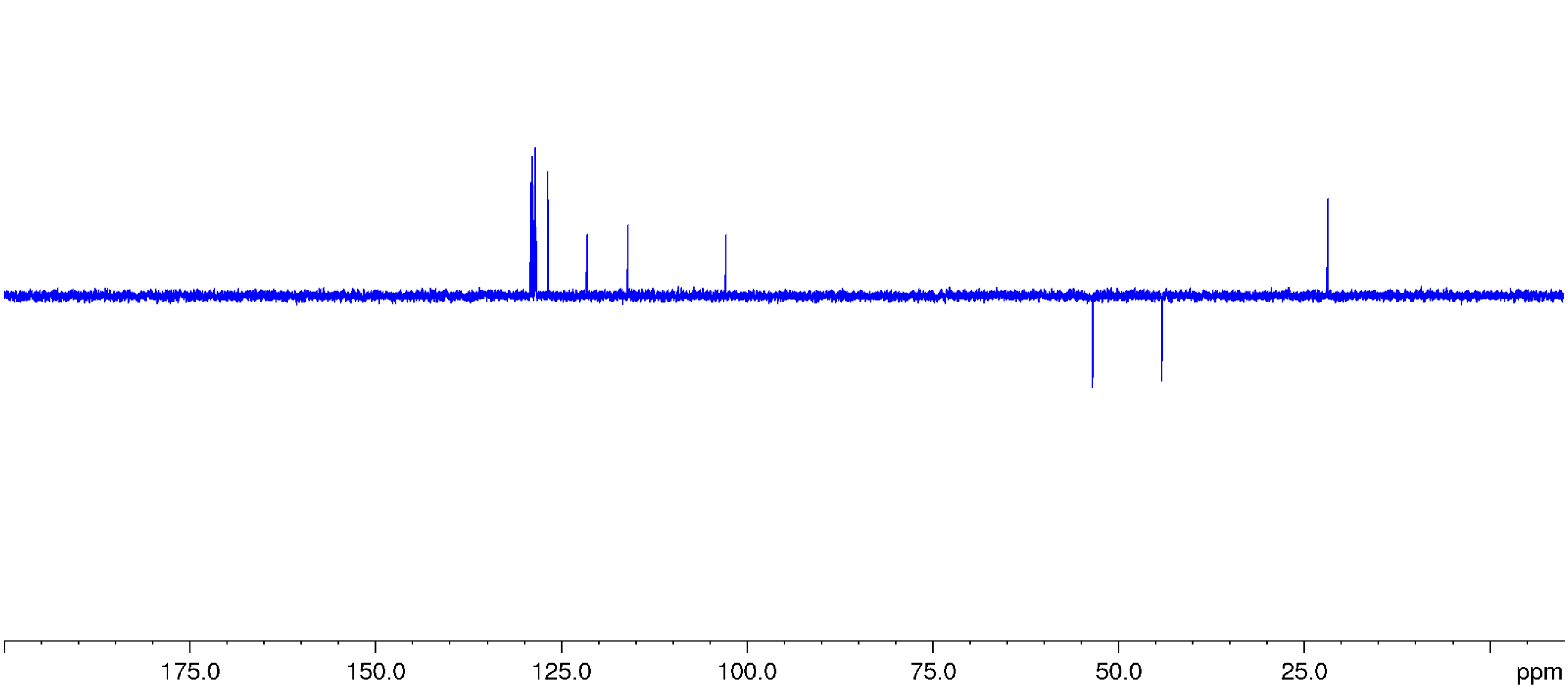

^{1}H NMR-spectrum (400 MHz, $CDCl_3$)

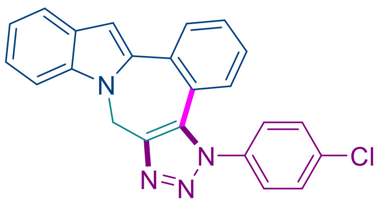

3a

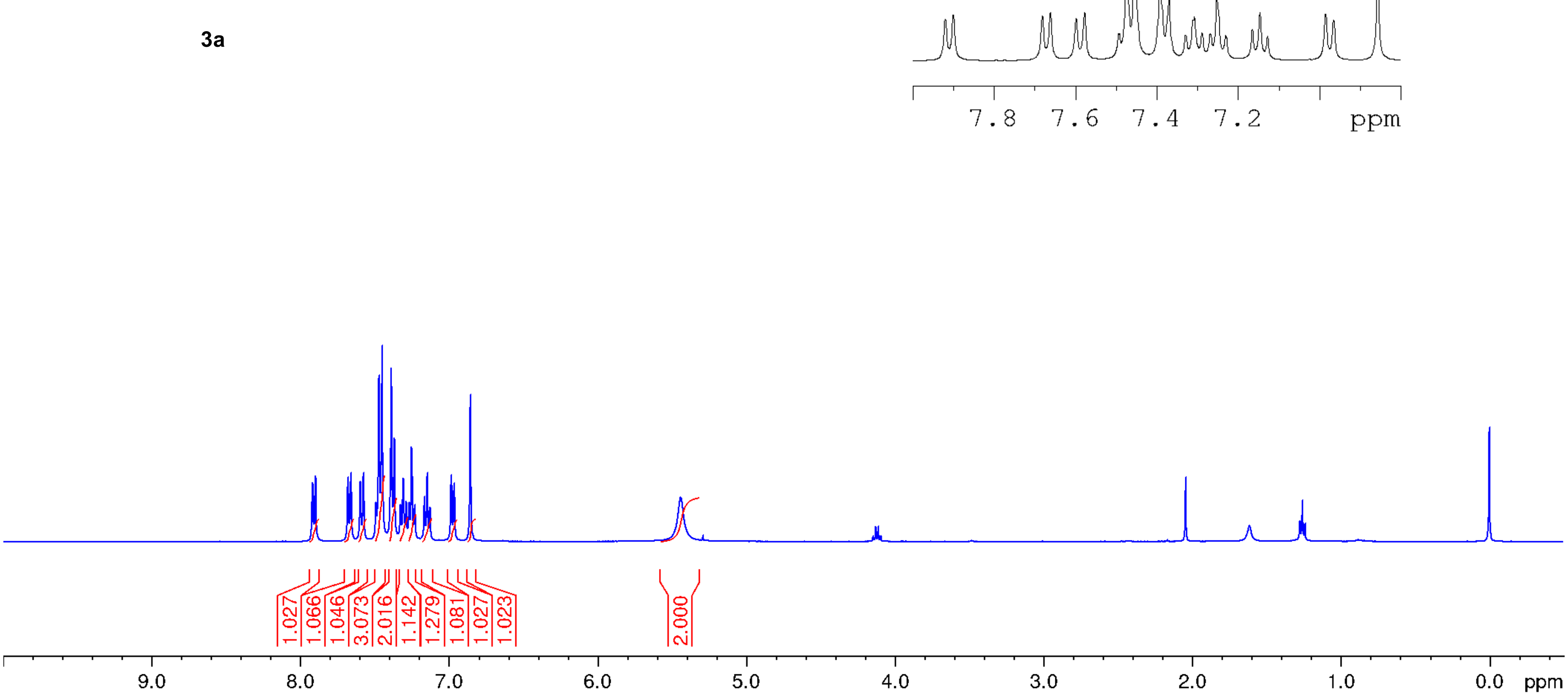

^{13}C NMR-spectrum (100 MHz, $CDCl_3$)

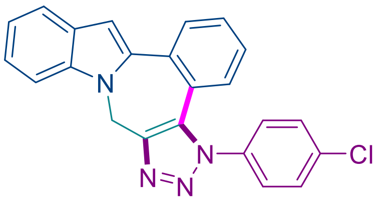

3a

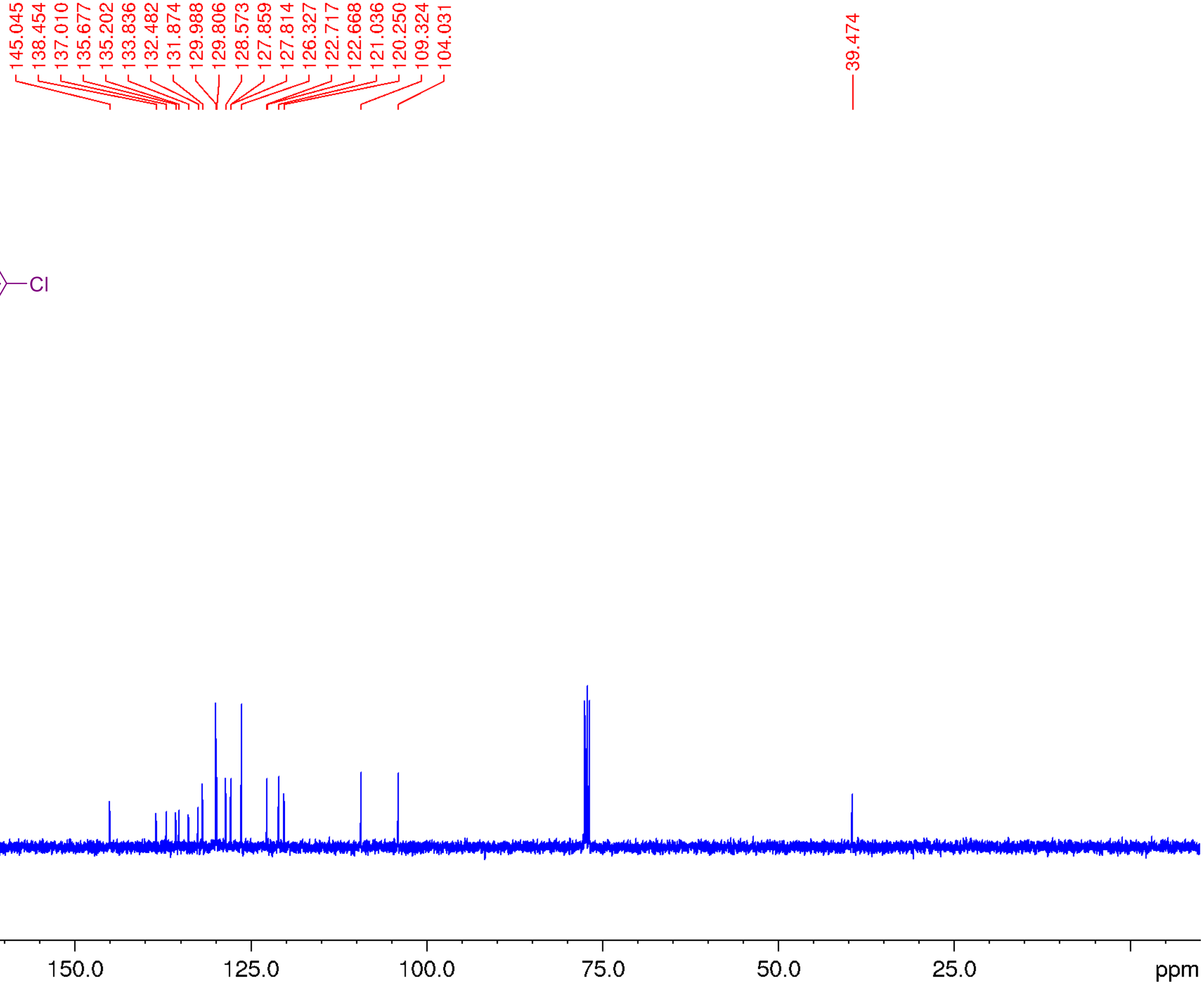

DEPT 135 NMR-spectrum ($CDCl_3$)

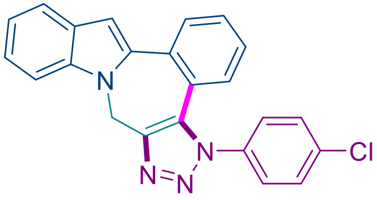

3a

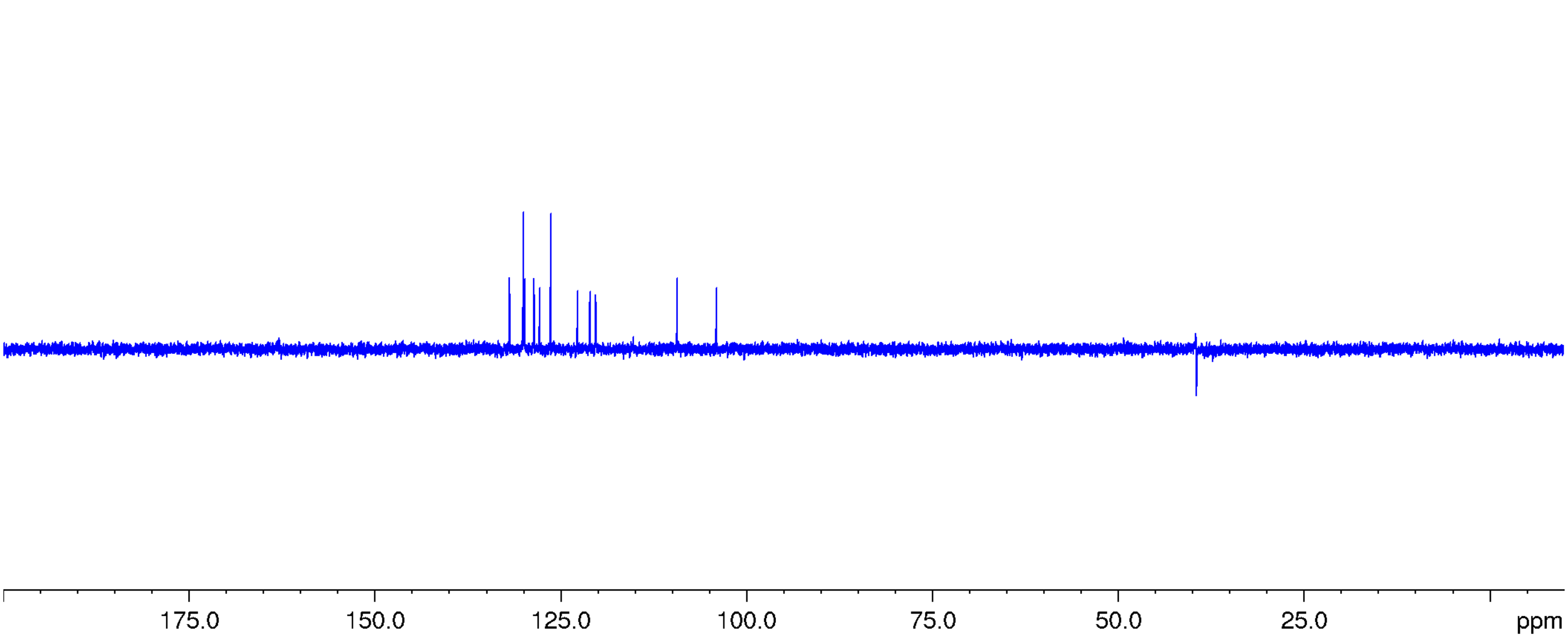

^{1}H NMR-spectrum (400 MHz, $CDCl_3$)

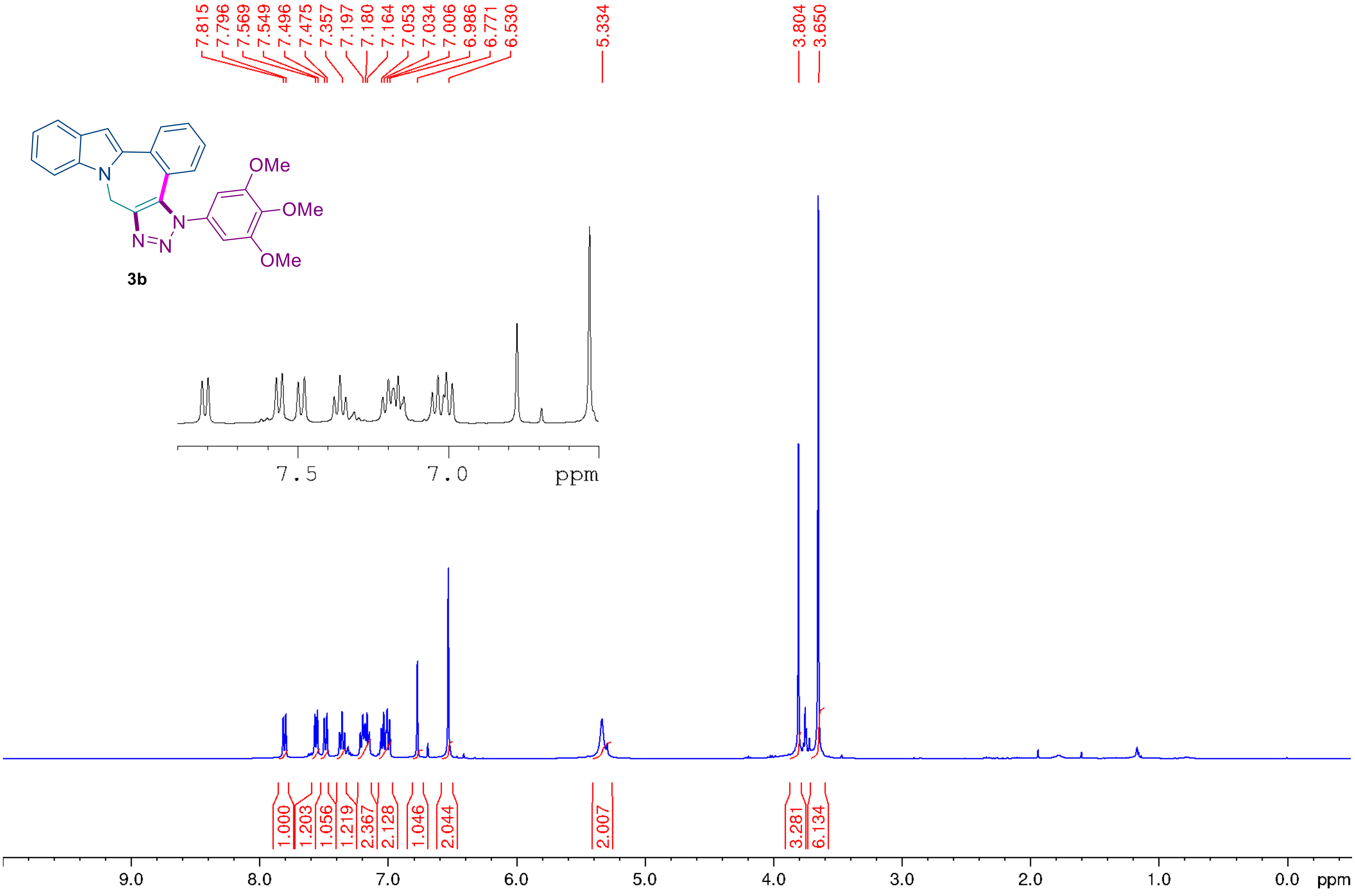

^{13}C NMR-spectrum (100 MHz, $CDCl_3$)

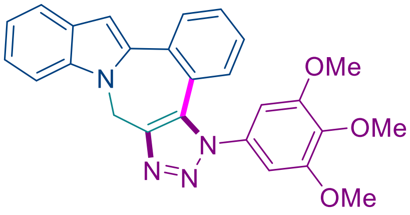

3b

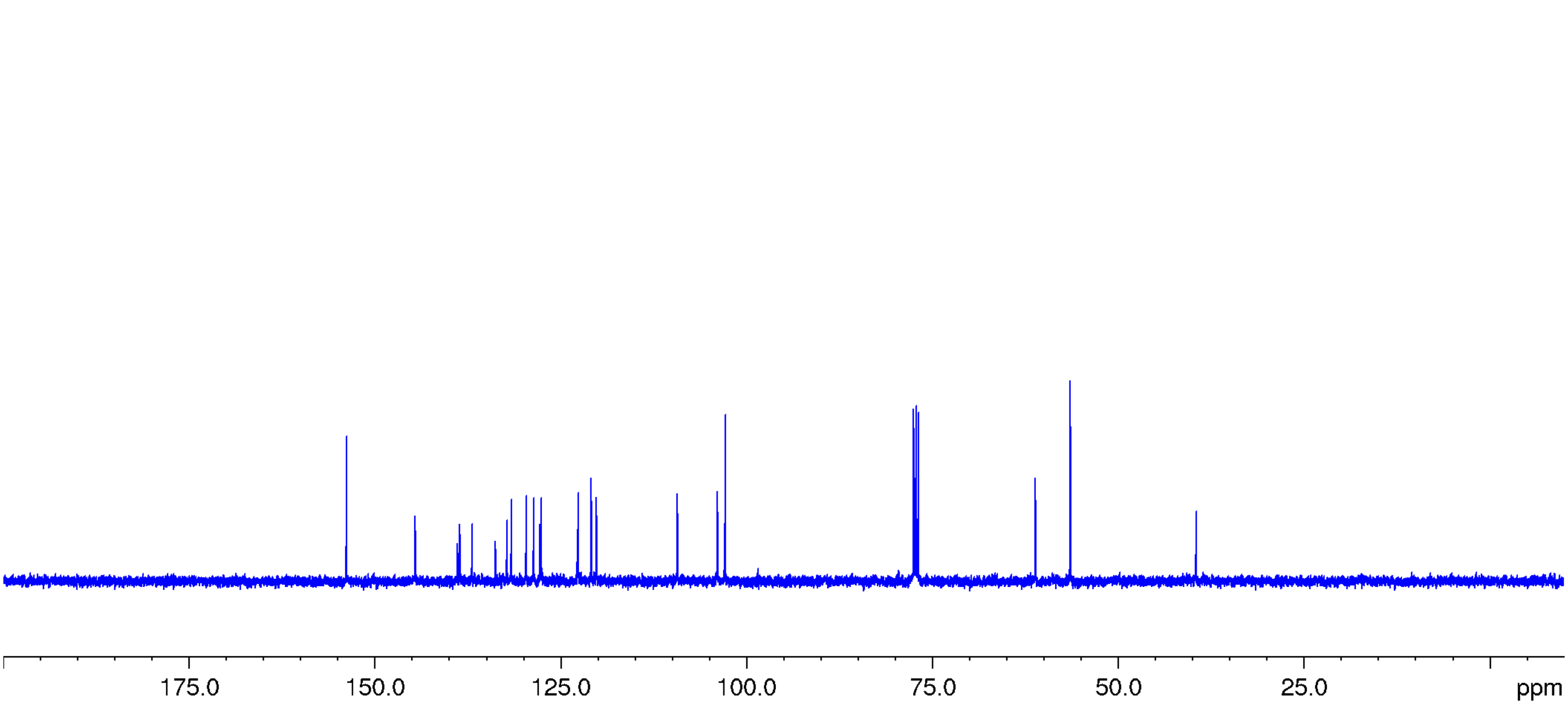

DEPT 135 NMR-spectrum ($CDCl_3$)

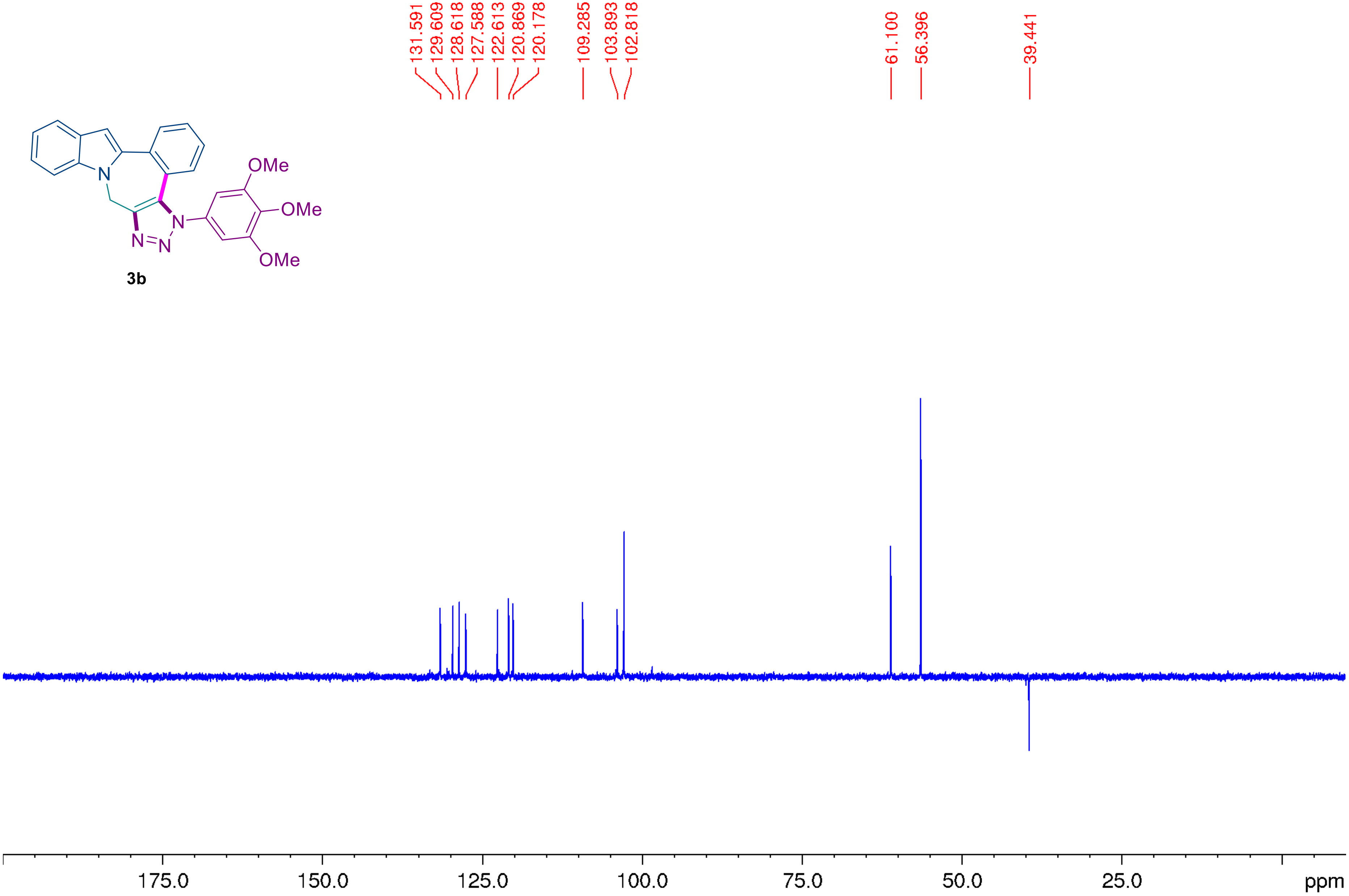

^{1}H NMR-spectrum (400 MHz, $CDCl_3$)

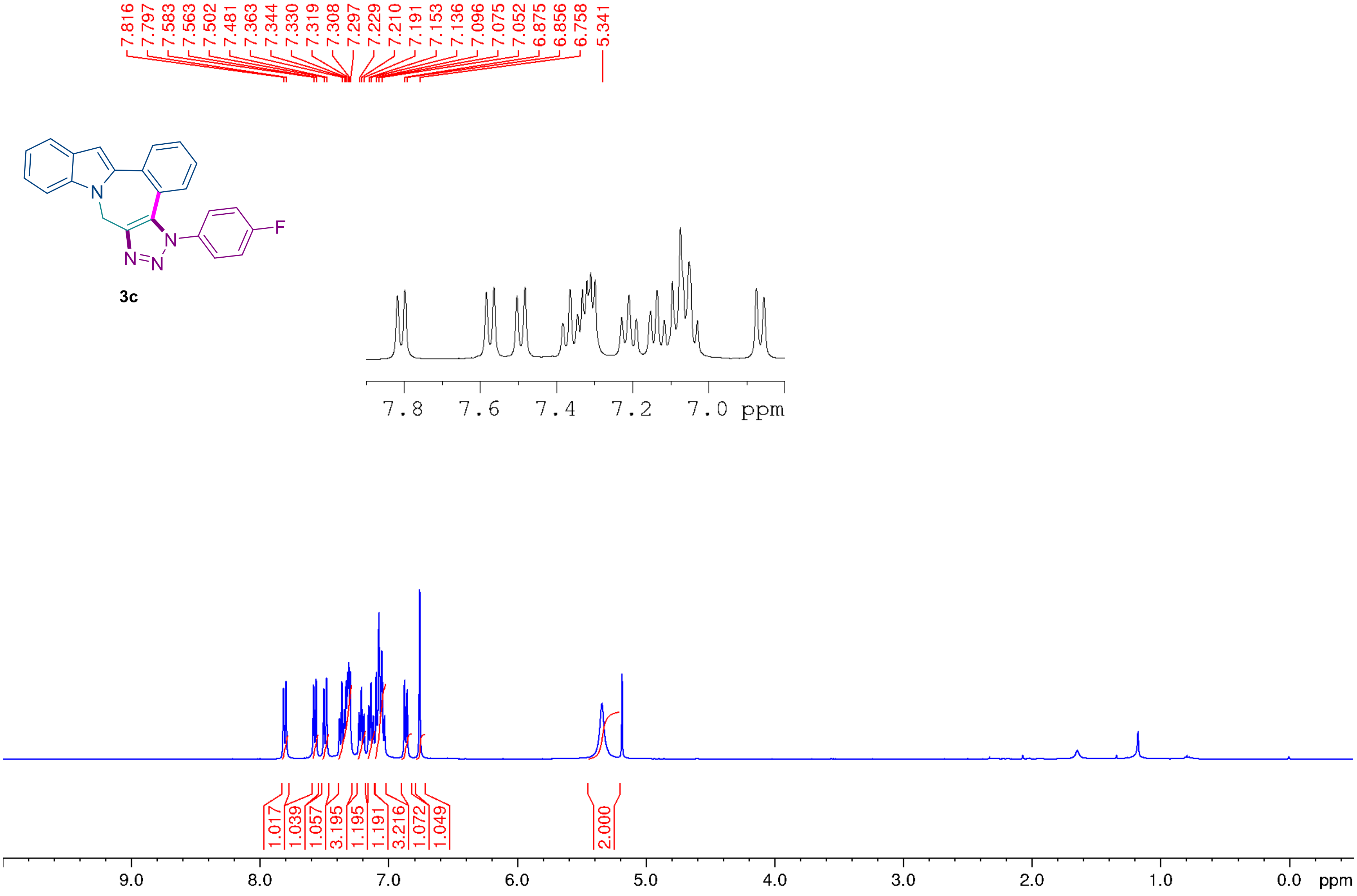

^{13}C NMR-spectrum (100 MHz, $CDCl_3$)

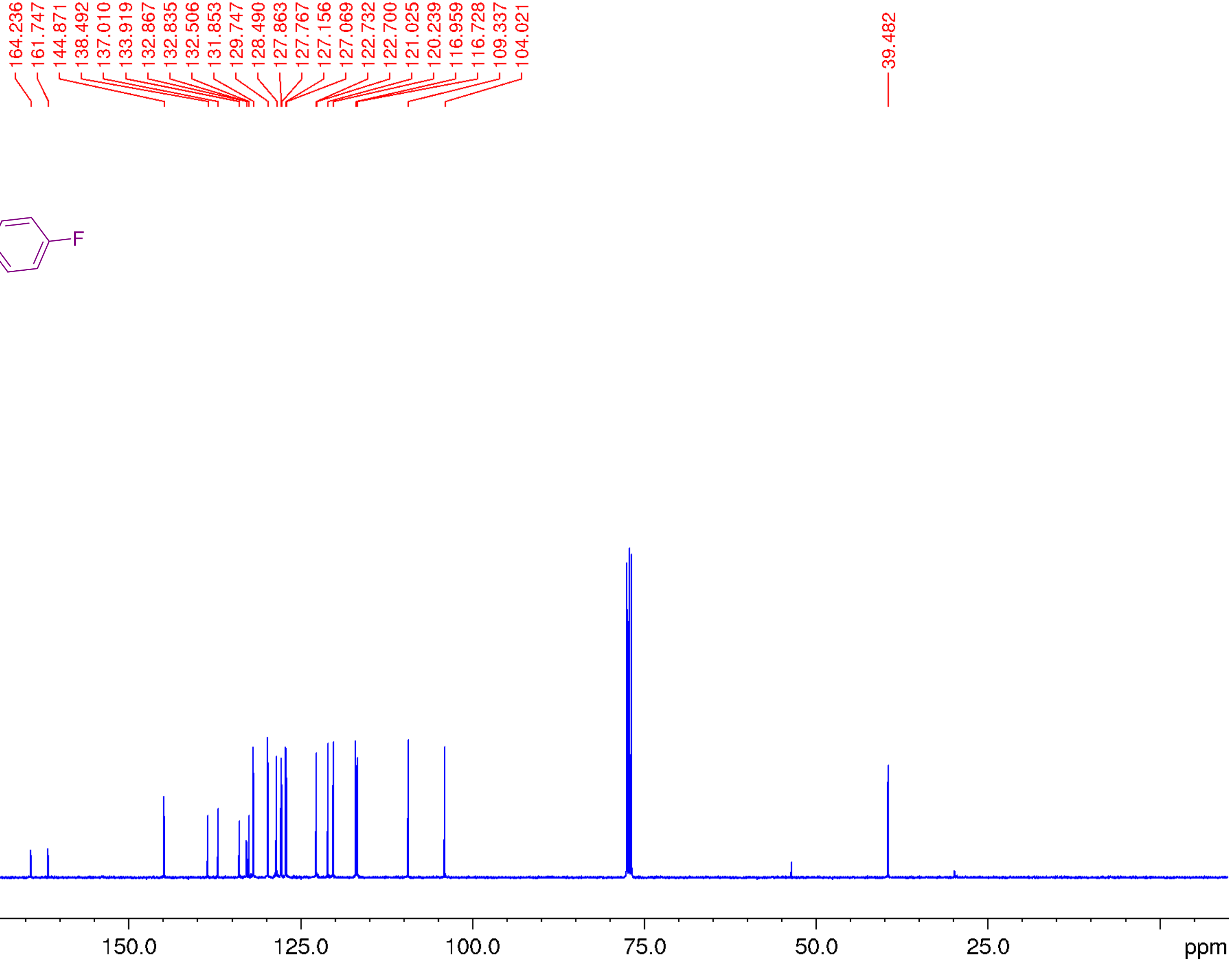

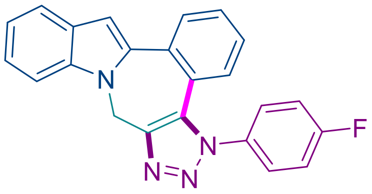

3c

DEPT 135 NMR-spectrum ($CDCl_3$)

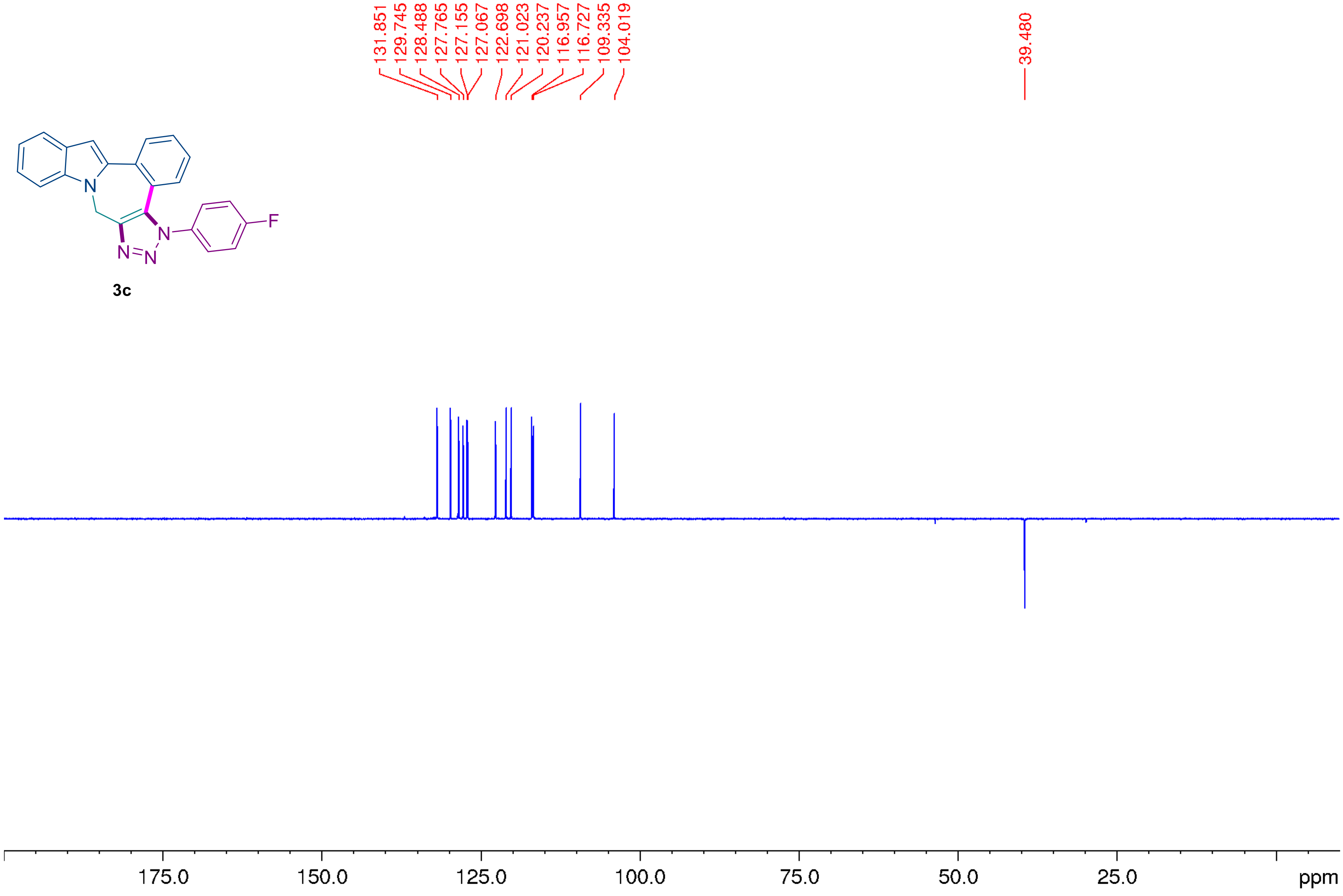

^{19}F NMR-spectrum (376.5 Hz, $CDCl_3$)

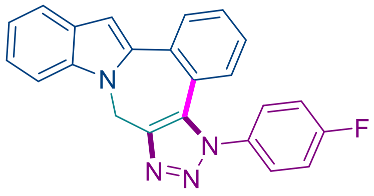

3c

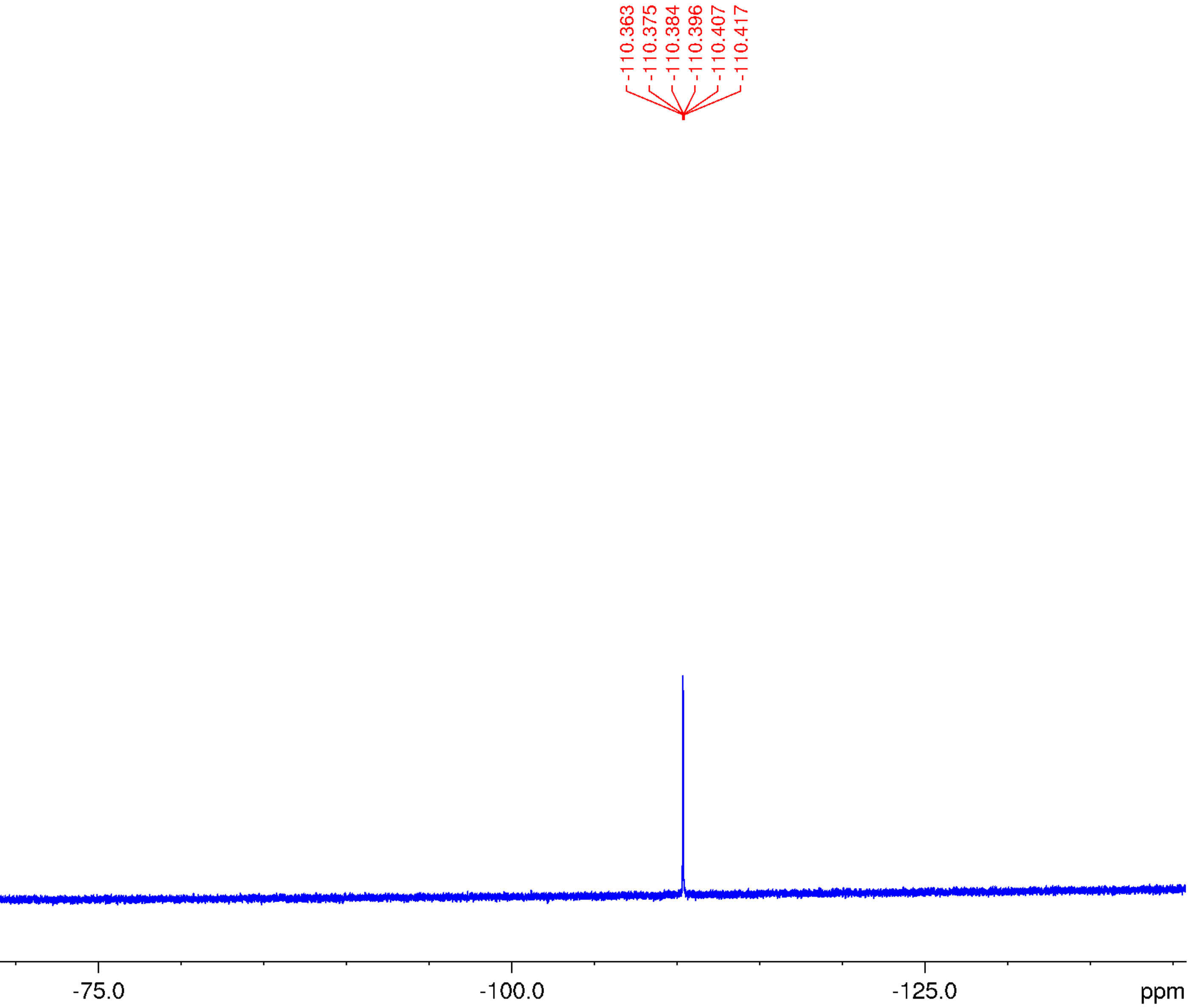

^{1}H NMR-spectrum (400 MHz, $CDCl_3$)

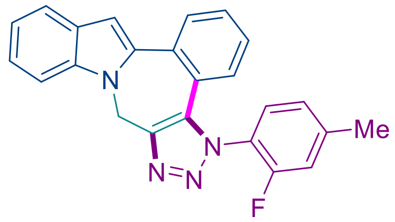

3d

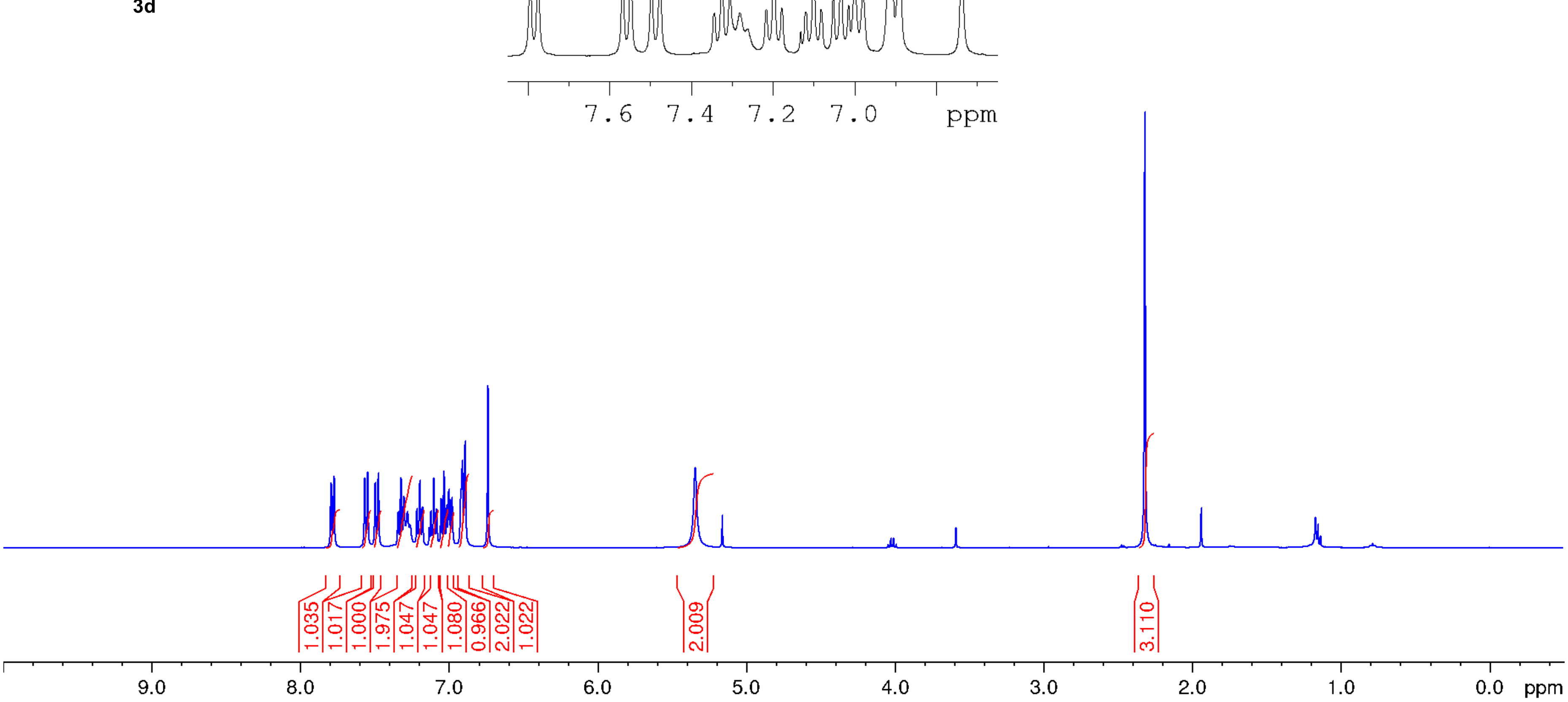

^{13}C NMR-spectrum (100 MHz, $CDCl_3$)

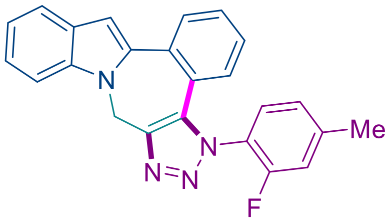

3d

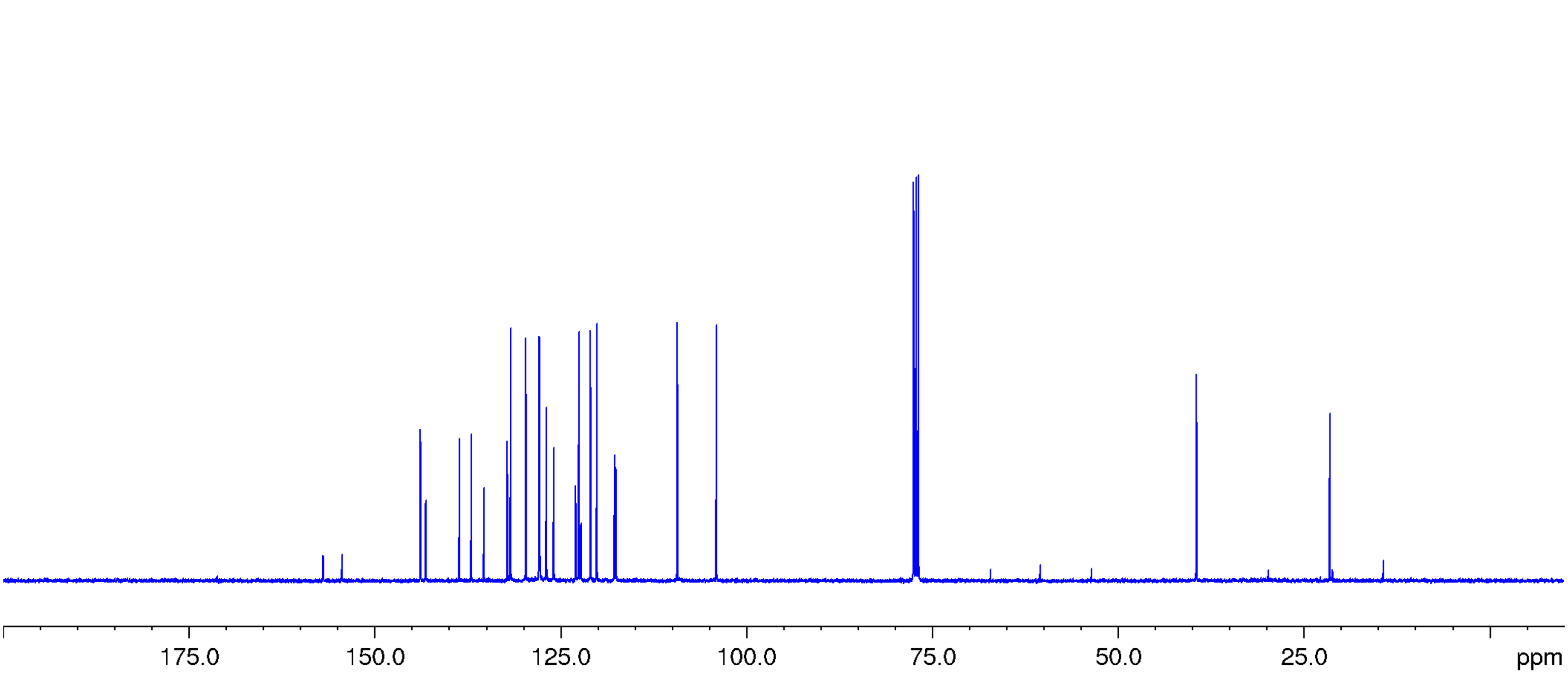

DEPT 135 NMR-spectrum ($CDCl_3$)

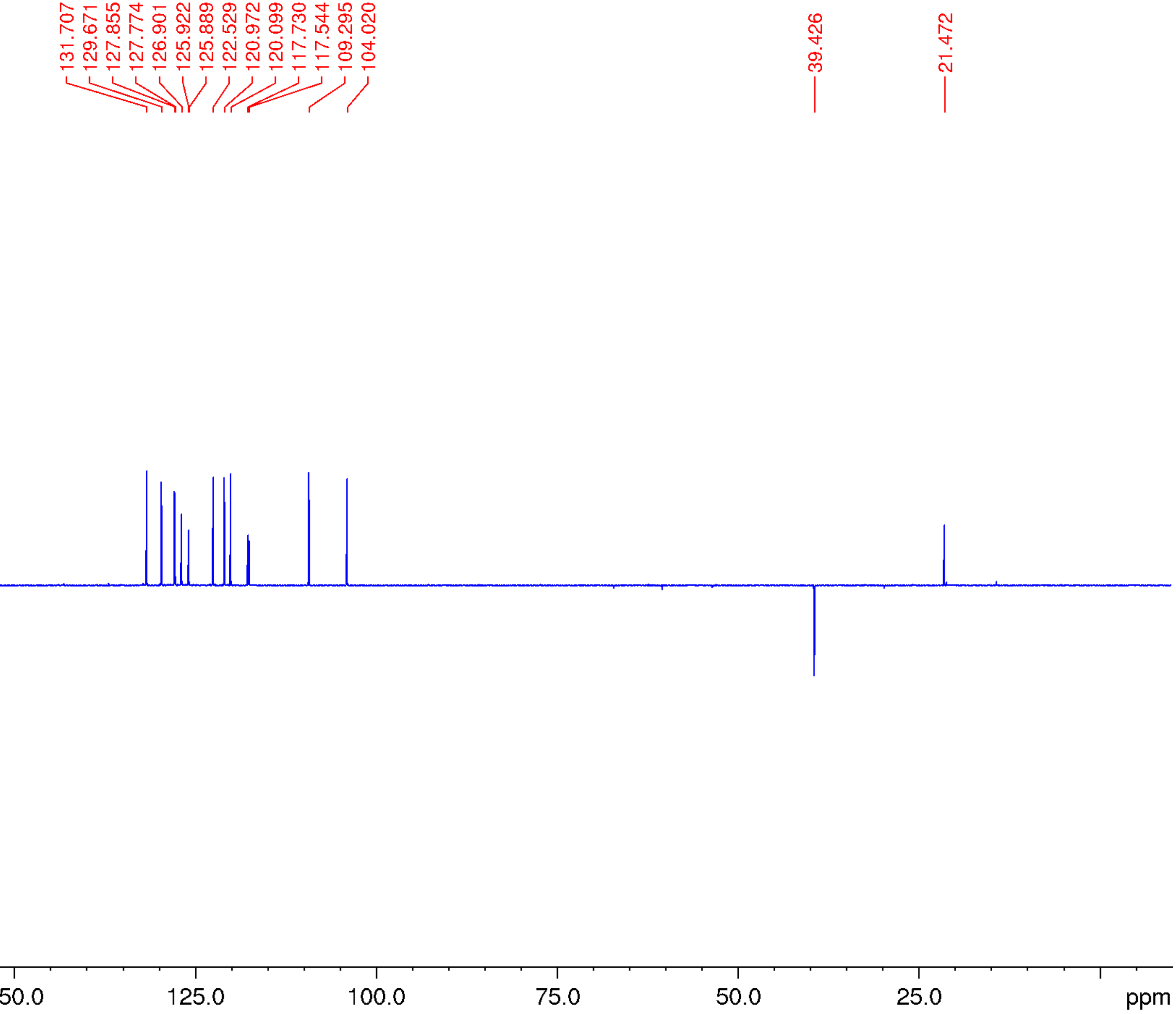

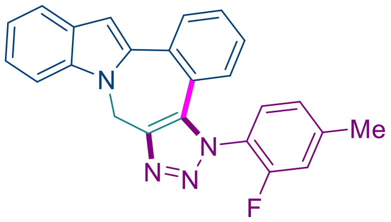

3d

^{19}F NMR-spectrum (376.5 Hz, $CDCl_3$)

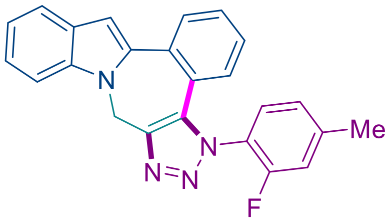

3d

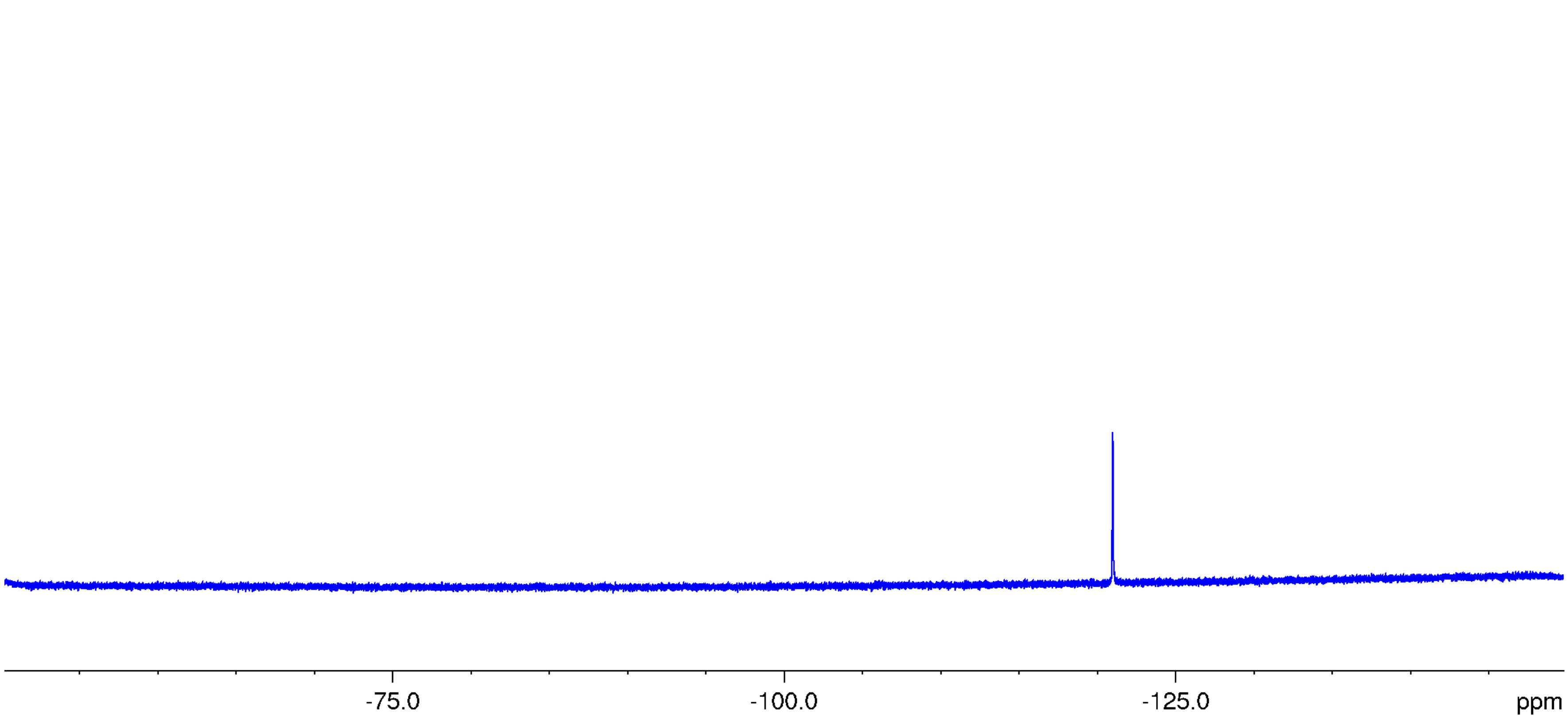

^{1}H NMR-spectrum (400 MHz, $CDCl_3$)

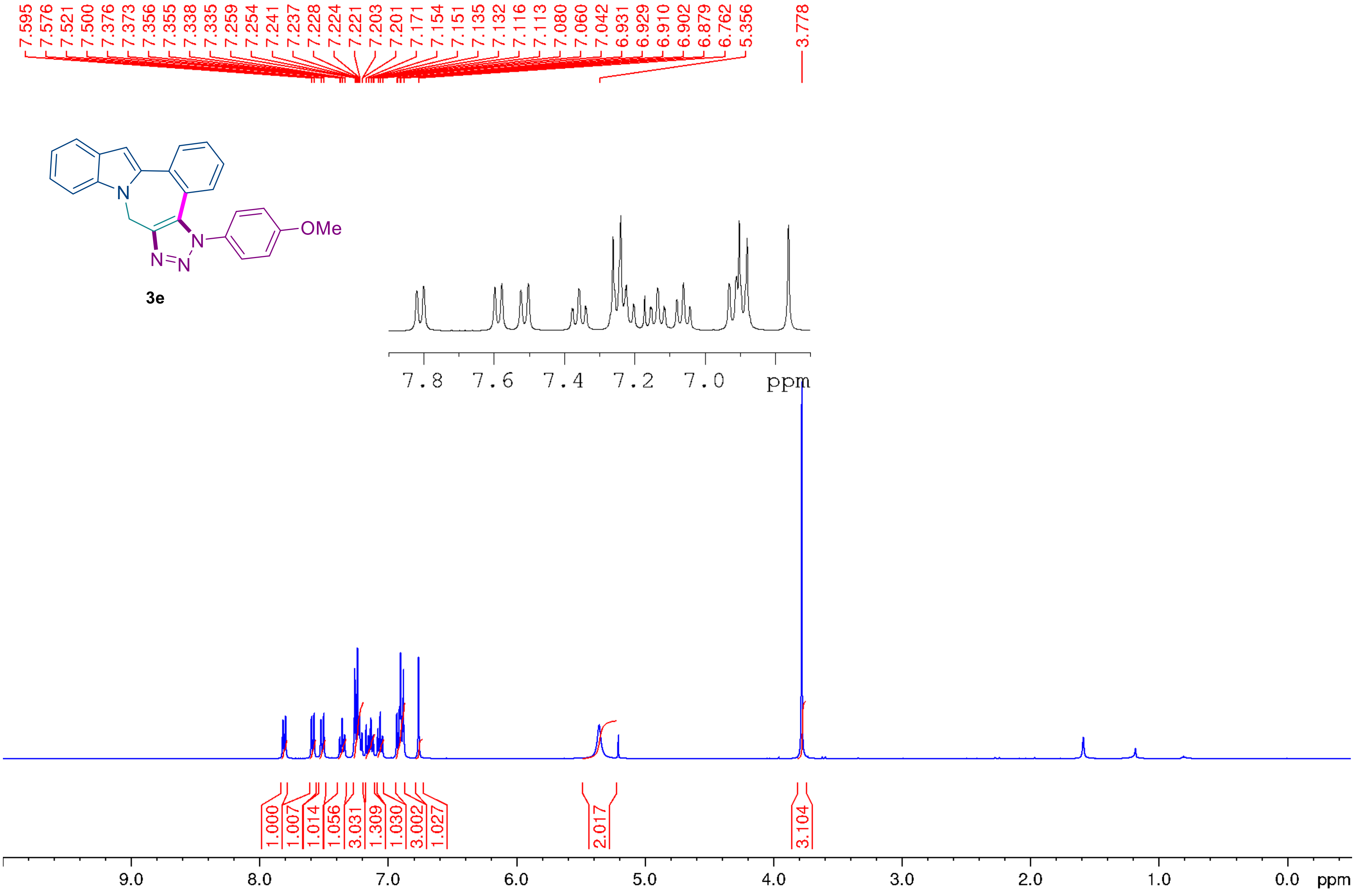

^{13}C NMR-spectrum (100 MHz, $CDCl_3$)

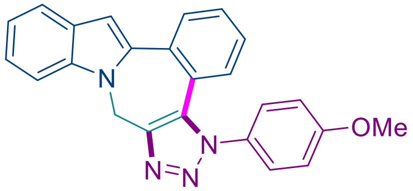

3e

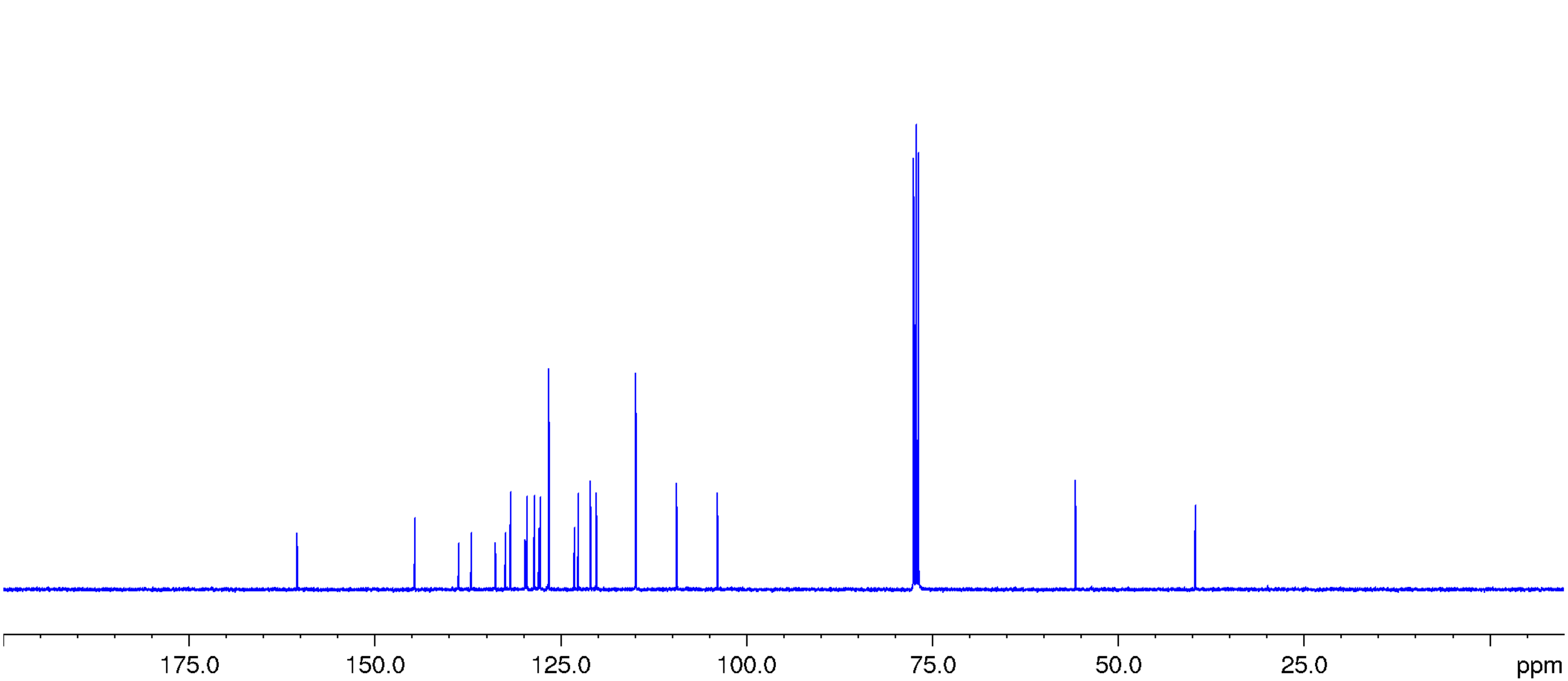

DEPT 135 NMR-spectrum ($CDCl_3$)

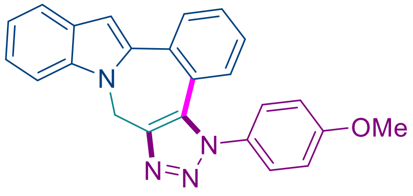

3e

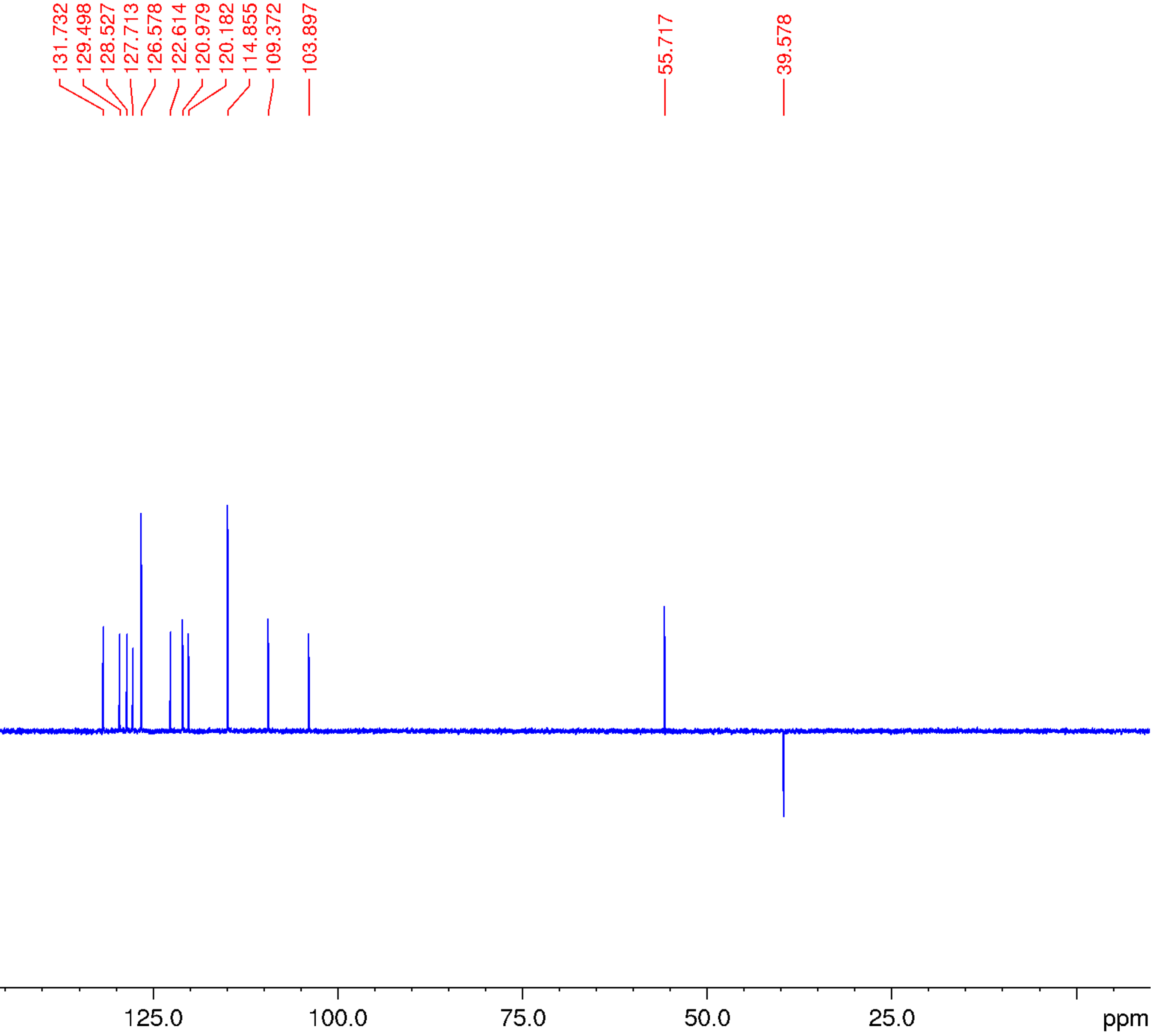

^{1}H NMR-spectrum (400 MHz, $CDCl_3$)

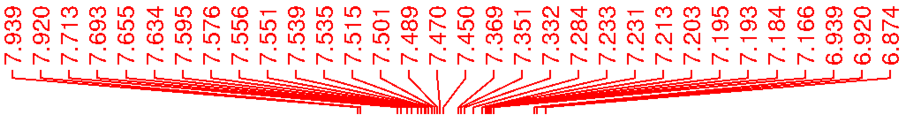

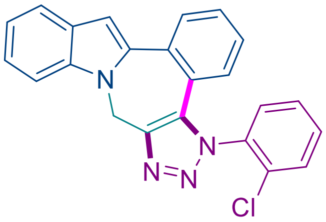

3f

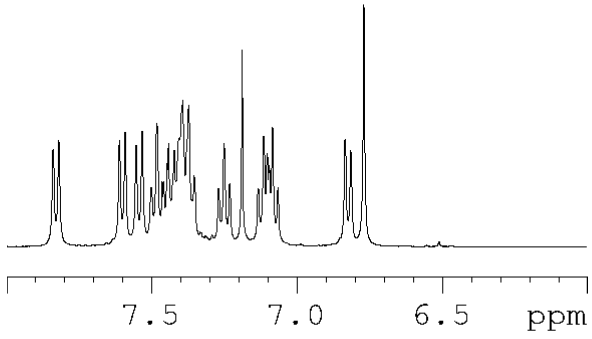

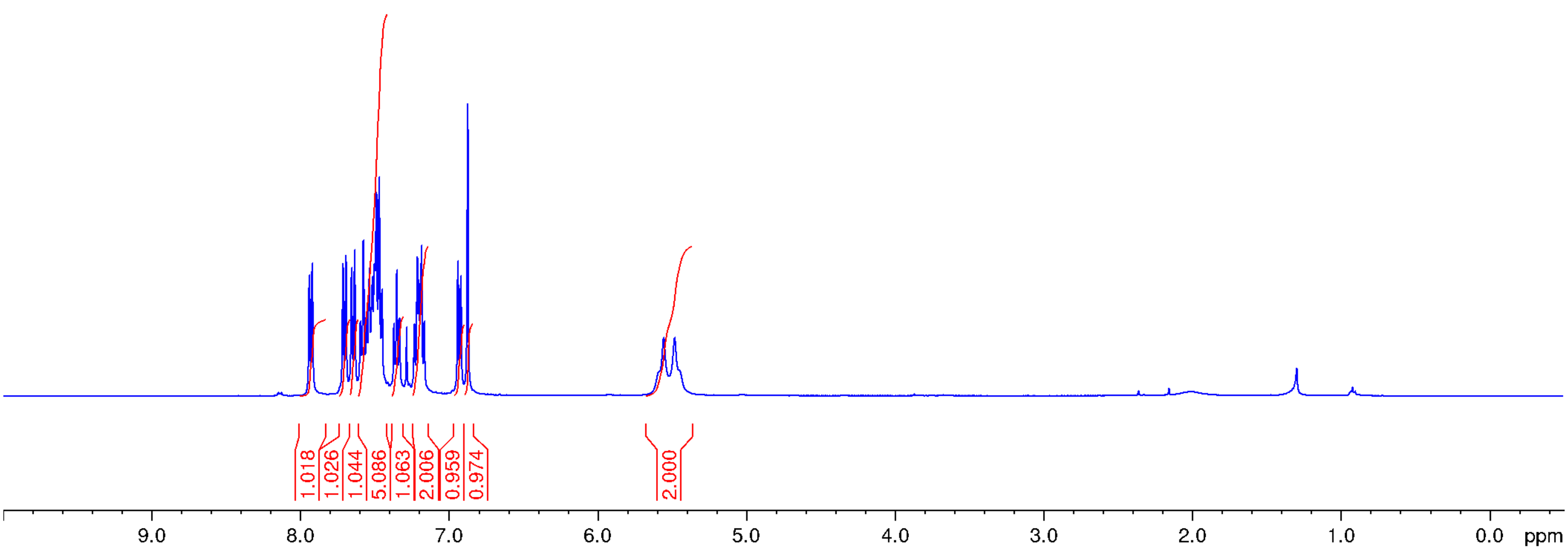

^{13}C NMR-spectrum (100 MHz, $CDCl_3$)

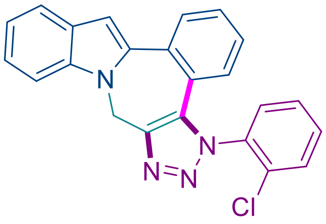

3f

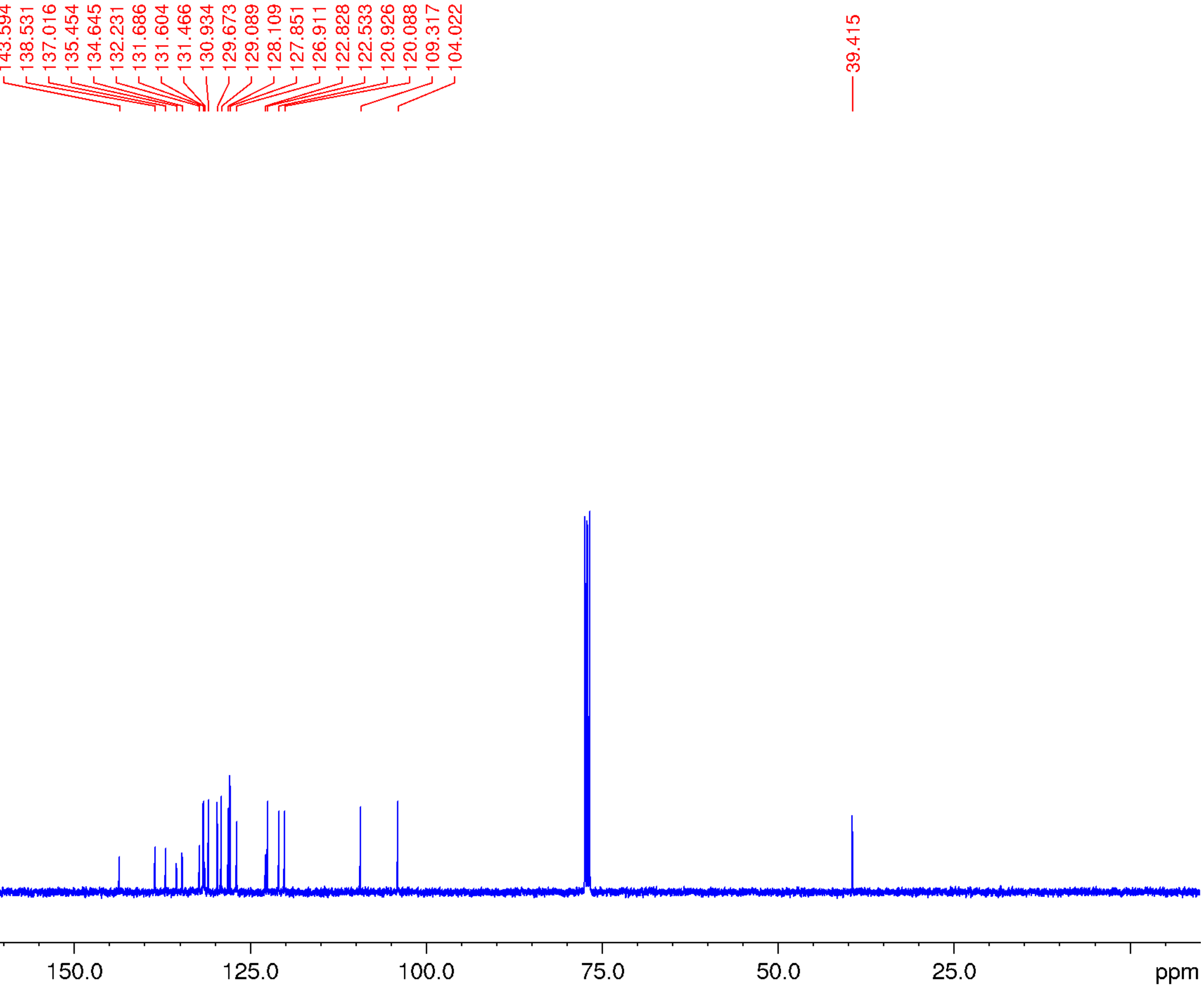

DEPT 135 NMR-spectrum ($CDCl_3$)

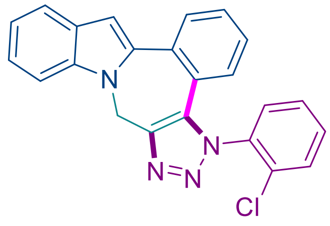

3f

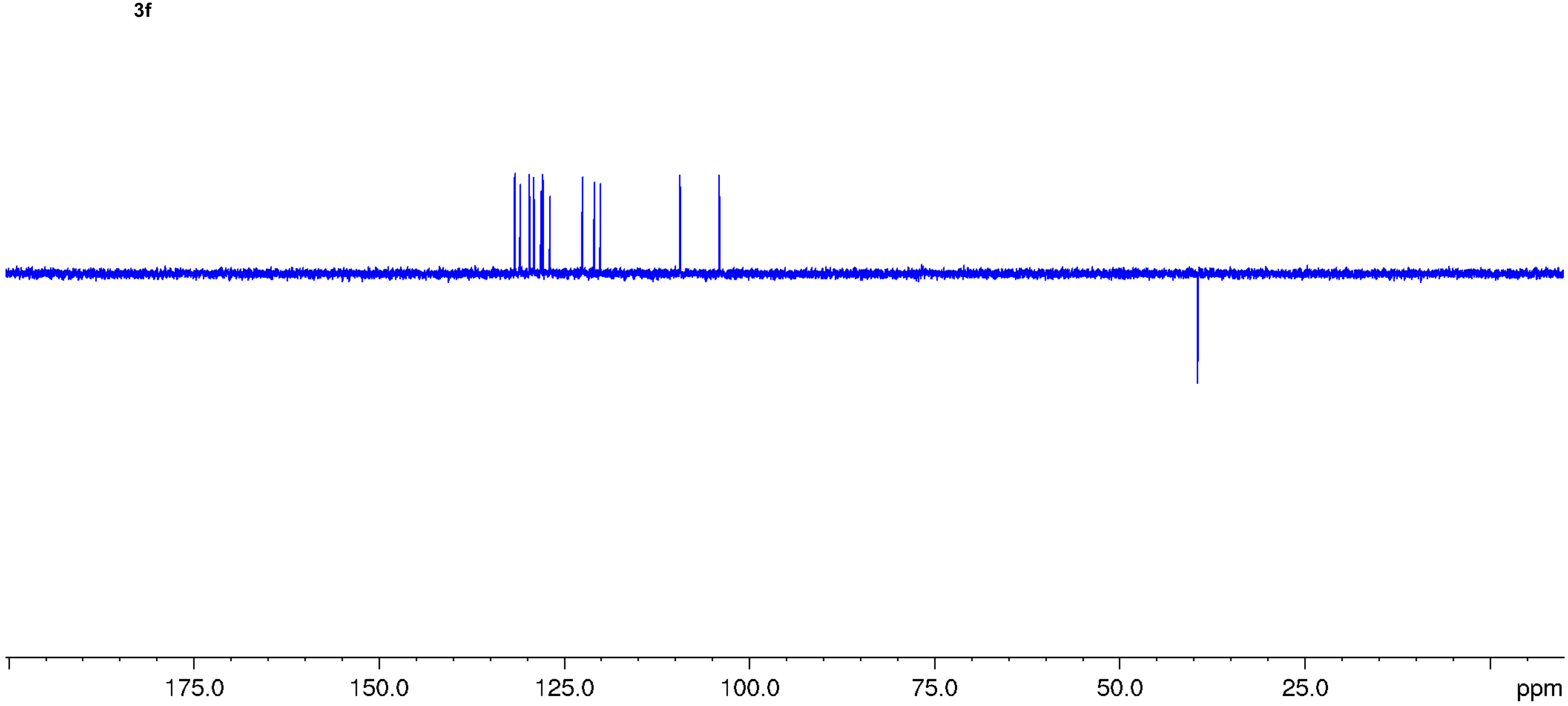

^{1}H NMR-spectrum (400 MHz, $CDCl_3$)

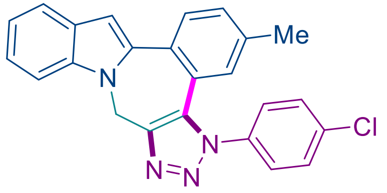

3g

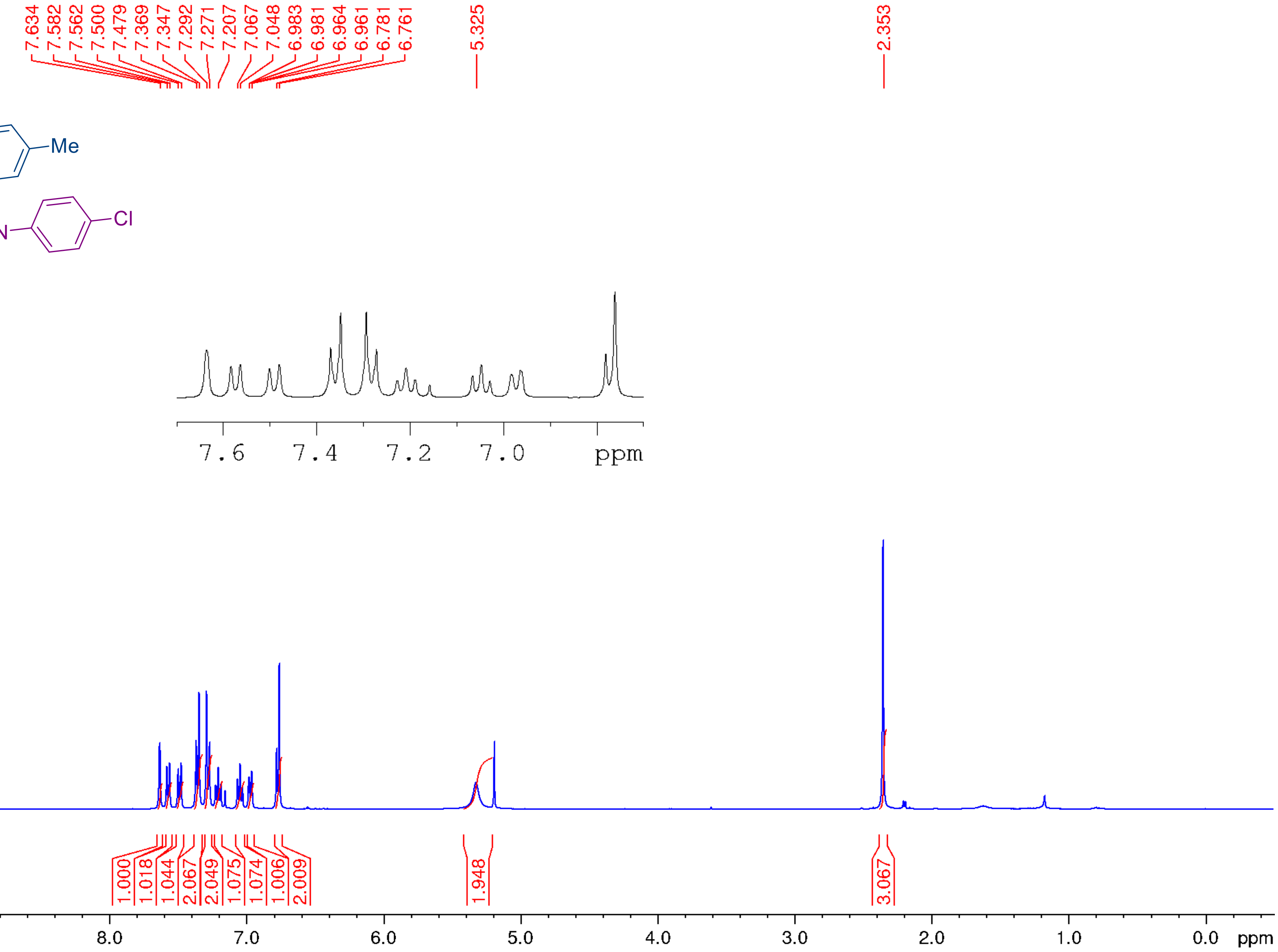

^{13}C NMR-spectrum (100 MHz, $CDCl_3$)

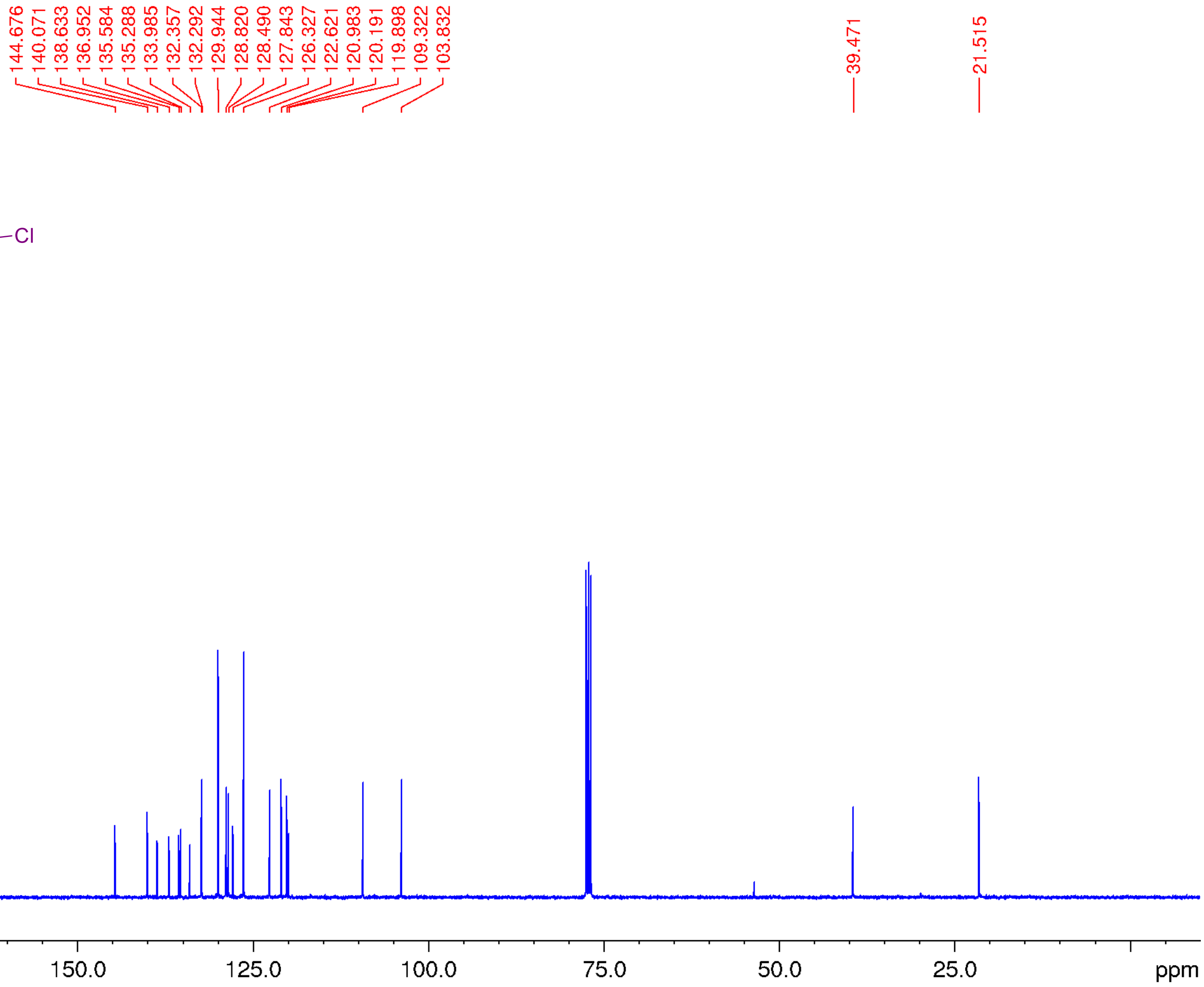

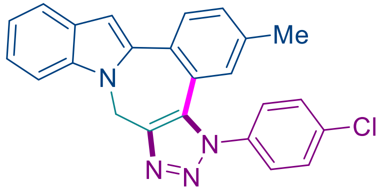

3g

DEPT 135 NMR-spectrum ($CDCl_3$)

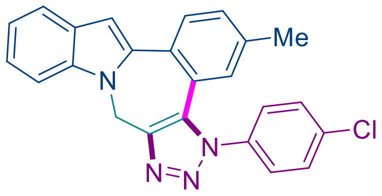

3g

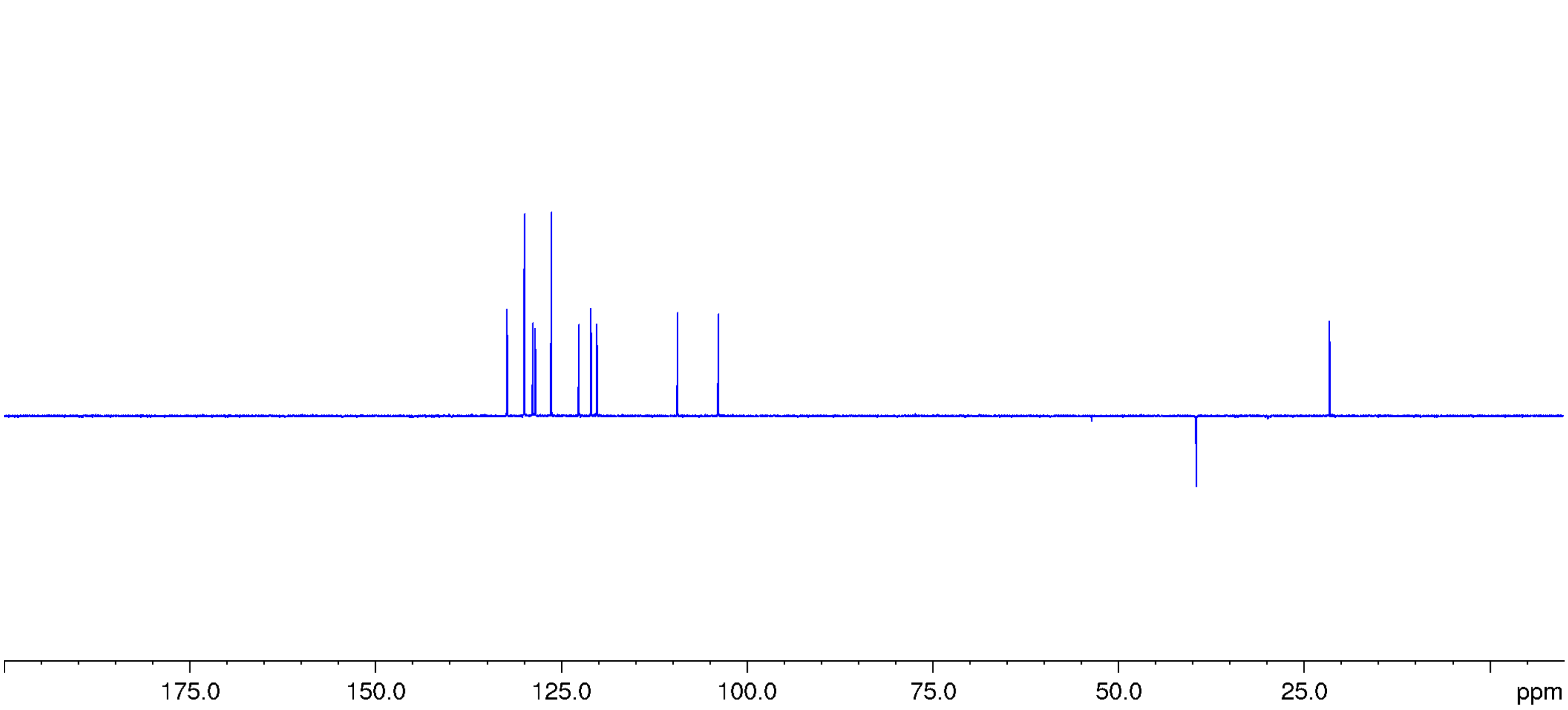

^{1}H NMR-spectrum (400 MHz, $CDCl_3$)

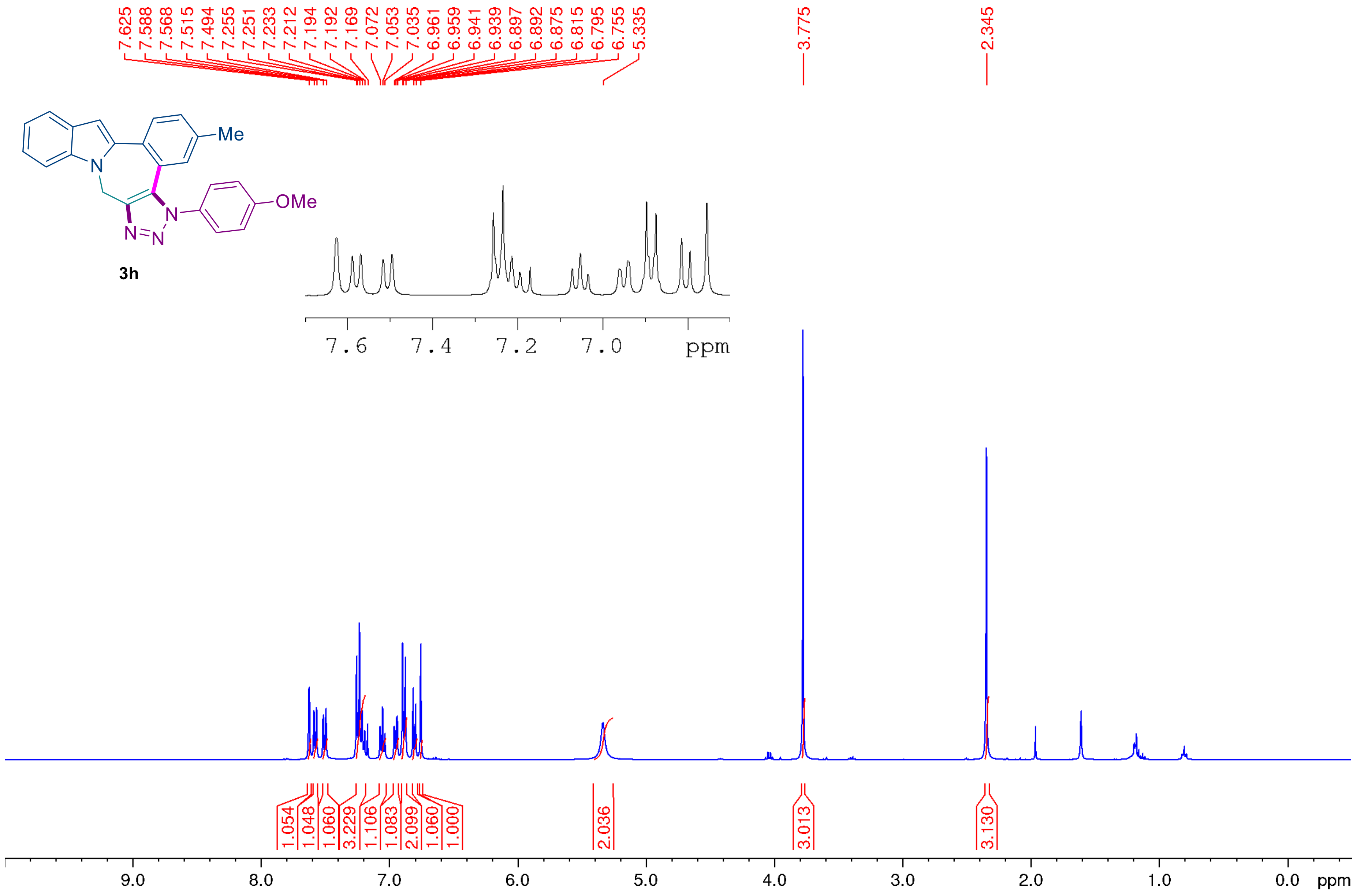

^{13}C NMR-spectrum (100 MHz, $CDCl_3$)

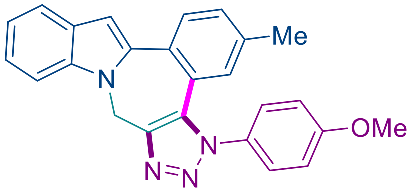

3h

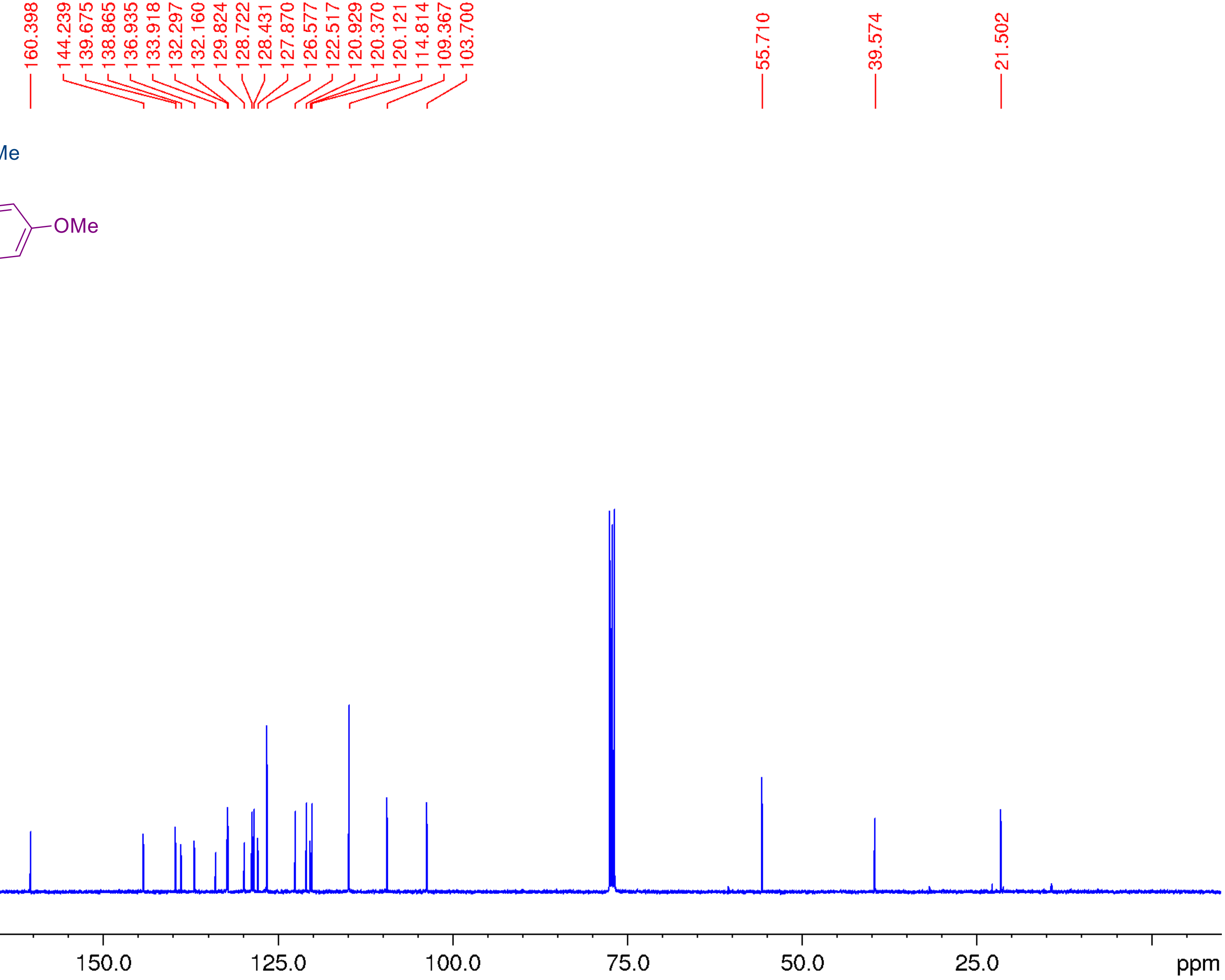

DEPT 135 NMR-spectrum ($CDCl_3$)

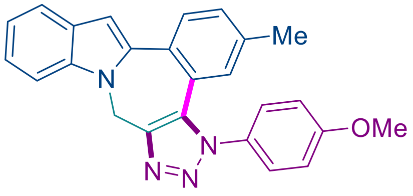

3h

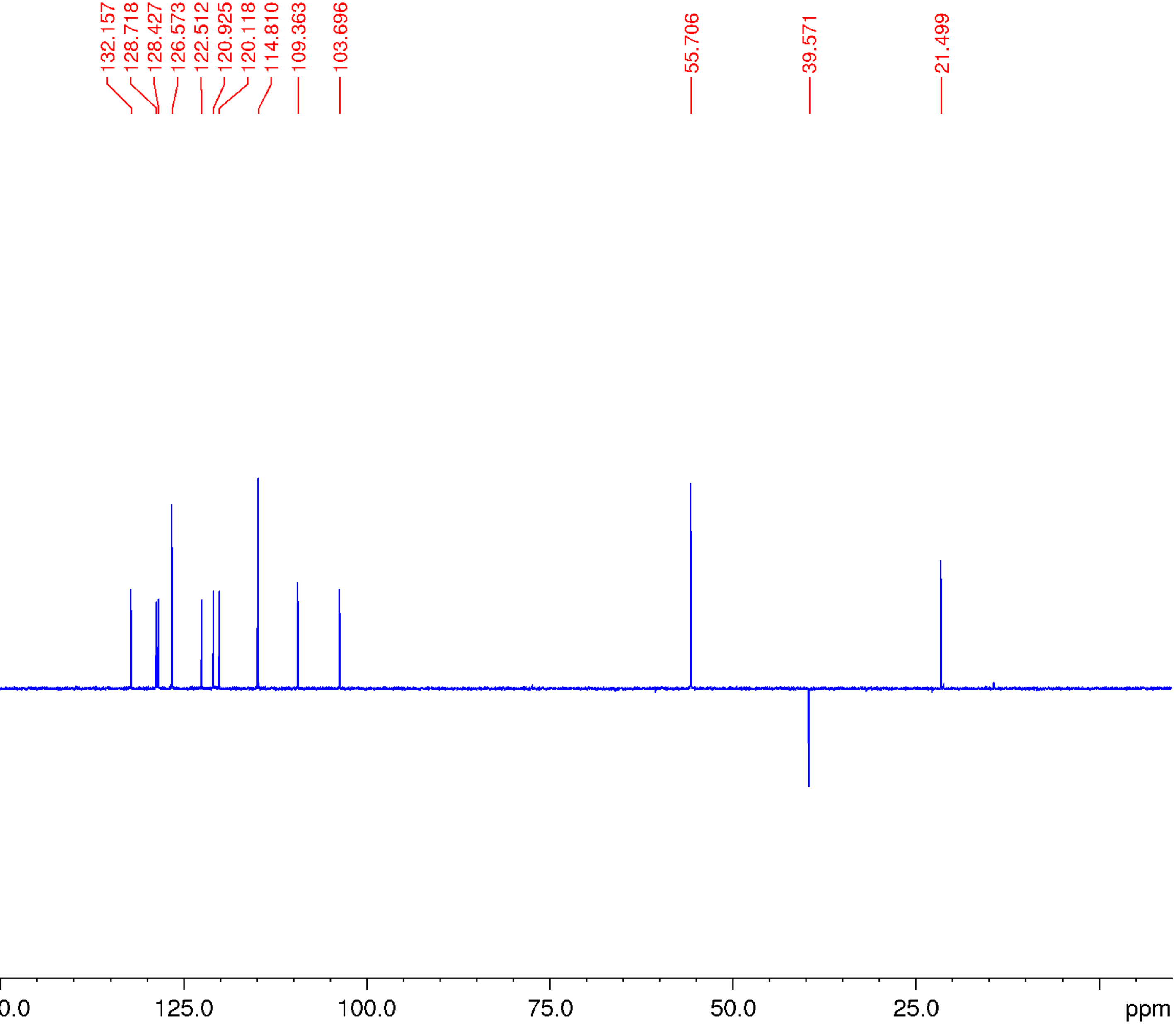

^{1}H NMR-spectrum (400 MHz, $CDCl_3$)

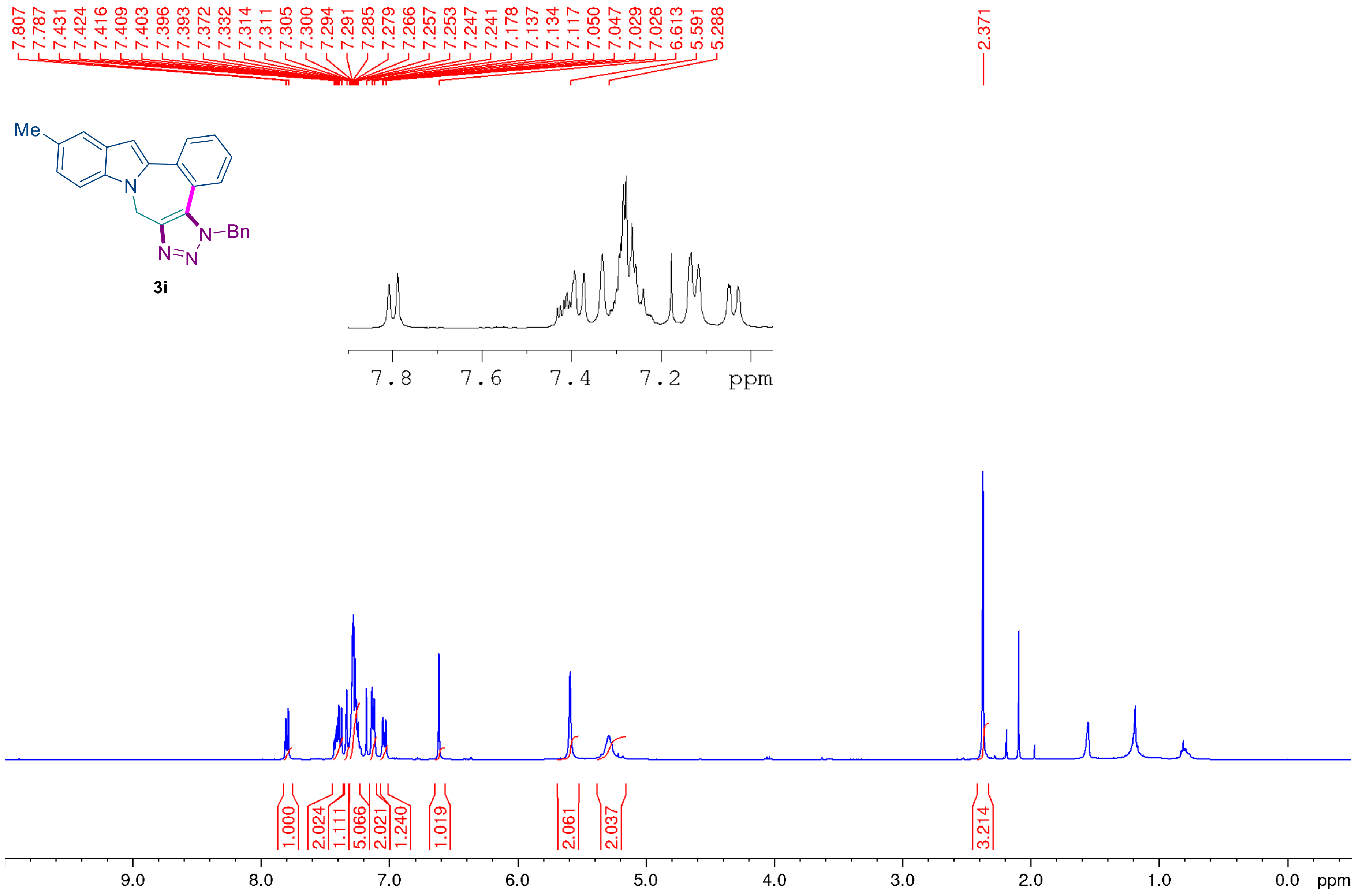

^{13}C NMR-spectrum (100 MHz, $CDCl_3$)

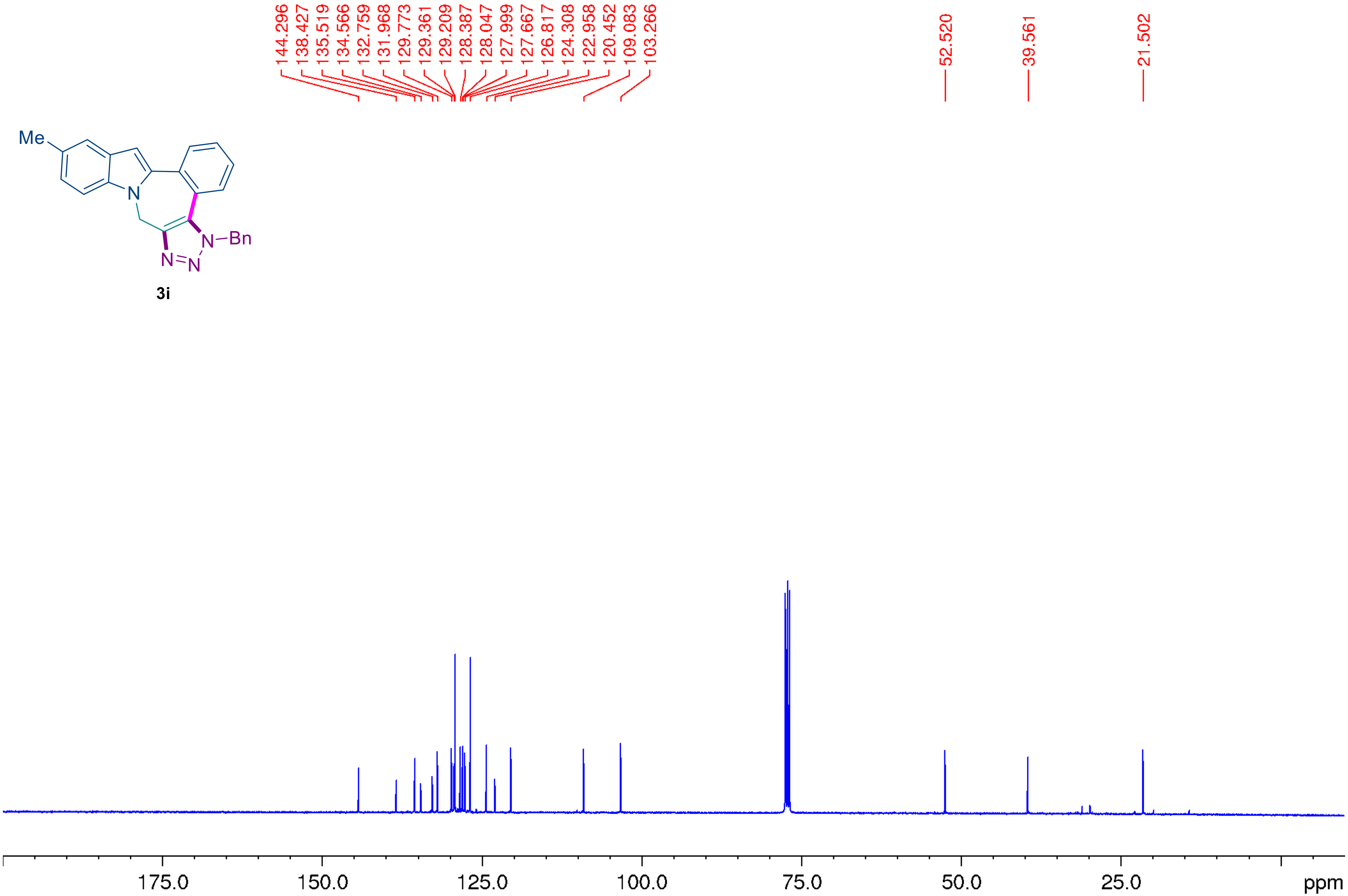

DEPT 135 NMR-spectrum ($CDCl_3$)

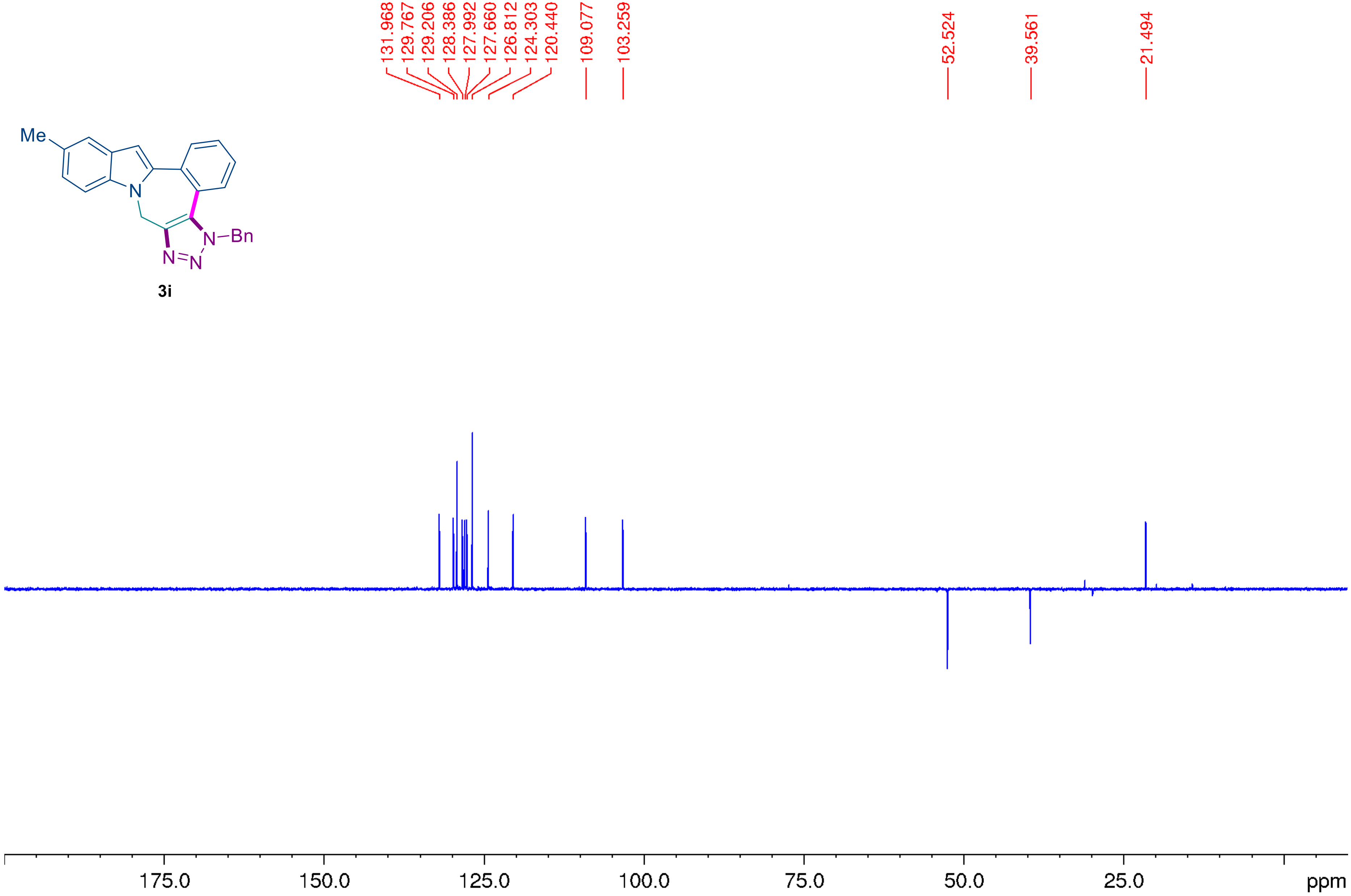

^{1}H NMR-spectrum (400 MHz, $CDCl_3$)

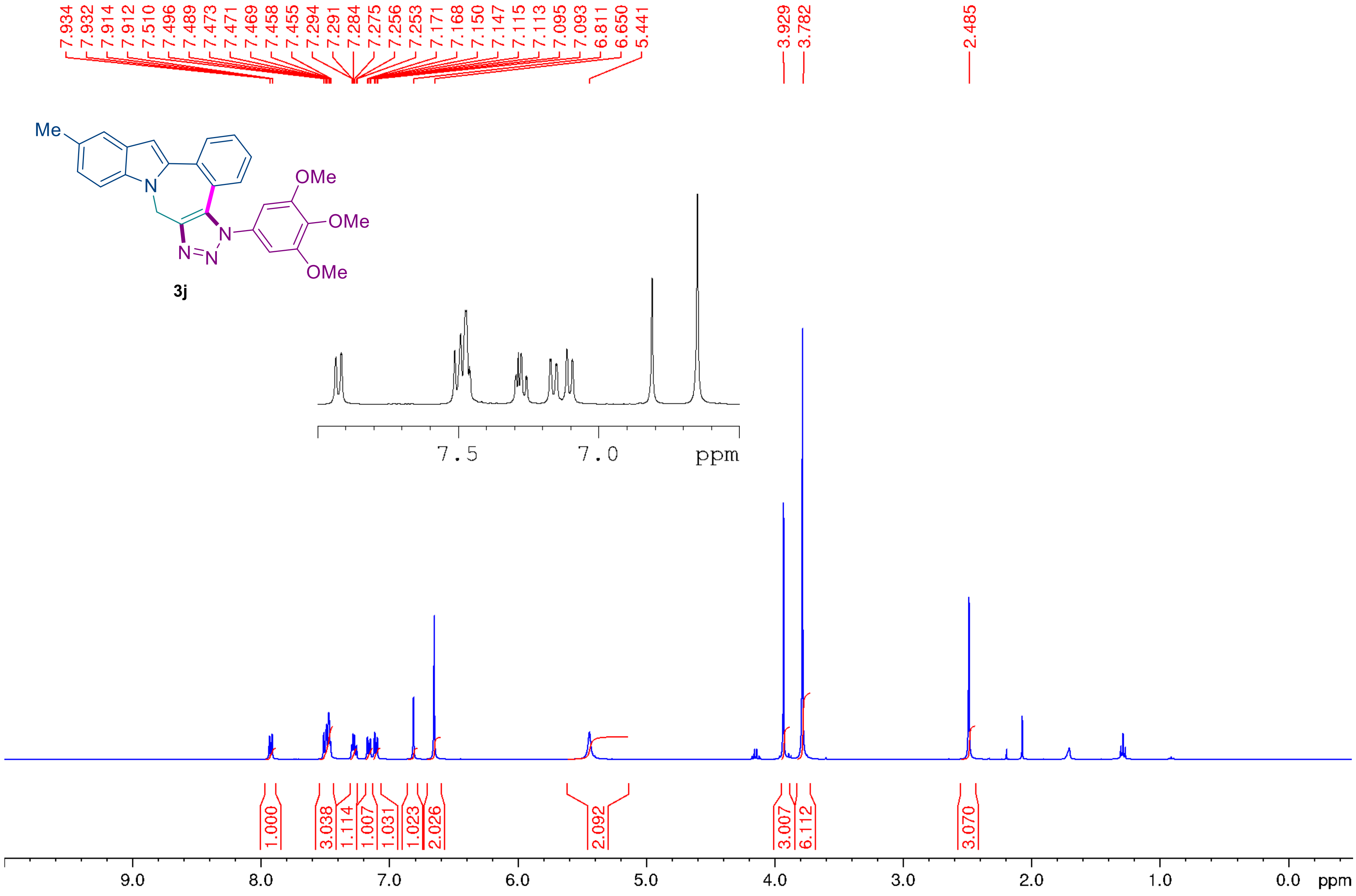

^{13}C NMR-spectrum (100 MHz, $CDCl_3$)

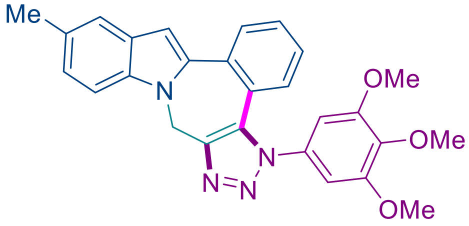

3j

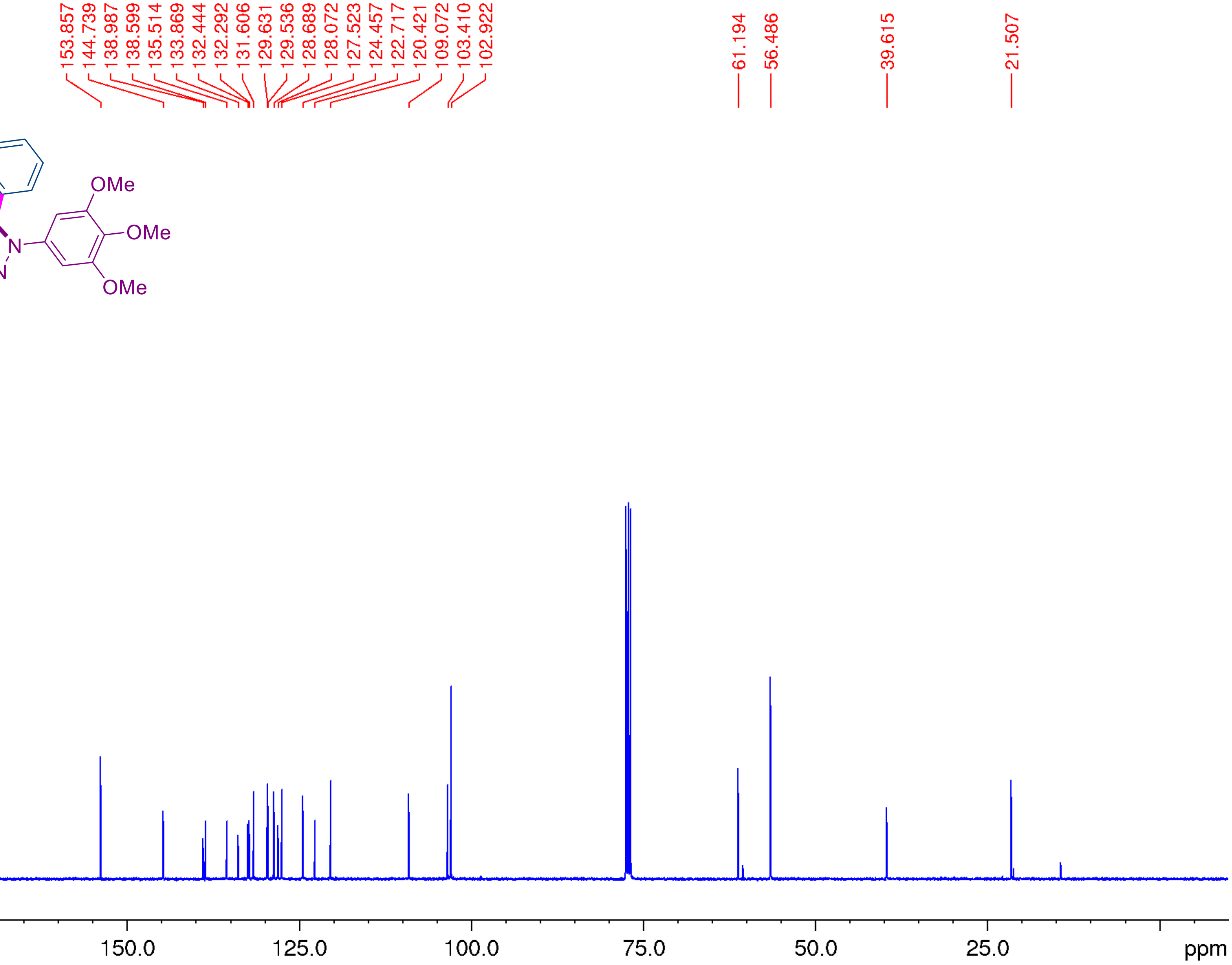

DEPT 135 NMR-spectrum ($CDCl_3$)

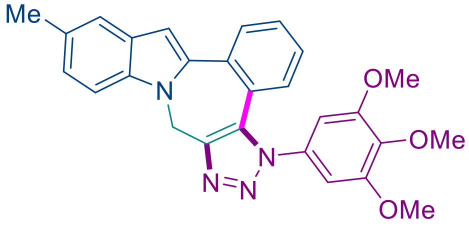

3j

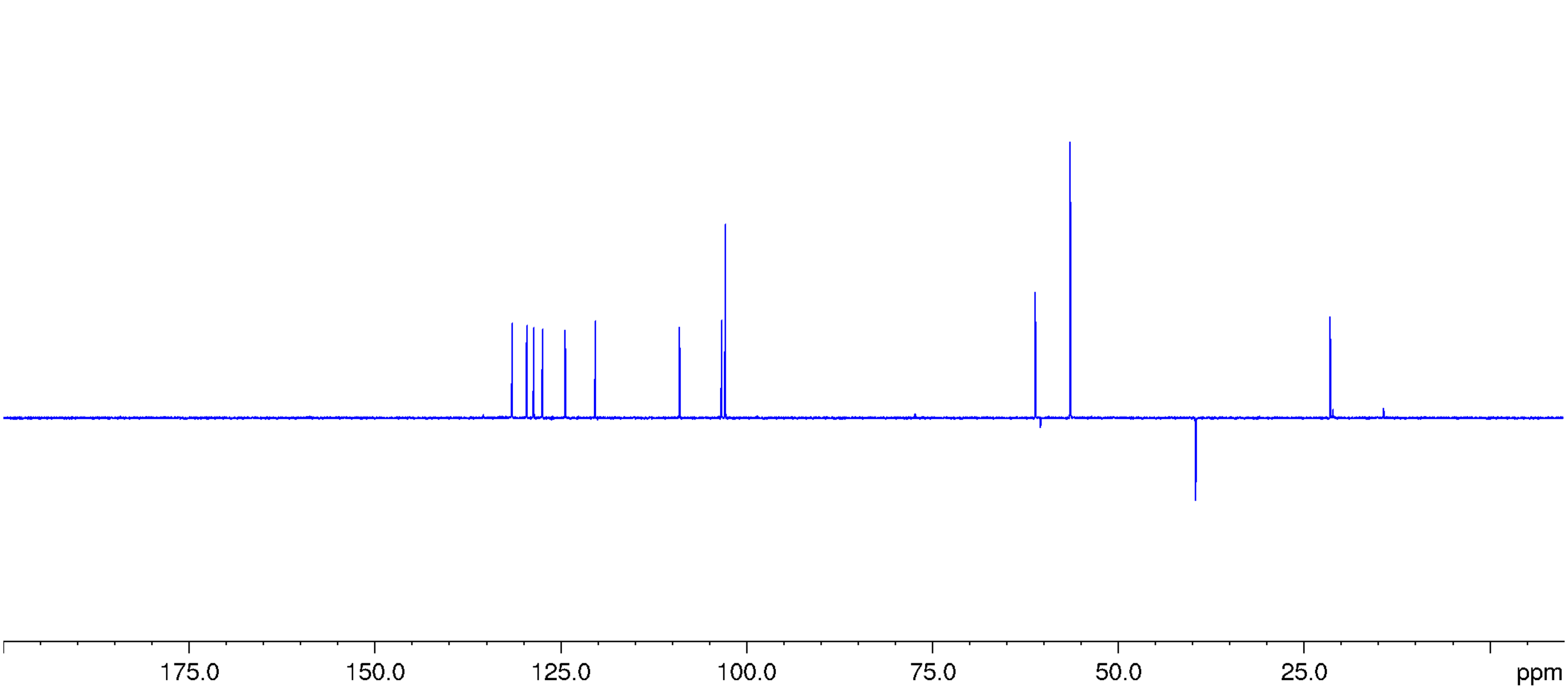

^{1}H NMR-spectrum (400 MHz, $CDCl_3$)

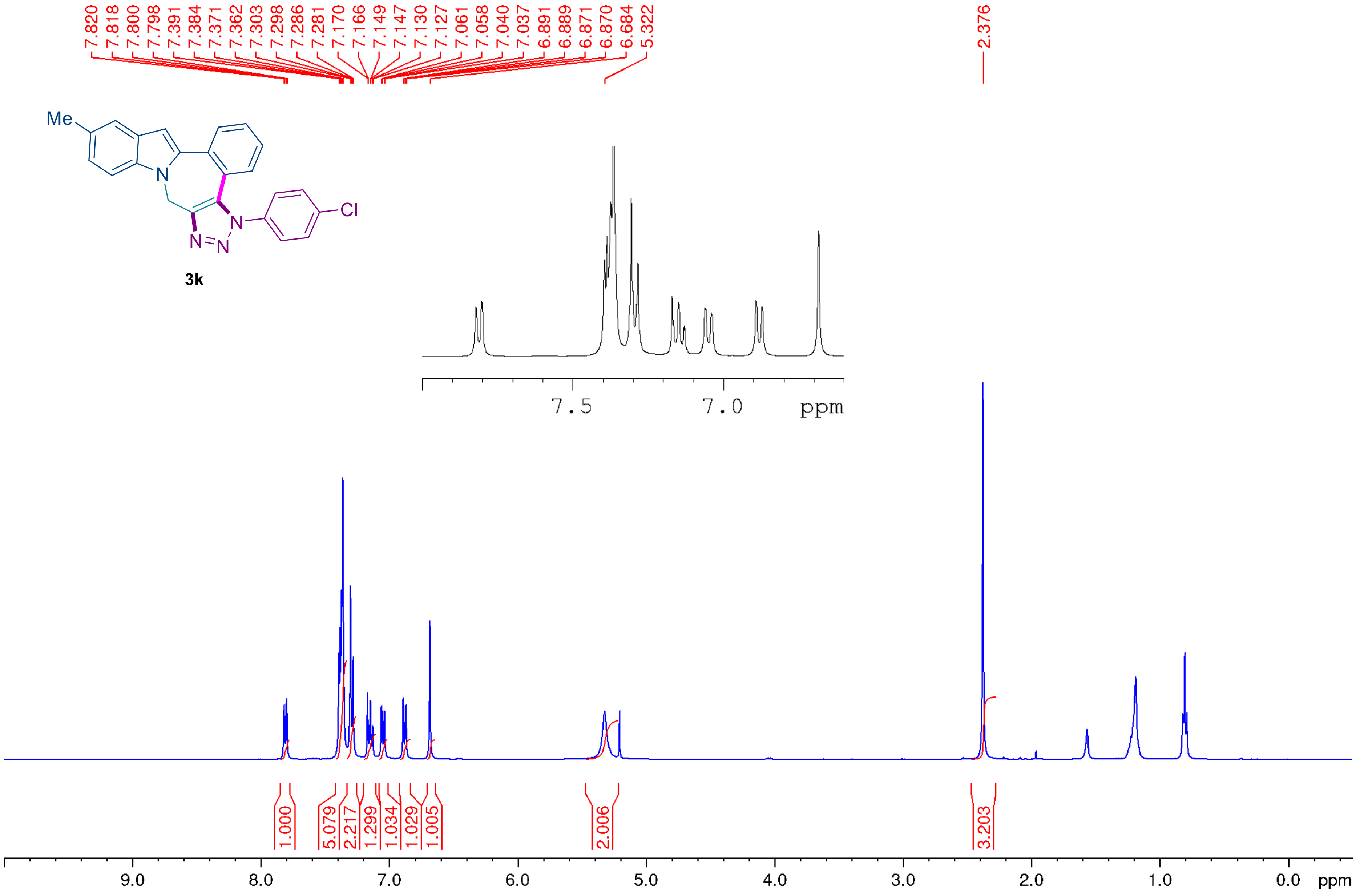

^{13}C NMR-spectrum (100 MHz, $CDCl_3$)

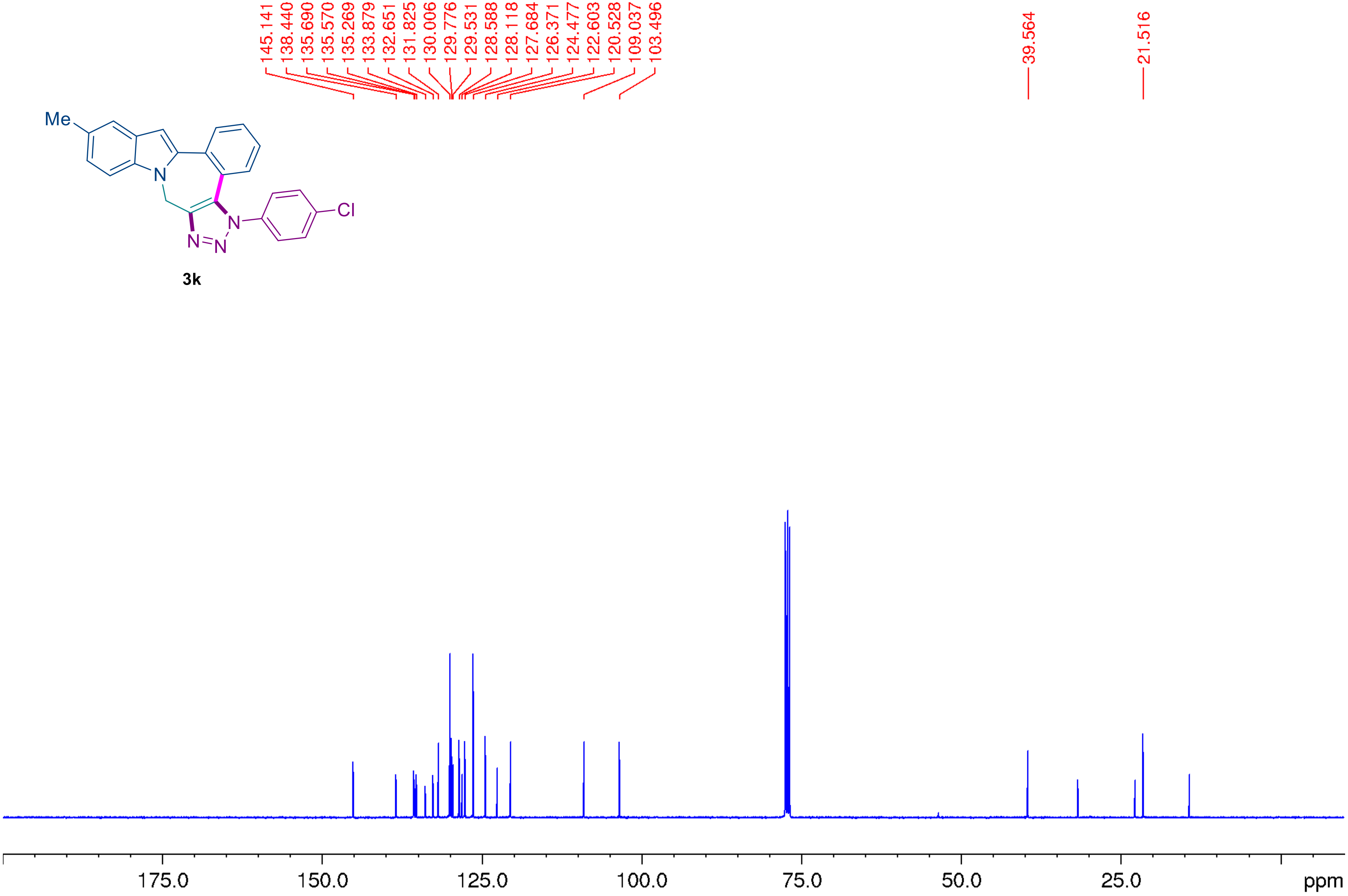

DEPT 135 NMR-spectrum ($CDCl_3$)

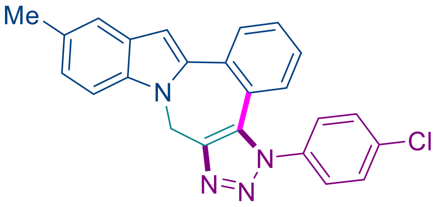

3k

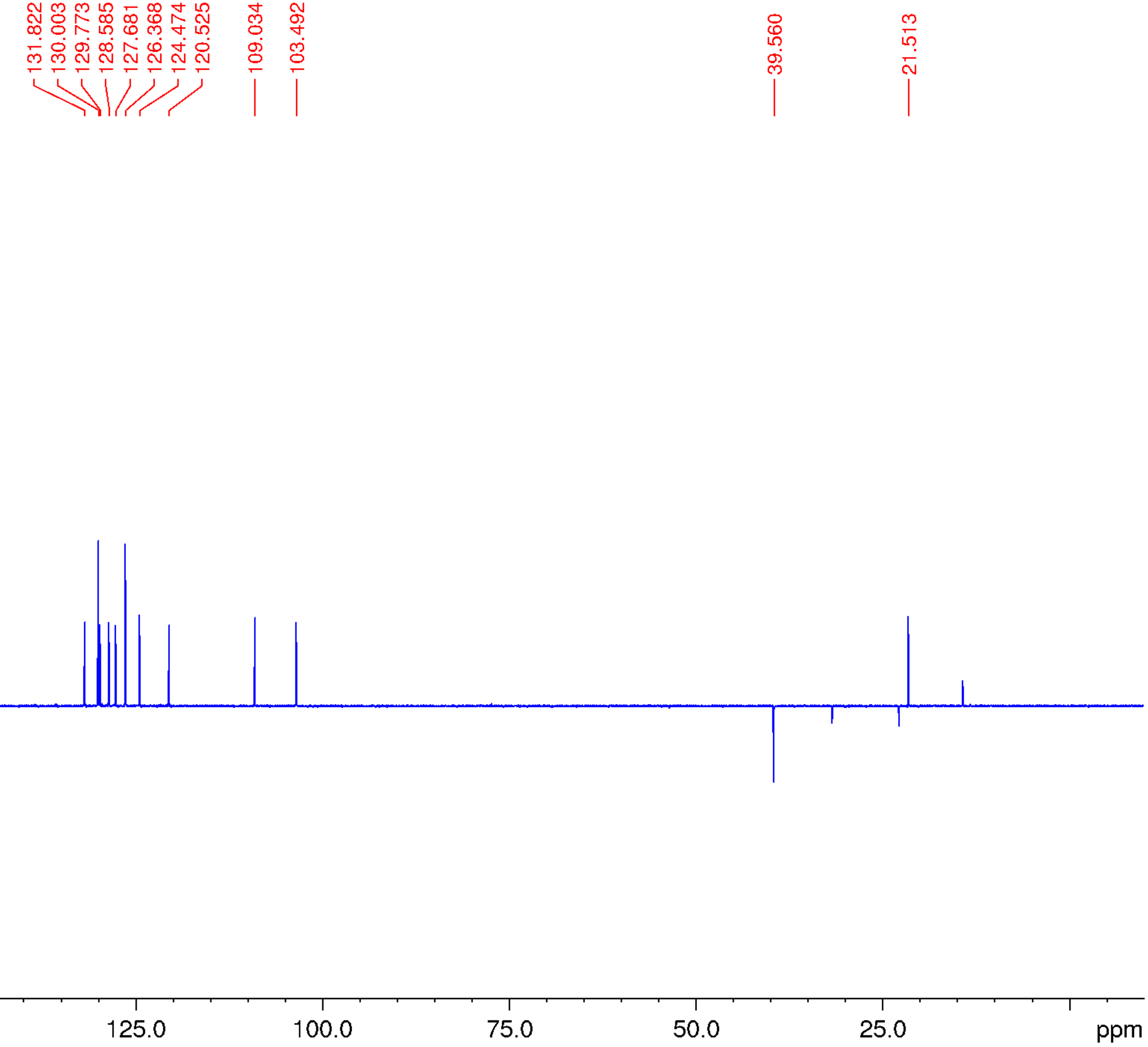

^{1}H NMR-spectrum (400 MHz, $CDCl_3$)

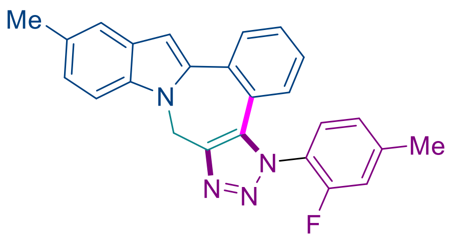

3l

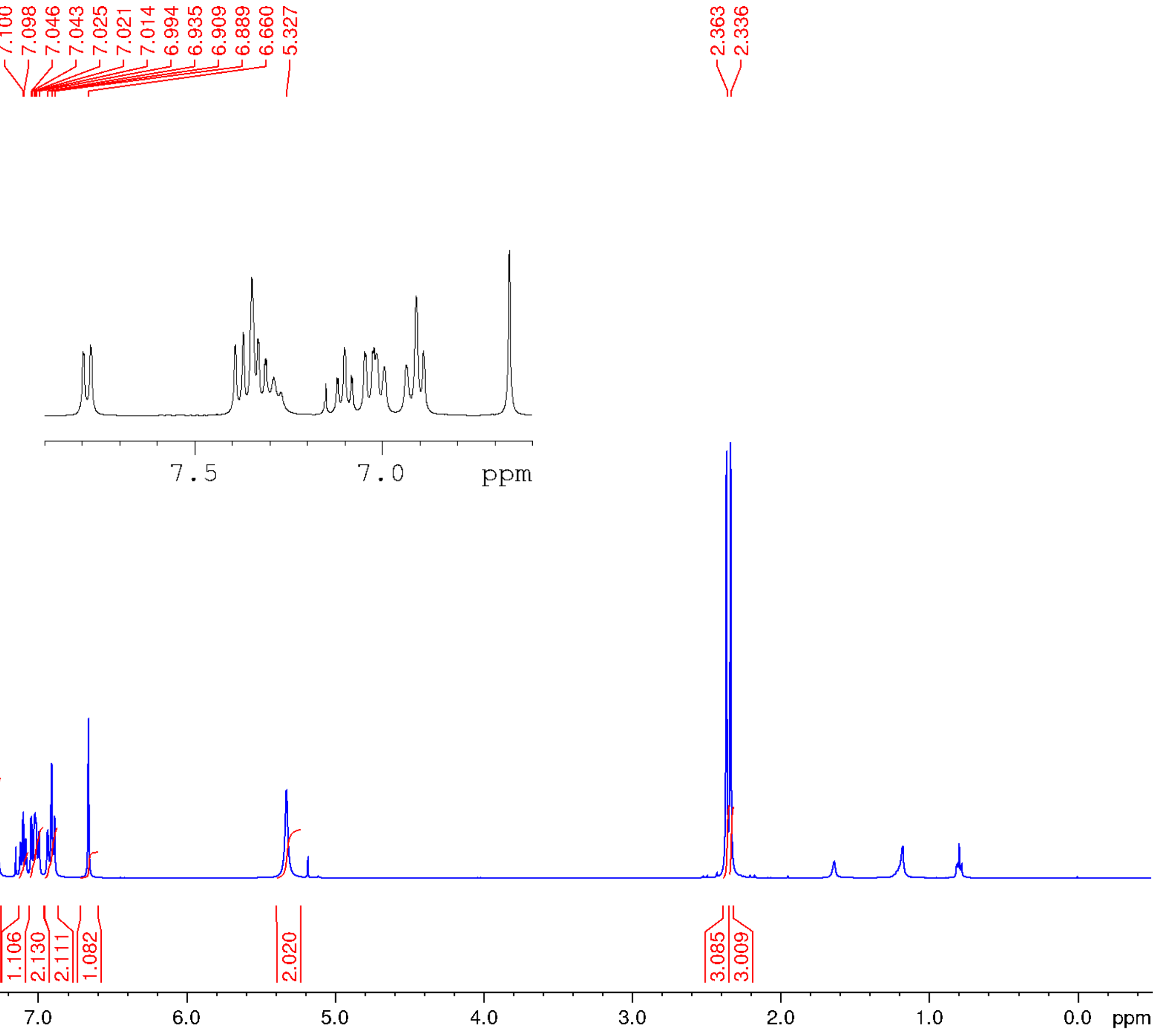

^{13}C NMR-spectrum (100 MHz, $CDCl_3$)

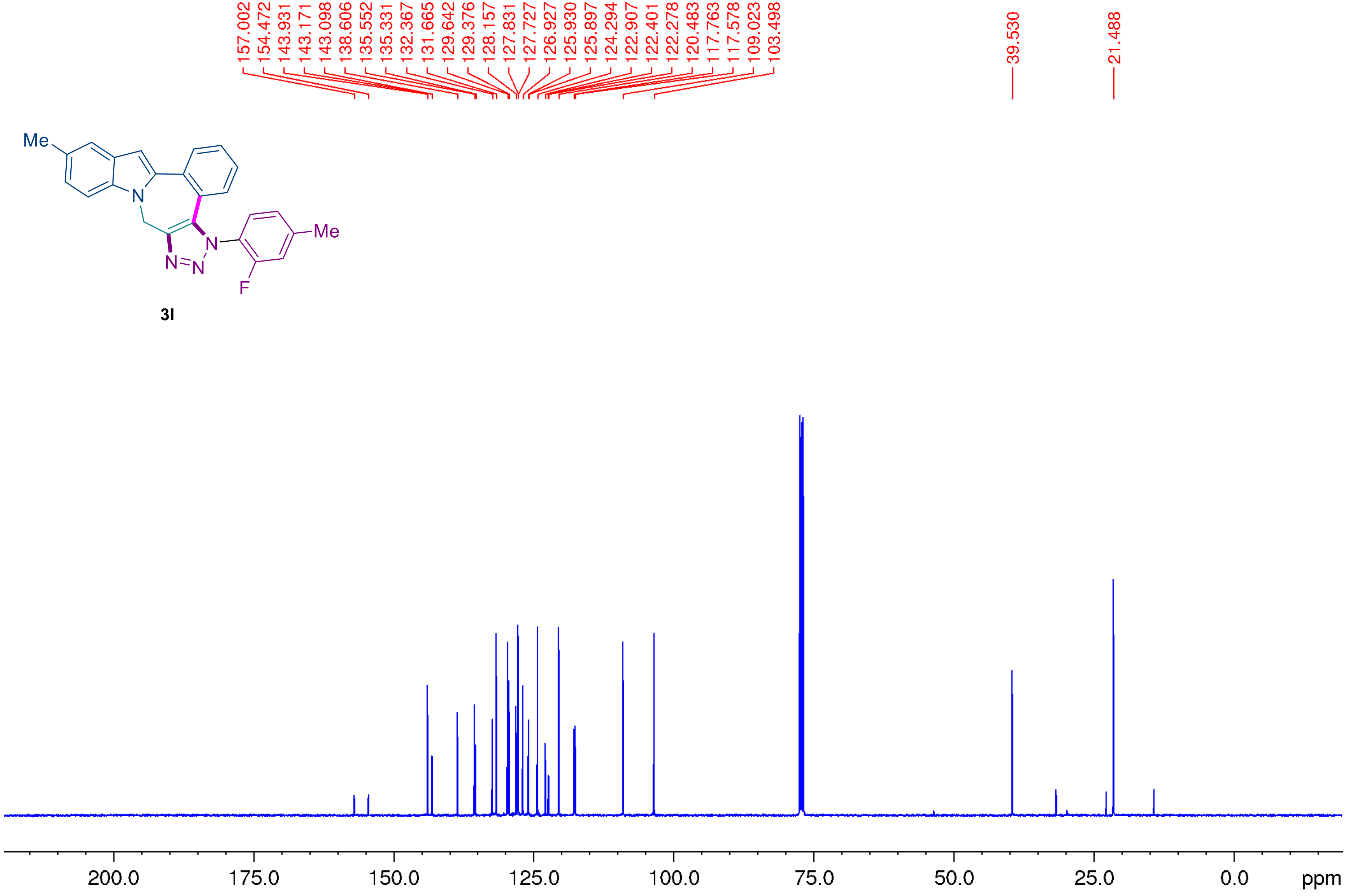

DEPT 135 NMR-spectrum ($CDCl_3$)

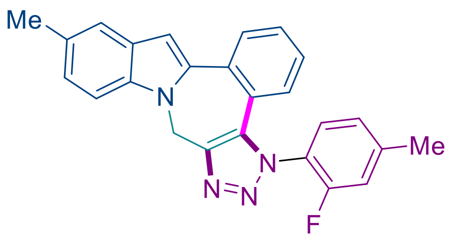

3l

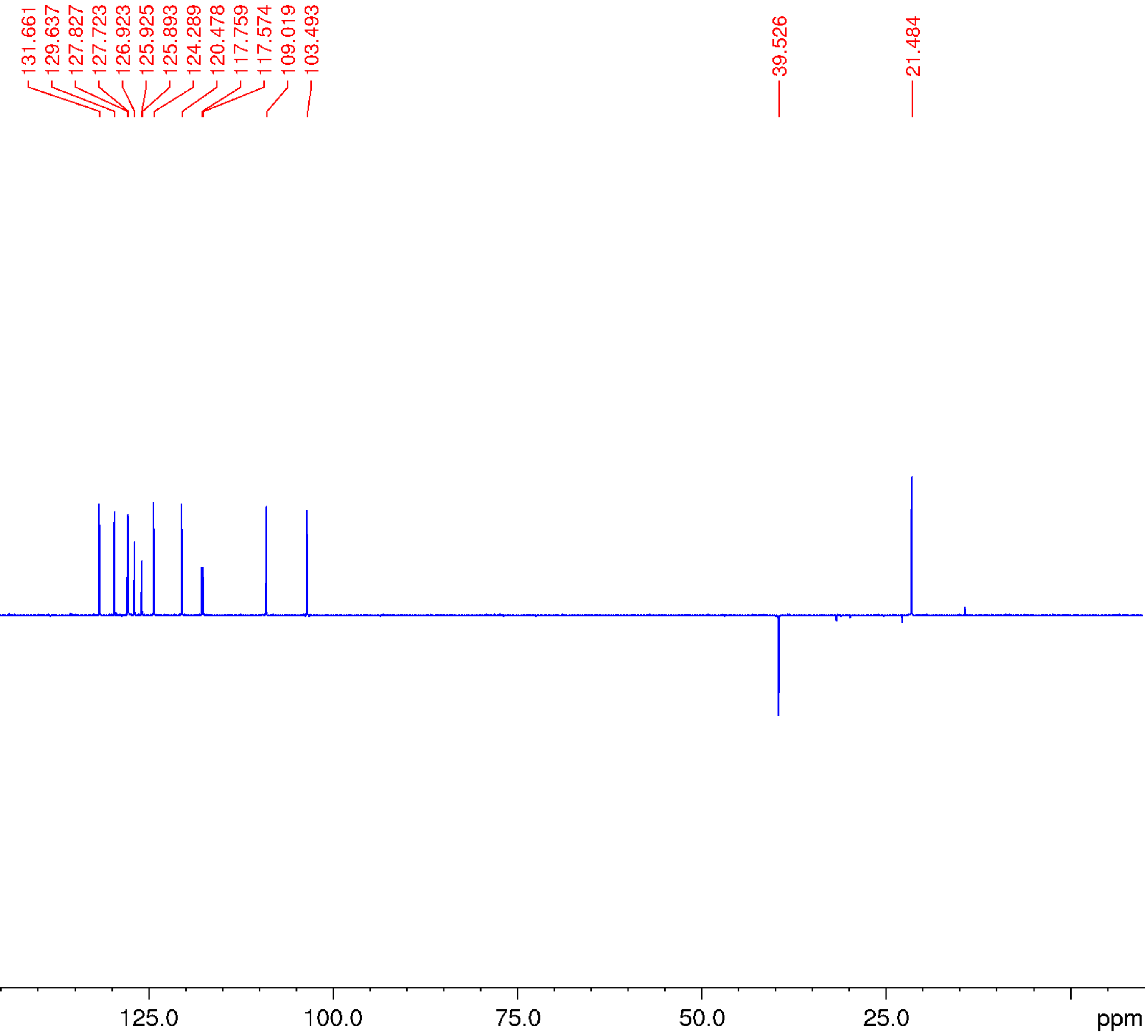

^{19}F NMR-spectrum (376.5 Hz, $CDCl_3$)

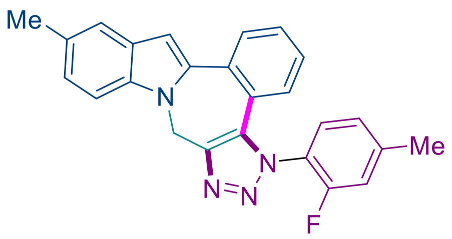

3l

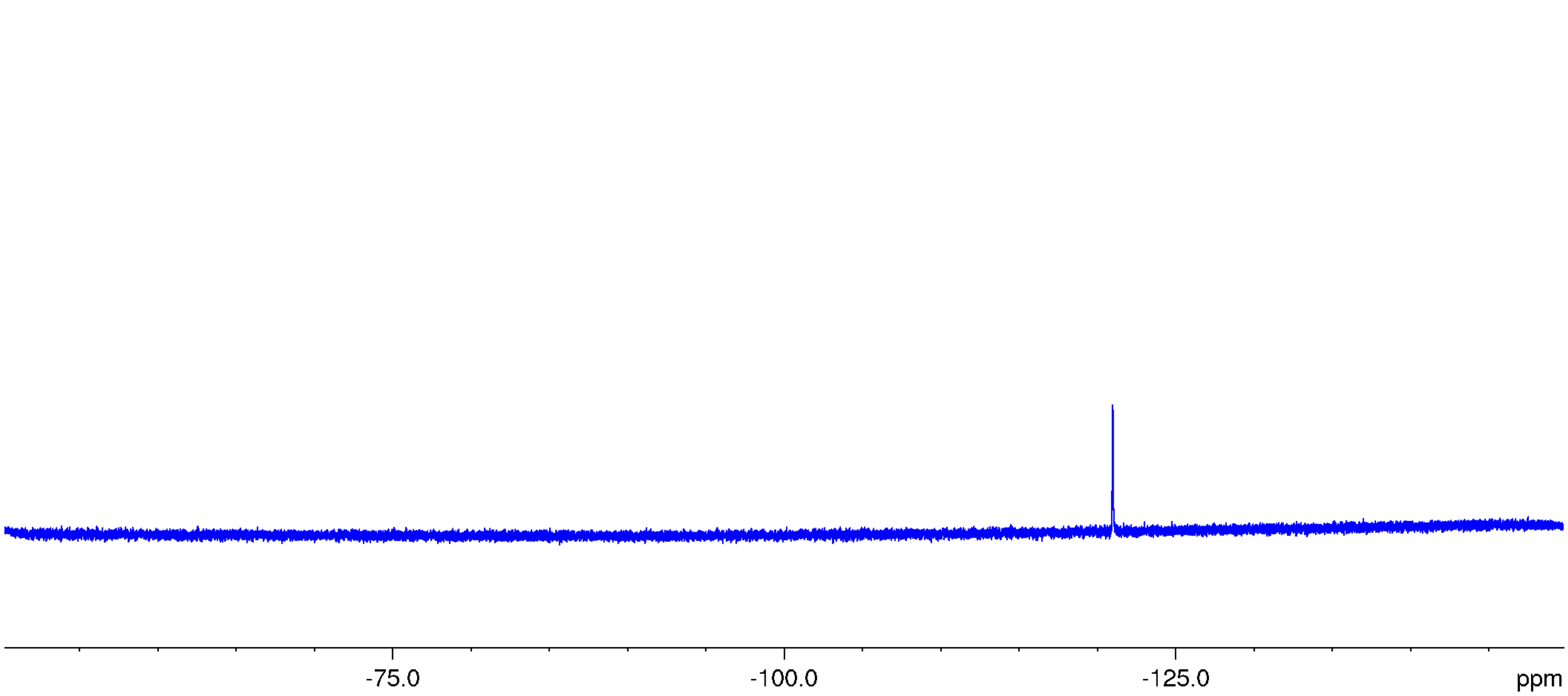

^{1}H NMR-spectrum (400 MHz, $CDCl_3$)

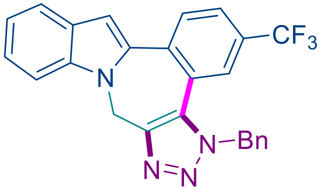

3m

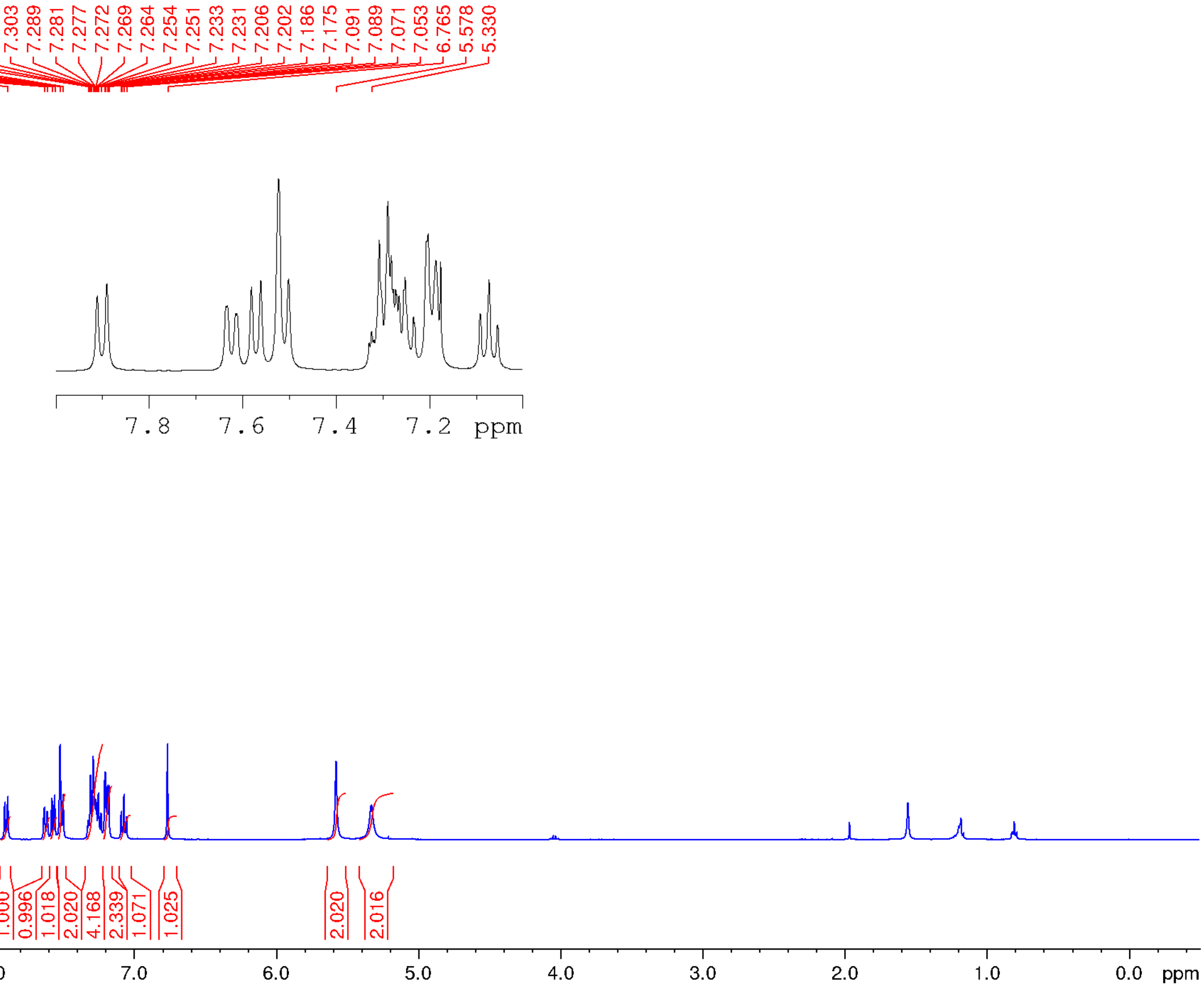

^{13}C NMR-spectrum (100 MHz, $CDCl_3$)

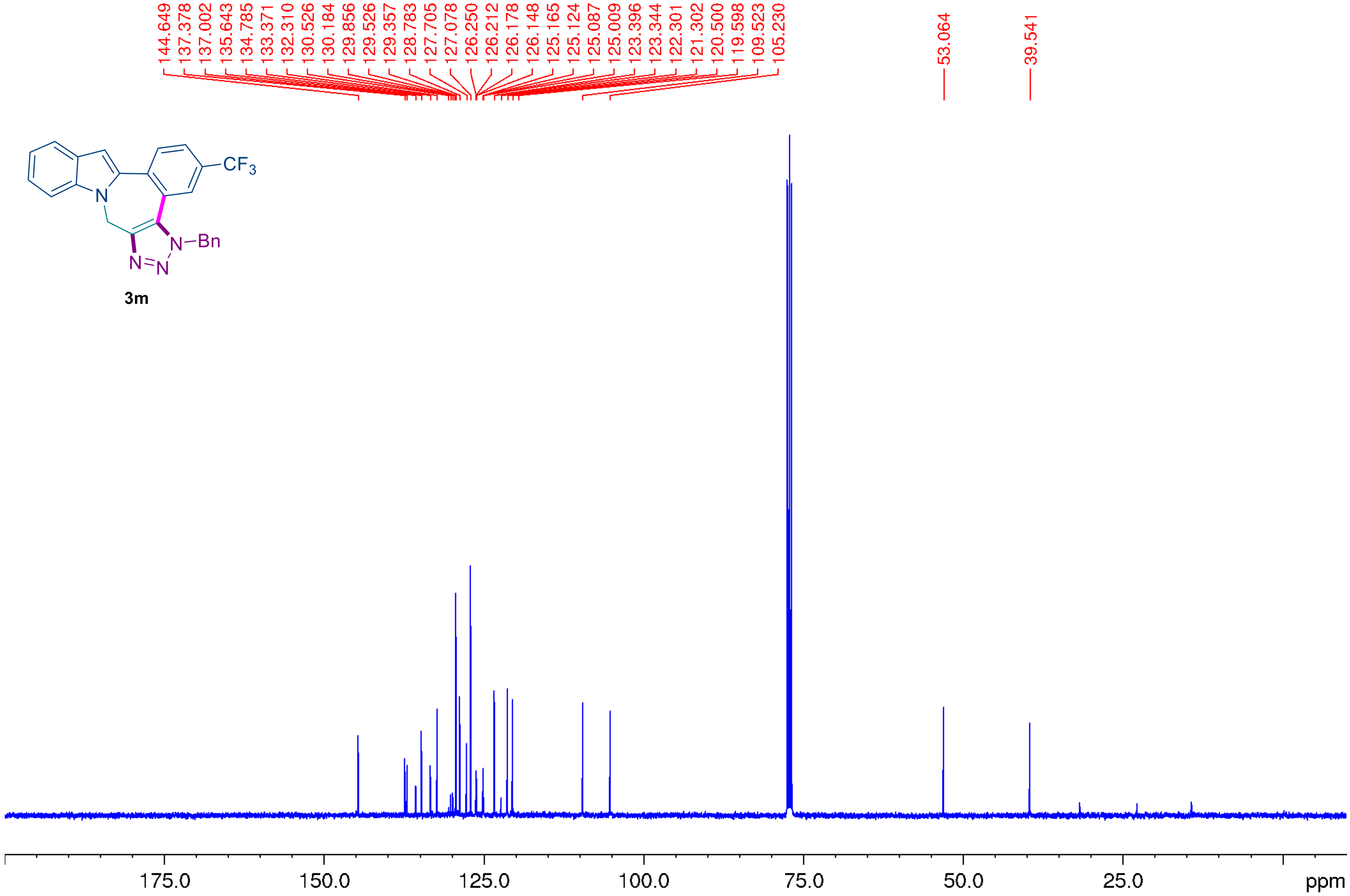

DEPT 135 NMR-spectrum ($CDCl_3$)

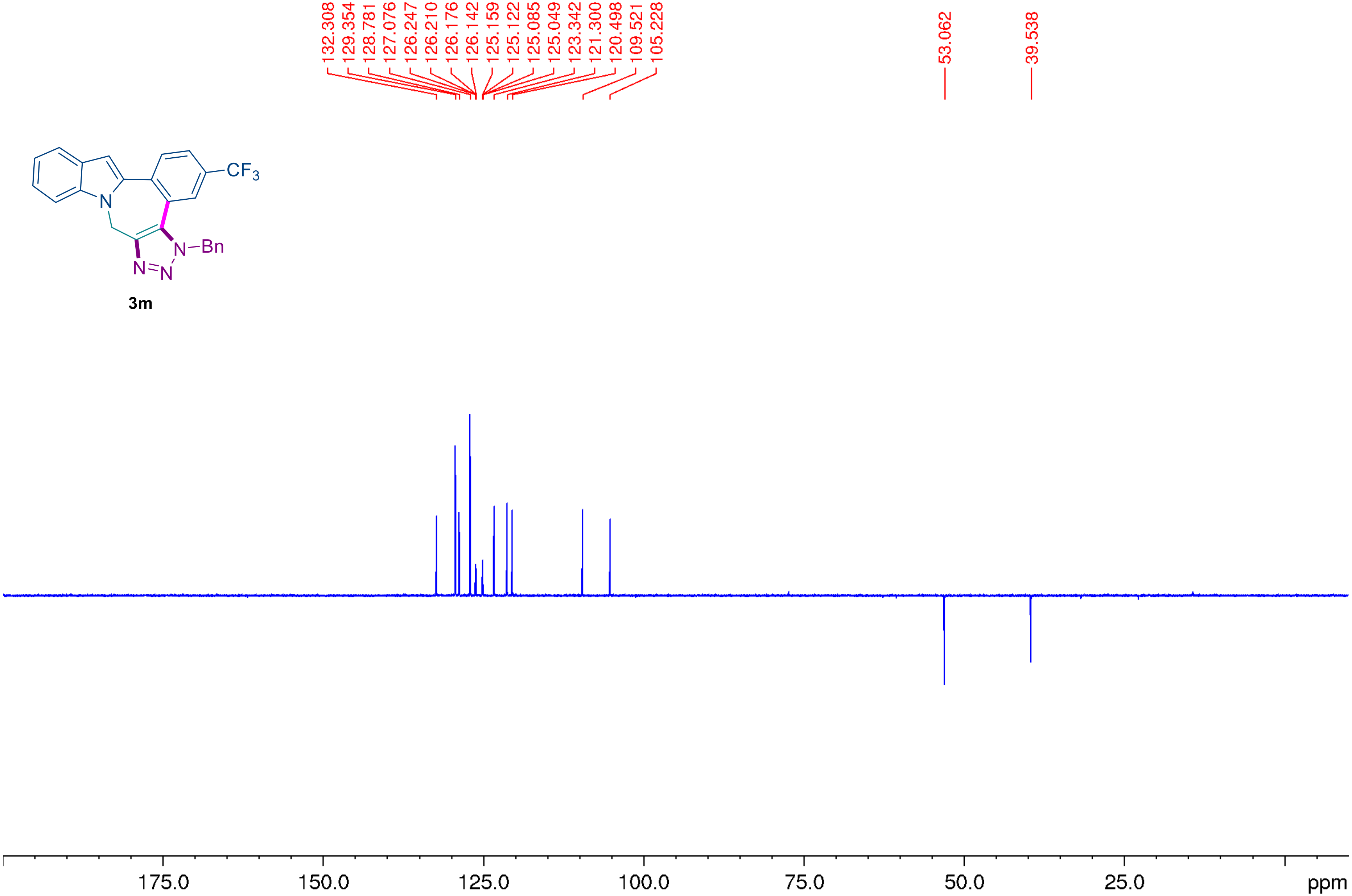

^{19}F NMR-spectrum (376.5 Hz, $CDCl_3$)

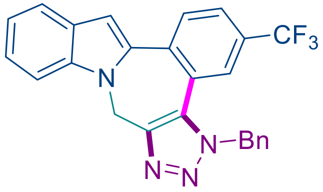

3m

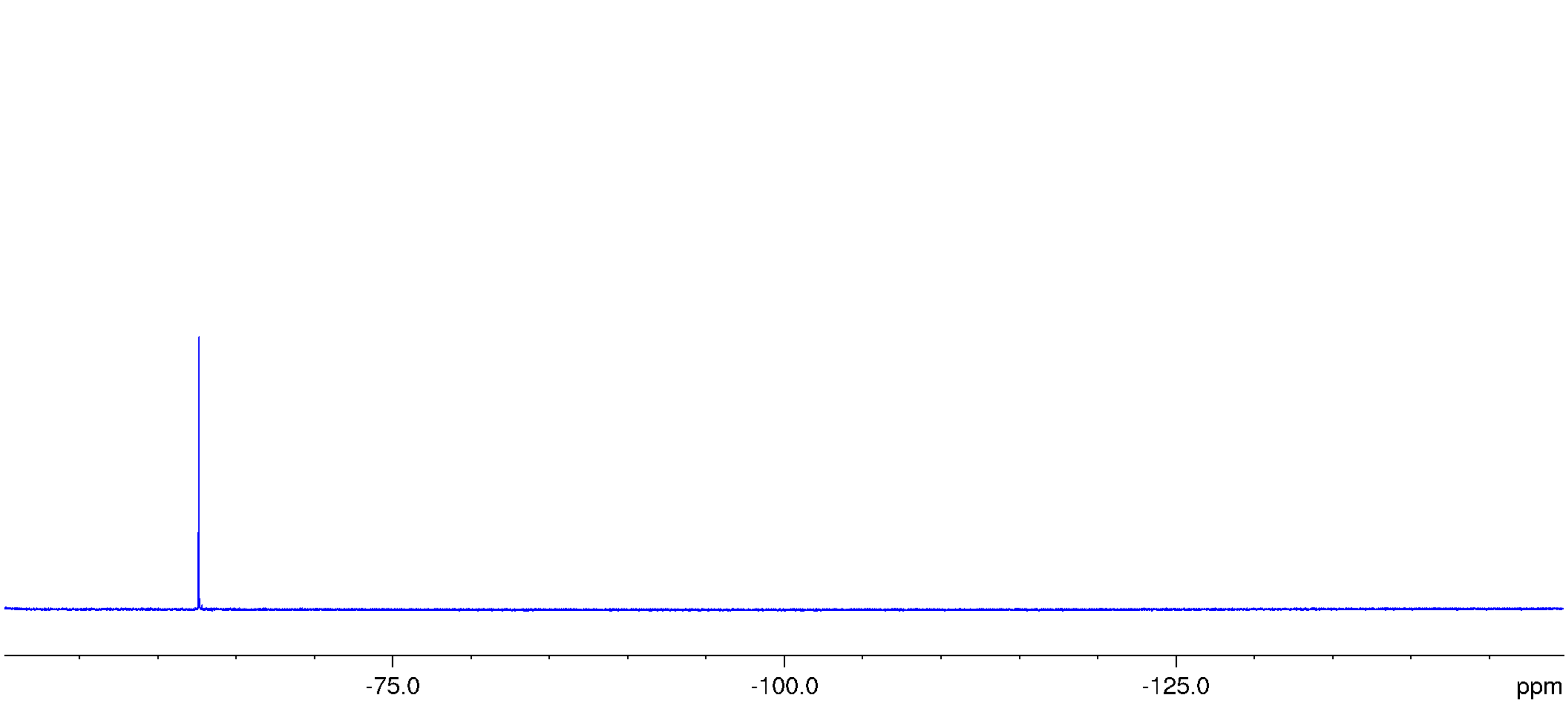

^{1}H NMR-spectrum (400 MHz, $CDCl_3$)

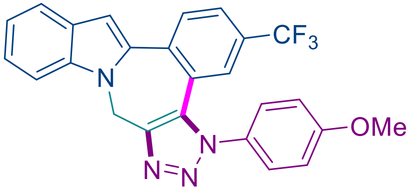

3n

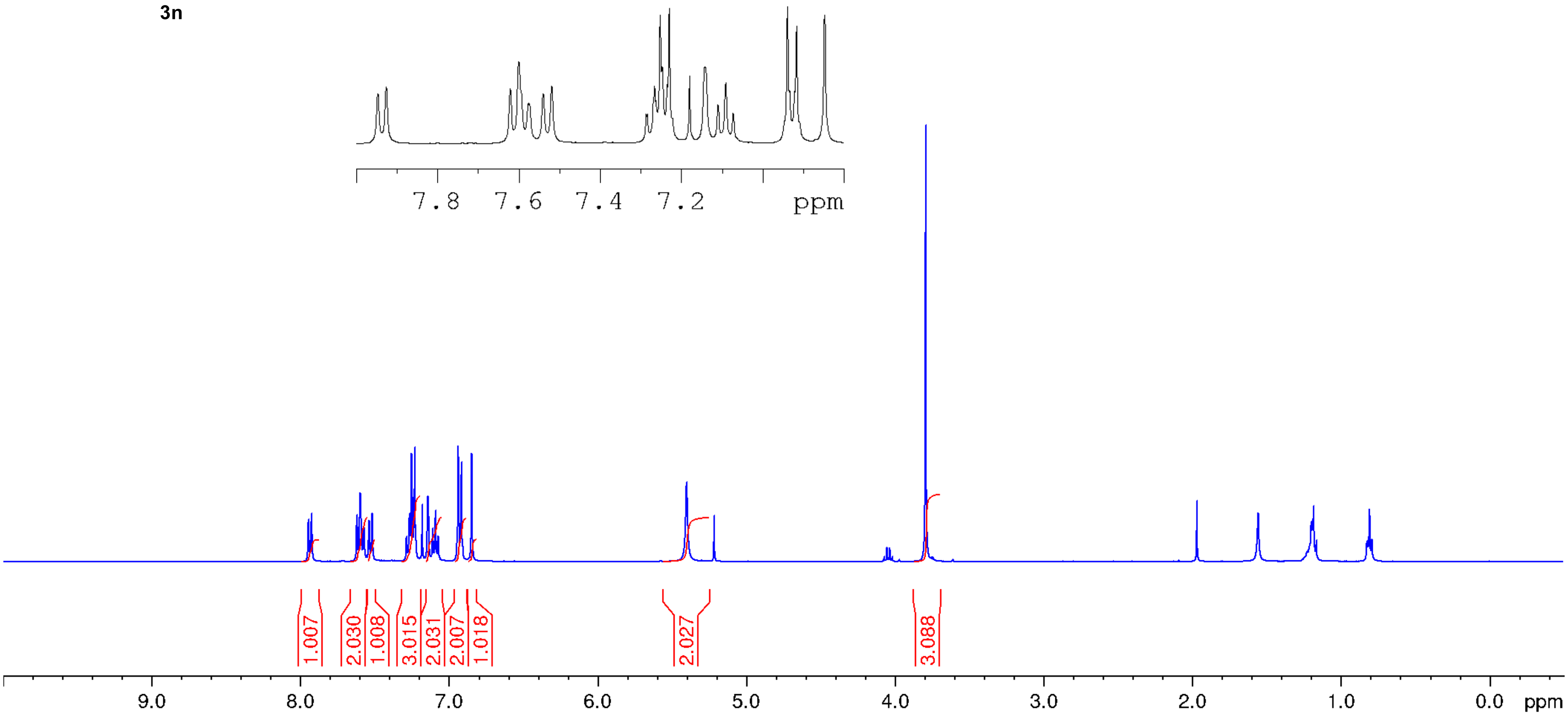

^{13}C NMR-spectrum (100 MHz, $CDCl_3$)

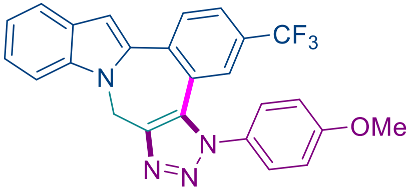

3n

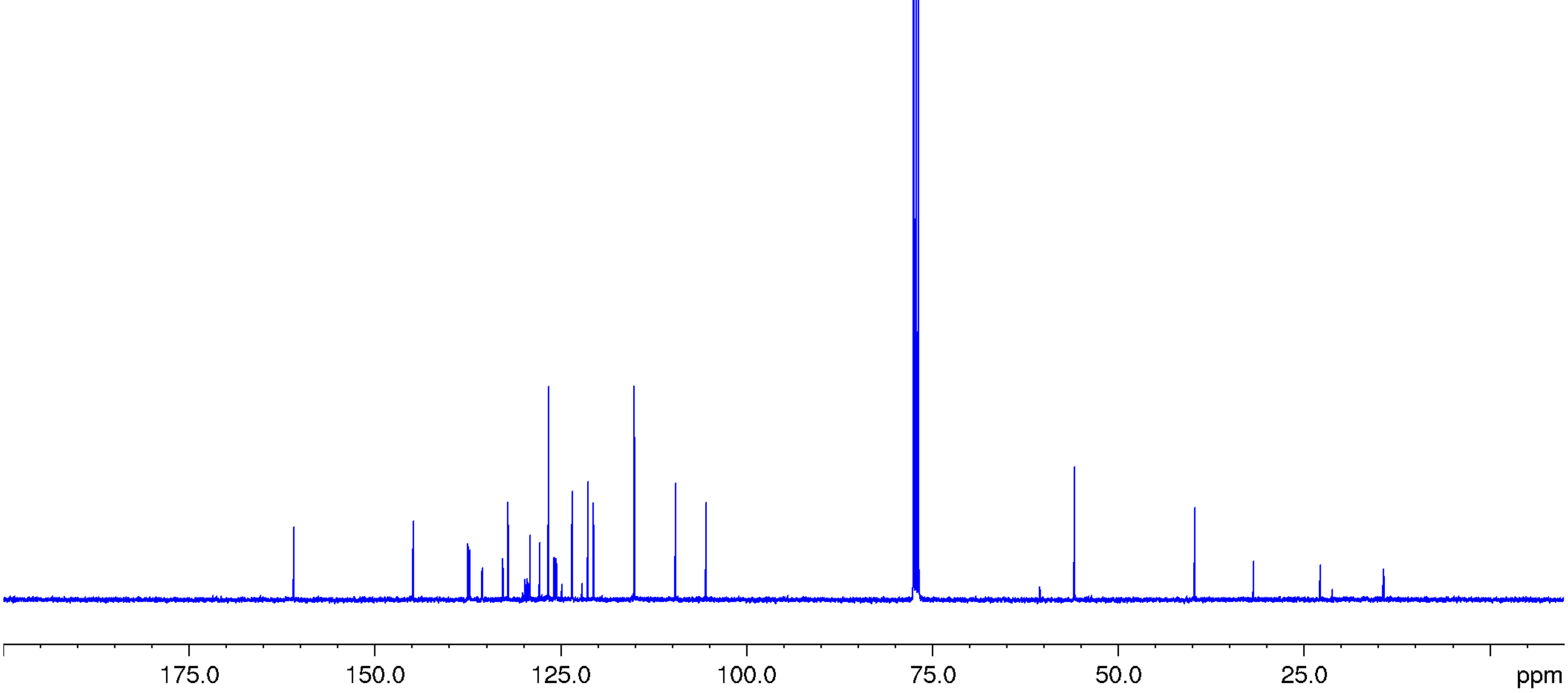

DEPT 135 NMR-spectrum ($CDCl_3$)

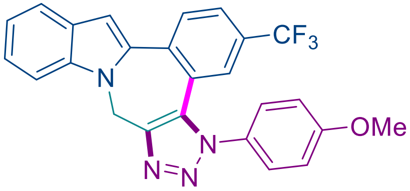

3n

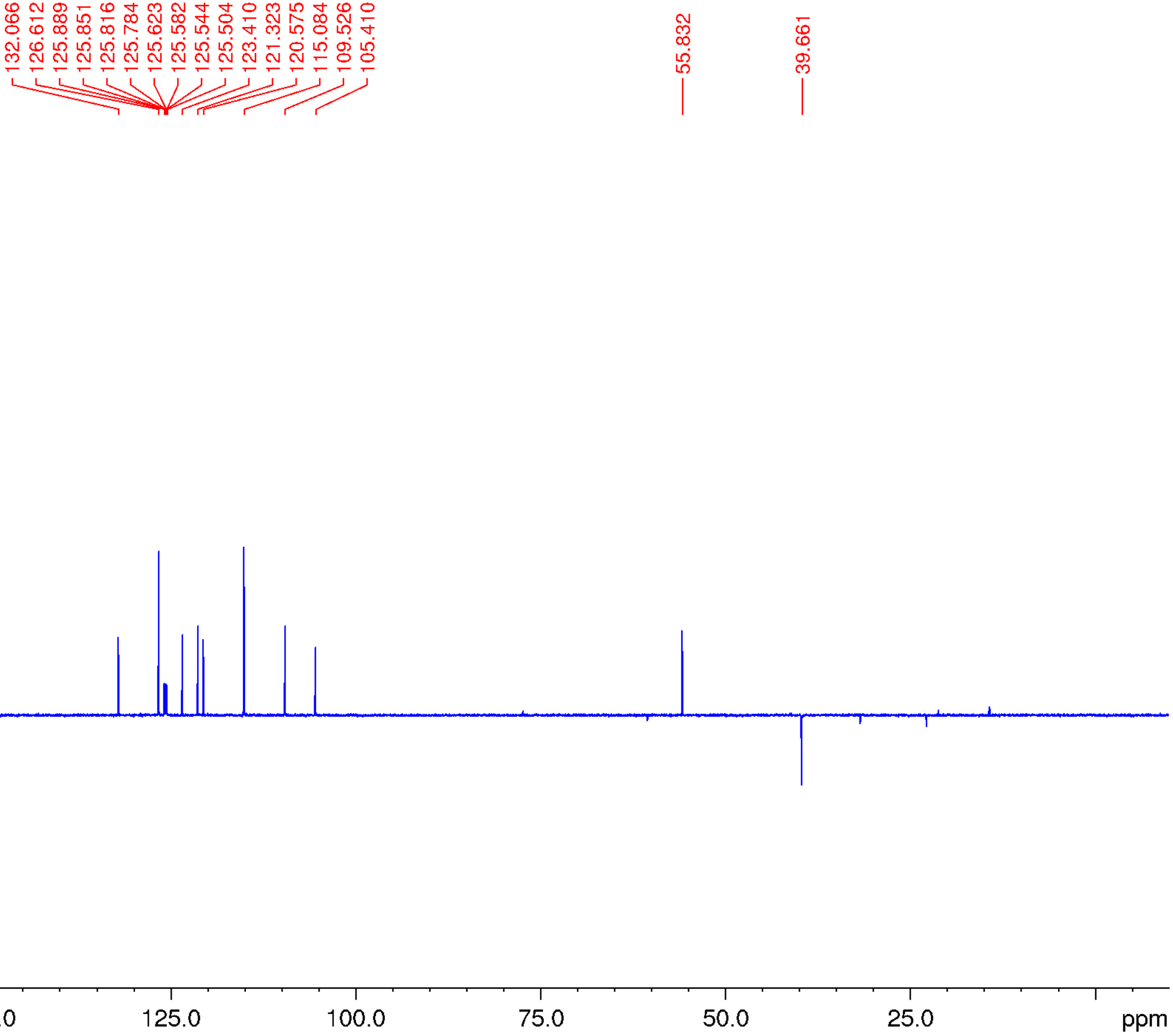

^{19}F NMR-spectrum (376.5 Hz, $CDCl_3$)

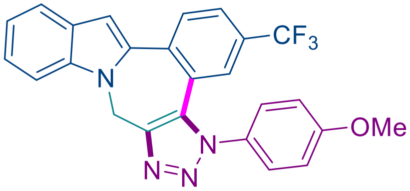

3n

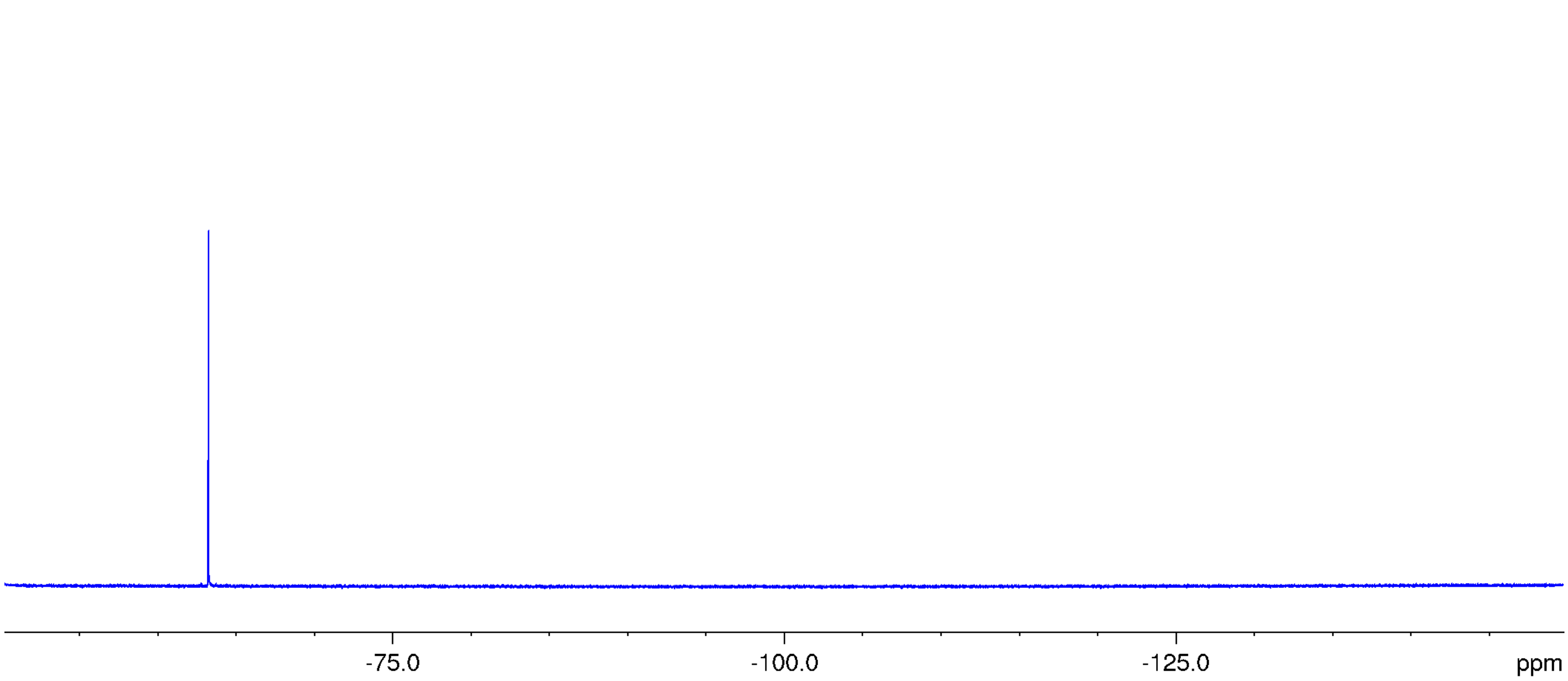

^1H NMR-spectrum (400 MHz, $CDCl_3$)

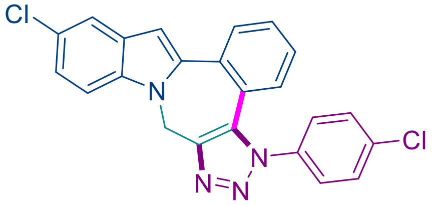

3o

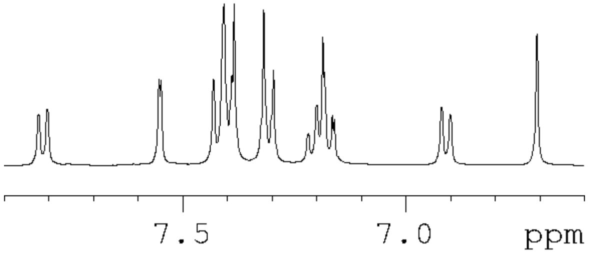

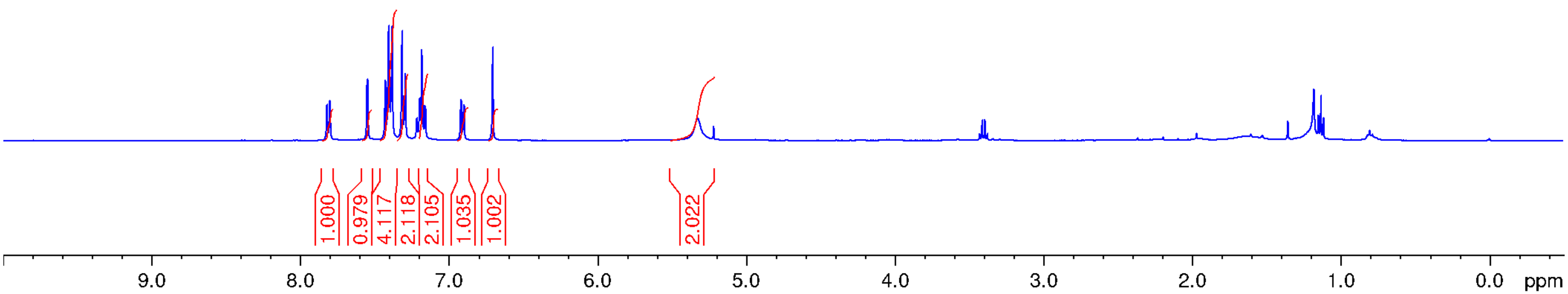

^{13}C NMR-spectrum (100 MHz, $CDCl_3$)

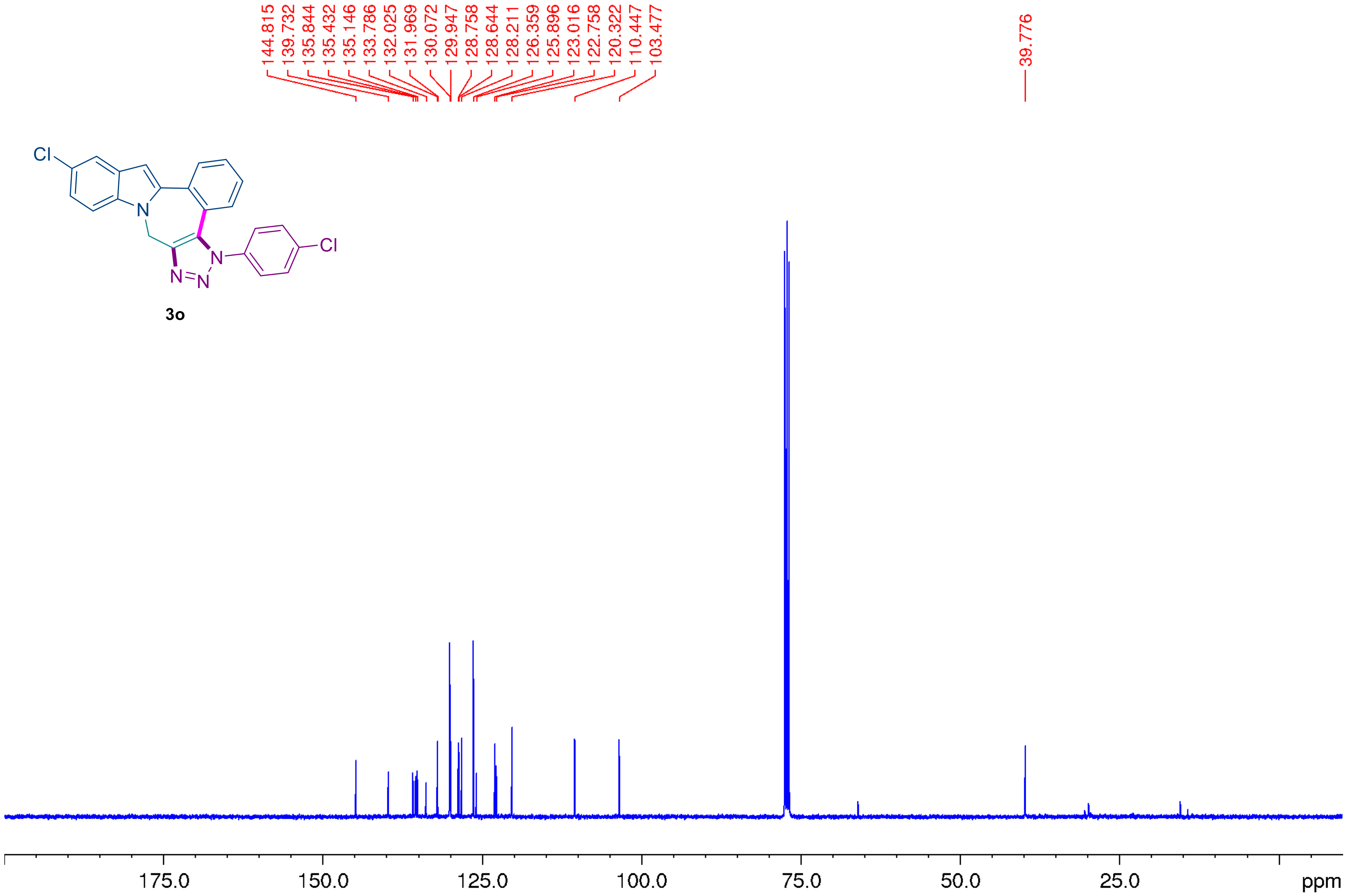

DEPT 135 NMR-spectrum ($CDCl_3$)

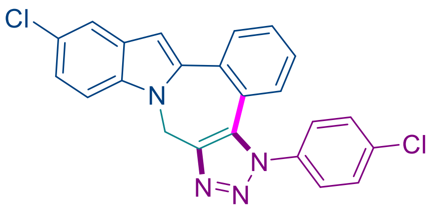

3o

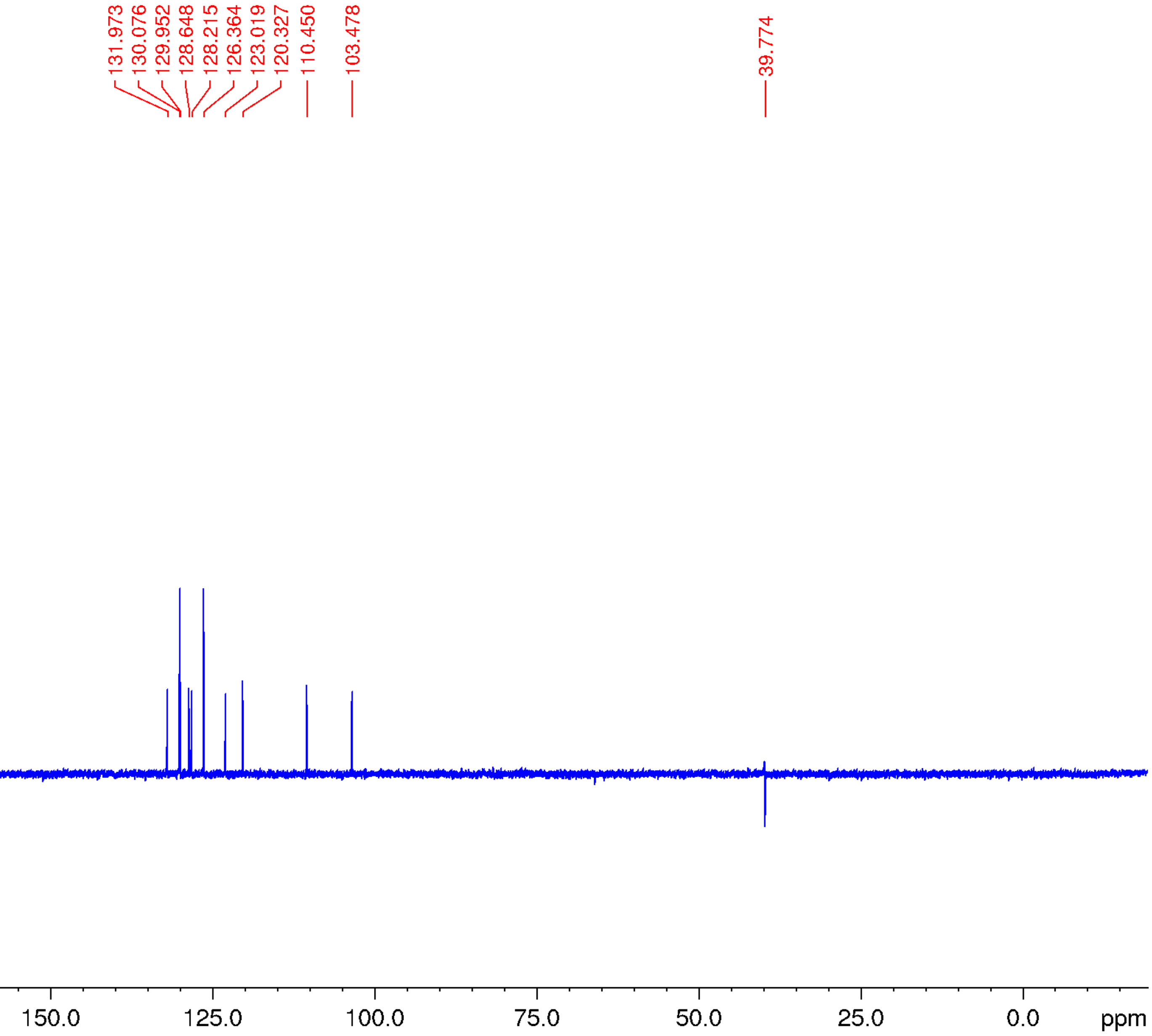

^{1}H NMR-spectrum (400 MHz, $CDCl_3$)

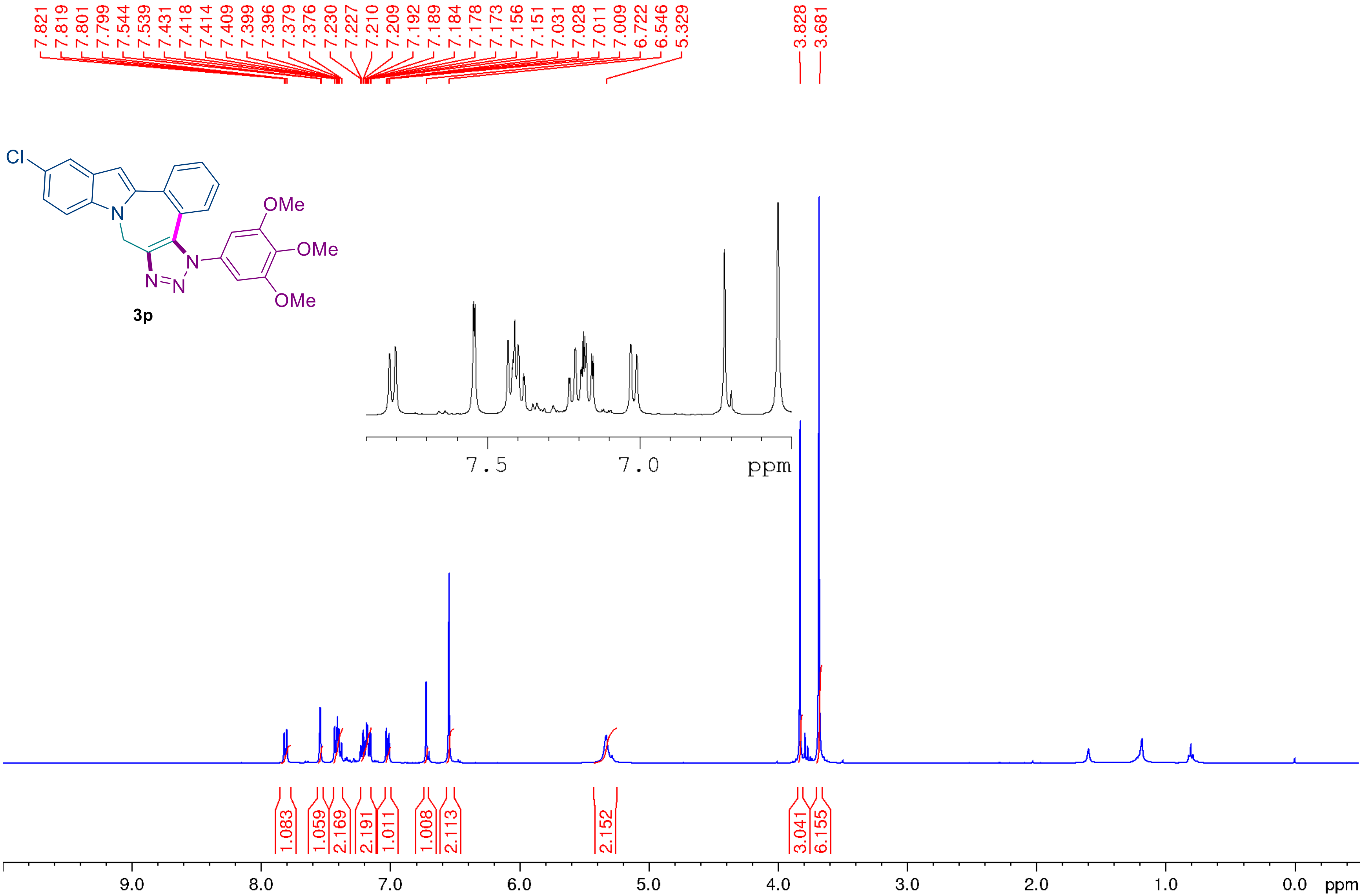

^{13}C NMR-spectrum (100 MHz, $CDCl_3$)

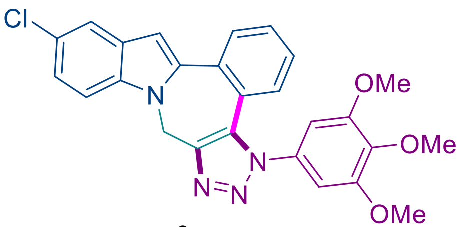

3p

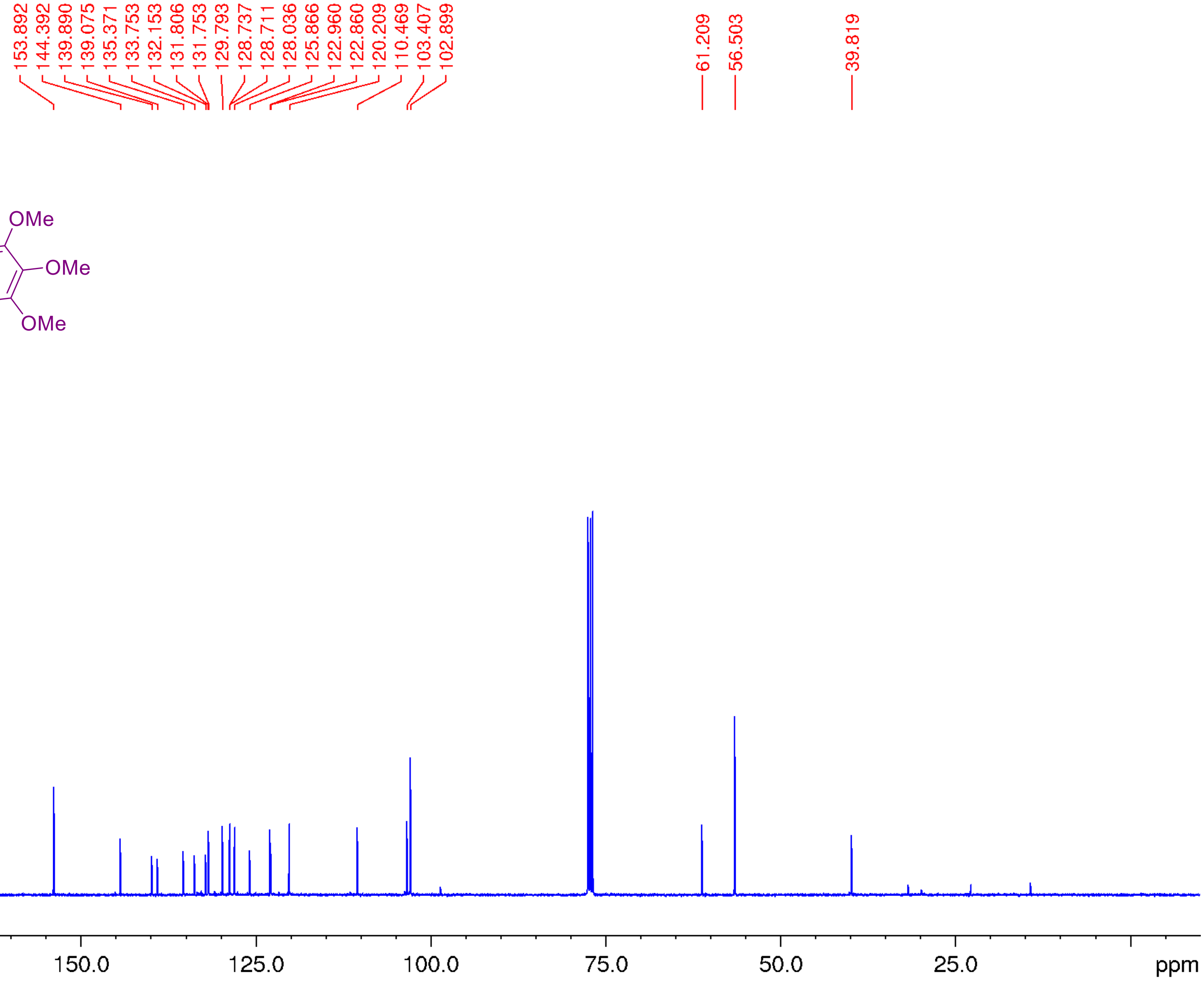

DEPT 135 NMR-spectrum ($CDCl_3$)

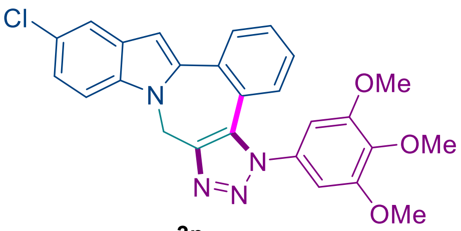

3p

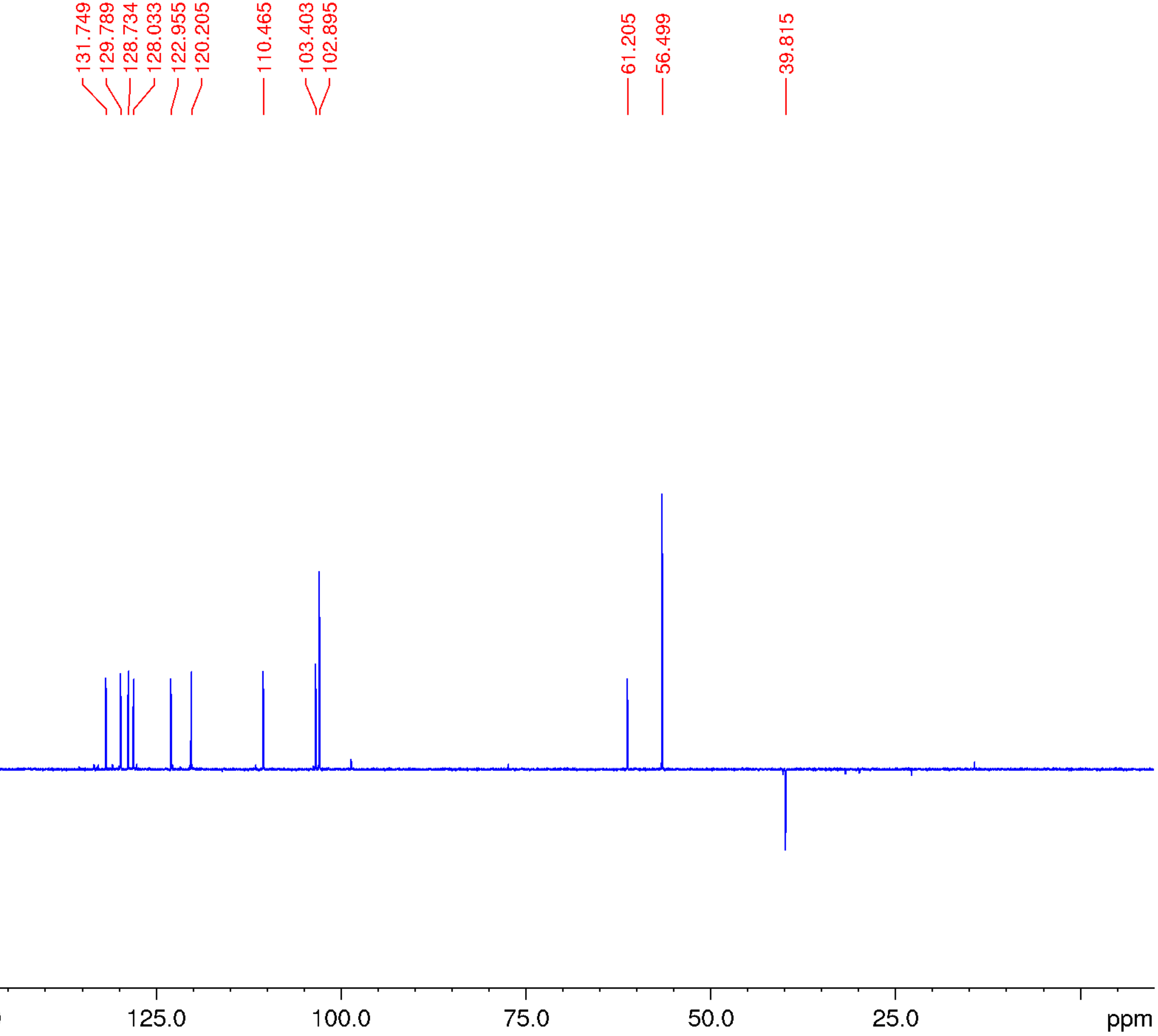

^{1}H NMR-spectrum (400 MHz, $CDCl_3$)

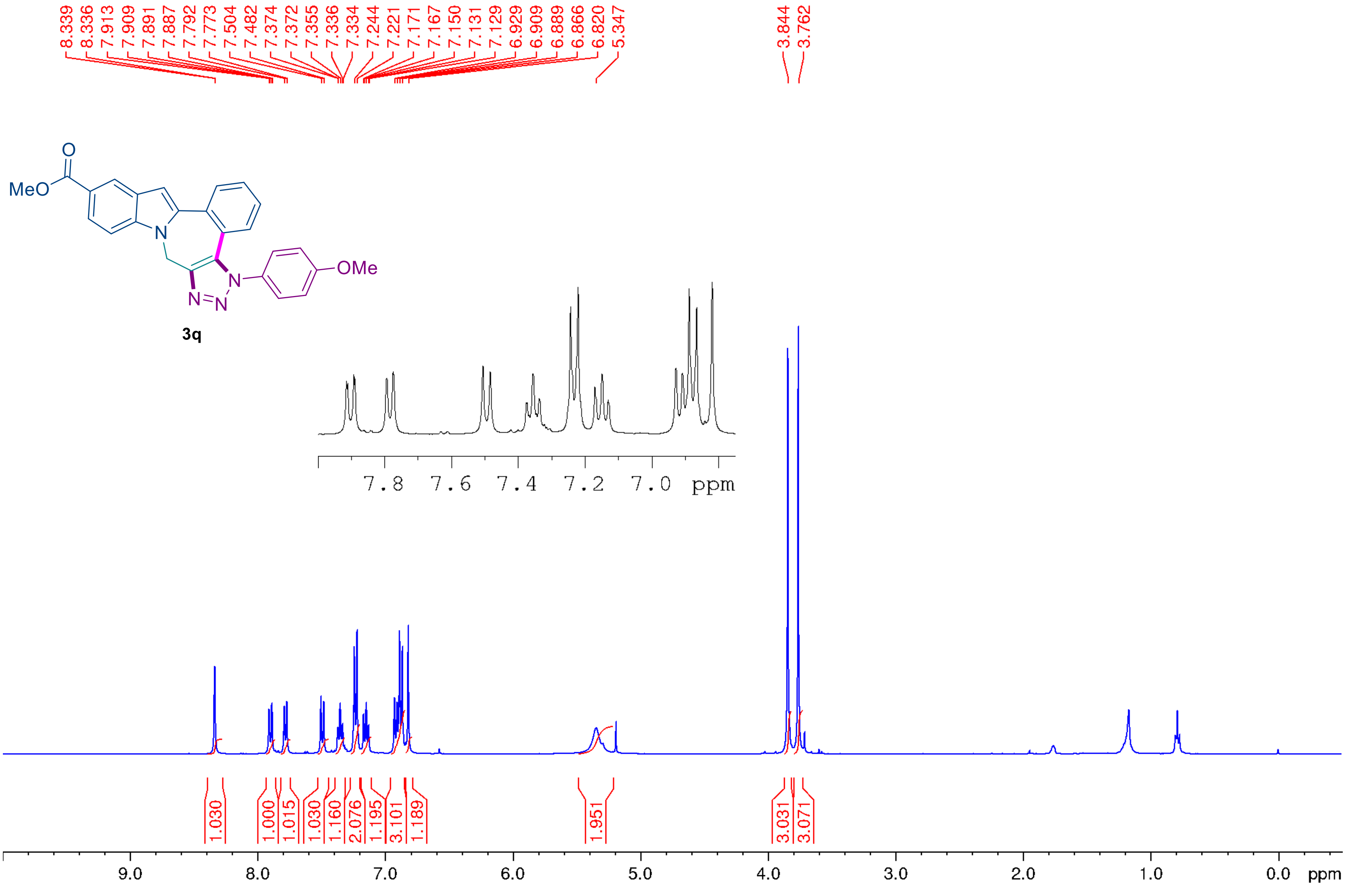

^{13}C NMR-spectrum (100 MHz, $CDCl_3$)

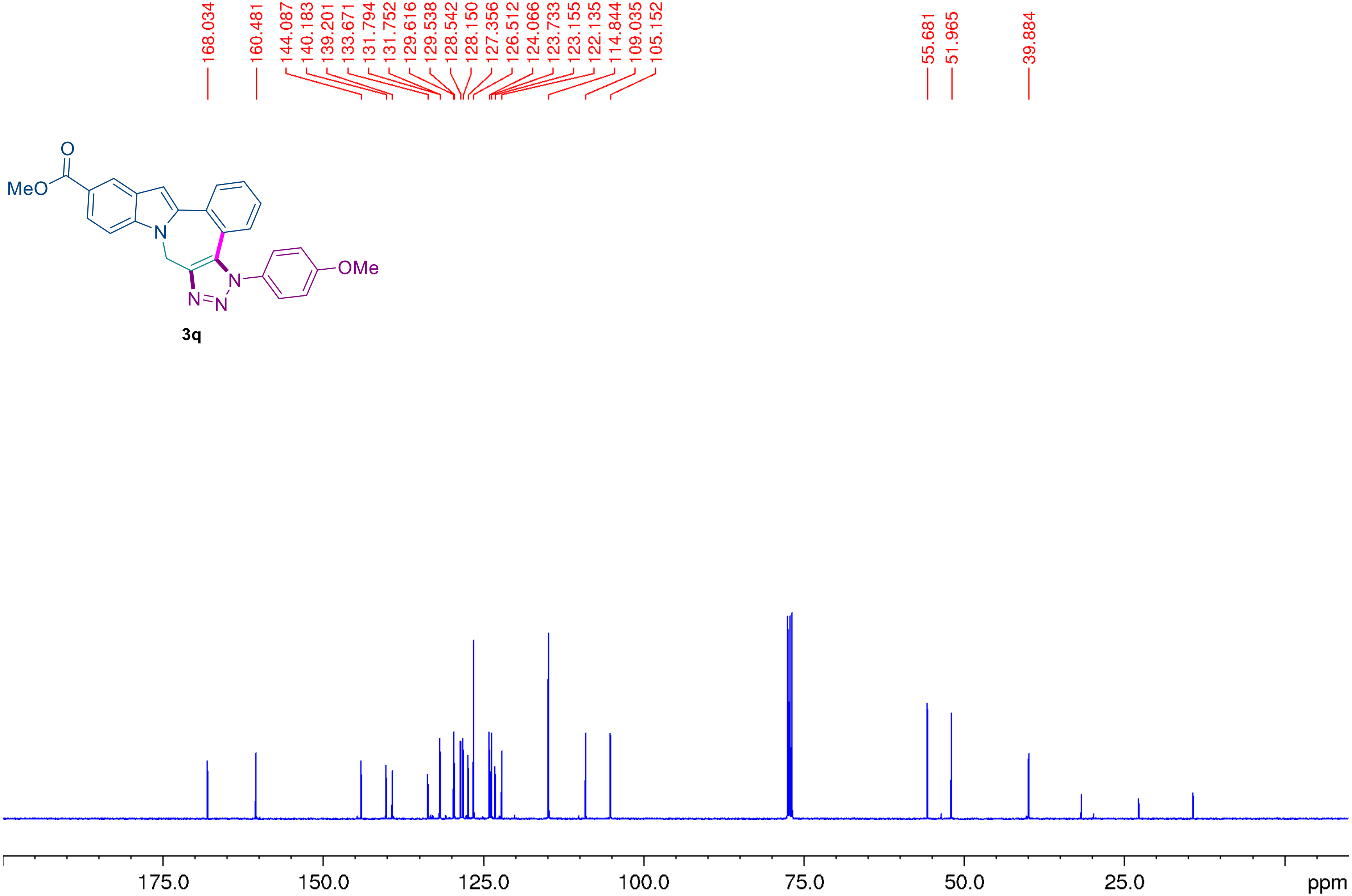

DEPT 135 NMR-spectrum ($CDCl_3$)

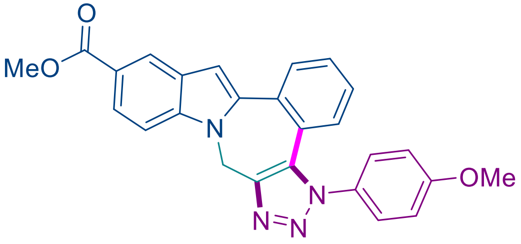

3q

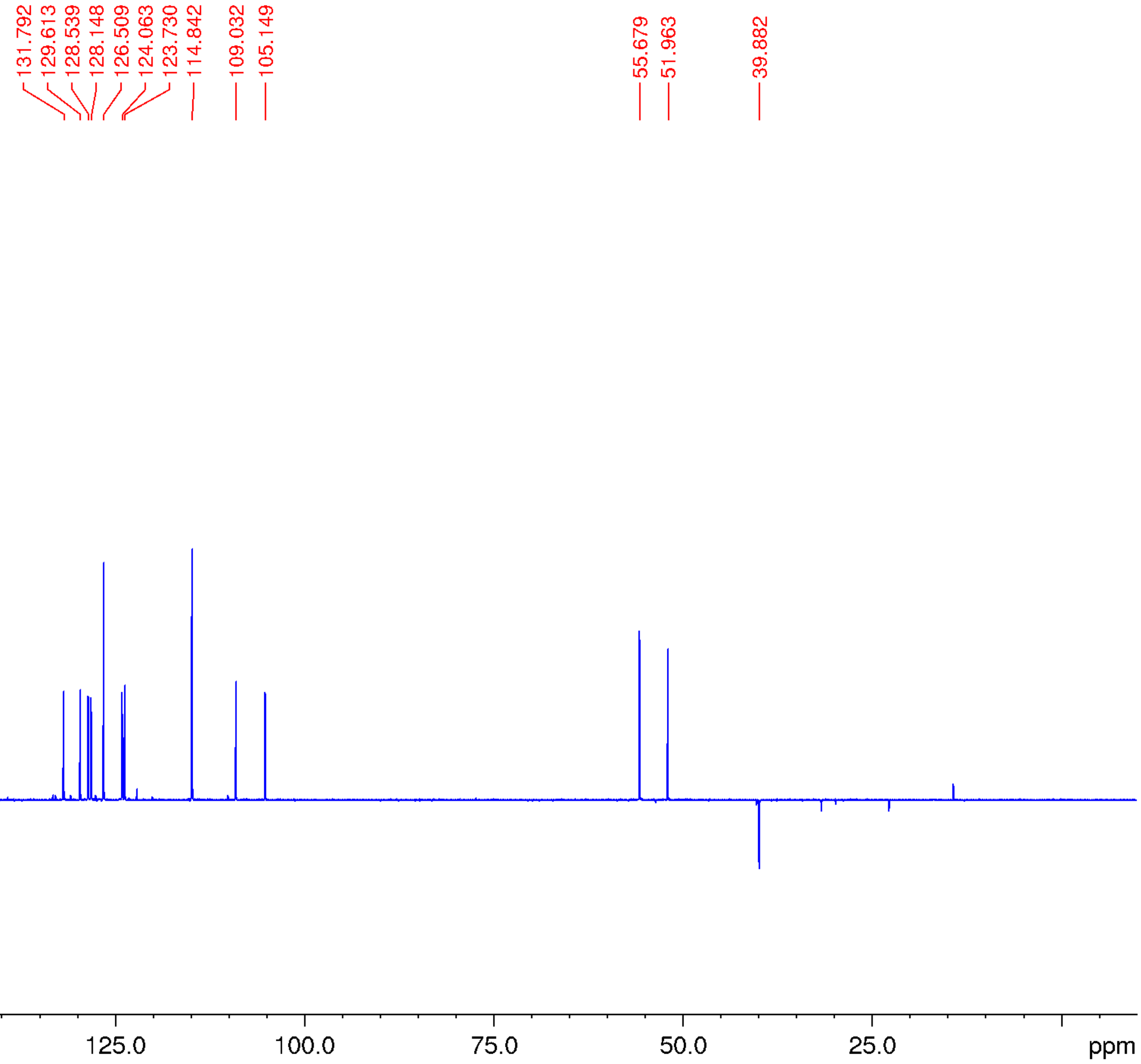

^{1}H NMR-spectrum (400 MHz, $CDCl_3$)

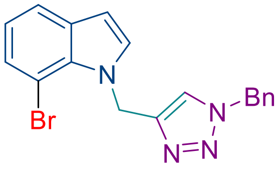

4a

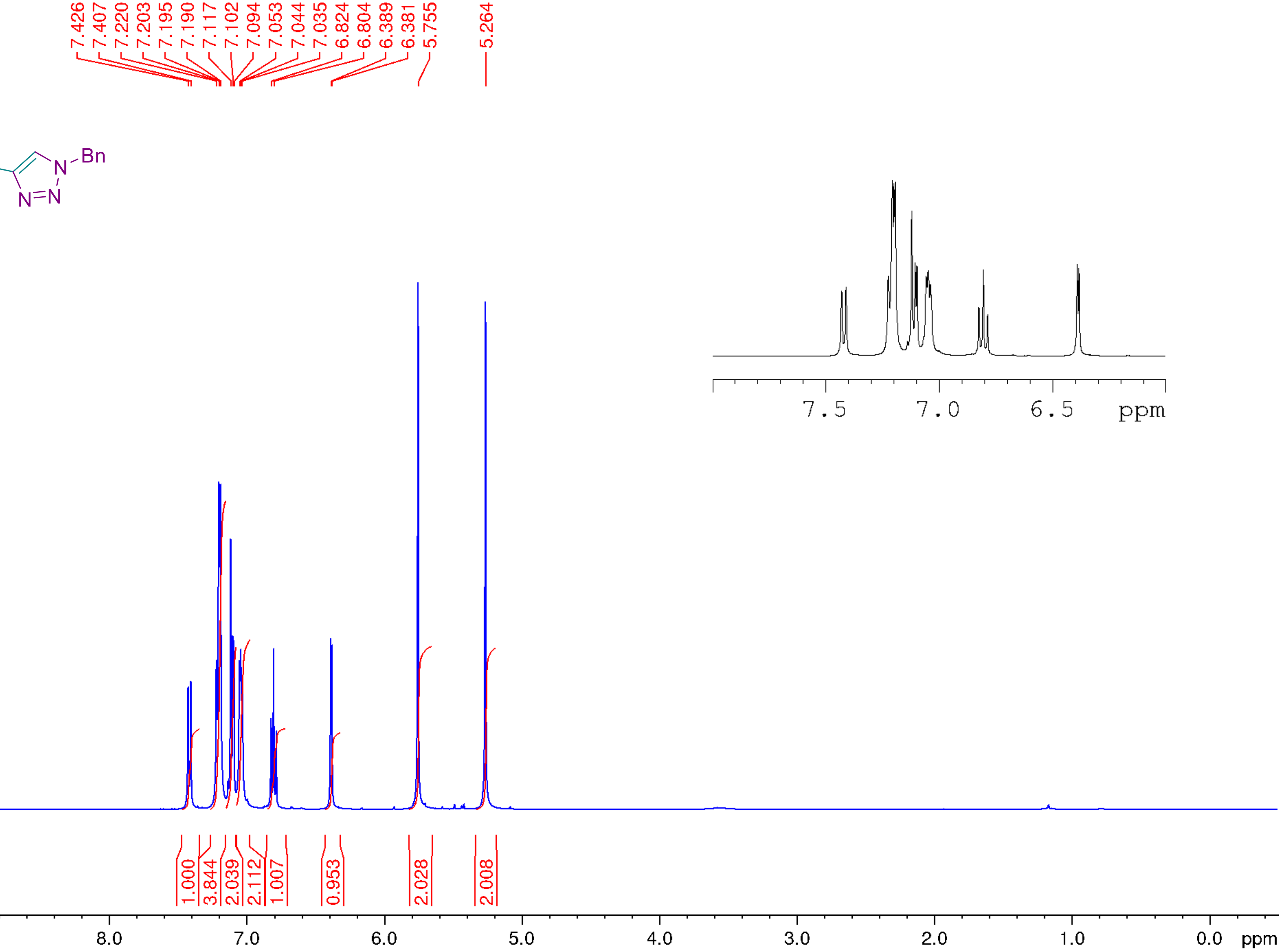

^{13}C NMR-spectrum (100 MHz, $CDCl_3$)

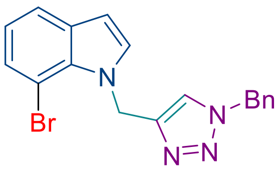

4a

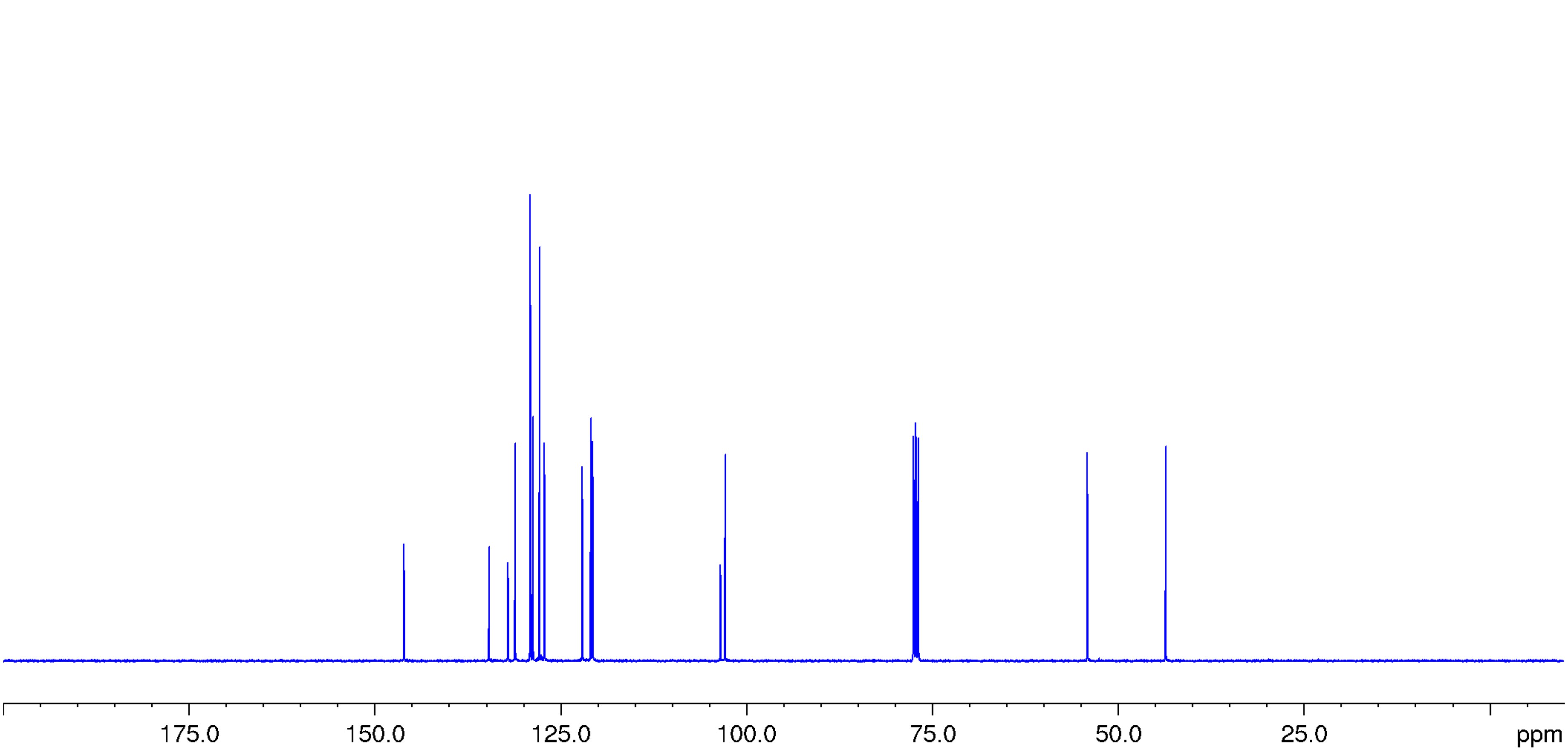

DEPT 135 NMR-spectrum ($CDCl_3$)

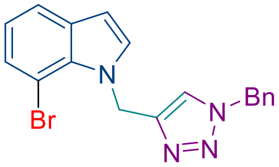

4a

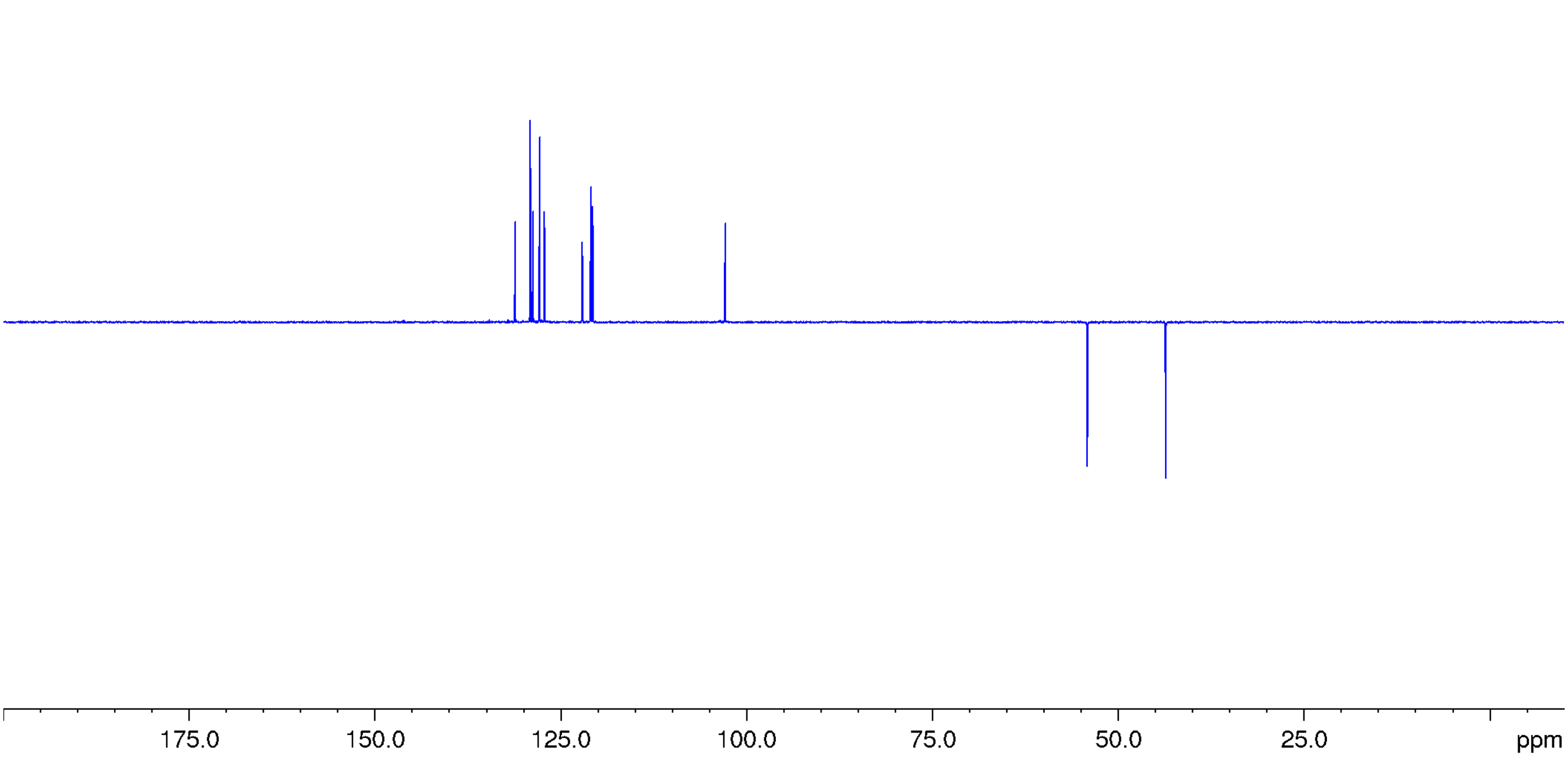

^{1}H NMR-spectrum (400 MHz, $CDCl_3$)

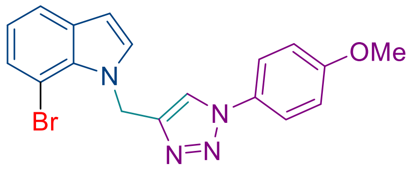

4b

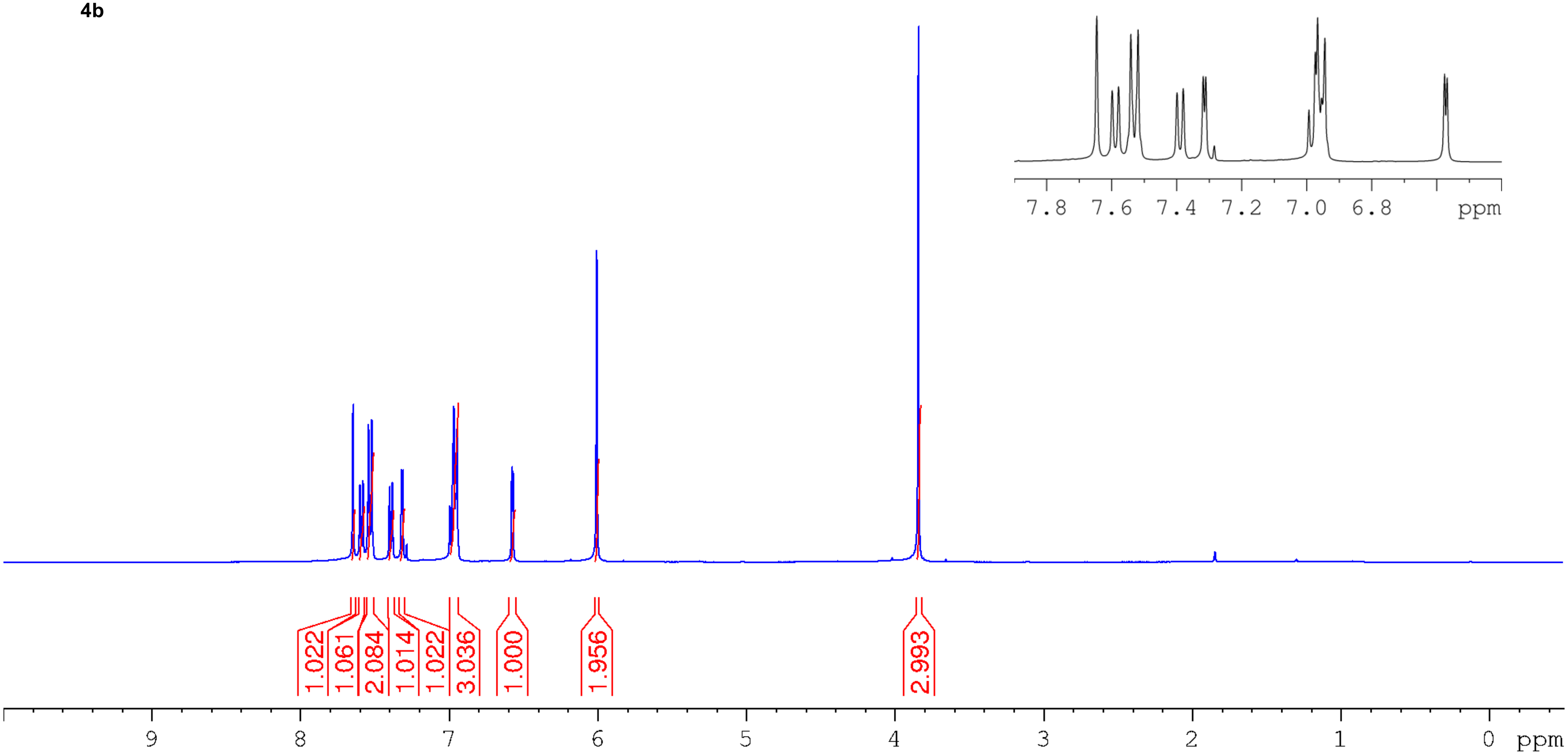

^{13}C NMR-spectrum (100 MHz, $CDCl_3$)

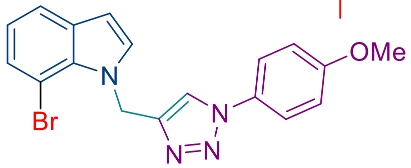

4b

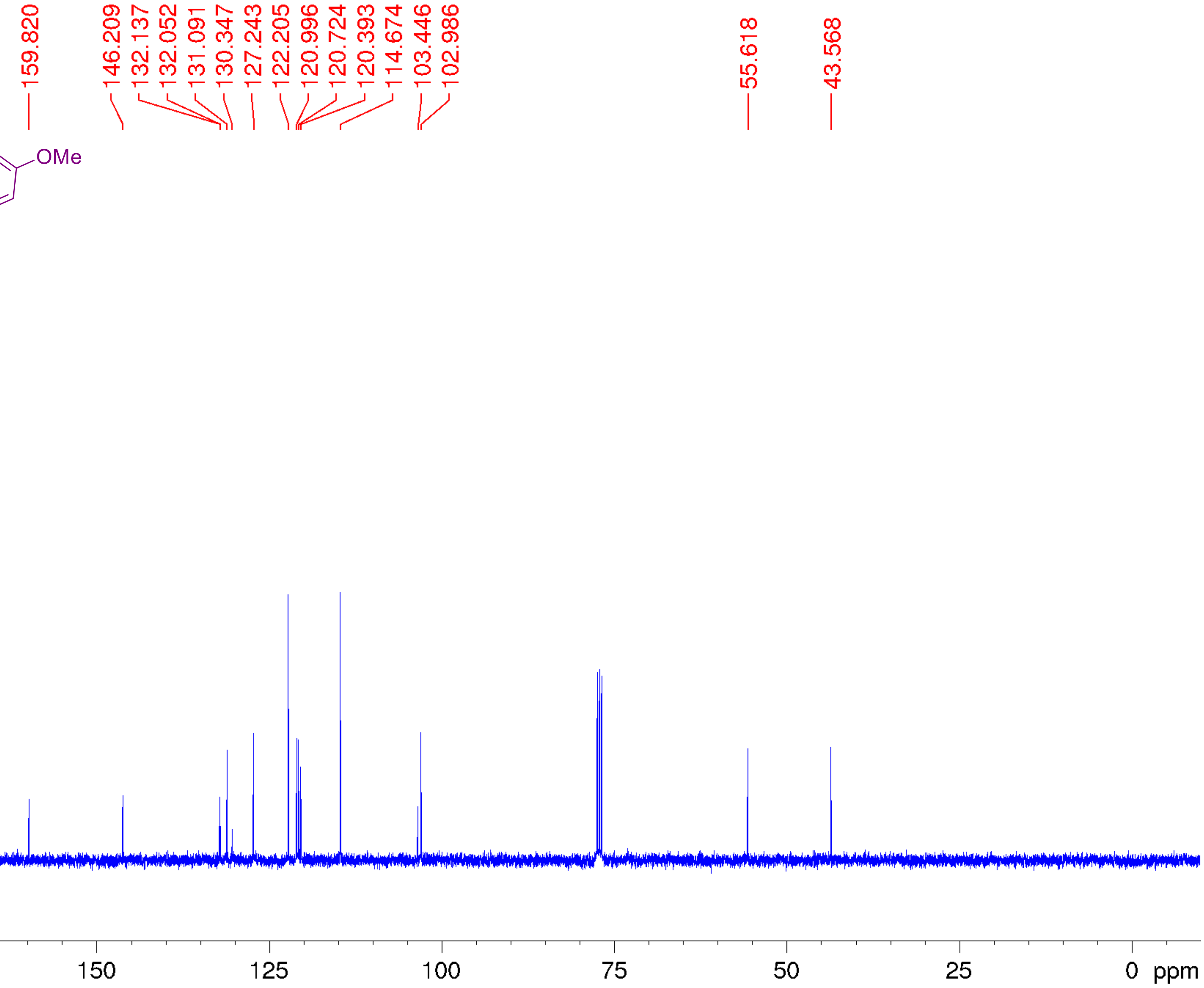

DEPT 135 NMR-spectrum ($CDCl_3$)

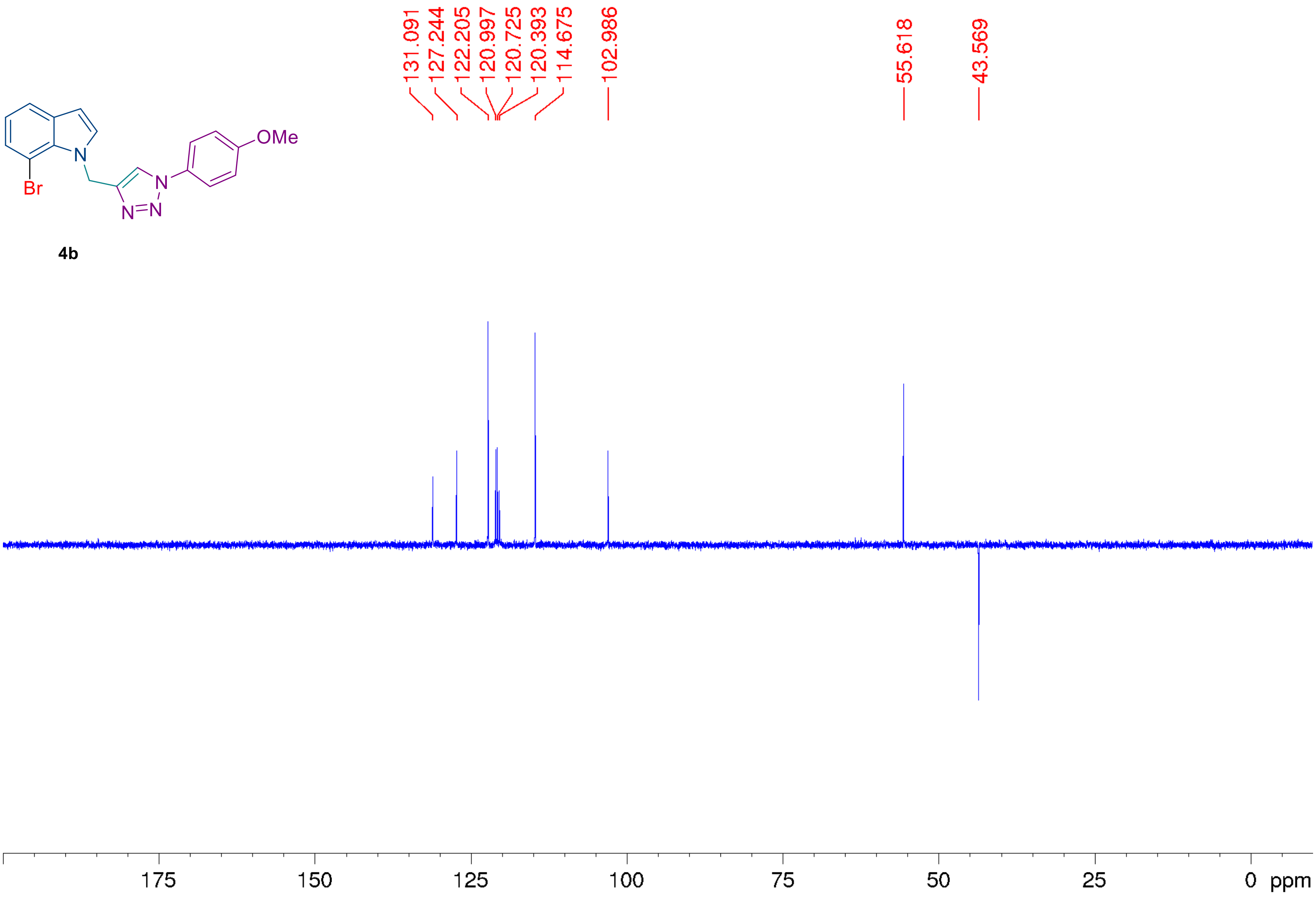

^{1}H NMR-spectrum (400 MHz, $CDCl_3$)

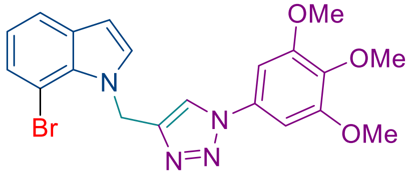

4c

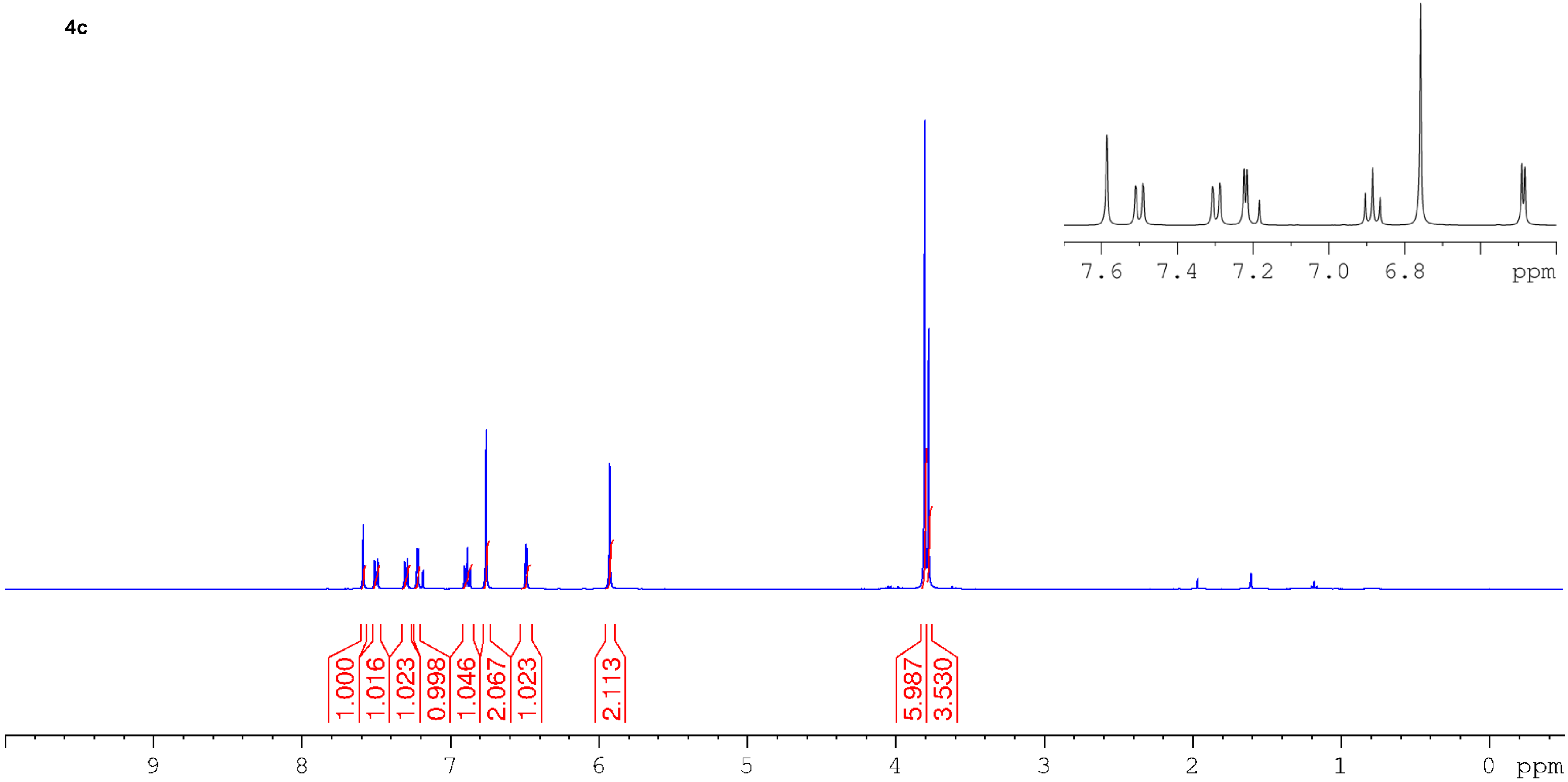

^{13}C NMR-spectrum (100 MHz, $CDCl_3$)

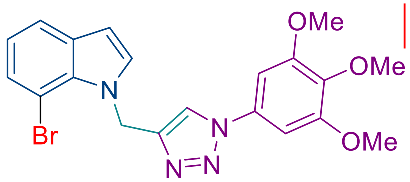

4c

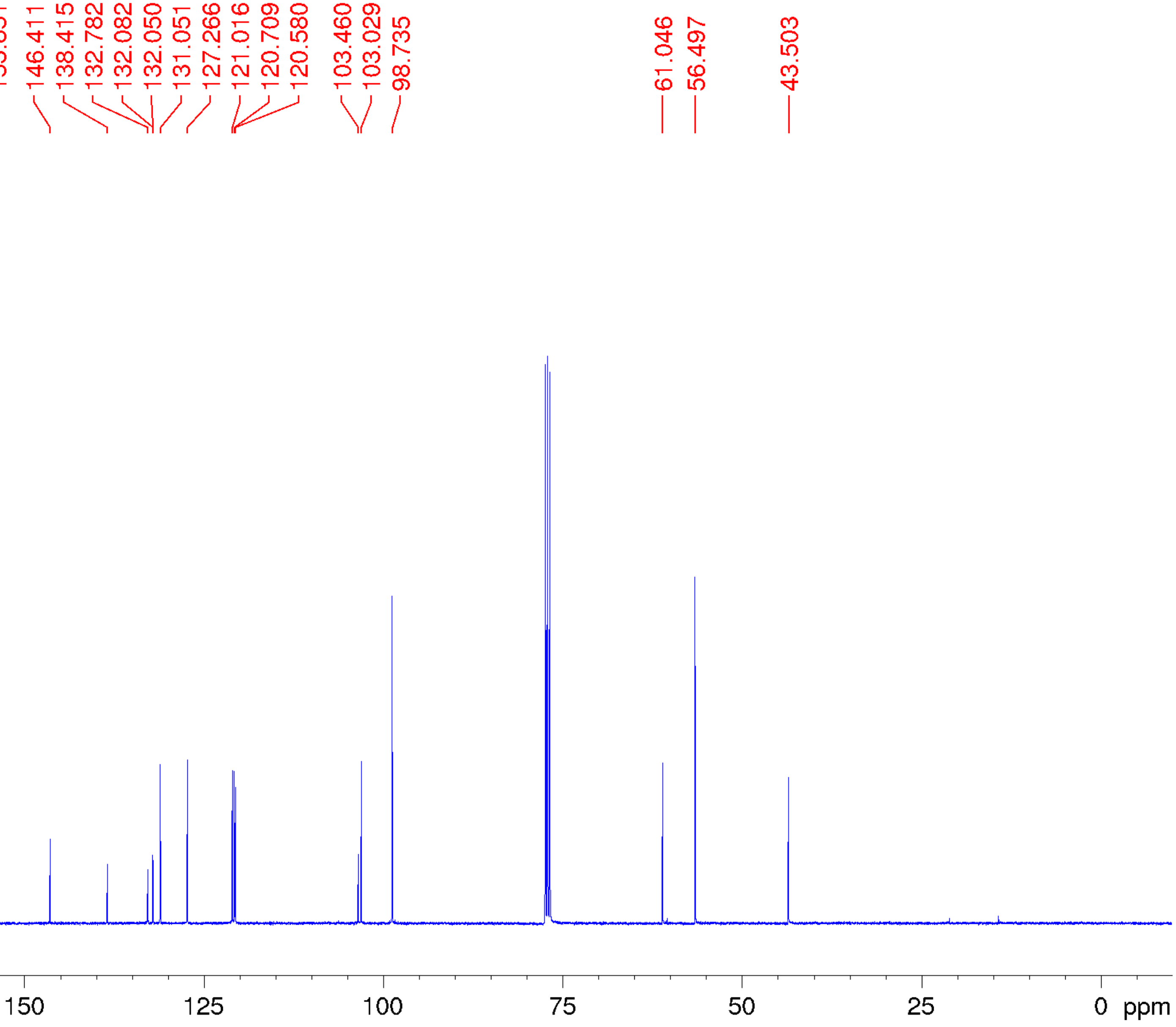

DEPT 135 NMR-spectrum ($CDCl_3$)

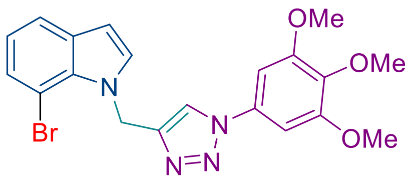

4c

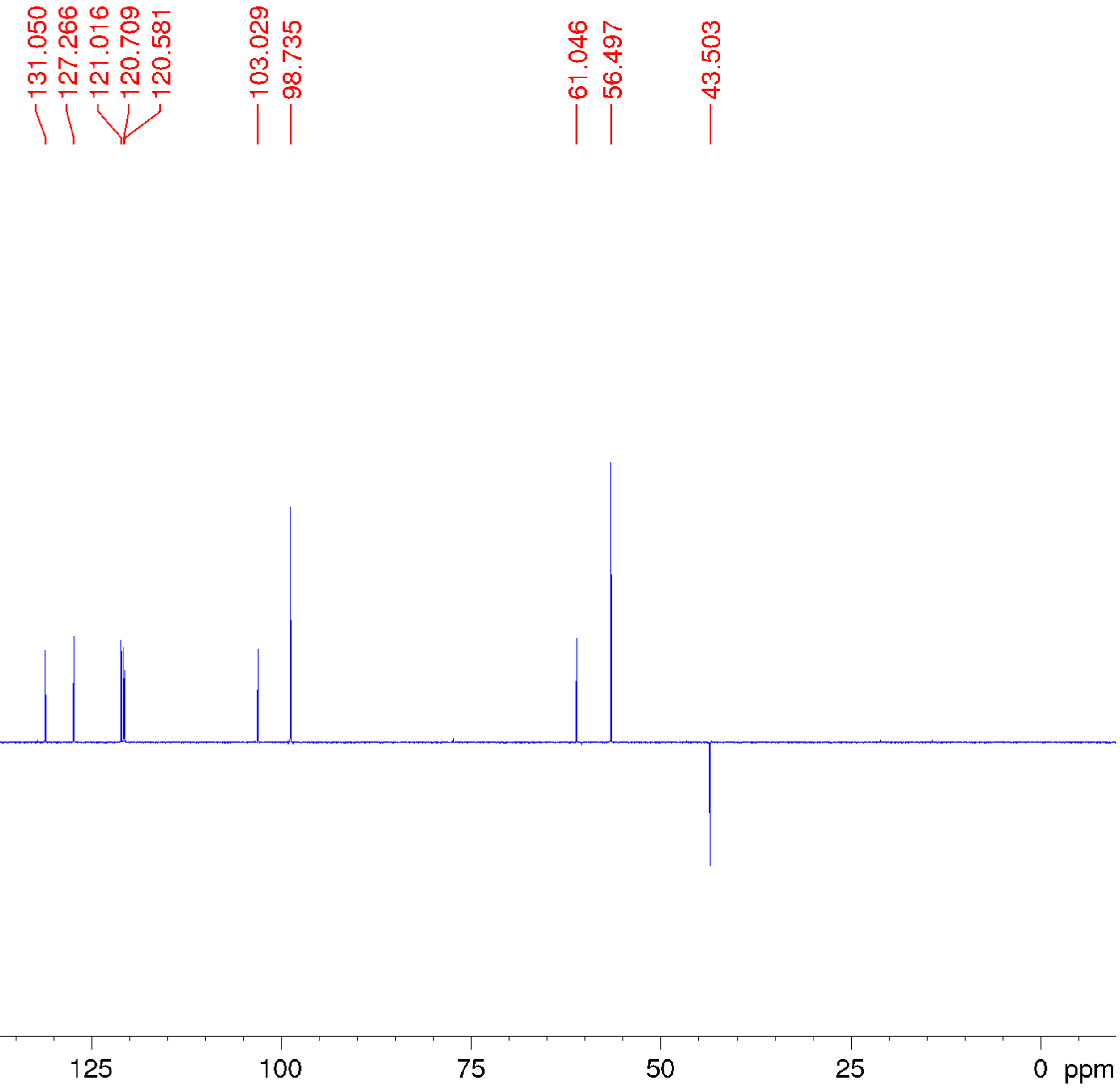

^{1}H NMR-spectrum (400 MHz, $CDCl_3$)

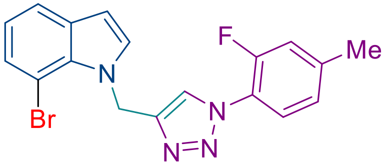

4d

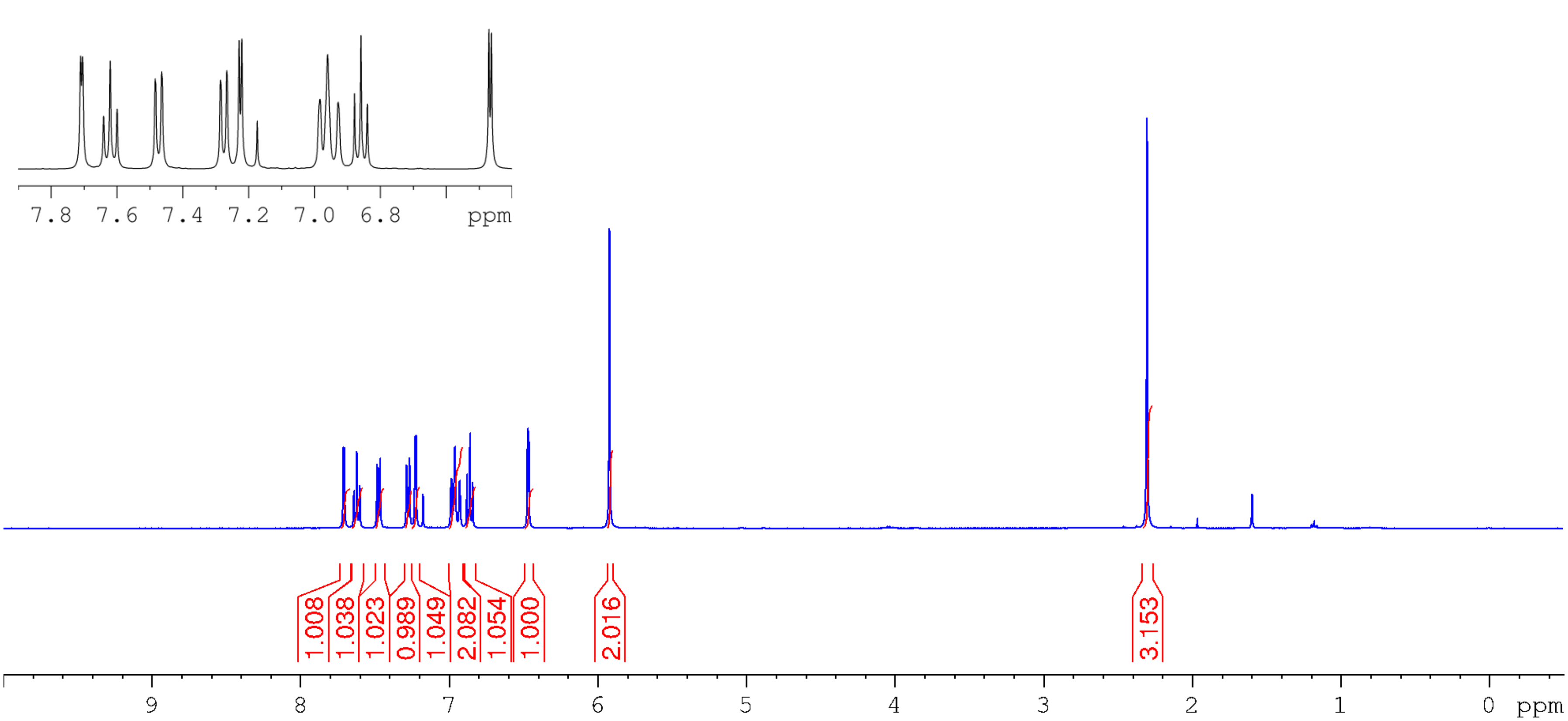

^{13}C NMR-spectrum (100 MHz, $CDCl_3$)

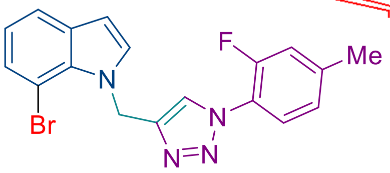

4d

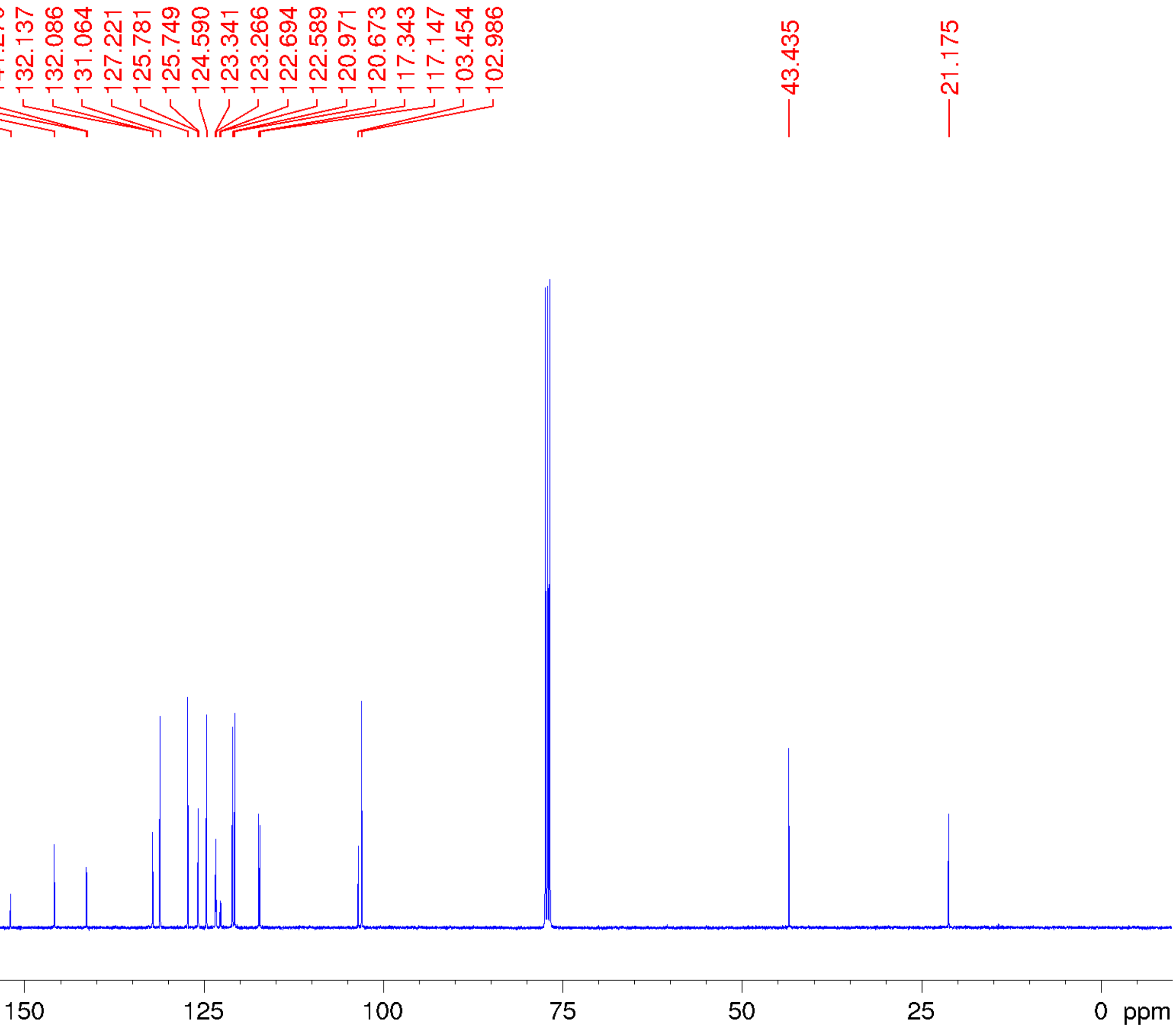

DEPT 135 NMR-spectrum ($CDCl_3$)

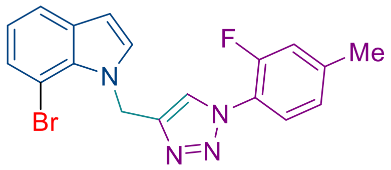

4d

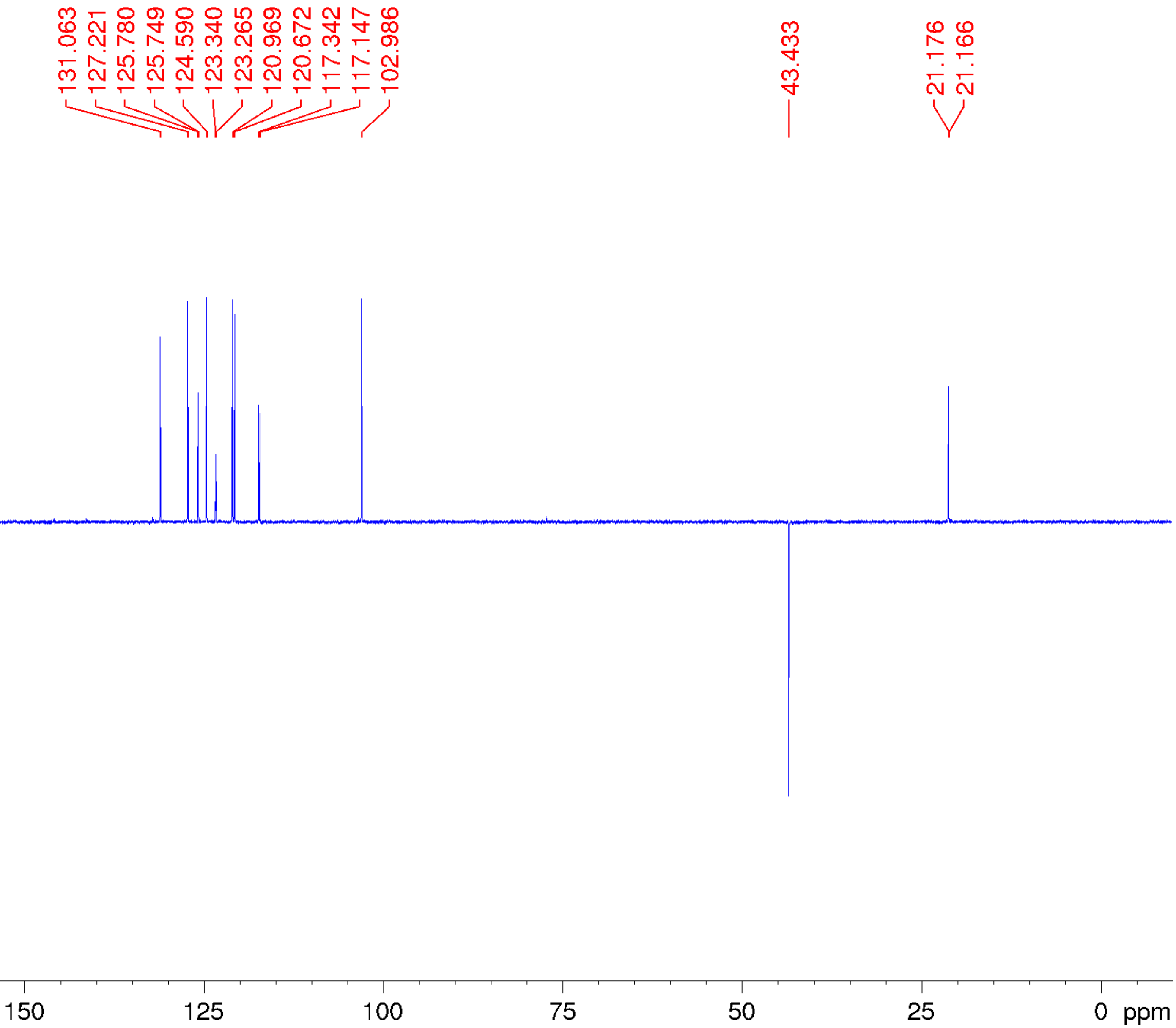

^{19}F NMR-spectrum (376.5 Hz, $CDCl_3$)

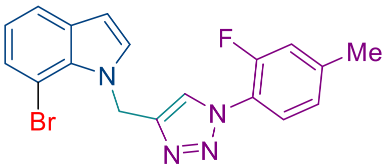

4d

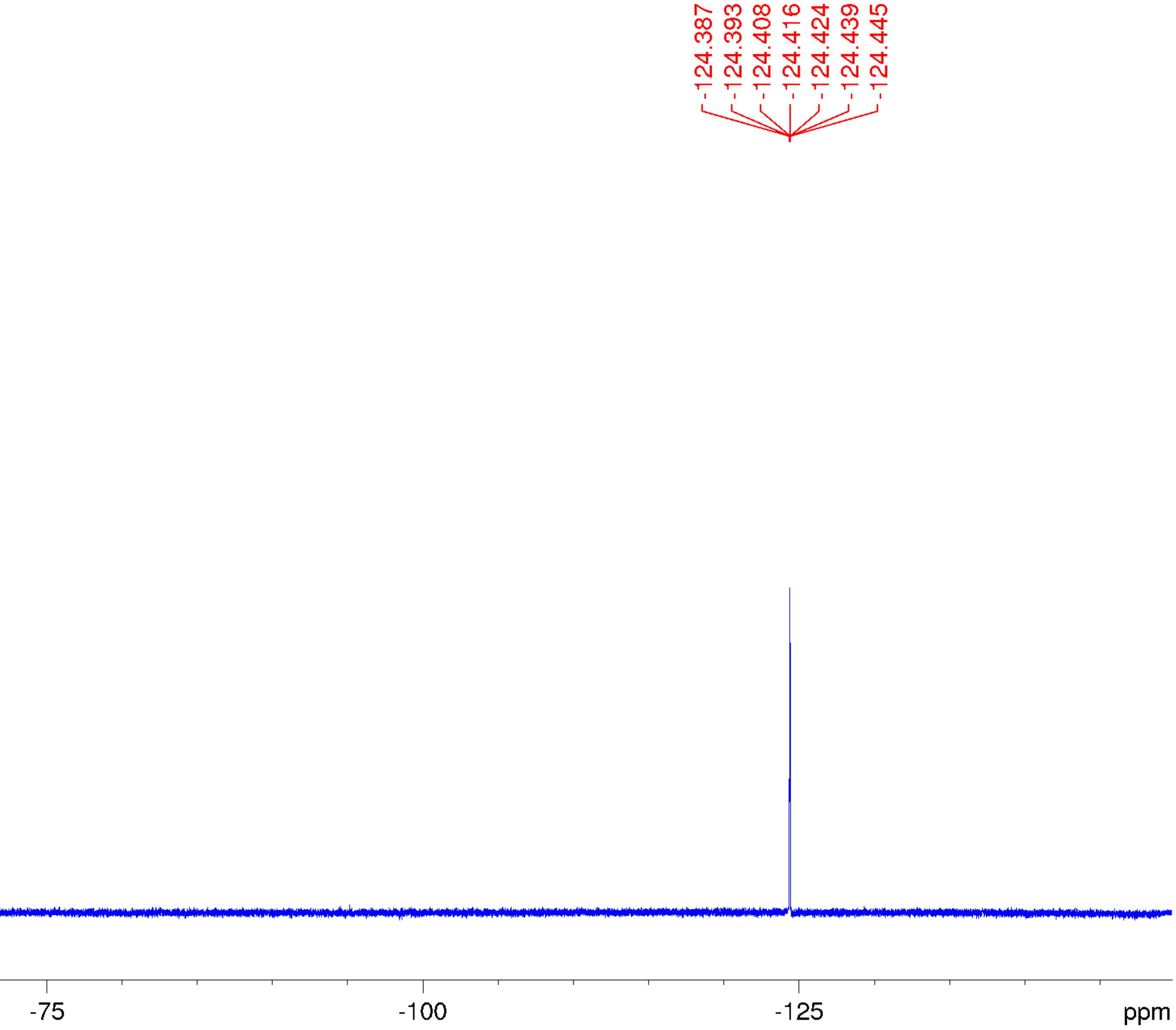

^{1}H NMR-spectrum (400 MHz, $CDCl_3$)

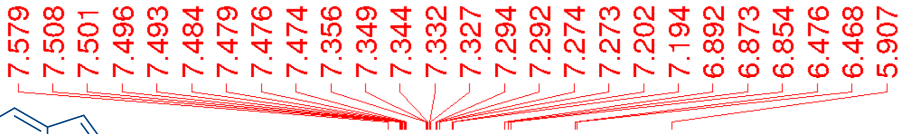

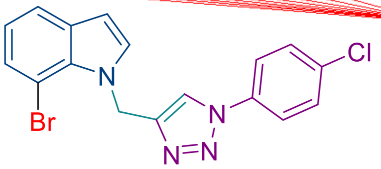

4e

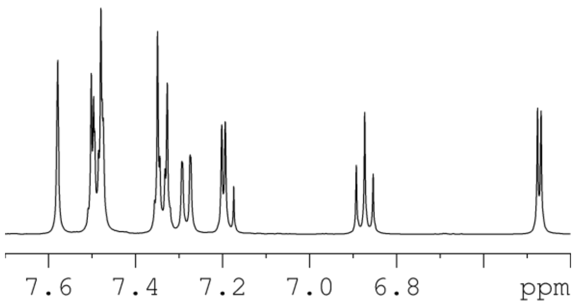

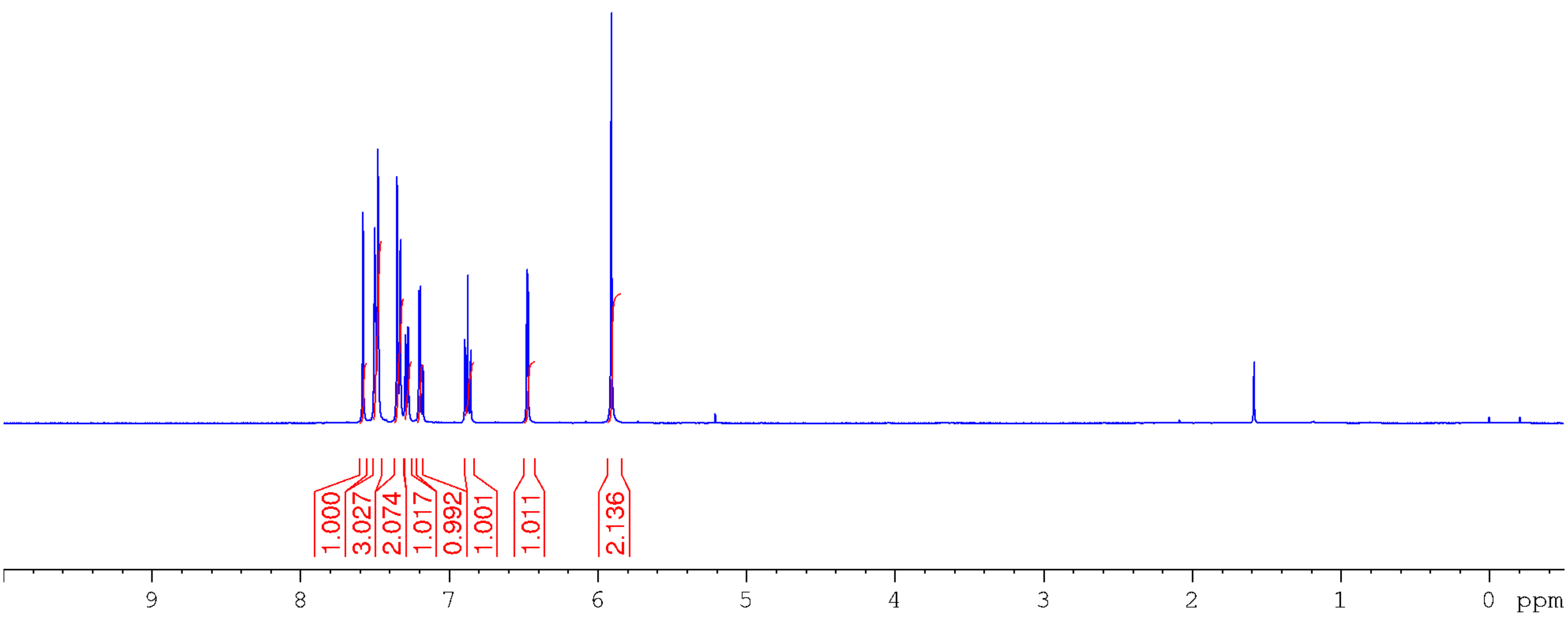

^{13}C NMR-spectrum (100 MHz, $CDCl_3$)

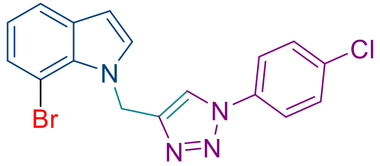

4e

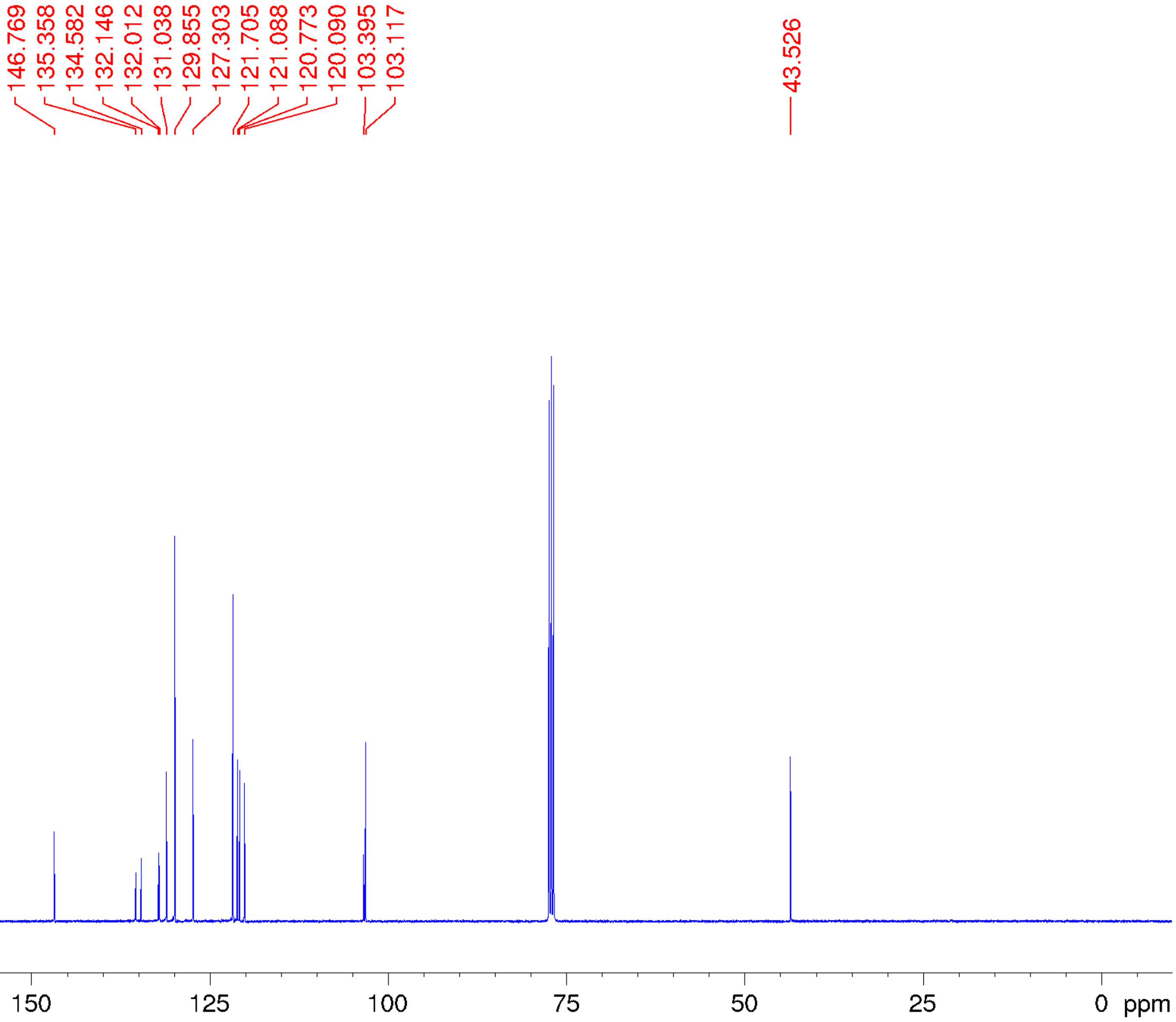

DEPT 135 NMR-spectrum ($CDCl_3$)

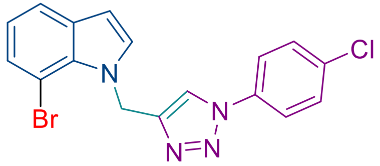

4e

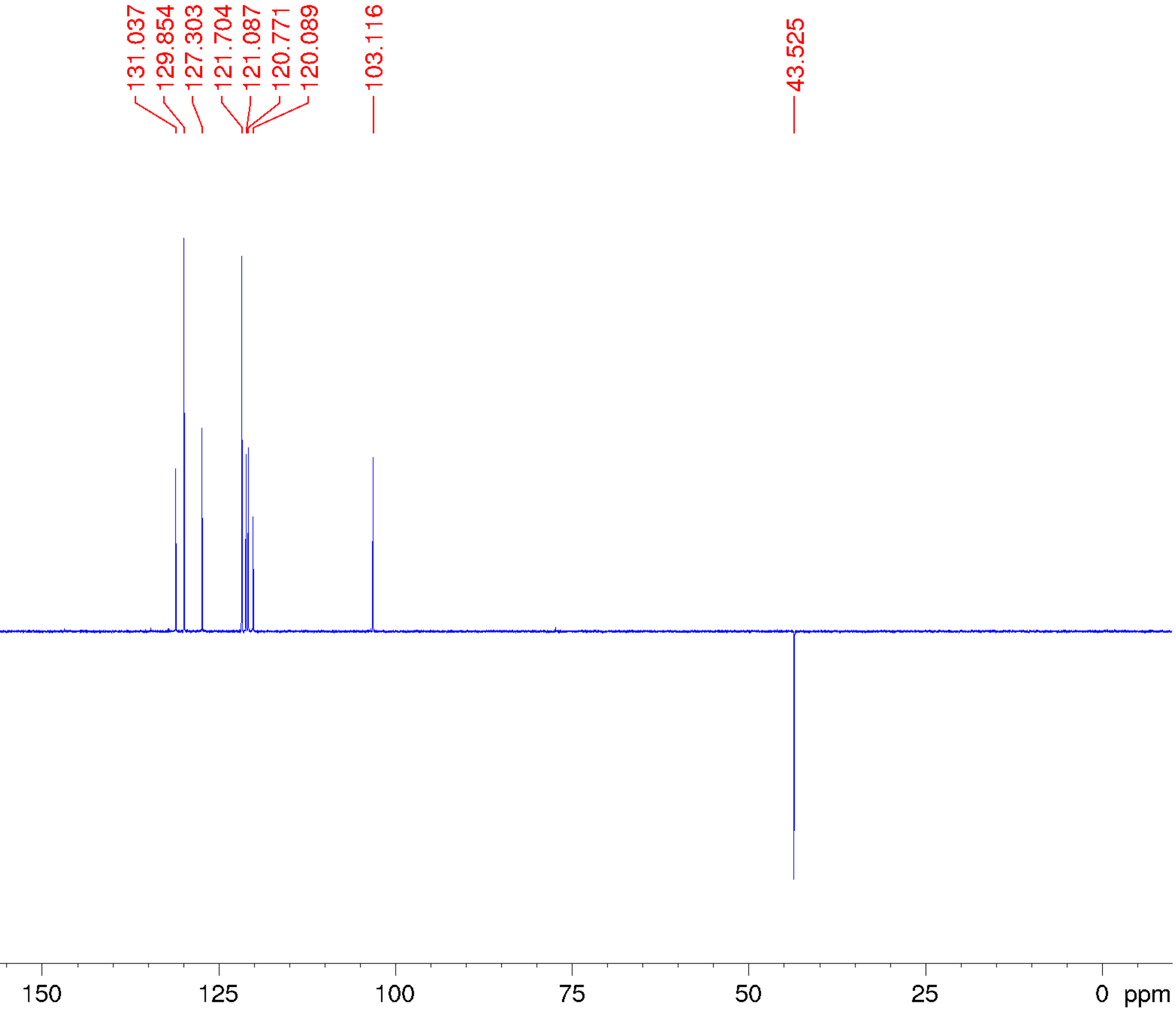

^{1}H NMR-spectrum (400 MHz, $CDCl_3$)

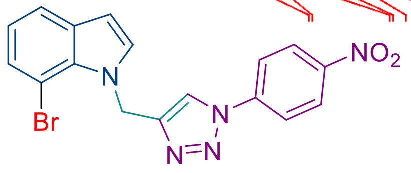

4f

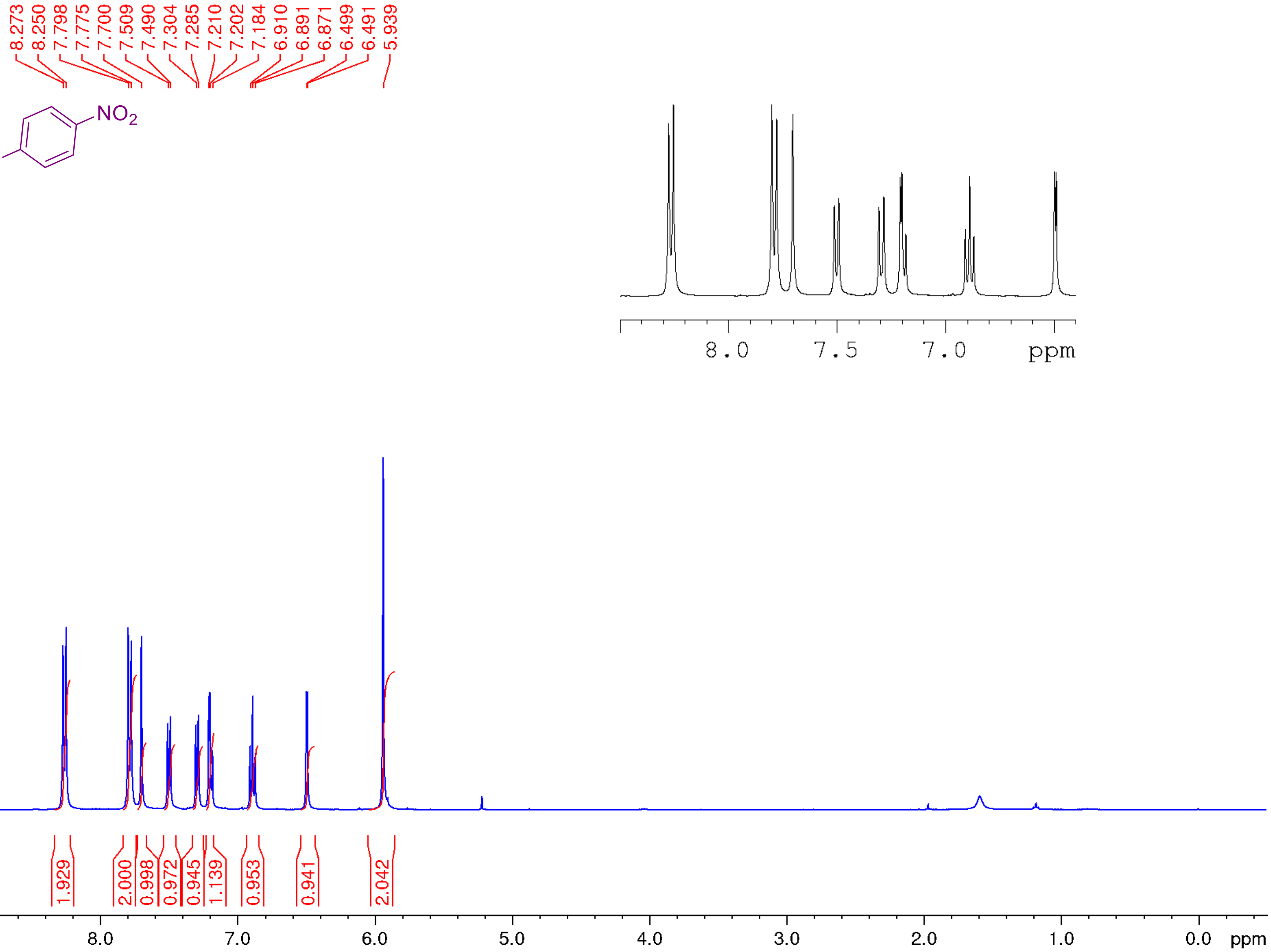

^{13}C NMR-spectrum (100 MHz, $CDCl_3$)

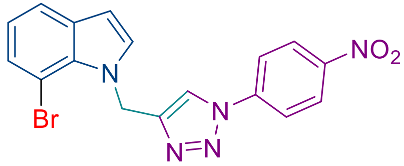

4f

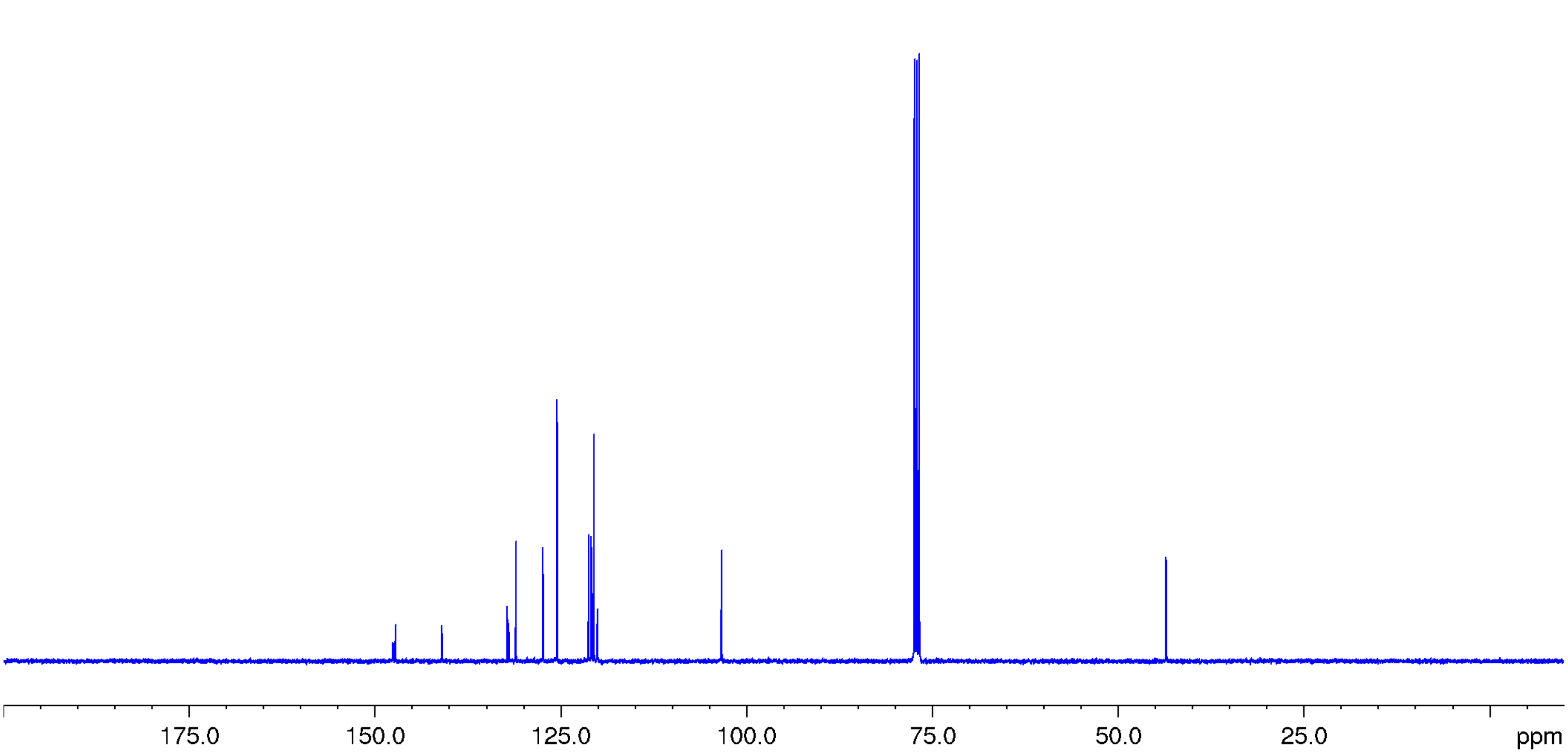

DEPT 135 NMR-spectrum ($CDCl_3$)

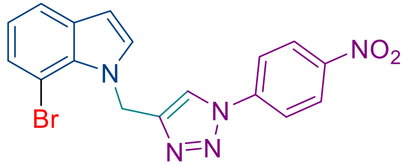

4f

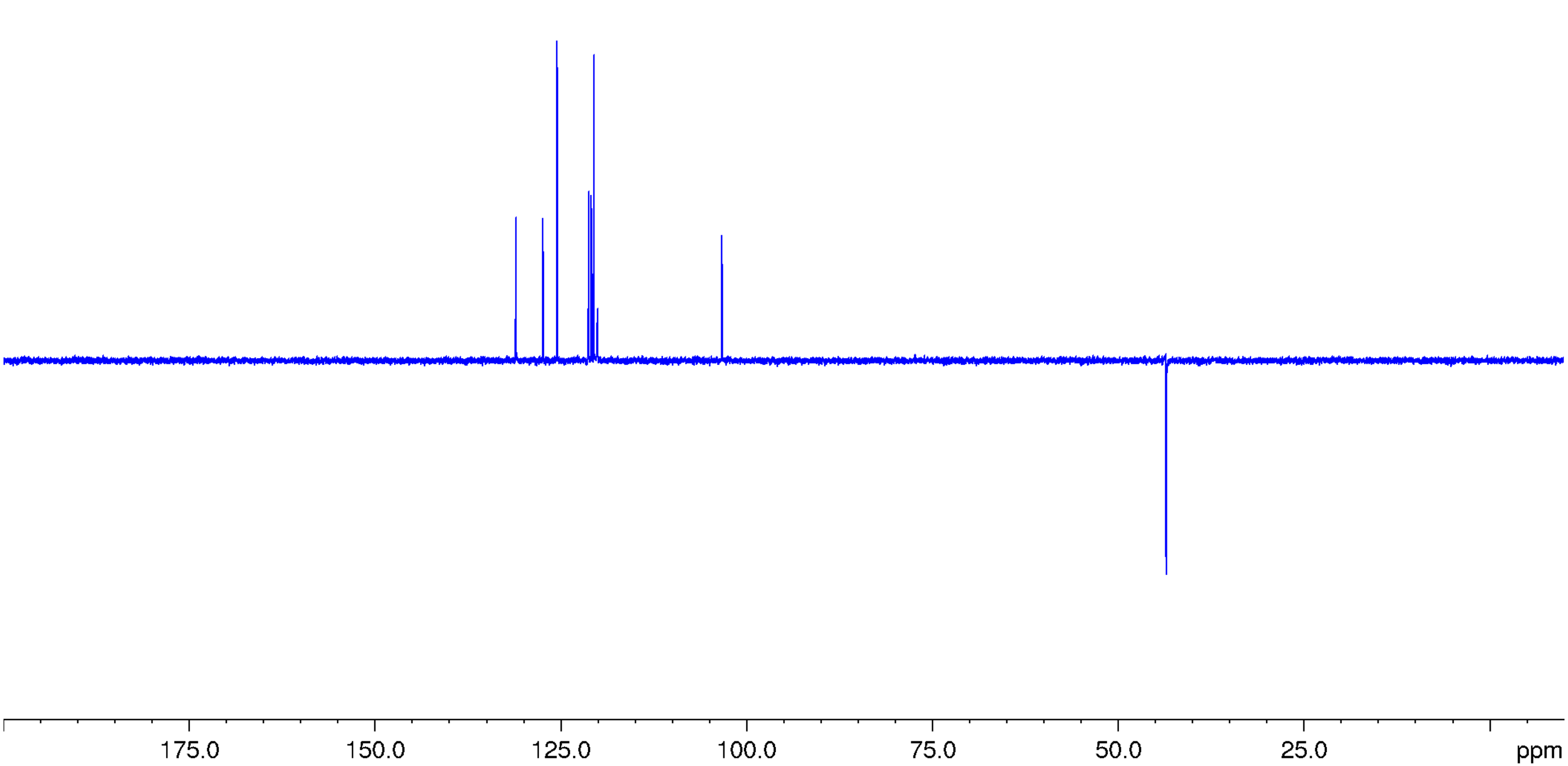

^{1}H NMR-spectrum (400 MHz, $CDCl_3$)

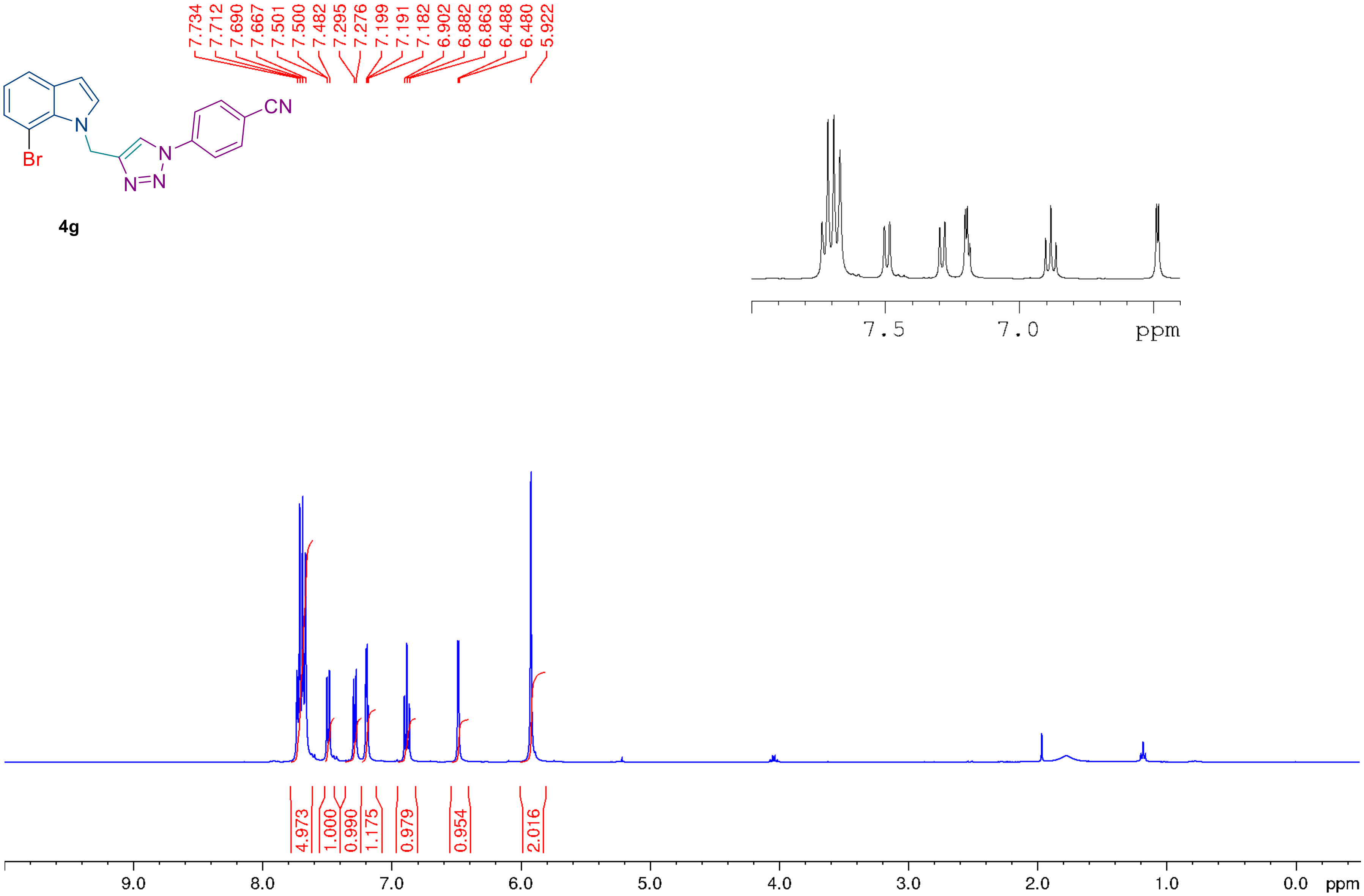

^{13}C NMR-spectrum (100 MHz, $CDCl_3$)

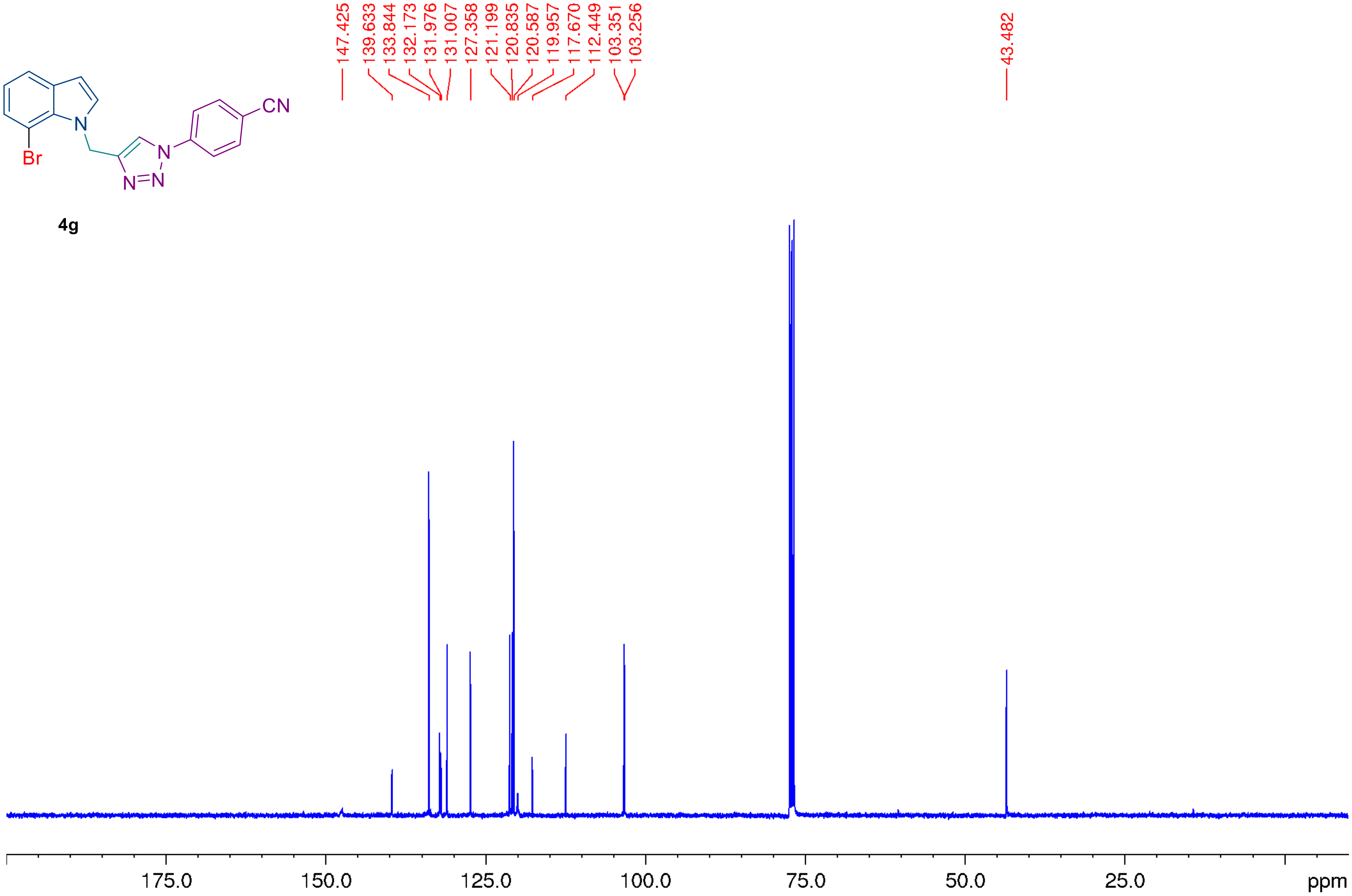

DEPT 135 NMR-spectrum ($CDCl_3$)

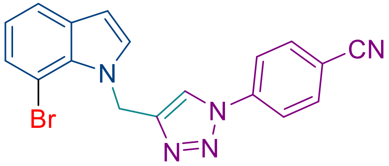

4g

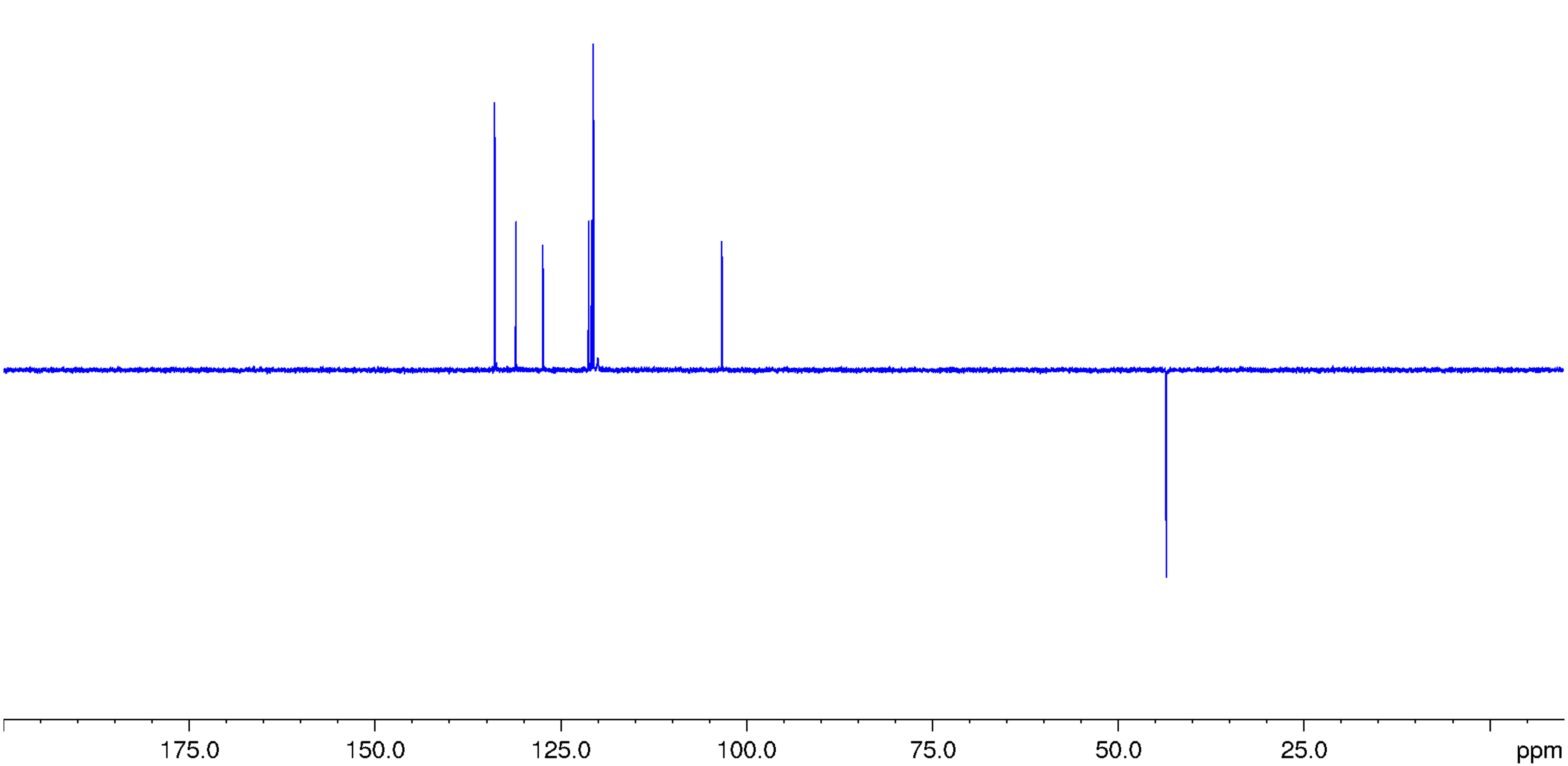

^{1}H NMR-spectrum (400 MHz, $CDCl_3$)

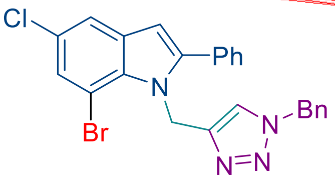

5a

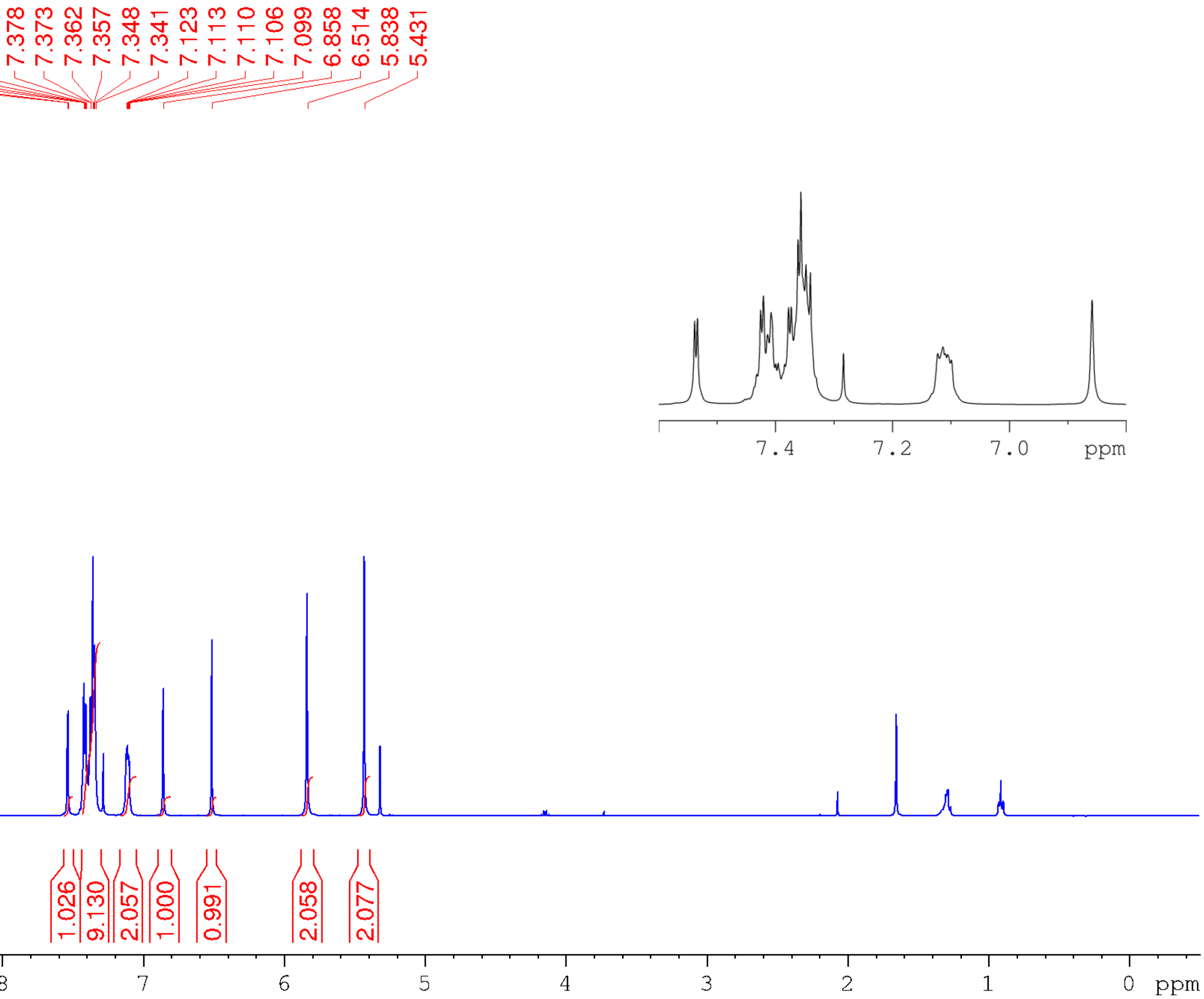

^{13}C NMR-spectrum (100 MHz, $CDCl_3$)

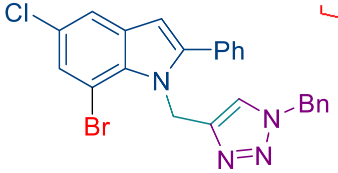

5a

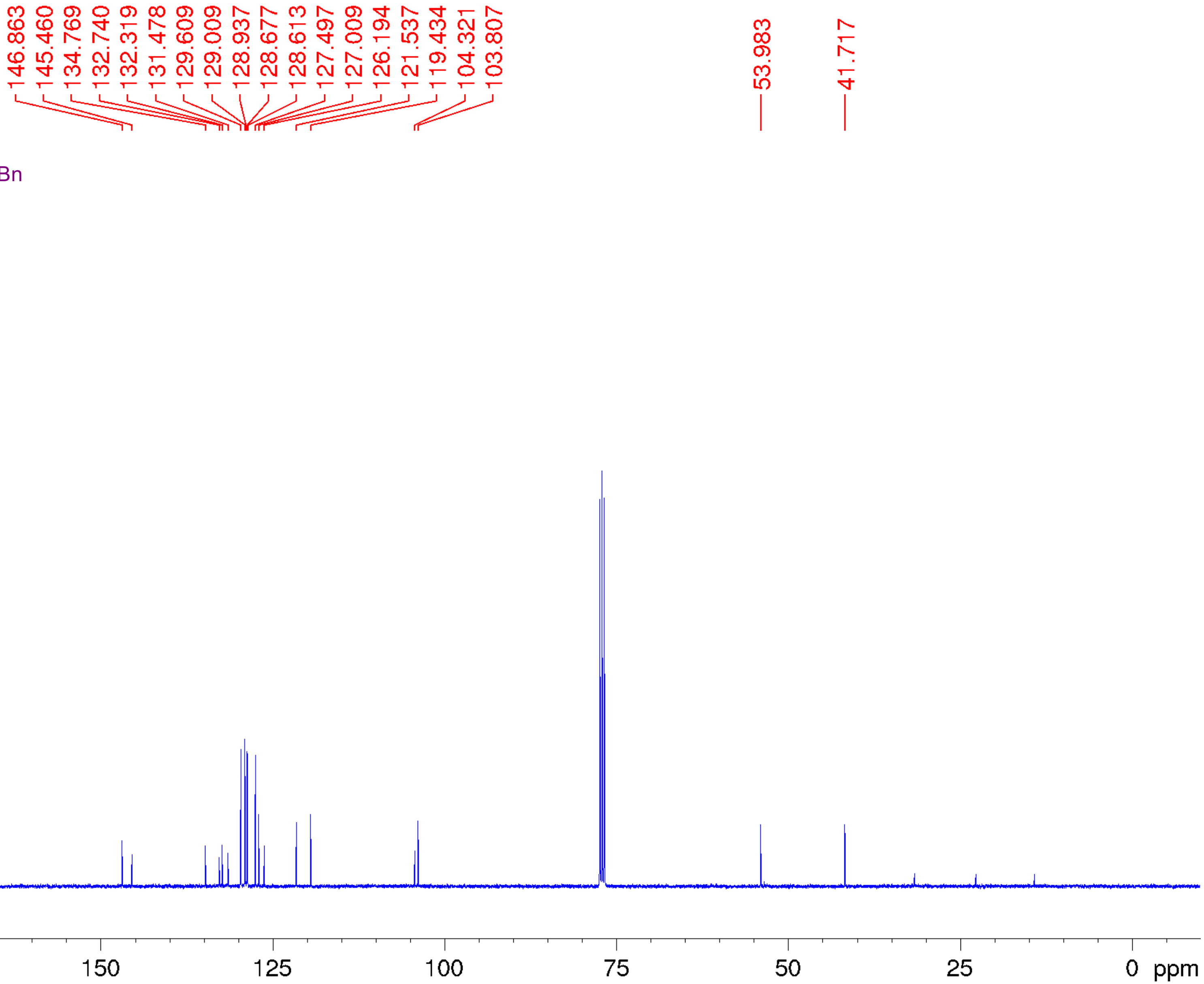

DEPT 135 NMR-spectrum ($CDCl_3$)

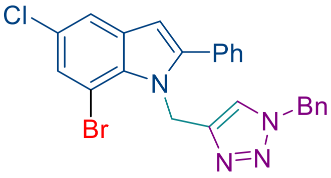

5a

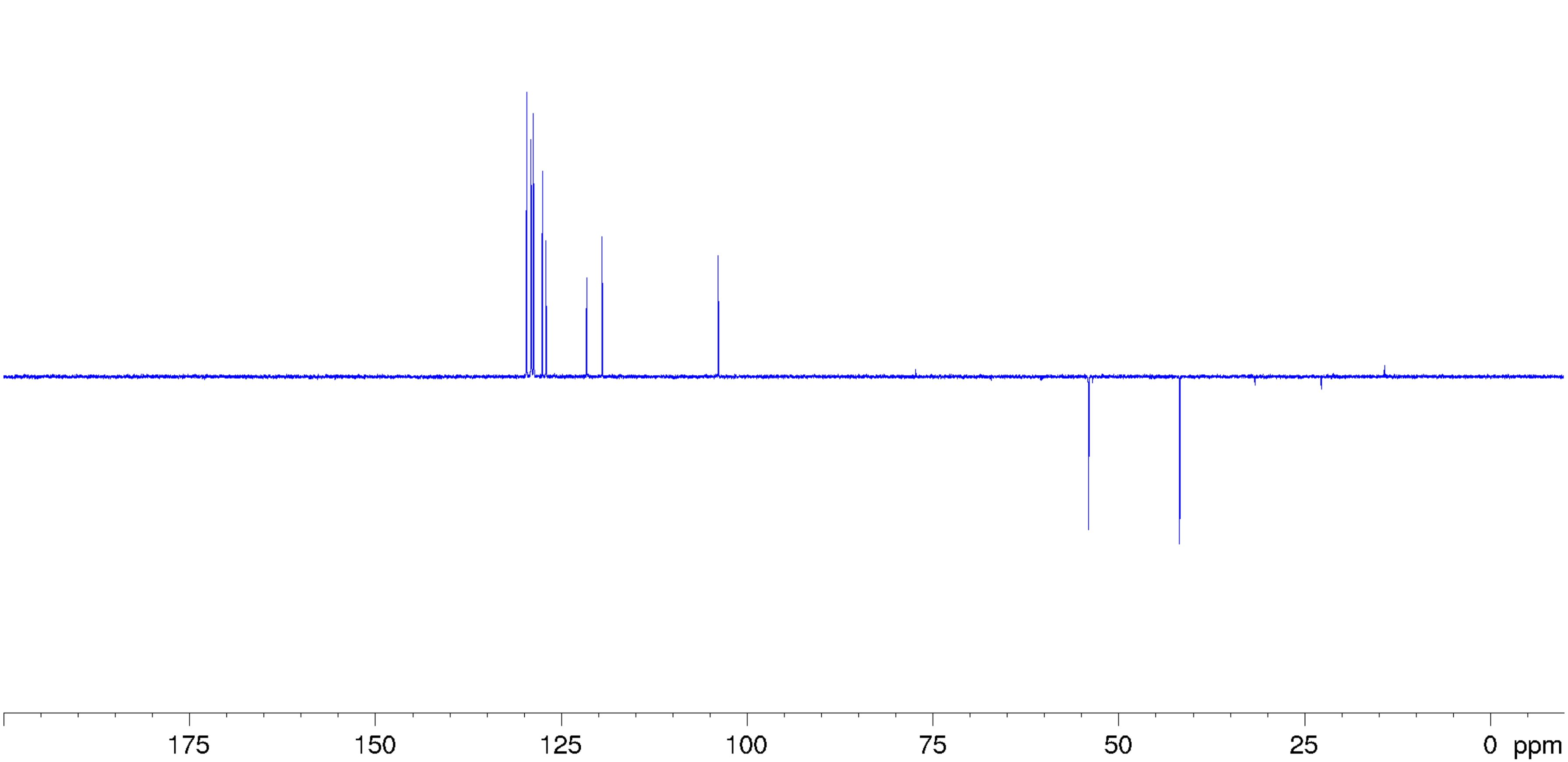

^{1}H NMR-spectrum (400 MHz, $CDCl_3$)

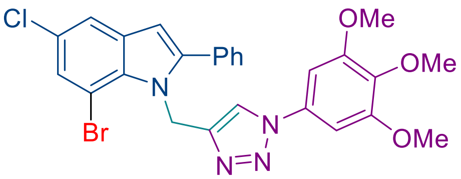

5b

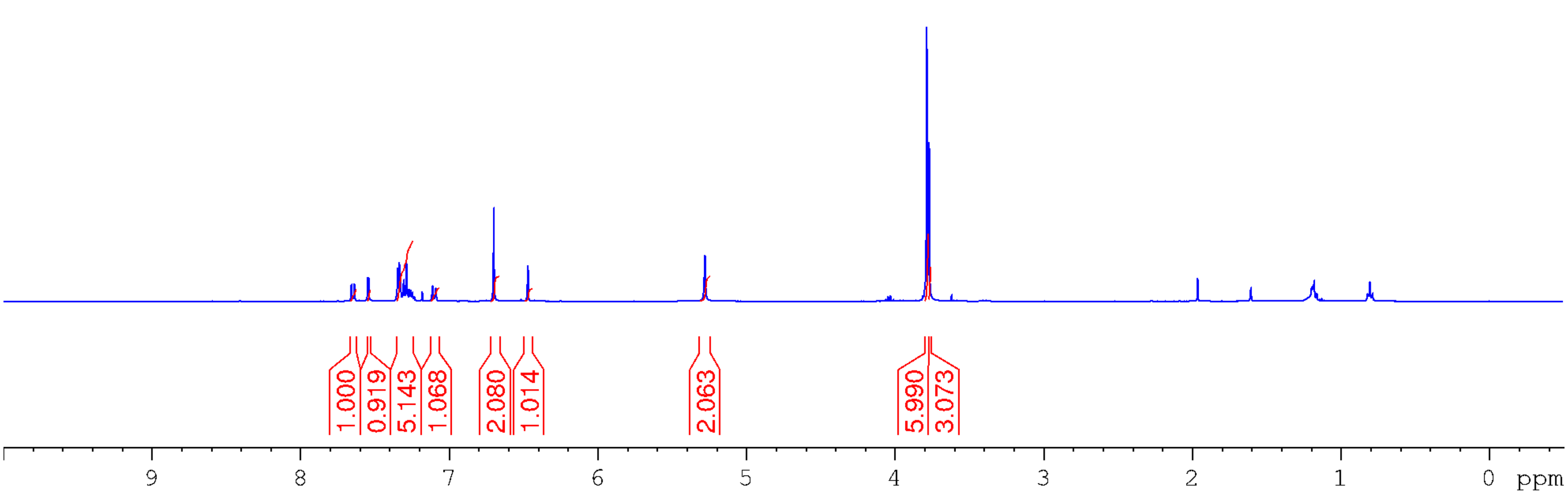

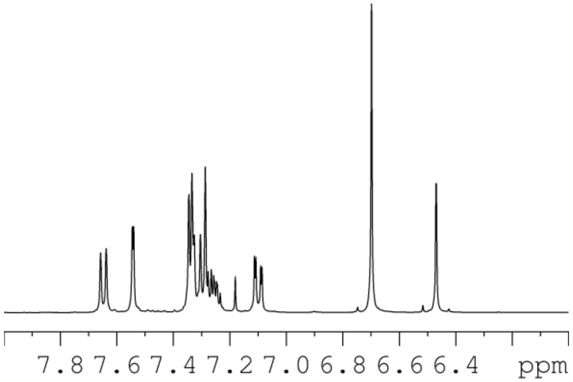

^{13}C NMR-spectrum (100 MHz, $CDCl_3$)

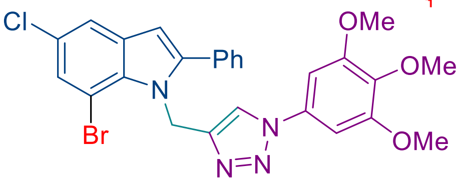

5b

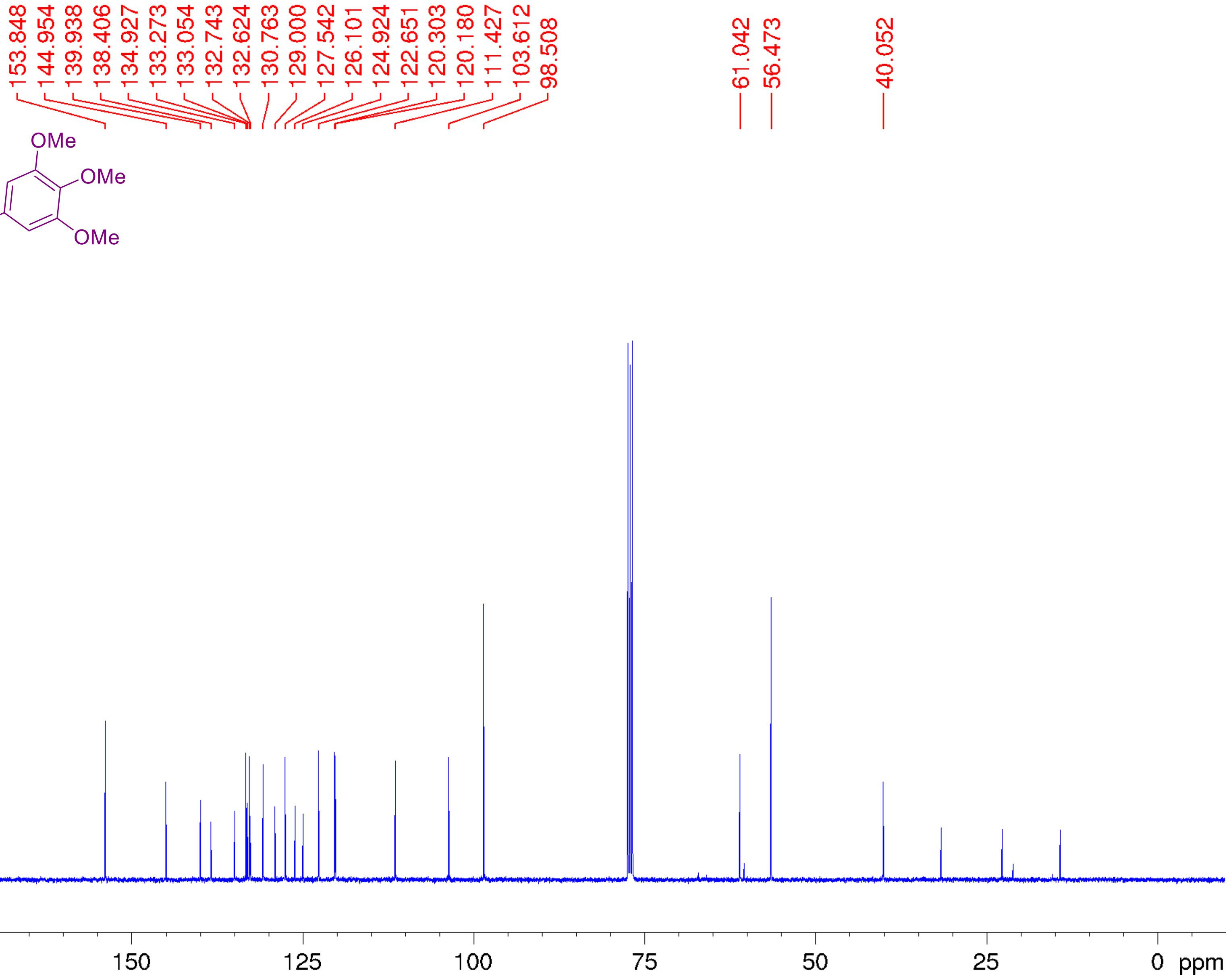

DEPT 135 NMR-spectrum ($CDCl_3$)

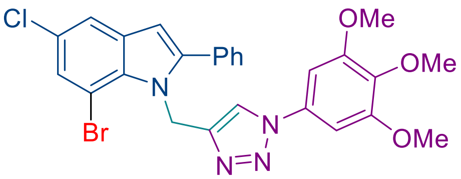

5b

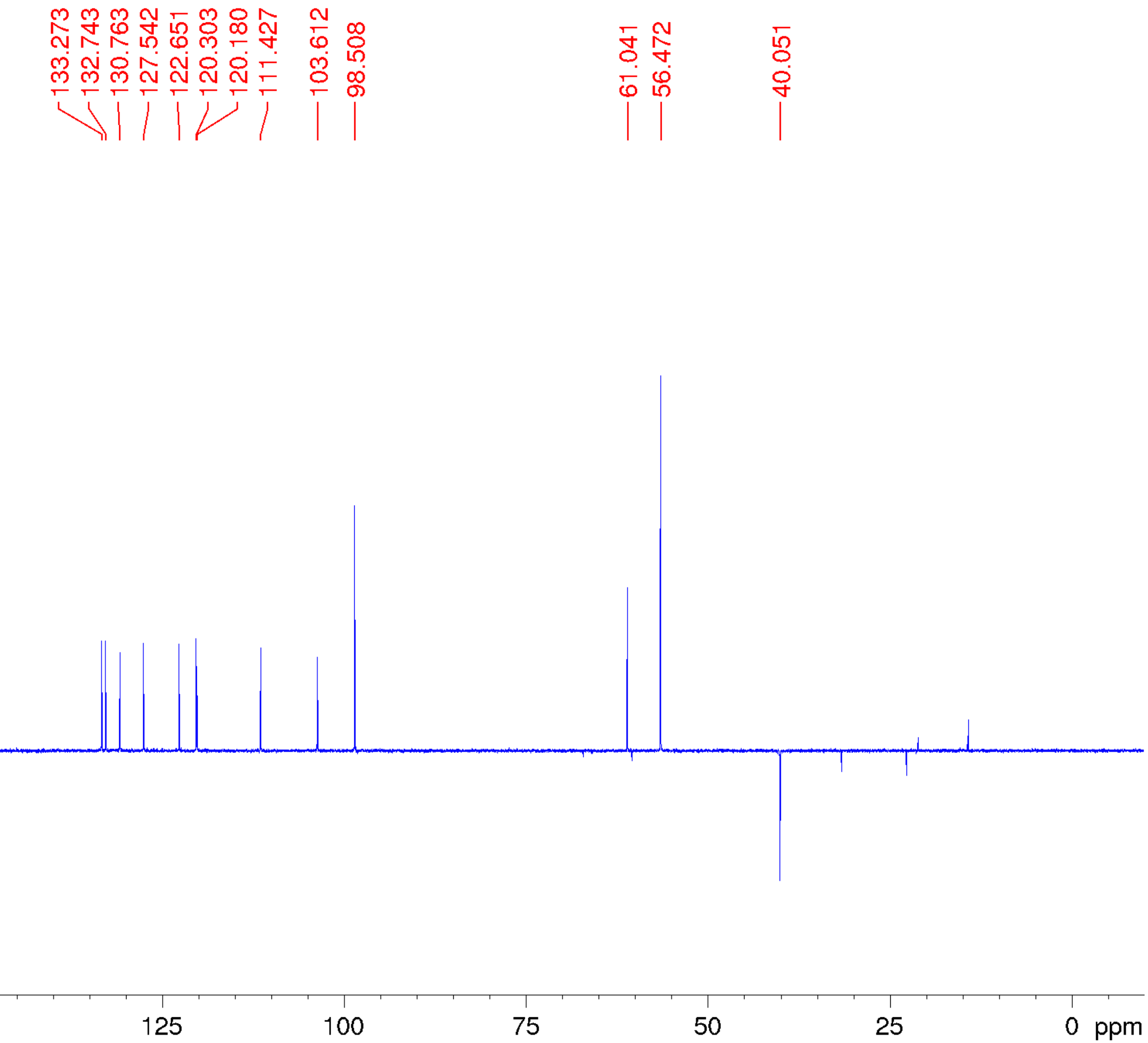

^{1}H NMR-spectrum (400 MHz, $CDCl_3$)

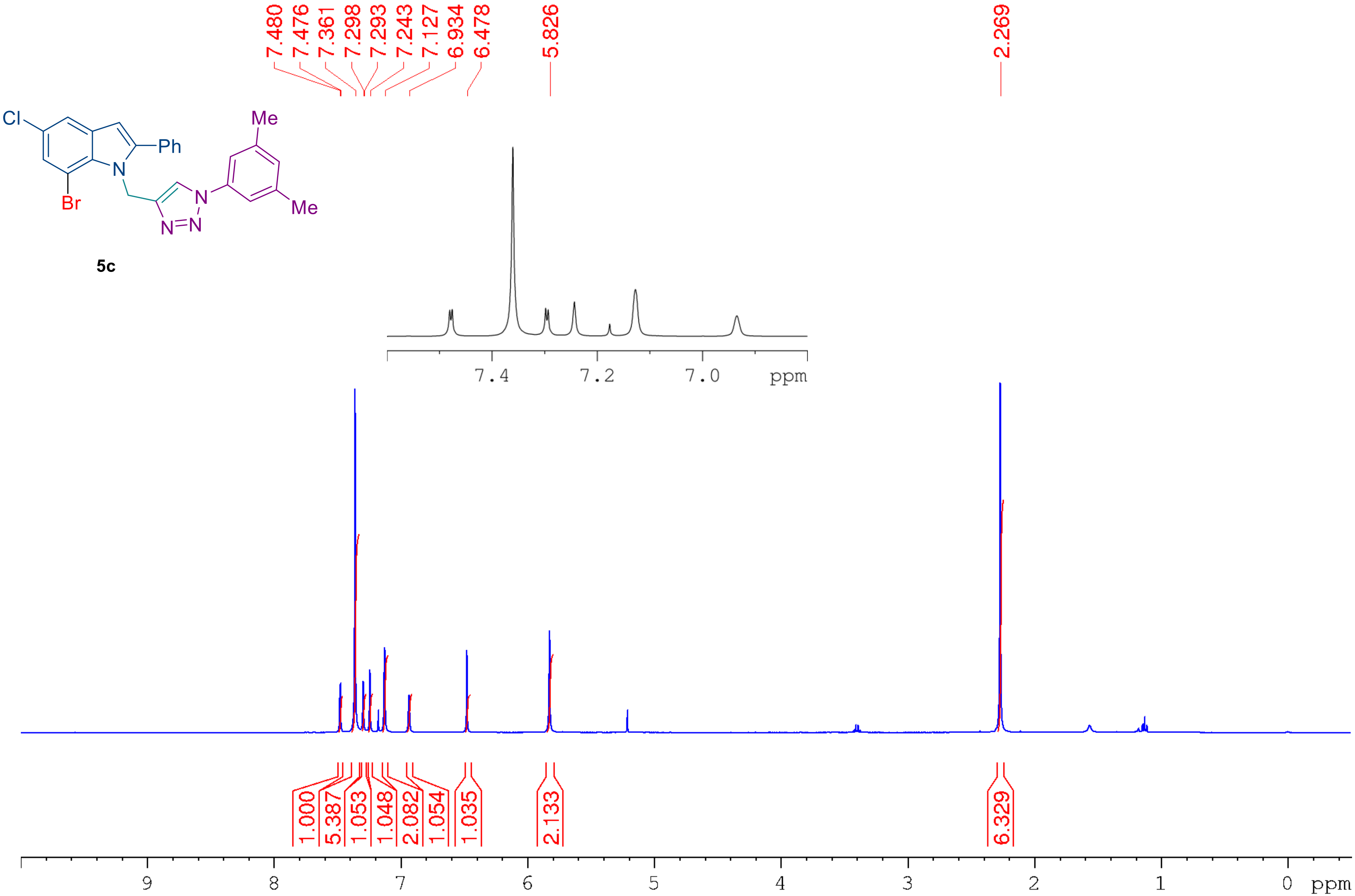

^{13}C NMR-spectrum (100 MHz, $CDCl_3$)

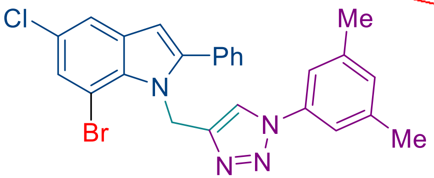

5c

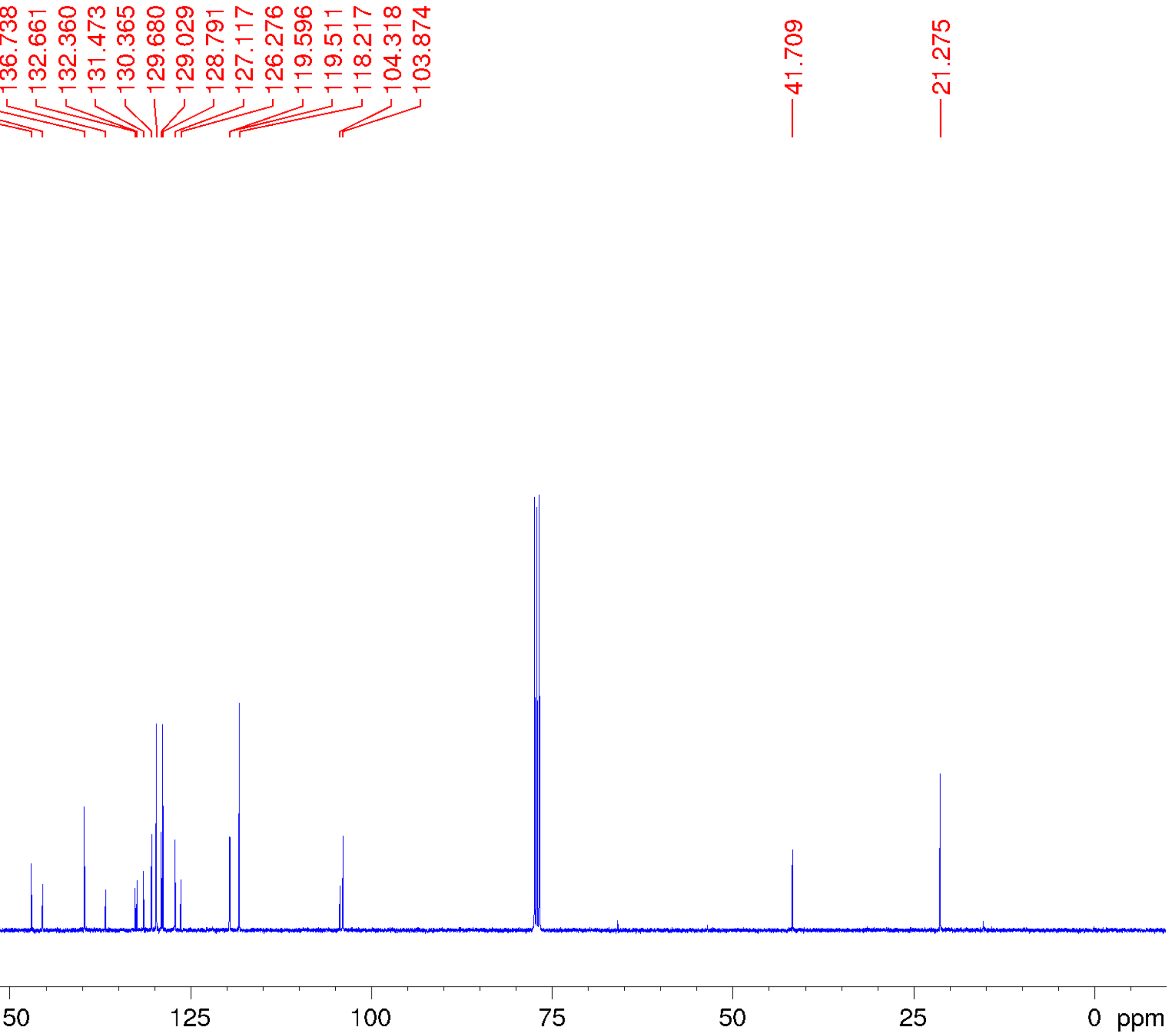

DEPT 135 NMR-spectrum ($CDCl_3$)

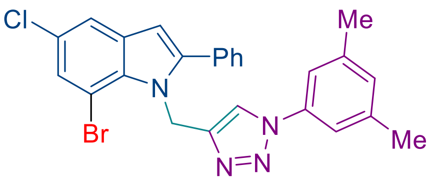

5c

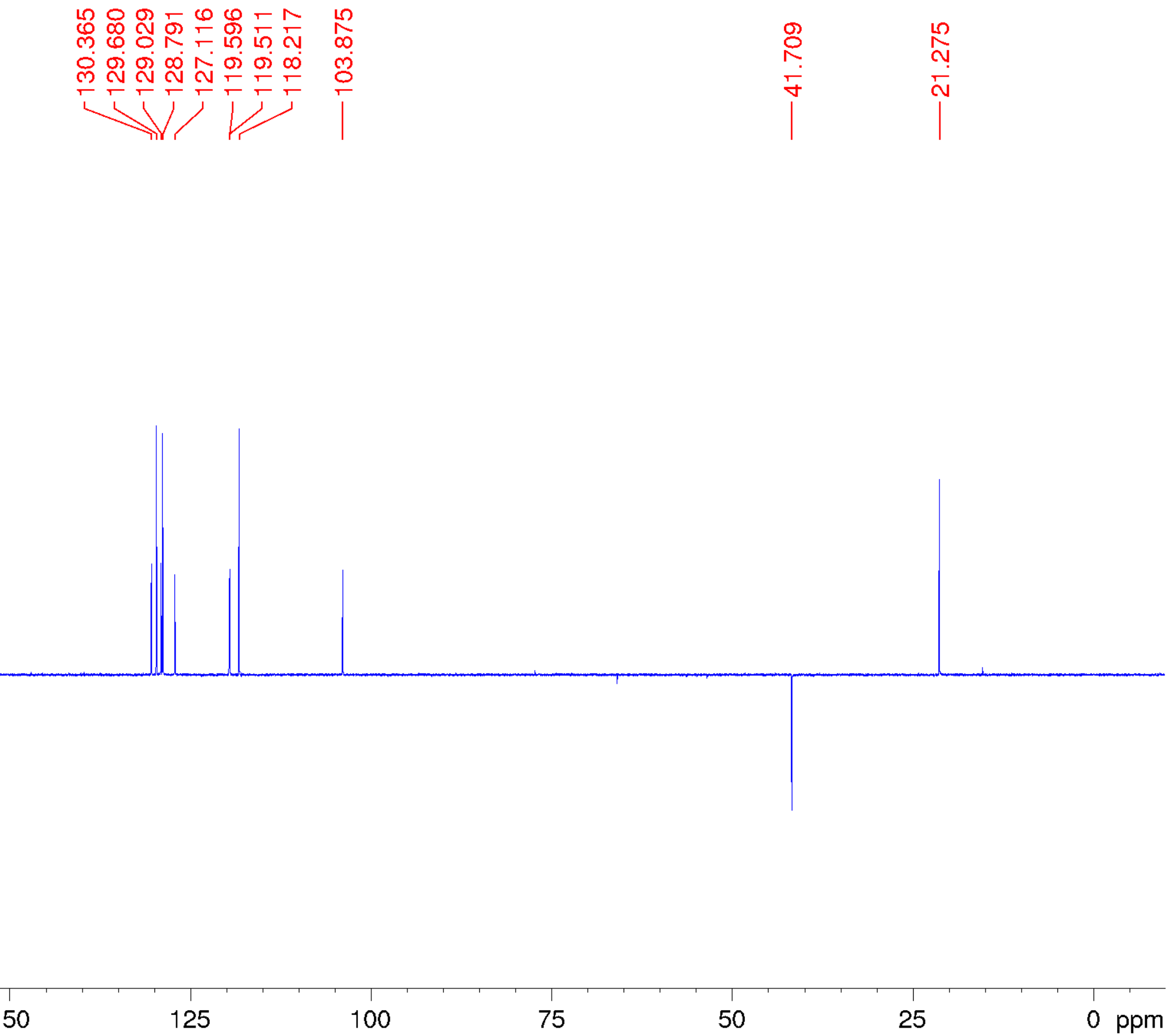

^{1}H NMR-spectrum (400 MHz, $CDCl_3$)

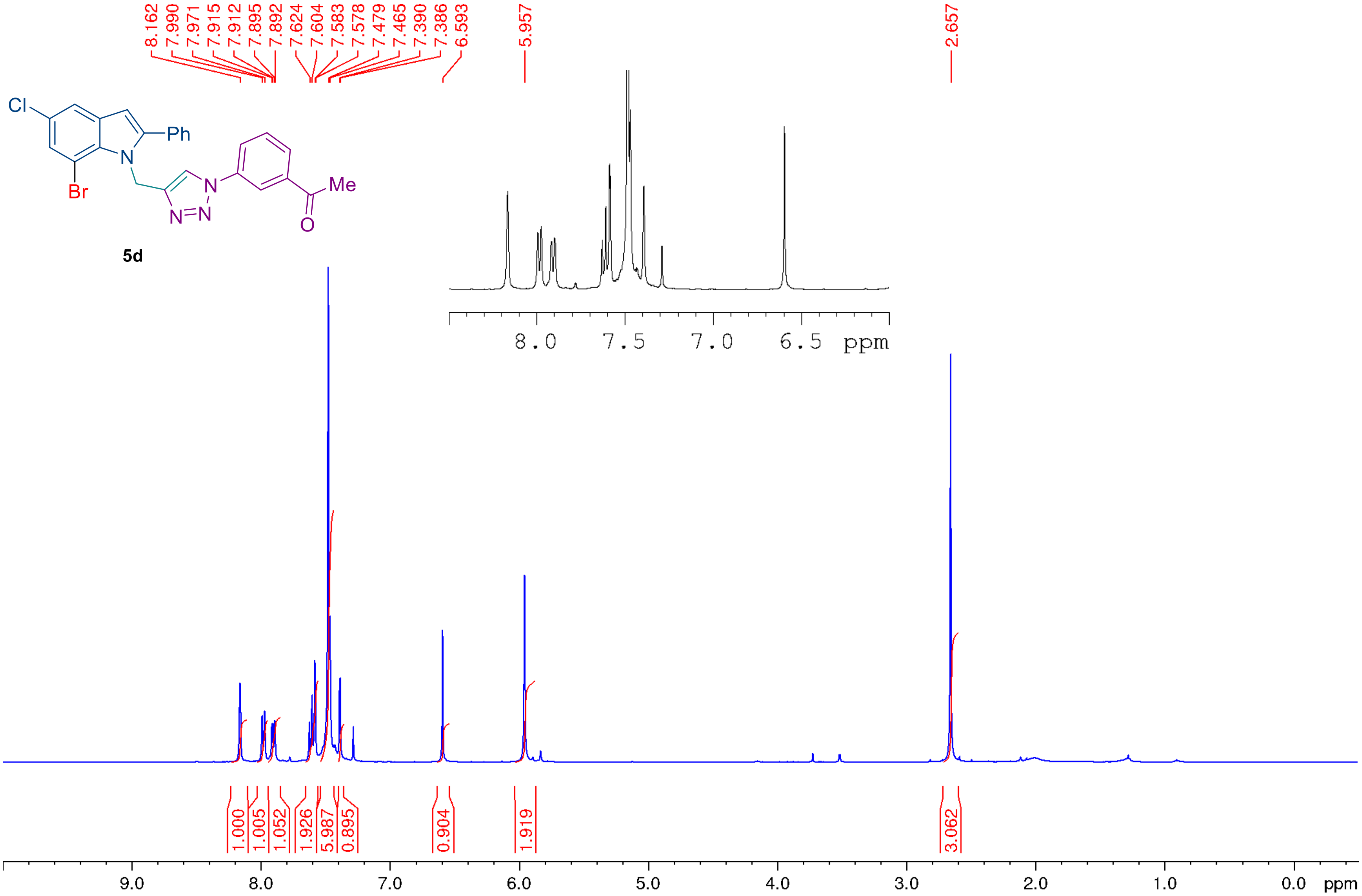

^{13}C NMR-spectrum (100 MHz, $CDCl_3$)

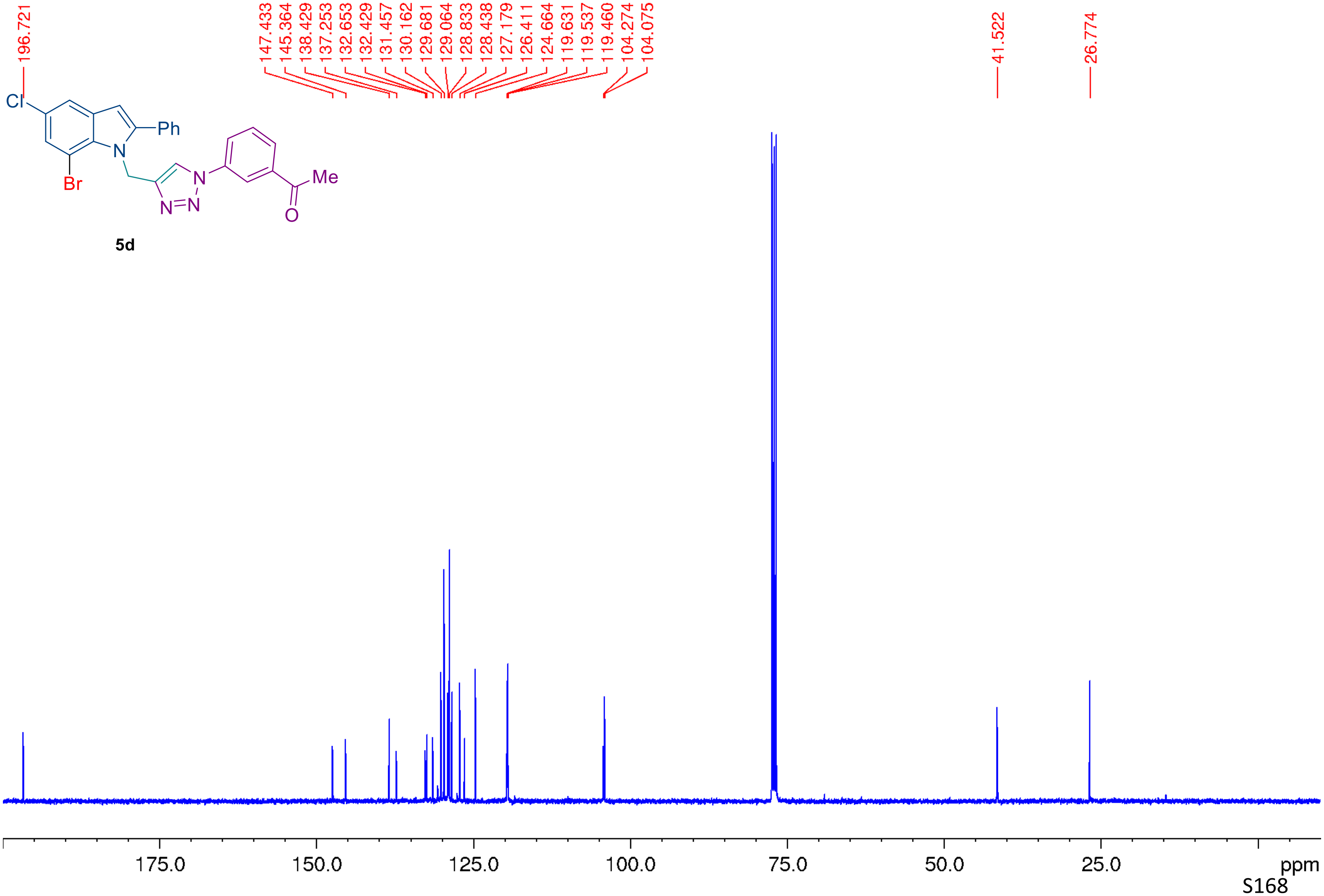

DEPT 135 NMR-spectrum ($CDCl_3$)

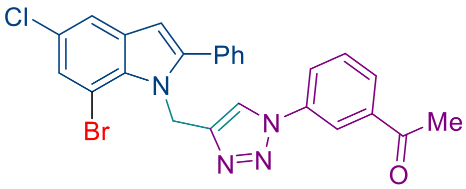

5d

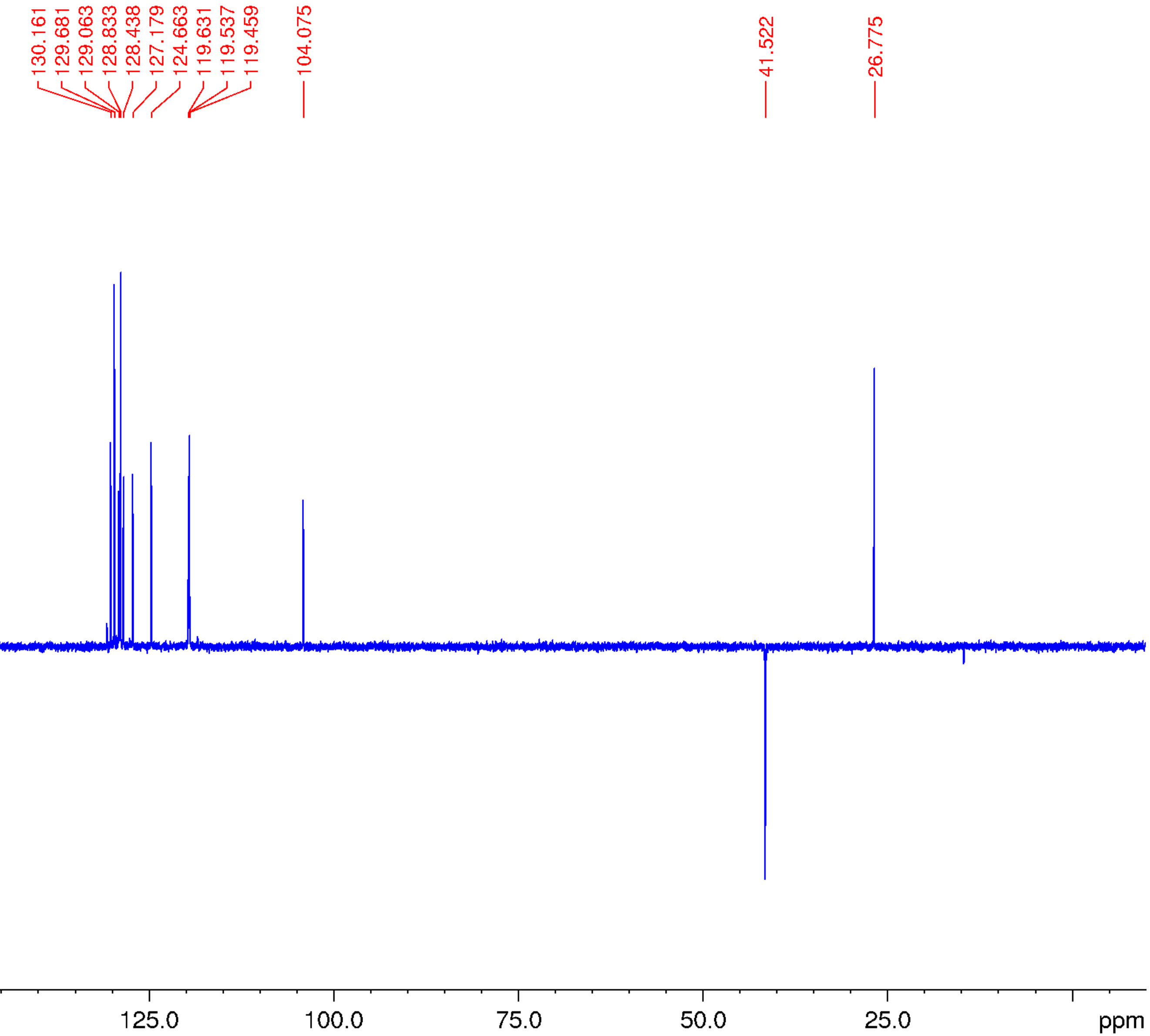

^{1}H NMR-spectrum (400 MHz, $CDCl_3$)

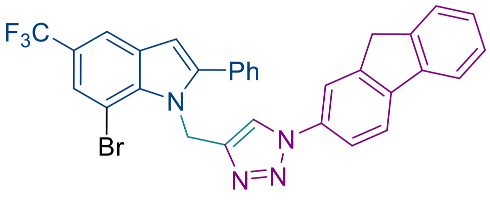

5e

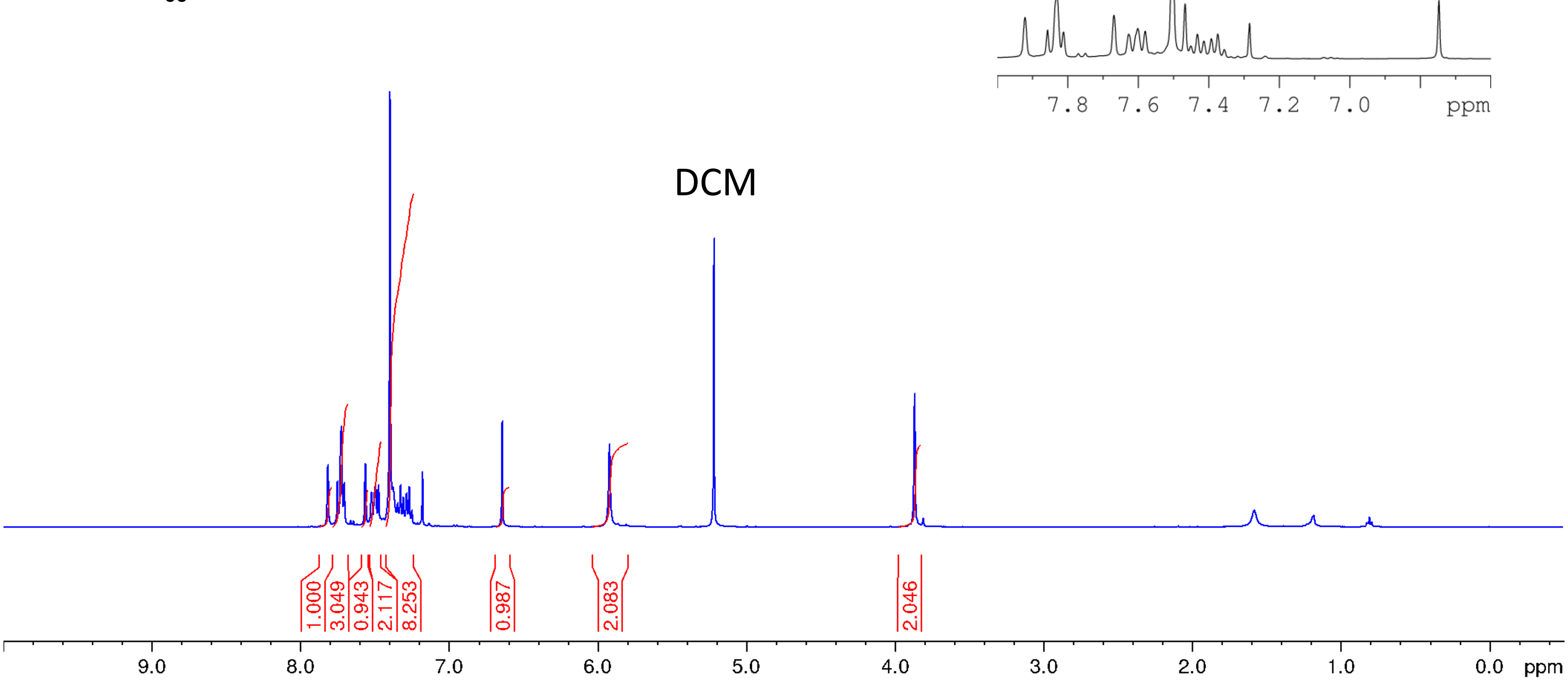

^{13}C NMR-spectrum (100 MHz, $CDCl_3$)

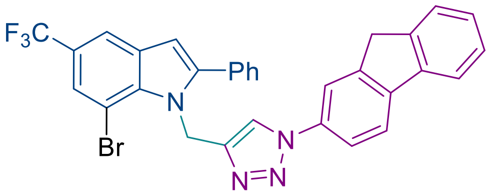

5e

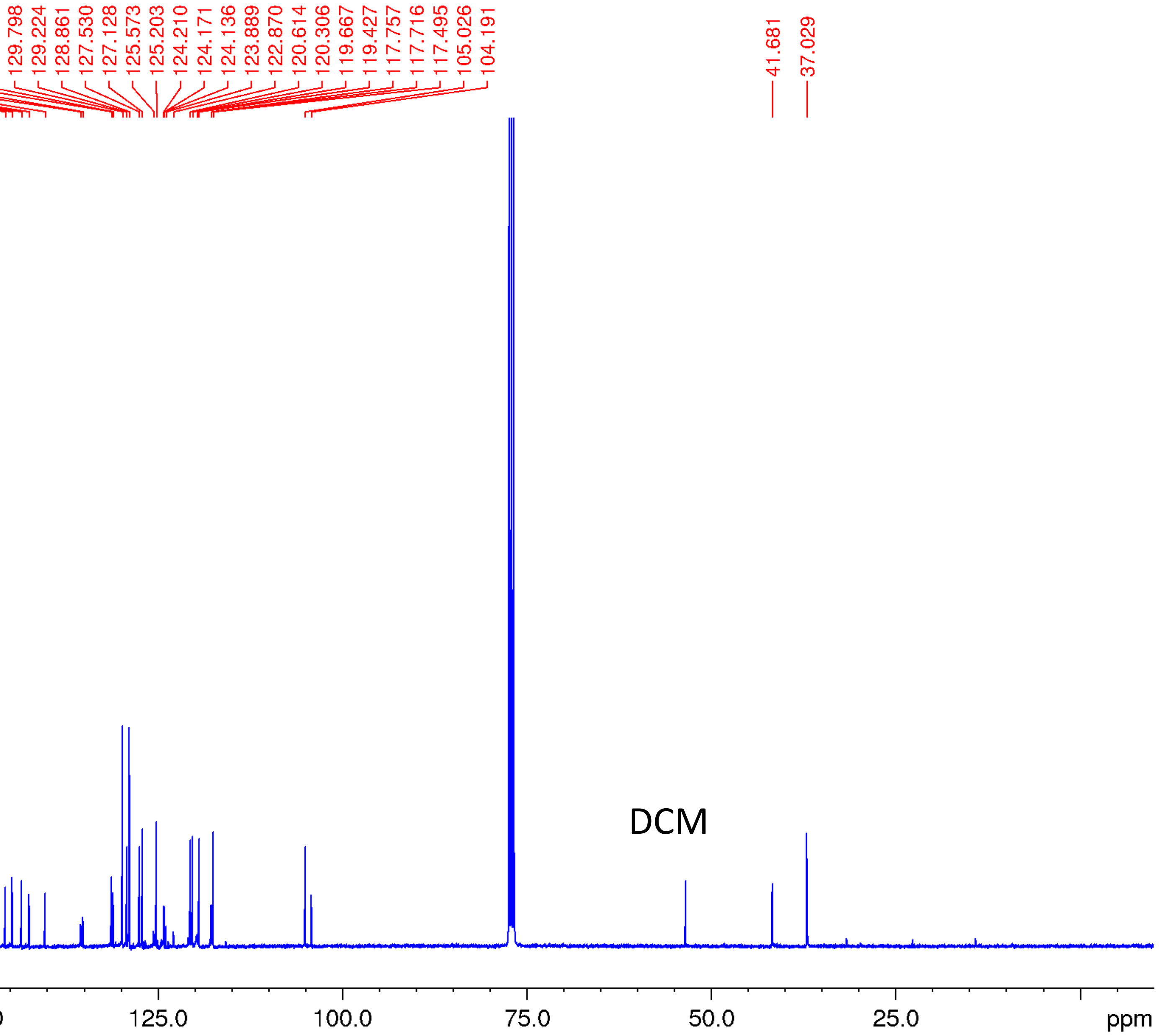

DEPT 135 NMR-spectrum ($CDCl_3$)

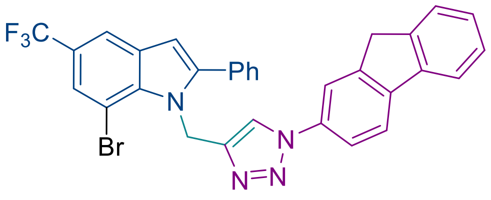

5e

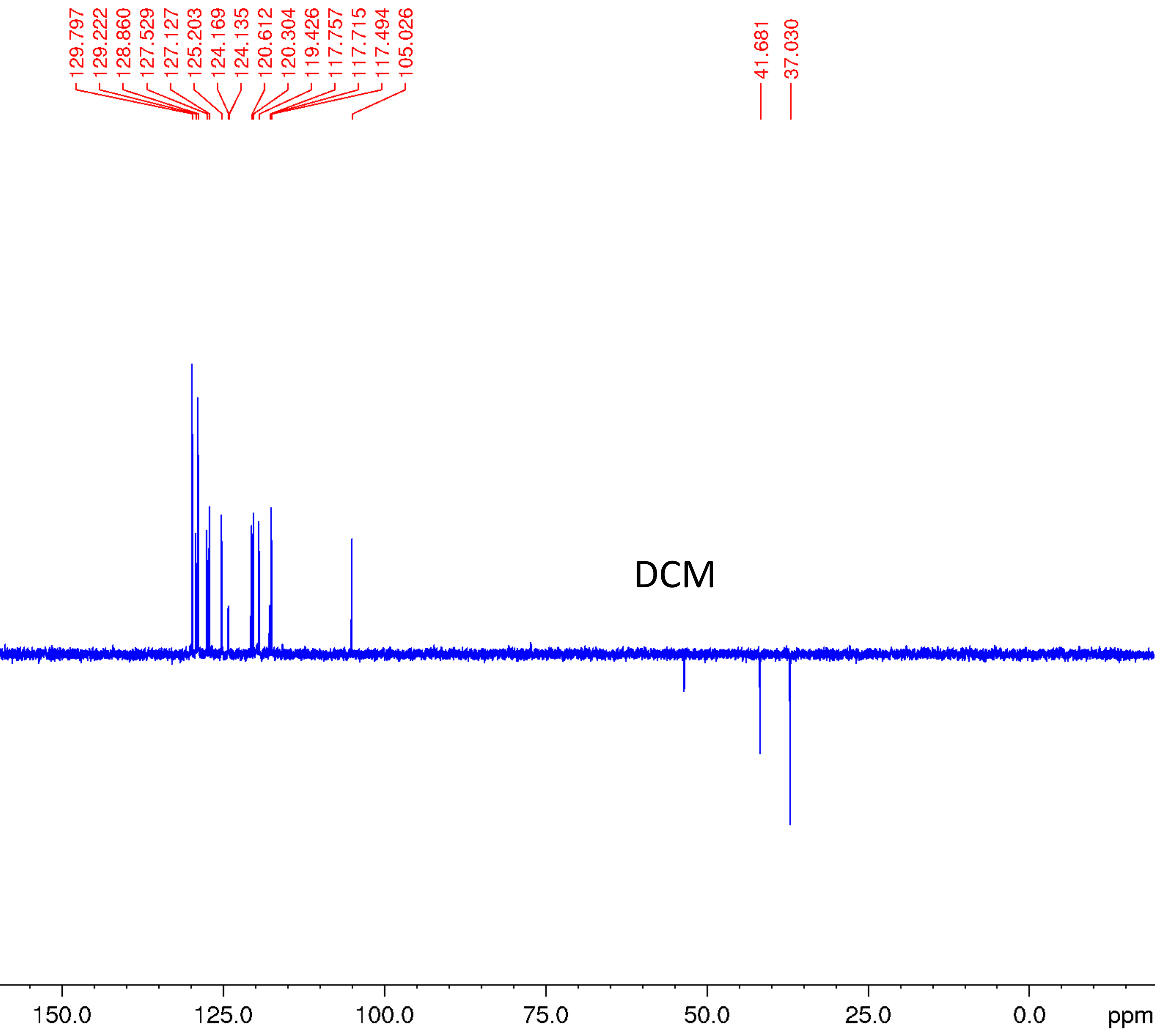

^{19}F NMR-spectrum (376.5 Hz, $CDCl_3$)

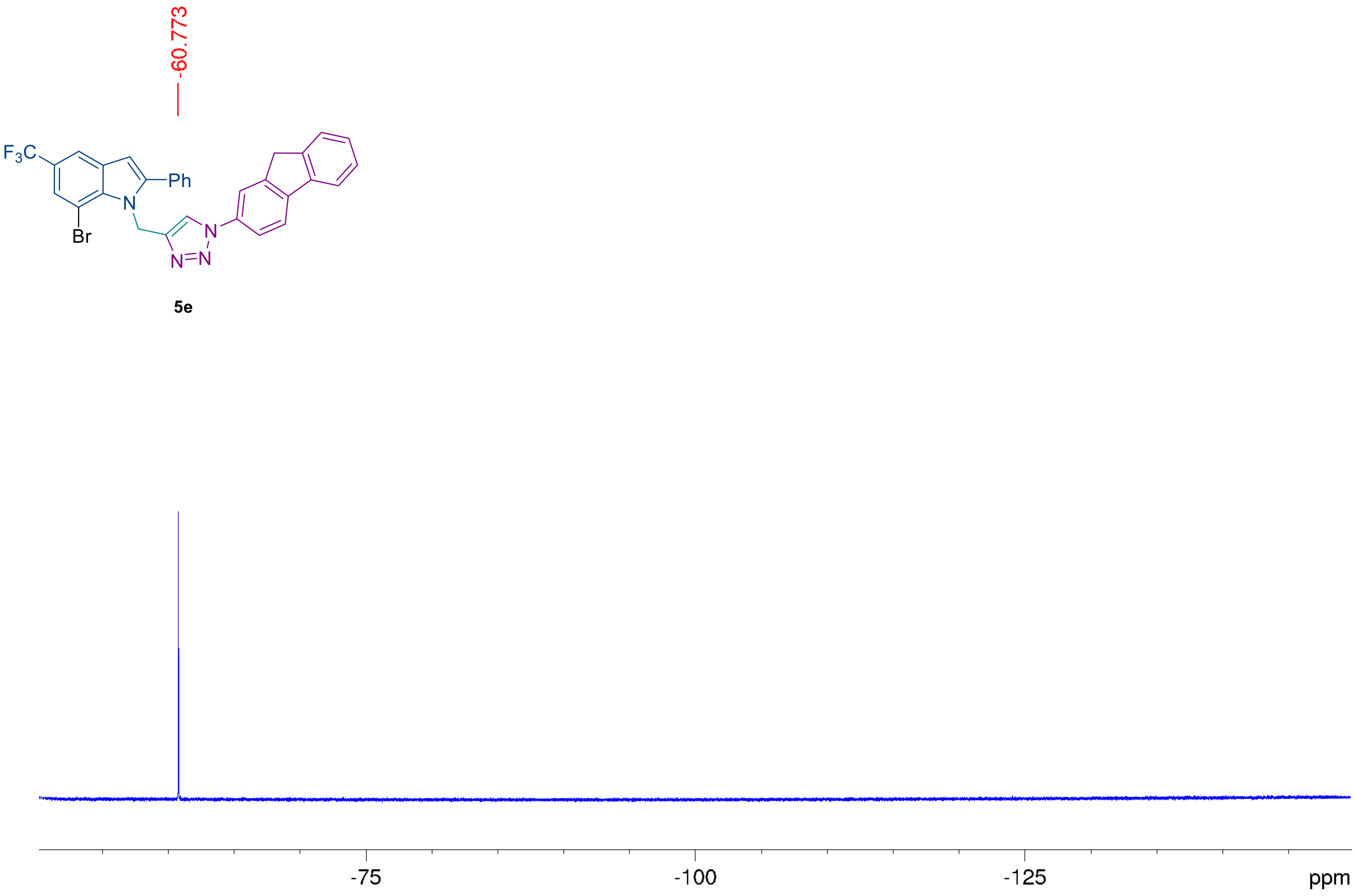

^{1}H NMR-spectrum (400 MHz, $CDCl_3$)

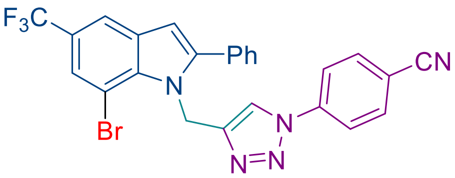

5f

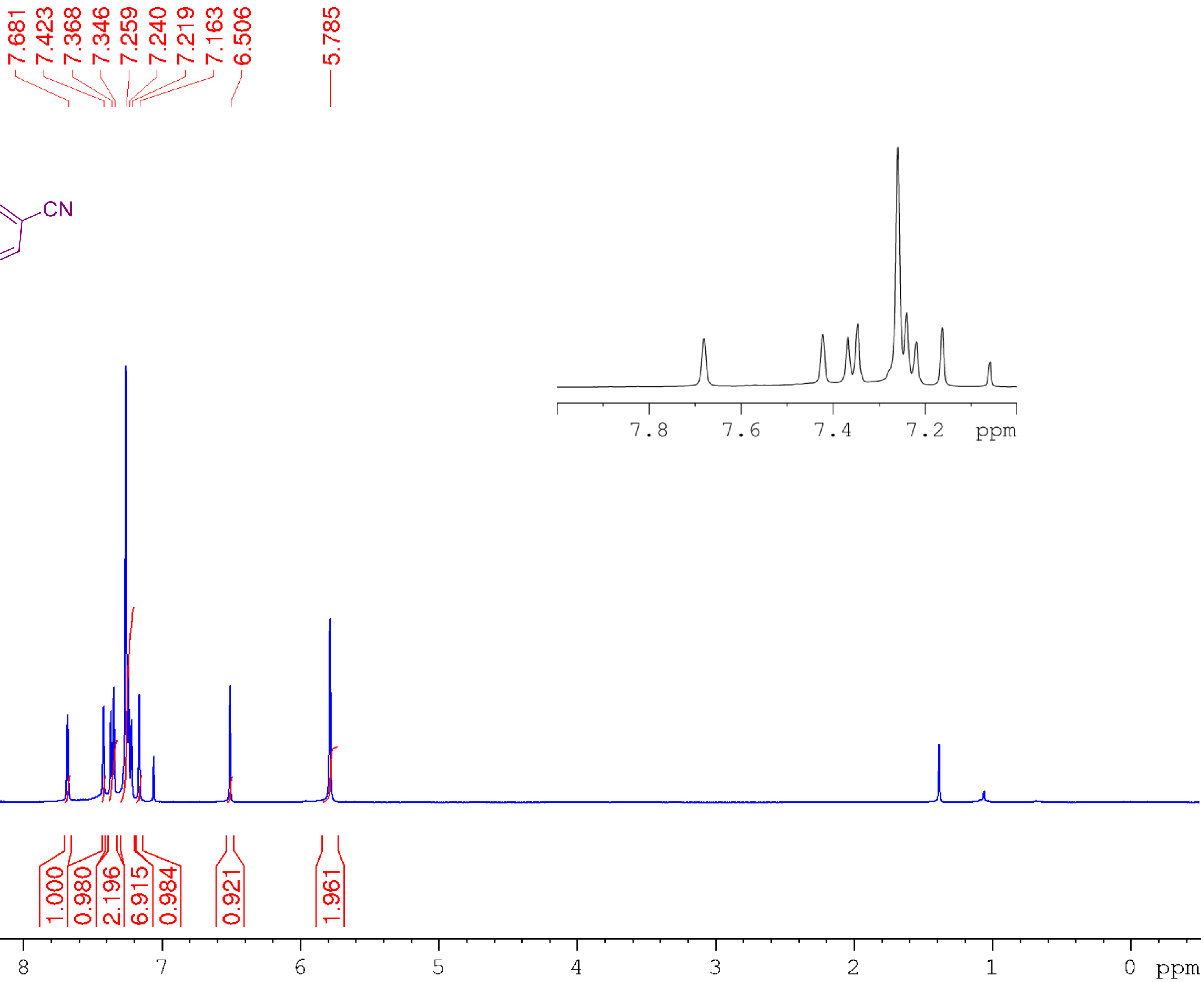

^{13}C NMR-spectrum (100 MHz, $CDCl_3$)

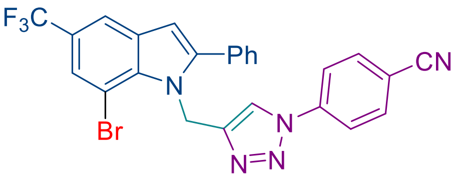

5f

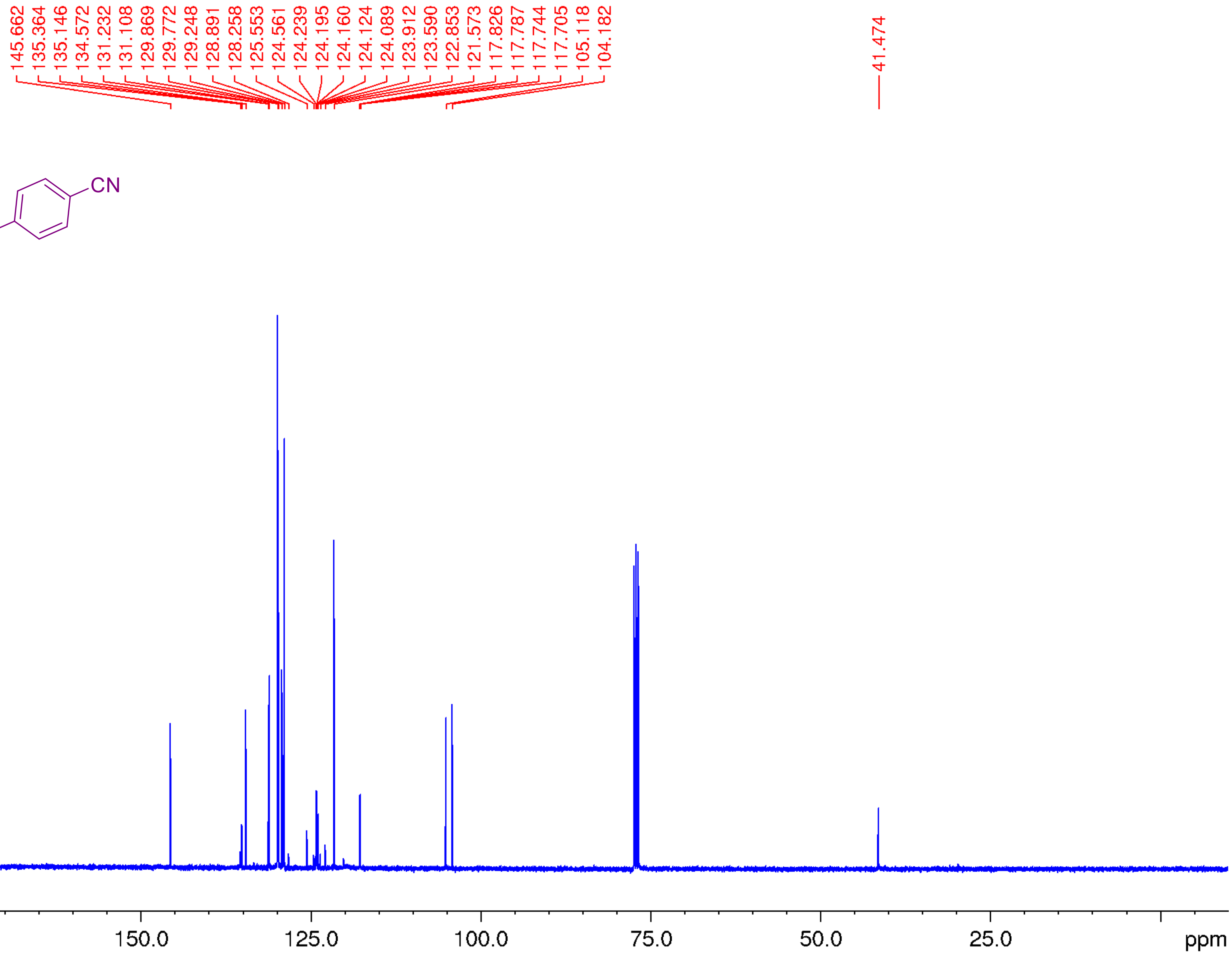

DEPT 135 NMR-spectrum ($CDCl_3$)

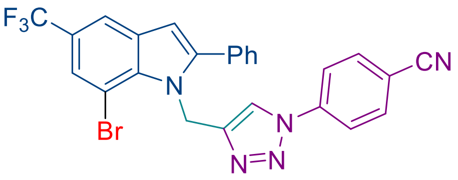

5f

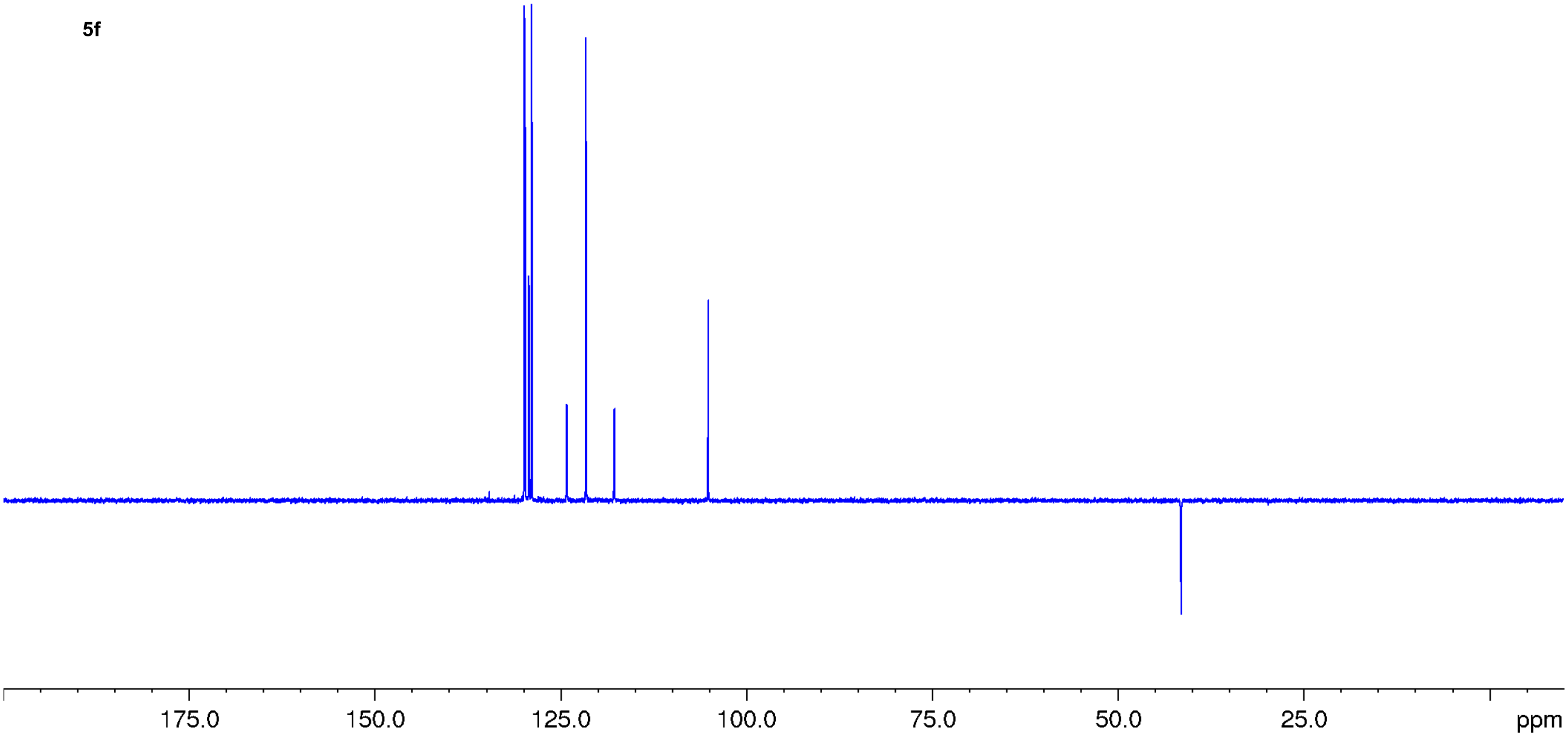

^{19}F NMR-spectrum (376.5 Hz, $CDCl_3$)

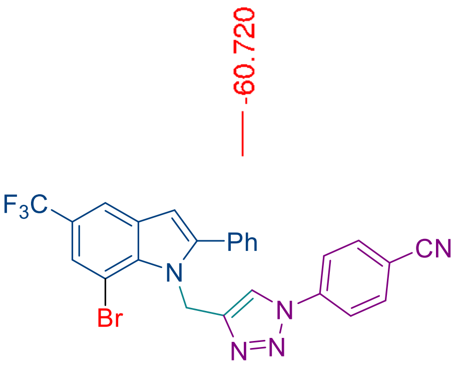

5f

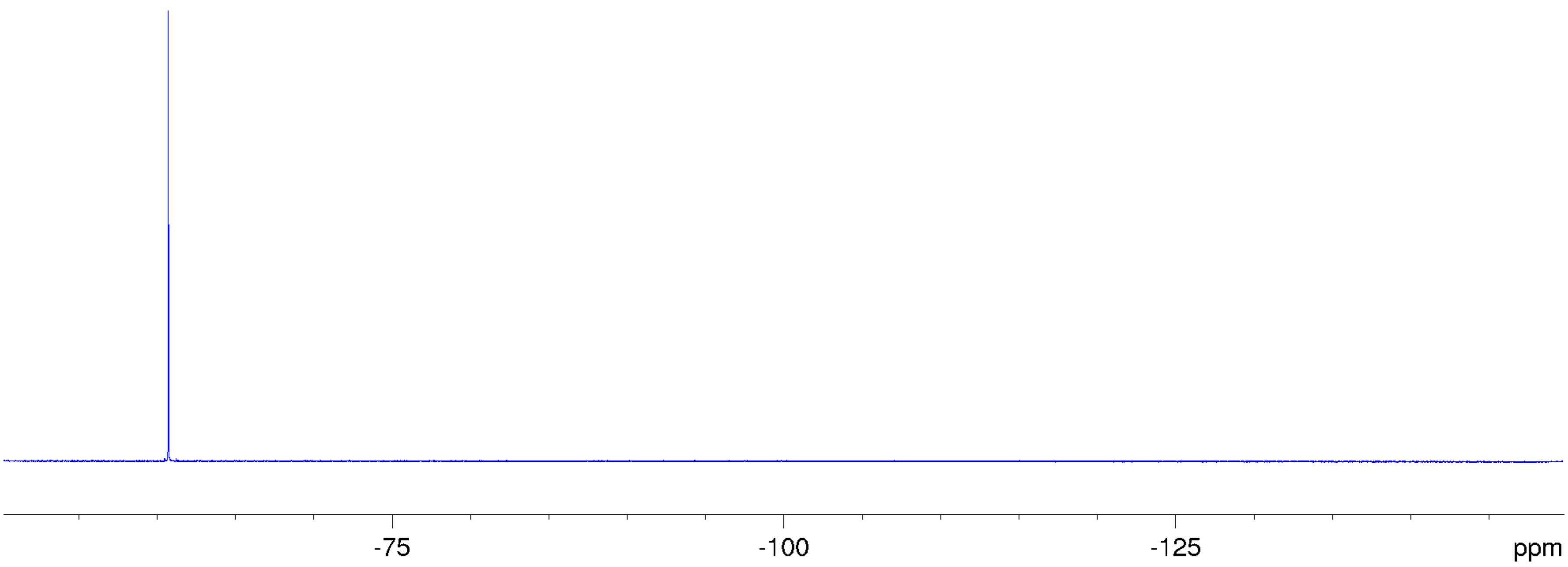

^{1}H NMR-spectrum (400 MHz, $CDCl_3$)

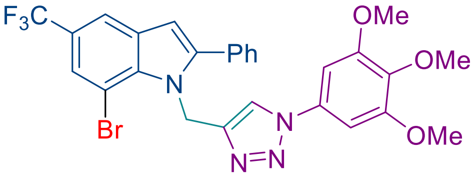

5g

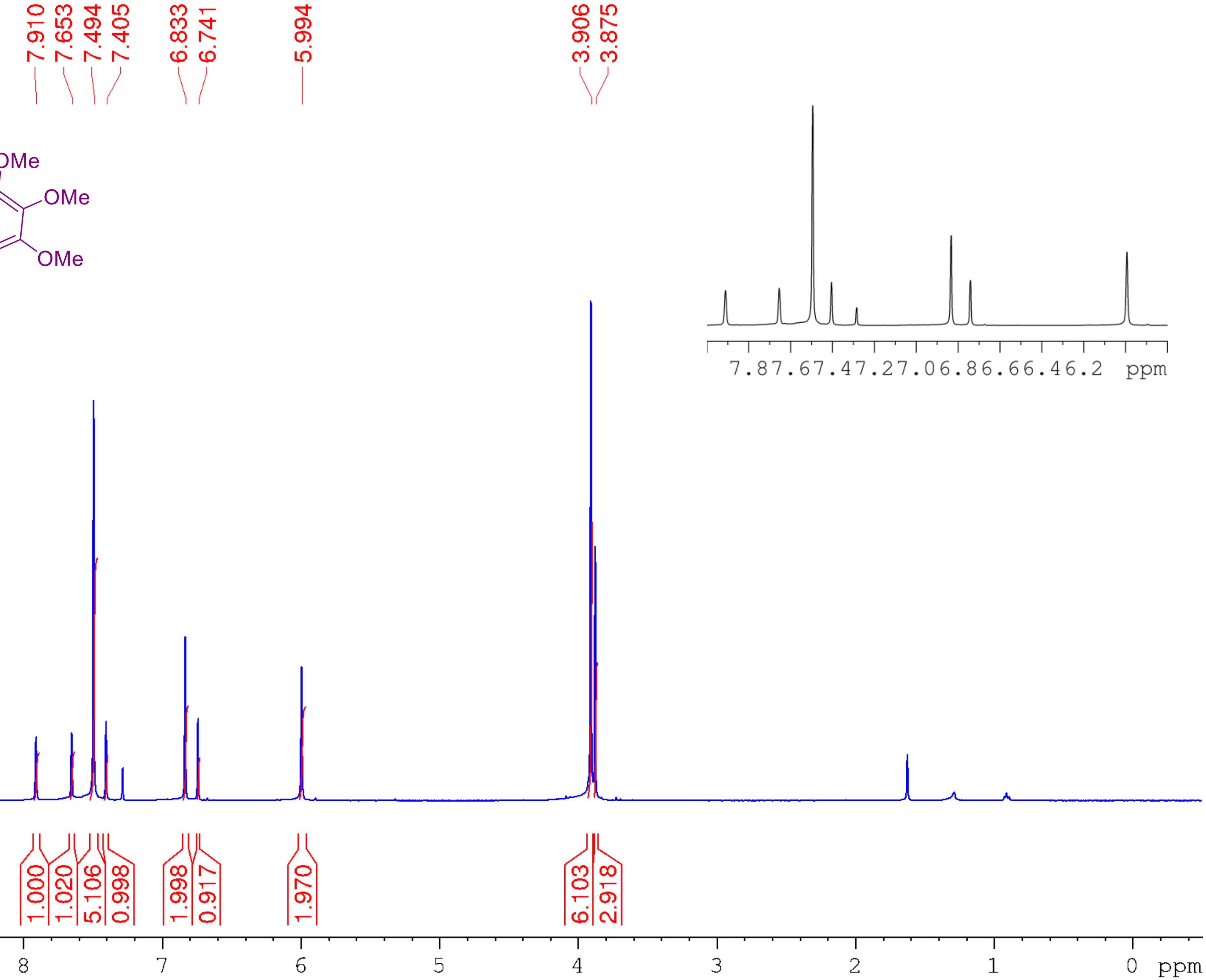

^{13}C NMR-spectrum (100 MHz, $CDCl_3$)

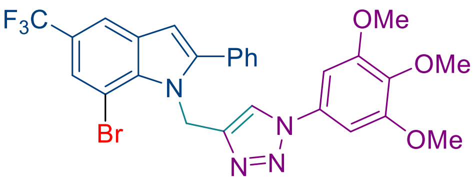

5g

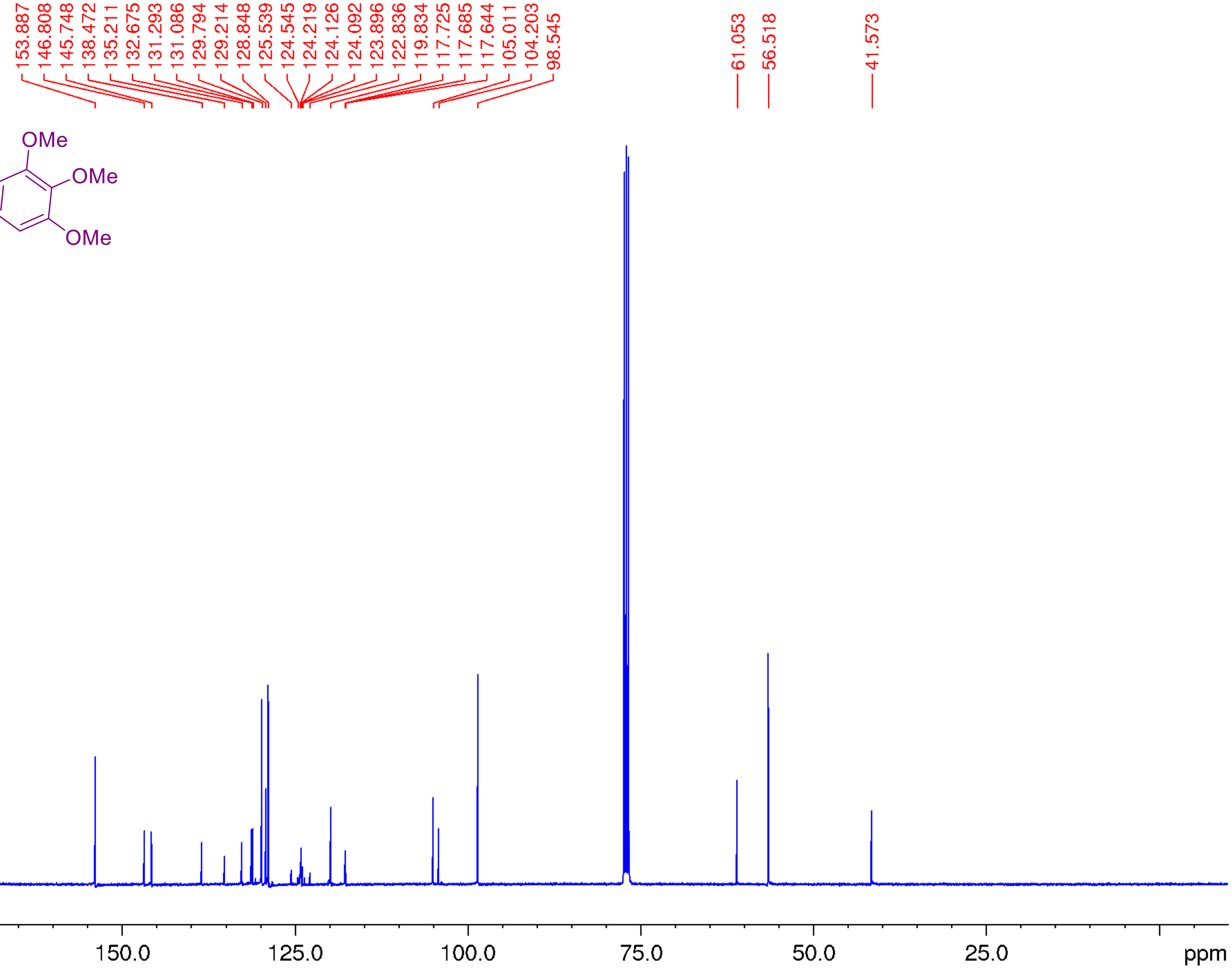

DEPT 135 NMR-spectrum ($CDCl_3$)

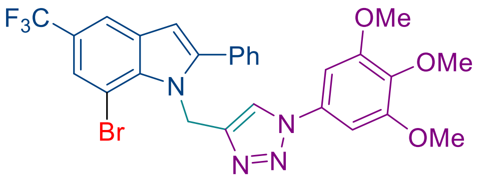

5g

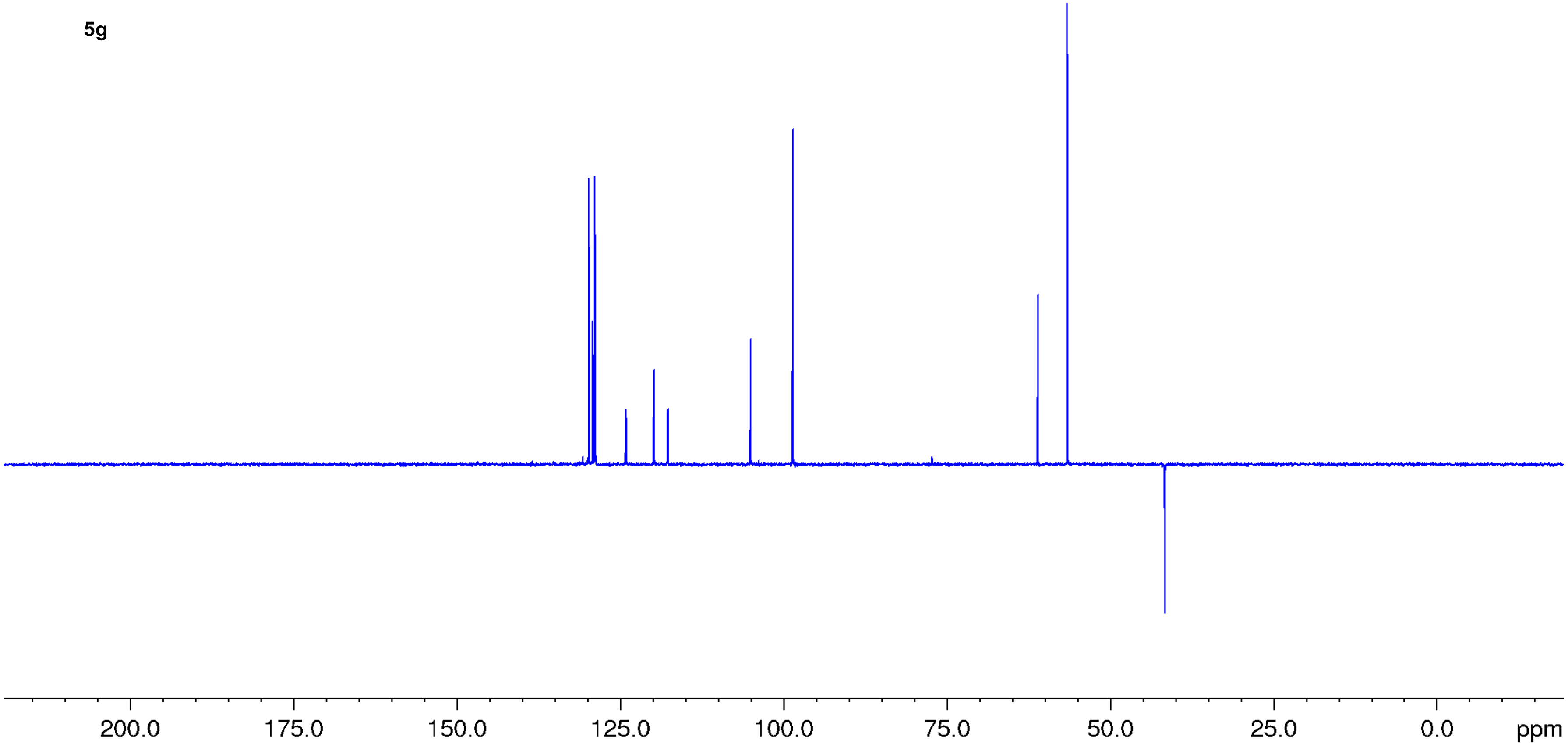

^{19}F NMR-spectrum (376.5 Hz, $CDCl_3$)

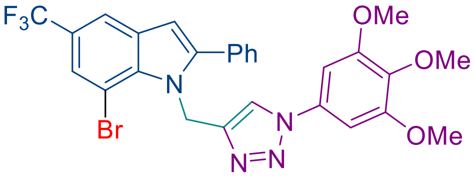

5g

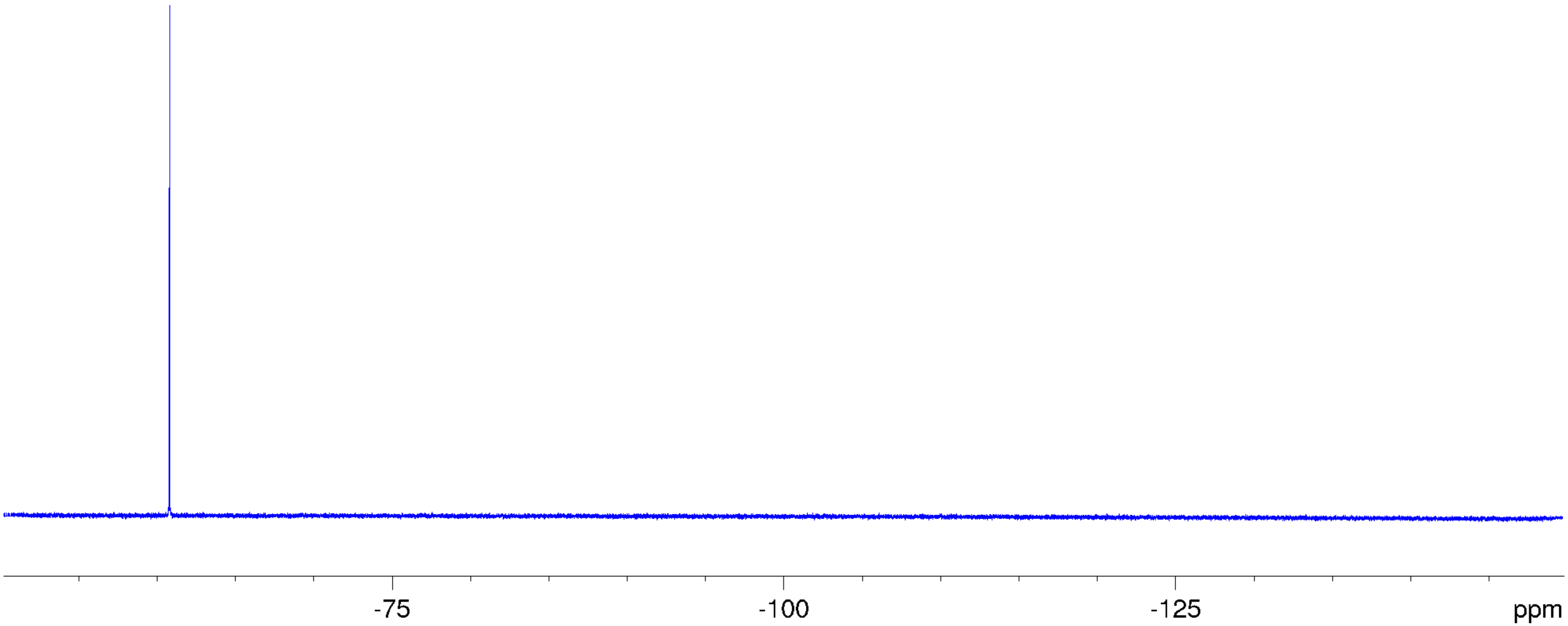

^{1}H NMR-spectrum (400 MHz, $CDCl_3$)

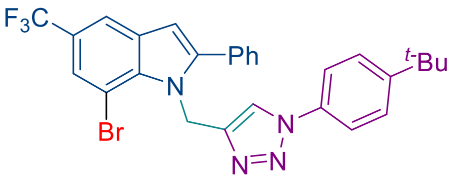

5h

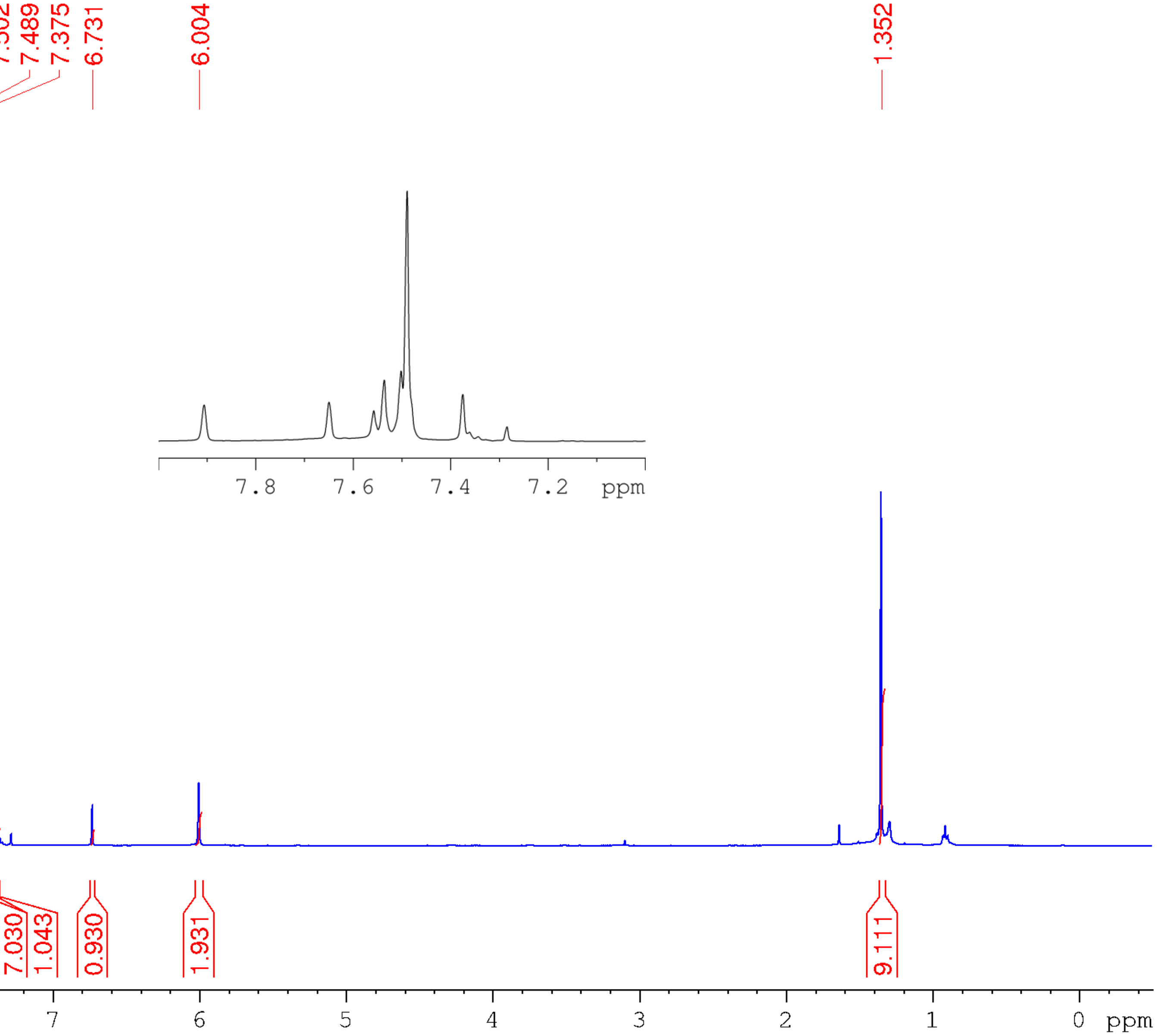

^{13}C NMR-spectrum (100 MHz, $CDCl_3$)

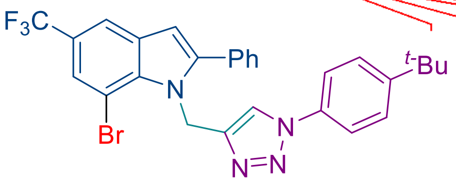

5h

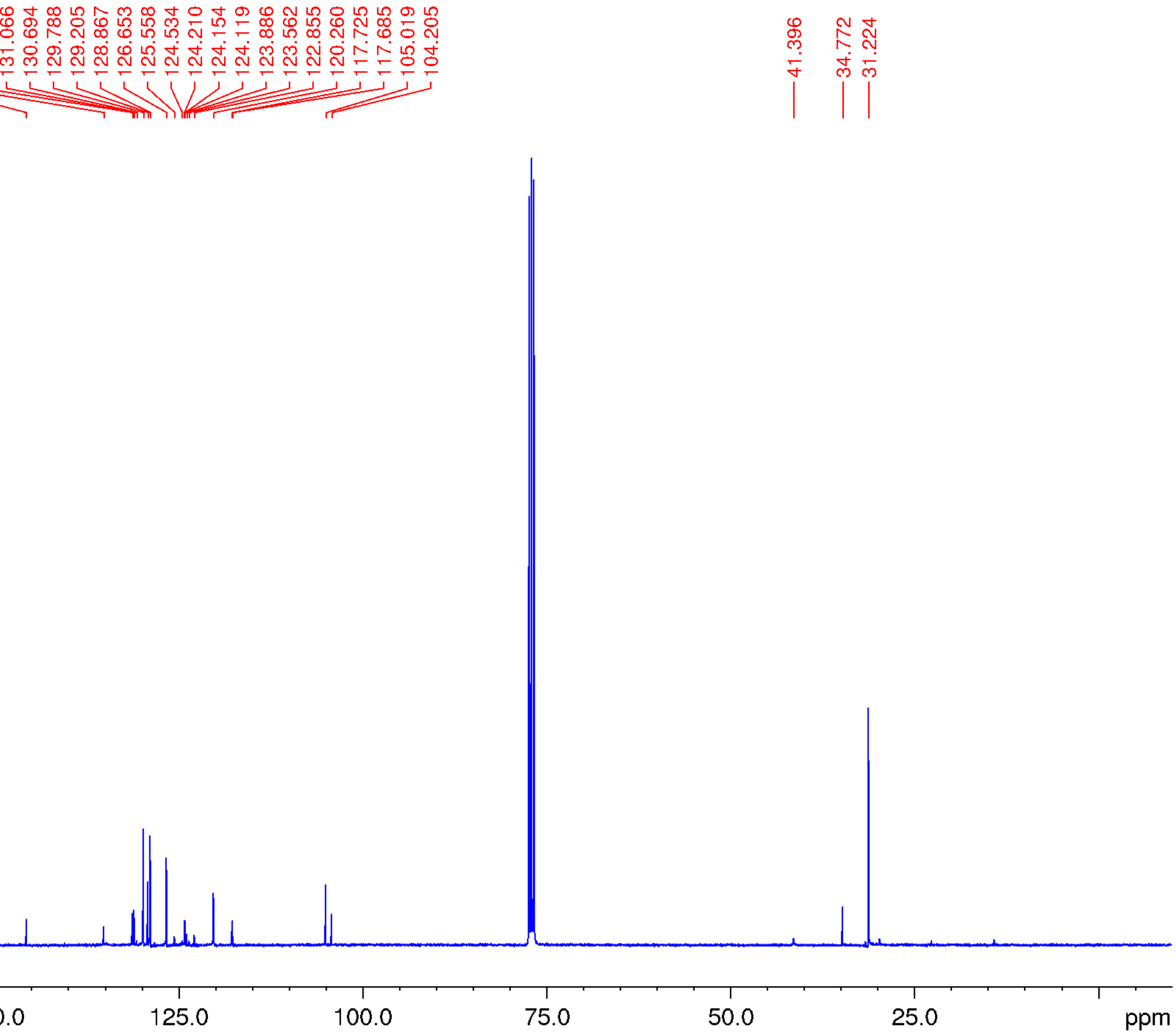

DEPT 135 NMR-spectrum ($CDCl_3$)

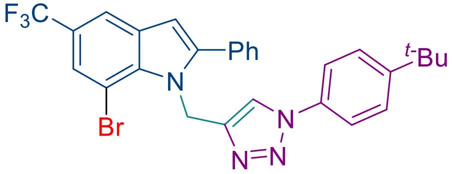

5h

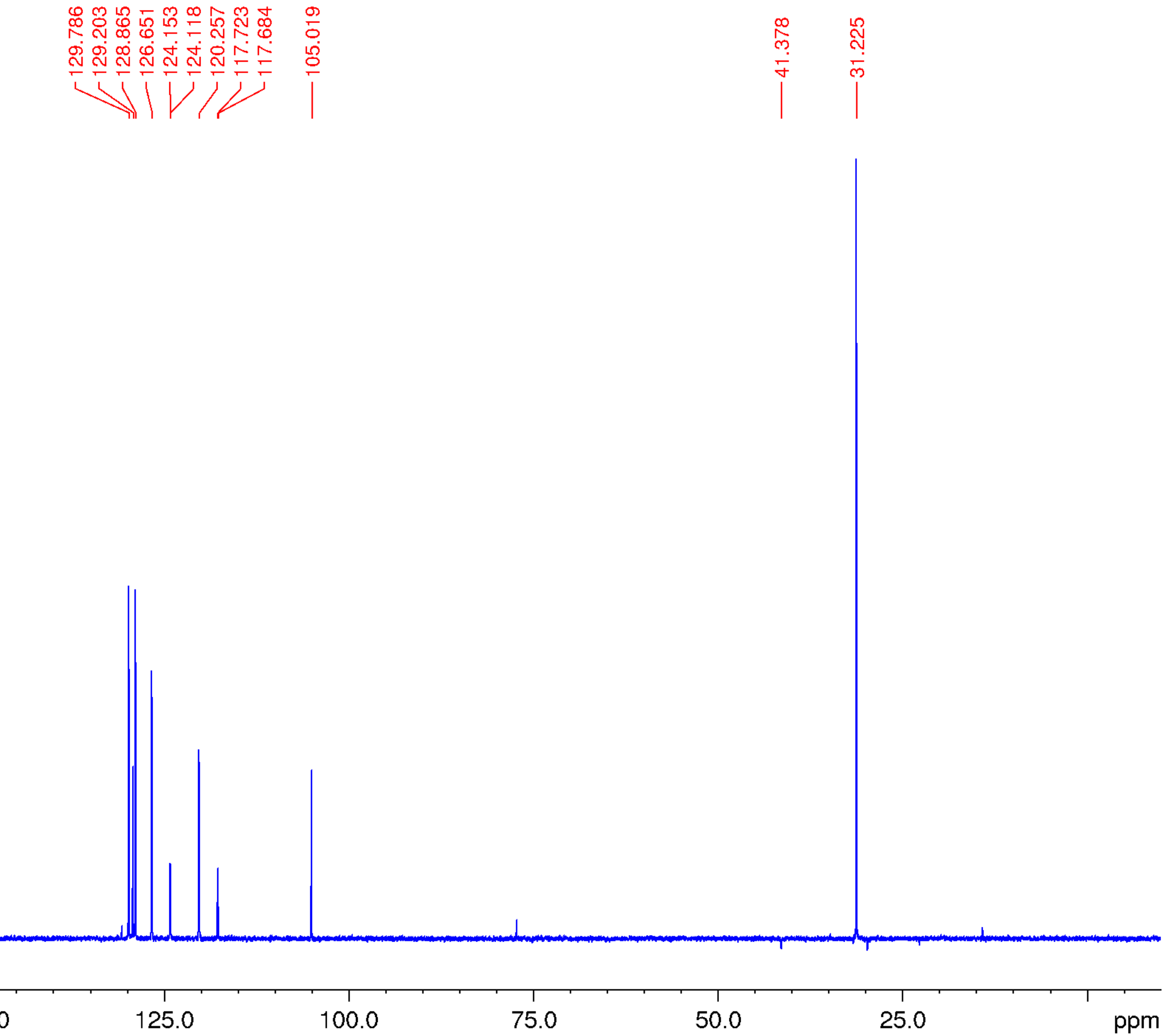

^{19}F NMR-spectrum (376.5 Hz, $CDCl_3$)

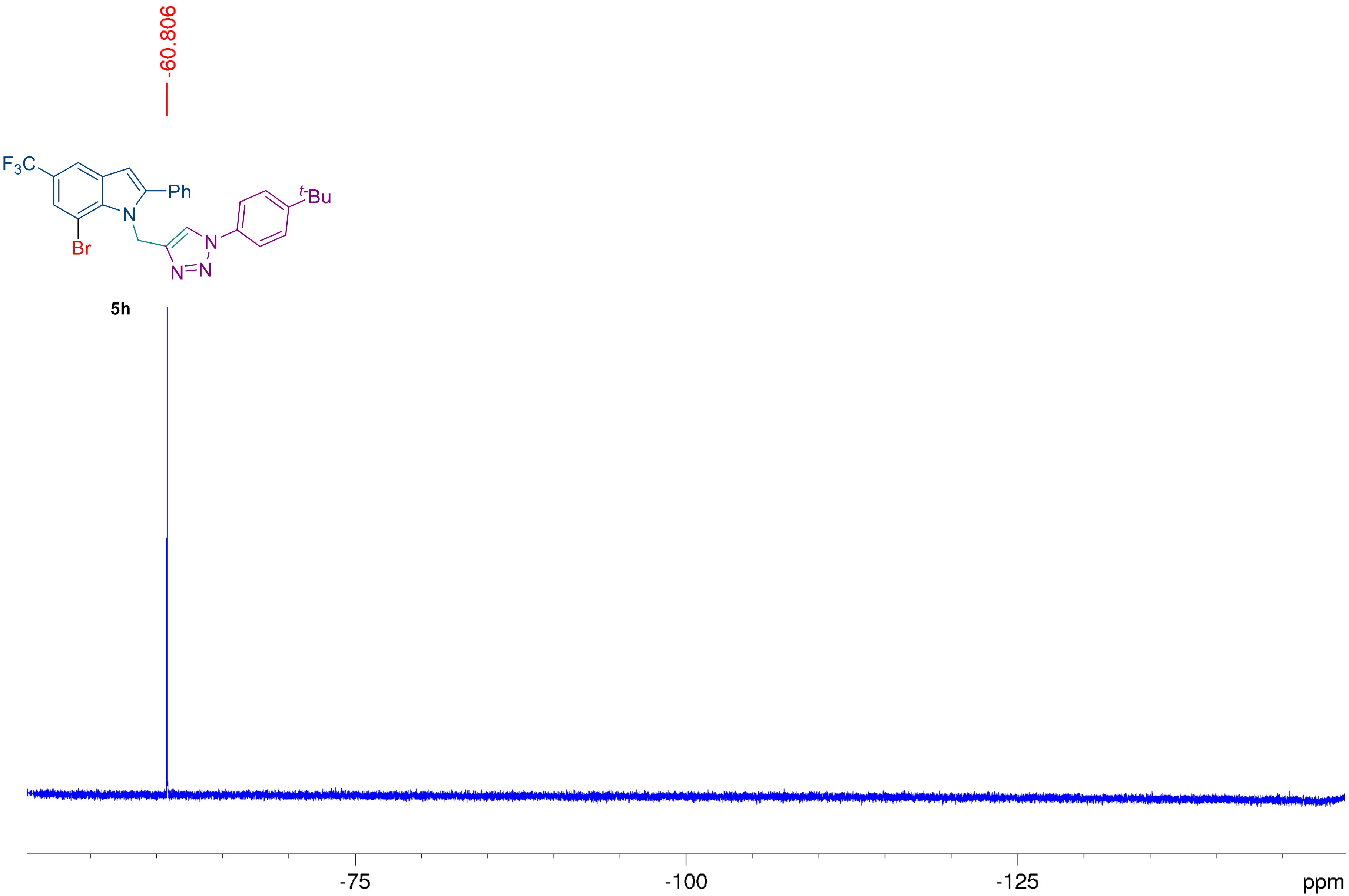

^{1}H NMR-spectrum (400 MHz, $CDCl_3$)

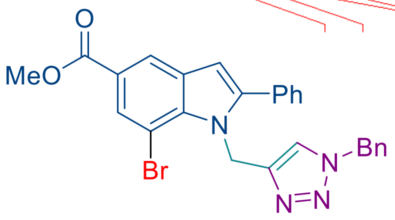

5i

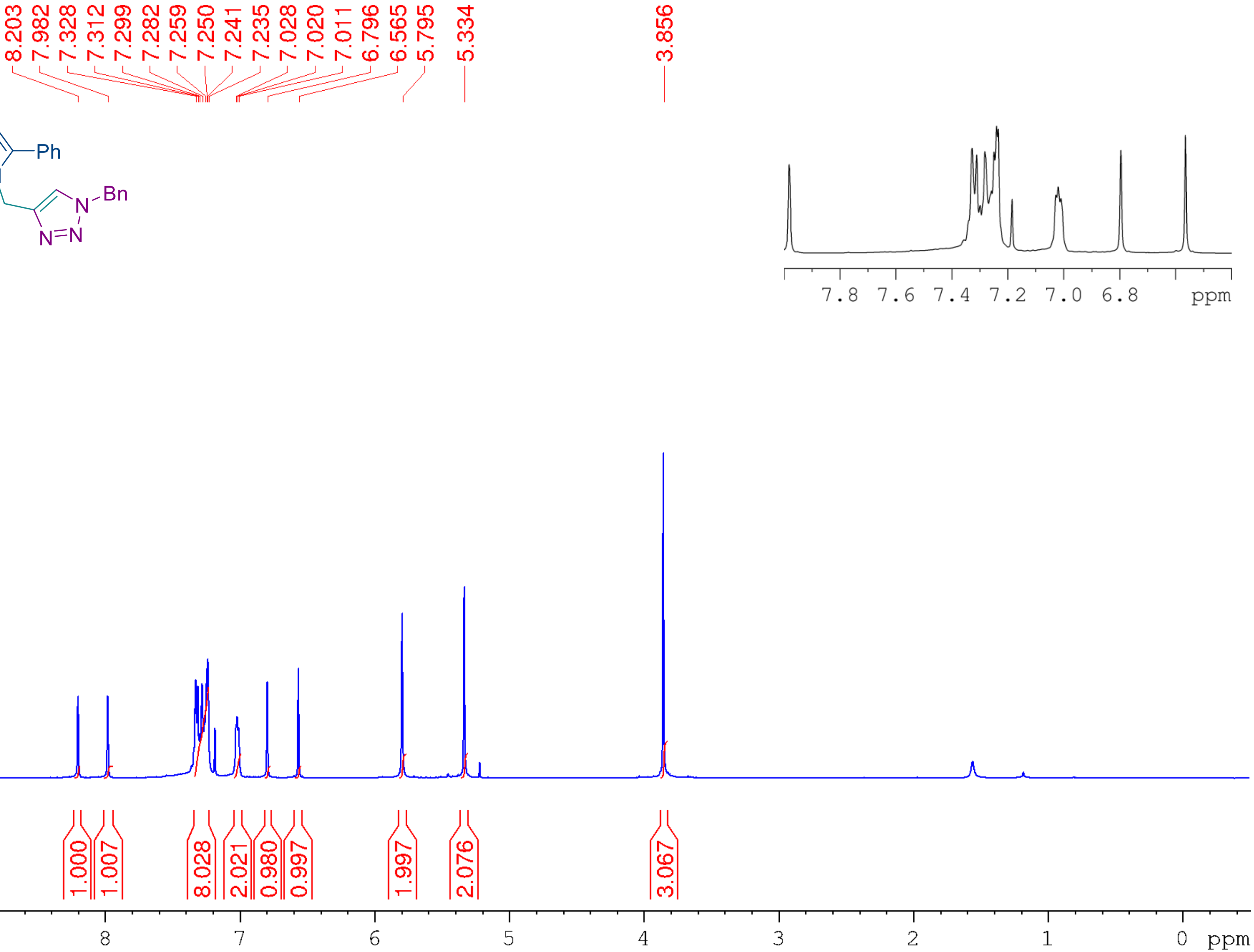

^{13}C NMR-spectrum (100 MHz, $CDCl_3$)

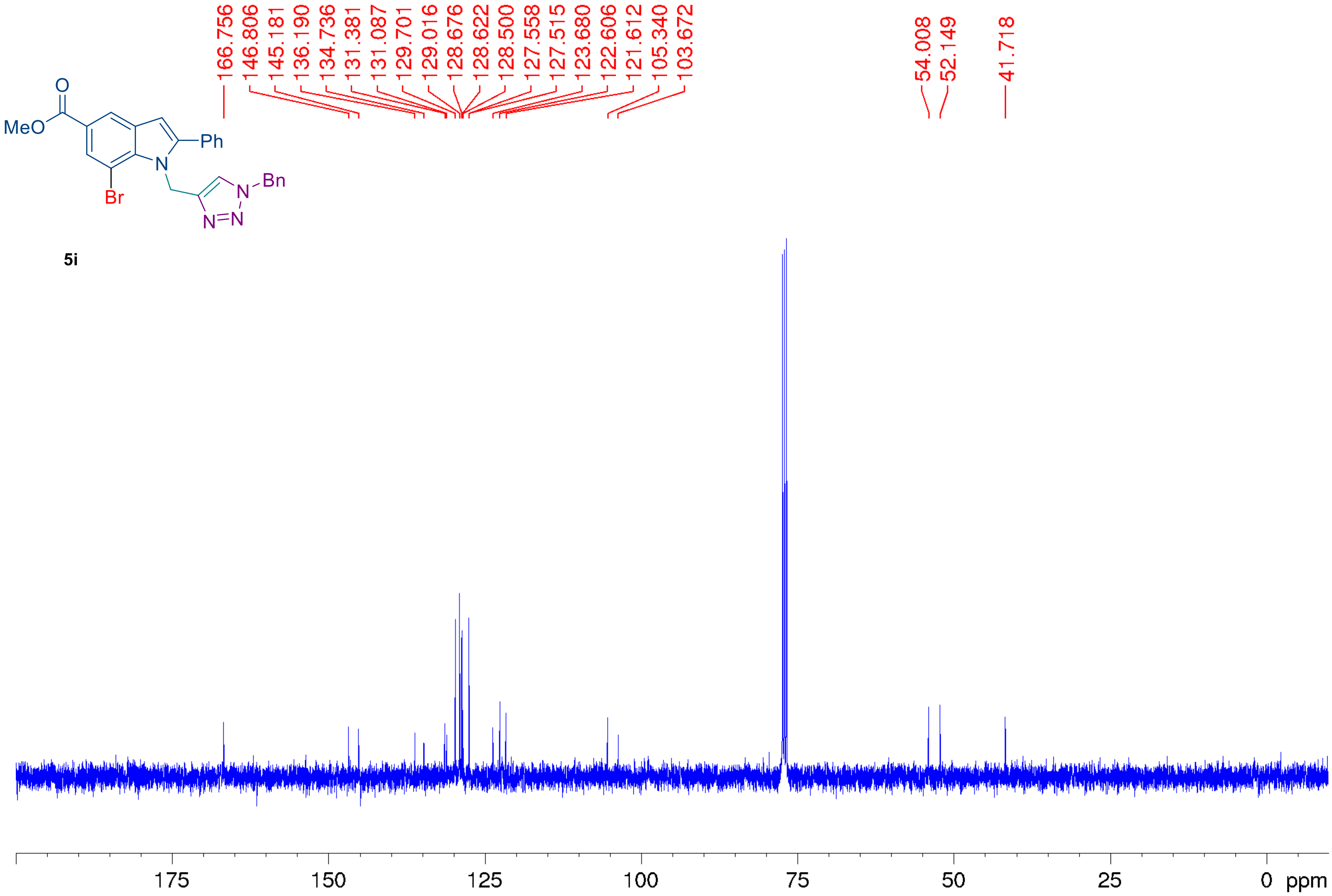

5i

DEPT 135 NMR-spectrum ($CDCl_3$)

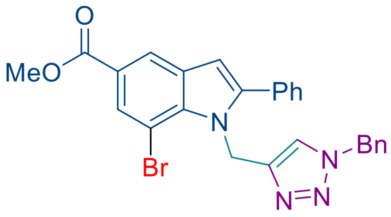

5i

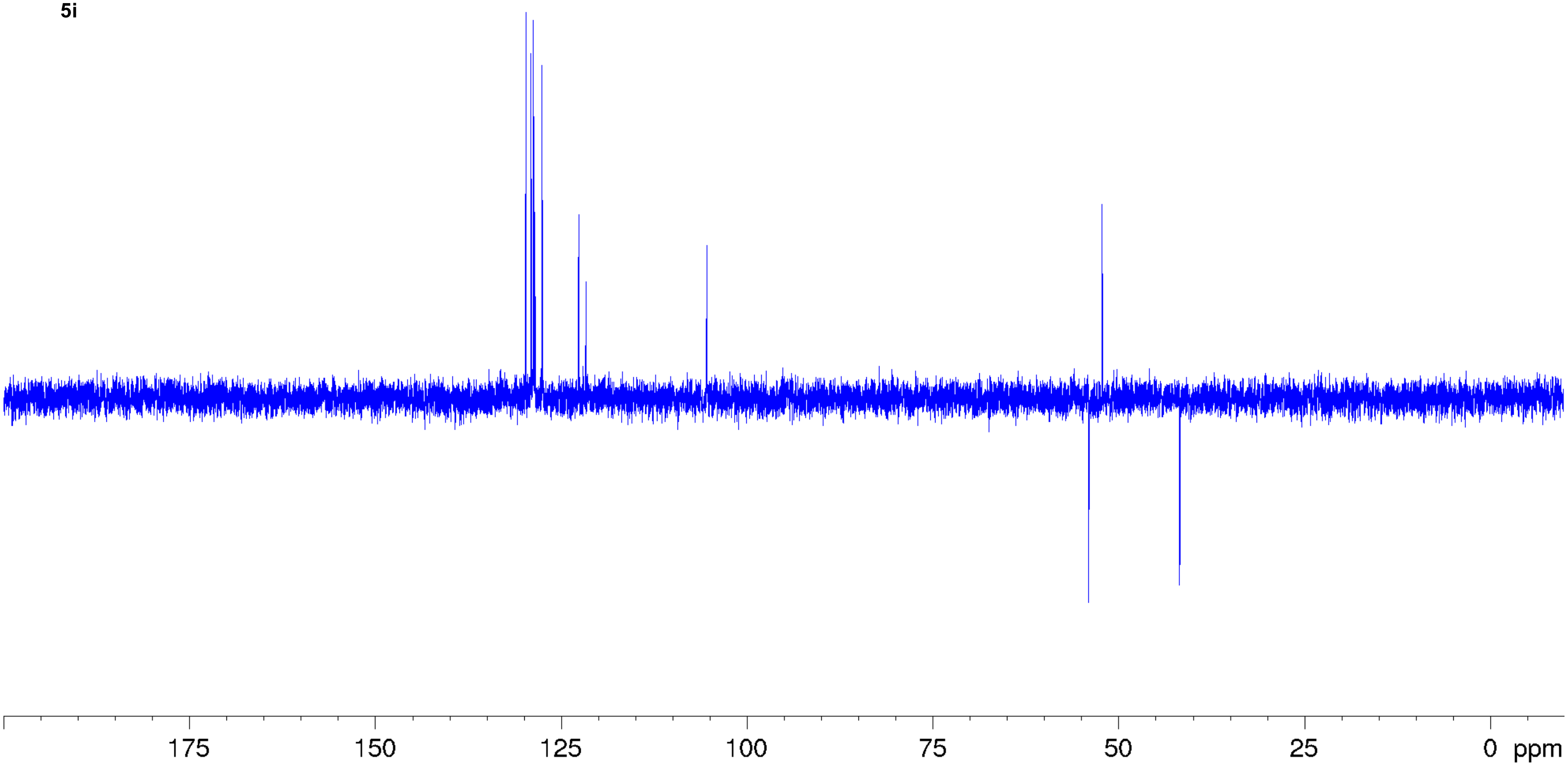

^{1}H NMR-spectrum (400 MHz, $CDCl_3$)

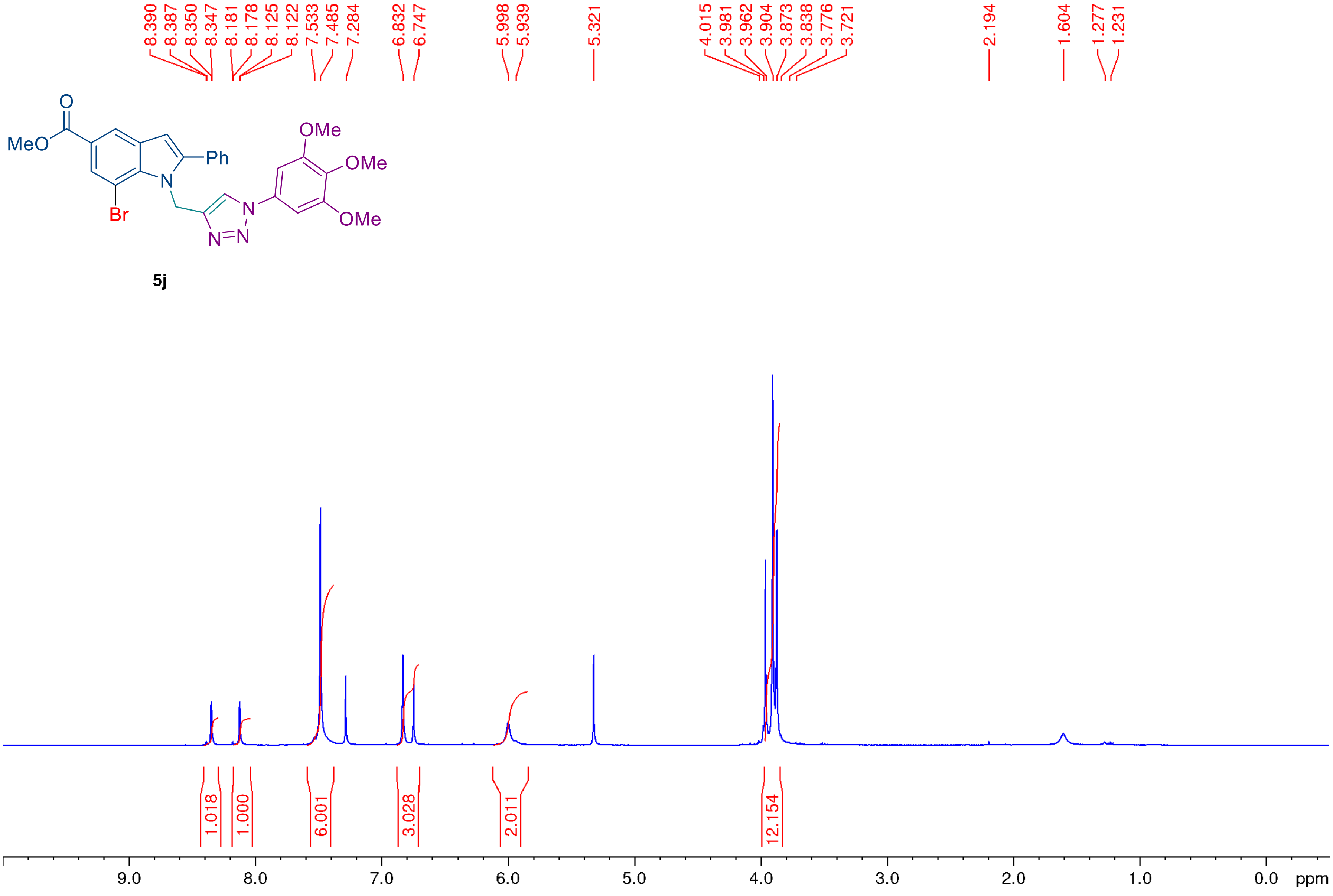

5j

^{13}C NMR-spectrum (100 MHz, $CDCl_3$)

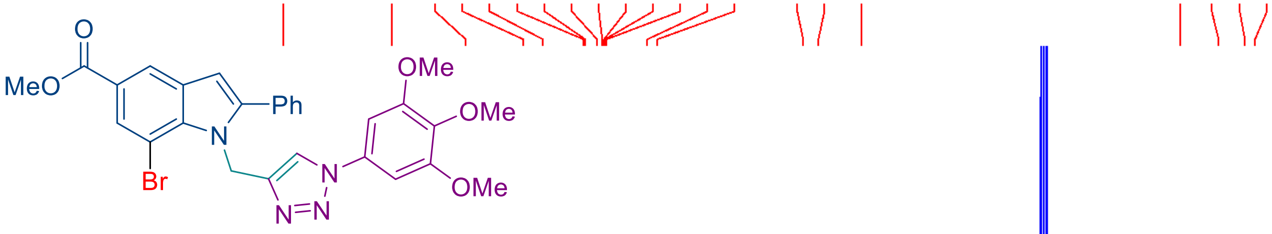

5j

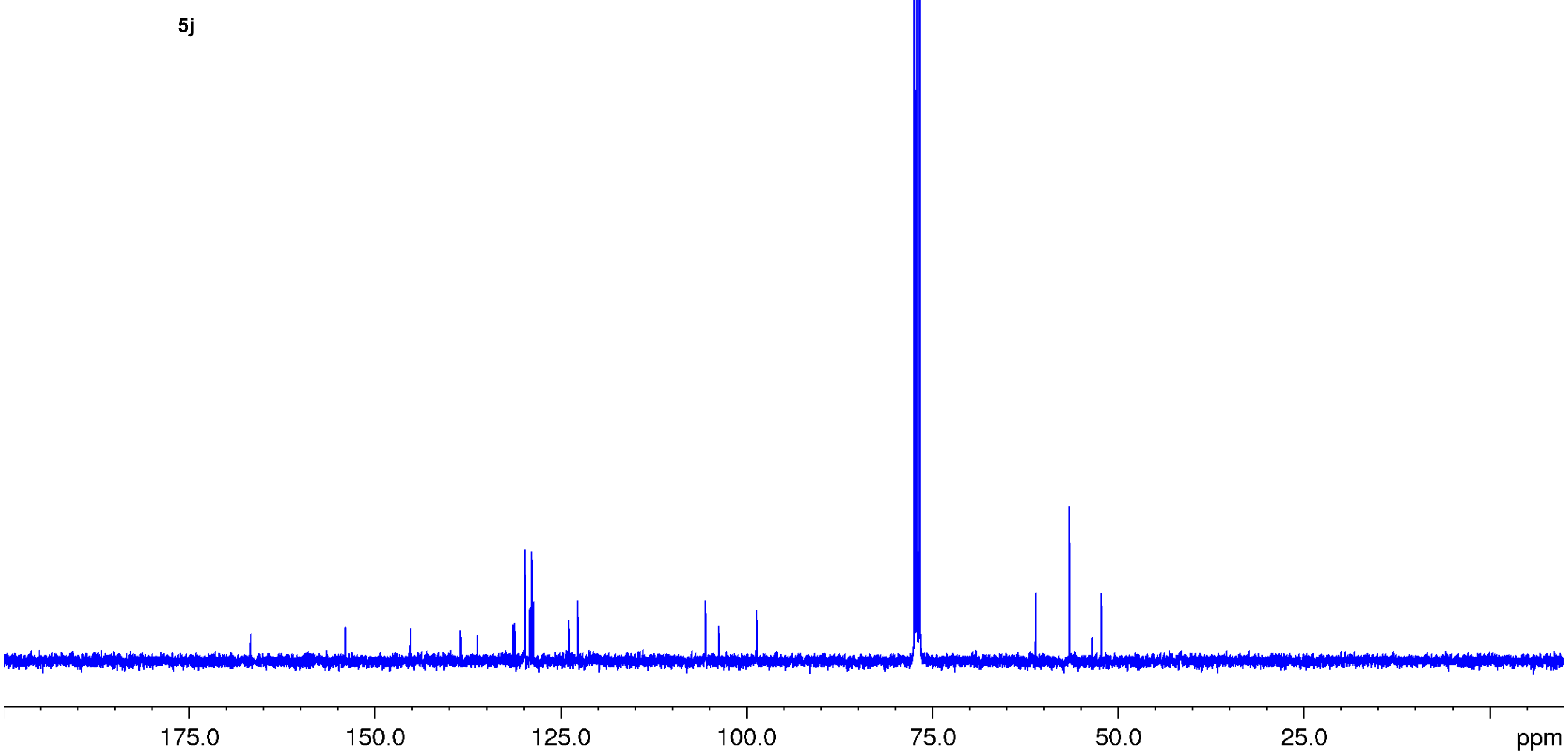

DEPT 135 NMR-spectrum ($CDCl_3$)

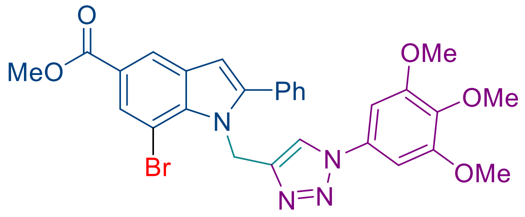

5j

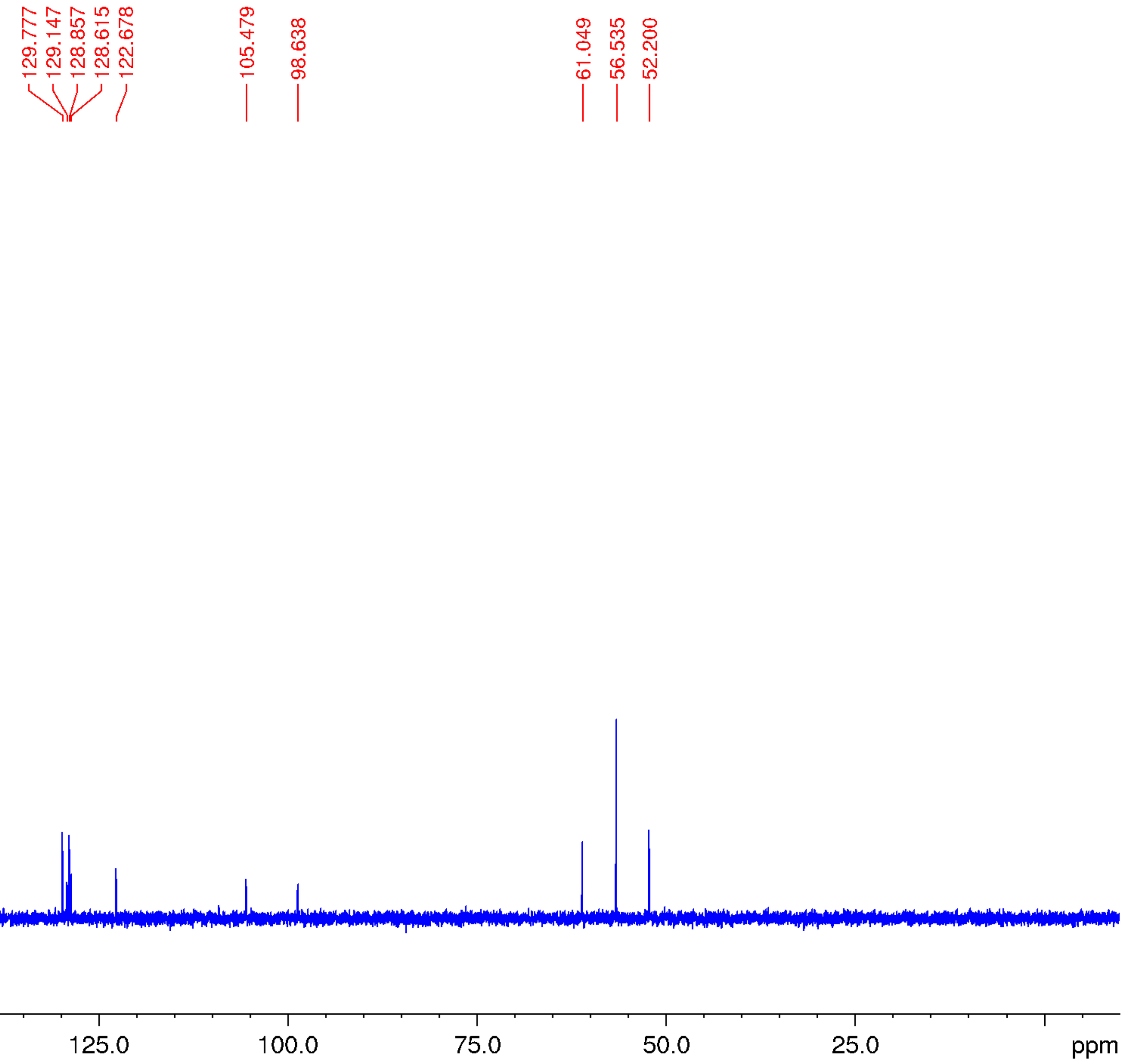

^{1}H NMR-spectrum (400 MHz, $CDCl_3$)

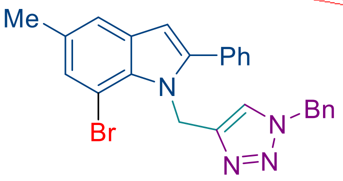

5k

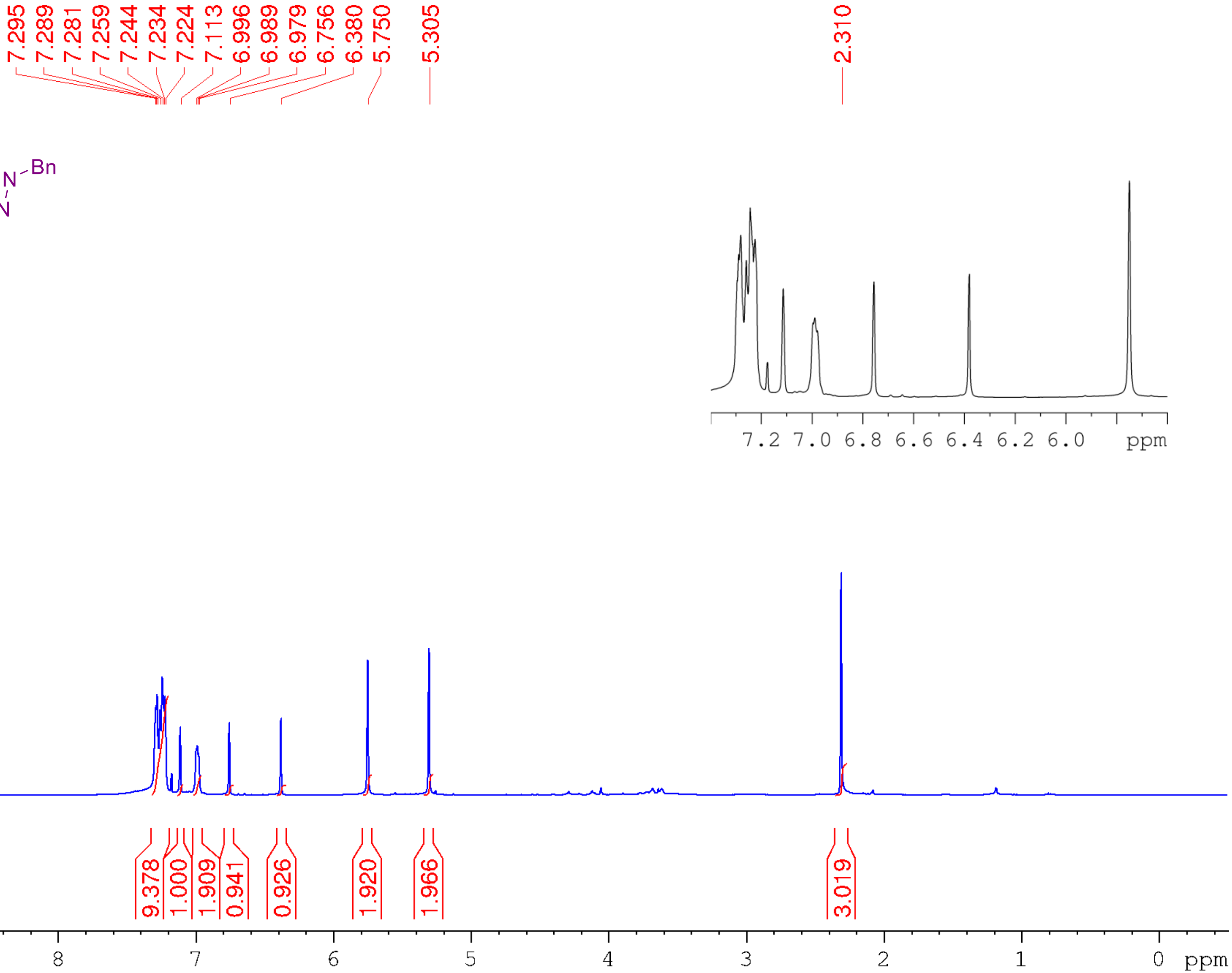

^{13}C NMR-spectrum (100 MHz, $CDCl_3$)

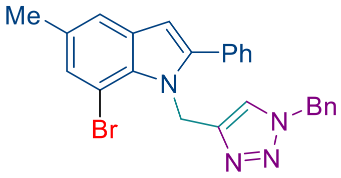

5k

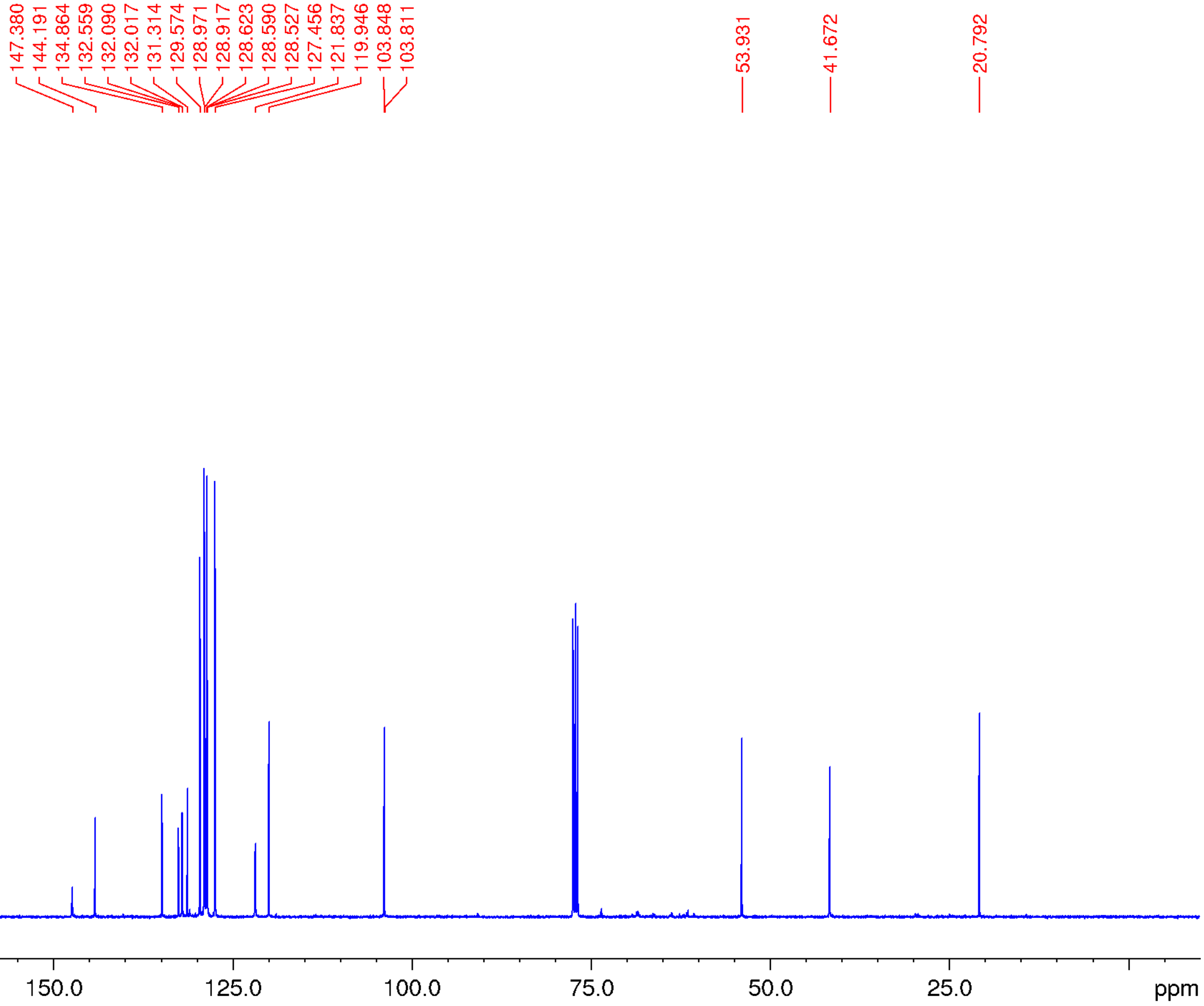

DEPT 135 NMR-spectrum ($CDCl_3$)

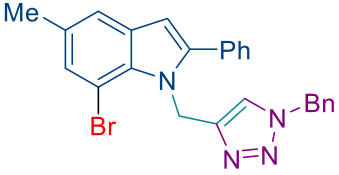

5k

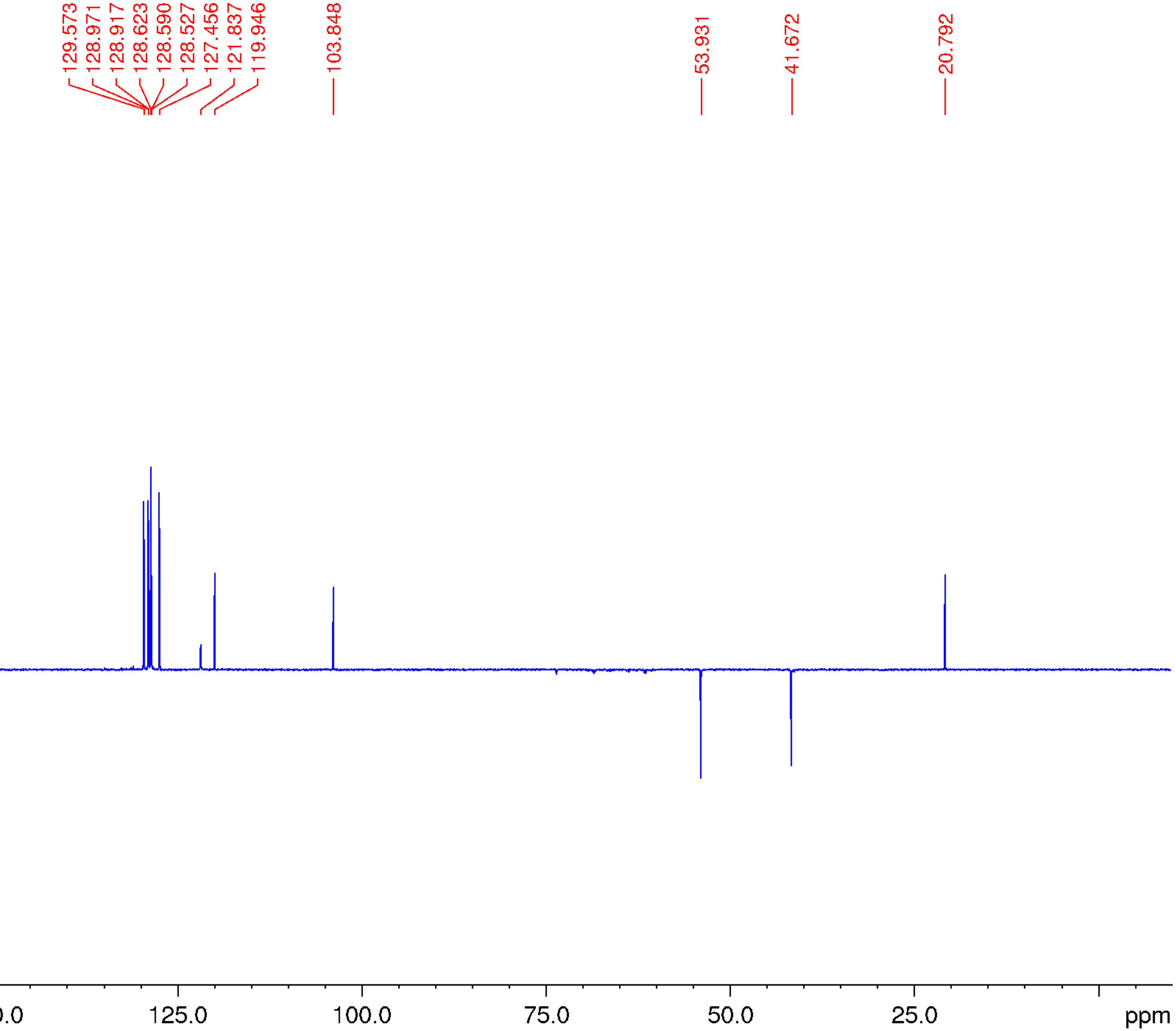

^{1}H NMR-spectrum (400 MHz, $CDCl_3$)

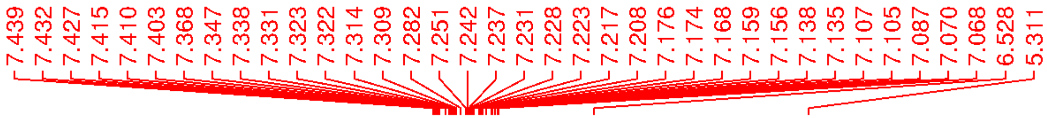

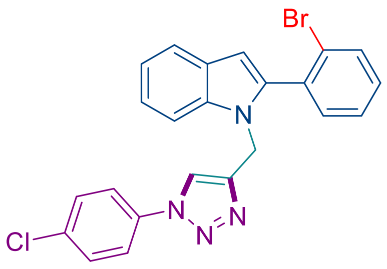

6a

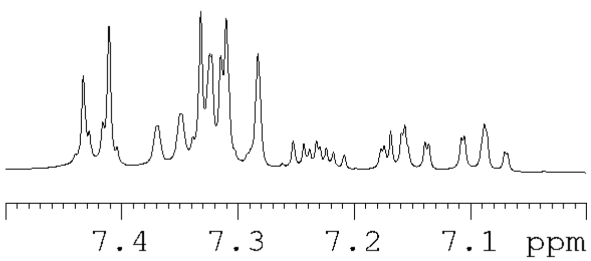

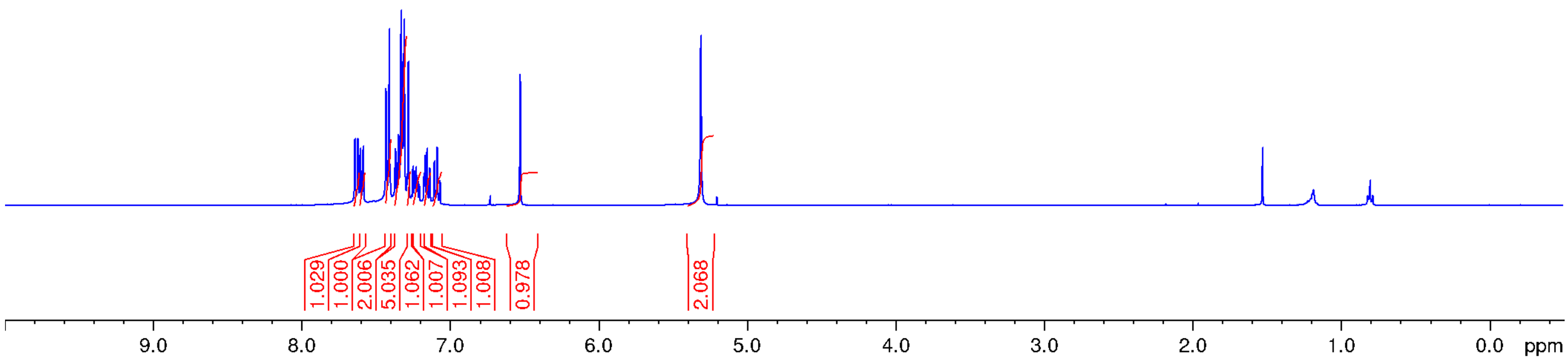

^{13}C NMR-spectrum (100 MHz, $CDCl_3$)

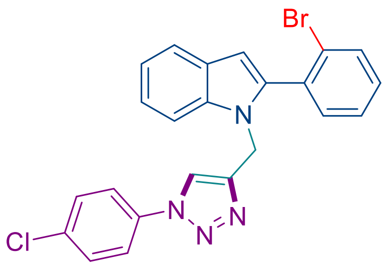

6a

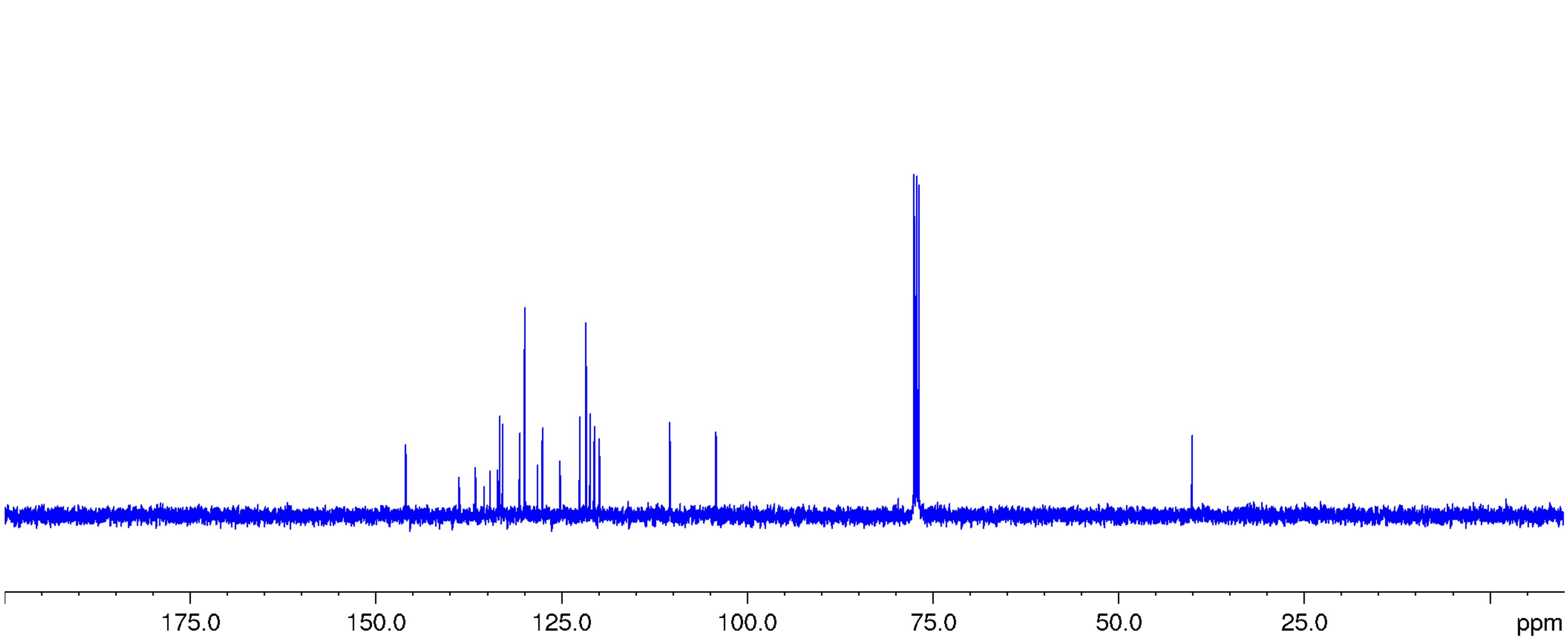

DEPT 135 NMR-spectrum ($CDCl_3$)

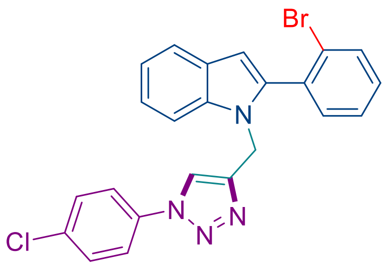

6a

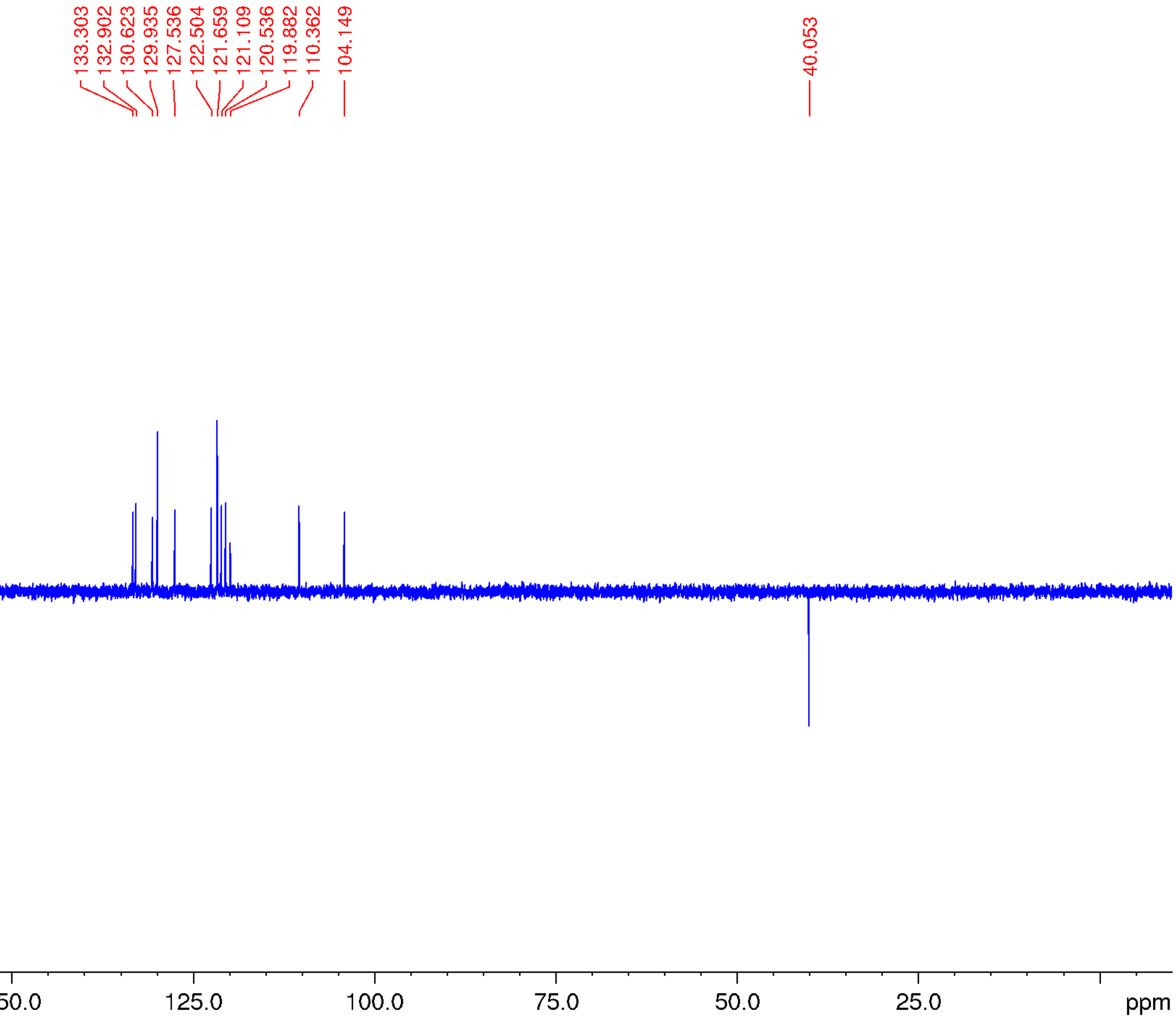

^{1}H NMR-spectrum (400 MHz, $CDCl_3$)

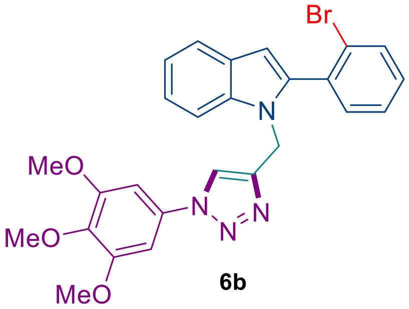

6b

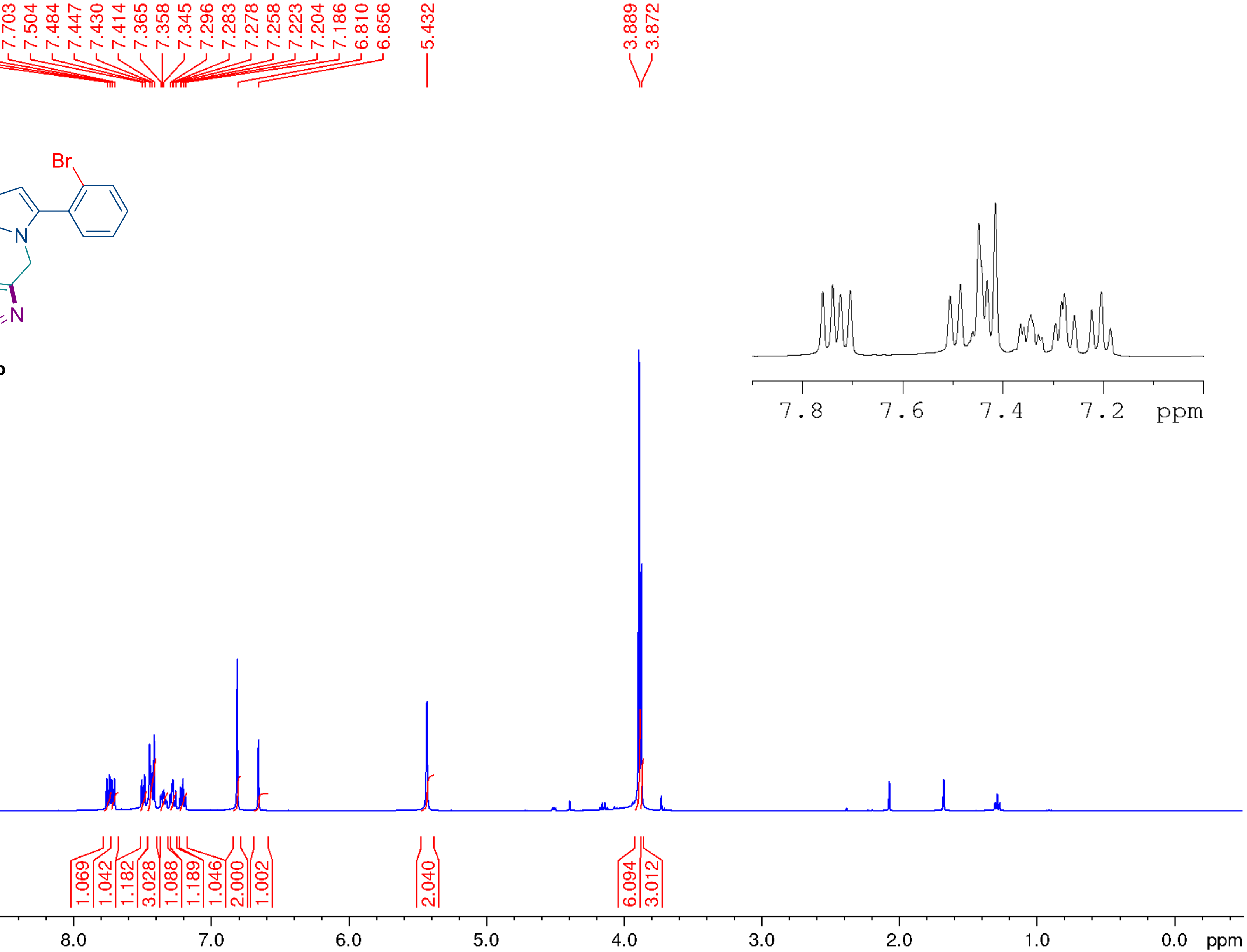

^{13}C NMR-spectrum (100 MHz, $CDCl_3$)

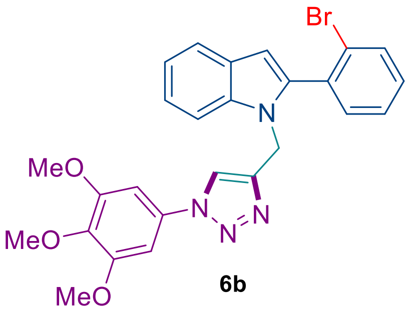

6b

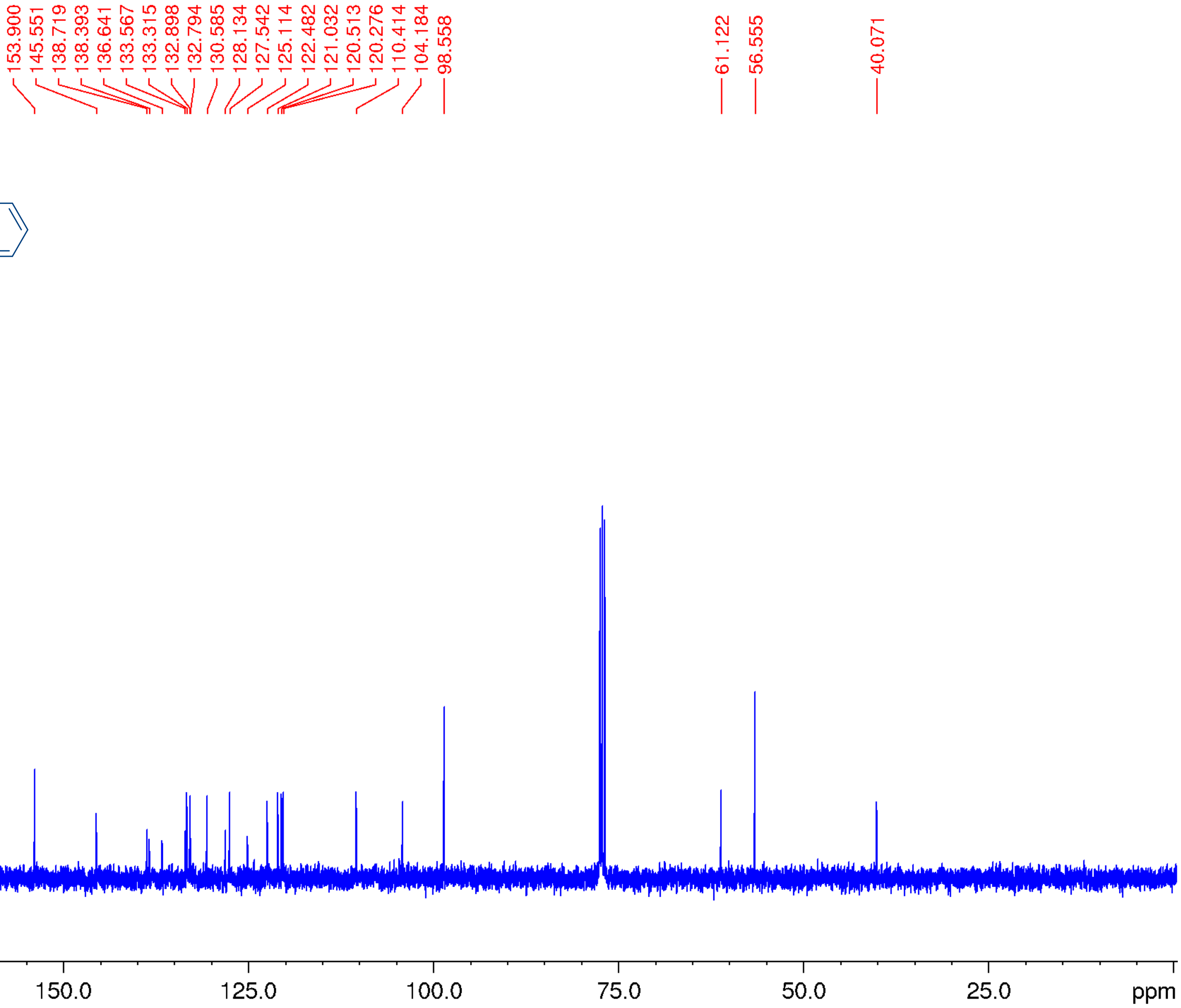

DEPT 135 NMR-spectrum ($CDCl_3$)

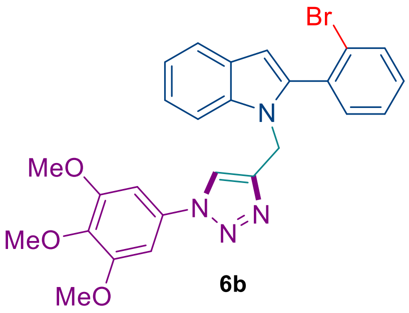

6b

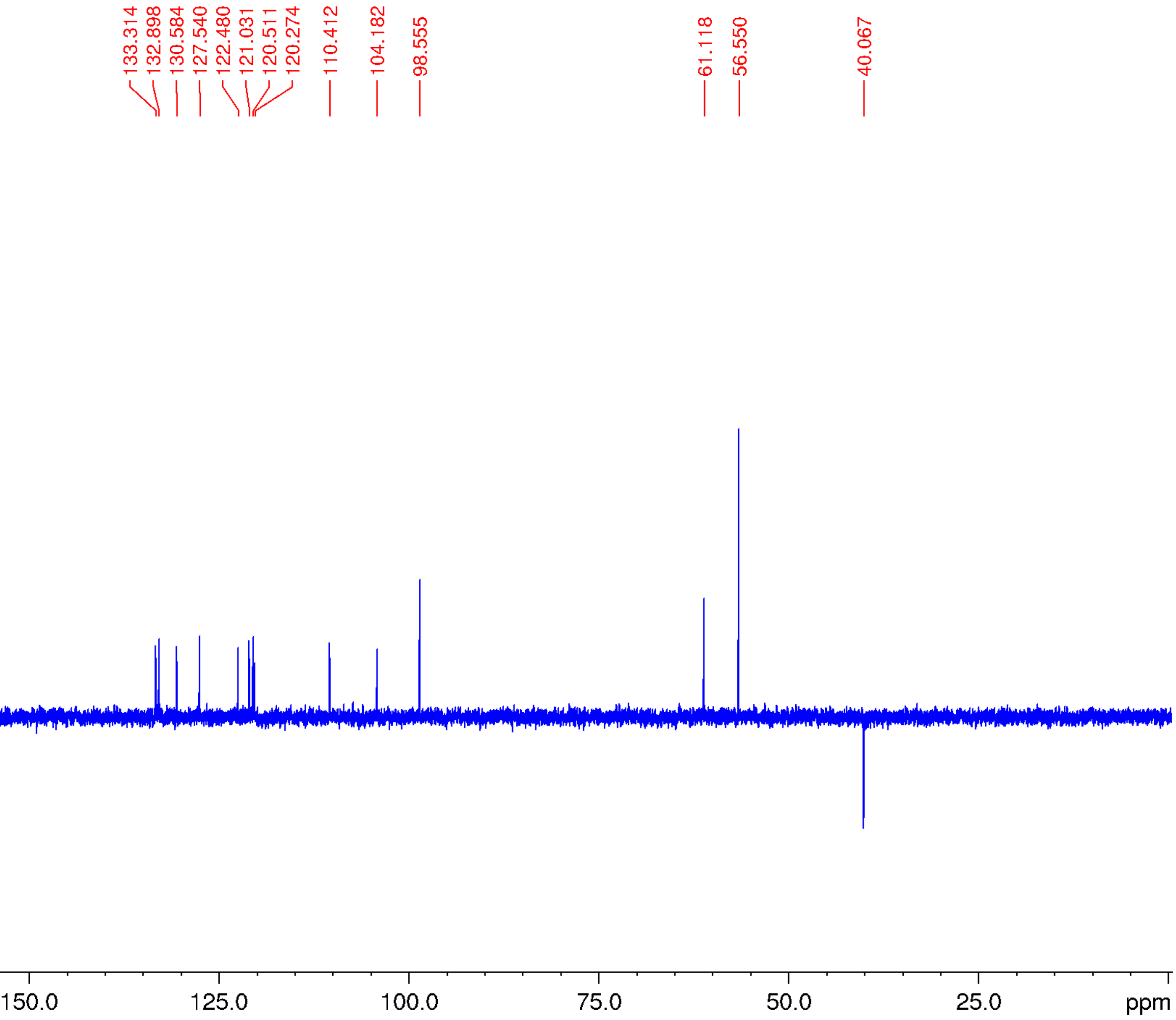

^{1}H NMR-spectrum (400 MHz, $CDCl_3$)

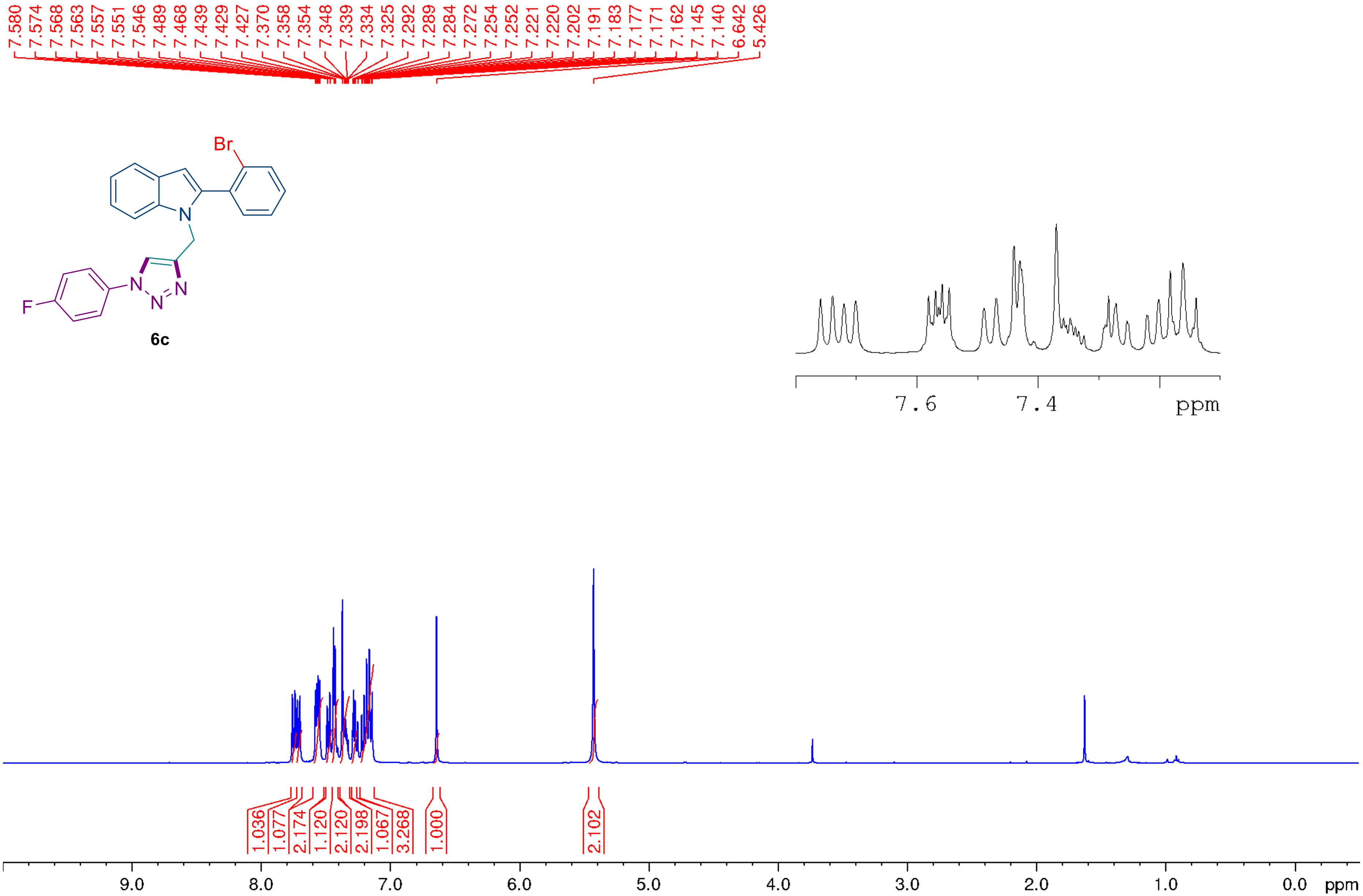

6c

^{13}C NMR-spectrum (100 MHz, $CDCl_3$)

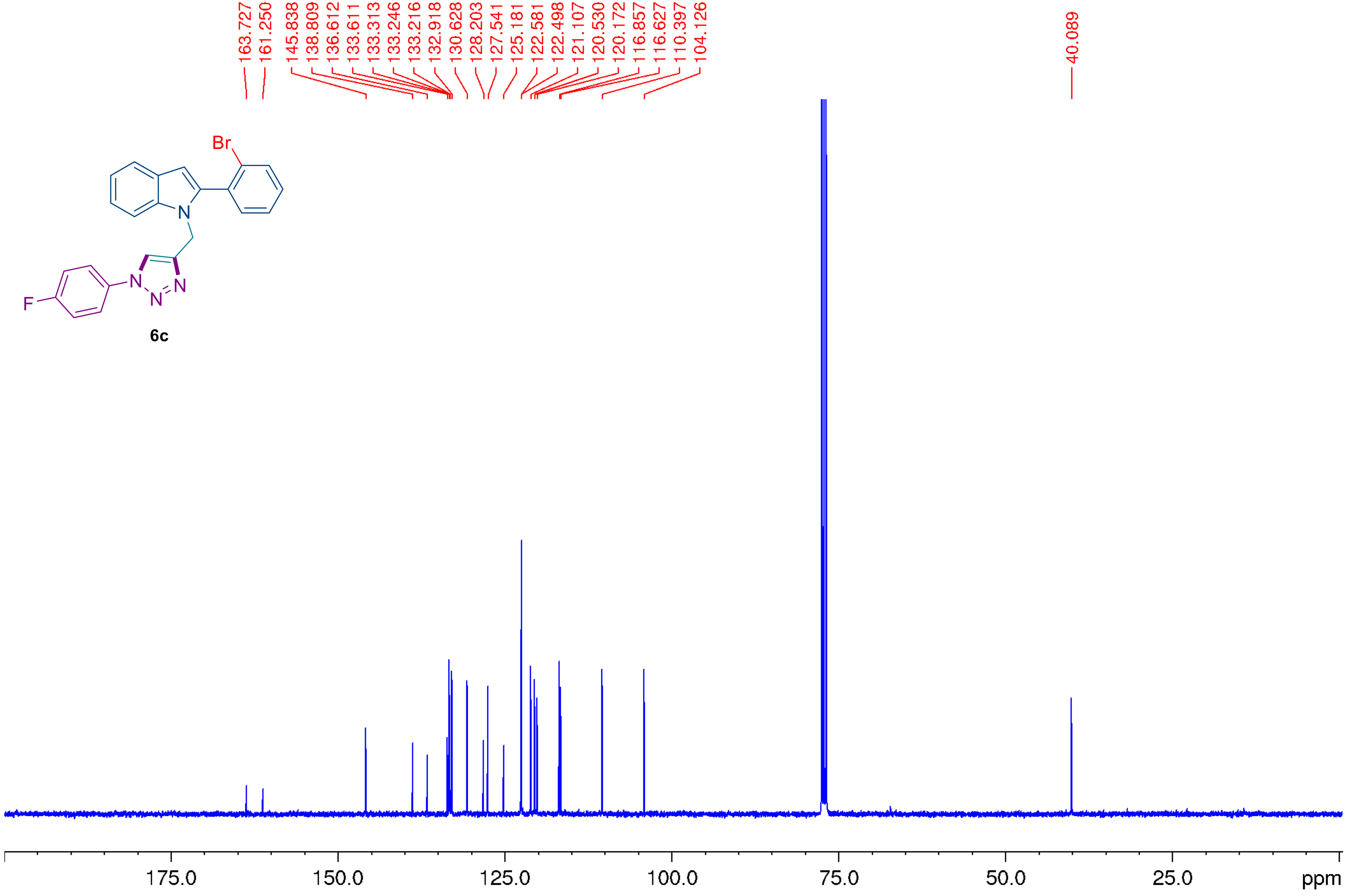

DEPT 135 NMR-spectrum ($CDCl_3$)

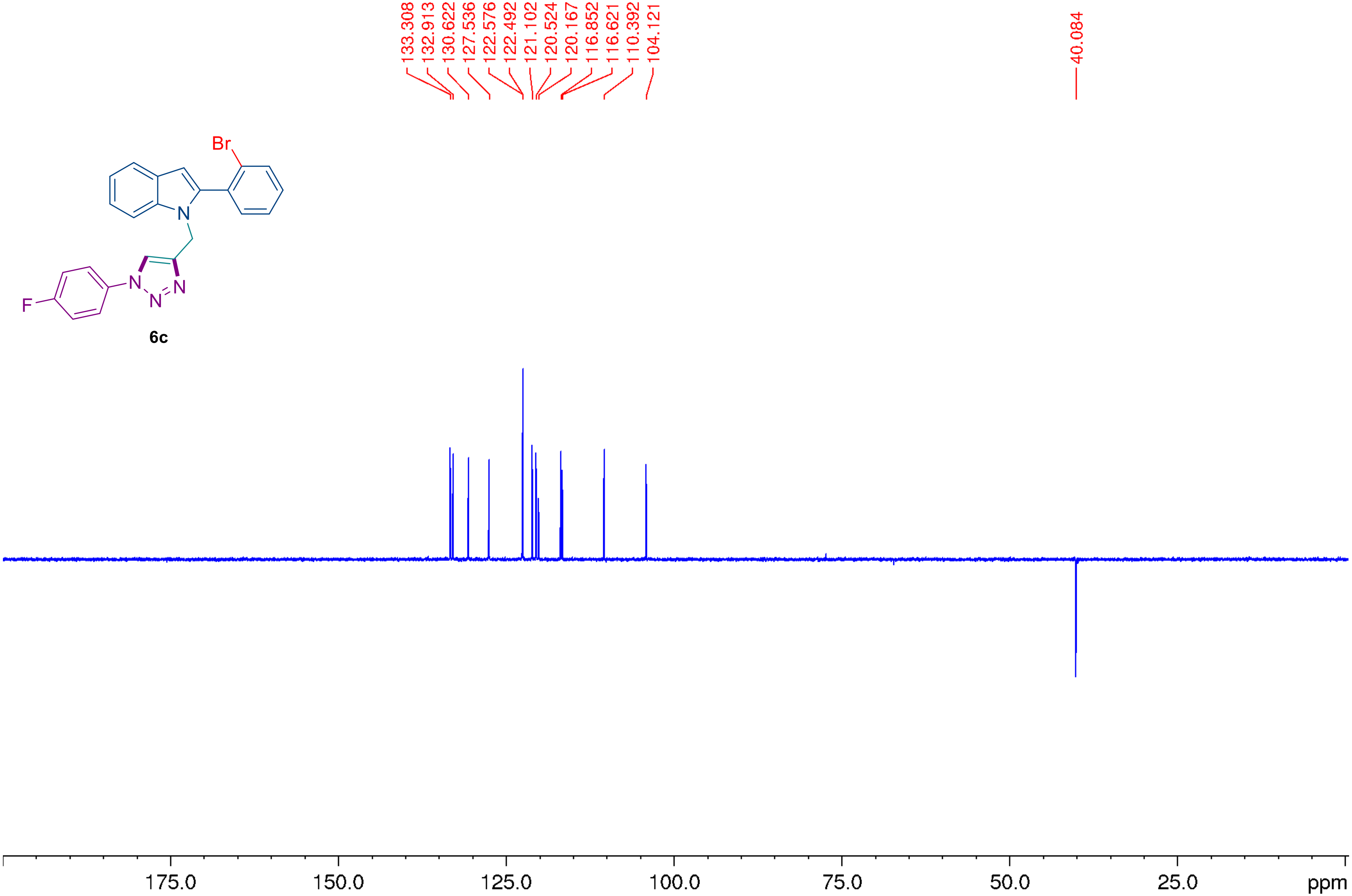

^{19}F NMR-spectrum (376.5 Hz, $CDCl_3$)

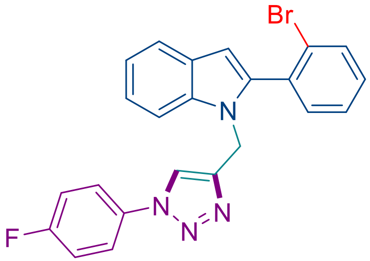

6c

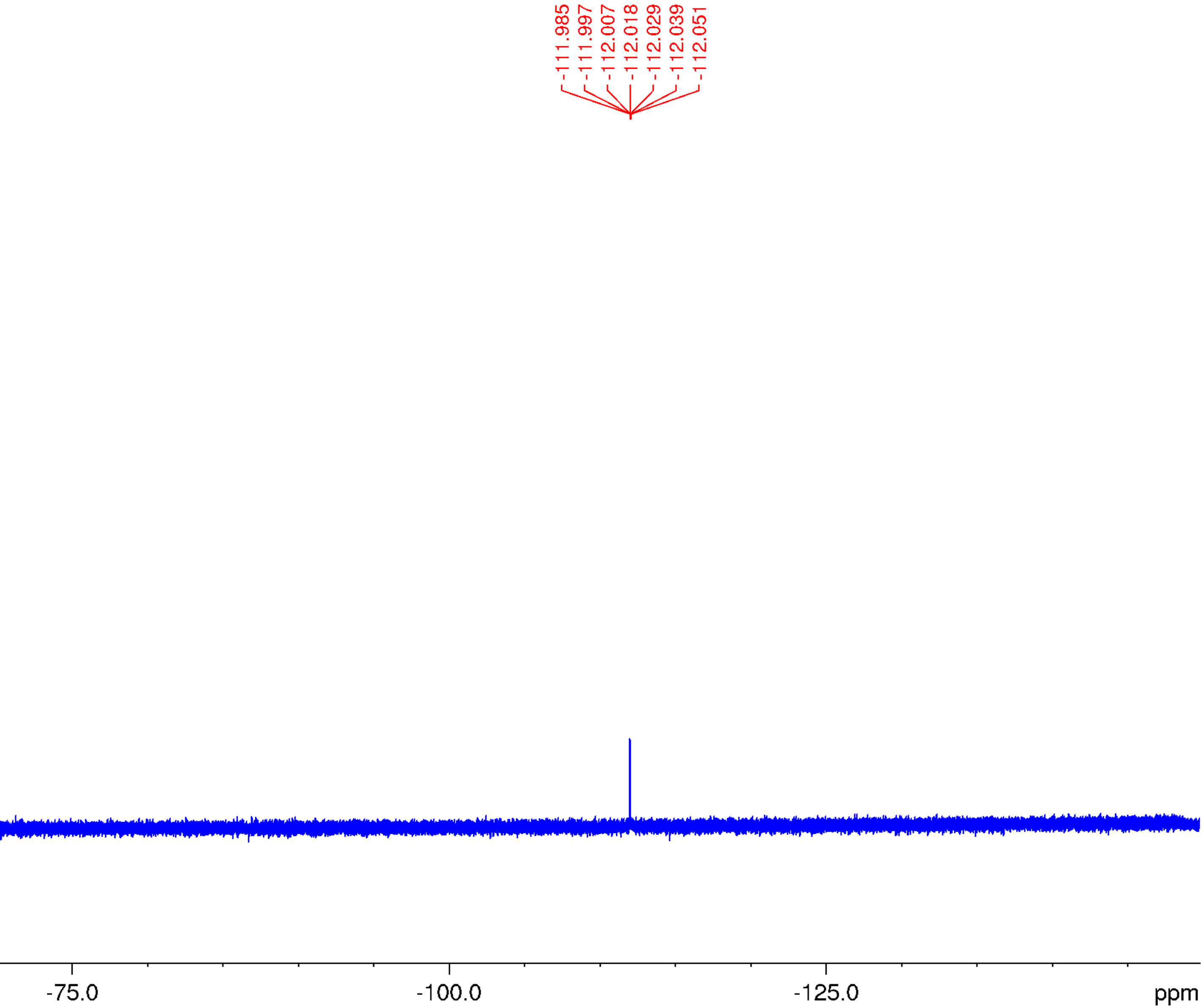

^{1}H NMR-spectrum (400 MHz, $CDCl_3$)

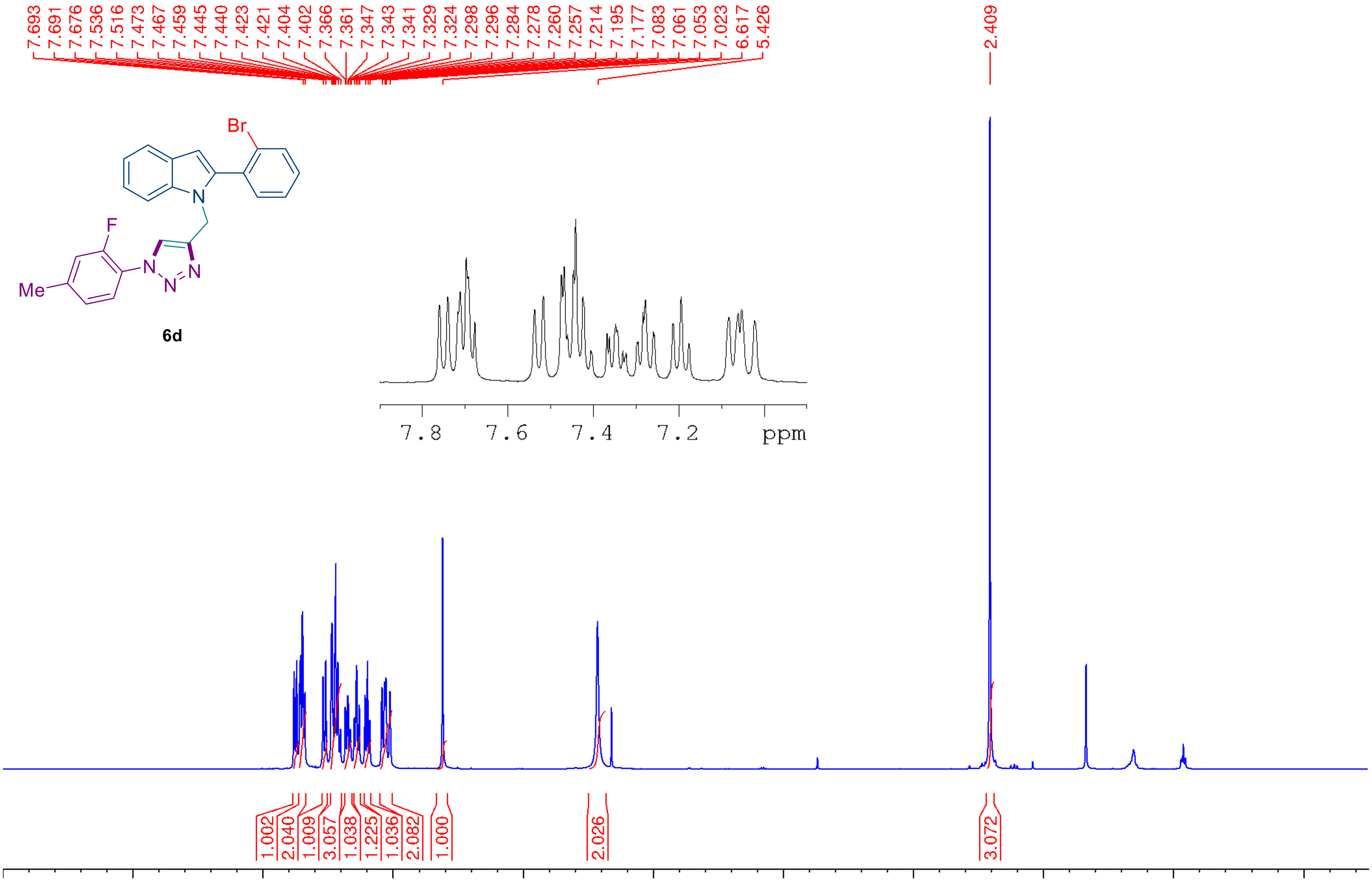

^{13}C NMR-spectrum (100 MHz, $CDCl_3$)

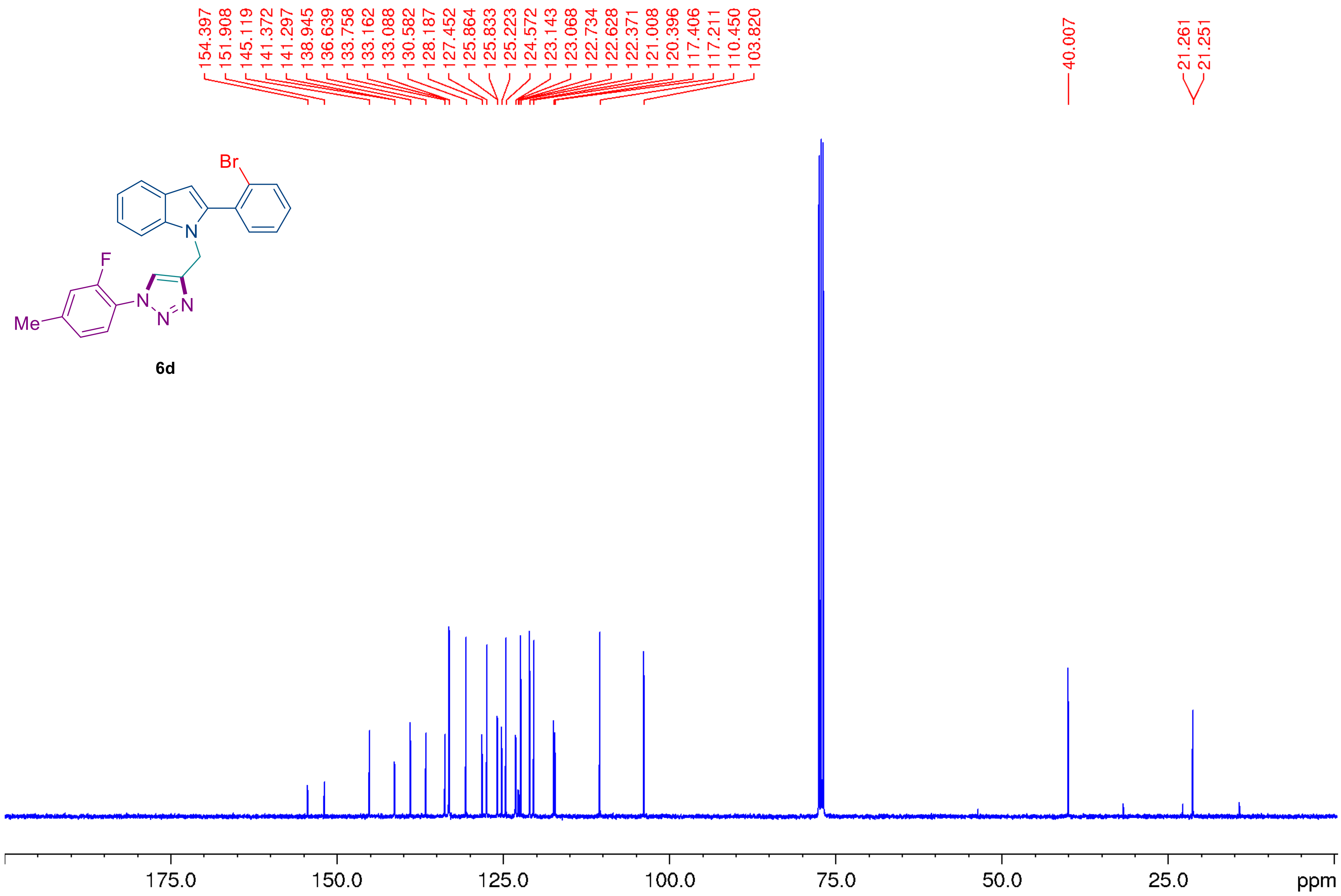

DEPT 135 NMR-spectrum ($CDCl_3$)

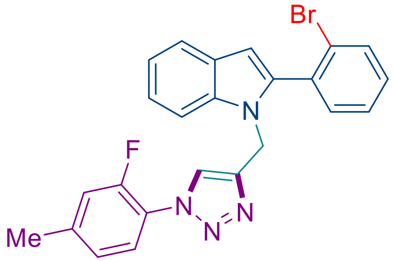

6d

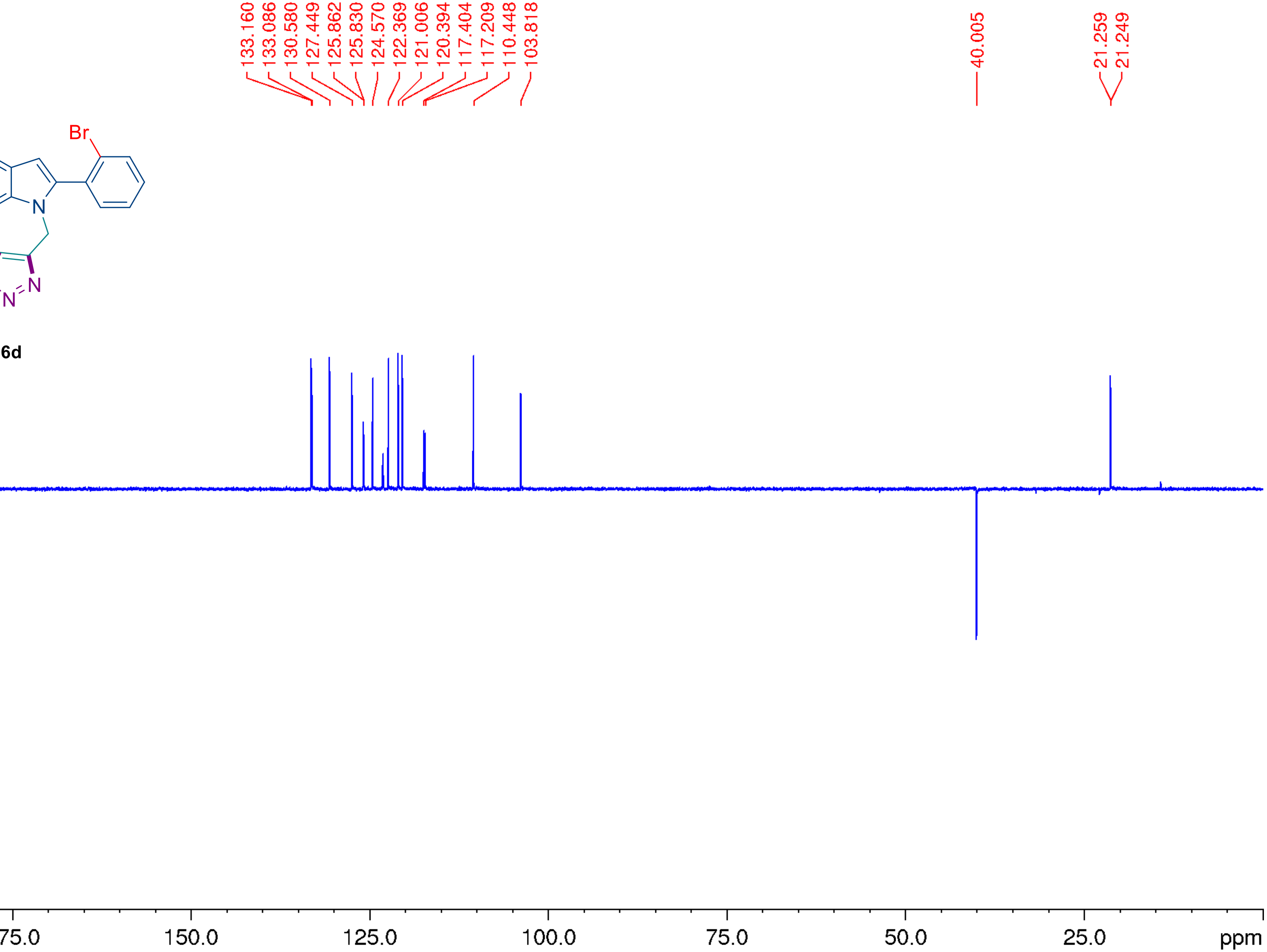

^{19}F NMR-spectrum (376.5 Hz, $CDCl_3$)

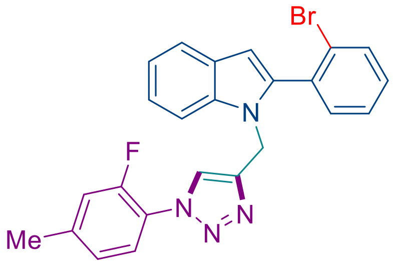

6d

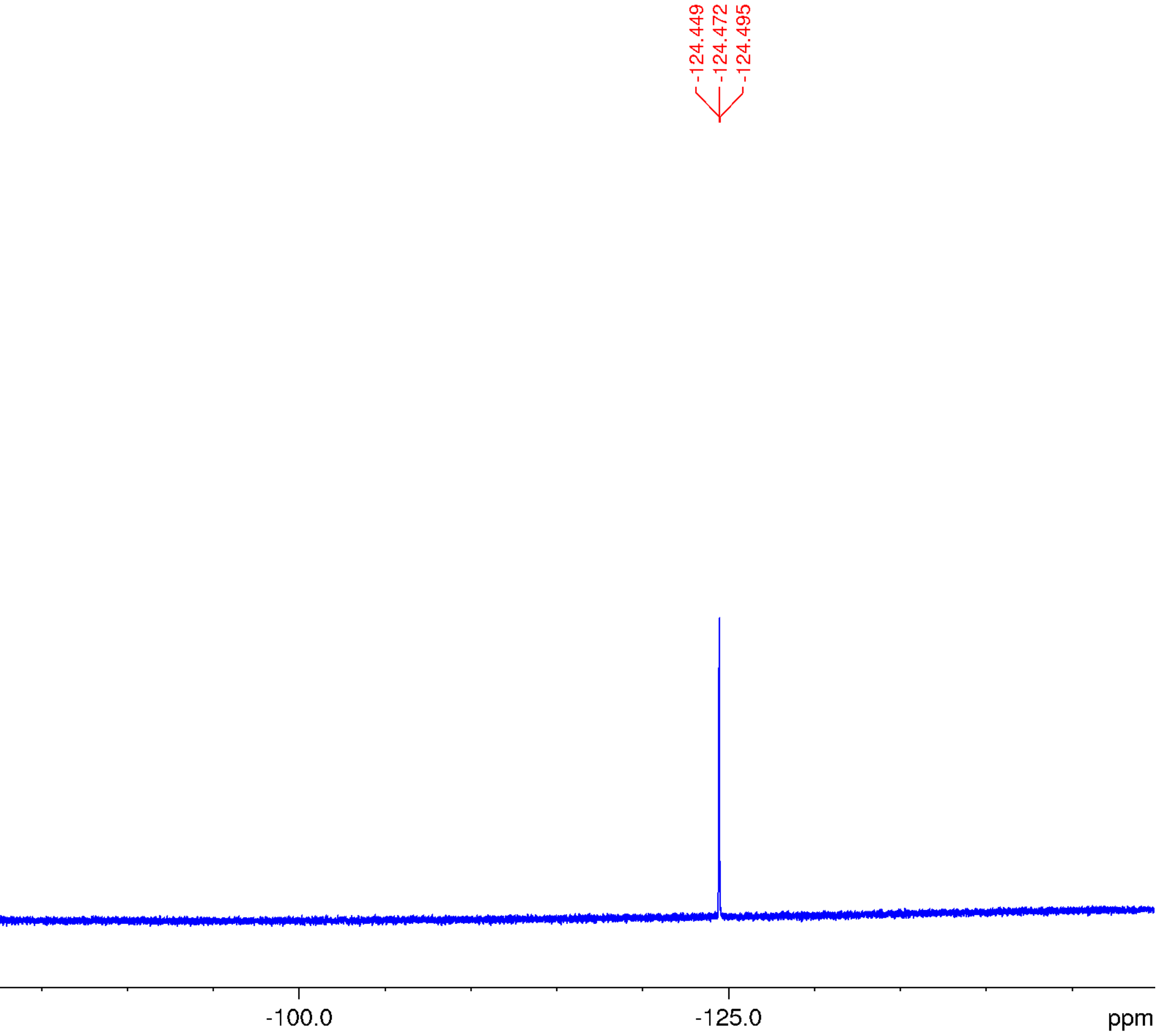

^{1}H NMR-spectrum (400 MHz, $CDCl_3$)

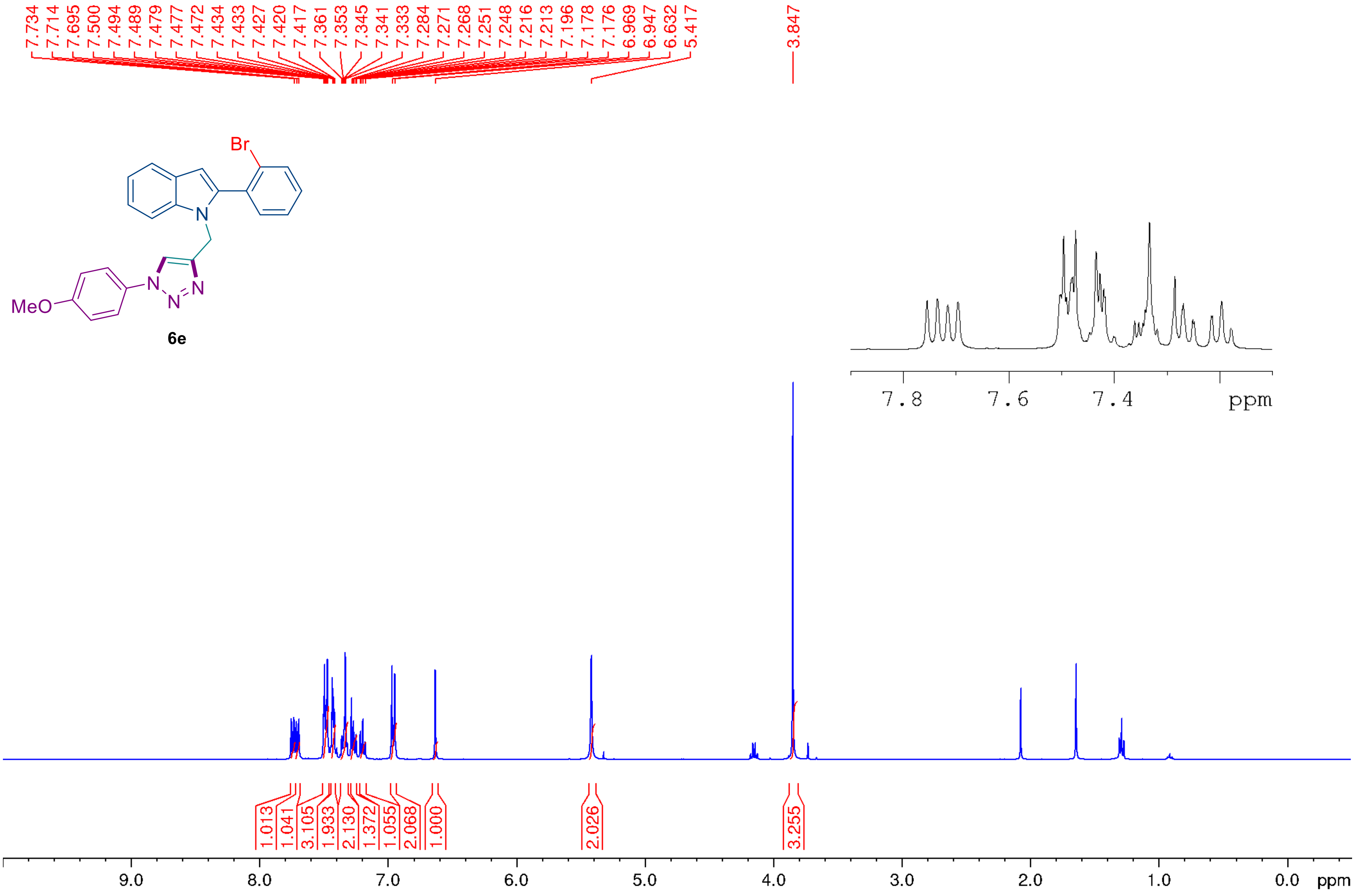

^{13}C NMR-spectrum (100 MHz, $CDCl_3$)

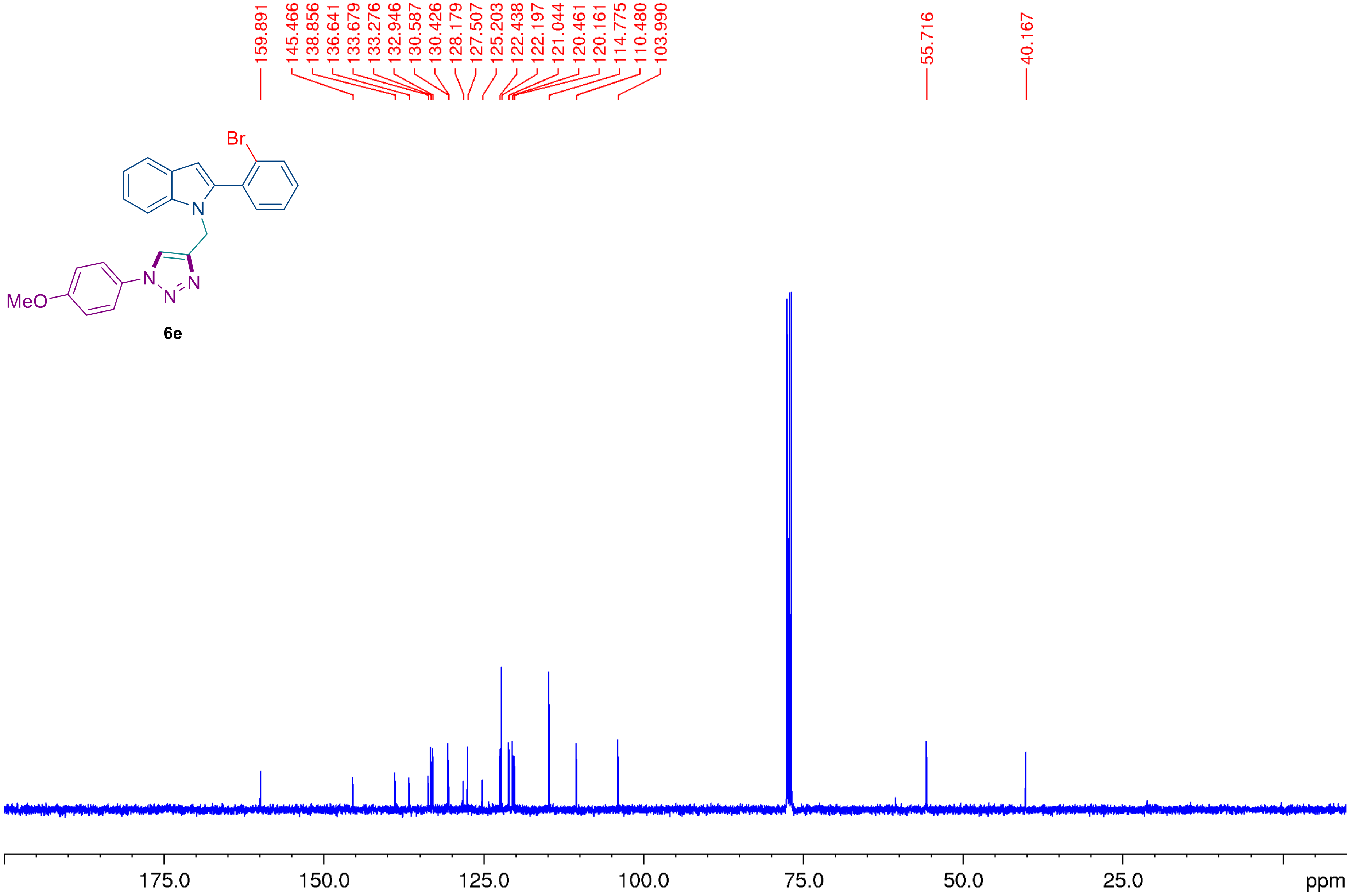

DEPT 135 NMR-spectrum ($CDCl_3$)

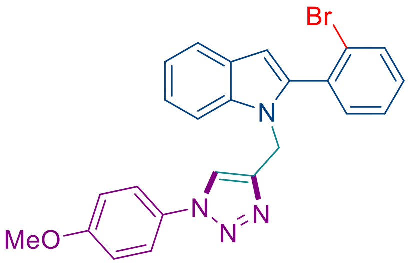

6e

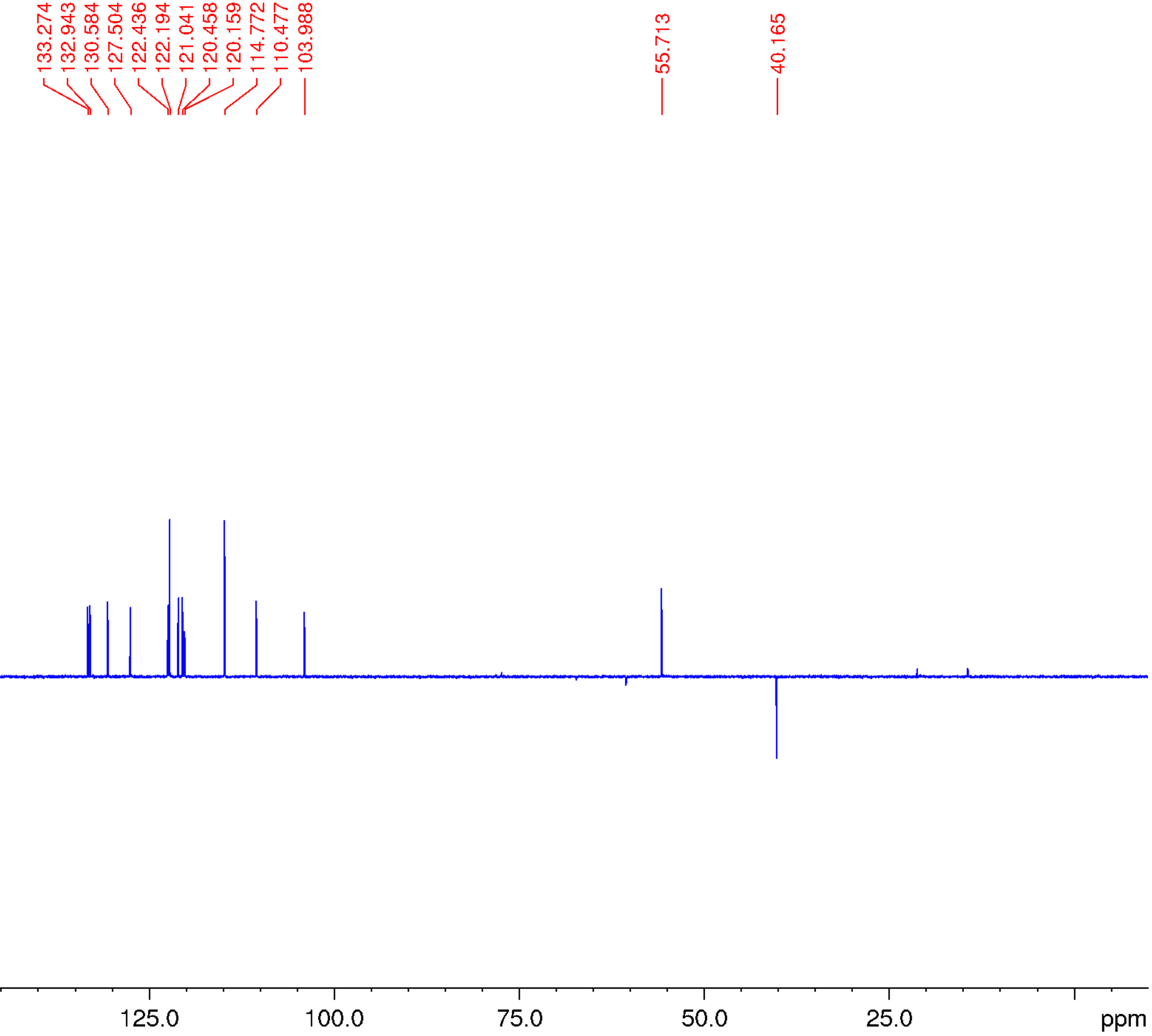

^{1}H NMR-spectrum (400 MHz, $CDCl_3$)

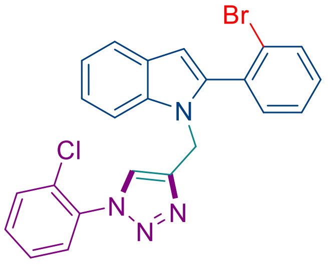

6f

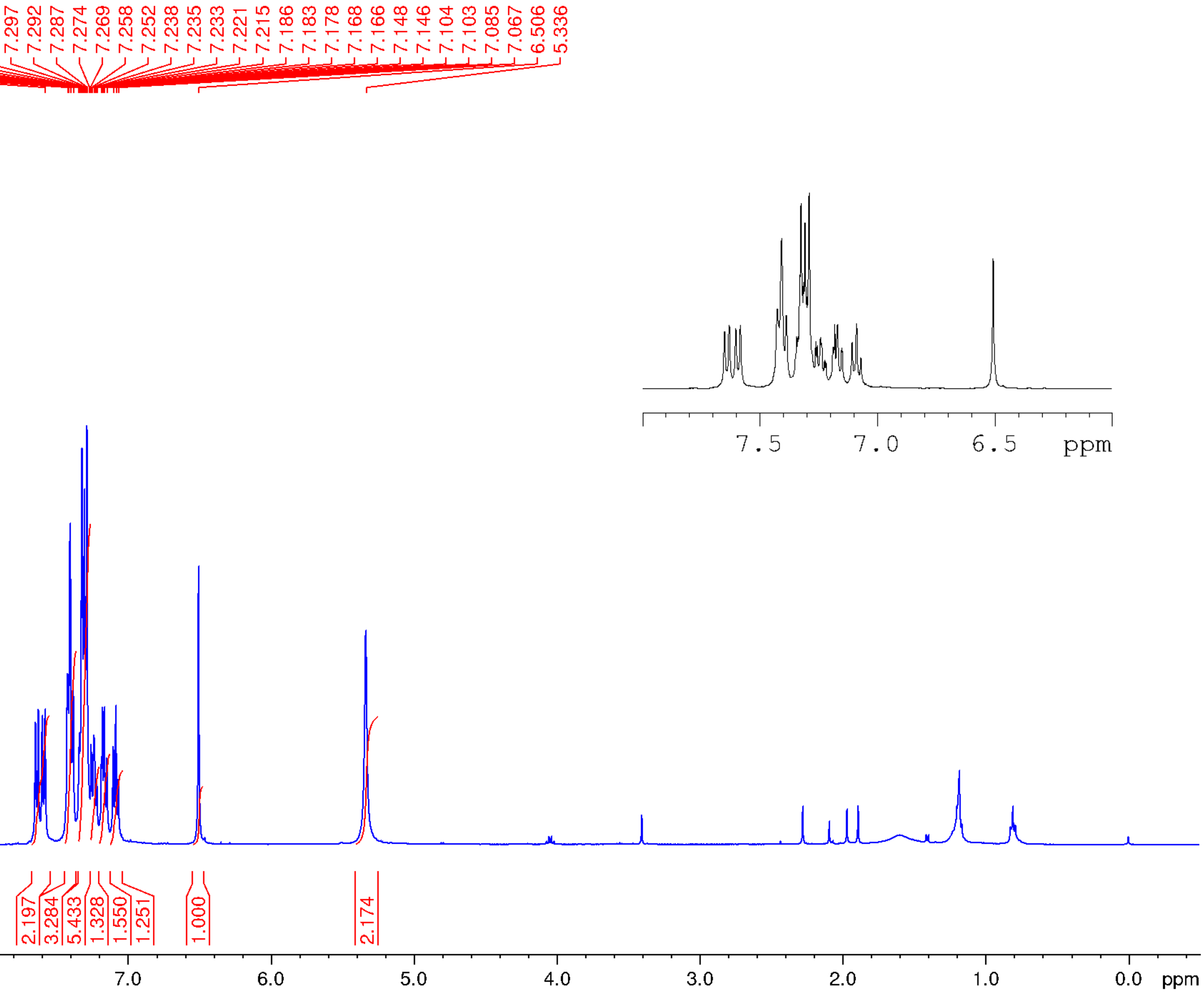

^{13}C NMR-spectrum (100 MHz, $CDCl_3$)

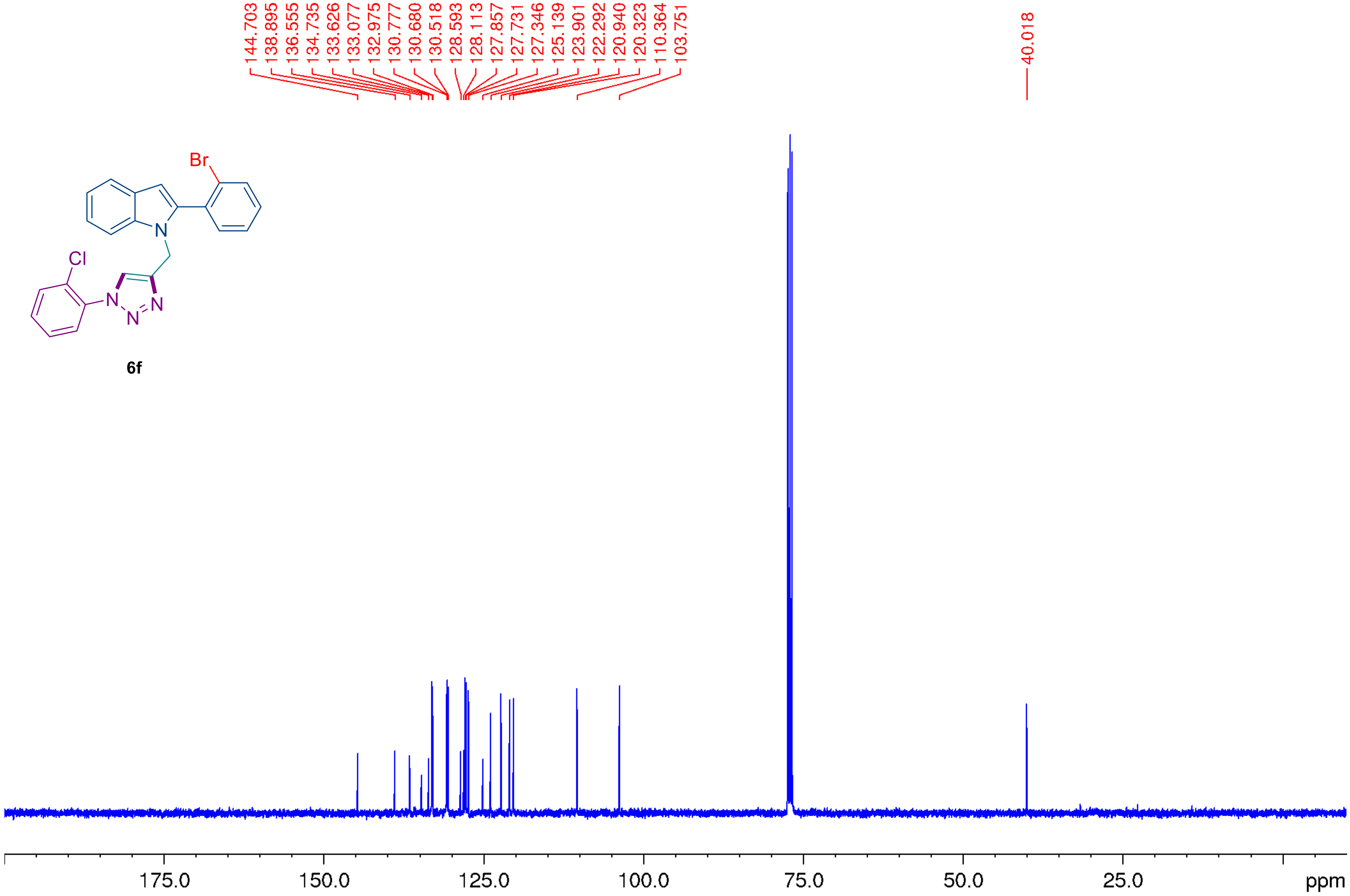

6f

DEPT 135 NMR-spectrum ($CDCl_3$)

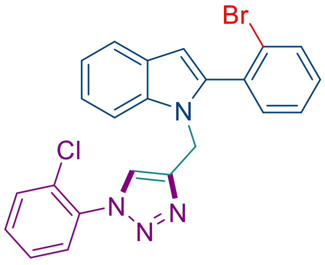

6f

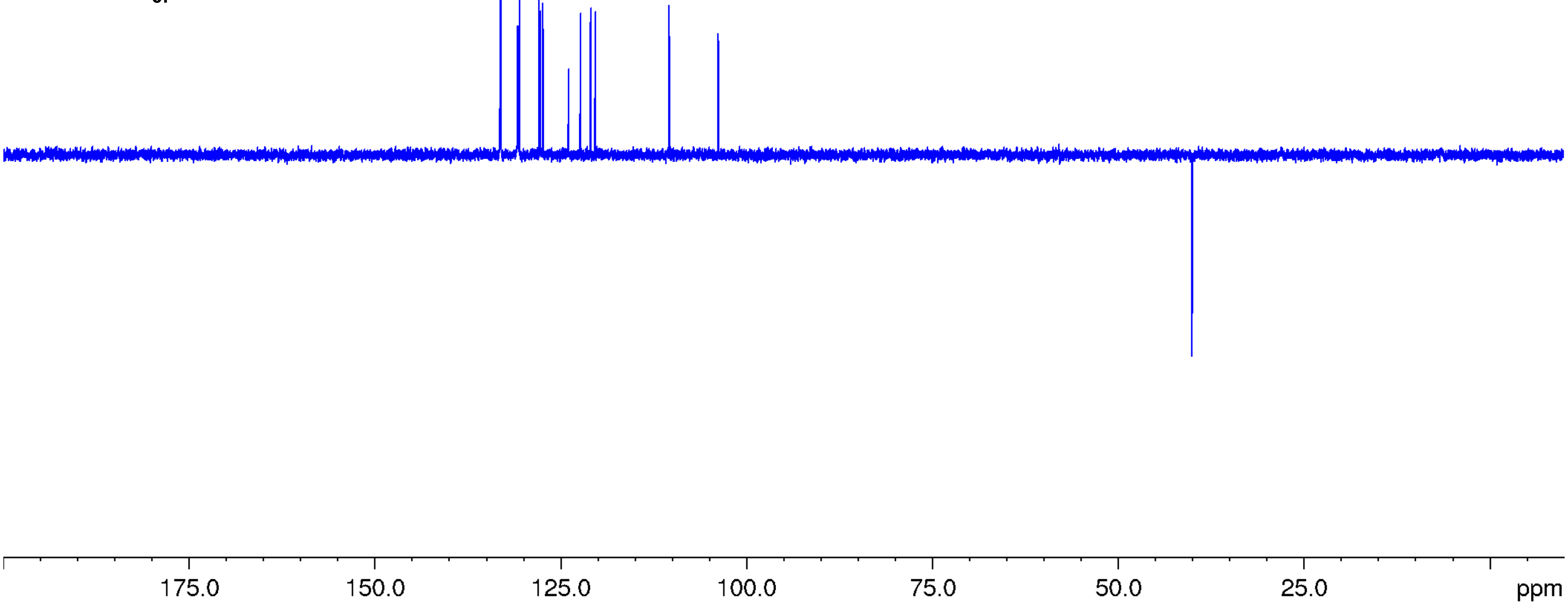

^{1}H NMR-spectrum (400 MHz, $CDCl_3$)

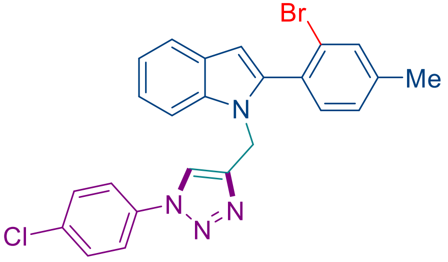

6g

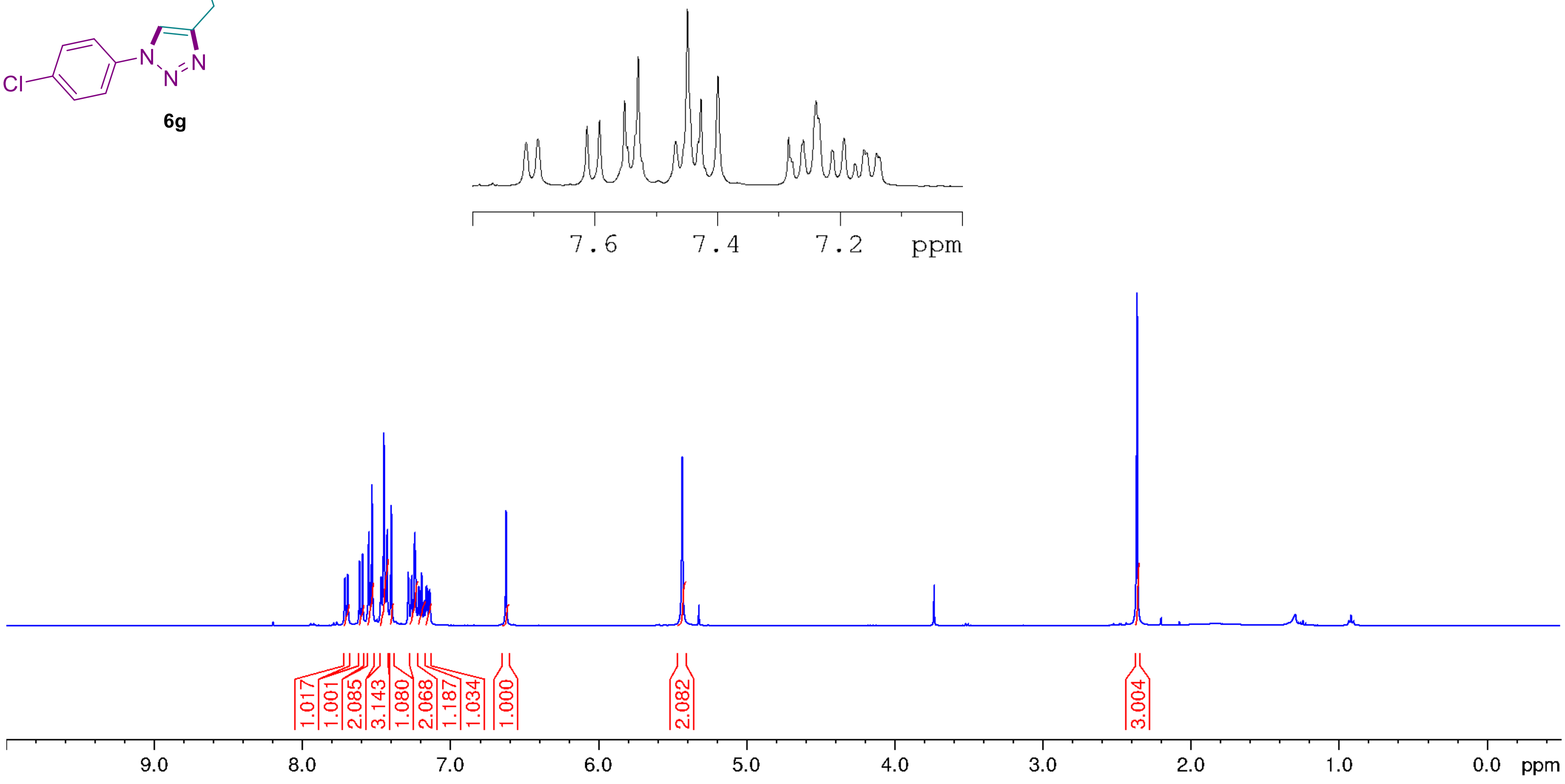

^{13}C NMR-spectrum (100 MHz, $CDCl_3$)

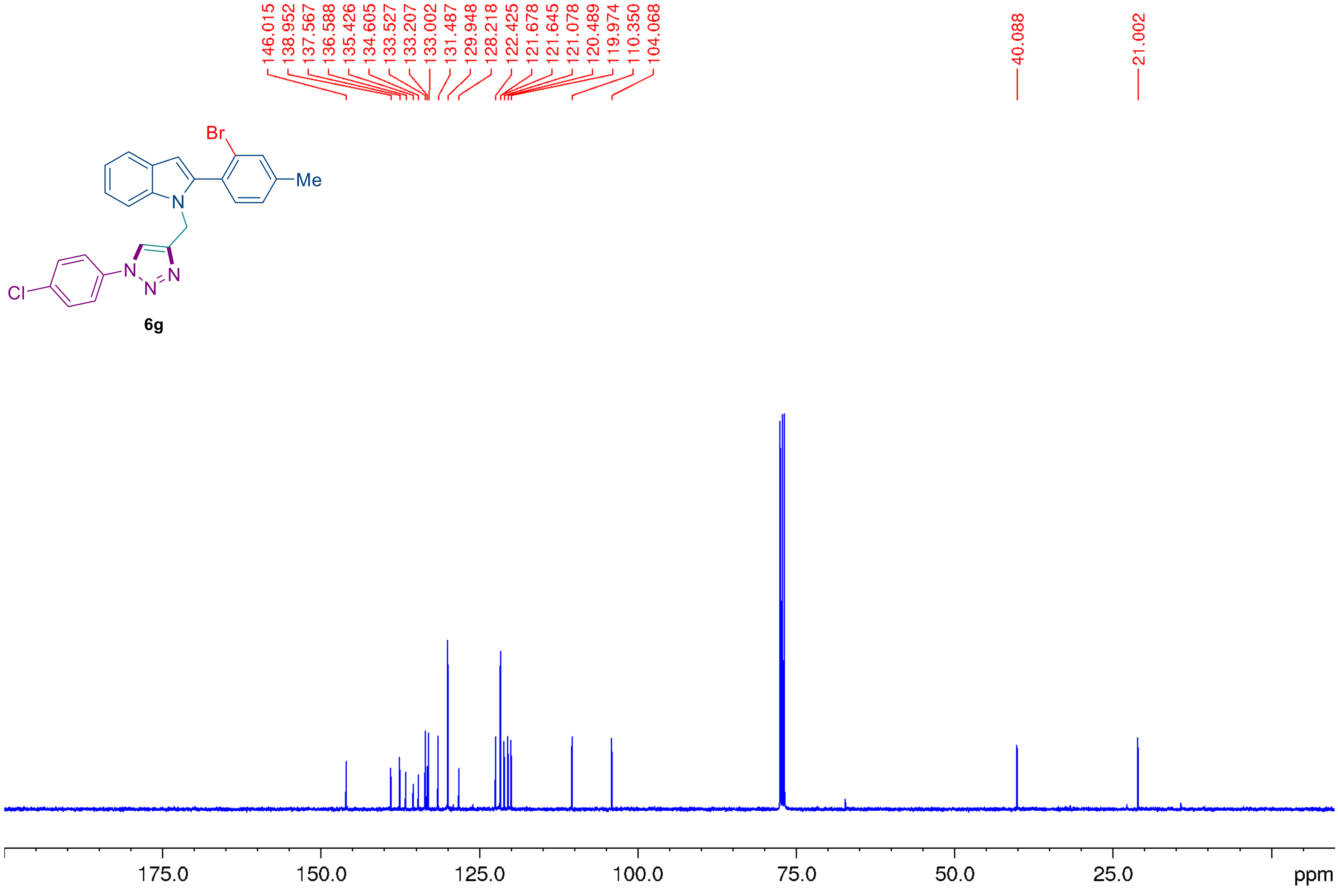

DEPT 135 NMR-spectrum ($CDCl_3$)

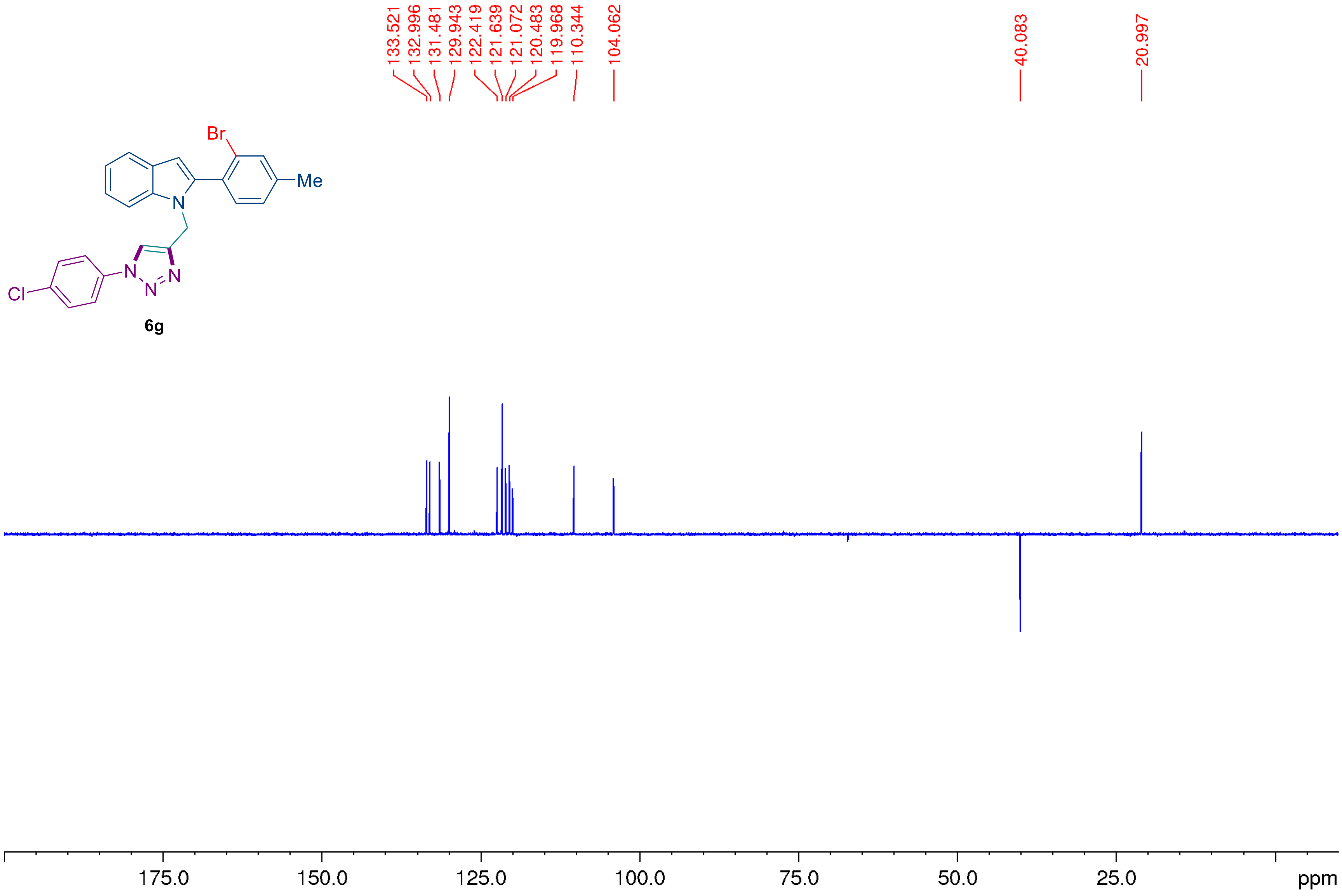

^{1}H NMR-spectrum (400 MHz, $CDCl_3$)

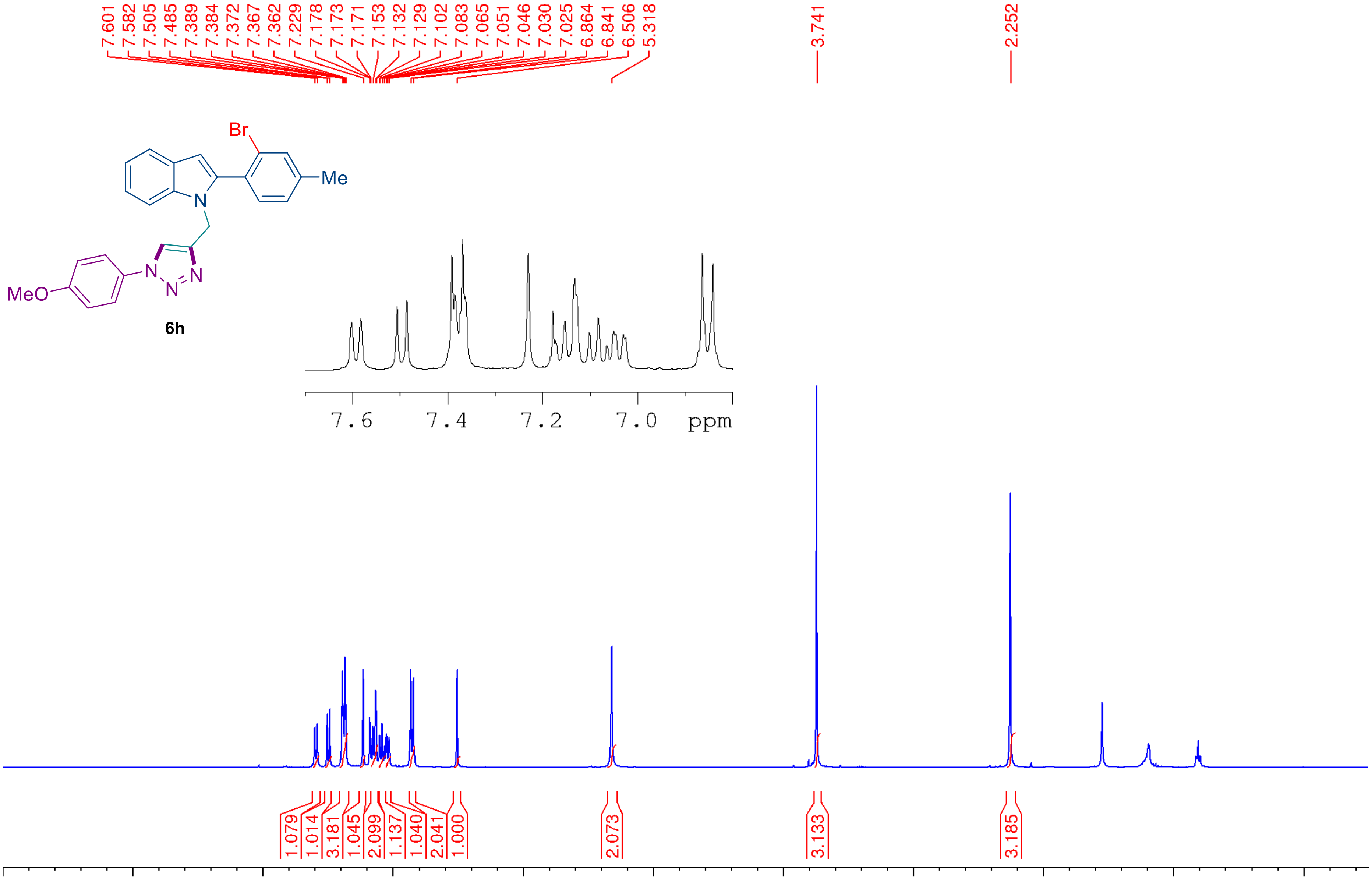

^{13}C NMR-spectrum (100 MHz, $CDCl_3$)

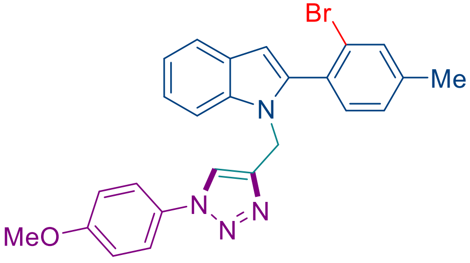

6h

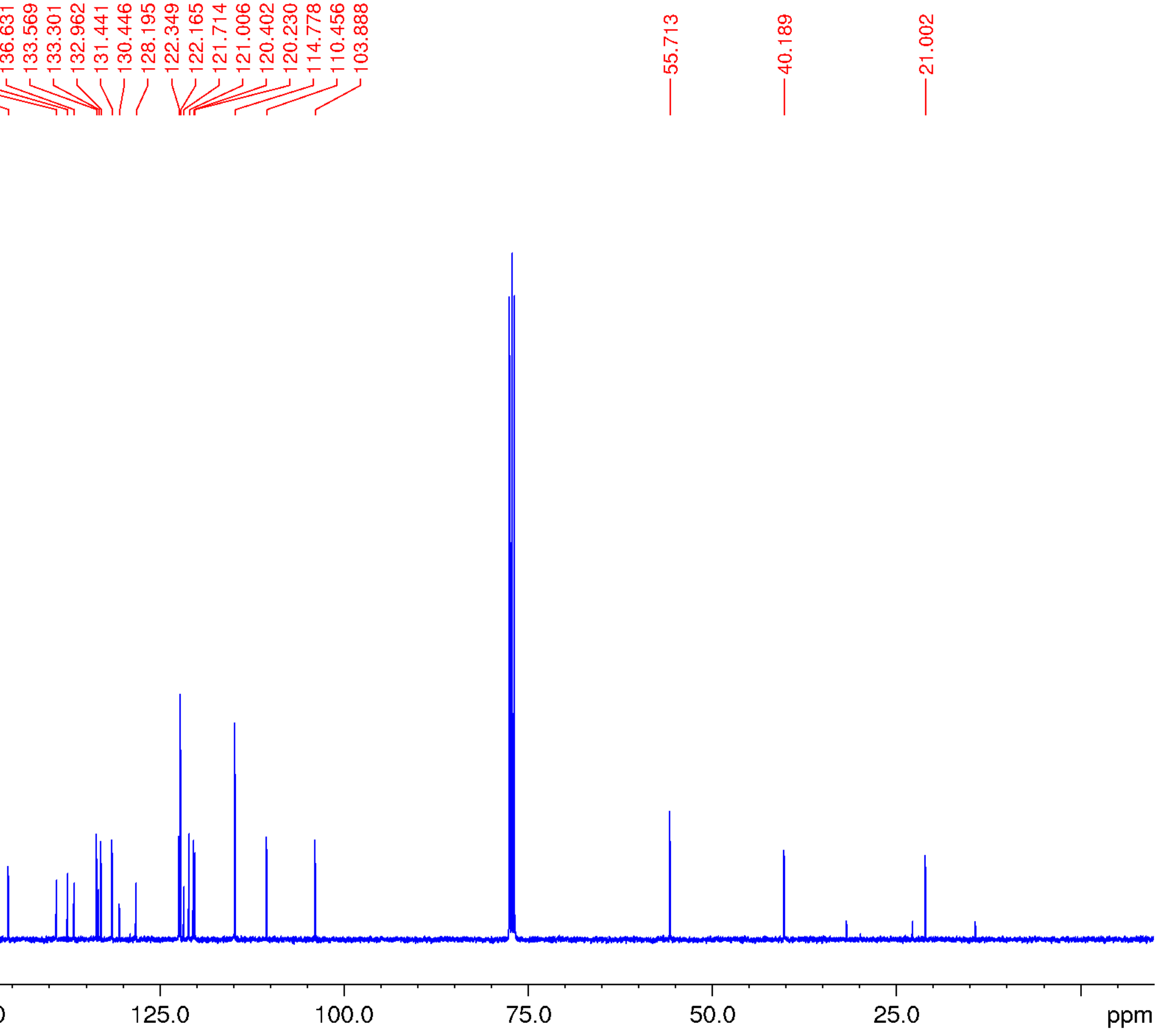

DEPT 135 NMR-spectrum ($CDCl_3$)

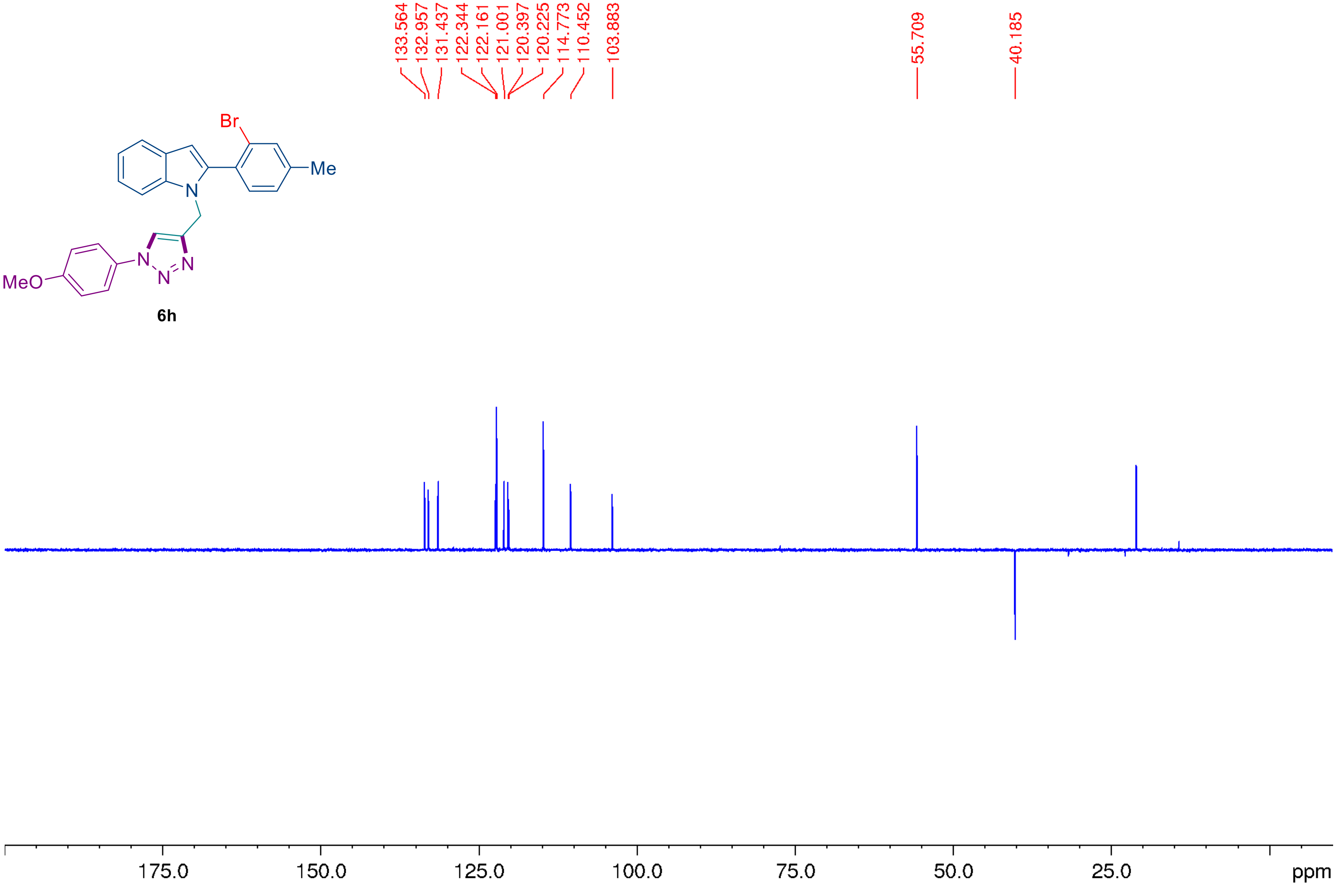

^1H NMR-spectrum (400 MHz, $CDCl_3$)

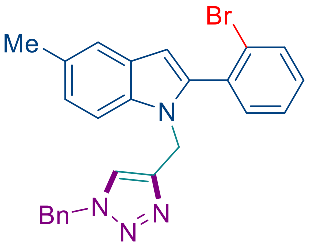

6i

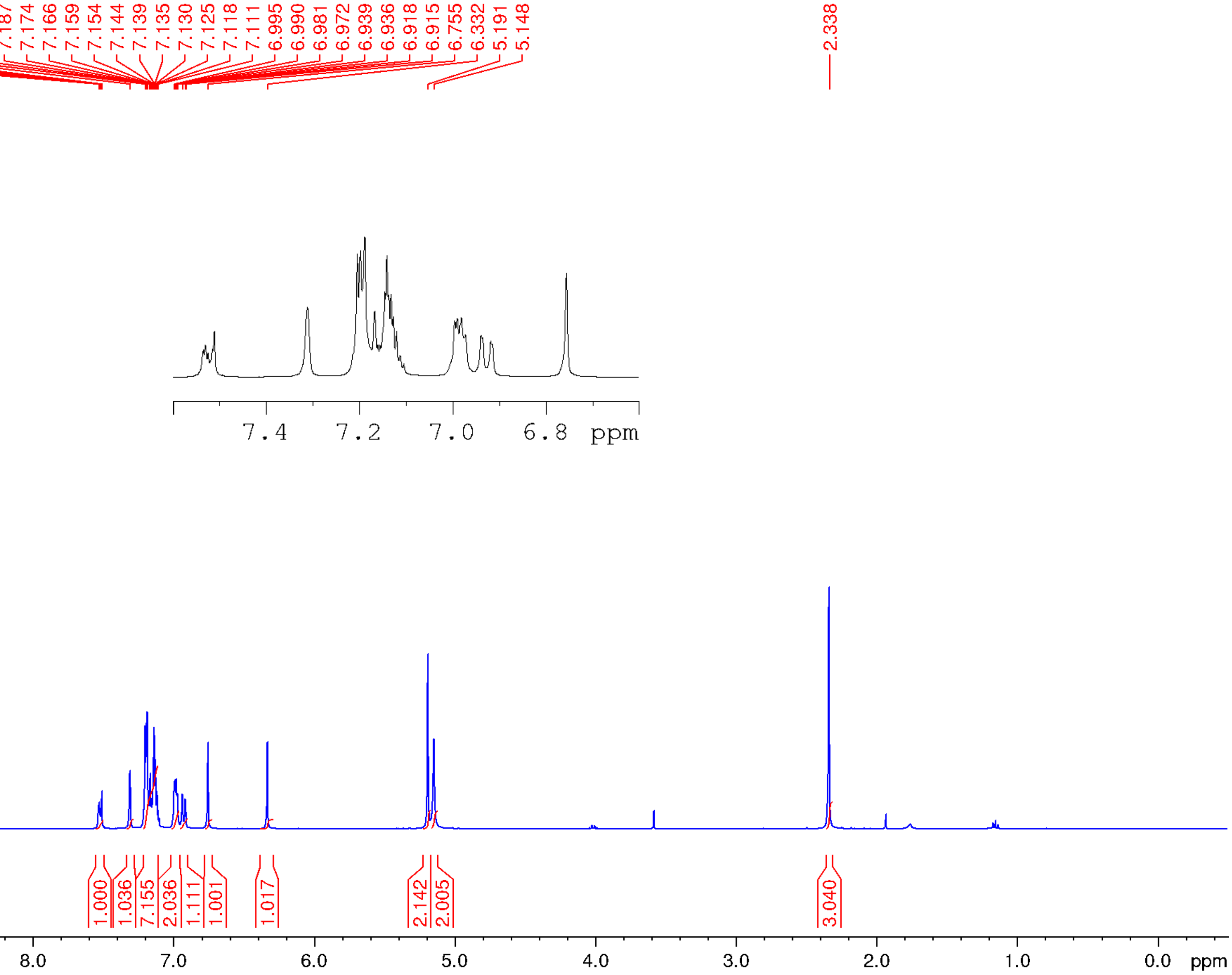

^{13}C NMR-spectrum (100 MHz, $CDCl_3$)

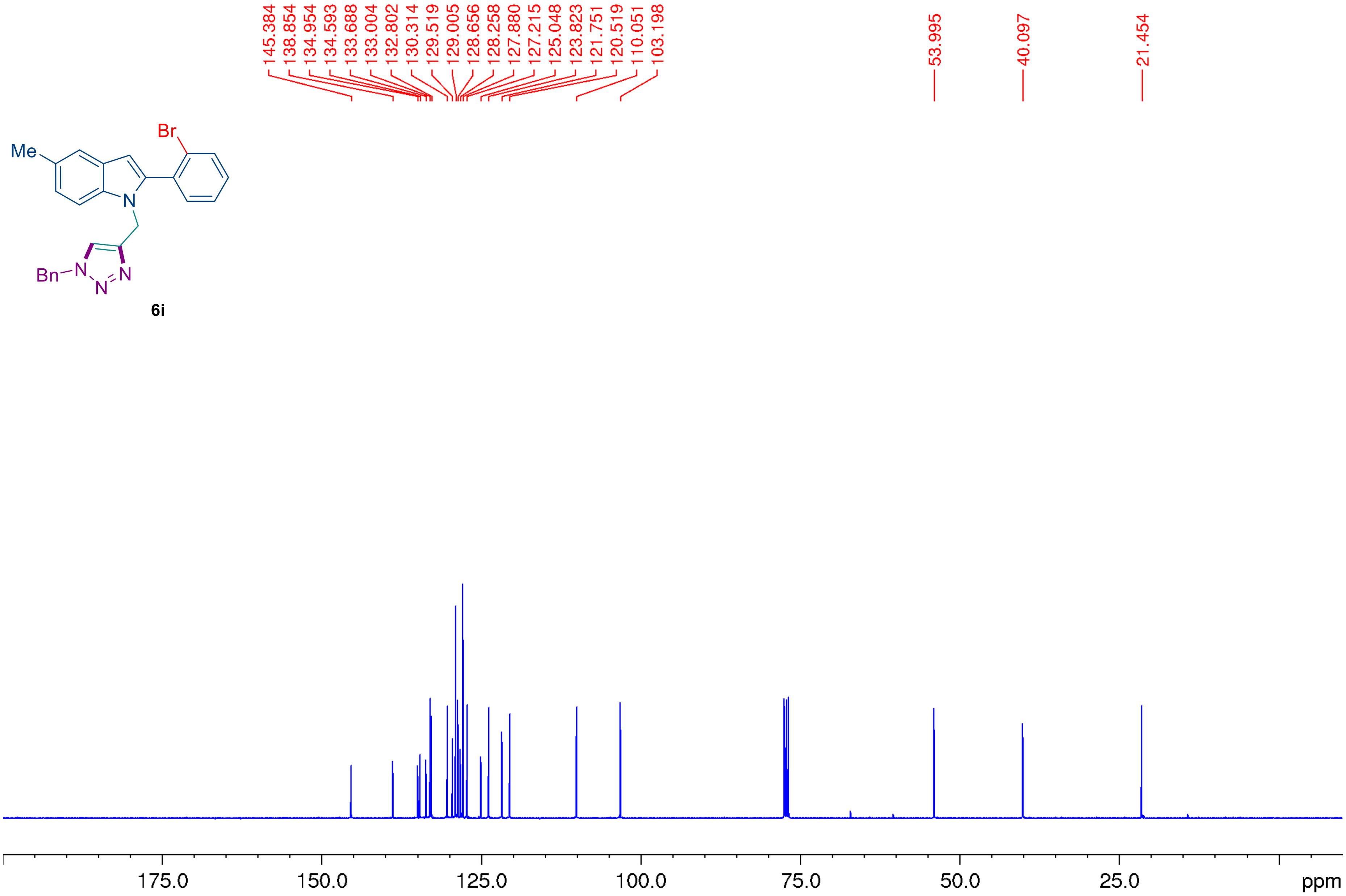

6i

DEPT 135 NMR-spectrum ($CDCl_3$)

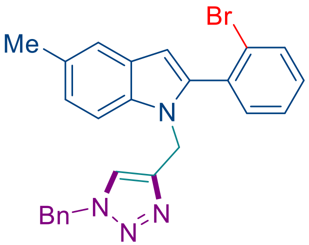

6i

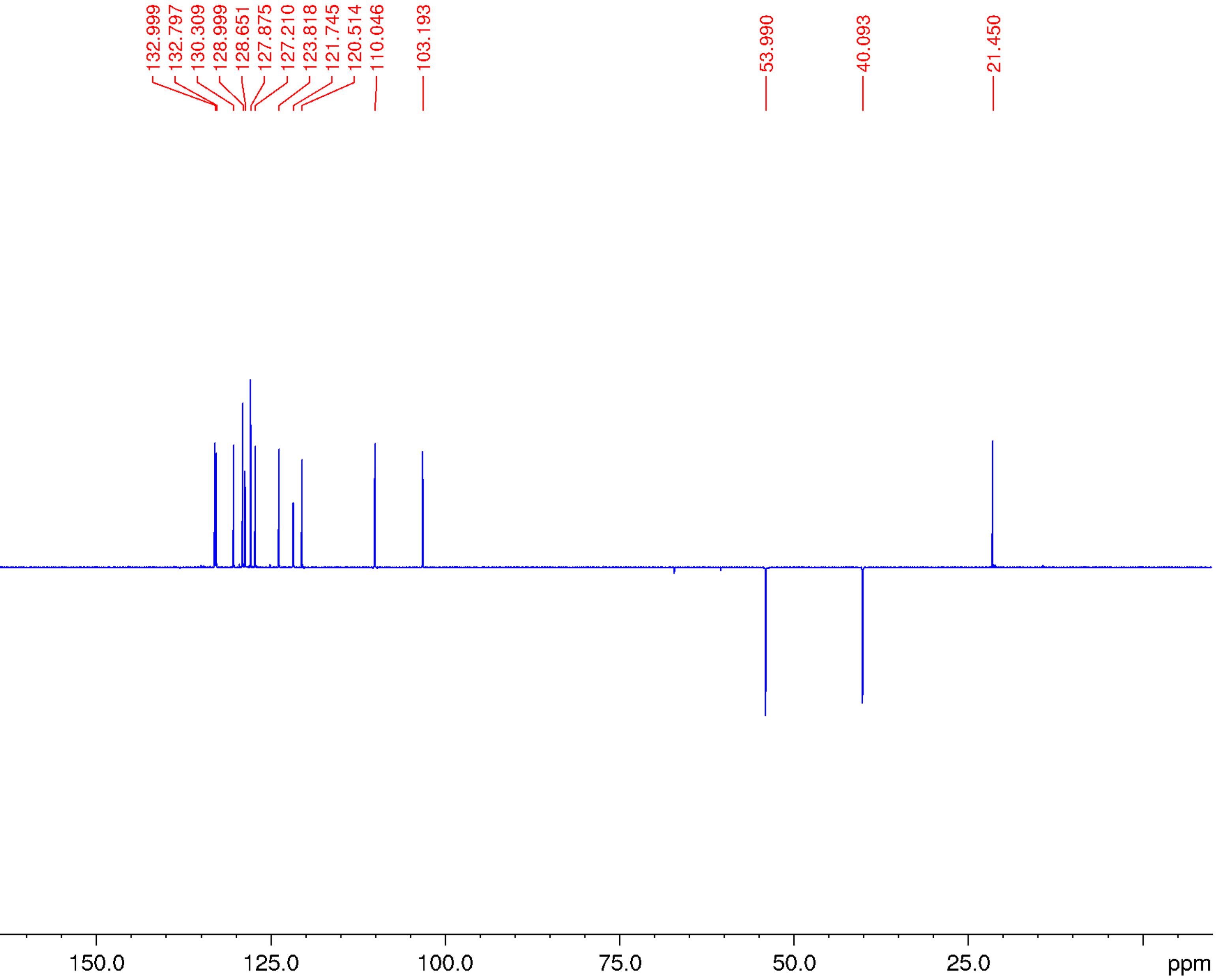

^{1}H NMR-spectrum (400 MHz, $CDCl_3$)

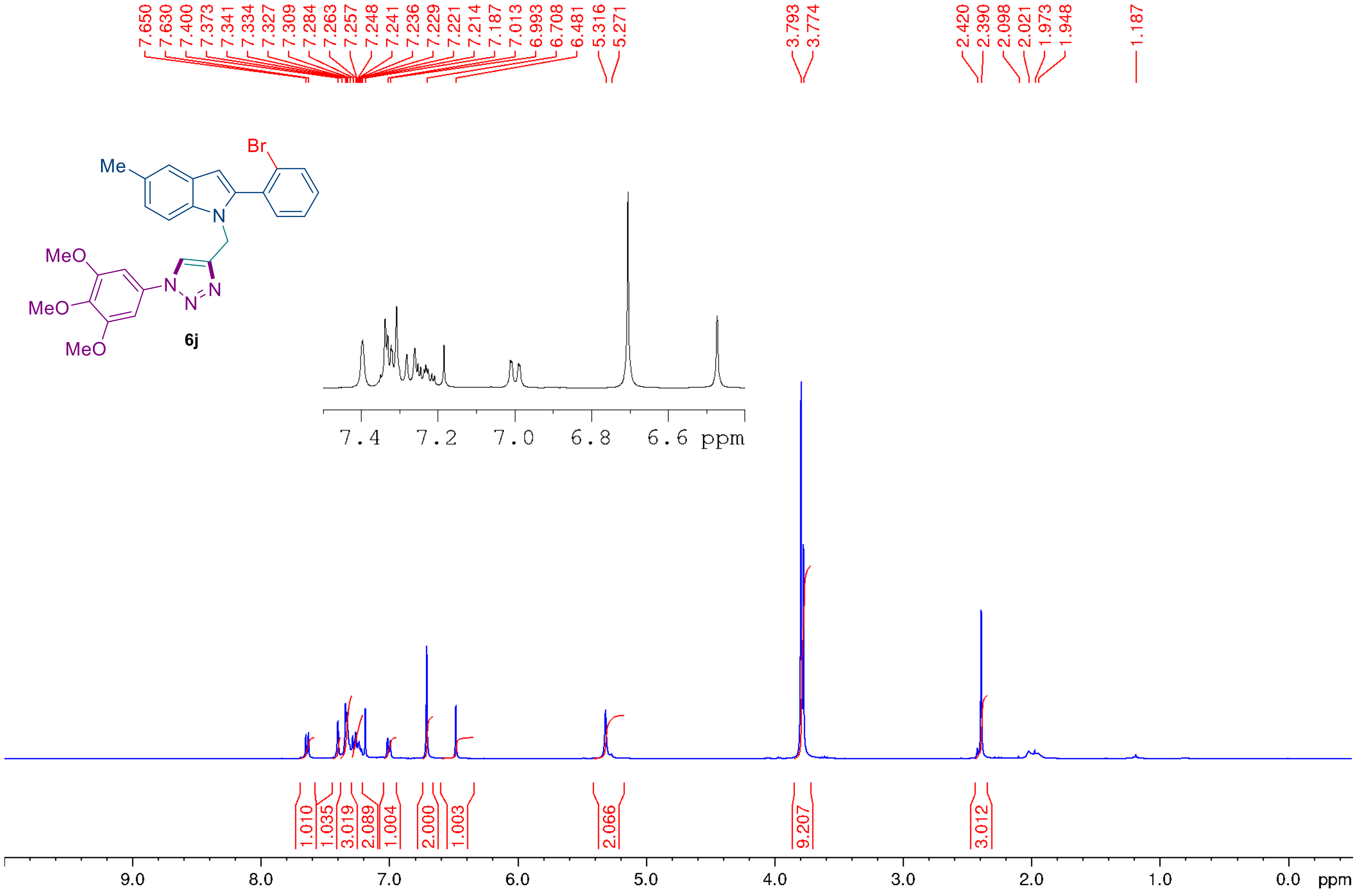

^{13}C NMR-spectrum (100 MHz, $CDCl_3$)

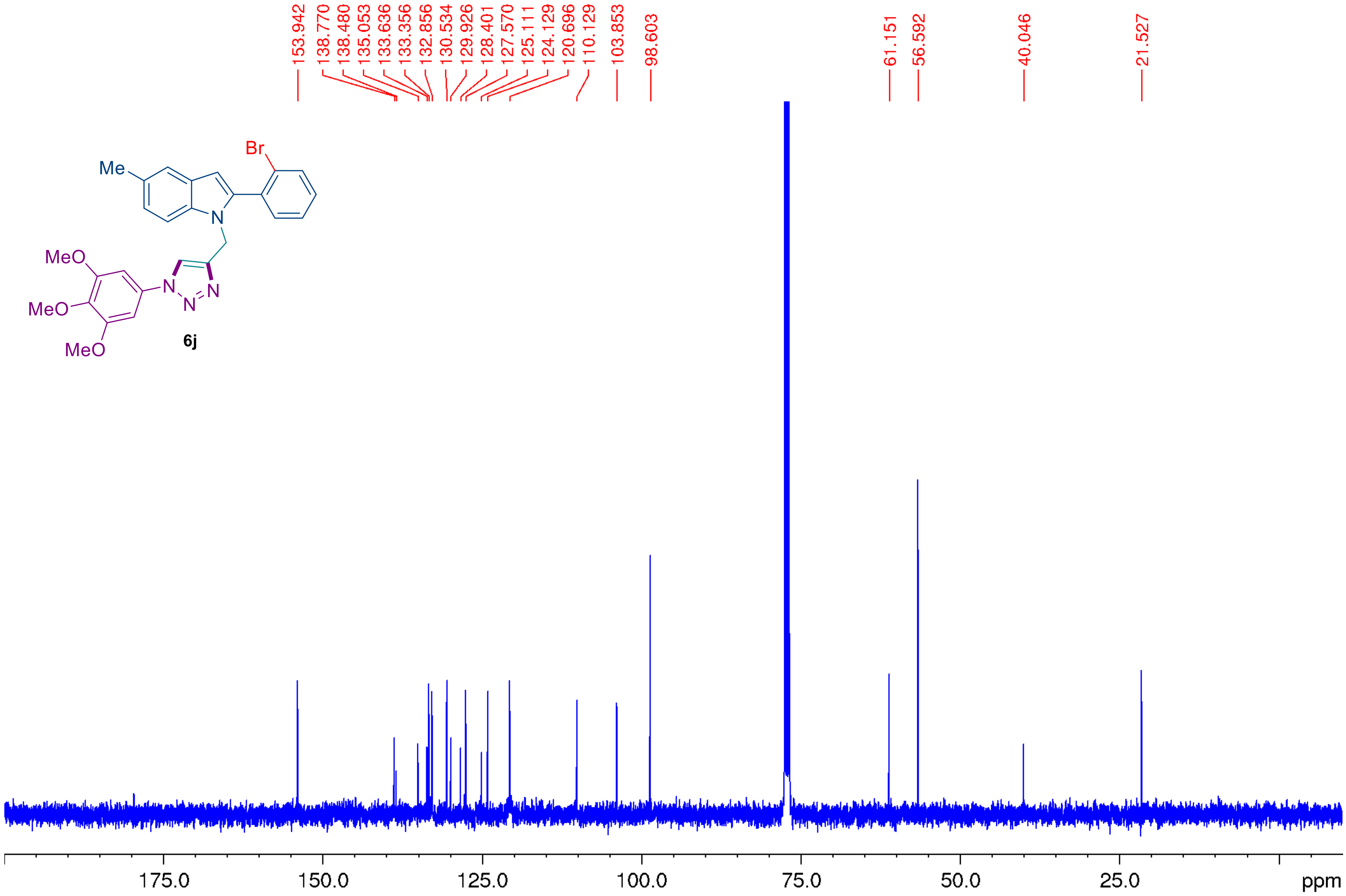

DEPT 135 NMR-spectrum ($CDCl_3$)

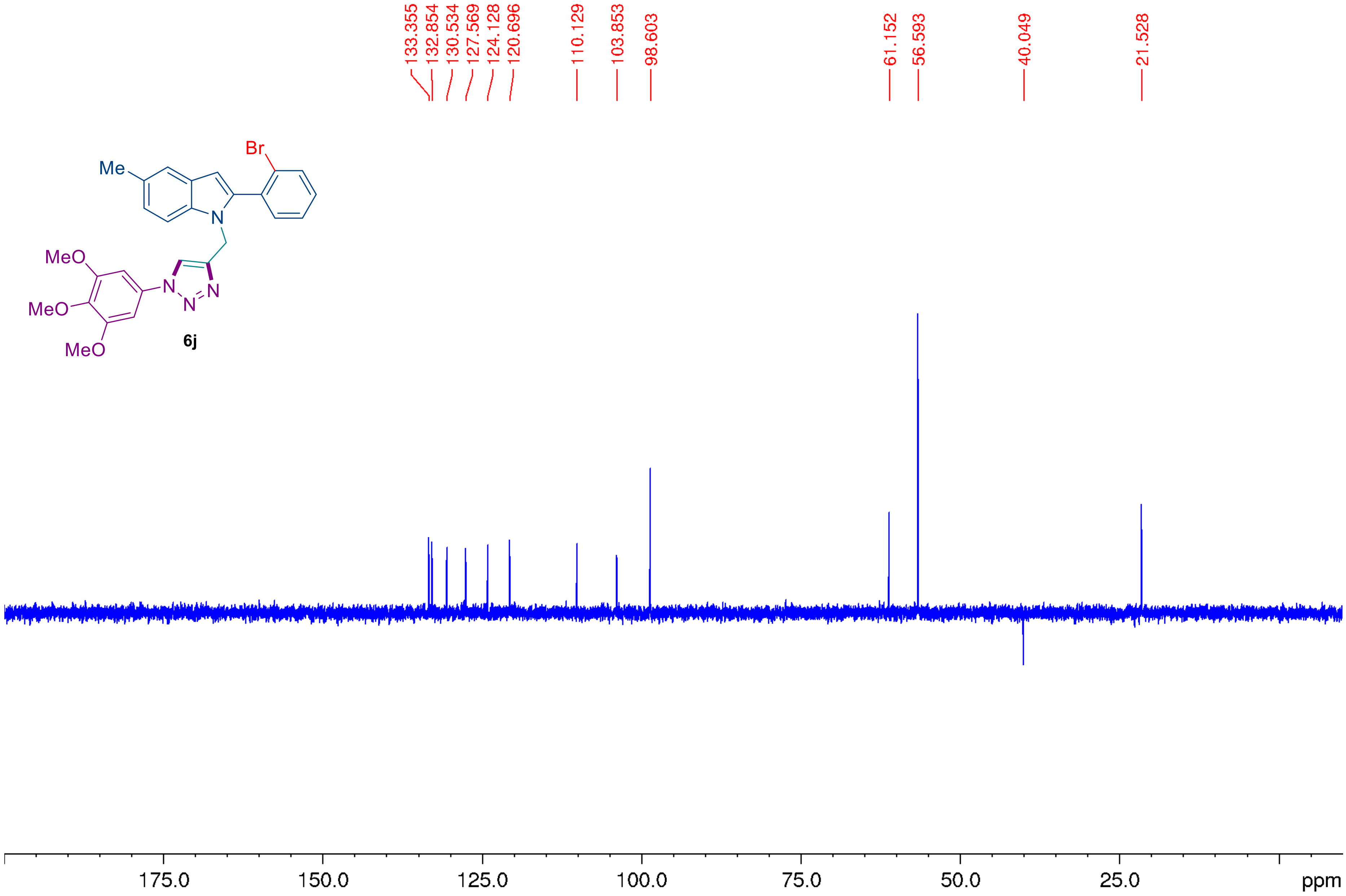

^{1}H NMR-spectrum (400 MHz, $CDCl_3$)

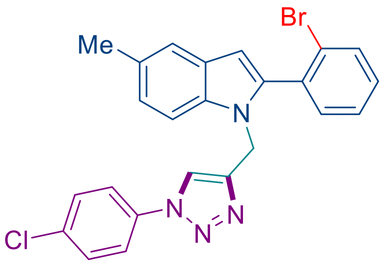

6k

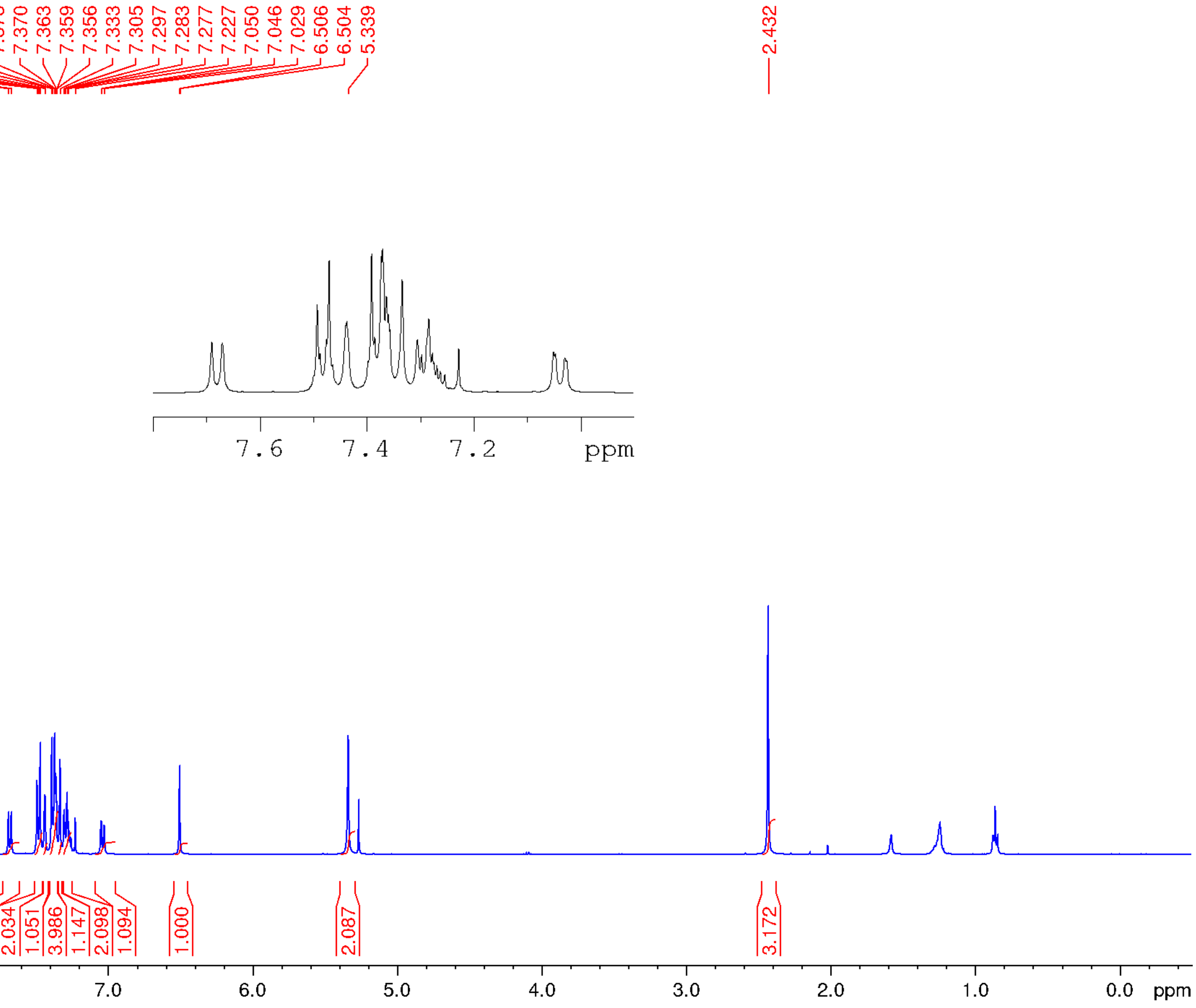

^{13}C NMR-spectrum (100 MHz, $CDCl_3$)

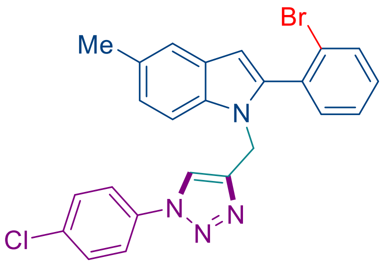

6k

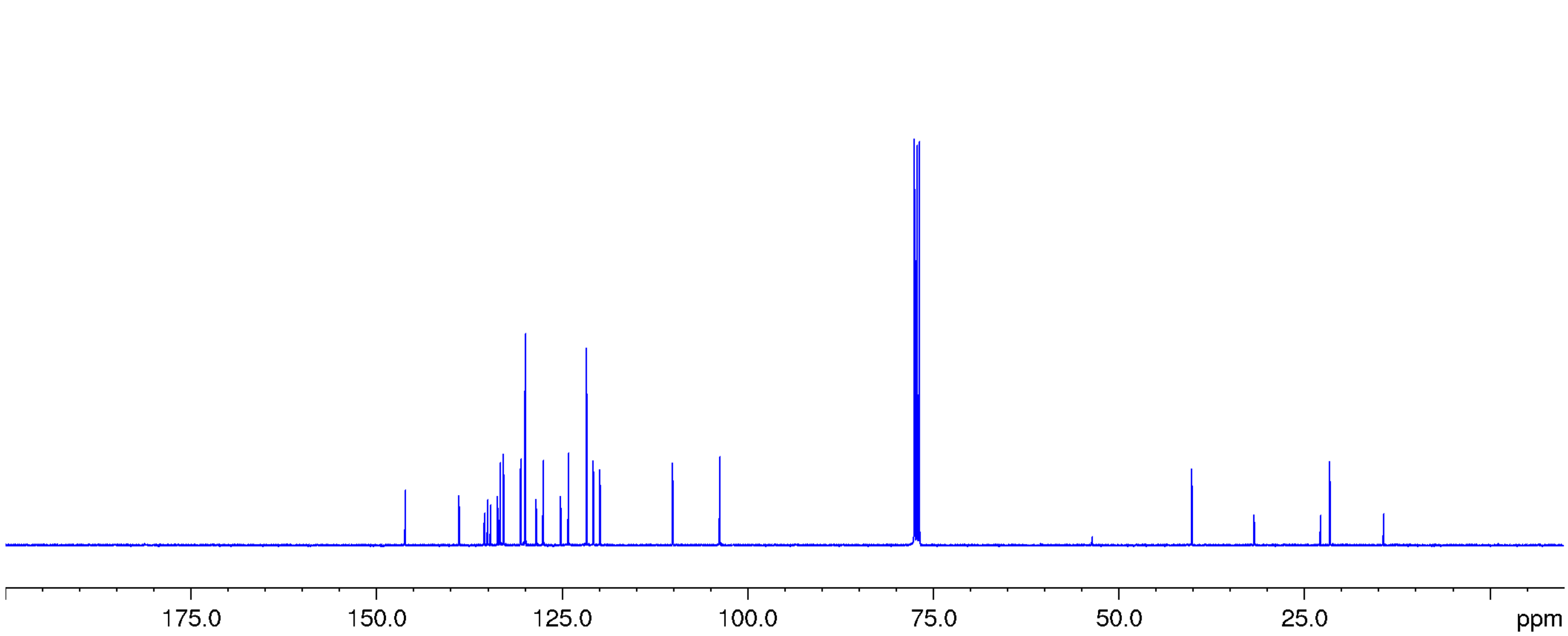

DEPT 135 NMR-spectrum ($CDCl_3$)

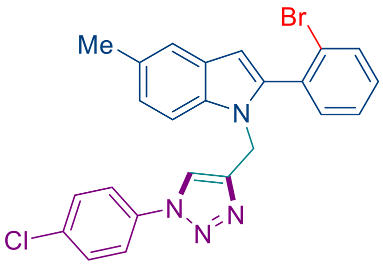

6k

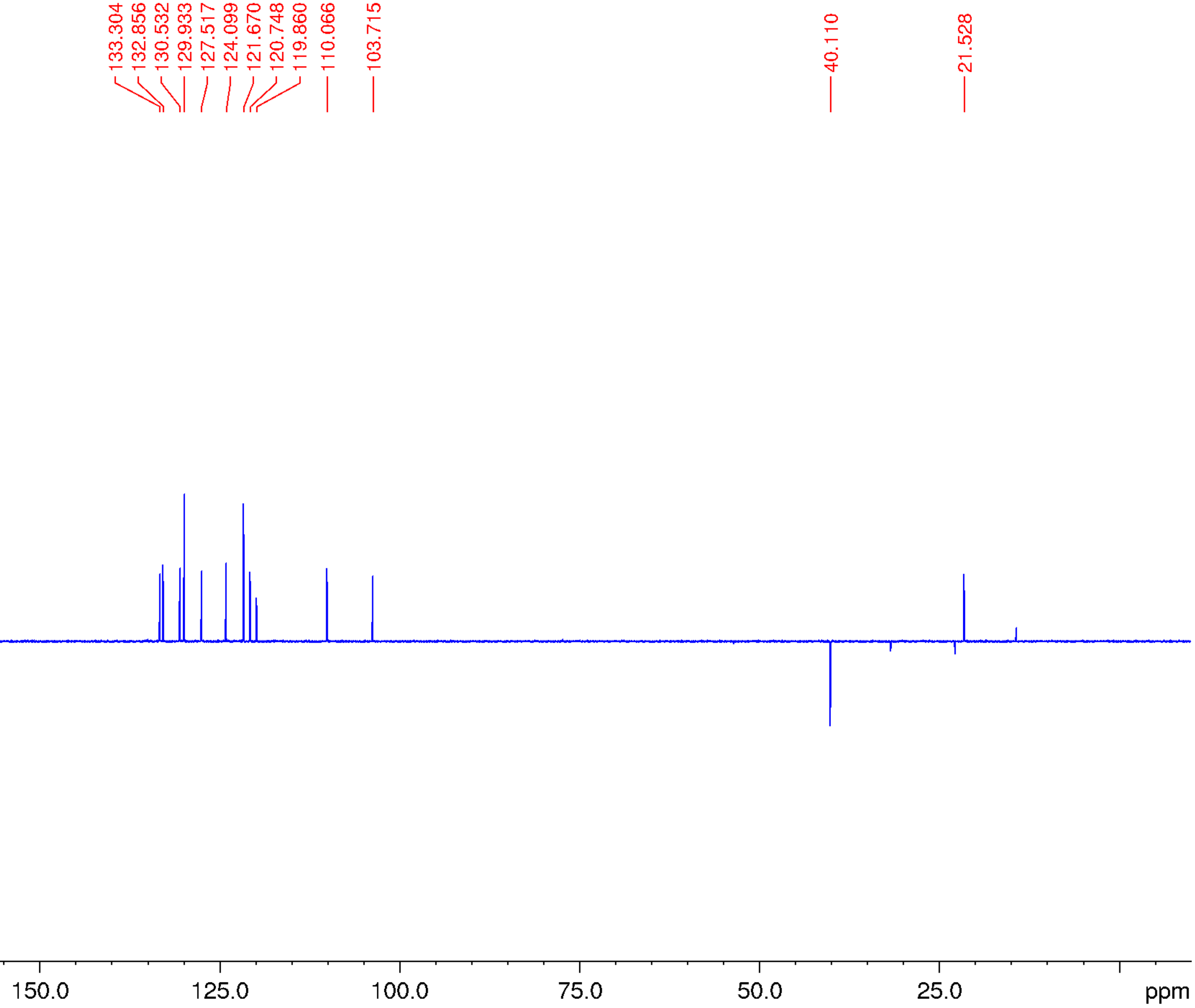

^{1}H NMR-spectrum (400 MHz, $CDCl_3$)

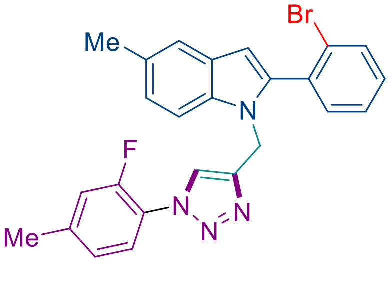

6l

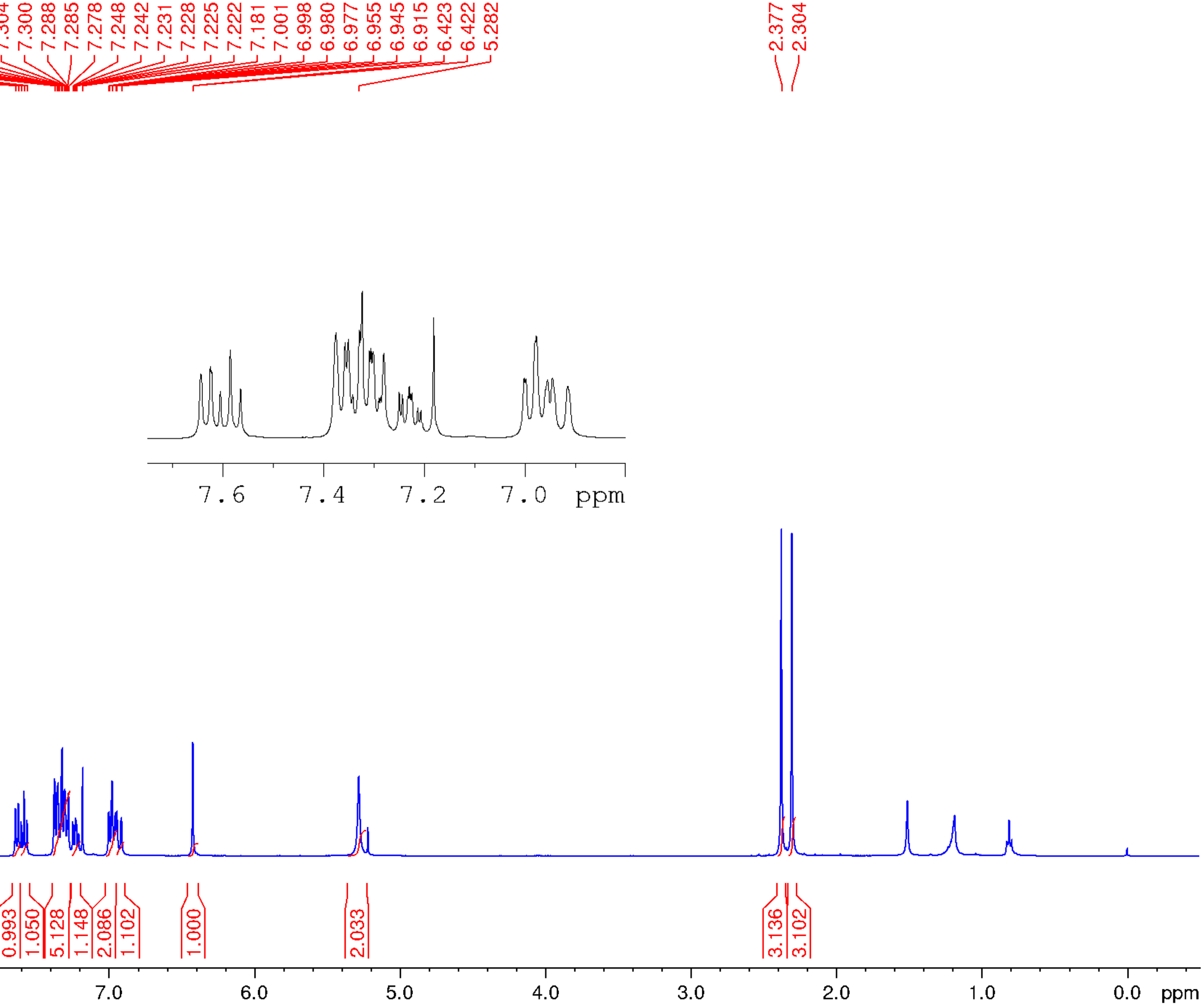

^{13}C NMR-spectrum (100 MHz, $CDCl_3$)

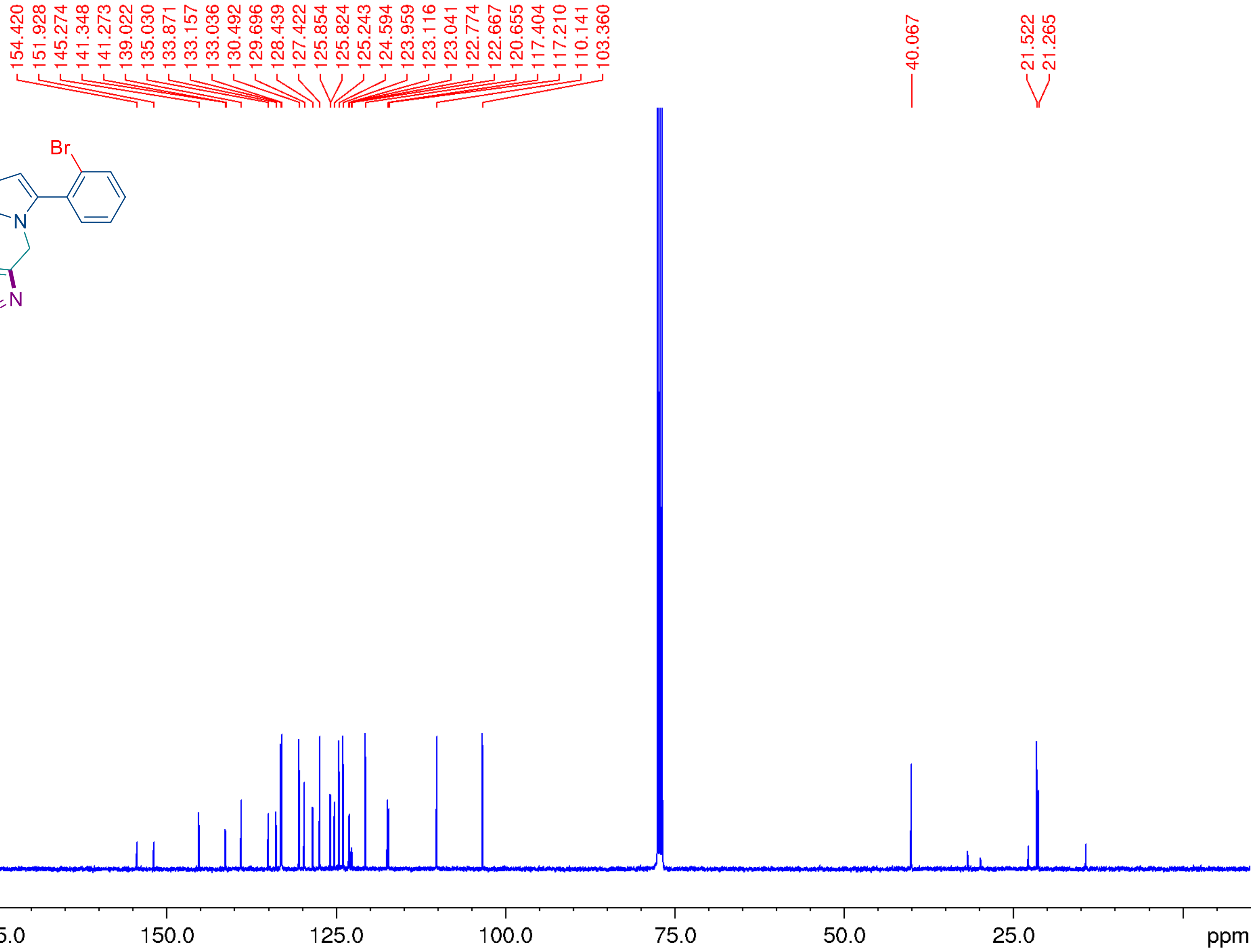

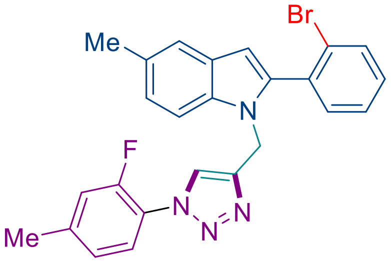

6l

DEPT 135 NMR-spectrum ($CDCl_3$)

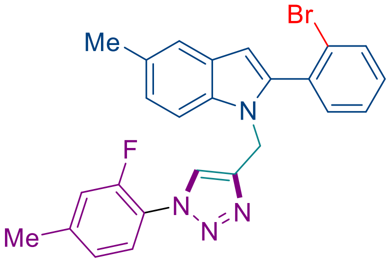

6l

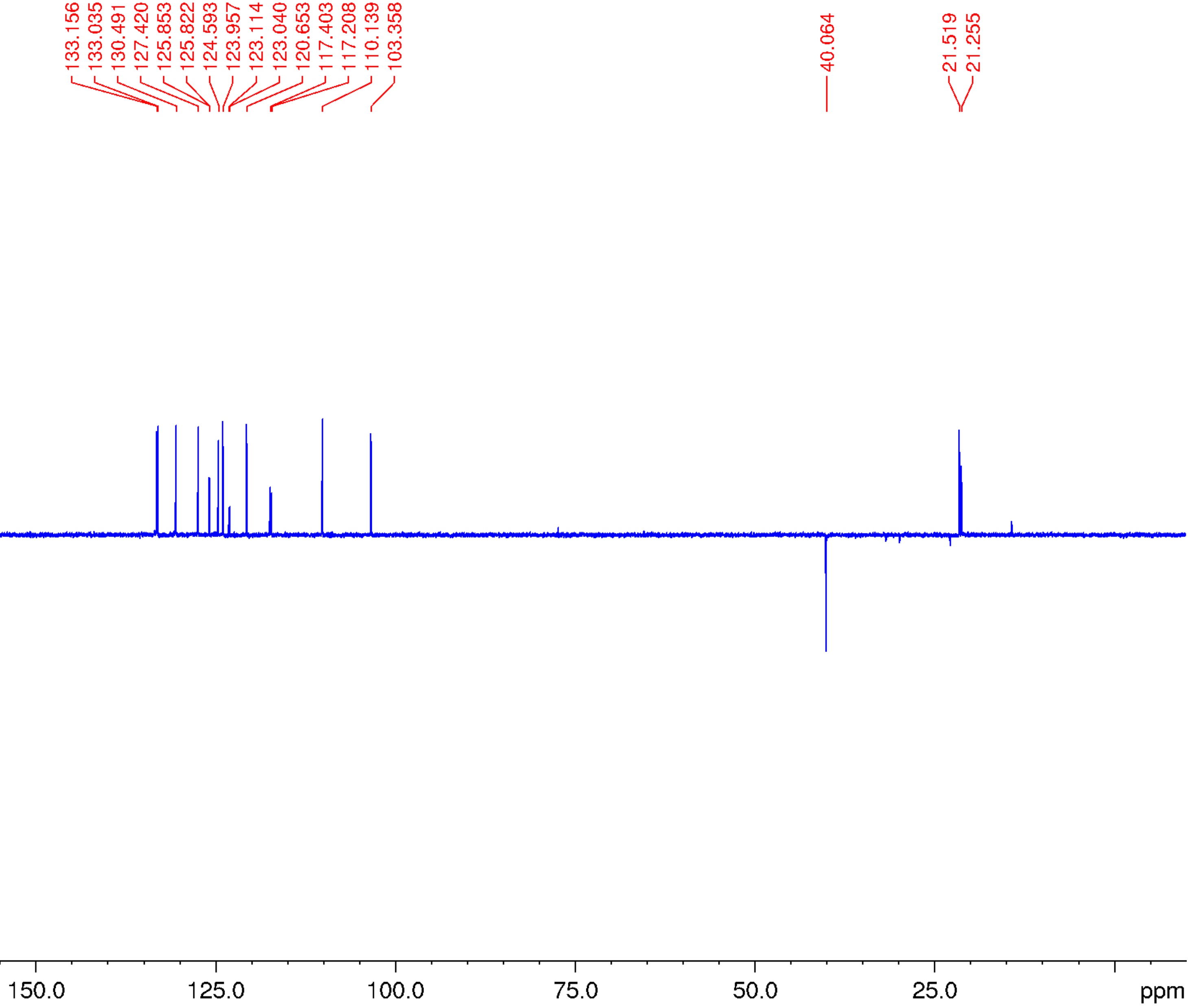

^{19}F NMR-spectrum (376.5 Hz, $CDCl_3$)

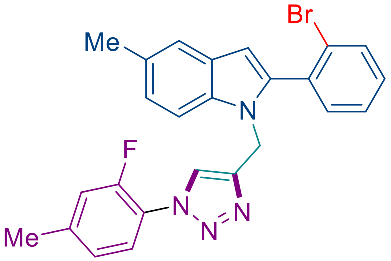

6l

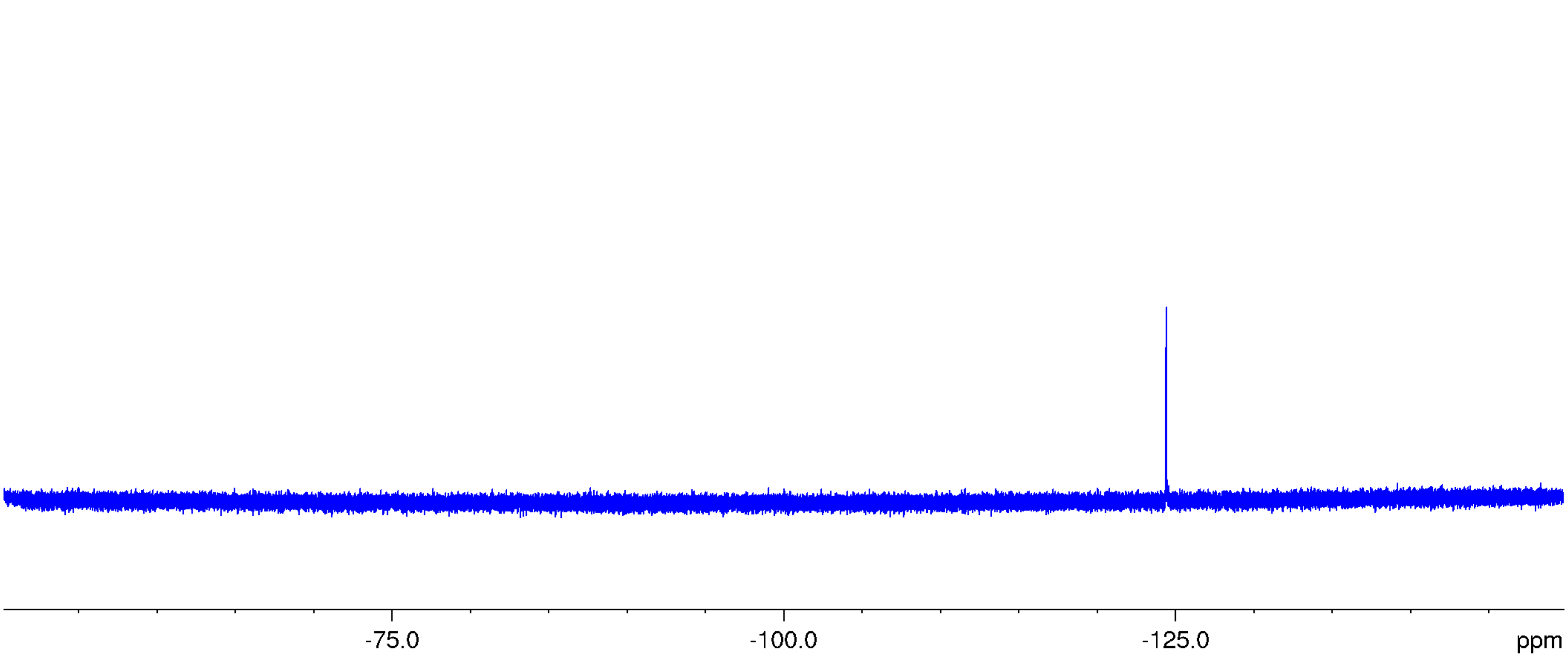

^1H NMR-spectrum (400 MHz, $CDCl_3$)

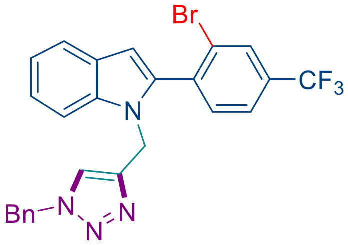

6m

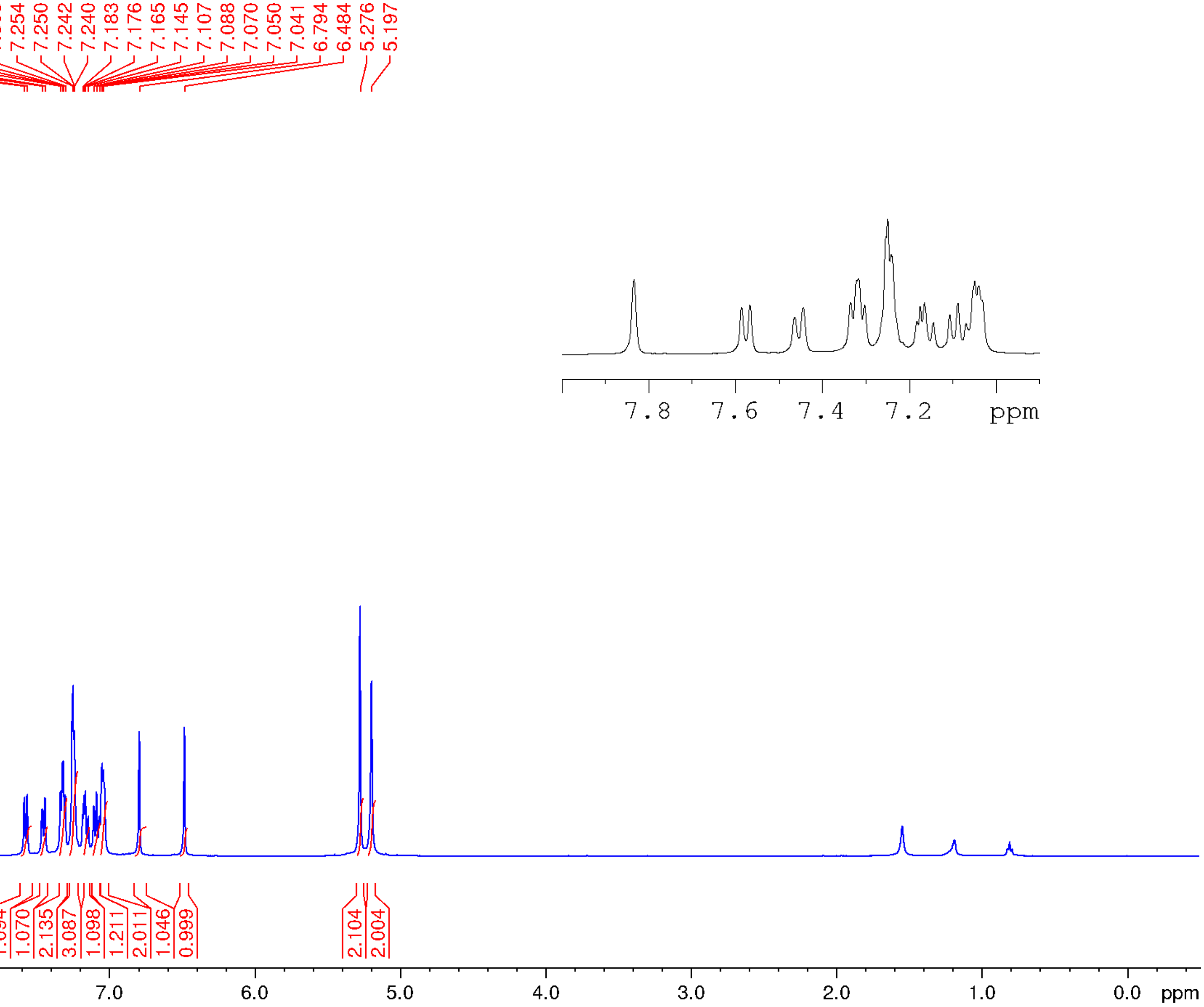

^{13}C NMR-spectrum (100 MHz, $CDCl_3$)

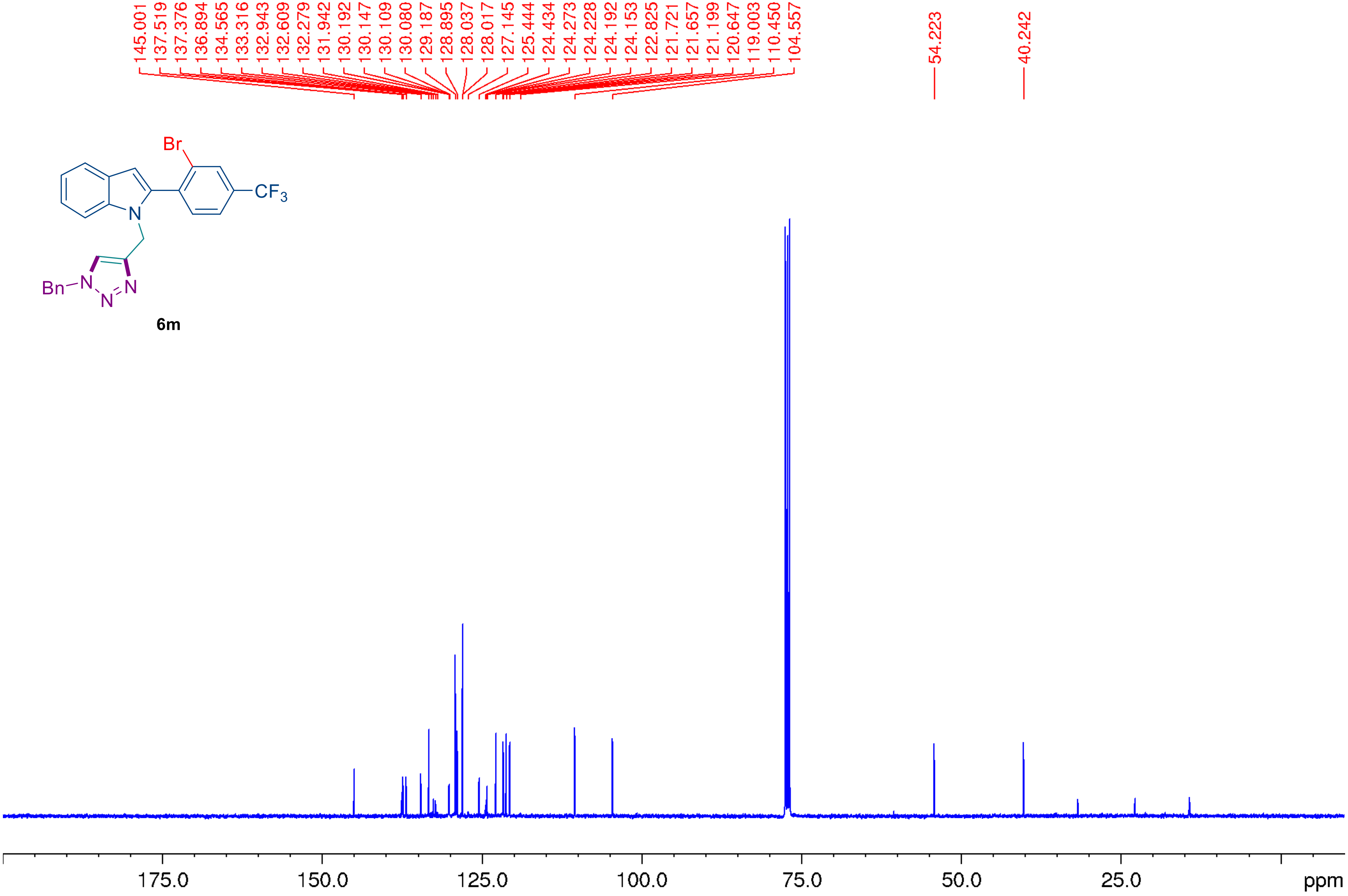

DEPT 135 NMR-spectrum ($CDCl_3$)

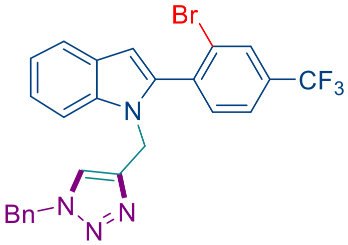

6m

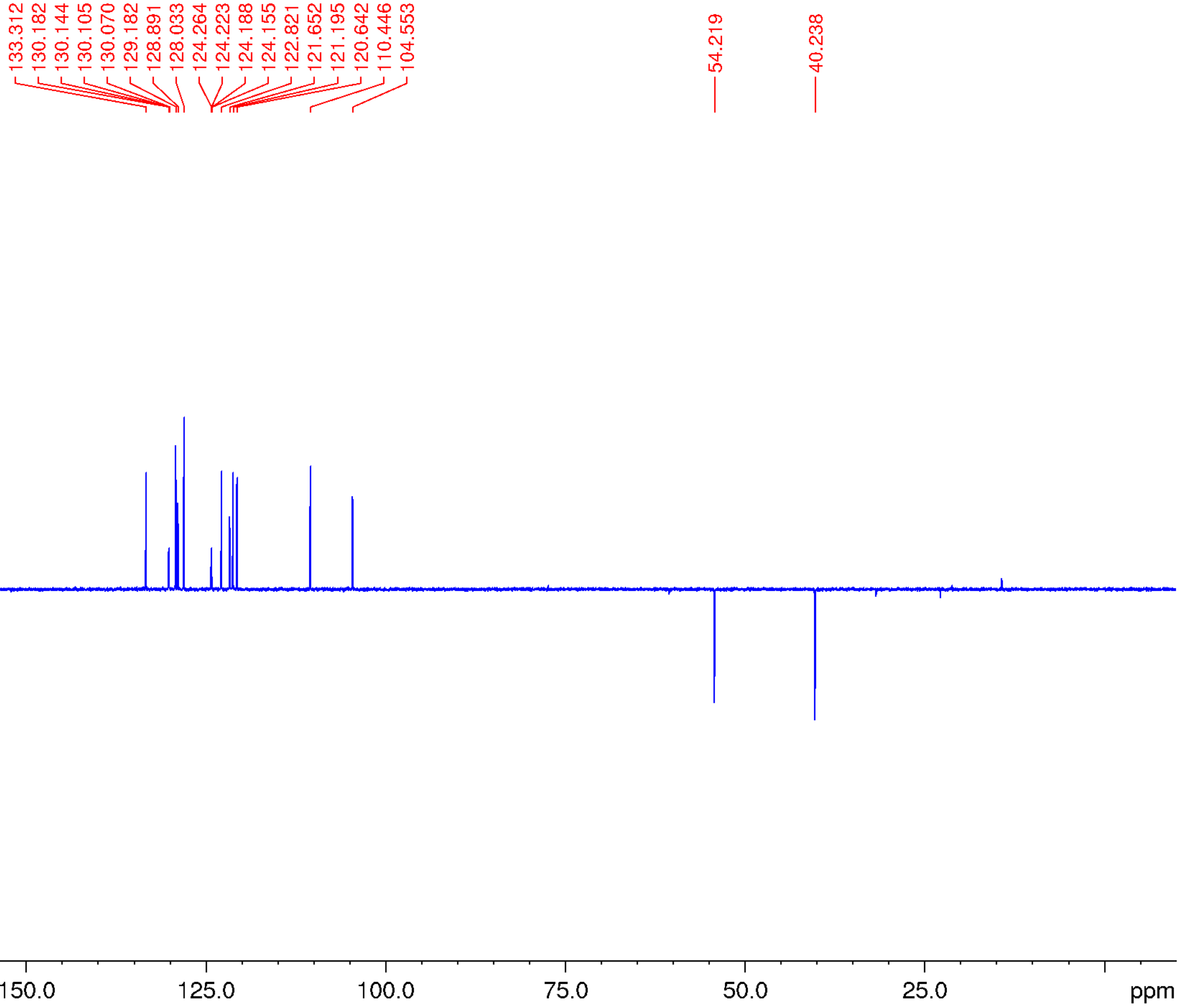

^{19}F NMR-spectrum (376.5 Hz, $CDCl_3$)

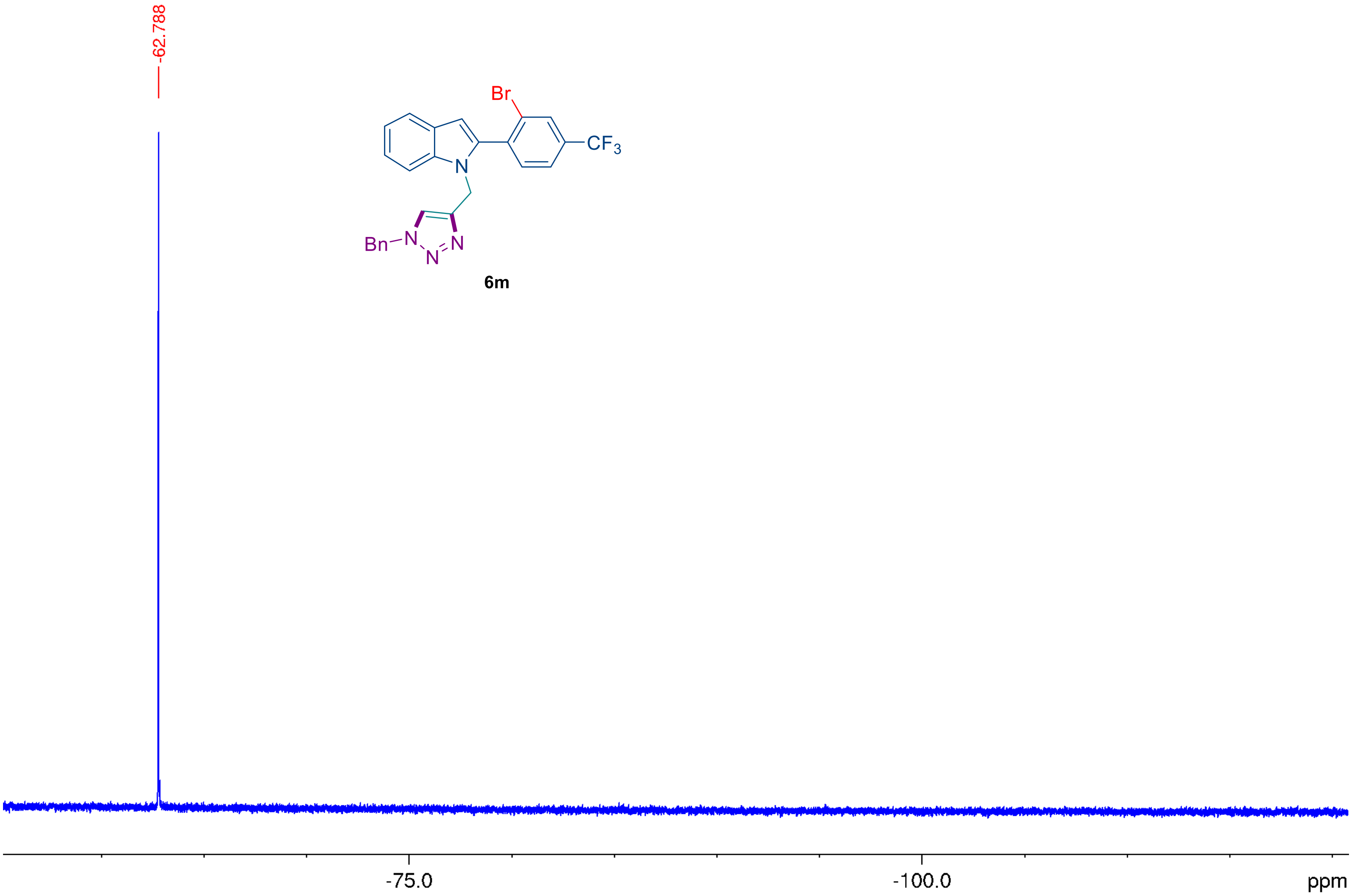

^{1}H NMR-spectrum (400 MHz, $CDCl_3$)

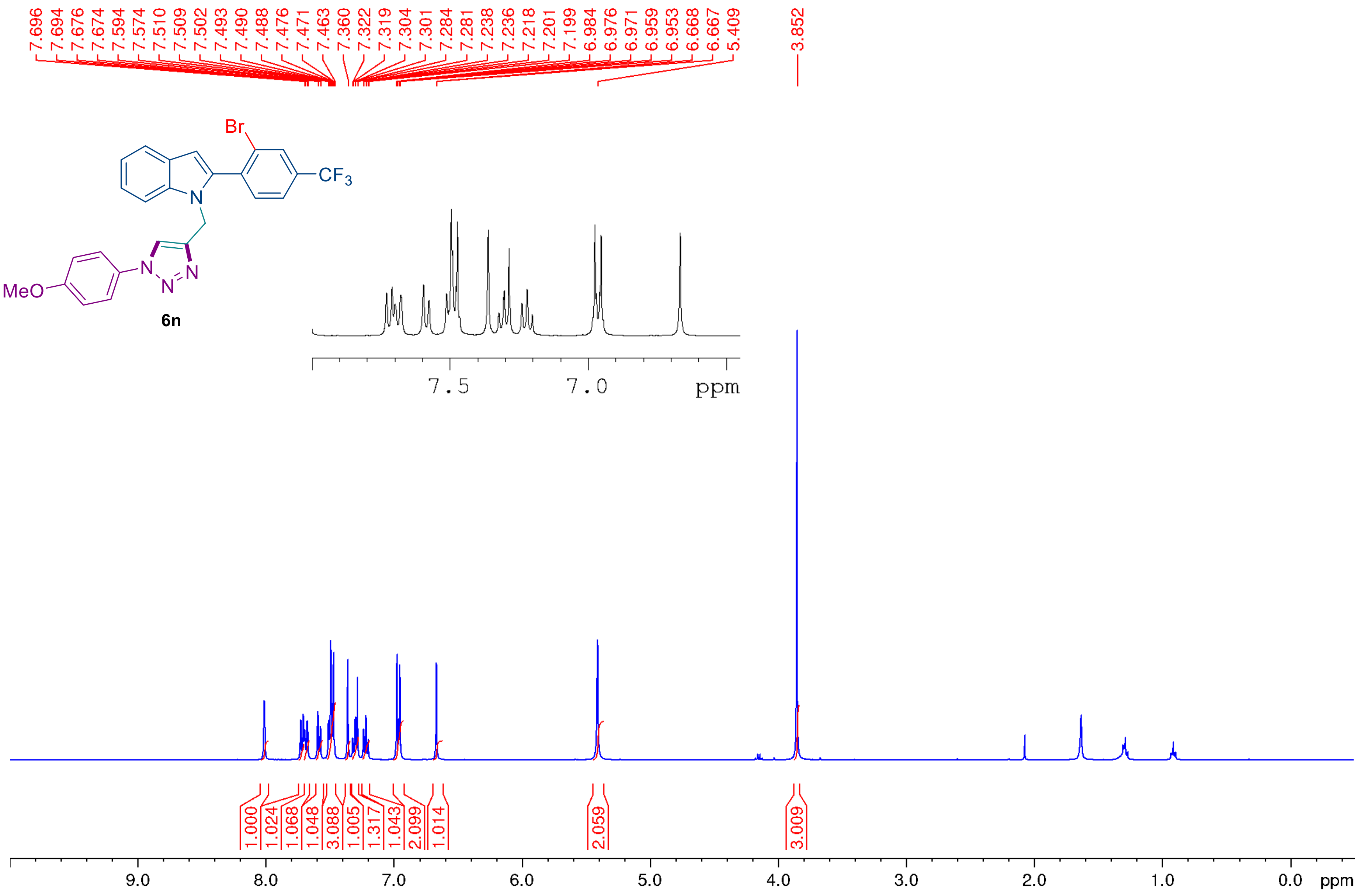

^{13}C NMR-spectrum (100 MHz, $CDCl_3$)

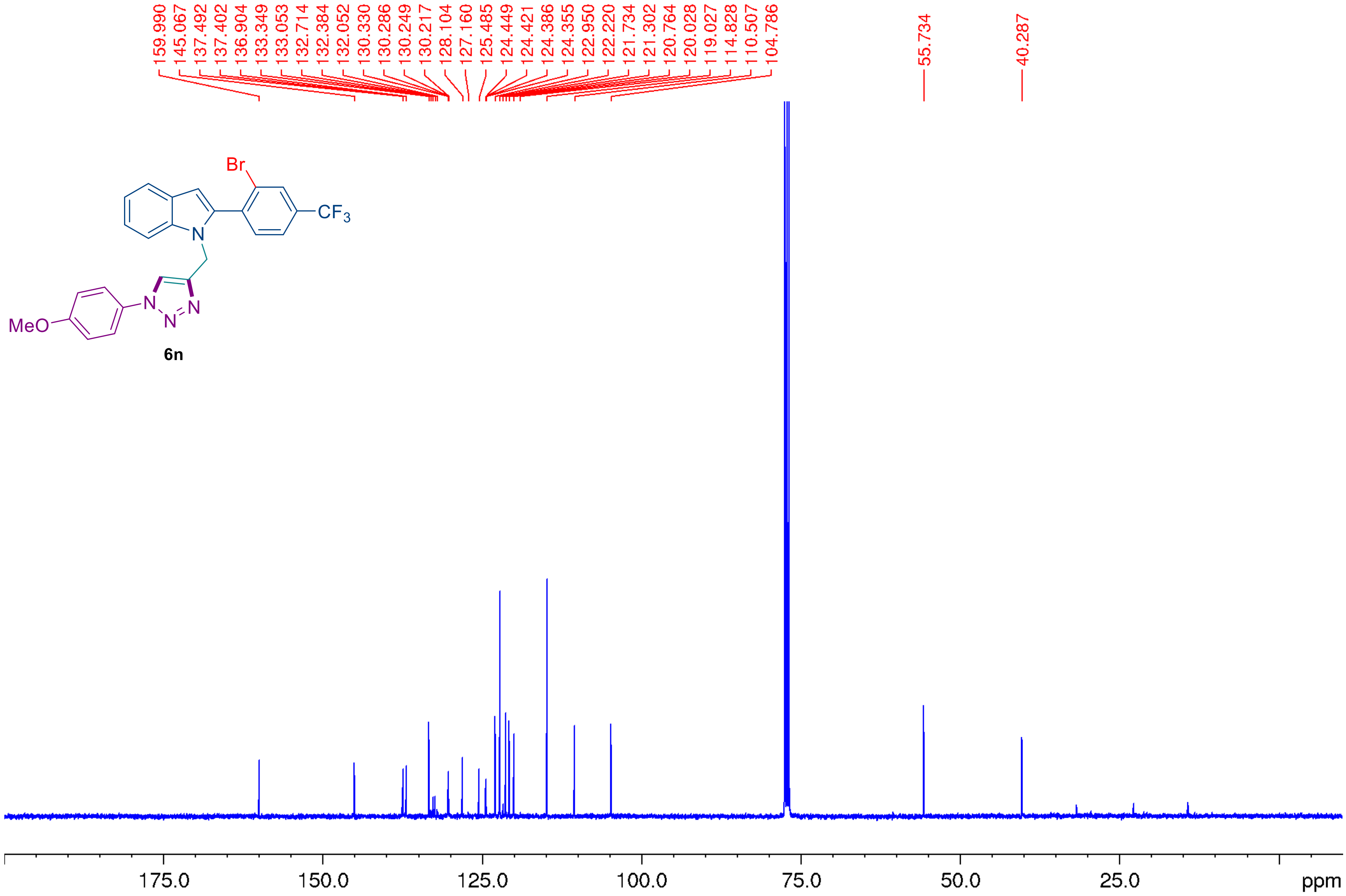

DEPT 135 NMR-spectrum ($CDCl_3$)

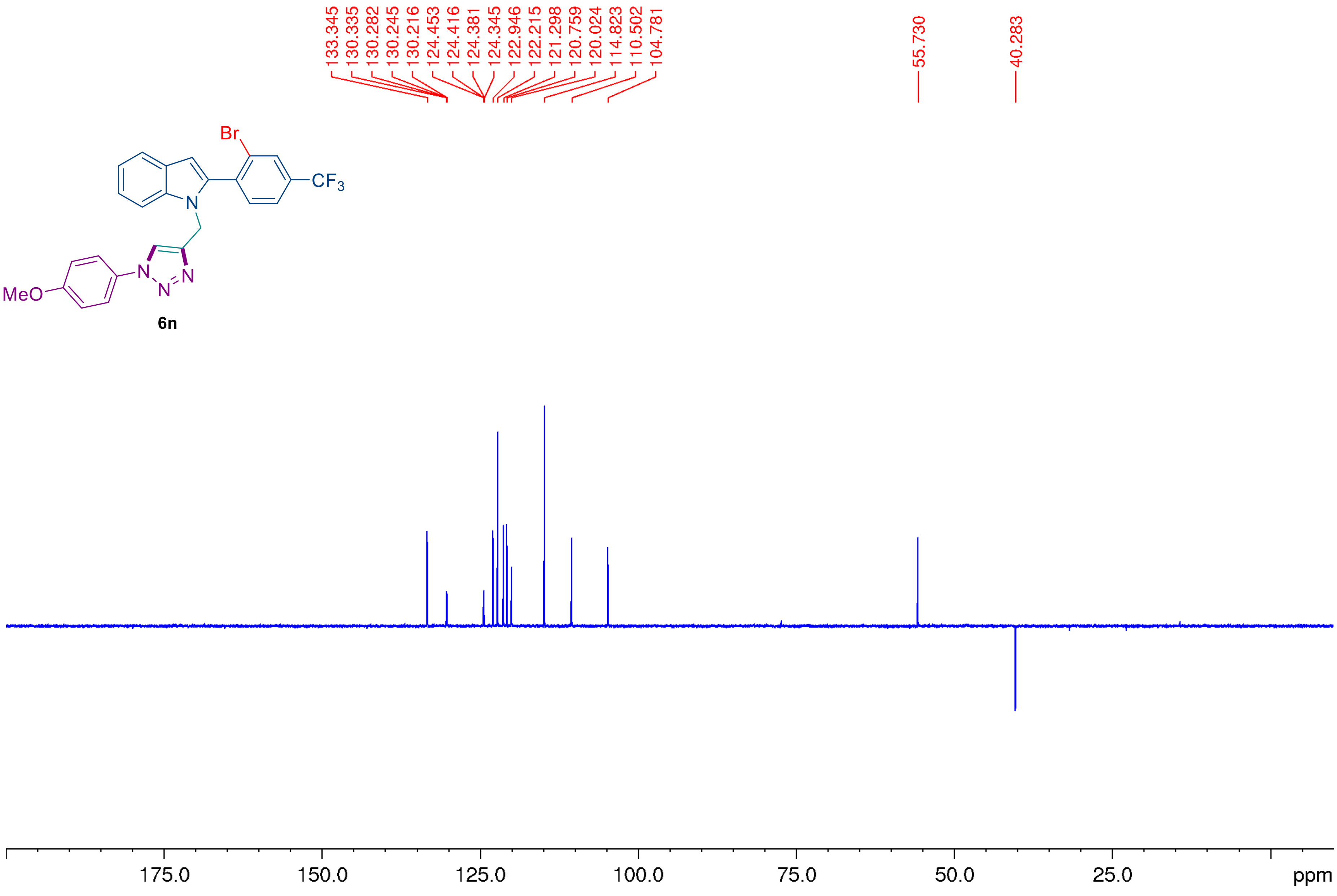

^{19}F NMR-spectrum (376.5 Hz, $CDCl_3$)

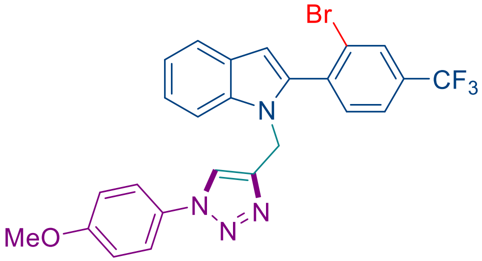

6n

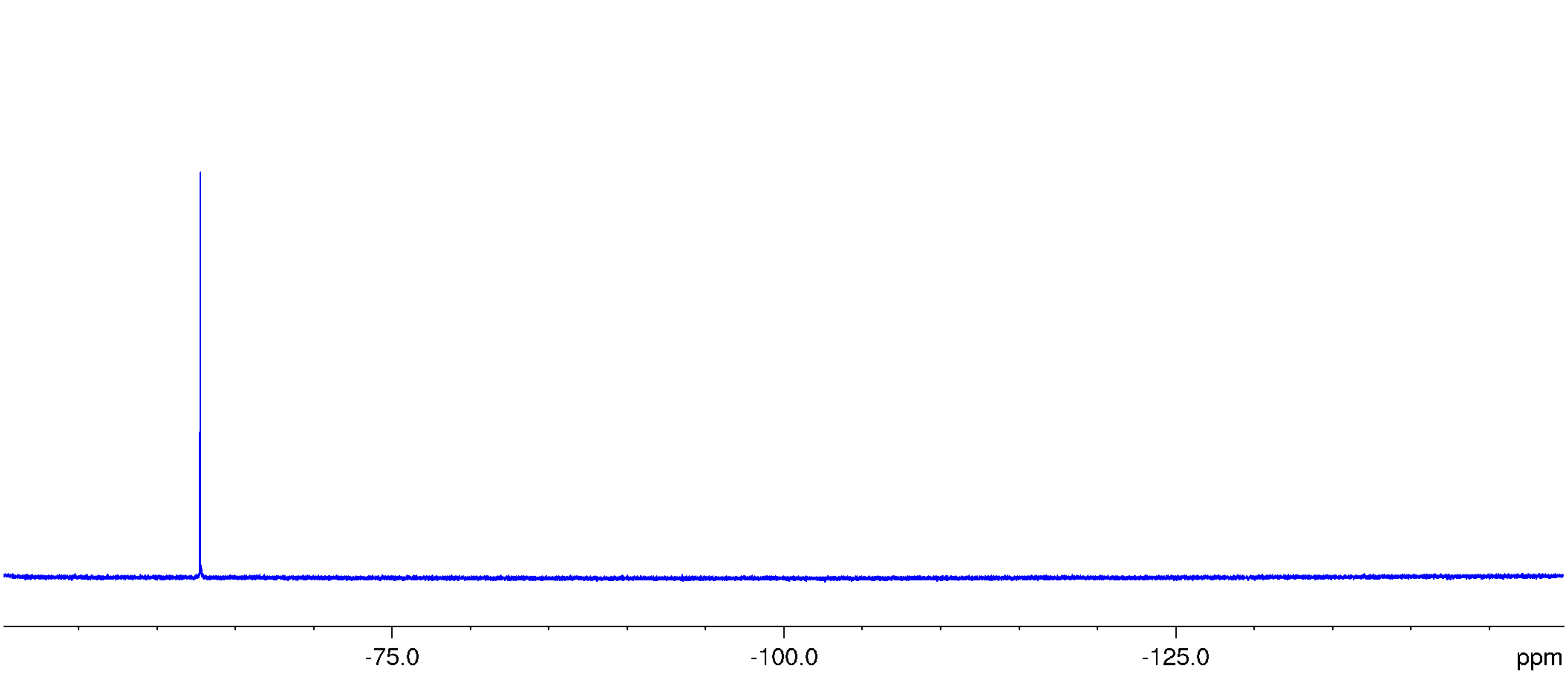

^{1}H NMR-spectrum (400 MHz, $CDCl_3$)

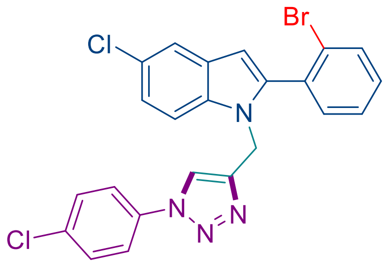

6o

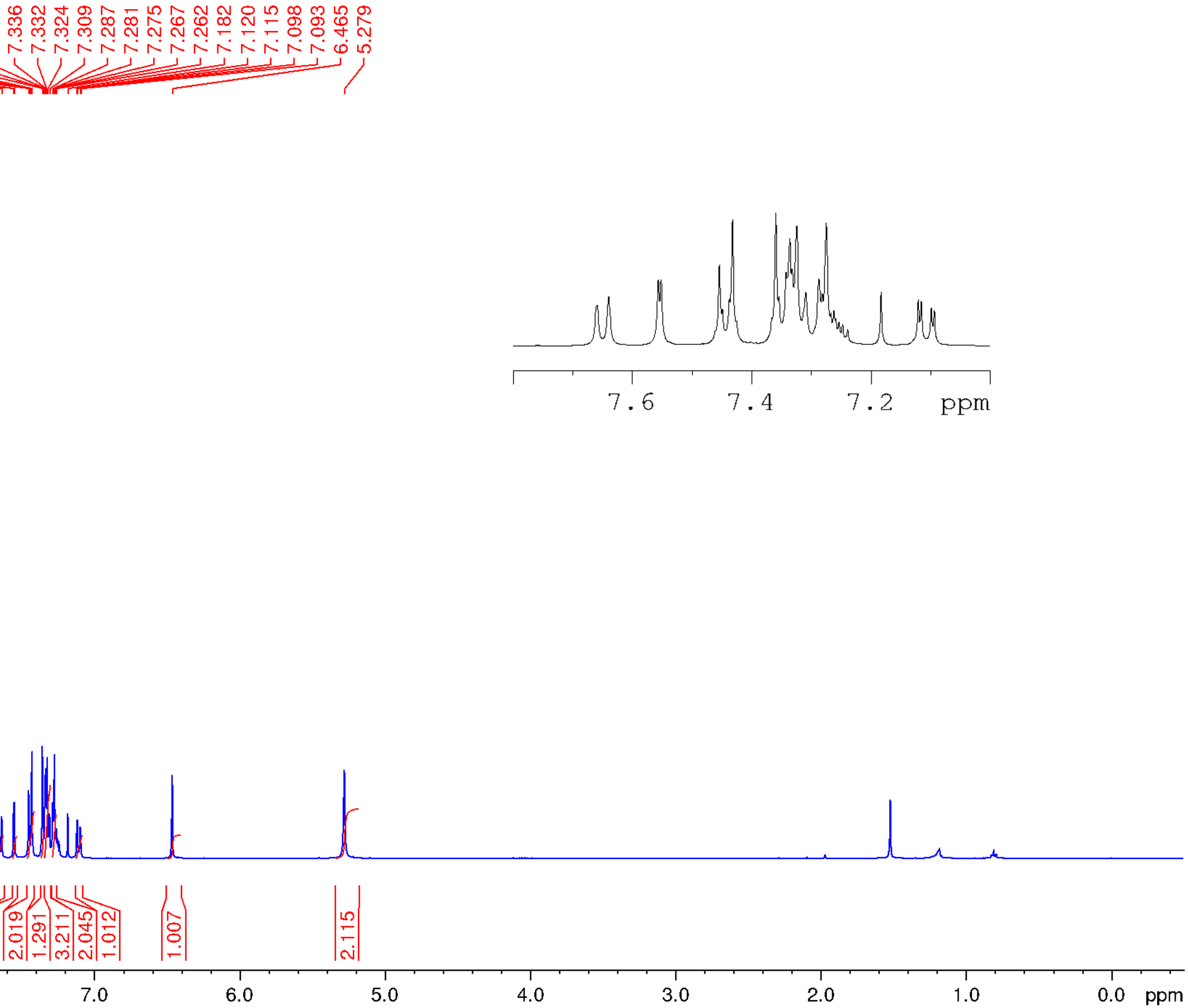

^{13}C NMR-spectrum (100 MHz, $CDCl_3$)

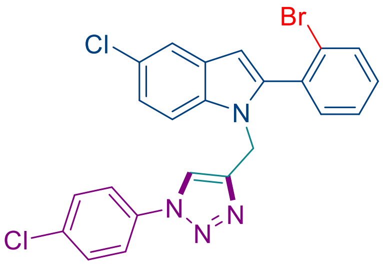

6o

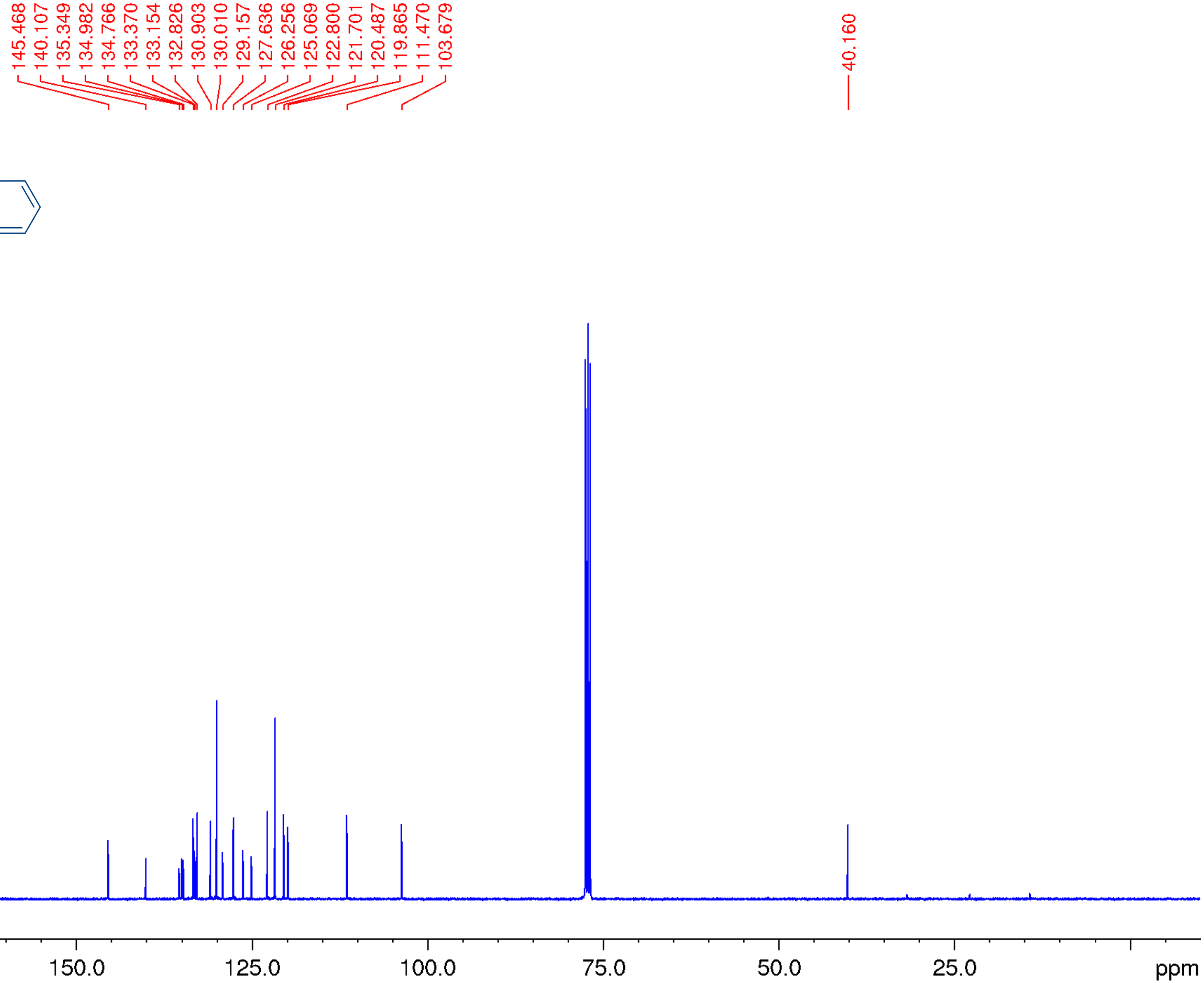

DEPT 135 NMR-spectrum ($CDCl_3$)

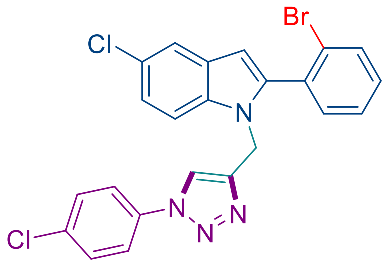

6o

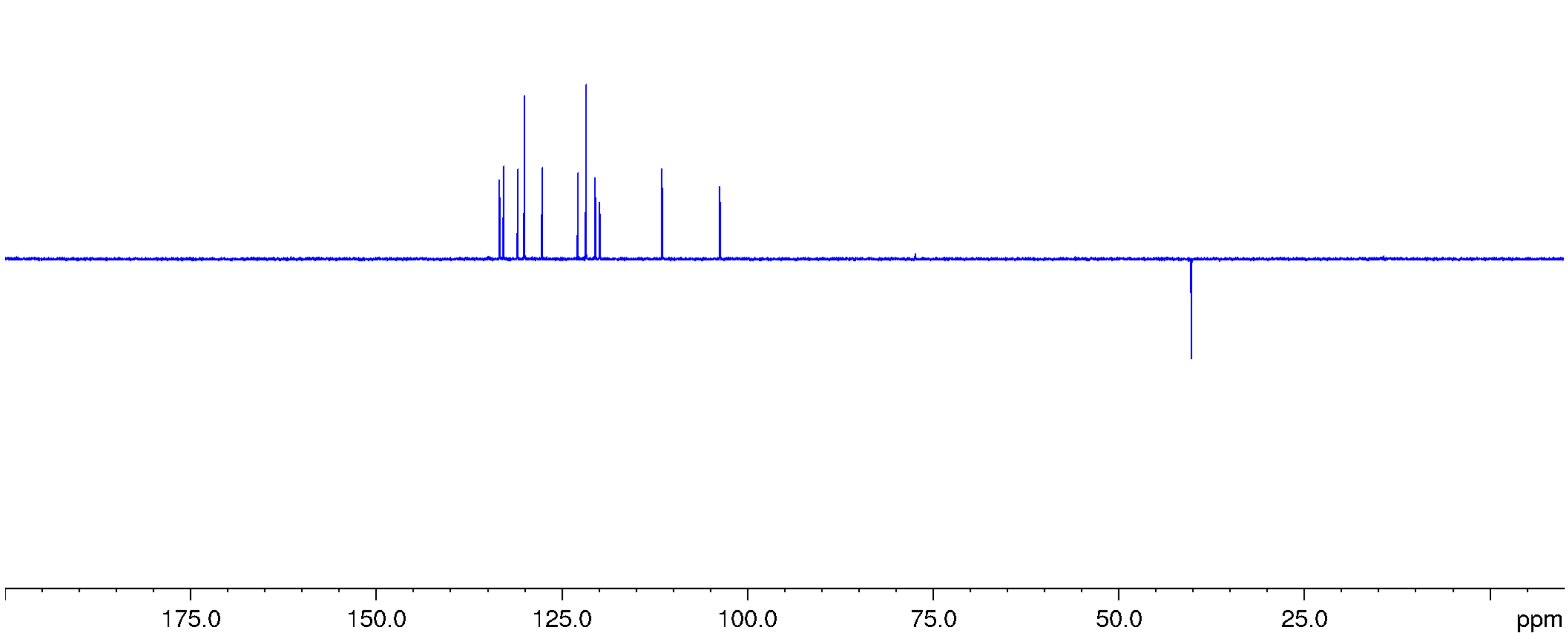

^{1}H NMR-spectrum (400 MHz, $CDCl_3$)

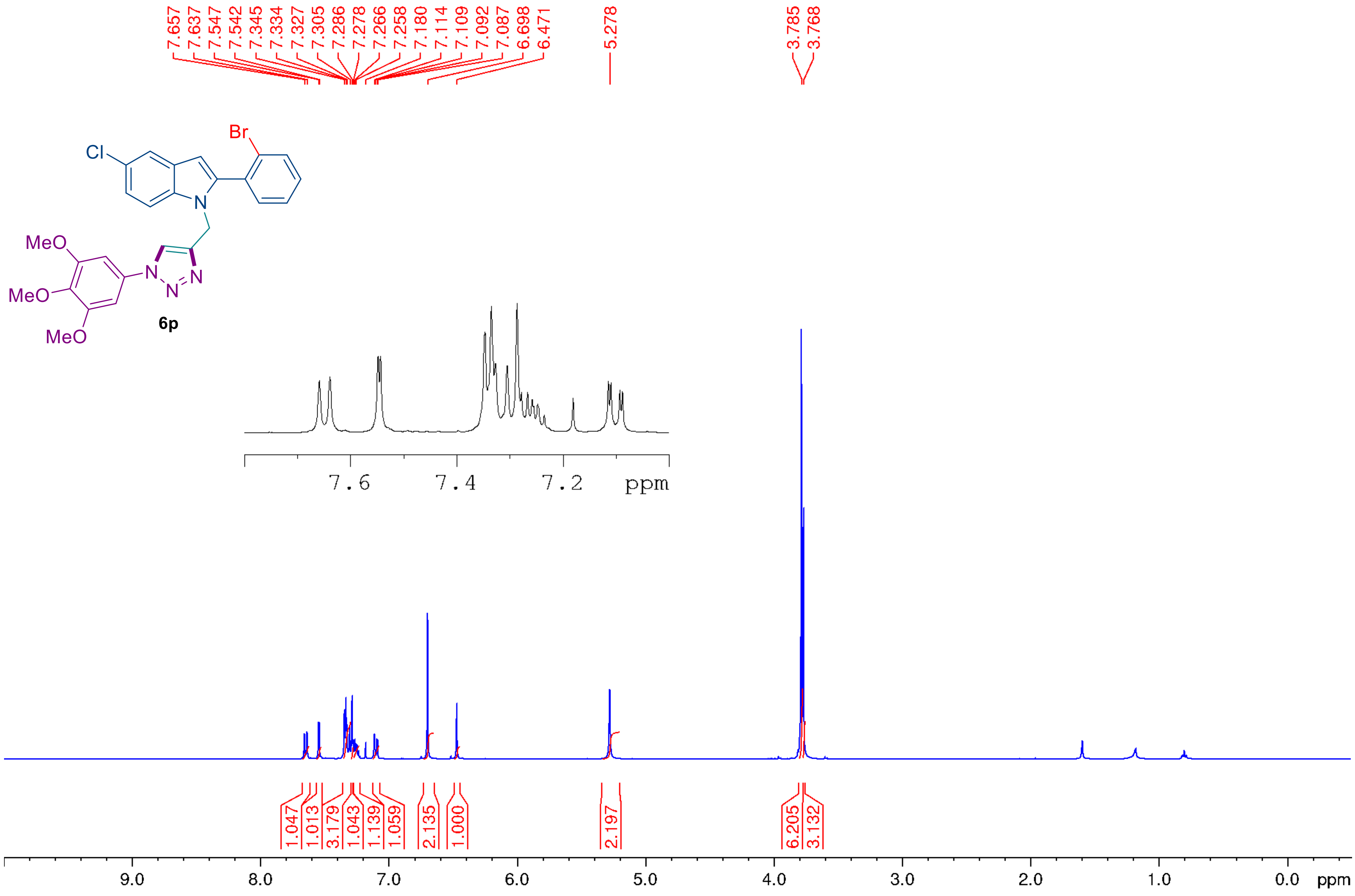

^{13}C NMR-spectrum (100 MHz, $CDCl_3$)

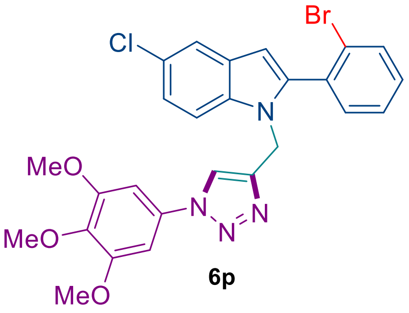

6p

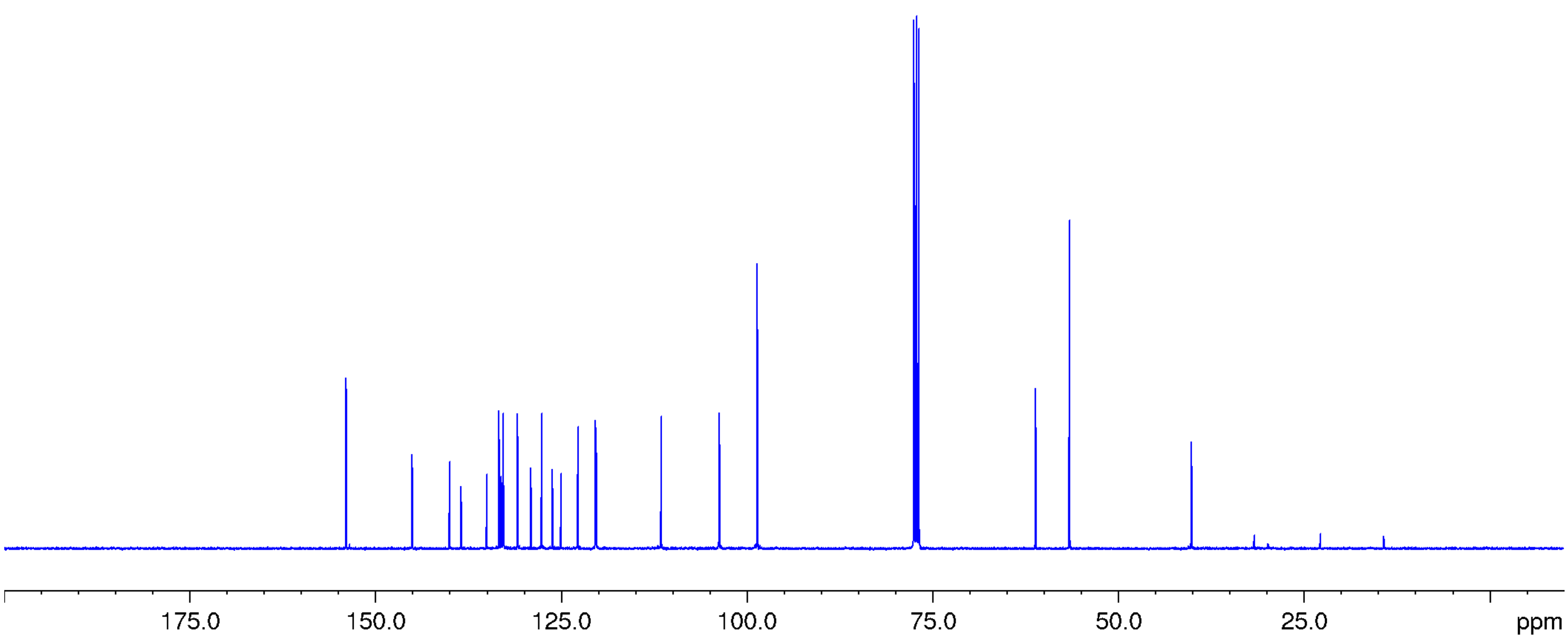

DEPT 135 NMR-spectrum ($CDCl_3$)

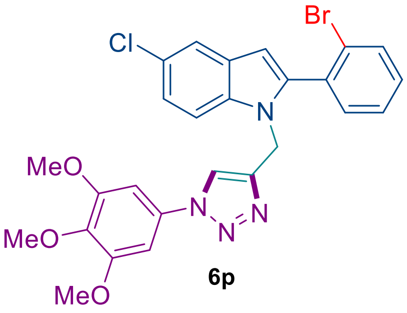

6p

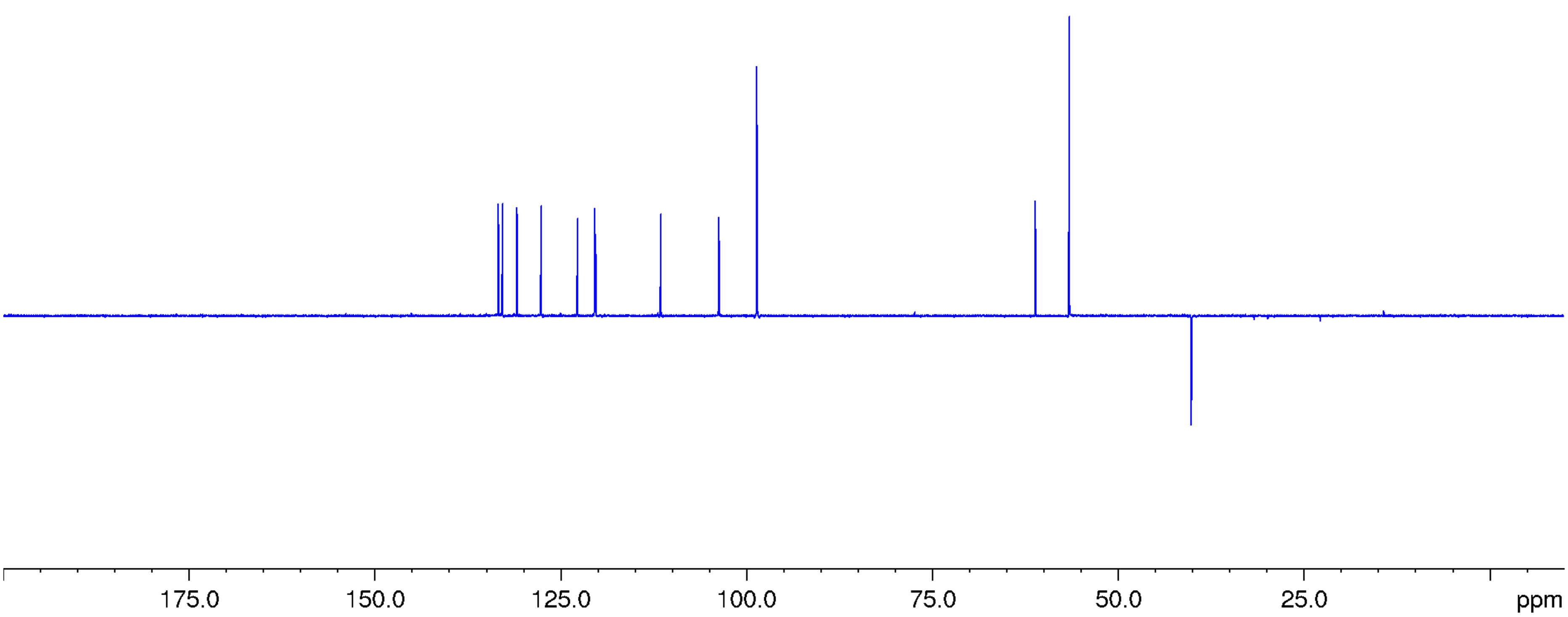

^{1}H NMR-spectrum (400 MHz, $CDCl_3$)

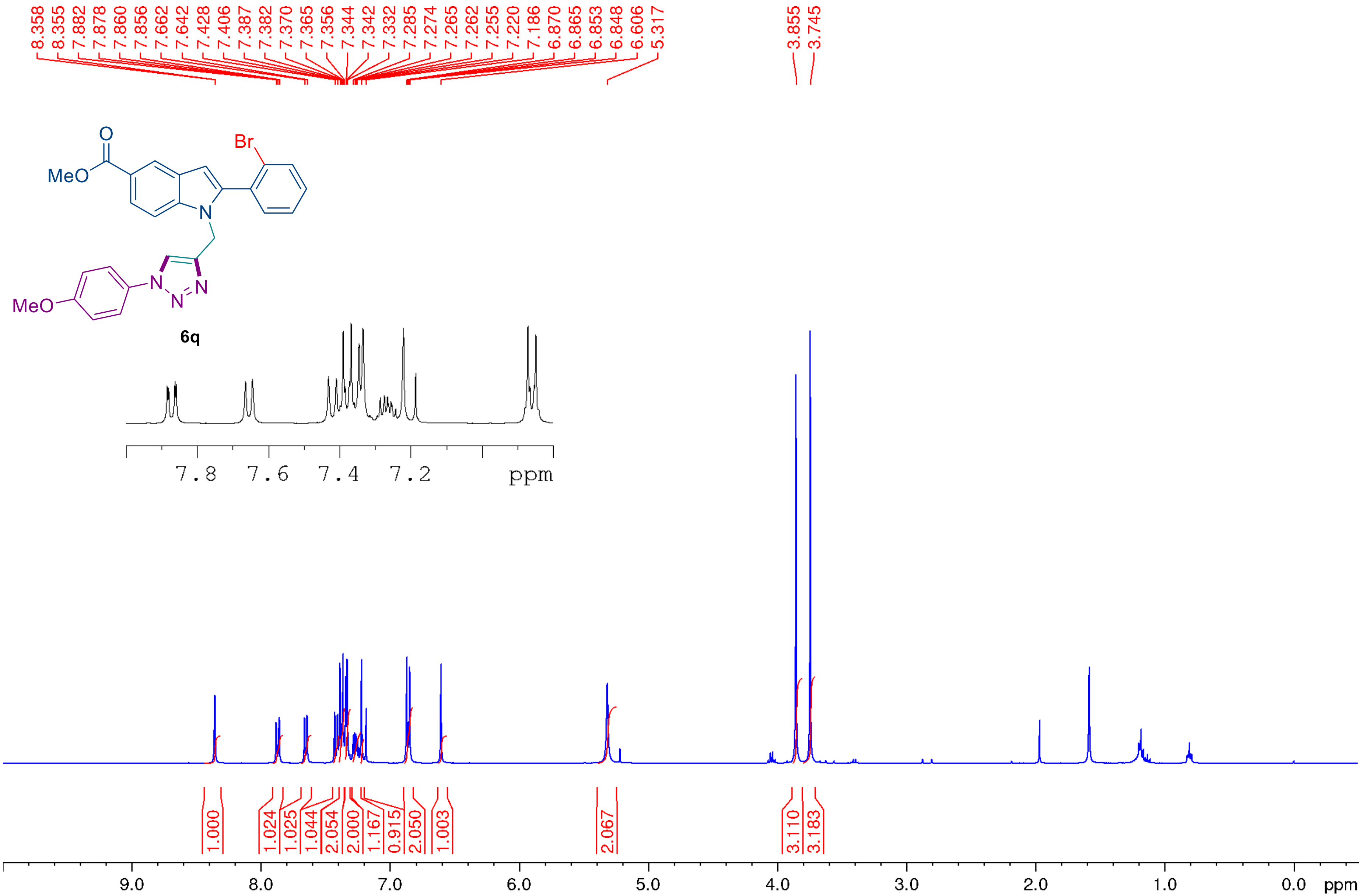

^{13}C NMR-spectrum (100 MHz, $CDCl_3$)

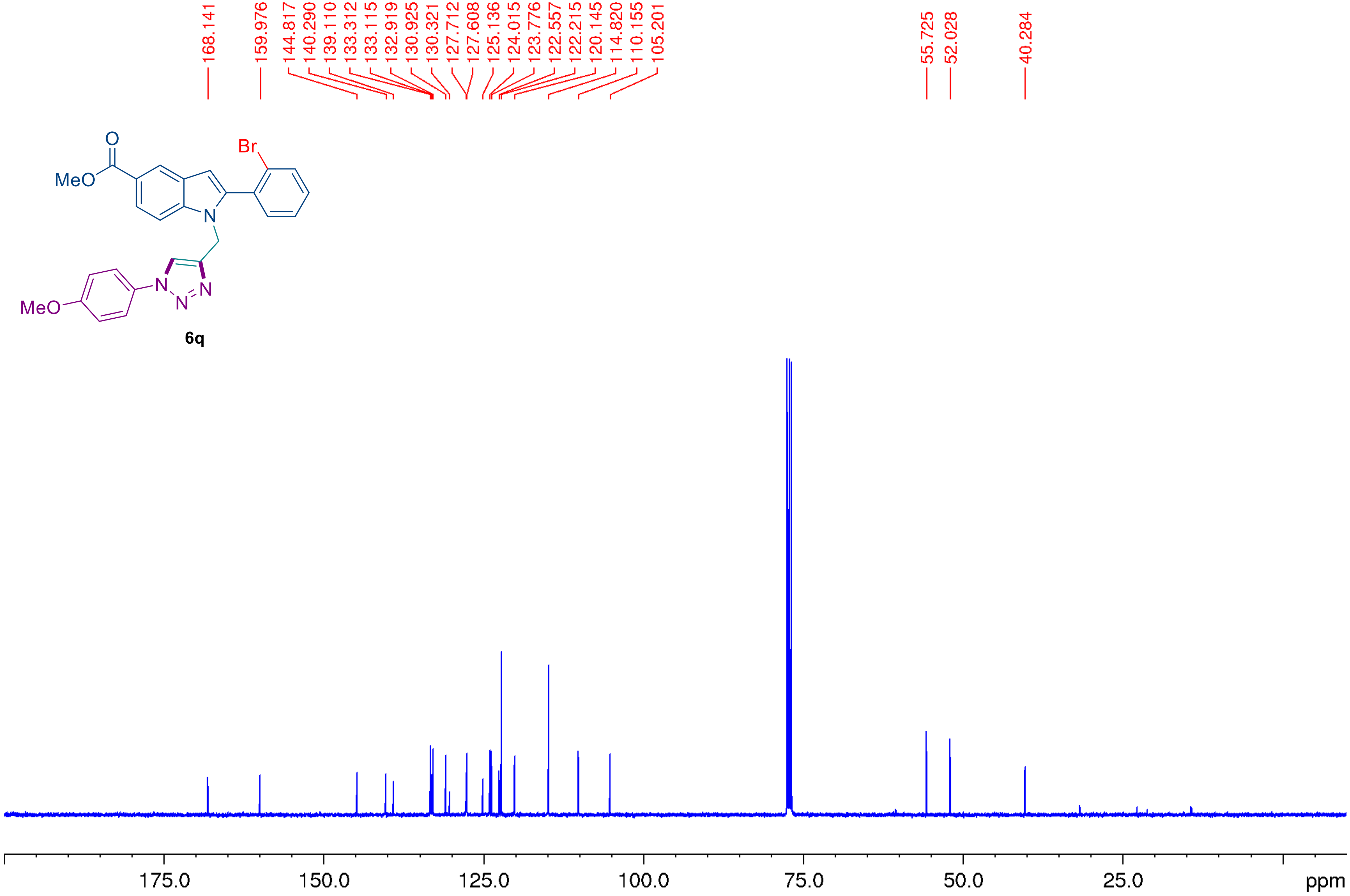

6q

DEPT 135 NMR-spectrum ($CDCl_3$)

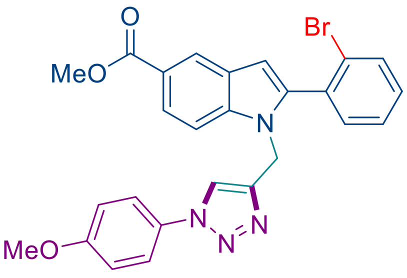

6q

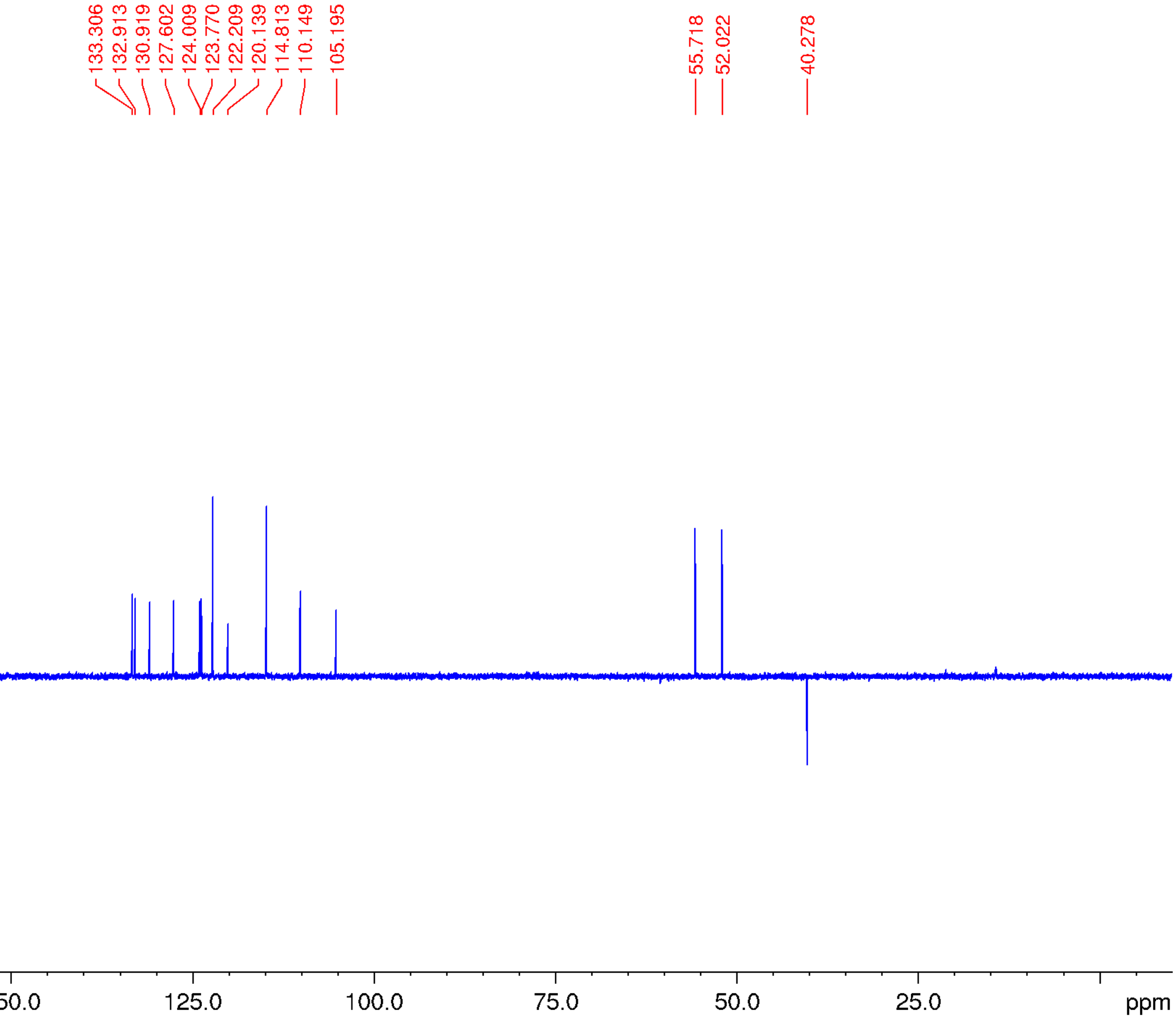

^{1}H NMR-spectrum (400 MHz, $CDCl_3$)

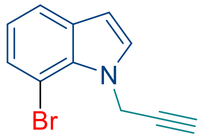

7a

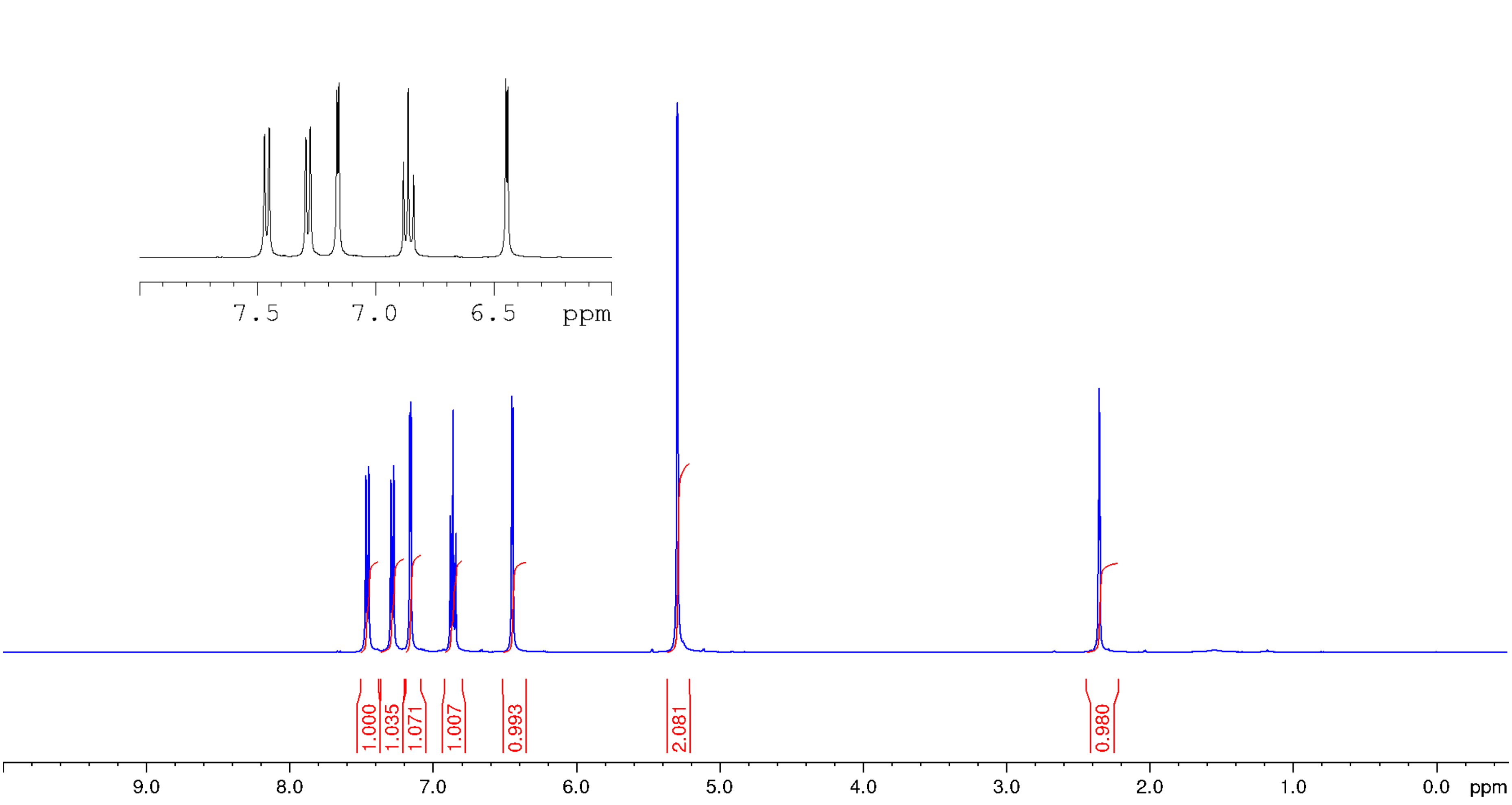

^{13}C NMR-spectrum (100 MHz, $CDCl_3$)

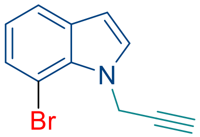

7a

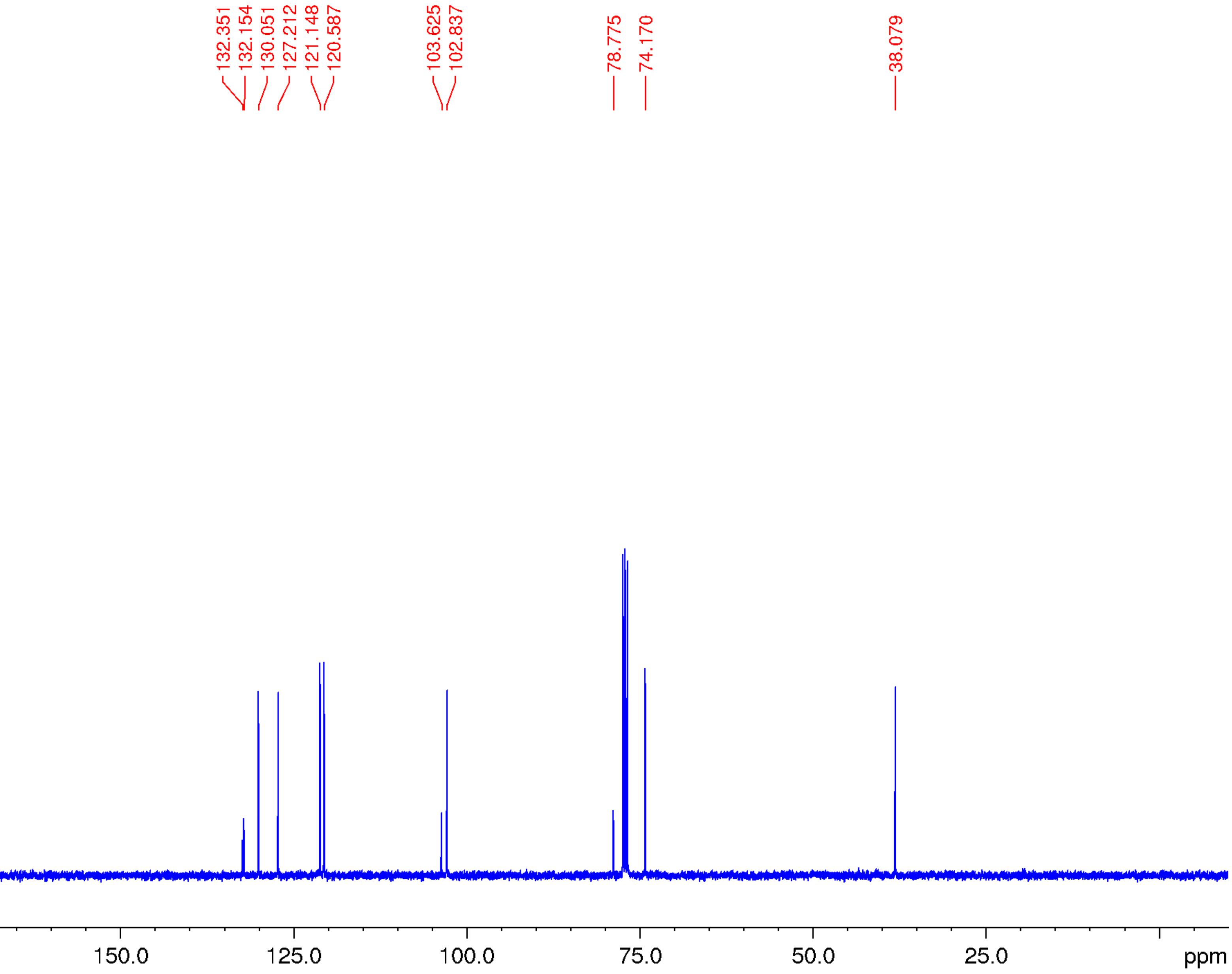

DEPT 135 NMR-spectrum ($CDCl_3$)

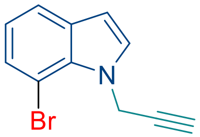

7a

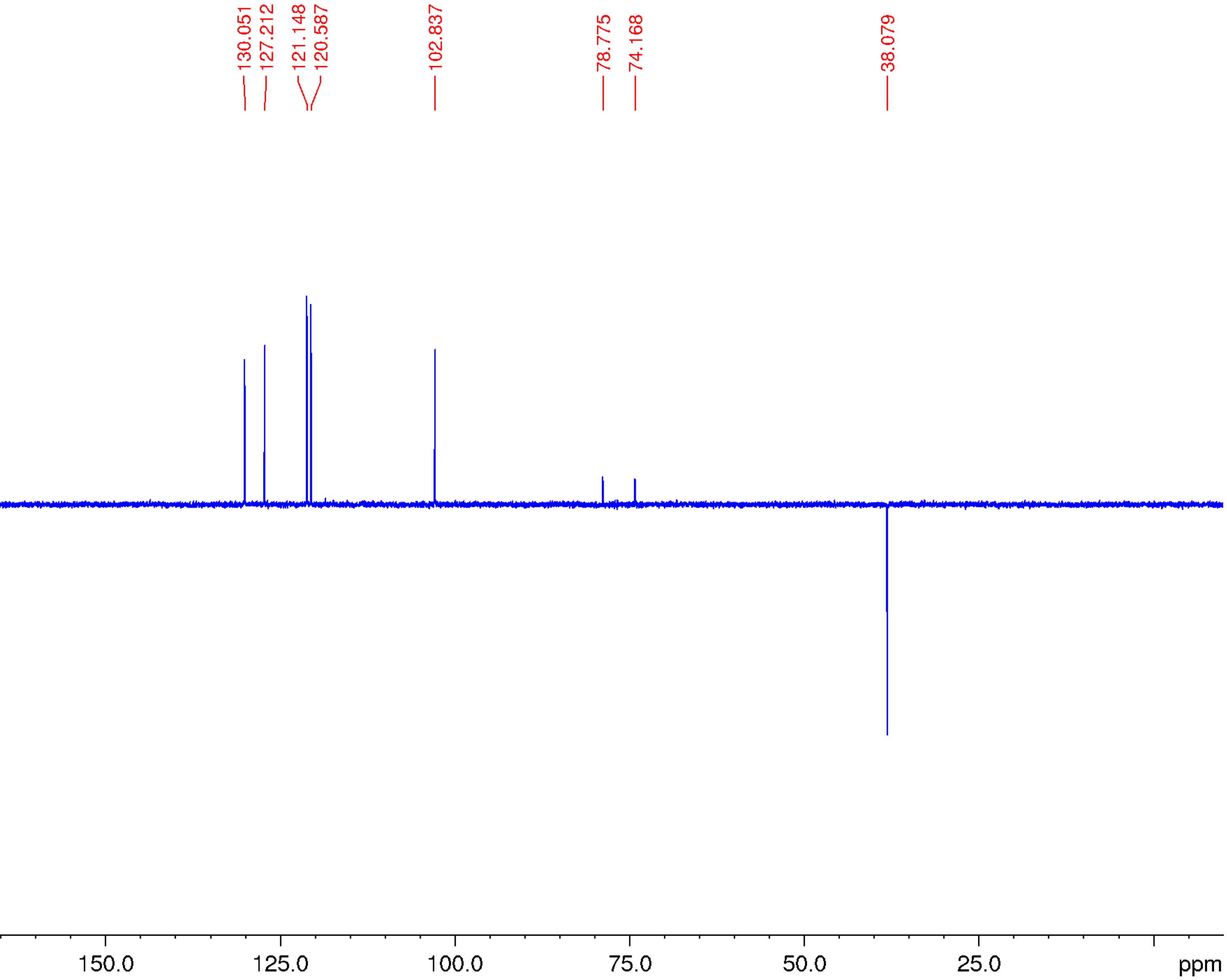

^{1}H NMR-spectrum (400 MHz, $CDCl_3$)

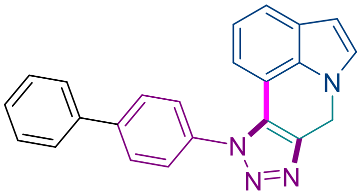

21a

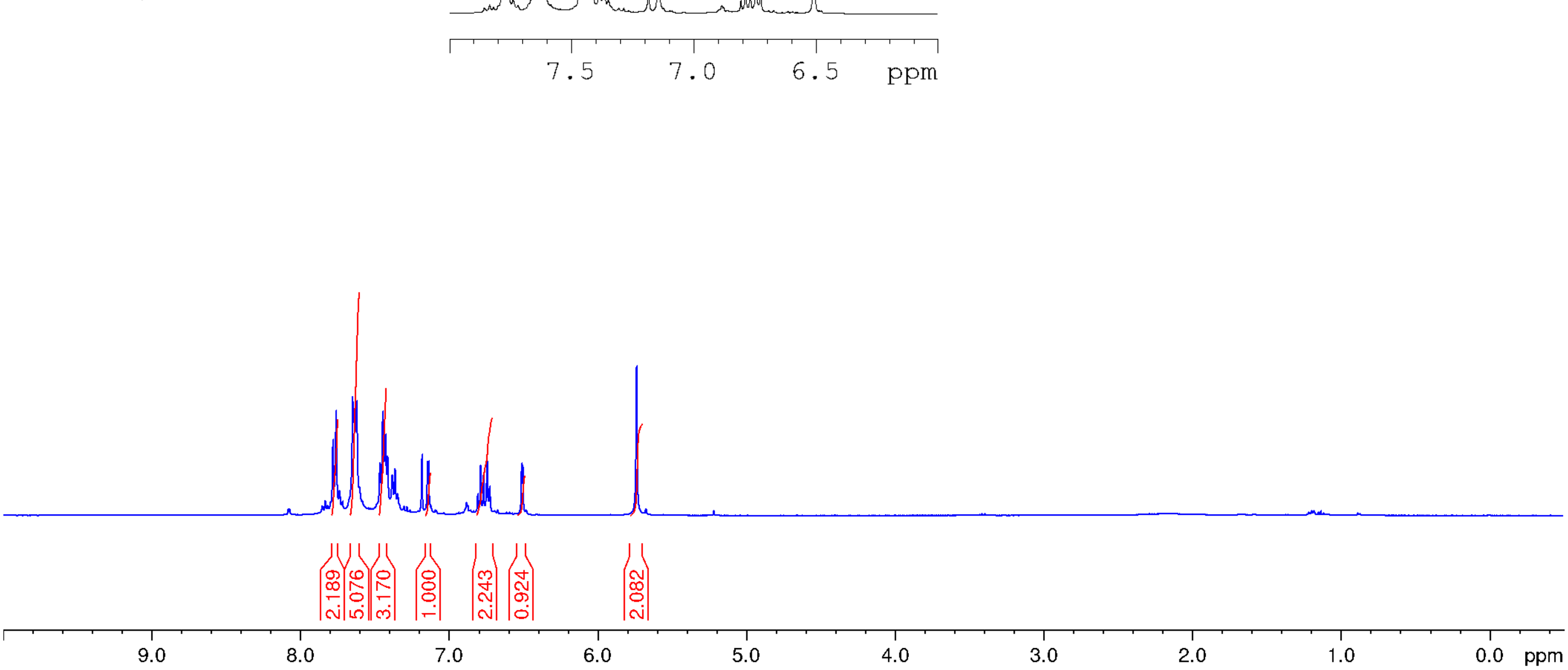

^{13}C NMR-spectrum (100 MHz, $CDCl_3$)

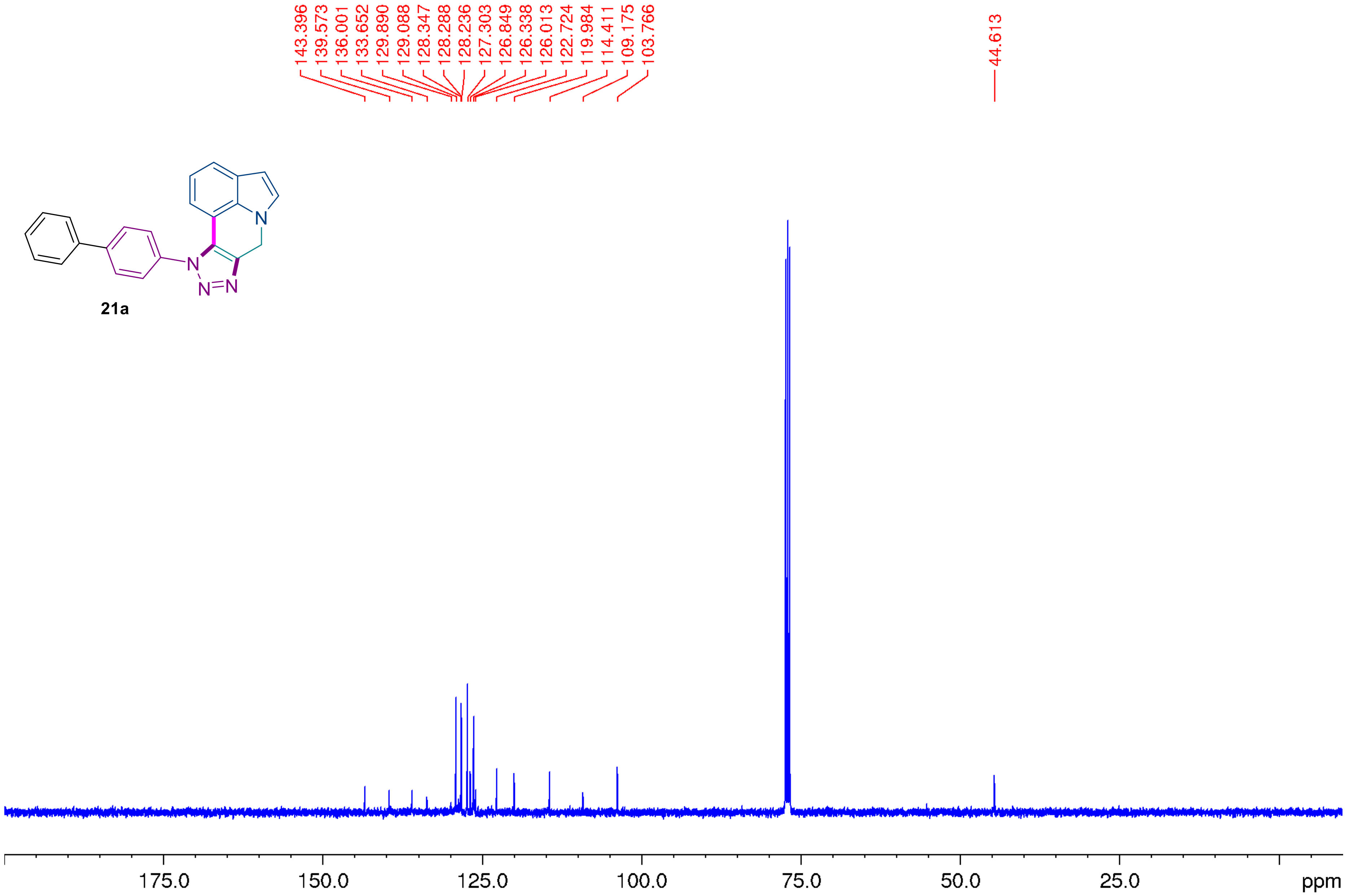

DEPT 135 NMR-spectrum ($CDCl_3$)

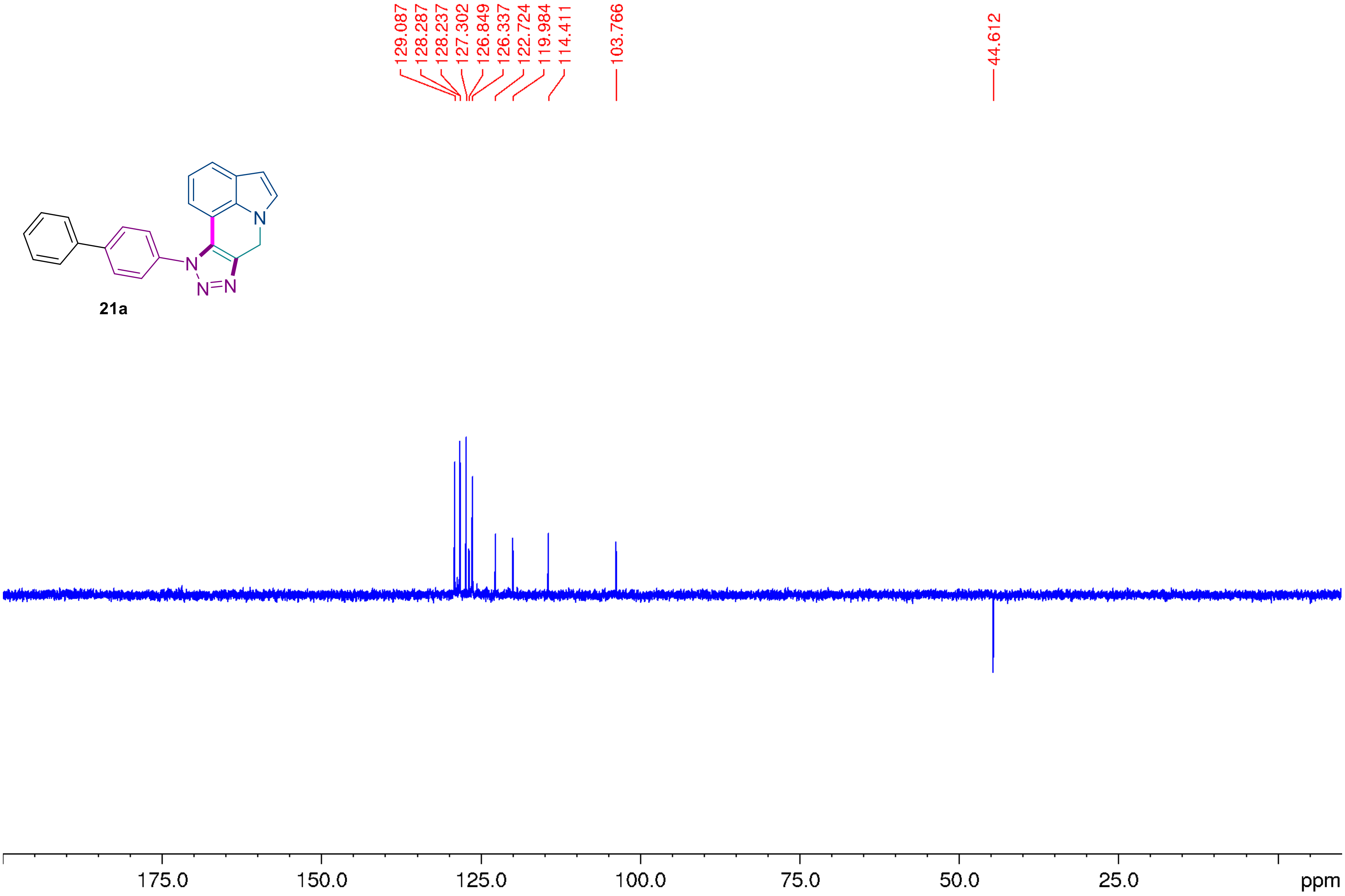

^1H NMR-spectrum (400 MHz, $CDCl_3$)

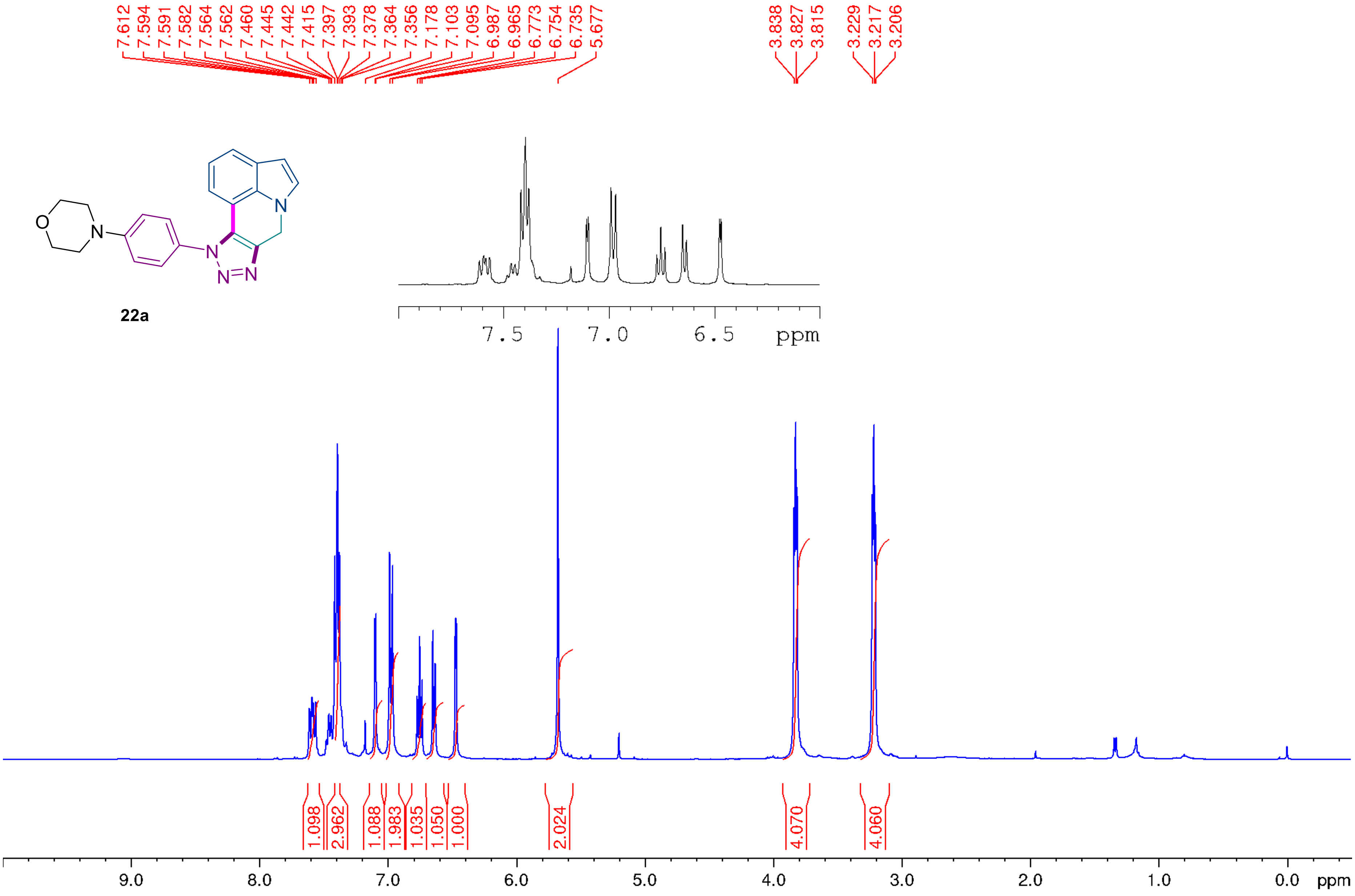

^{13}C NMR-spectrum (100 MHz, $CDCl_3$)

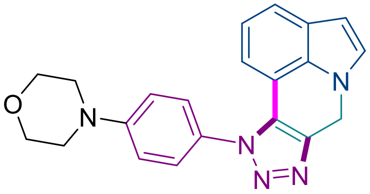

22a

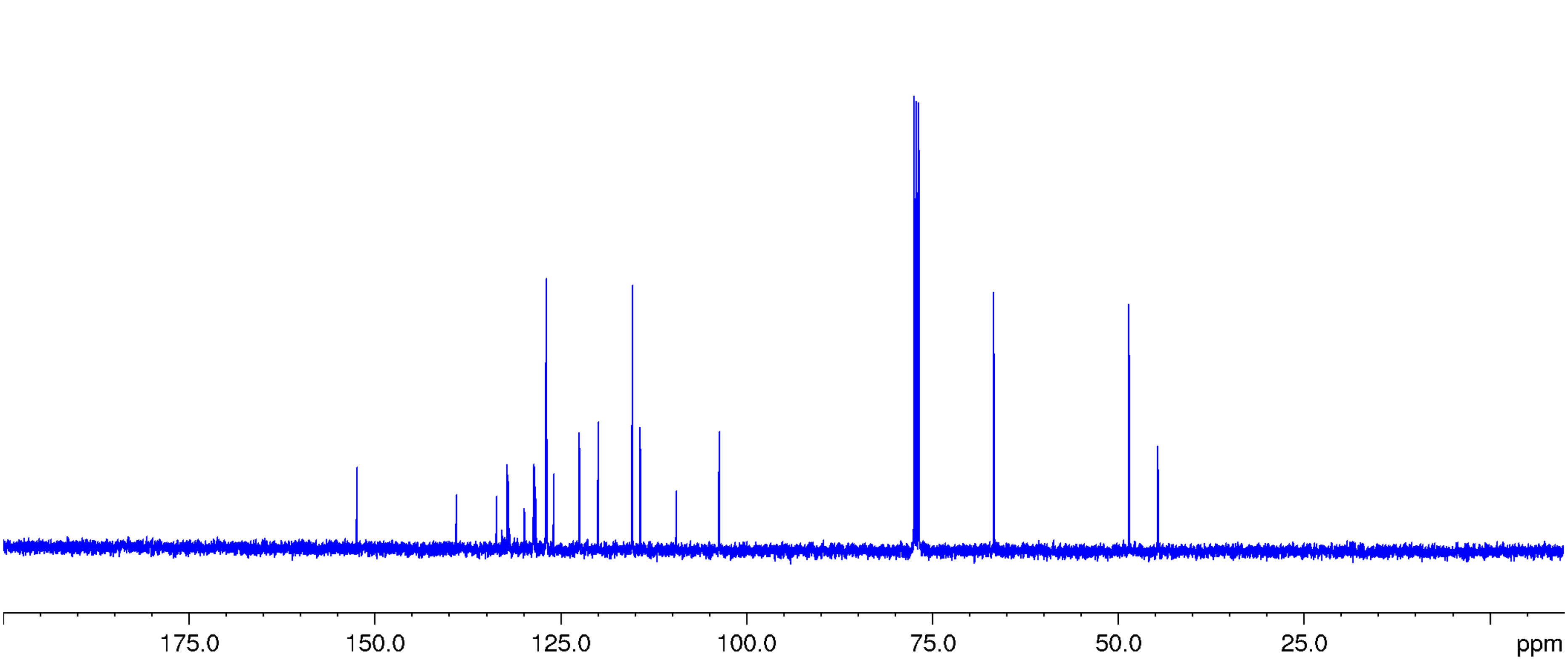

DEPT 135 NMR-spectrum ($CDCl_3$)

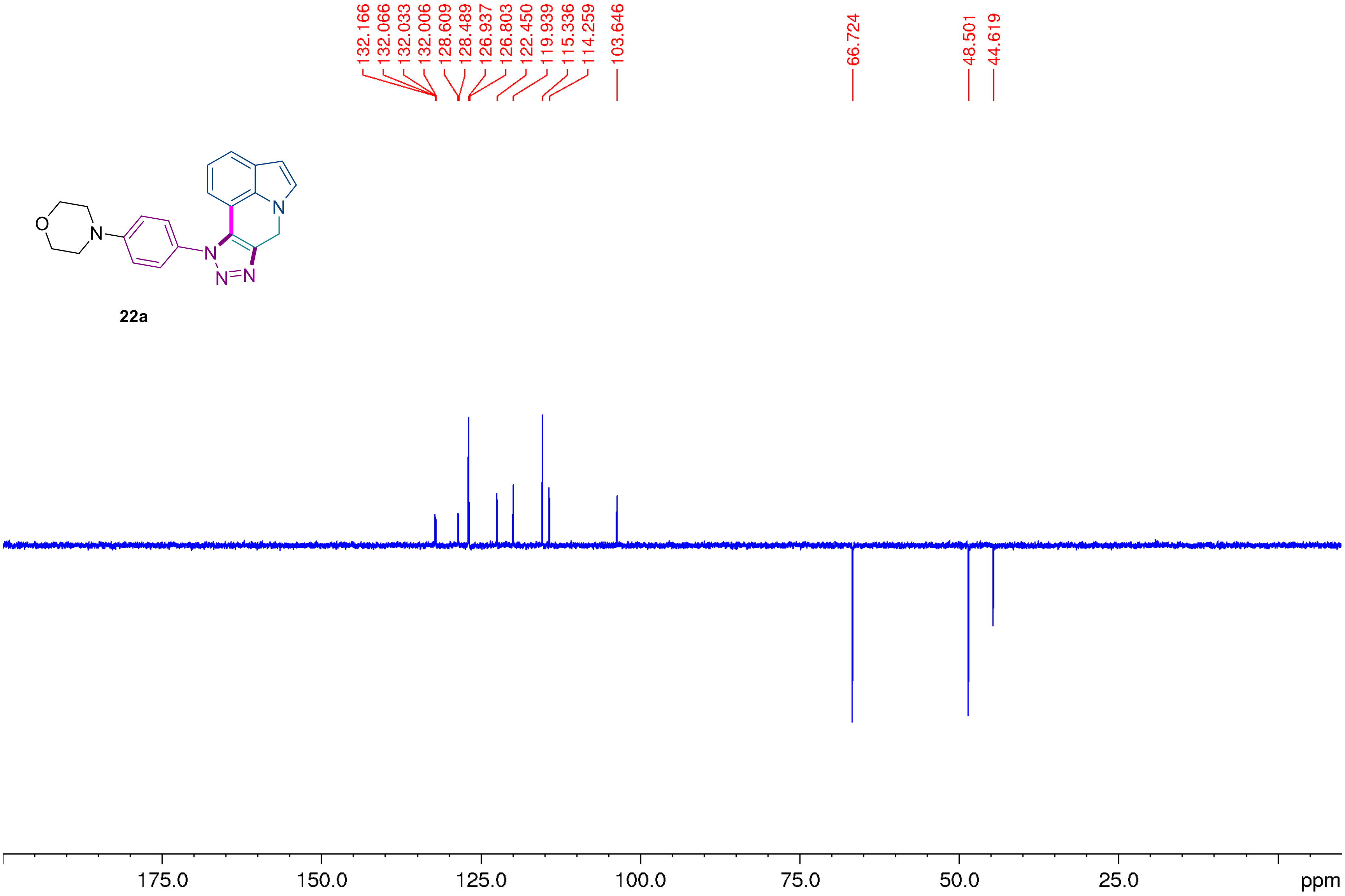

^1H NMR-spectrum (400 MHz, $CDCl_3$)

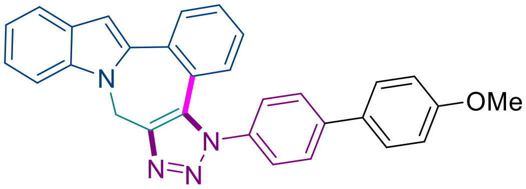

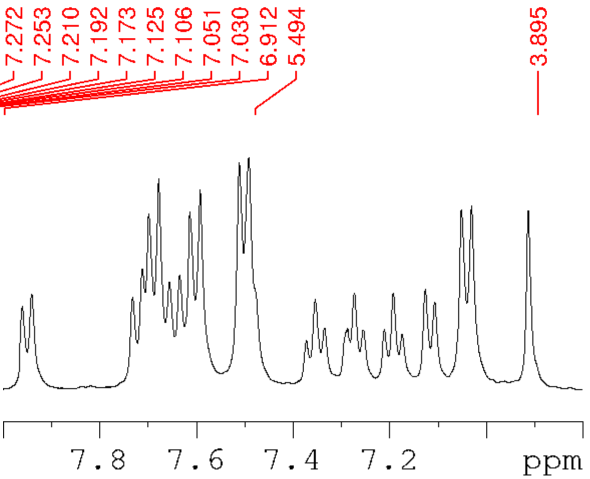

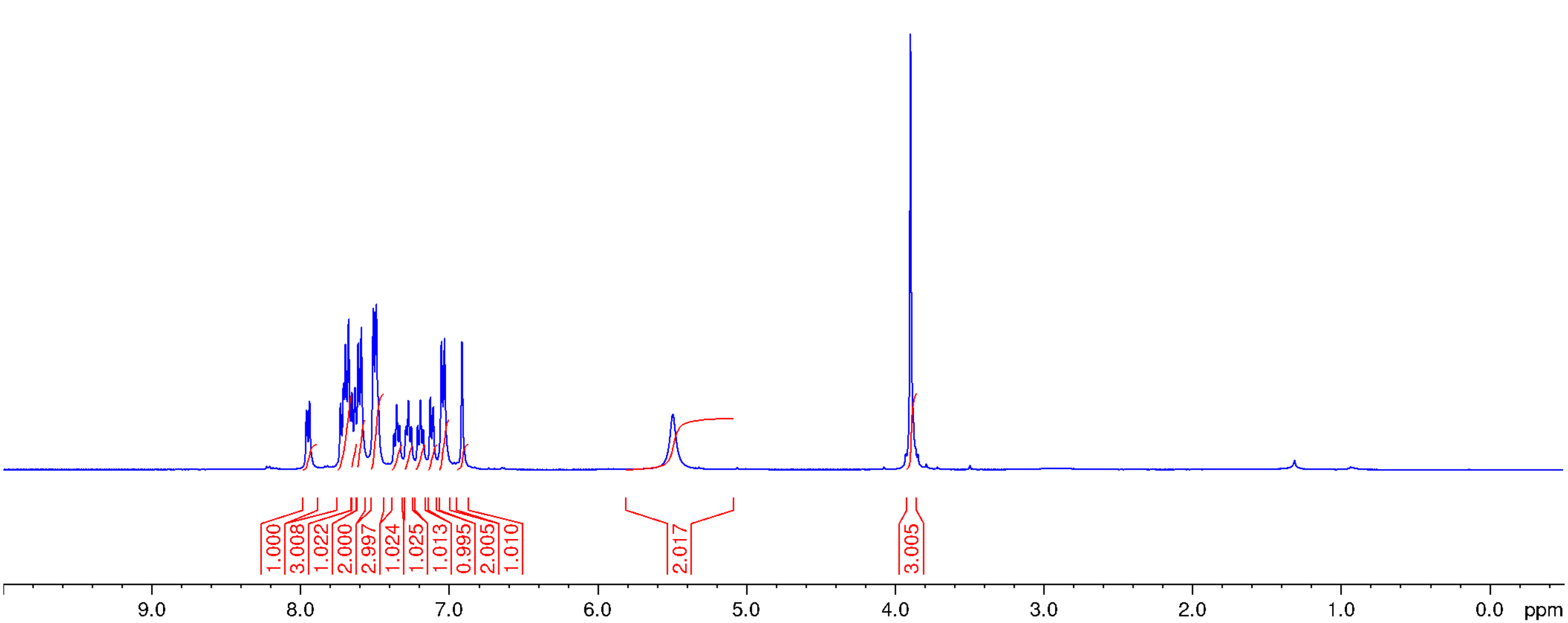

^{13}C NMR-spectrum (100 MHz, $CDCl_3$)

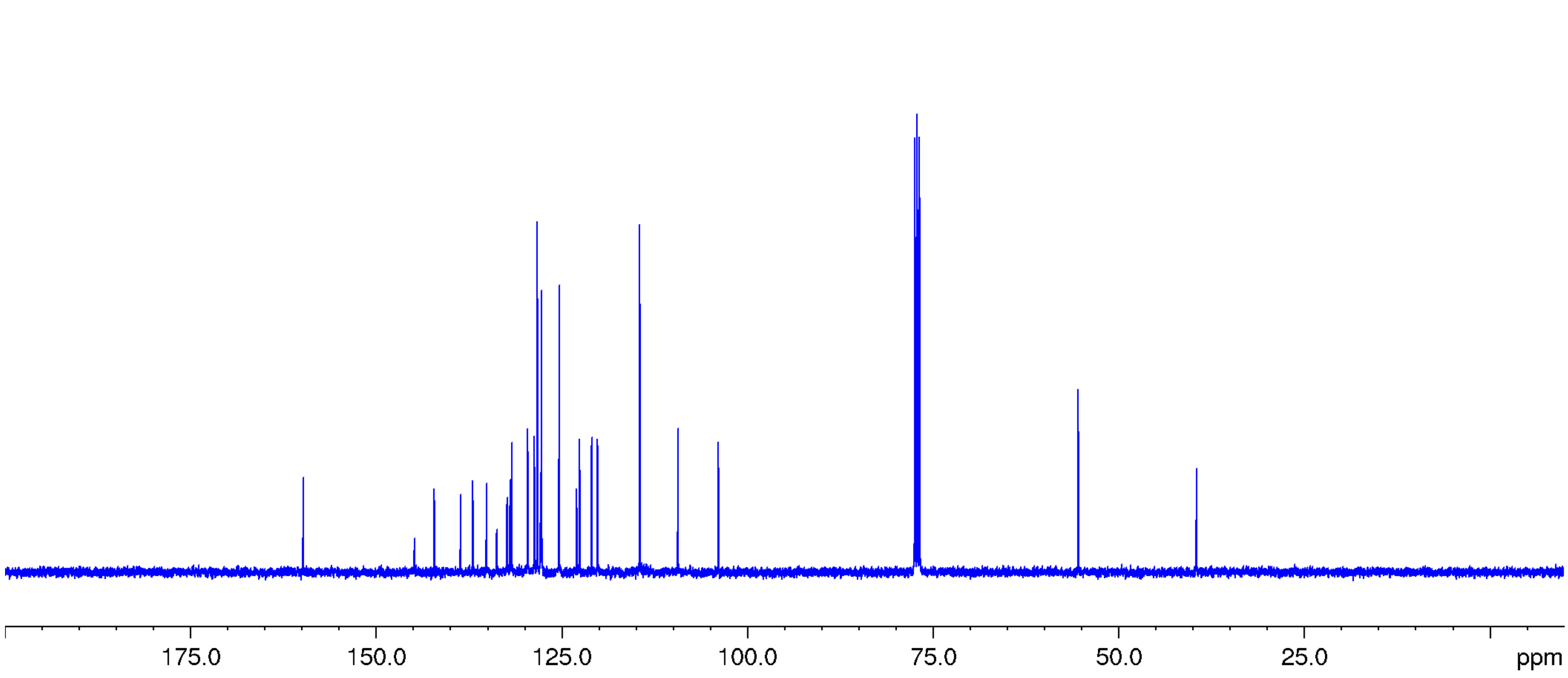

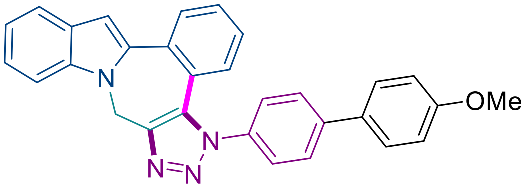

23a

DEPT 135 NMR-spectrum ($CDCl_3$)

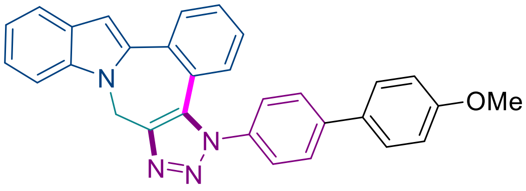

23a

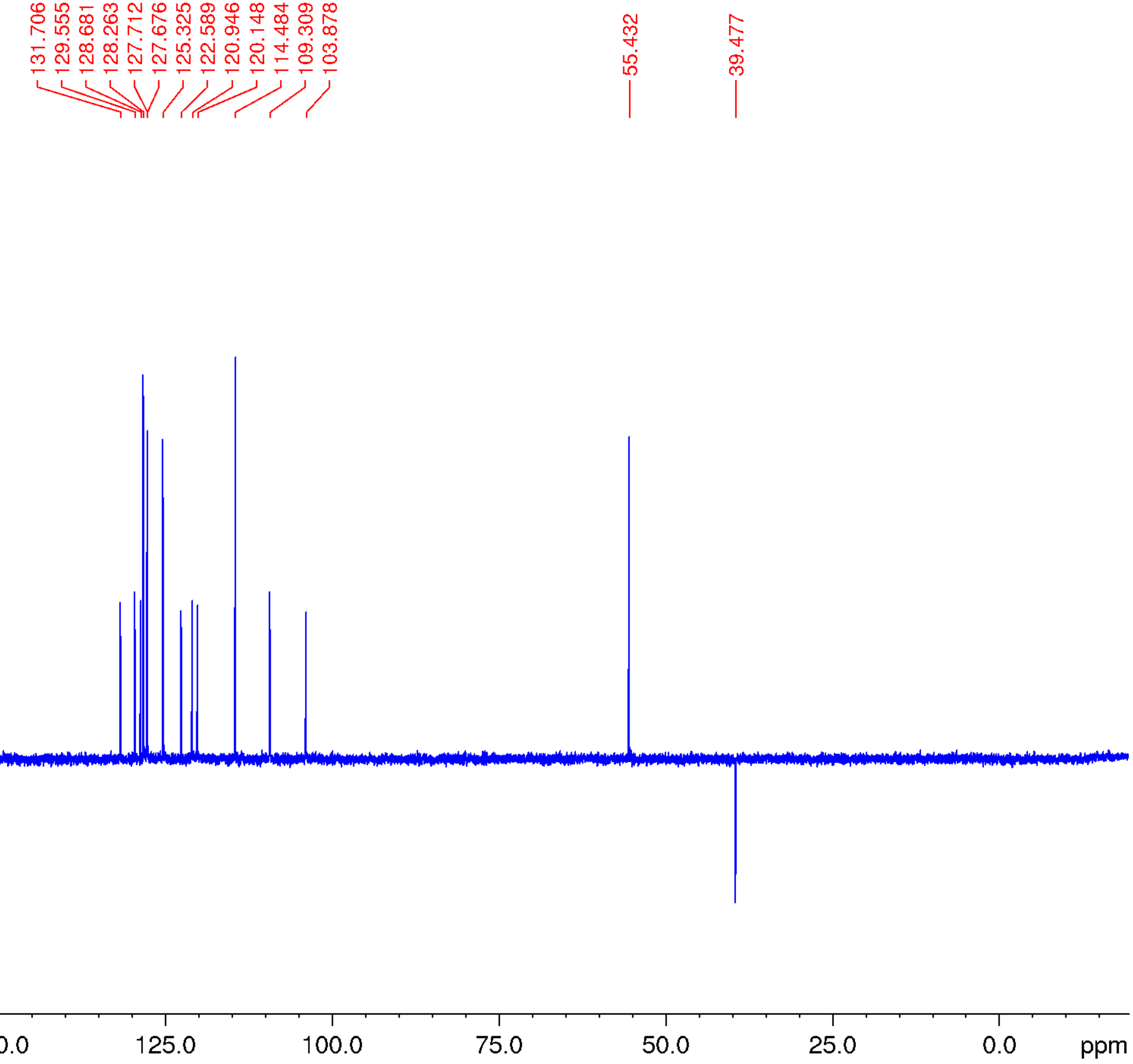

^1H NMR-spectrum (400 MHz, $CDCl_3$)

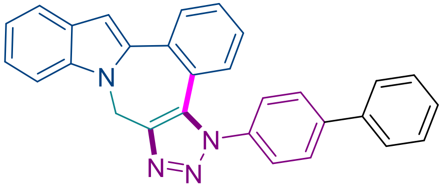

23b

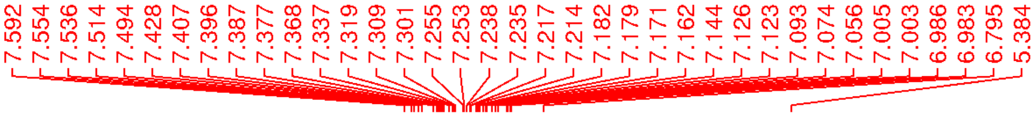

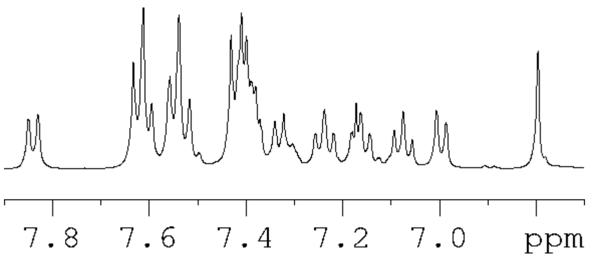

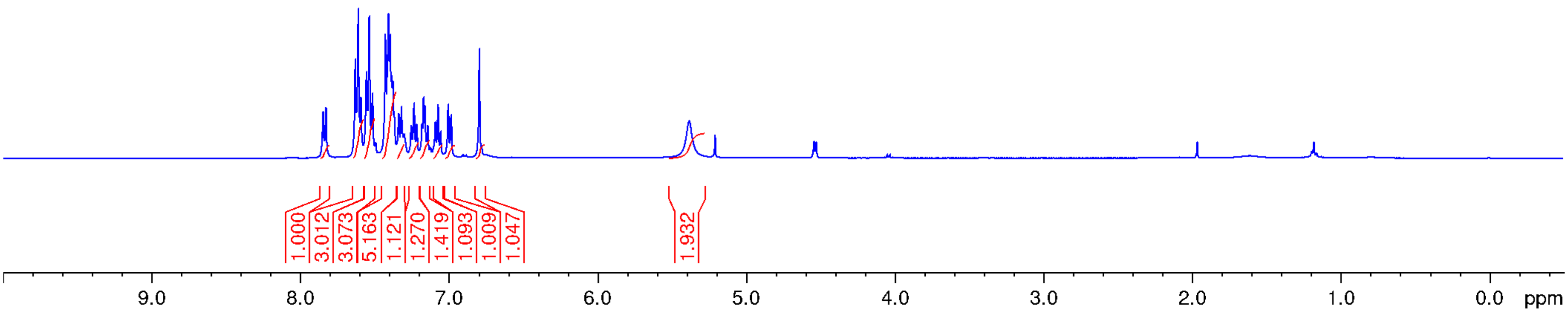

^{13}C NMR-spectrum (100 MHz, $CDCl_3$)

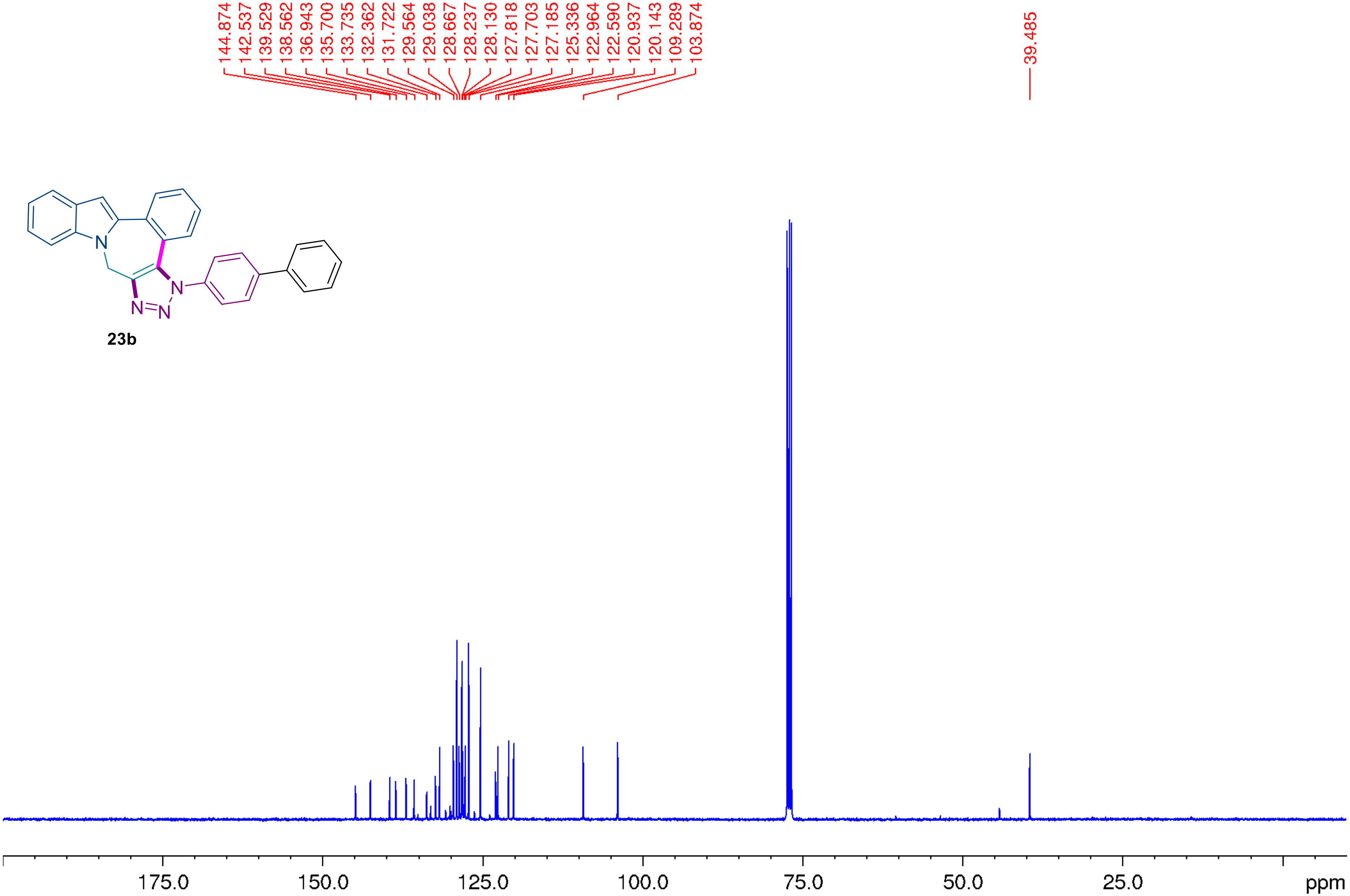

23b

DEPT 135 NMR-spectrum ($CDCl_3$)

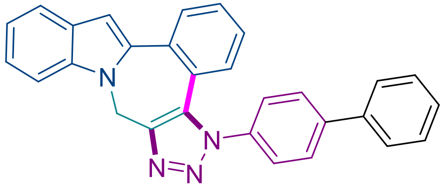

23b

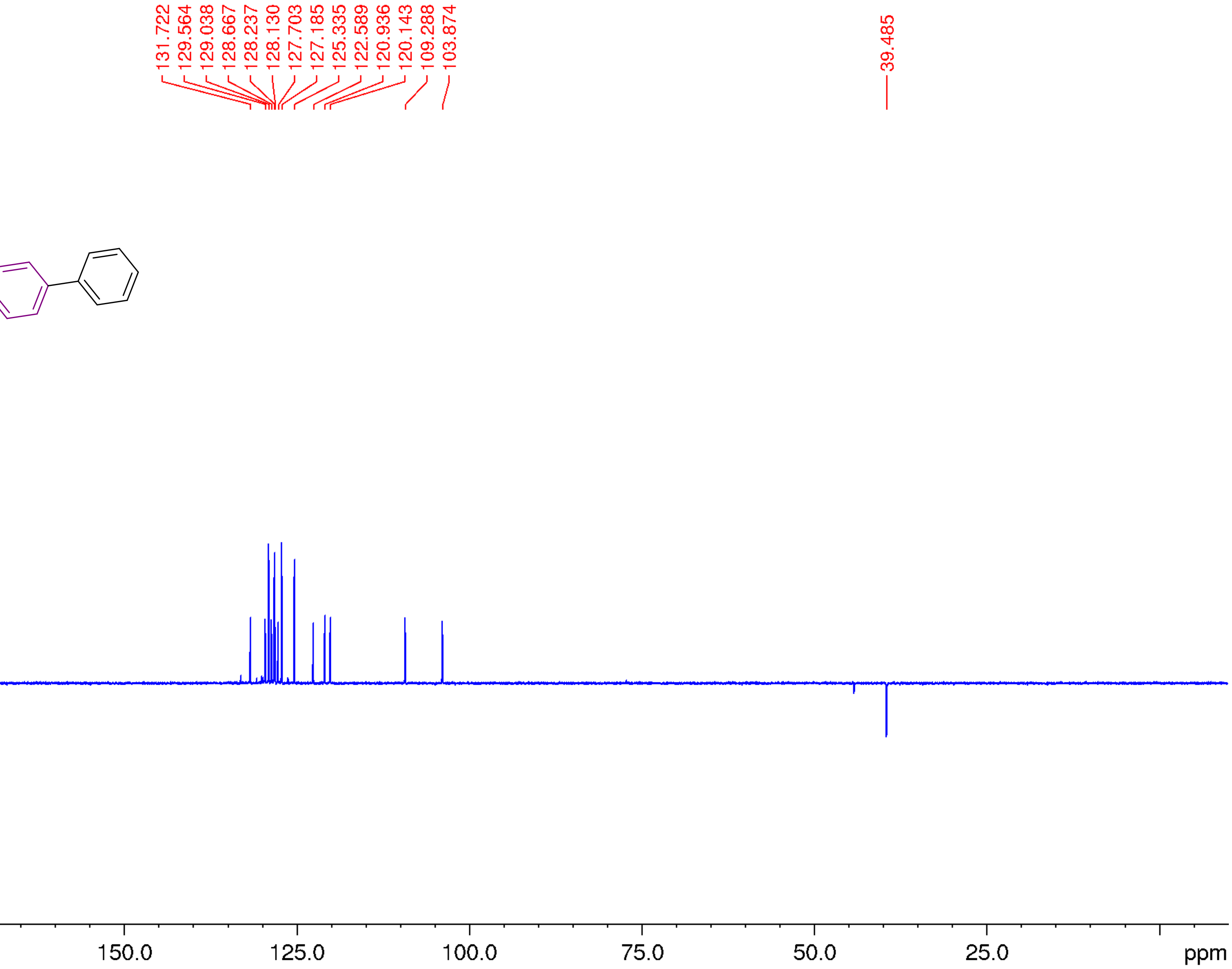

^{1}H NMR-spectrum (400 MHz, $CDCl_3$)

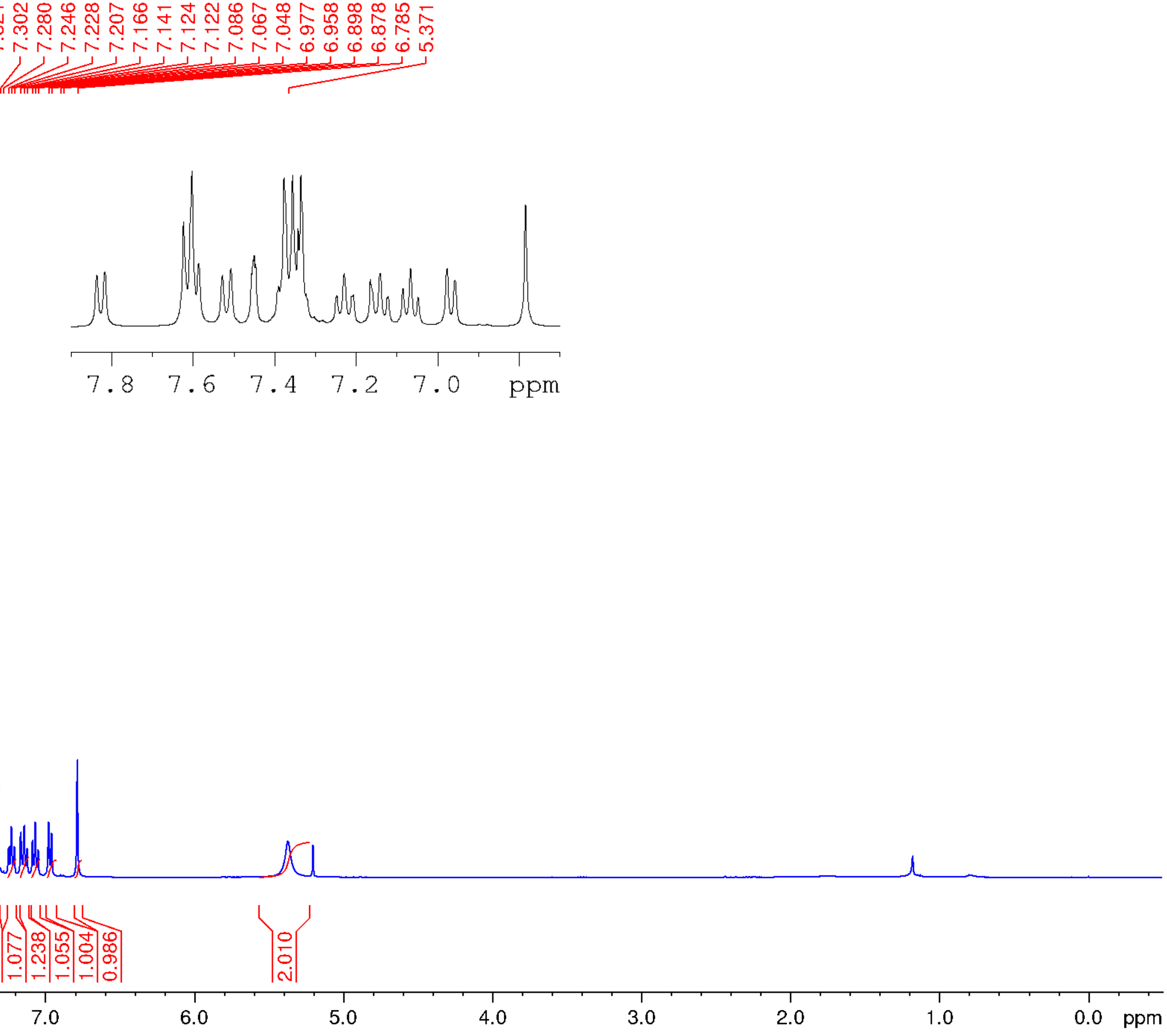

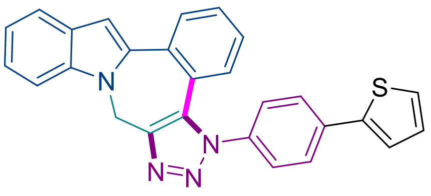

23c

^{13}C NMR-spectrum (100 MHz, $CDCl_3$)

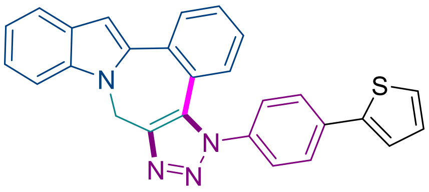

23c

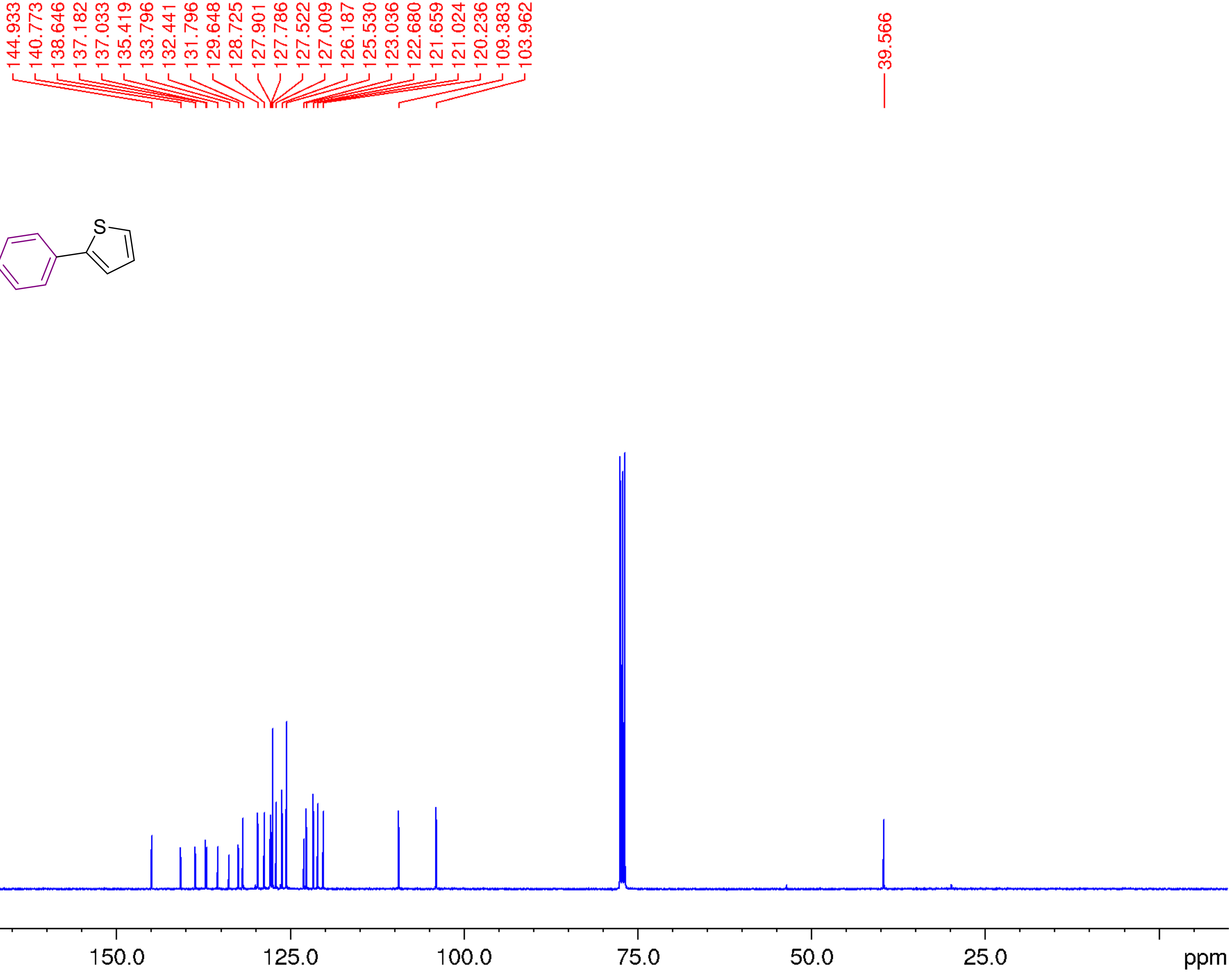

DEPT 135 NMR-spectrum ($CDCl_3$)

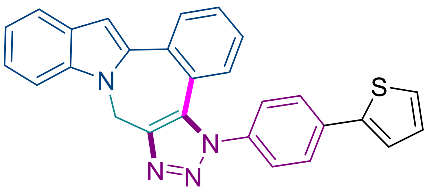

23c

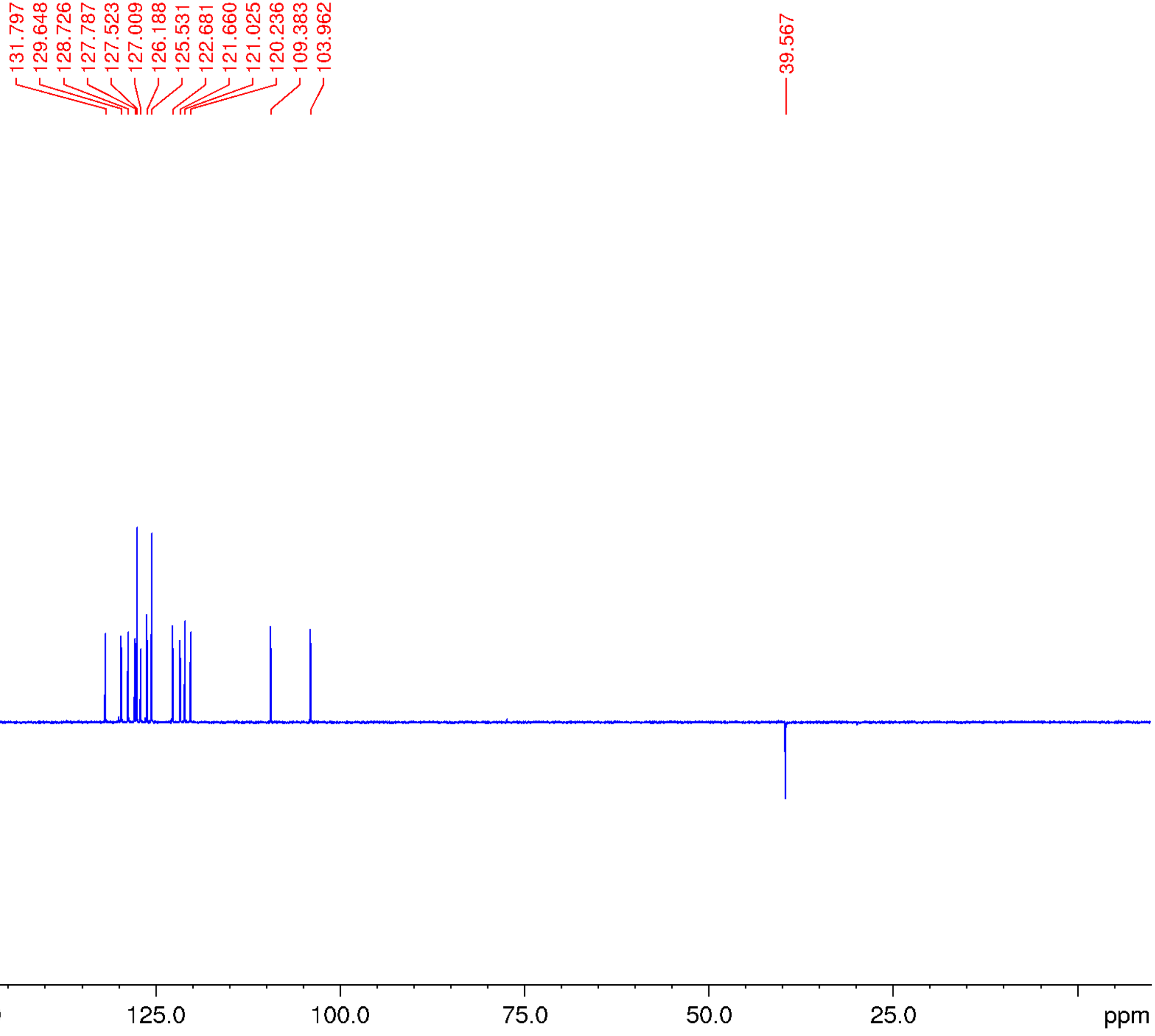

^{1}H NMR-spectrum (400 MHz, $CDCl_3$)

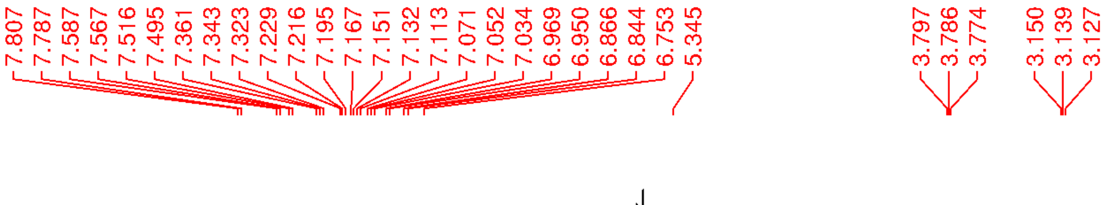

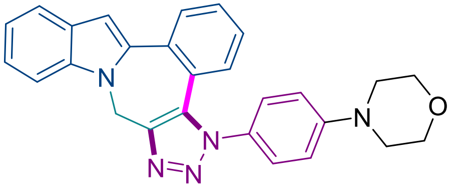

24a

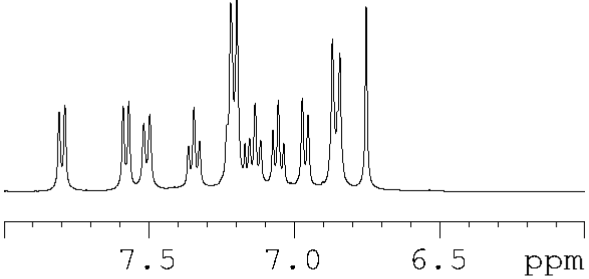

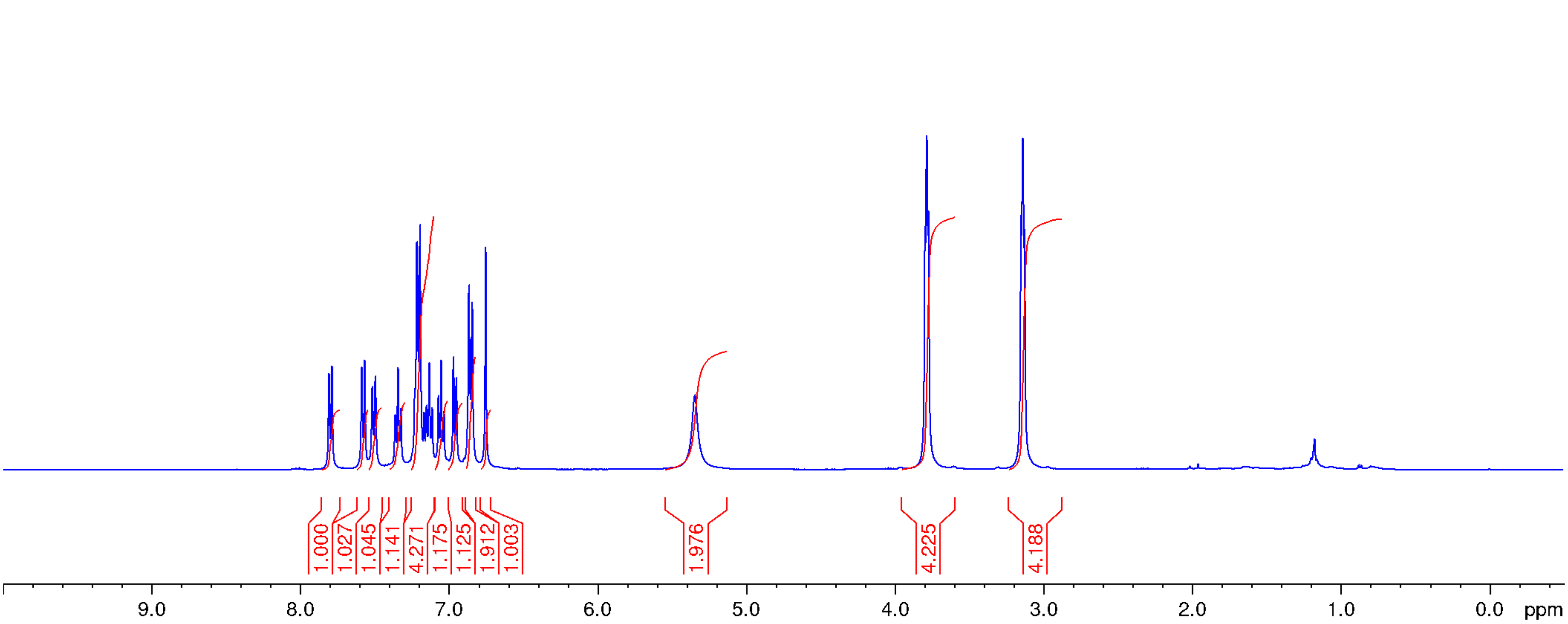

^{13}C NMR-spectrum (100 MHz, $CDCl_3$)

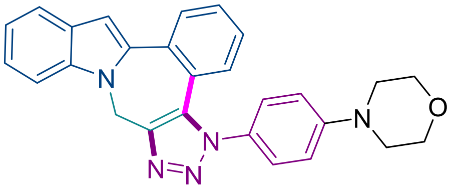

24a

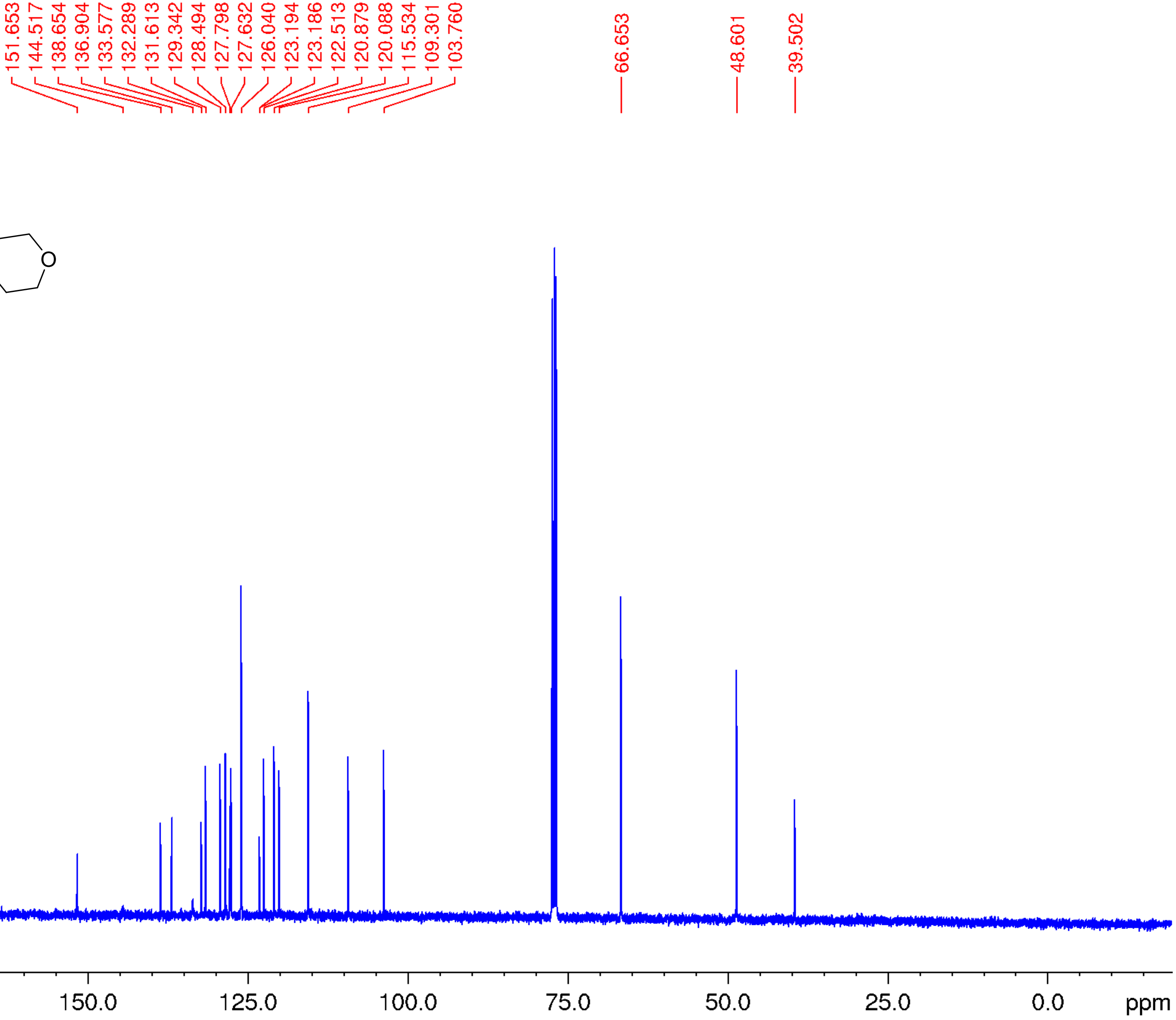

DEPT 135 NMR-spectrum ($CDCl_3$)

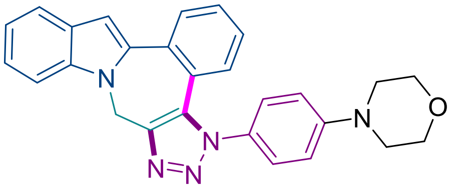

24a

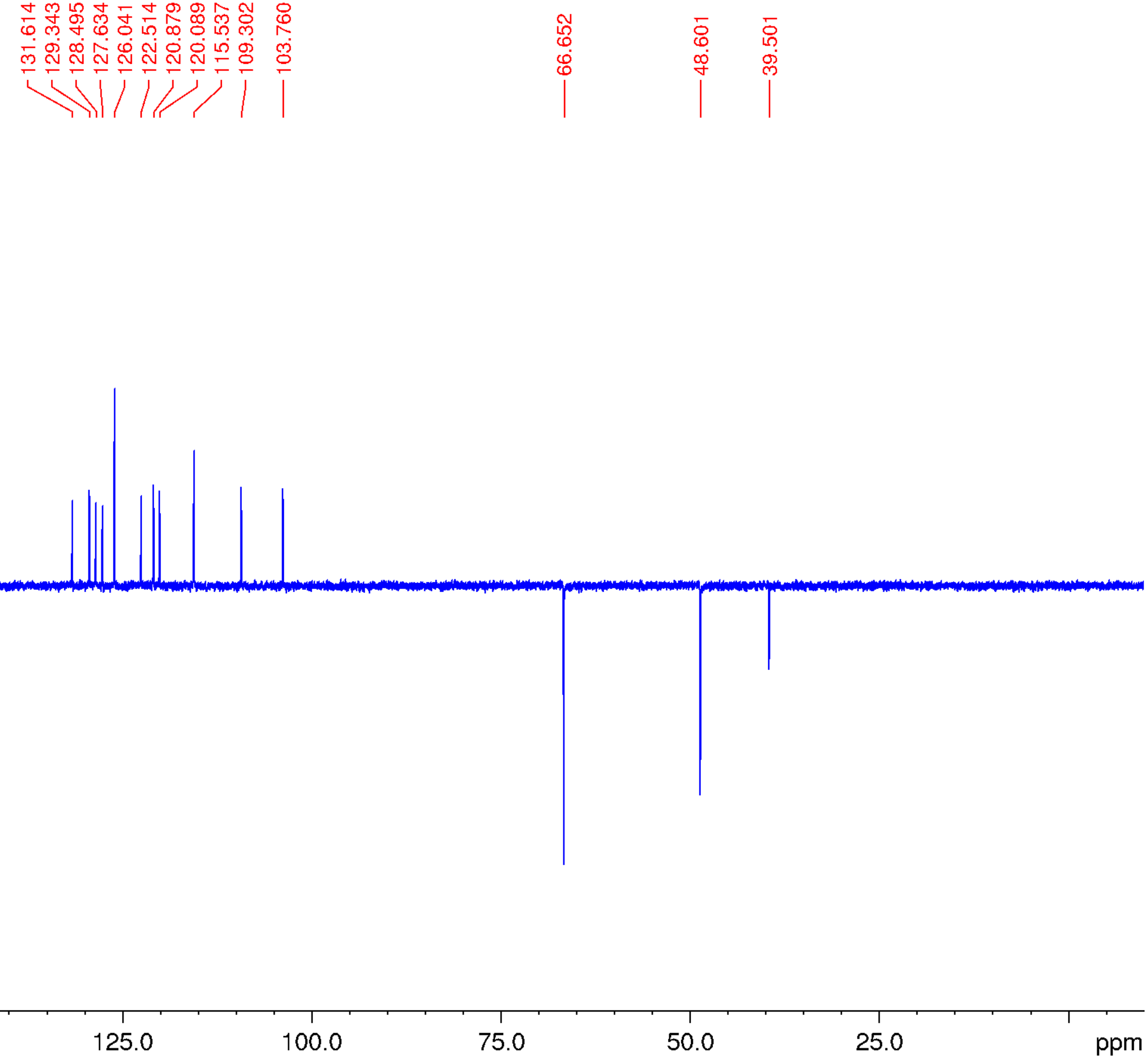

^{1}H NMR-spectrum (400 MHz, $CDCl_3$)

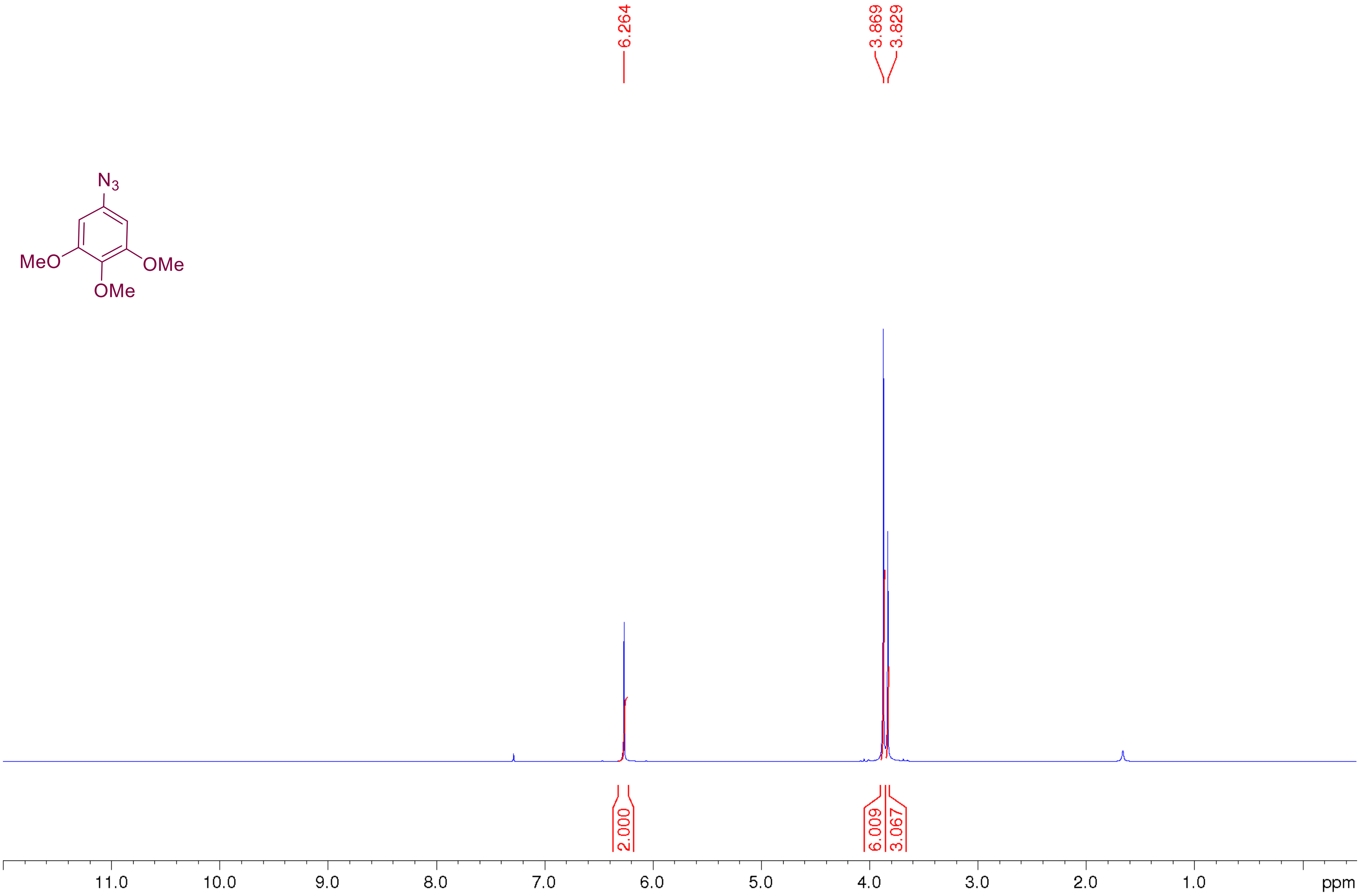

^{13}C NMR-spectrum (100 MHz, $CDCl_3$)

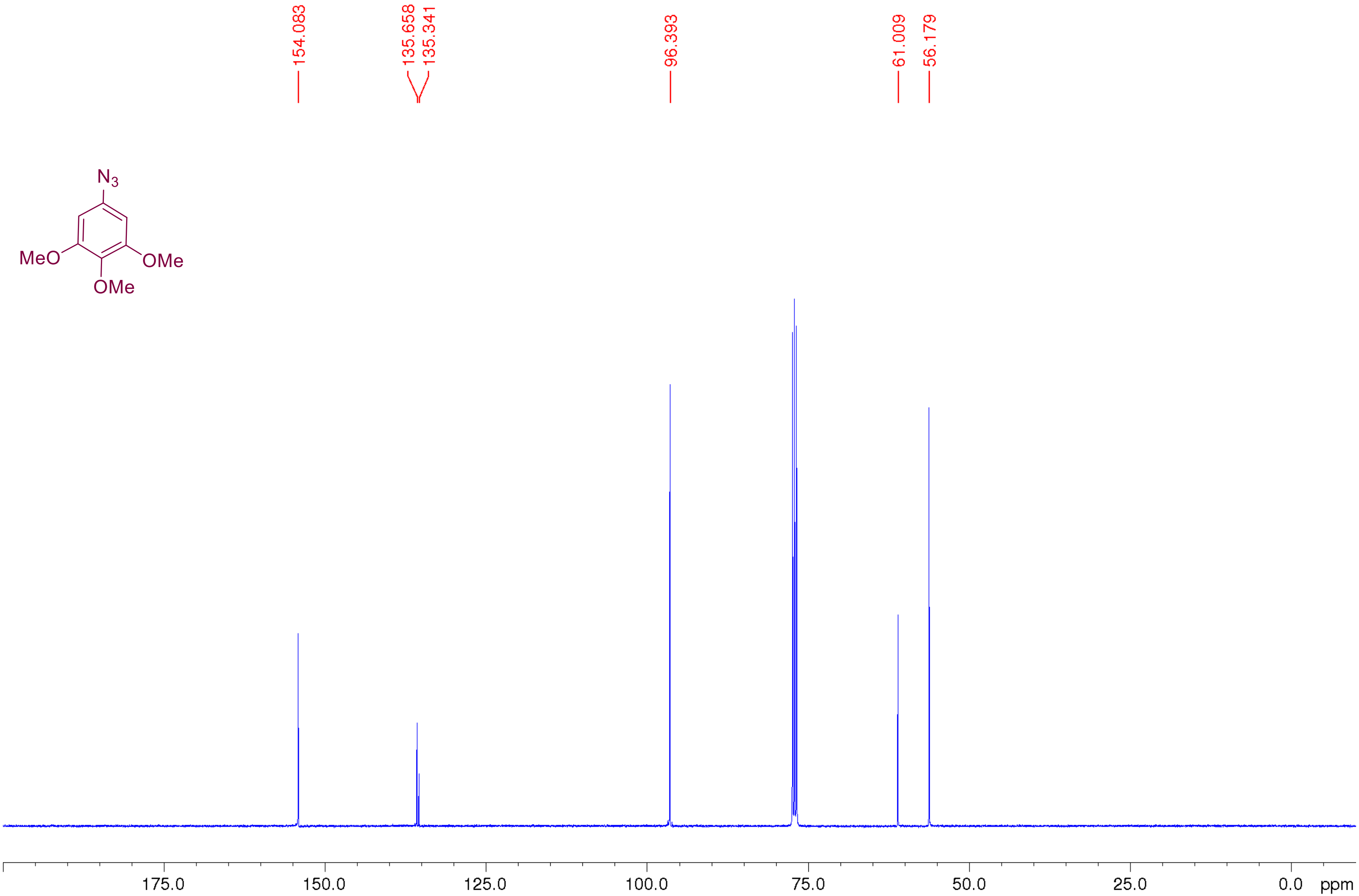

DEPT 135 NMR-spectrum ($CDCl_3$)

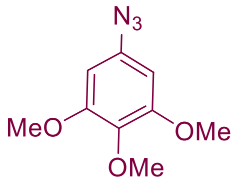

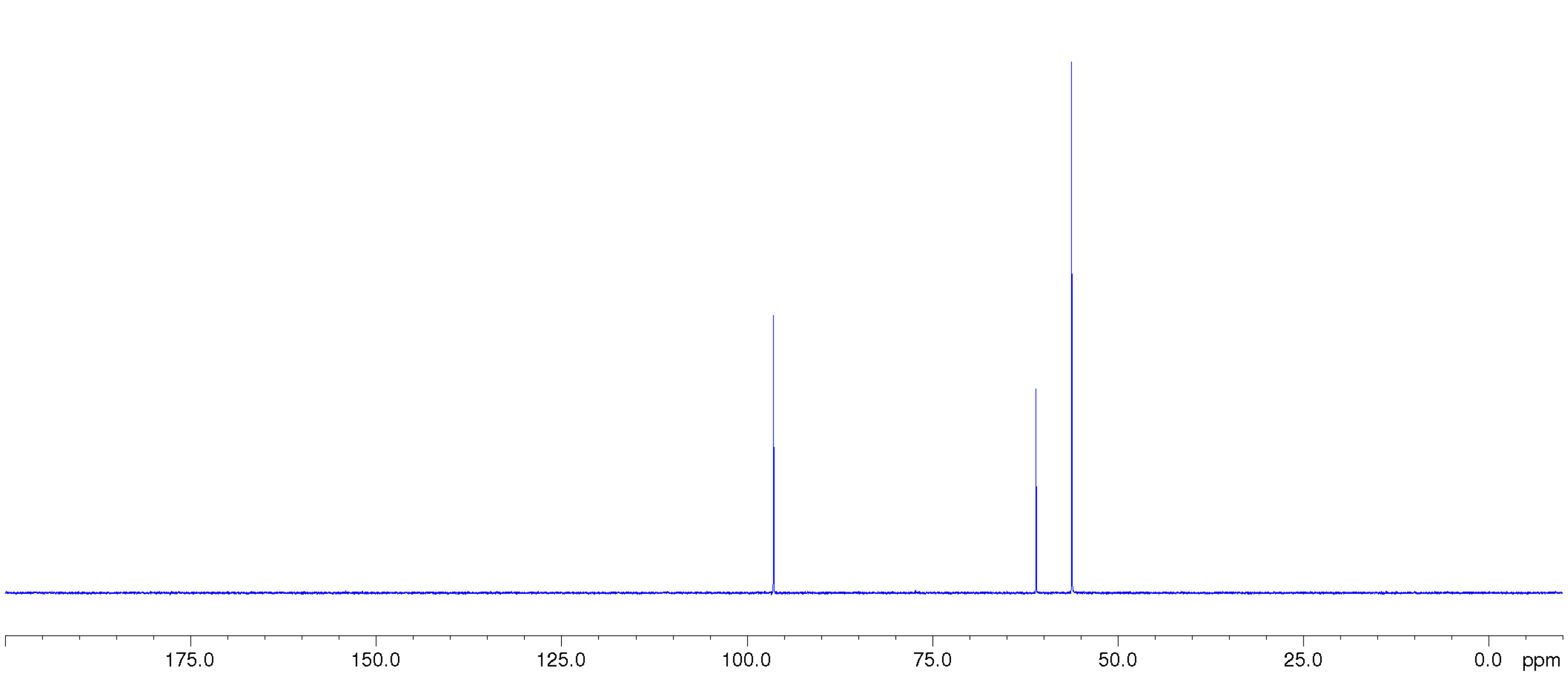

^{1}H NMR-spectrum (400 MHz, $CDCl_3$)

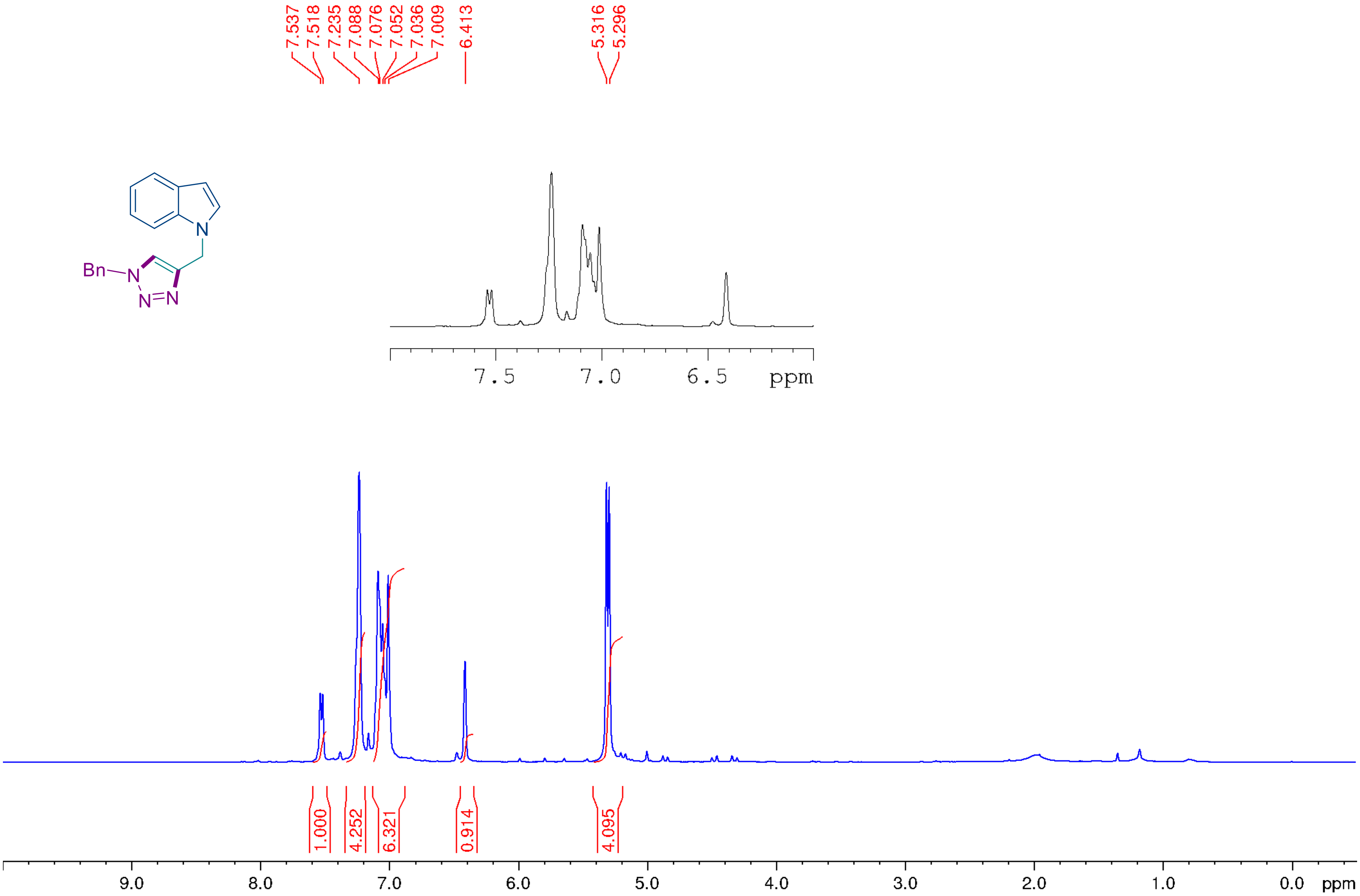

^{13}C NMR-spectrum (100 MHz, $CDCl_3$)

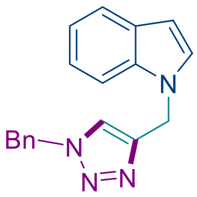

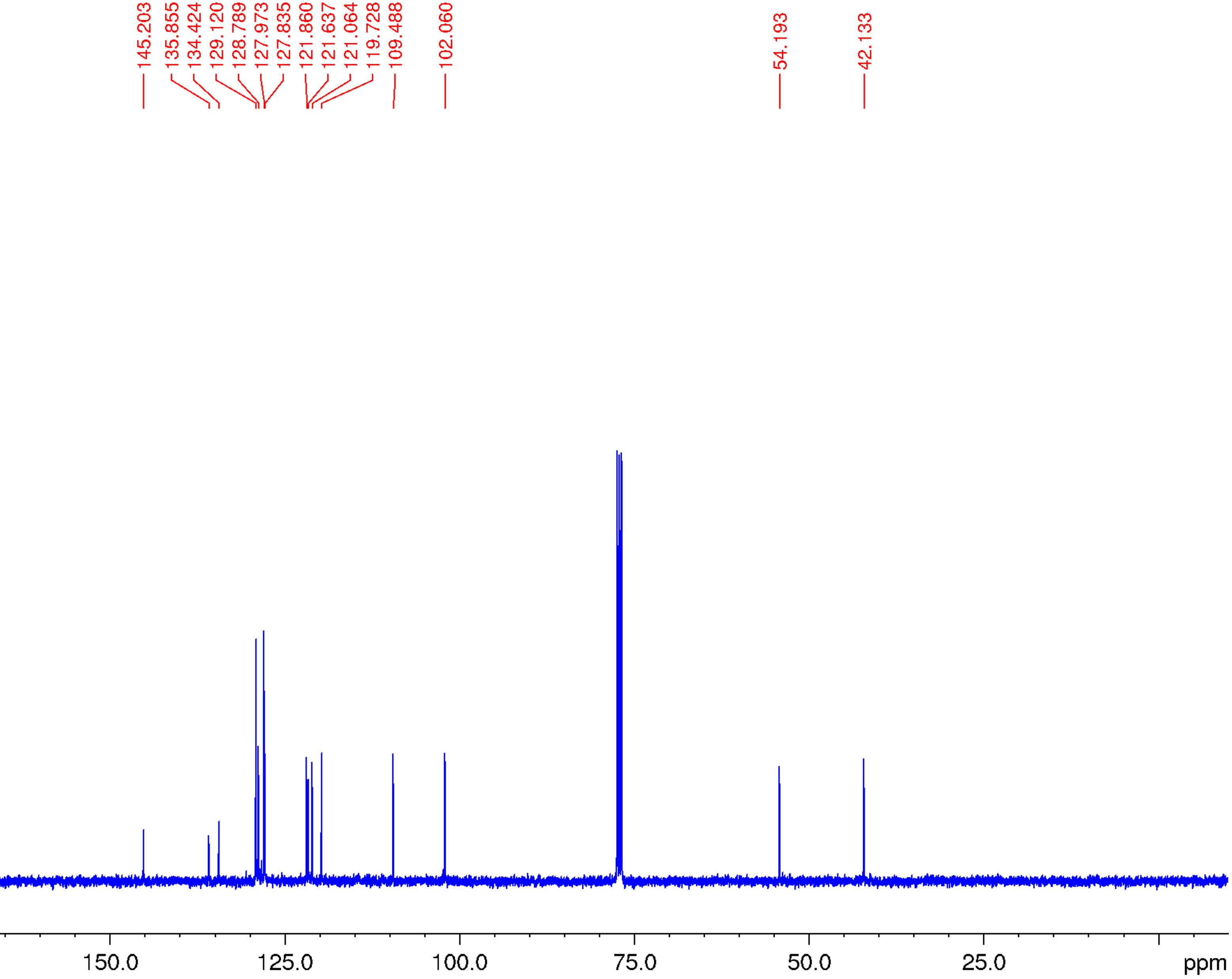

DEPT 135 NMR-spectrum ($CDCl_3$)

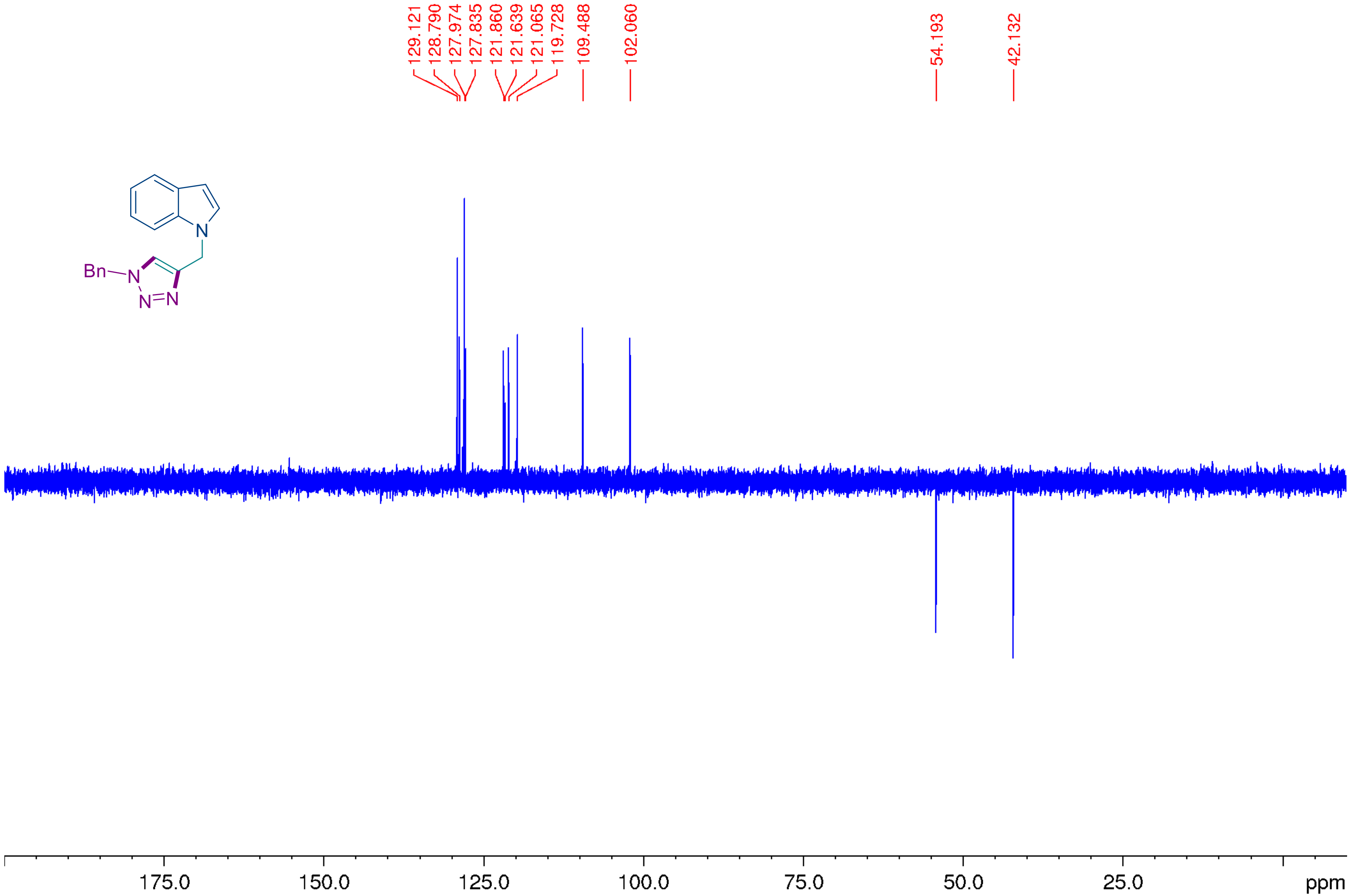

Supplement: Supplementary file 1 [file molecules-30-02588-s001.zip › molecules-3684956-supplementary.pdf]
